# Supplementary material for: BRCA1 preserves genome integrity during the formation of undifferentiated spermatogonia
Source: EMBO Rep. 2025 May 28;26(15):3747–72. doi: 10.1038/s44319-025-00487-5 (PMC12332178; doi:10.1038/s44319-025-00487-5)
Supplement: Supplementary file 1 — Appendix [file 44319_2025_487_MOESM1_ESM.pdf]

Appendix

Table of Contents

Page 2: **Appendix Figure S1.** Differential gene expression and pathway enrichment analysis of SPG1, SPG2, and SPG3 between control and *Brca1* vKO testes at PD7.

Page 3: **Appendix Figure S2.** GFRα1-positive spermatogonial stem cells continue to hyper-proliferate upon BRCA1 loss in mouse testes at PD14 and PD21.

Page 4-5: **Appendix Figure S3.** Apoptotic analysis of GFRα1+ and SOX3+ cells in testes of control and *Brca1* vKO mice at PD7, PD14, and PD21.

Page 6: **Appendix Figure S4.** DNA replication rate analysis of Sertoli cells in WT male mice at PD4 and PD7.

Page 7-8: **Appendix Figure S5.** *Aldh2* KO exacerbates the defects in the formation of undifferentiated spermatogonia in *Brca1* vKO testes.

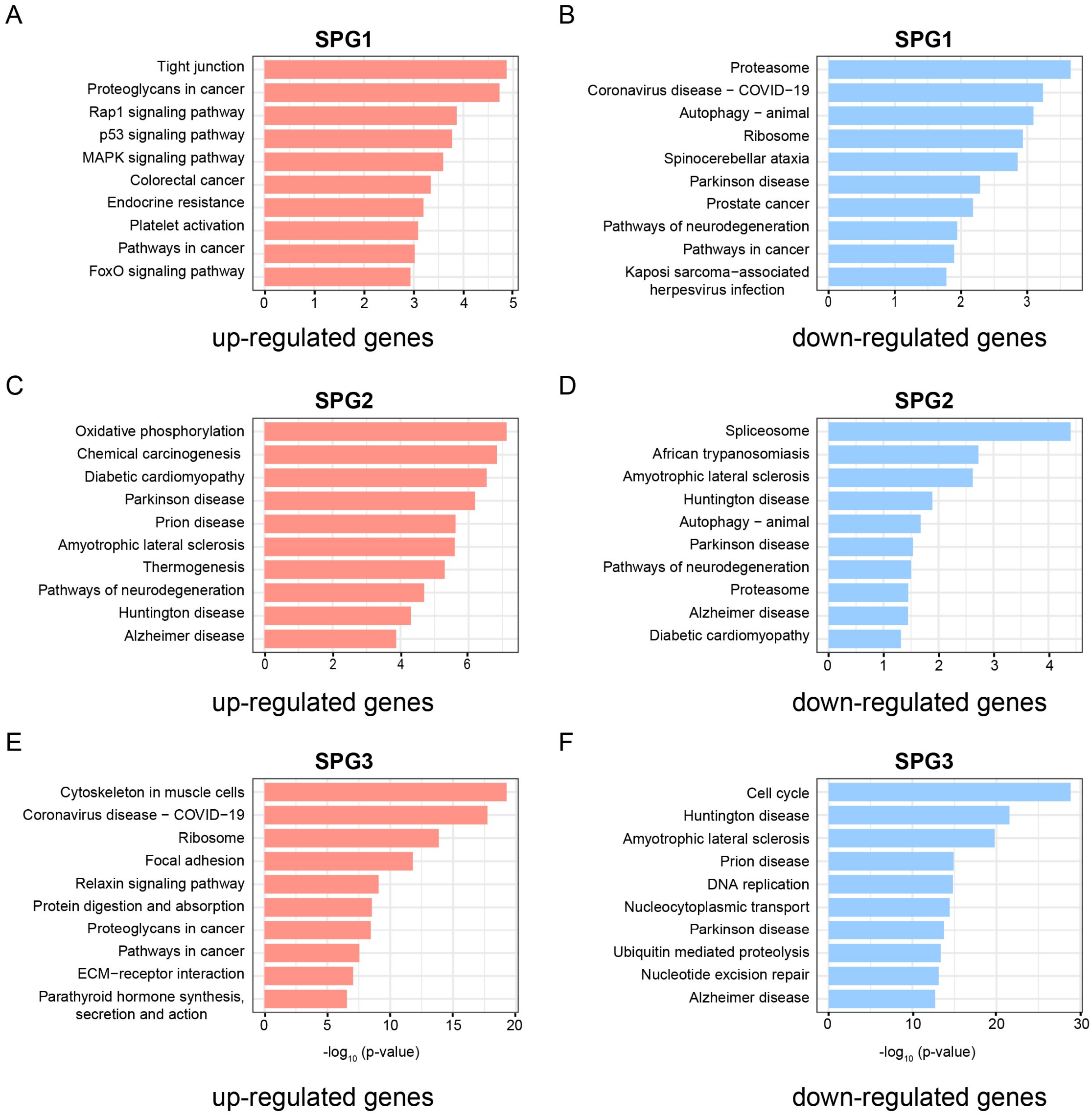

Appendix Figure S1. Differential gene expression and pathway enrichment analysis of SPG1, SPG2, and SPG3 between control and *Brca1* vKO testes at PD7.

A-B. KEGG pathway enrichment analysis of significantly up-regulated genes (A) and down-regulated genes (B) in SPG1.

C-D. KEGG pathway enrichment analysis of significantly up-regulated genes (C) and down-regulated genes (D) in SPG2.

E-F. KEGG pathway enrichment analysis of significantly up-regulated genes (E) and down-regulated genes (F) in SPG3.

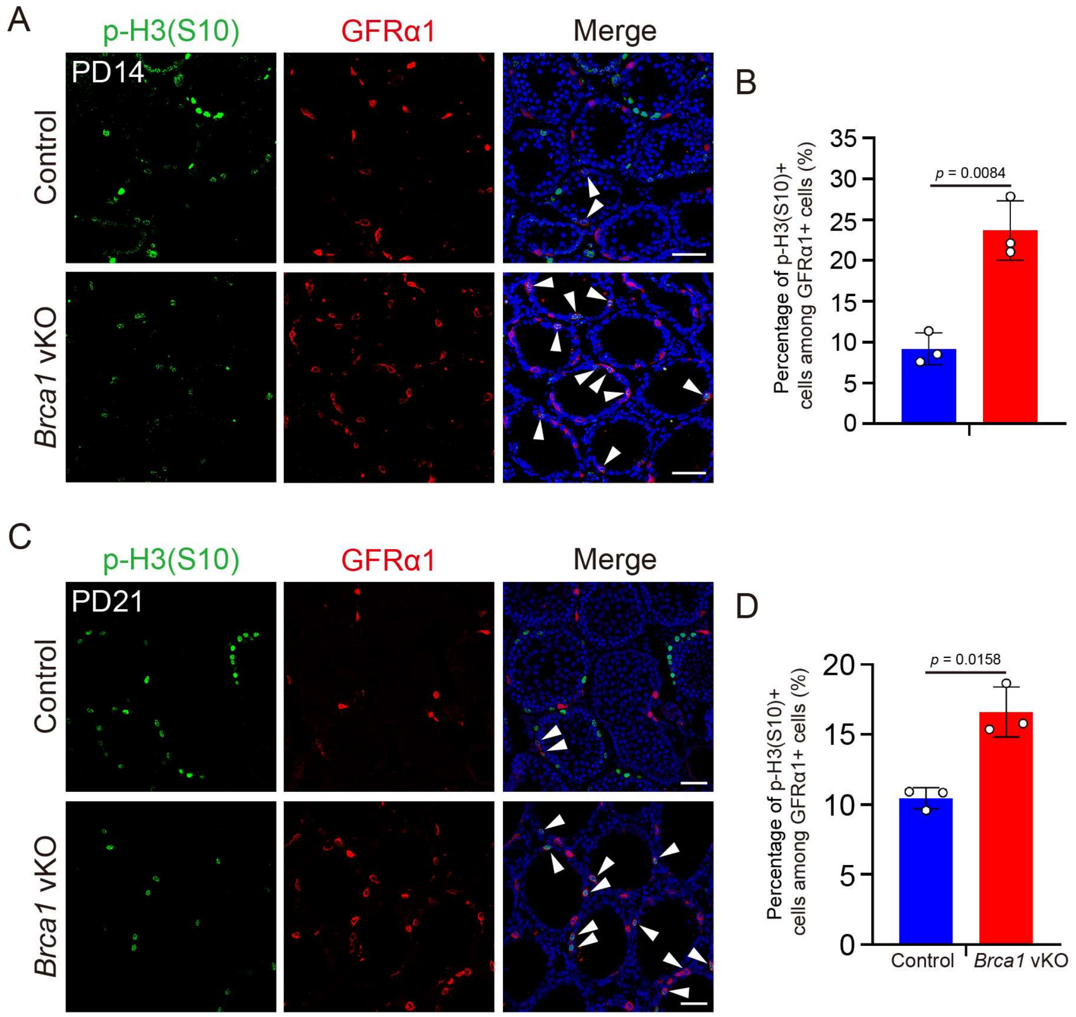

Appendix Figure S2. GFRα1-positive spermatogonial stem cells continue to hyper-proliferate upon BRCA1 loss in mouse testes at PD14 and PD21.

A. IF staining of the mitosis marker p-H3(S10) and GFRα1 in frozen sections of testes from Control and *Brca1* vKO male mice at PD14. The double-positive cells were marked by white arrowhead. Scale bar, 50 μm

B. Statistical analysis of the percentage of p-H3(S10)+ cells among GFRα1+ cells in testes of Control and *Brca1* vKO male mice at PD14.

C. IF staining of the mitosis marker p-H3(S10) and GFRα1 in frozen sections of testes from Control and *Brca1* vKO male mice at PD21. The double-positive cells were marked by white arrowhead. Scale bar, 50 μm

D. Statistical analysis of the percentage of p-H3(S10)+ cells among GFRα1+ cells in testes of Control and *Brca1* vKO male mice at PD21.

Data are presented as mean ± SD. 3 mice of each genotype were analyzed. p value, two-tailed unpaired student’s t-test.

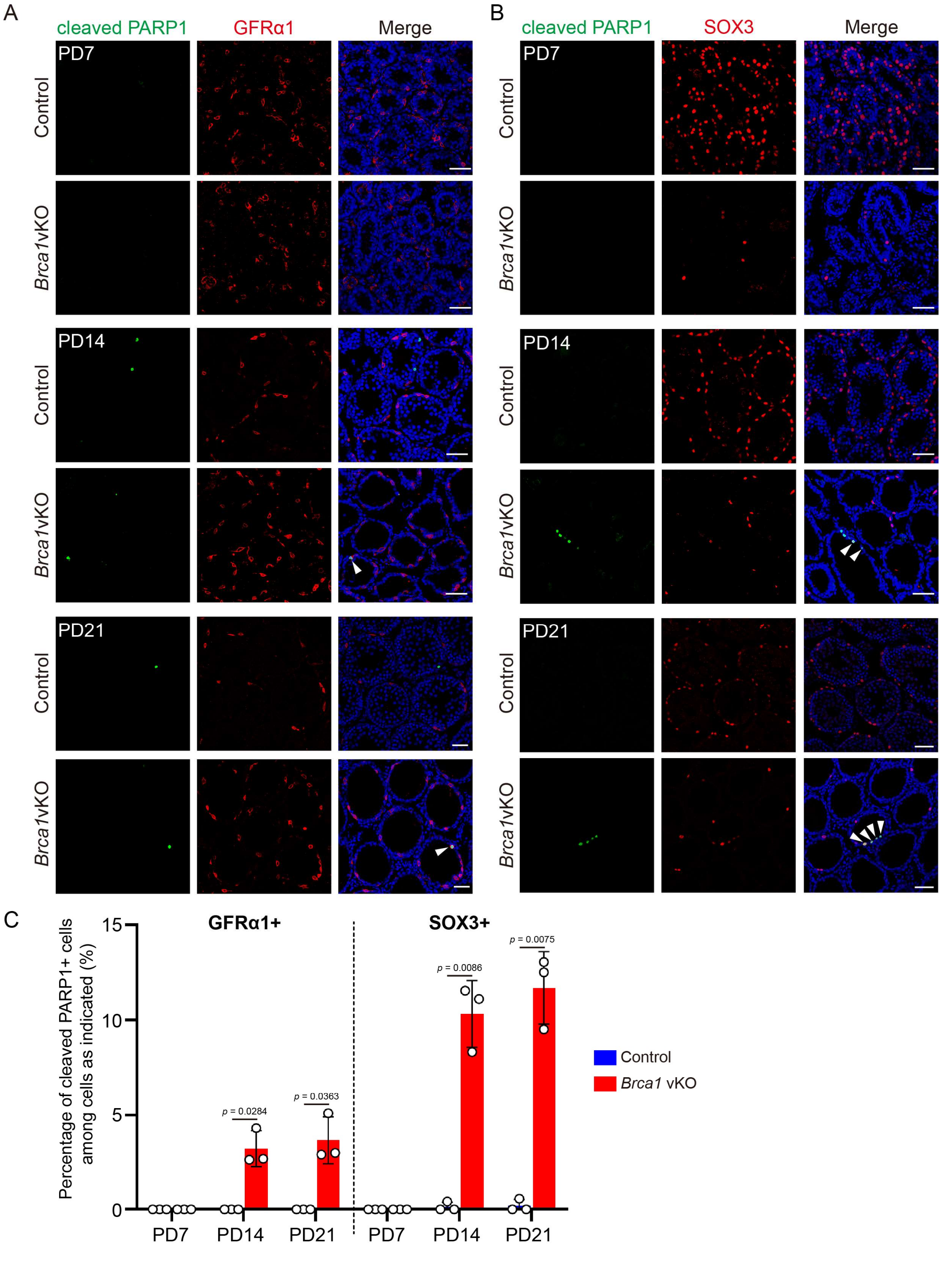

Appendix Figure S3. Apoptotic analysis of GFRα1+ and SOX3+ cells in testes of control and *Brca1* vKO mice at PD7, PD14, and PD21.

A-B. Representative images of cleaved PARP1 co-stained with GFRα1 (A) or SOX3 (B) in frozen sections of testes from control and *Brca1* vKO mice at PD7, PD14, and PD21. The double-positive cells were labeled by white arrowhead. Scale bar, 50 μm.

C. Statistical analysis of the percentage of GFRα1+ or SOX3+ cells positive for cleaved

PARP1 in testes from control and *Brca1* vKO mice at PD7, PD14, and PD21. Data are presented as mean ± SD. 3 mice of each genotype were analyzed. p value, two-tailed unpaired student's t-test.

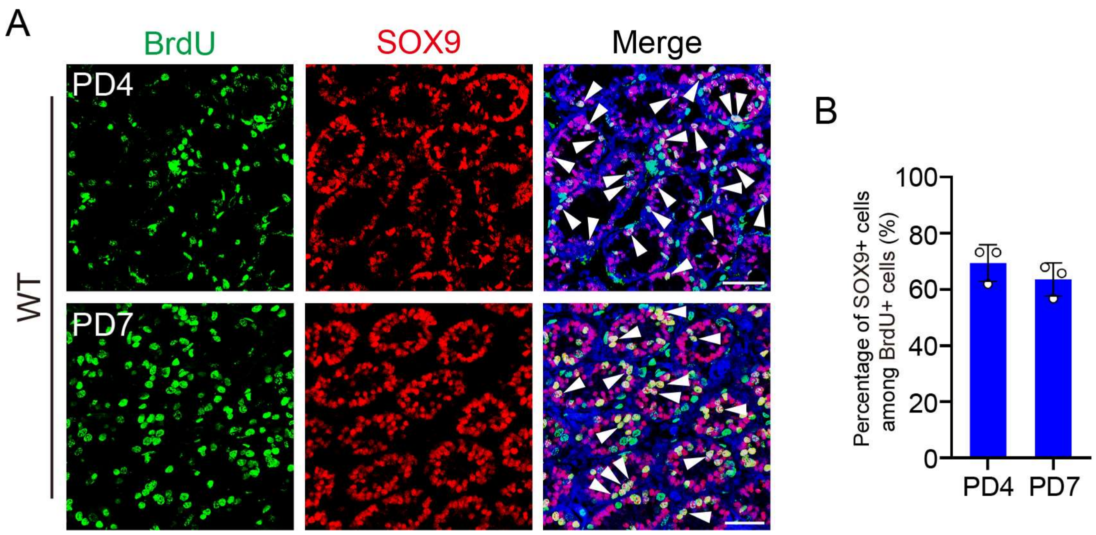

Appendix Figure S4. DNA replication rate analysis of Sertoli cells in WT male mice at PD4 and PD7.

A. IF staining of BrdU and SOX9 in frozen sections of testes from WT mice at PD4 and PD7. The double-positive cells were marked by white arrowhead. Scale bar, 50 µm.

B. Statistical analysis of the percentage of SOX9+ cells among BrdU+ cells in testes from WT mice at PD4 and PD7. Data are presented as mean ± SD. 3 mice were analyzed.

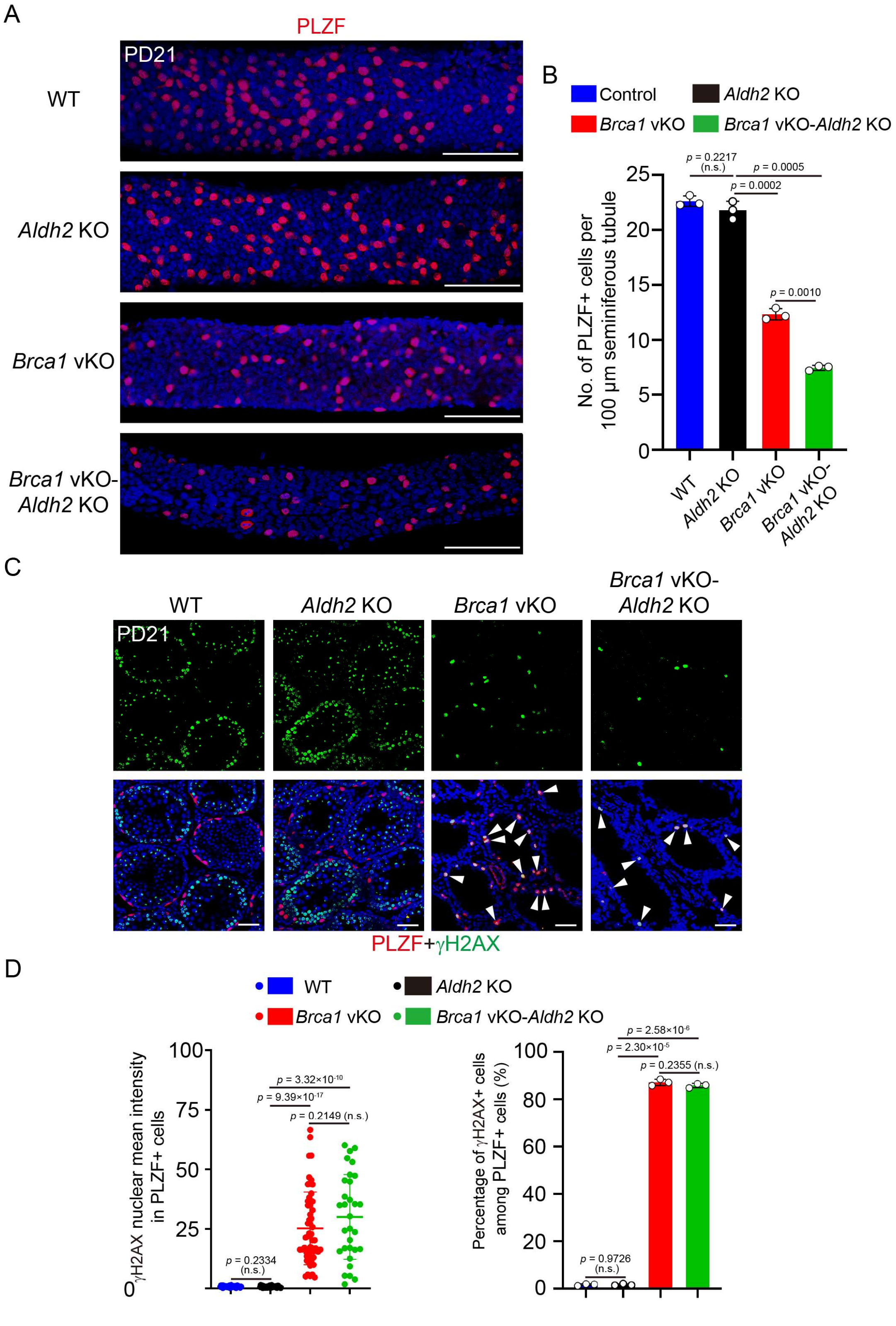

Appendix Figure S5. *Aldh2* KO exacerbates the defects in the formation of undifferentiated spermatogonia in *Brca1* vKO testes.

A. Whole-mount IF staining of PLZF in seminiferous tubules of testes from WT, *Aldh2* KO, *Brca1* vKO, and *Brca1* vKO-*Aldh2* KO male mice at PD21. Scale bar, 100 μm.

B. Statistical analysis of the number of PLZF+ cells per 100 μm seminiferous tubule in testes from WT, *Aldh2* KO, *Brca1* vKO, and *Brca1* vKO-*Aldh2* KO male mice at PD21.

C. IF staining of γH2AX and PLZF in frozen sections of testes from WT, *Aldh2* KO, *Brca1* vKO, and *Brca1* vKO-*Aldh2* KO male mice at PD21. The double-positive cells were marked by white arrowhead. Scale bar, 50 μm.

D. Statistical analysis of γH2AX nuclear mean intensity of PLZF+ cells and the percentage of γH2AX+ cells among PLZF+ cells in frozen sections of testes from WT, *Aldh2* KO, *Brca1* vKO, and *Brca1* vKO-*Aldh2* KO male mice at PD21.

Data are presented as mean ± SD. 3 mice of each genotype were analyzed. p value, two-tailed unpaired student's t-test. n.s., not significant.
