# Supplementary figures and images for: BRCA1 preserves genome integrity during the formation of undifferentiated spermatogonia (part 1 of 3)
Source: EMBO Rep. 2025 May 28;26(15):3747–72. doi: 10.1038/s44319-025-00487-5 (PMC12332178; doi:10.1038/s44319-025-00487-5)

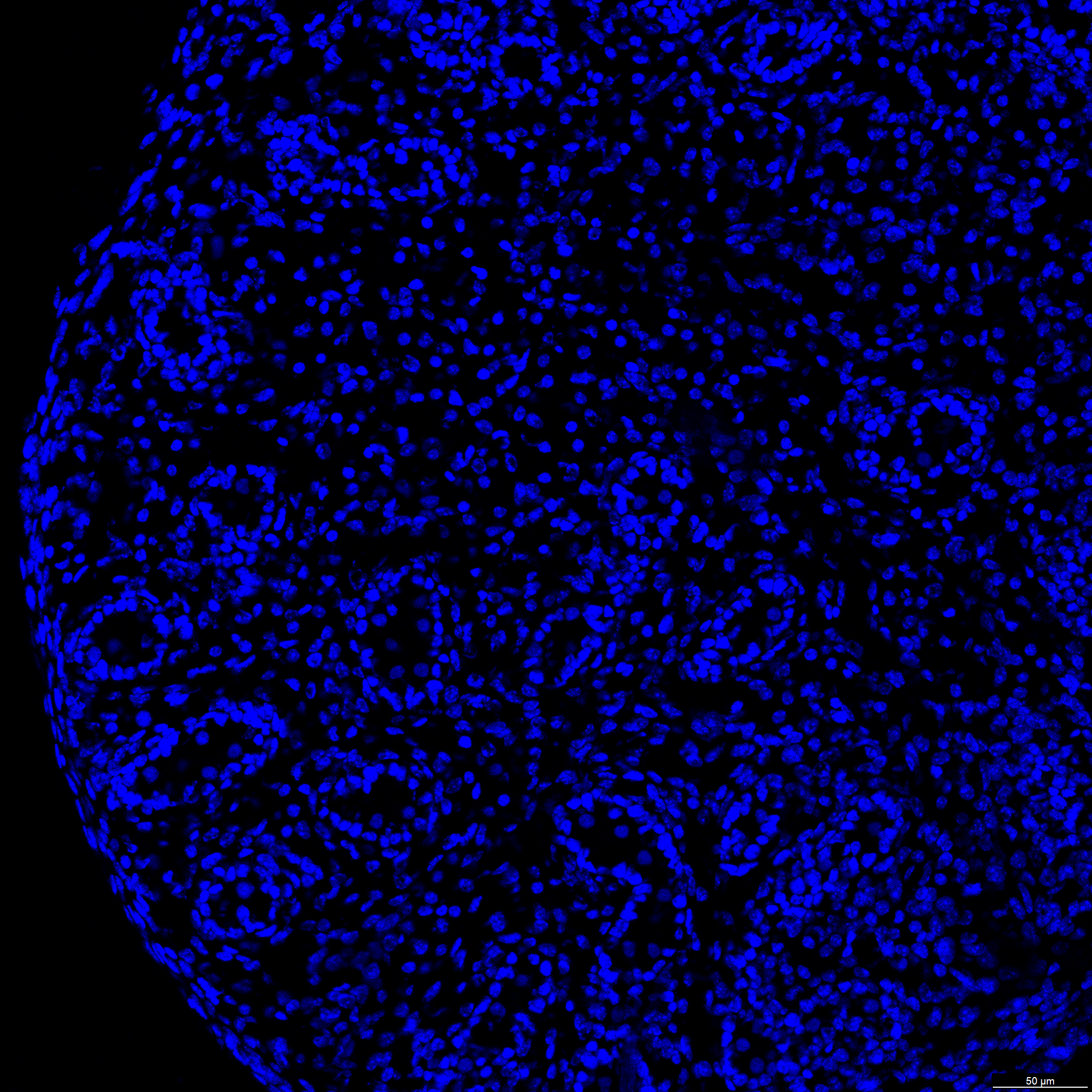

Supplement: Supplementary file 4 — Source data Fig. 1 [file 44319_2025_487_MOESM4_ESM.zip › Figure 1/1A/E18.5 PLZF&γH2AX/E18.5 WT testis PLZF&γH2AX Hoechst.tif]

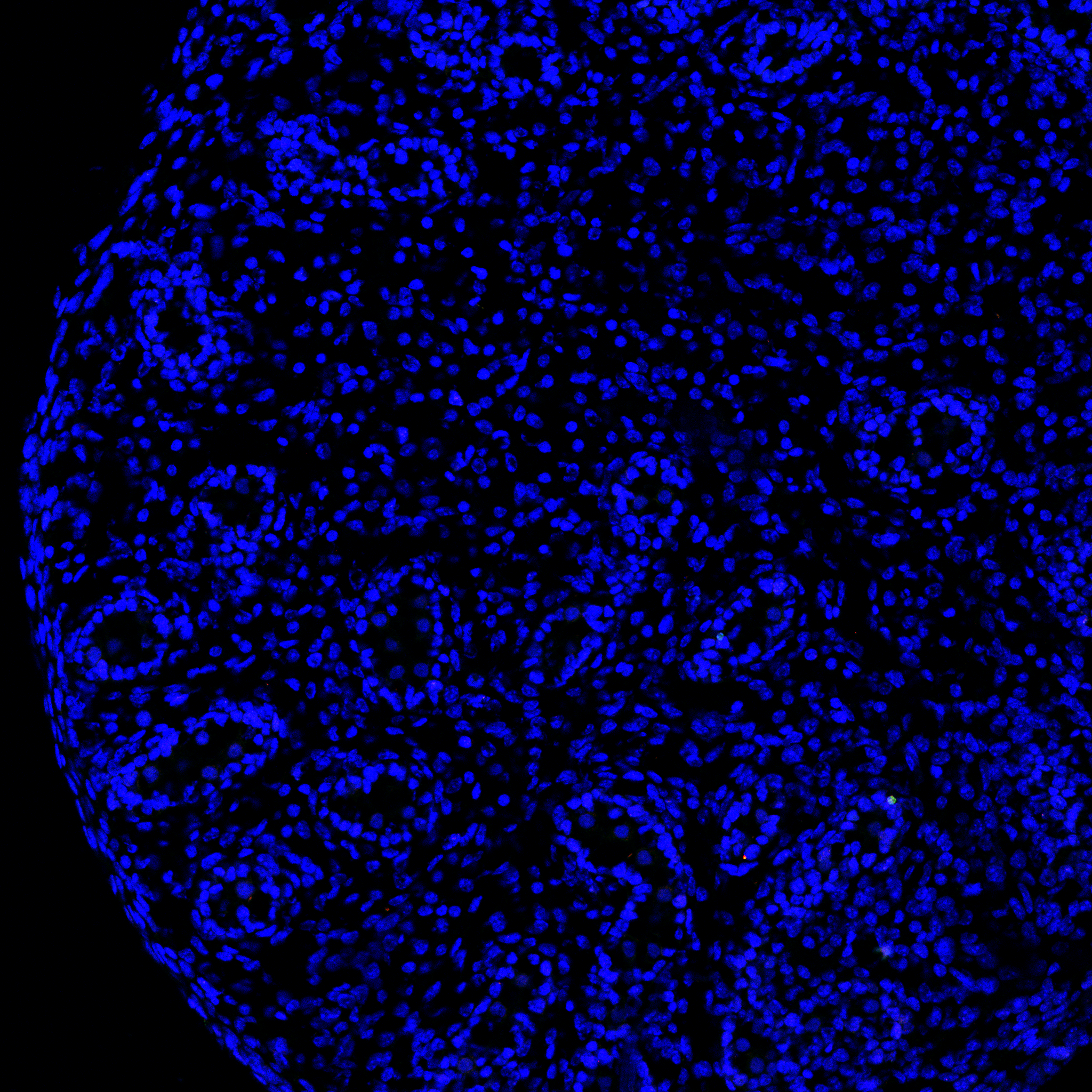

Supplement: Supplementary file 4 — Source data Fig. 1 [file 44319_2025_487_MOESM4_ESM.zip › Figure 1/1A/E18.5 PLZF&γH2AX/E18.5 WT testis PLZF&γH2AX overlay.tif]

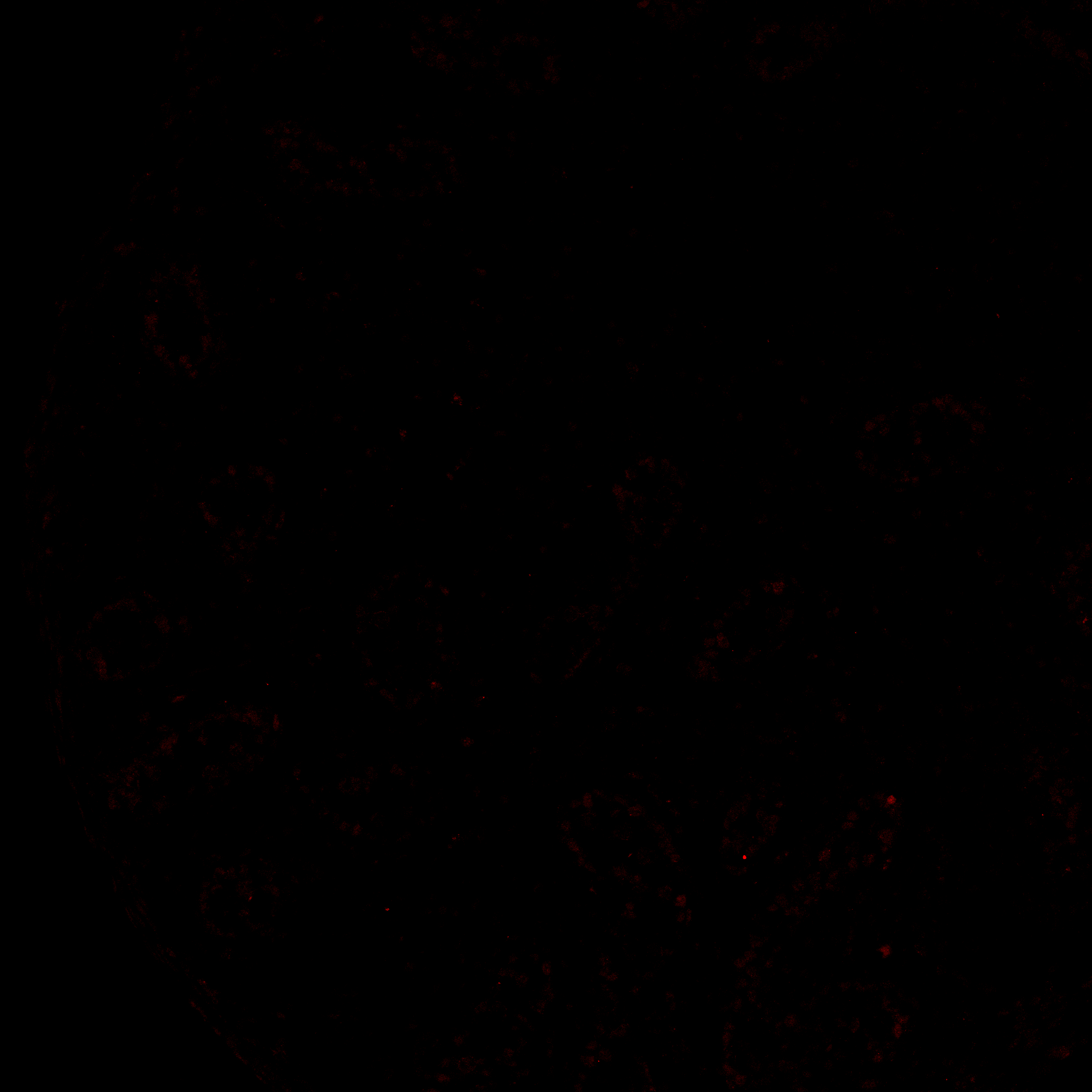

Supplement: Supplementary file 4 — Source data Fig. 1 [file 44319_2025_487_MOESM4_ESM.zip › Figure 1/1A/E18.5 PLZF&γH2AX/E18.5 WT testis PLZF.tif]

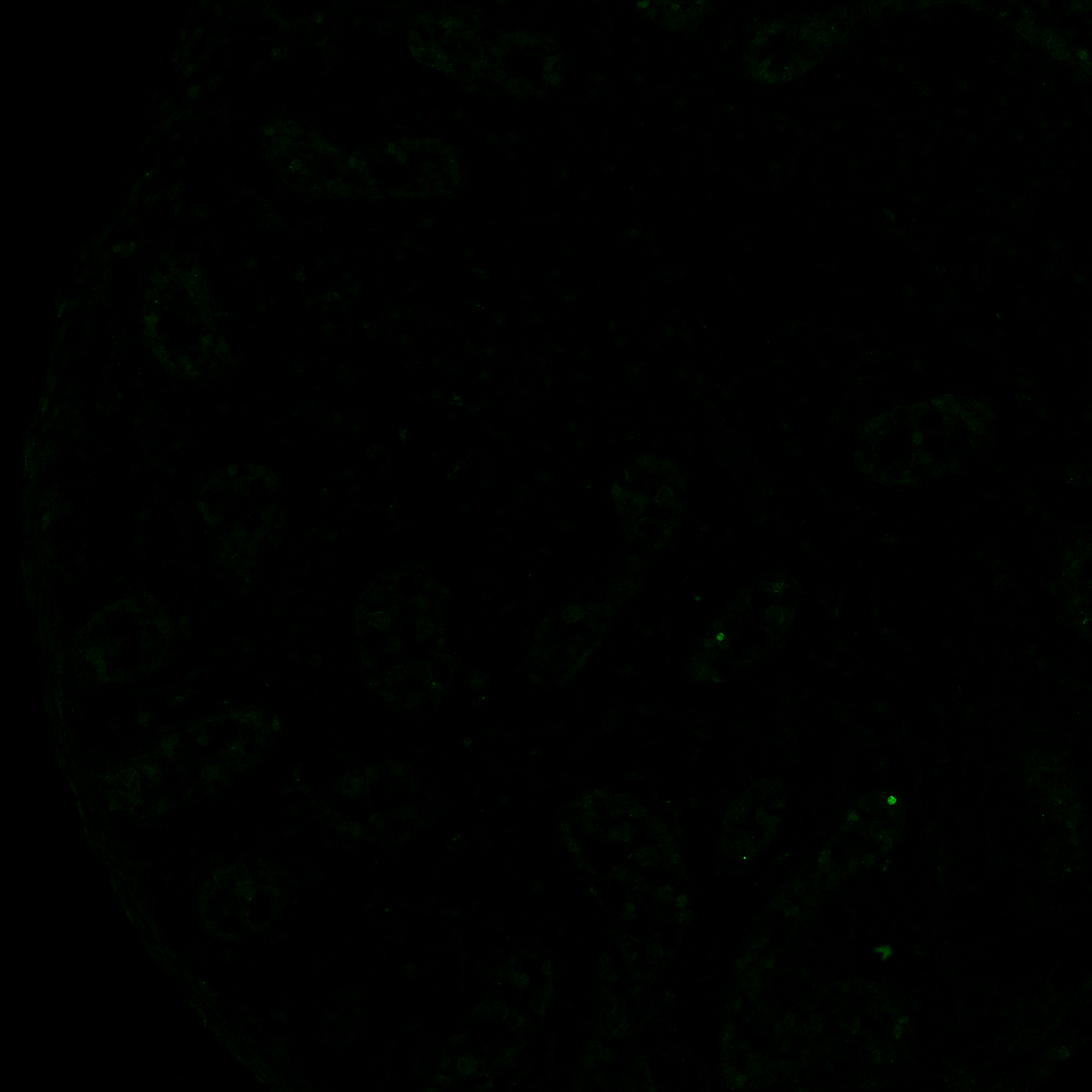

Supplement: Supplementary file 4 — Source data Fig. 1 [file 44319_2025_487_MOESM4_ESM.zip › Figure 1/1A/E18.5 PLZF&γH2AX/E18.5 WT testis γH2AX.tif]

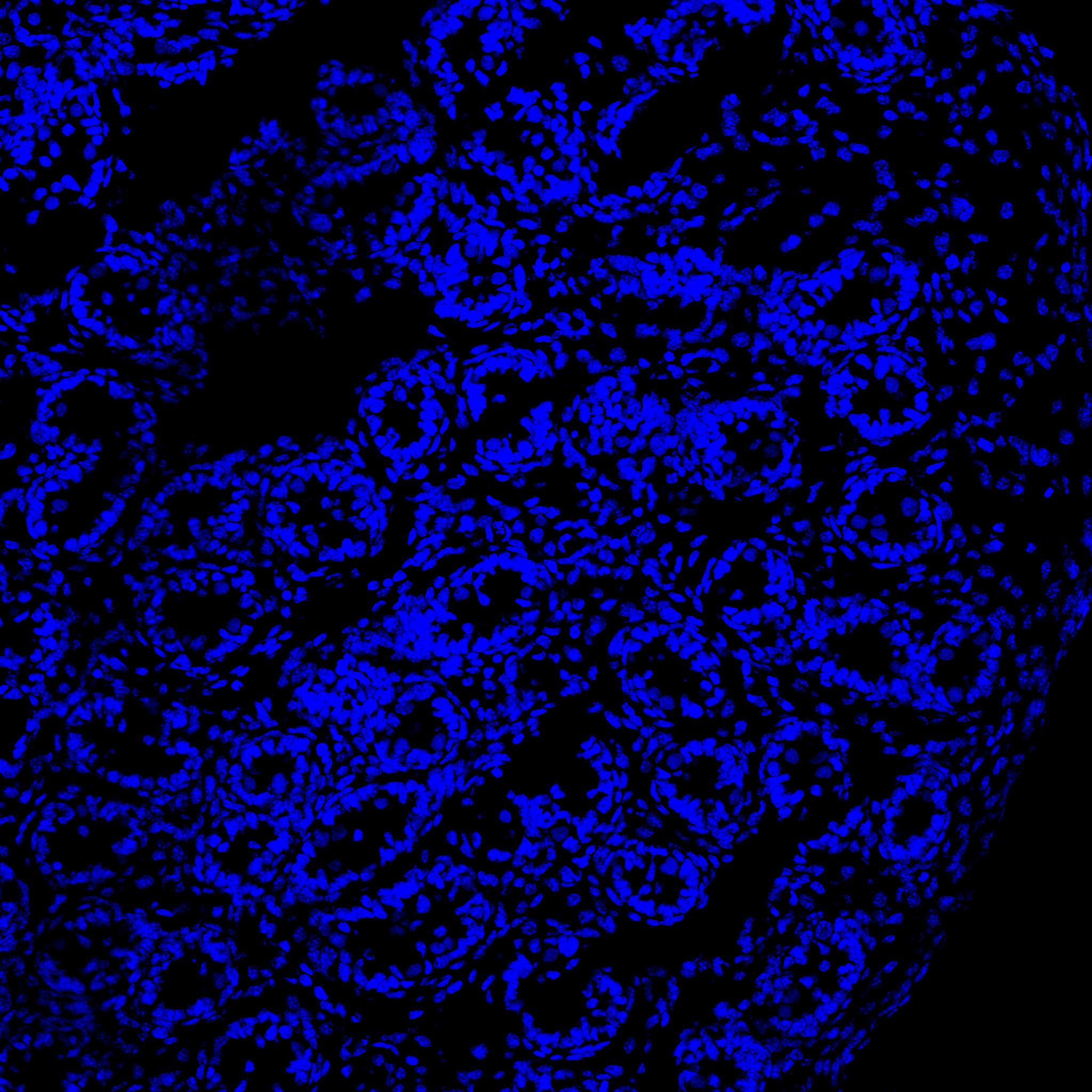

Supplement: Supplementary file 4 — Source data Fig. 1 [file 44319_2025_487_MOESM4_ESM.zip › Figure 1/1A/PD1 PLZF&γH2AX/PD1 WT testis PLZF&γH2AX Hoechst.tif]

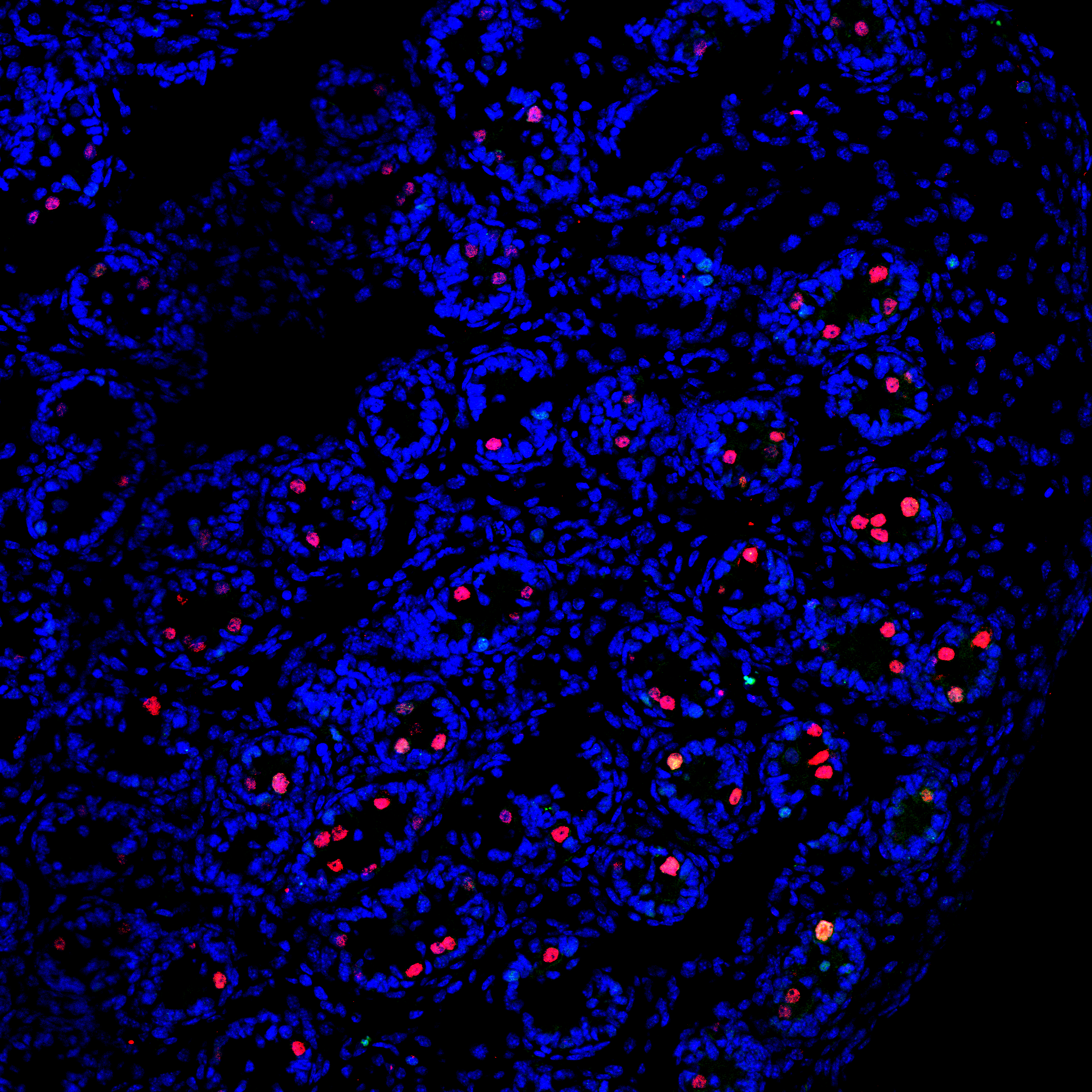

Supplement: Supplementary file 4 — Source data Fig. 1 [file 44319_2025_487_MOESM4_ESM.zip › Figure 1/1A/PD1 PLZF&γH2AX/PD1 WT testis PLZF&γH2AX Hoechst_overlay.tif]

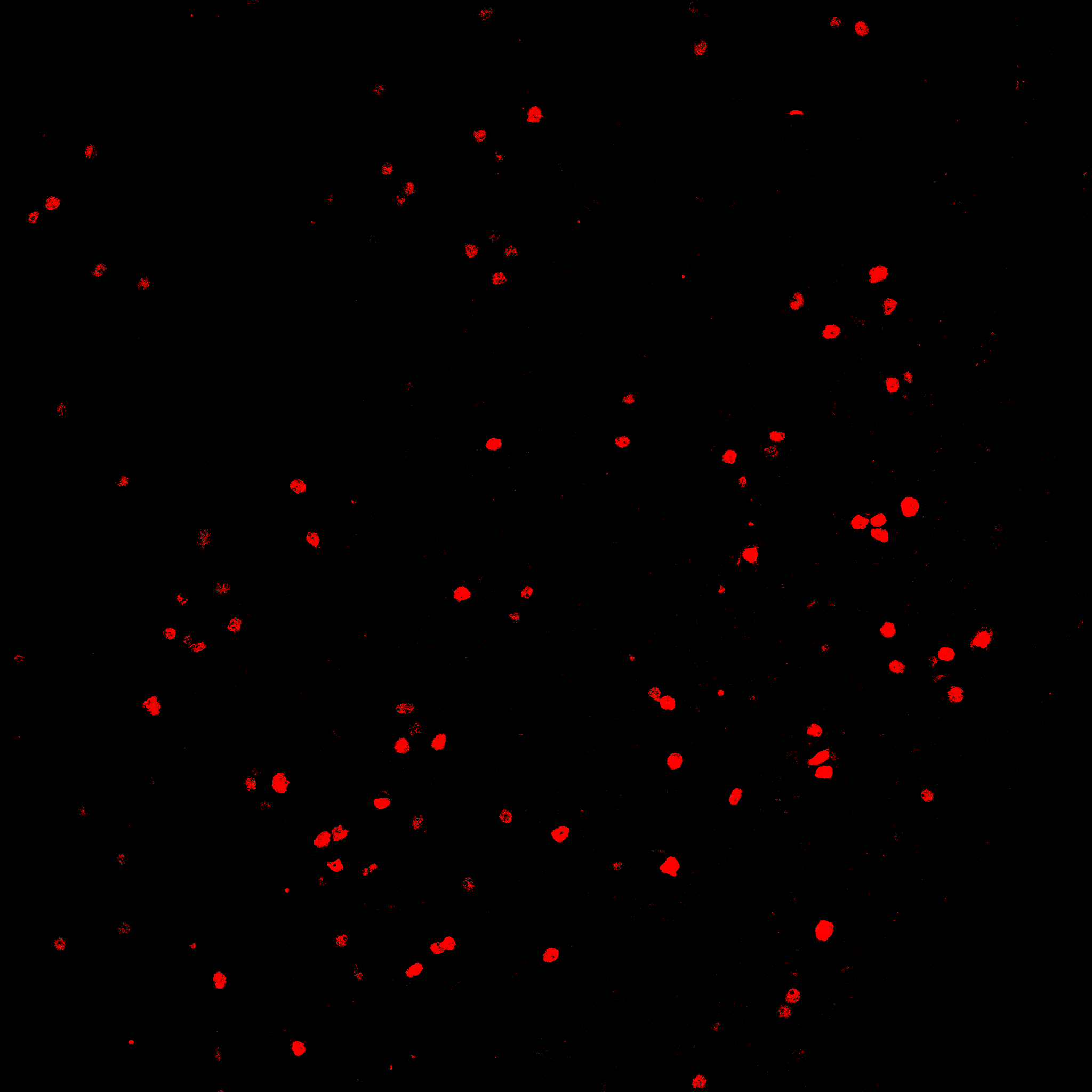

Supplement: Supplementary file 4 — Source data Fig. 1 [file 44319_2025_487_MOESM4_ESM.zip › Figure 1/1A/PD1 PLZF&γH2AX/PD1 WT testis PLZF.tif]

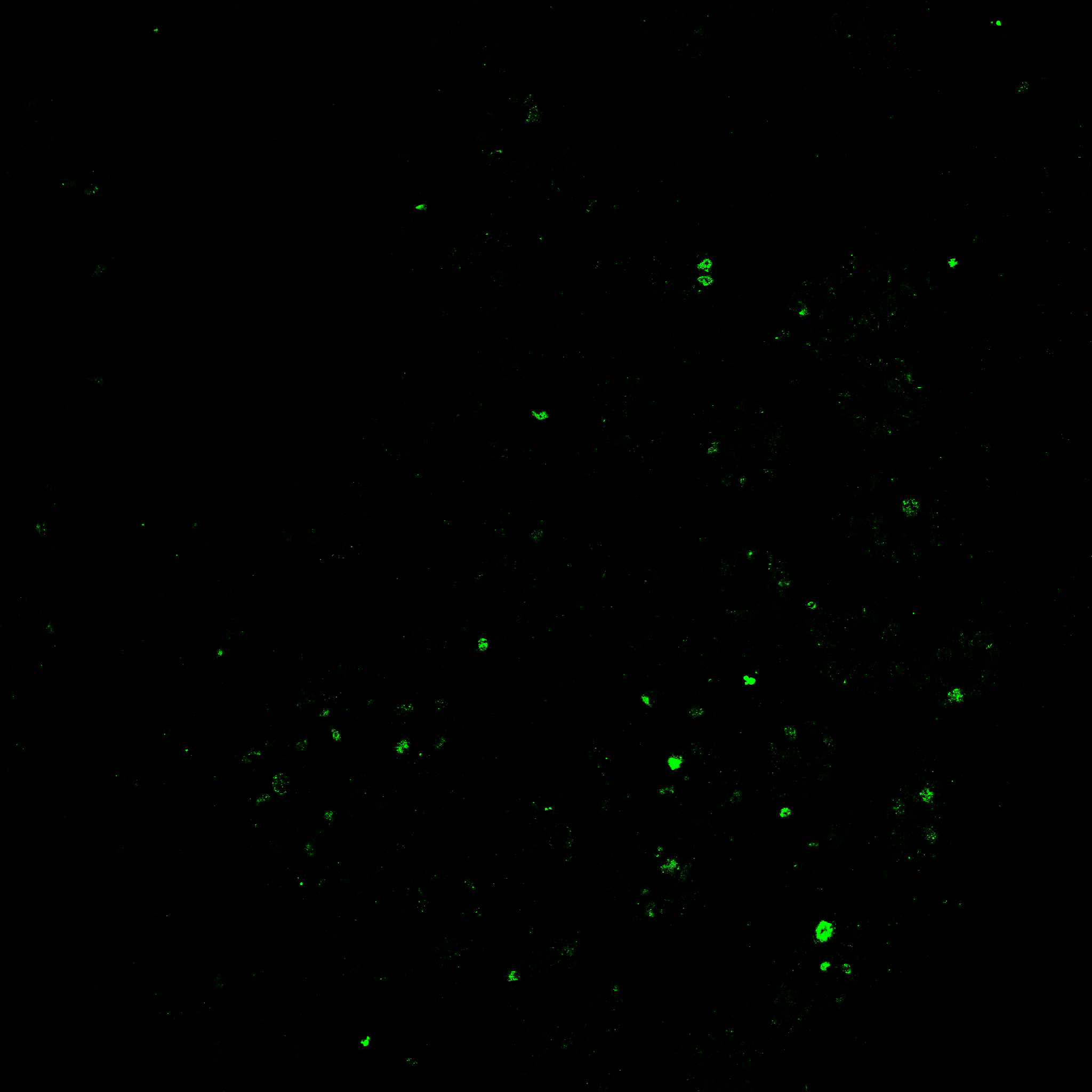

Supplement: Supplementary file 4 — Source data Fig. 1 [file 44319_2025_487_MOESM4_ESM.zip › Figure 1/1A/PD1 PLZF&γH2AX/PD1 WT testis γH2AX.tif]

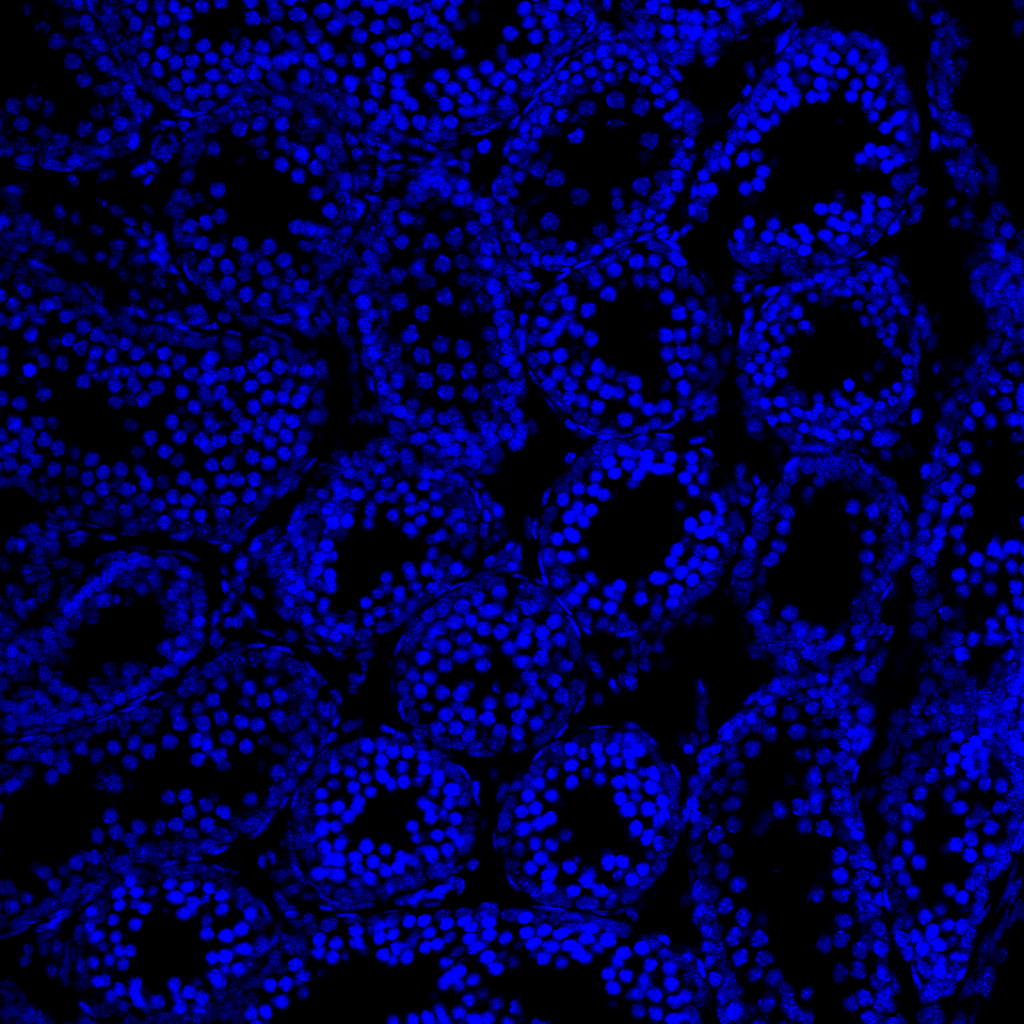

Supplement: Supplementary file 4 — Source data Fig. 1 [file 44319_2025_487_MOESM4_ESM.zip › Figure 1/1A/PD14 PLZF&γH2AX/PD14 WT testis PLZF&γH2AX Hoechst.tif]

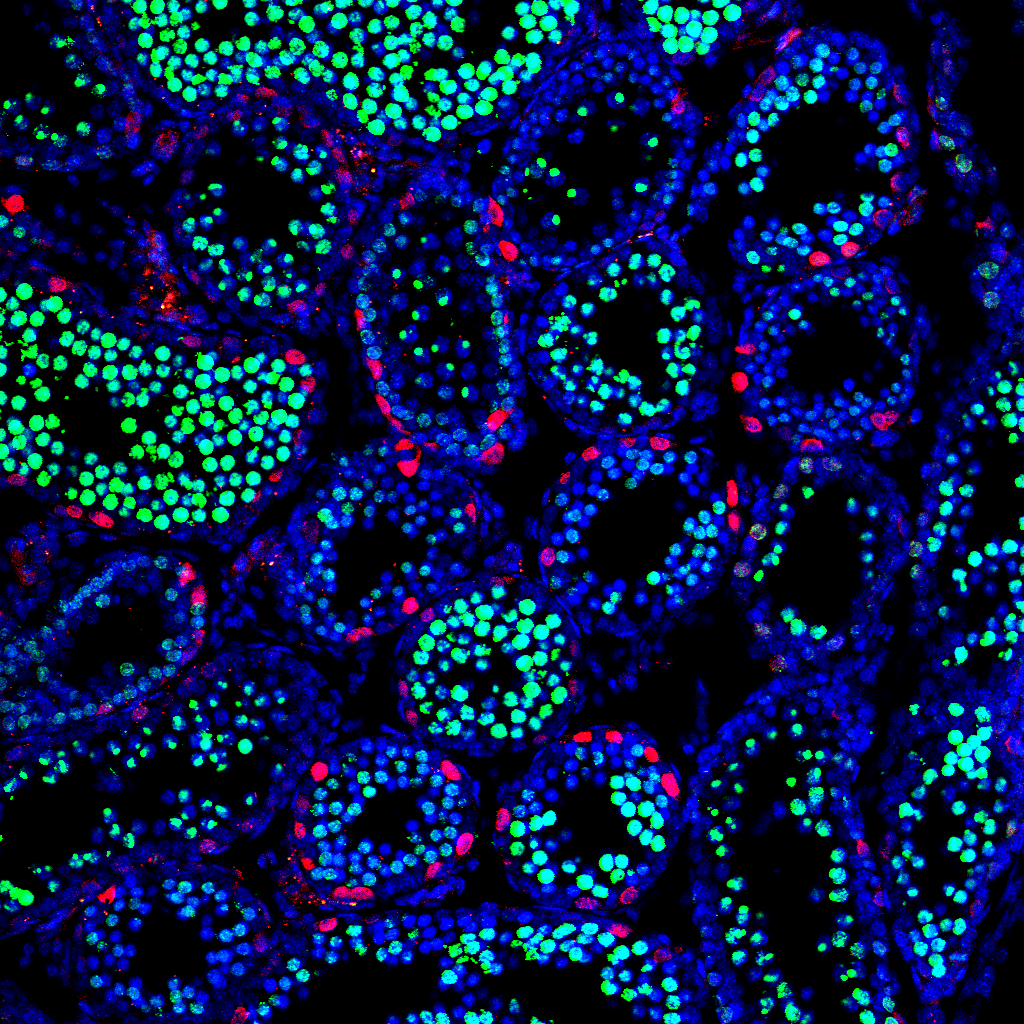

Supplement: Supplementary file 4 — Source data Fig. 1 [file 44319_2025_487_MOESM4_ESM.zip › Figure 1/1A/PD14 PLZF&γH2AX/PD14 WT testis PLZF&γH2AX Hoechst_overlay.tif]

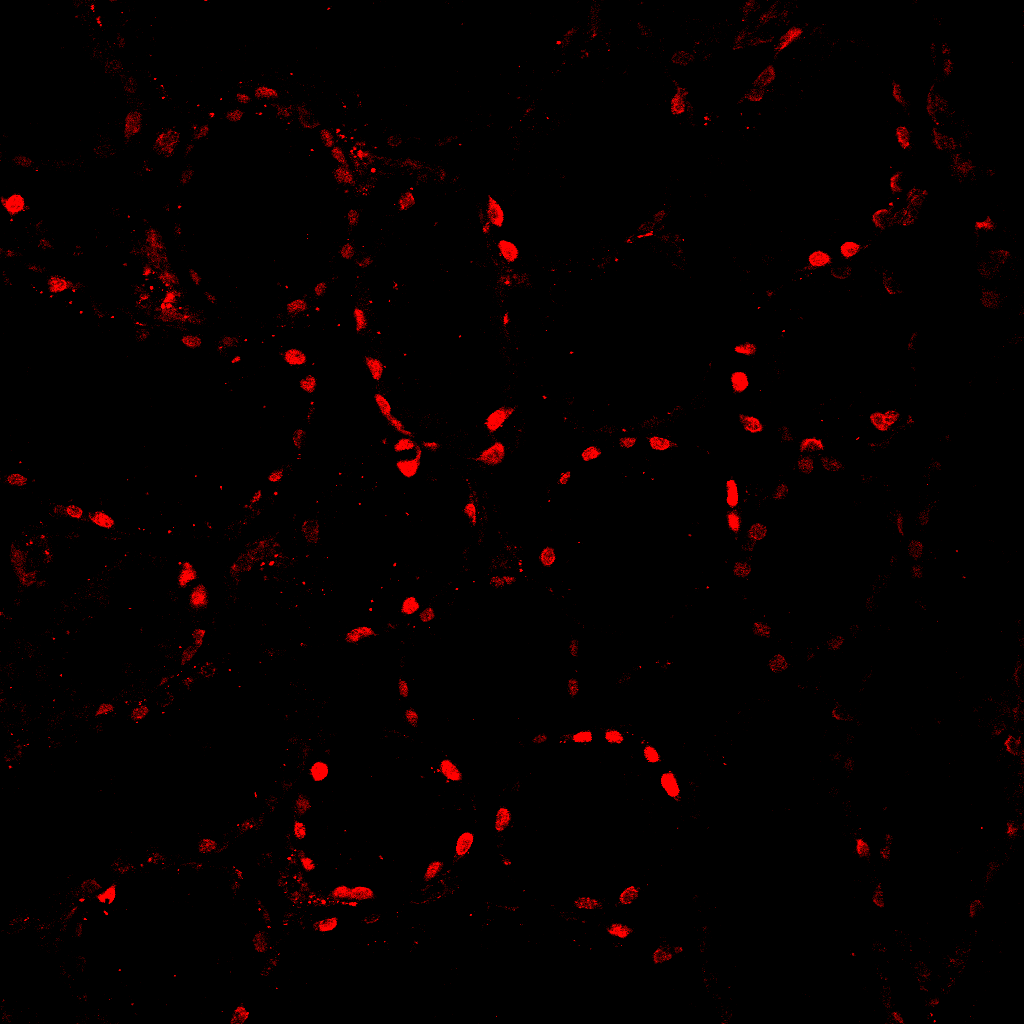

Supplement: Supplementary file 4 — Source data Fig. 1 [file 44319_2025_487_MOESM4_ESM.zip › Figure 1/1A/PD14 PLZF&γH2AX/PD14 WT testis PLZF.tif]

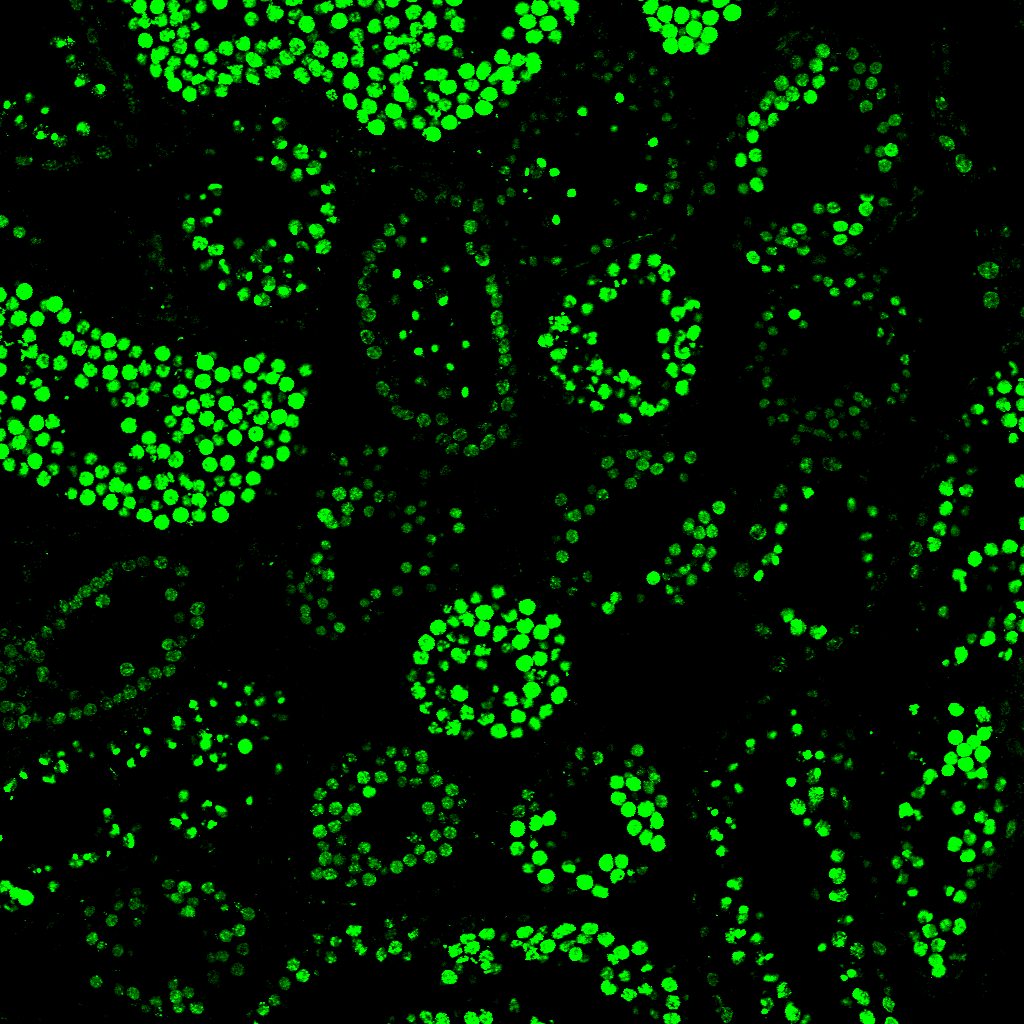

Supplement: Supplementary file 4 — Source data Fig. 1 [file 44319_2025_487_MOESM4_ESM.zip › Figure 1/1A/PD14 PLZF&γH2AX/PD14 WT testis γH2AX.tif]

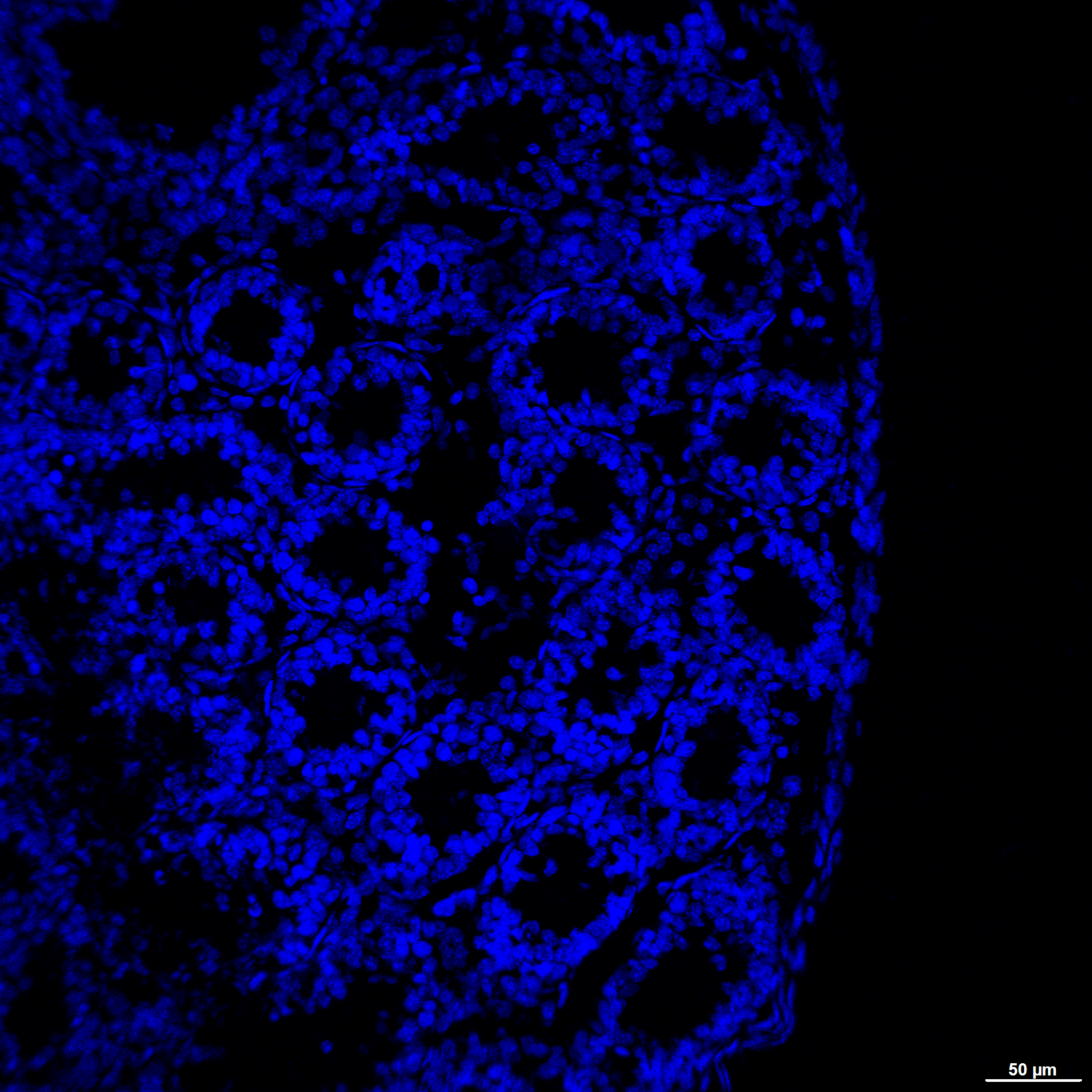

Supplement: Supplementary file 4 — Source data Fig. 1 [file 44319_2025_487_MOESM4_ESM.zip › Figure 1/1A/PD4 PLZF&γH2AX/PD4 WT testis PLZF&γH2AX Hoechst.tif]

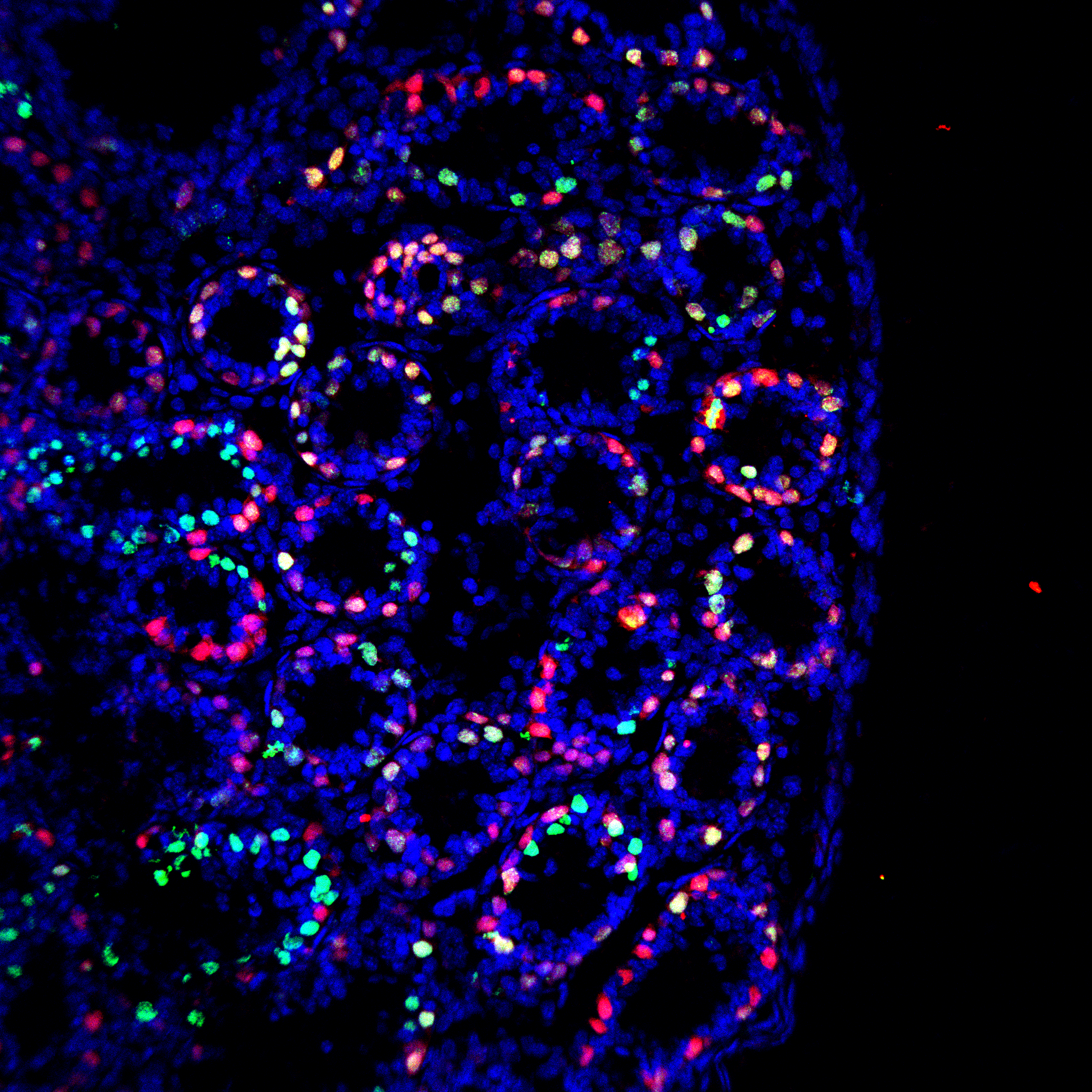

Supplement: Supplementary file 4 — Source data Fig. 1 [file 44319_2025_487_MOESM4_ESM.zip › Figure 1/1A/PD4 PLZF&γH2AX/PD4 WT testis PLZF&γH2AX Hoechst_overlay.tif]

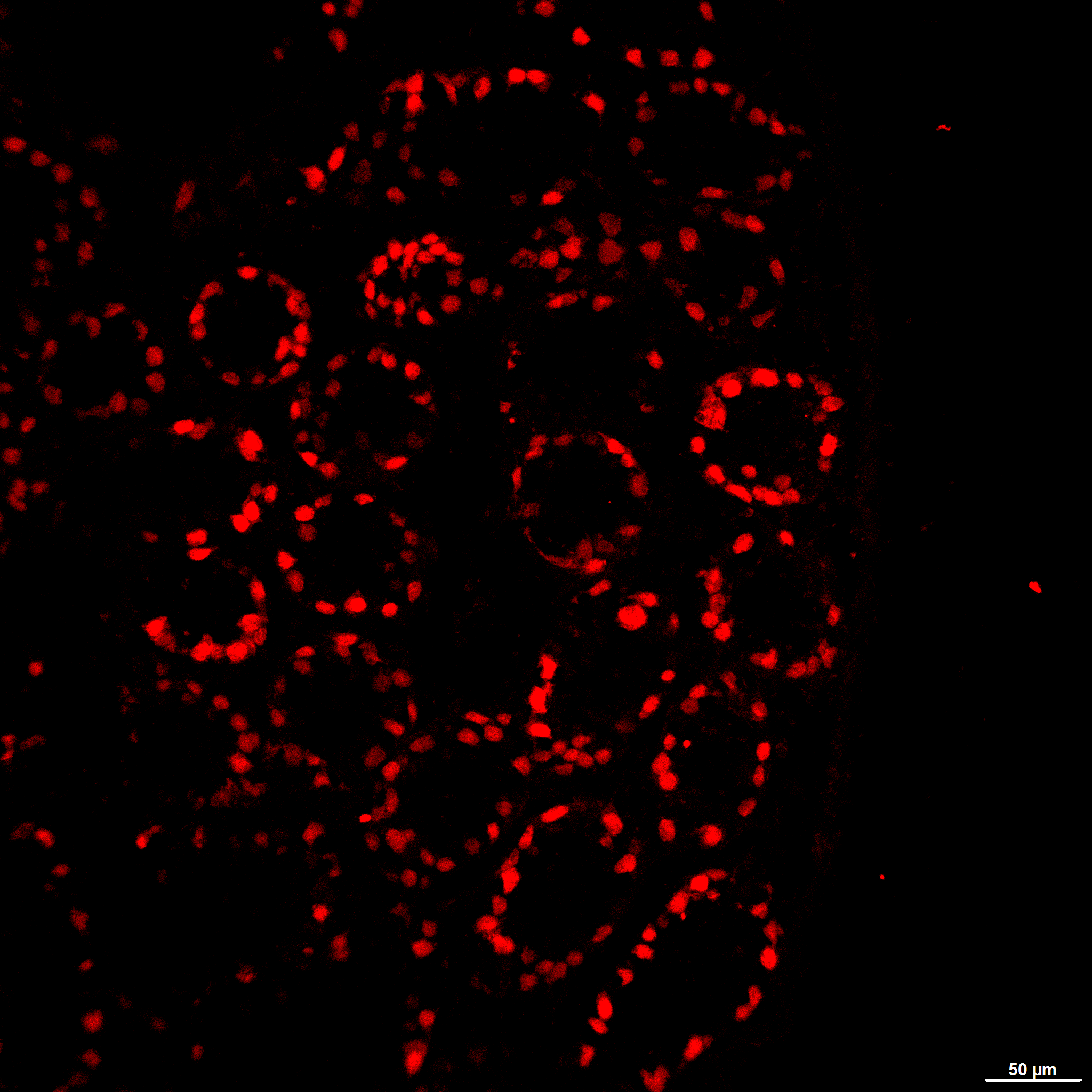

Supplement: Supplementary file 4 — Source data Fig. 1 [file 44319_2025_487_MOESM4_ESM.zip › Figure 1/1A/PD4 PLZF&γH2AX/PD4 WT testis PLZF.tif]

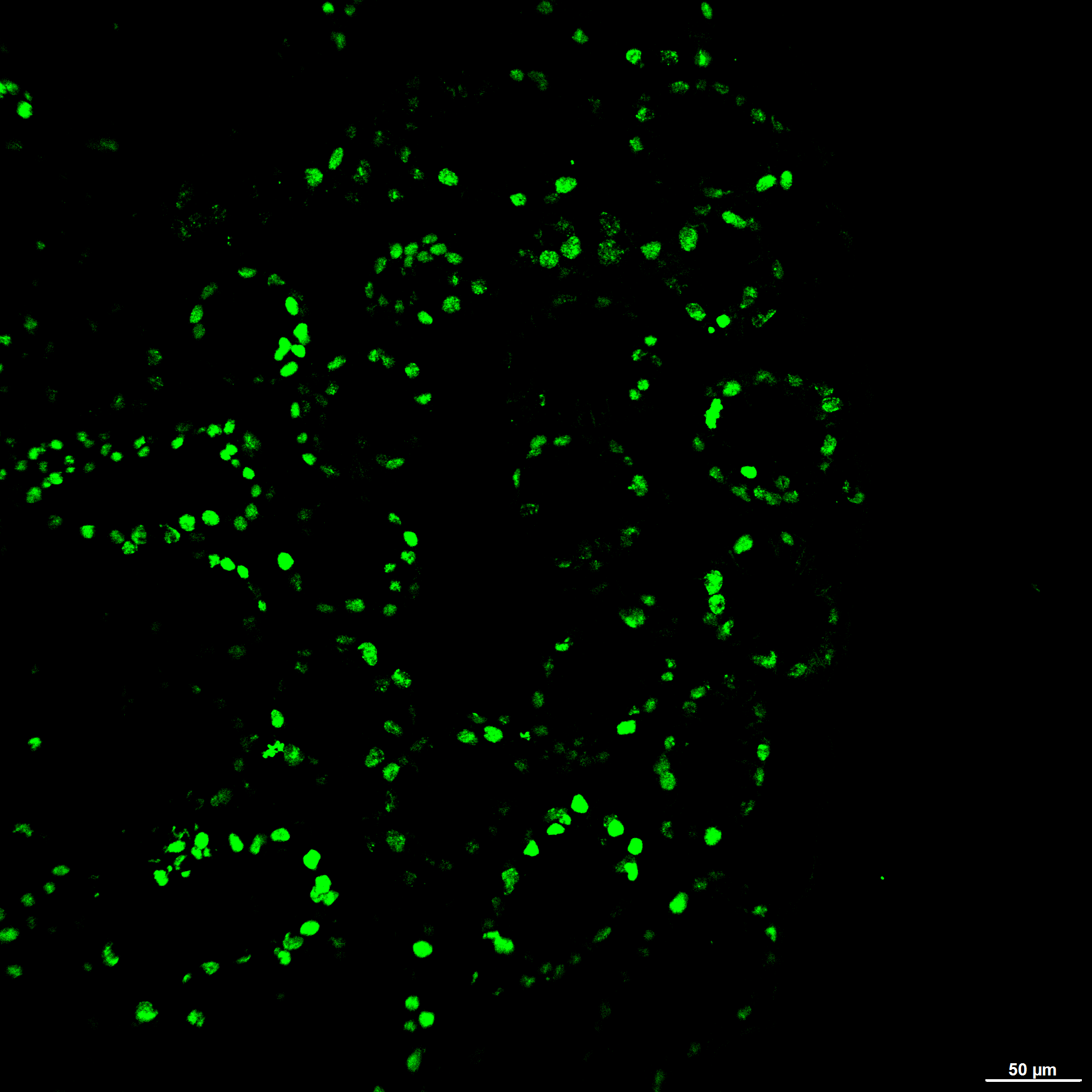

Supplement: Supplementary file 4 — Source data Fig. 1 [file 44319_2025_487_MOESM4_ESM.zip › Figure 1/1A/PD4 PLZF&γH2AX/PD4 WT testis γH2AX.tif]

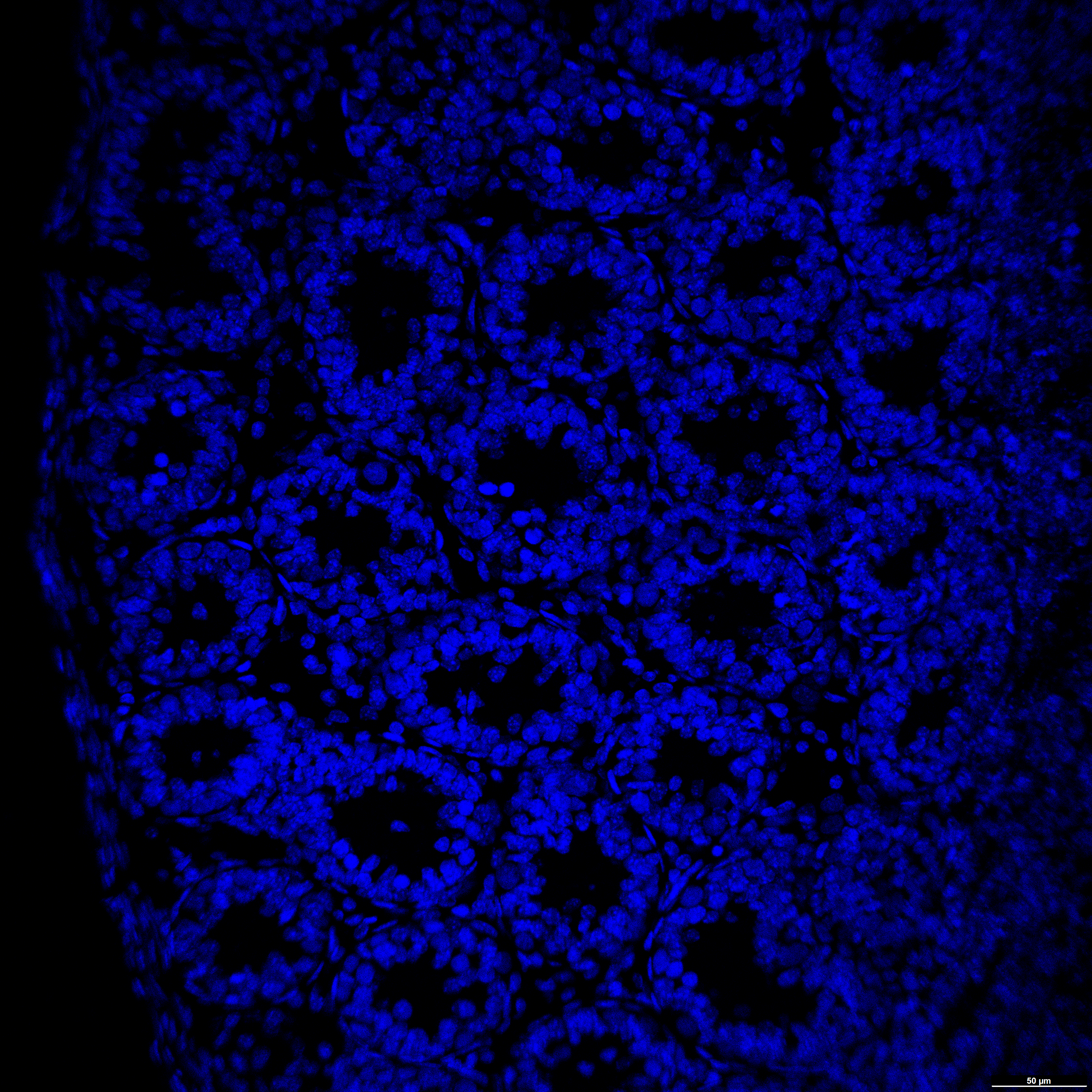

Supplement: Supplementary file 4 — Source data Fig. 1 [file 44319_2025_487_MOESM4_ESM.zip › Figure 1/1A/PD7 PLZF&γH2AX/PD7 WT testis PLZF&γH2AX Hoechst.tif]

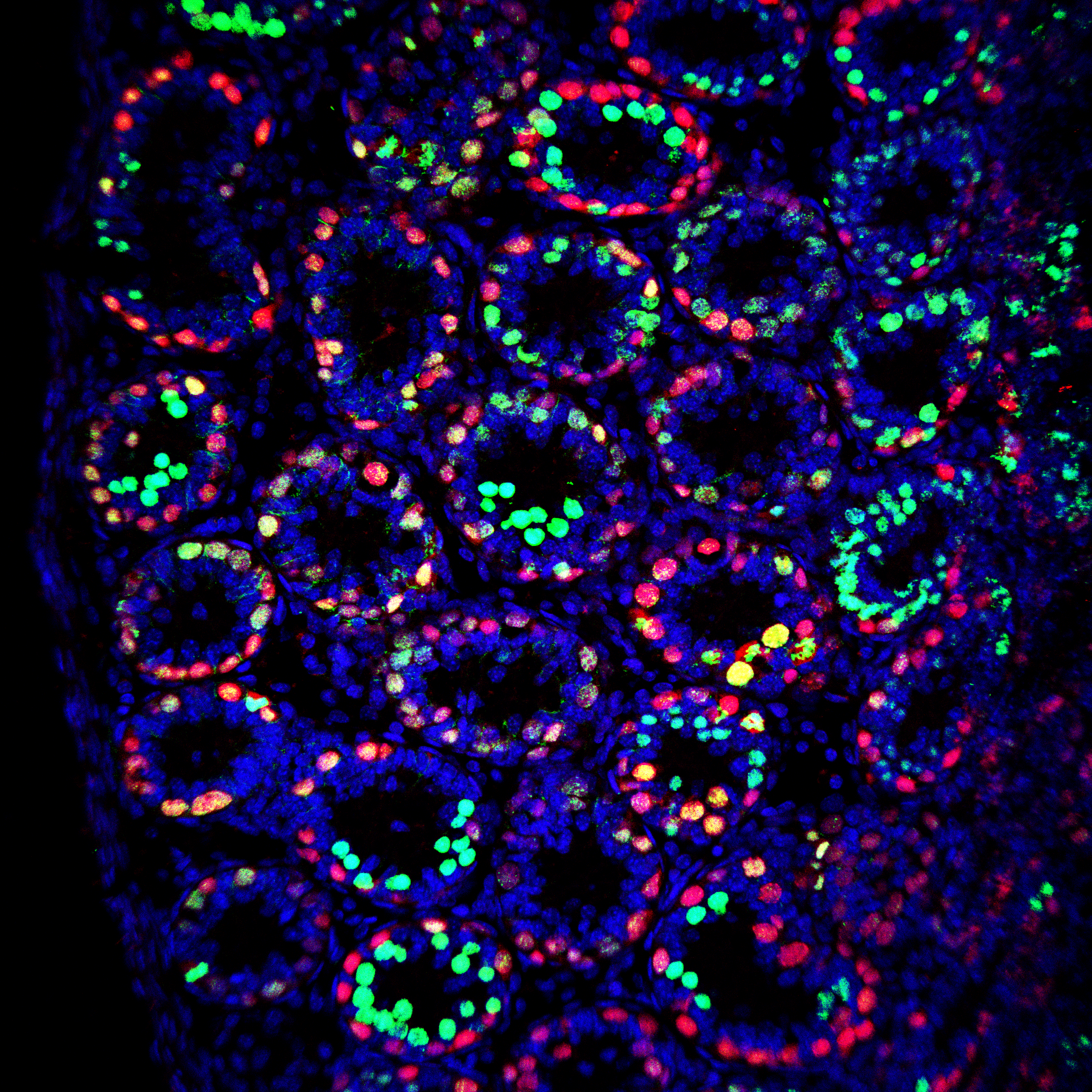

Supplement: Supplementary file 4 — Source data Fig. 1 [file 44319_2025_487_MOESM4_ESM.zip › Figure 1/1A/PD7 PLZF&γH2AX/PD7 WT testis PLZF&γH2AX Hoechst_overlay.tif]

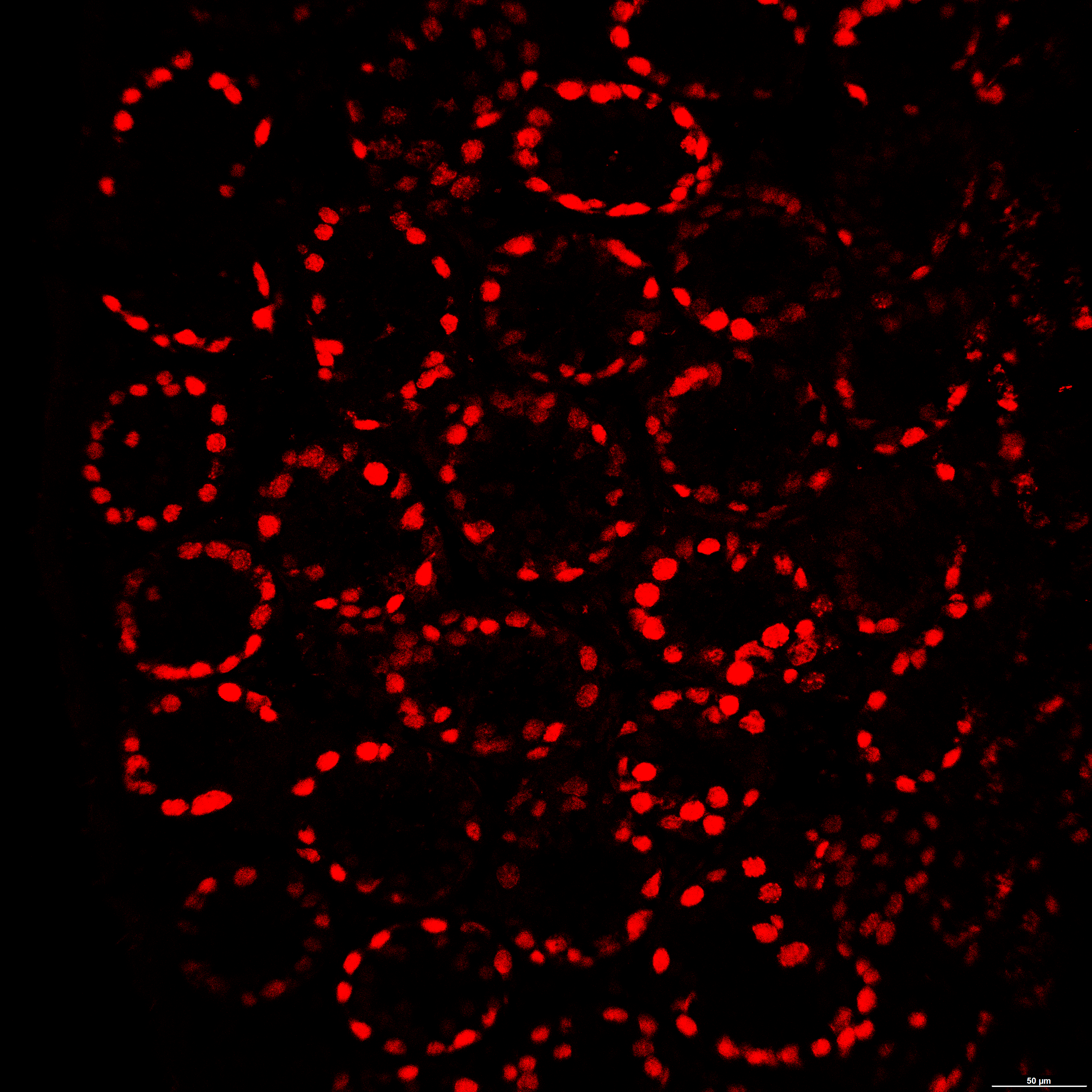

Supplement: Supplementary file 4 — Source data Fig. 1 [file 44319_2025_487_MOESM4_ESM.zip › Figure 1/1A/PD7 PLZF&γH2AX/PD7 WT testis PLZF.tif]

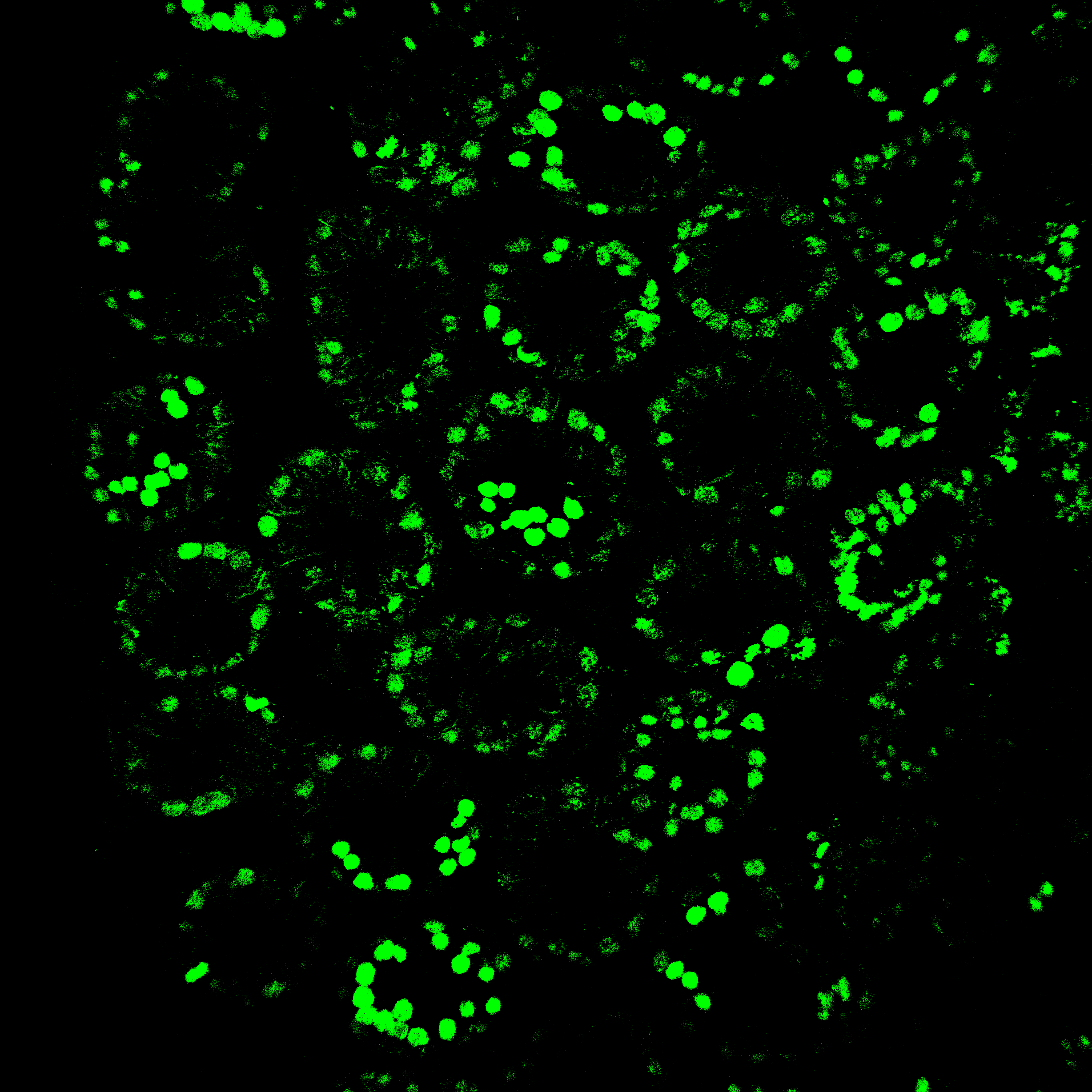

Supplement: Supplementary file 4 — Source data Fig. 1 [file 44319_2025_487_MOESM4_ESM.zip › Figure 1/1A/PD7 PLZF&γH2AX/PD7 WT testis γH2AX.tif]

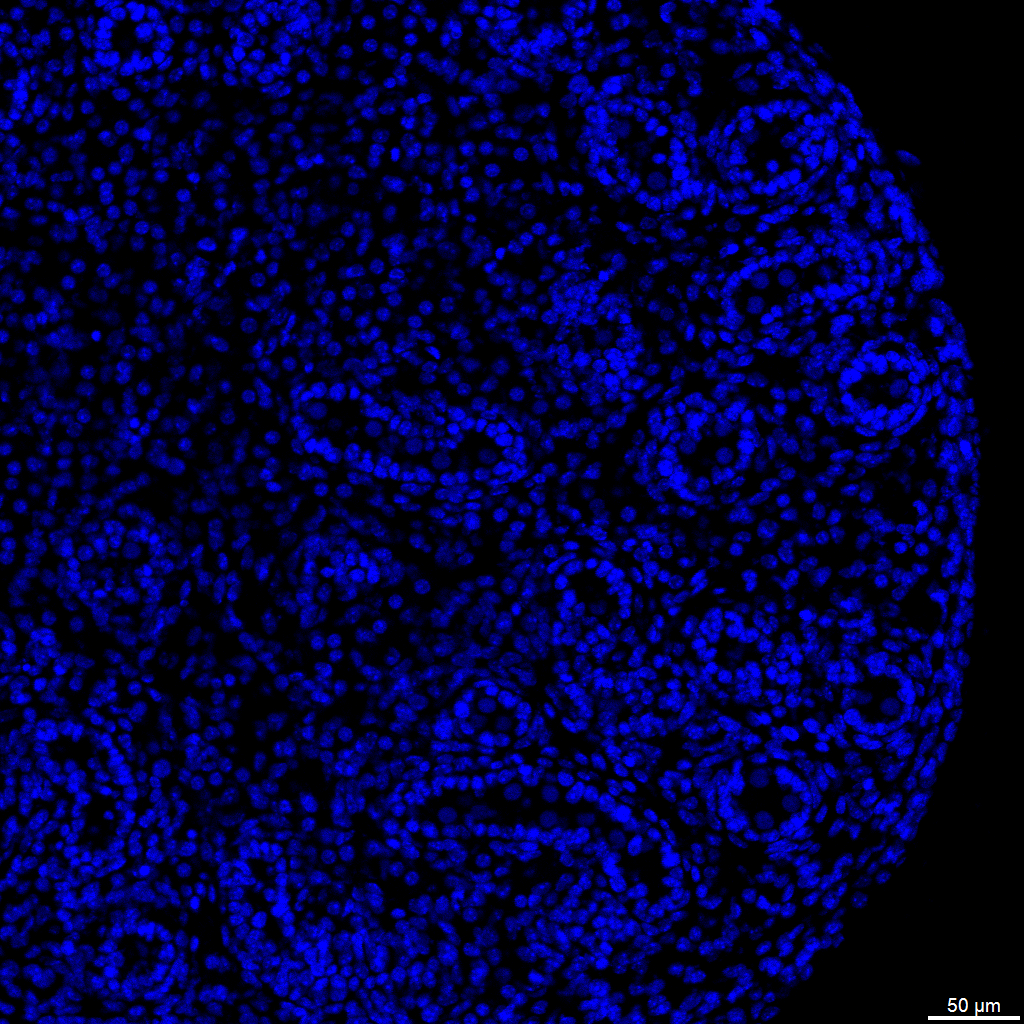

Supplement: Supplementary file 4 — Source data Fig. 1 [file 44319_2025_487_MOESM4_ESM.zip › Figure 1/1B/E18.5 SOX9&γH2AX/E18.5 WT testis SOX9&γH2AX Hoechst.tif]

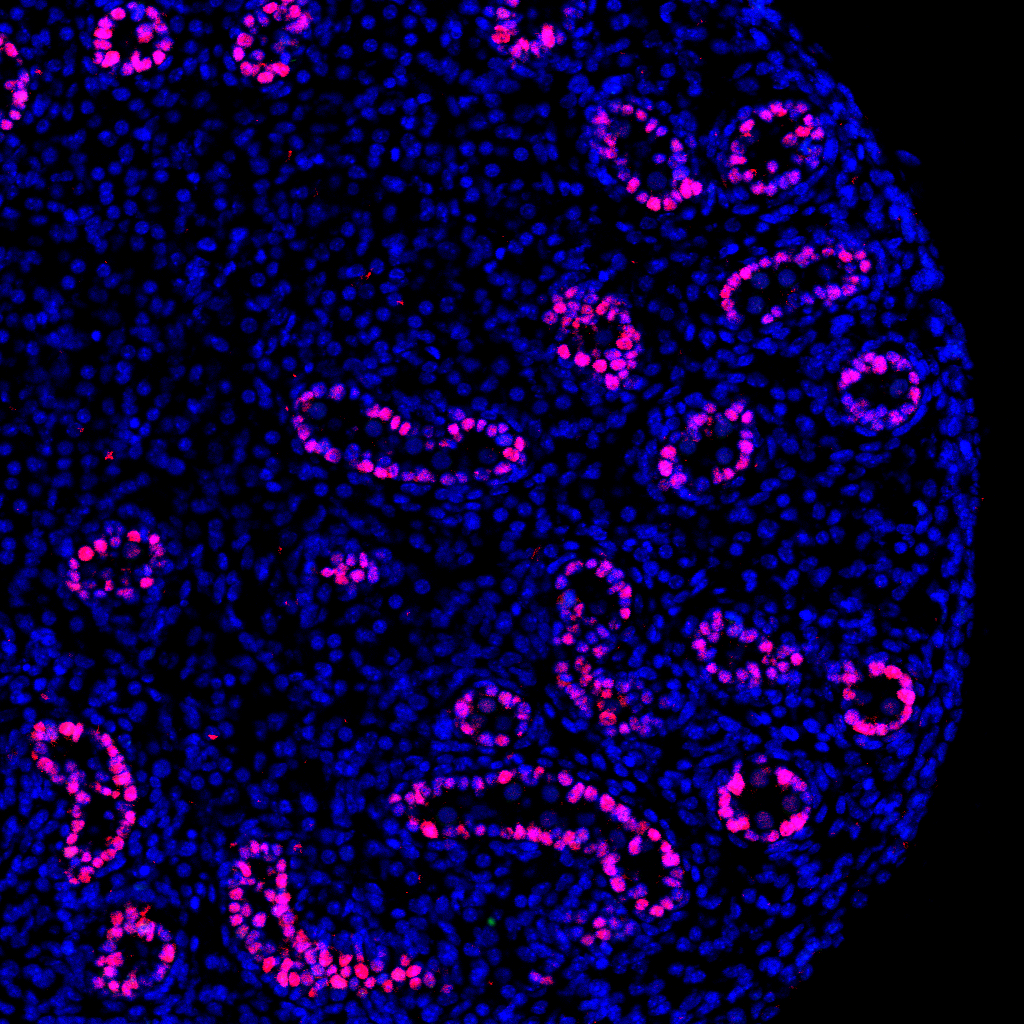

Supplement: Supplementary file 4 — Source data Fig. 1 [file 44319_2025_487_MOESM4_ESM.zip › Figure 1/1B/E18.5 SOX9&γH2AX/E18.5 WT testis SOX9&γH2AX _overlay.tif]

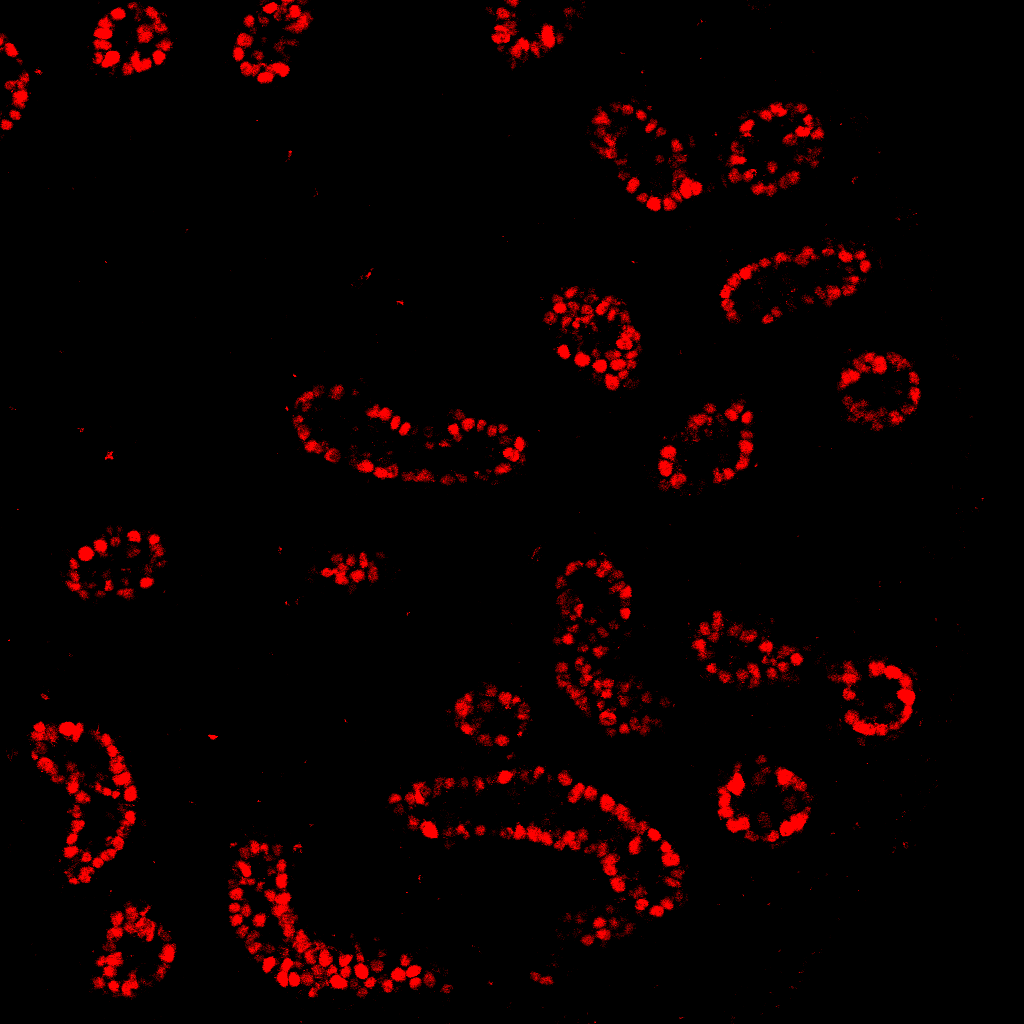

Supplement: Supplementary file 4 — Source data Fig. 1 [file 44319_2025_487_MOESM4_ESM.zip › Figure 1/1B/E18.5 SOX9&γH2AX/E18.5 WT testis SOX9.tif]

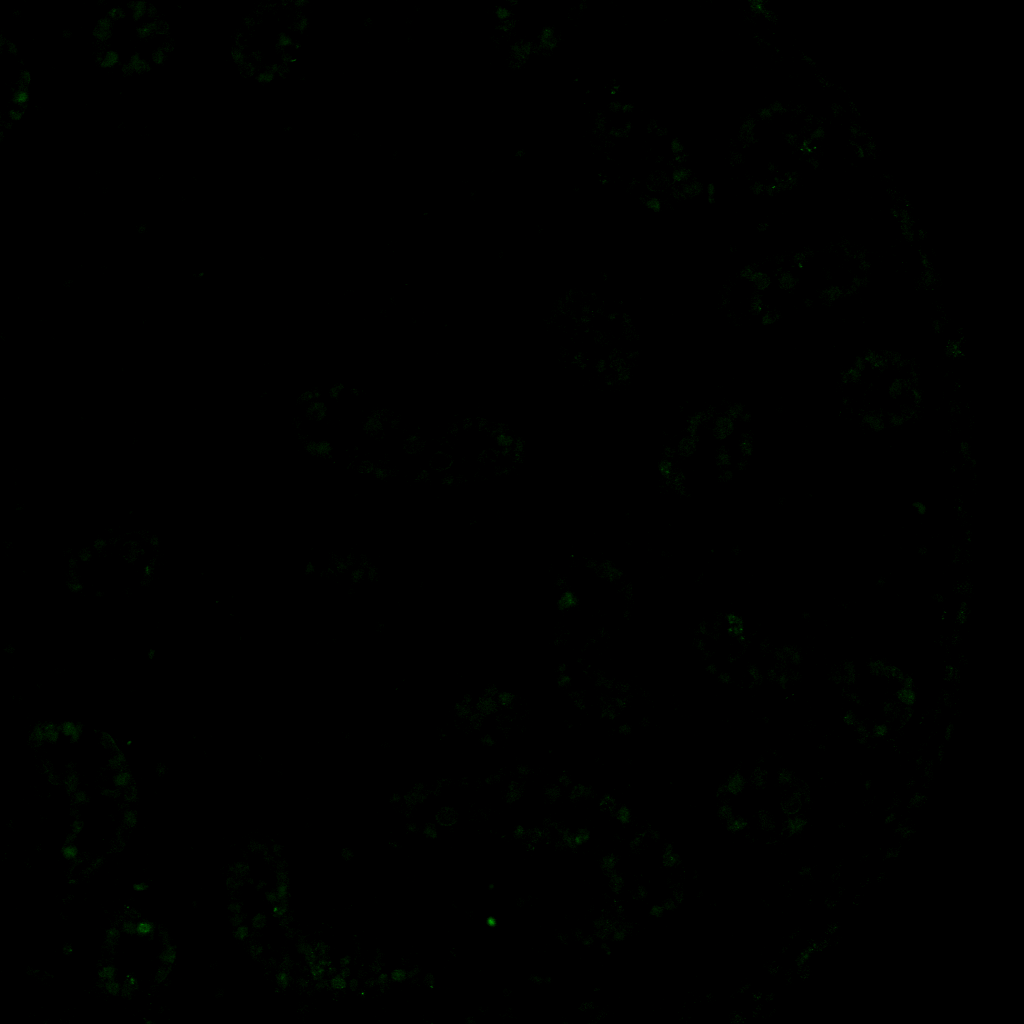

Supplement: Supplementary file 4 — Source data Fig. 1 [file 44319_2025_487_MOESM4_ESM.zip › Figure 1/1B/E18.5 SOX9&γH2AX/E18.5 WT testis γH2AX .tif]

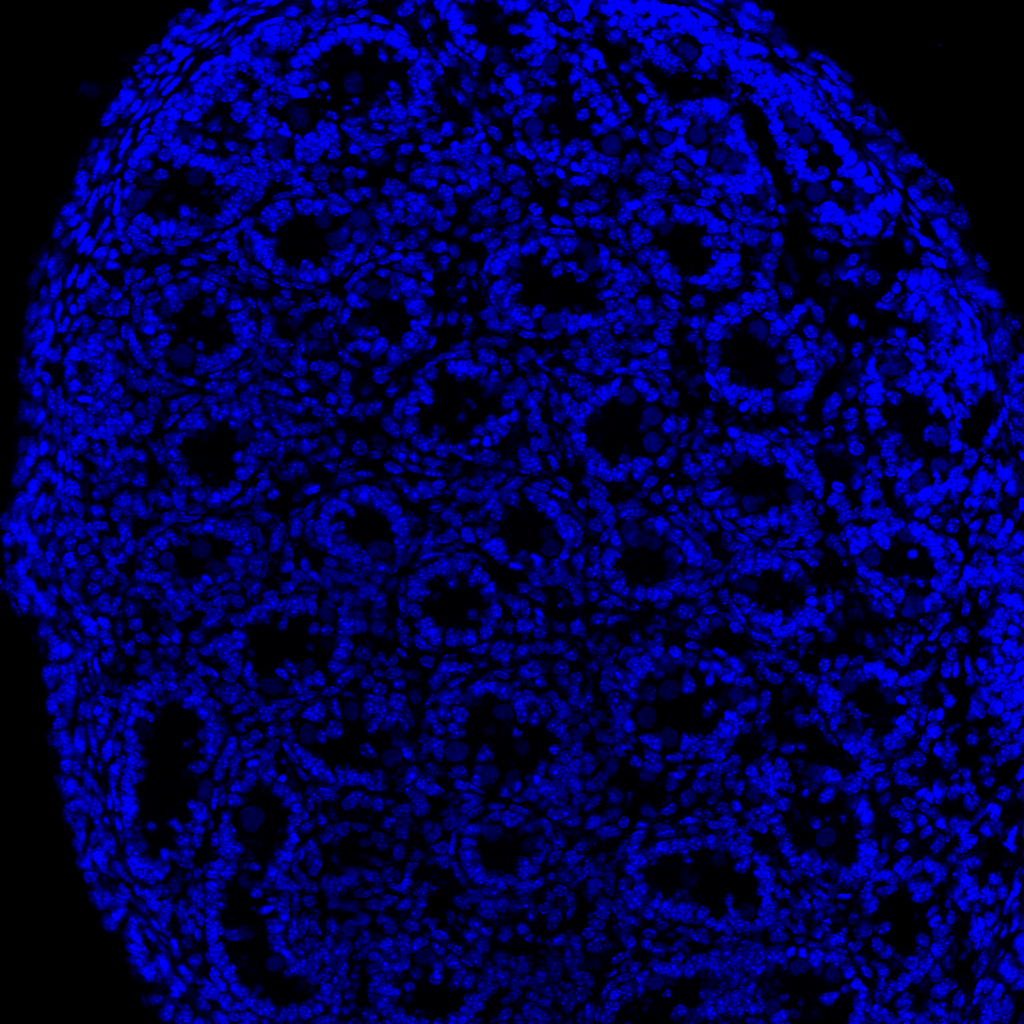

Supplement: Supplementary file 4 — Source data Fig. 1 [file 44319_2025_487_MOESM4_ESM.zip › Figure 1/1B/PD1 SOX9&γH2AX/PD1 WT testis SOX9&γH2AX Hoechst.tif]

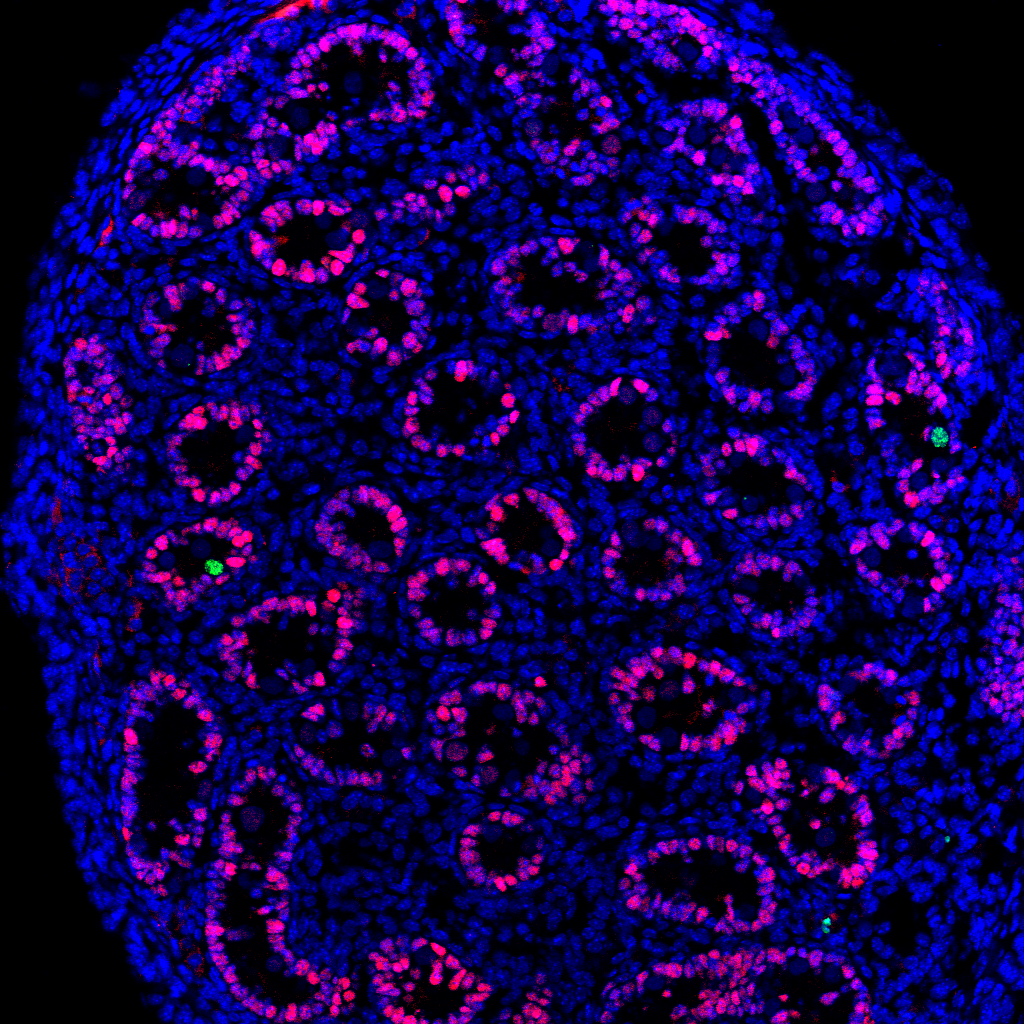

Supplement: Supplementary file 4 — Source data Fig. 1 [file 44319_2025_487_MOESM4_ESM.zip › Figure 1/1B/PD1 SOX9&γH2AX/PD1 WT testis SOX9&γH2AX_overlay.tif]

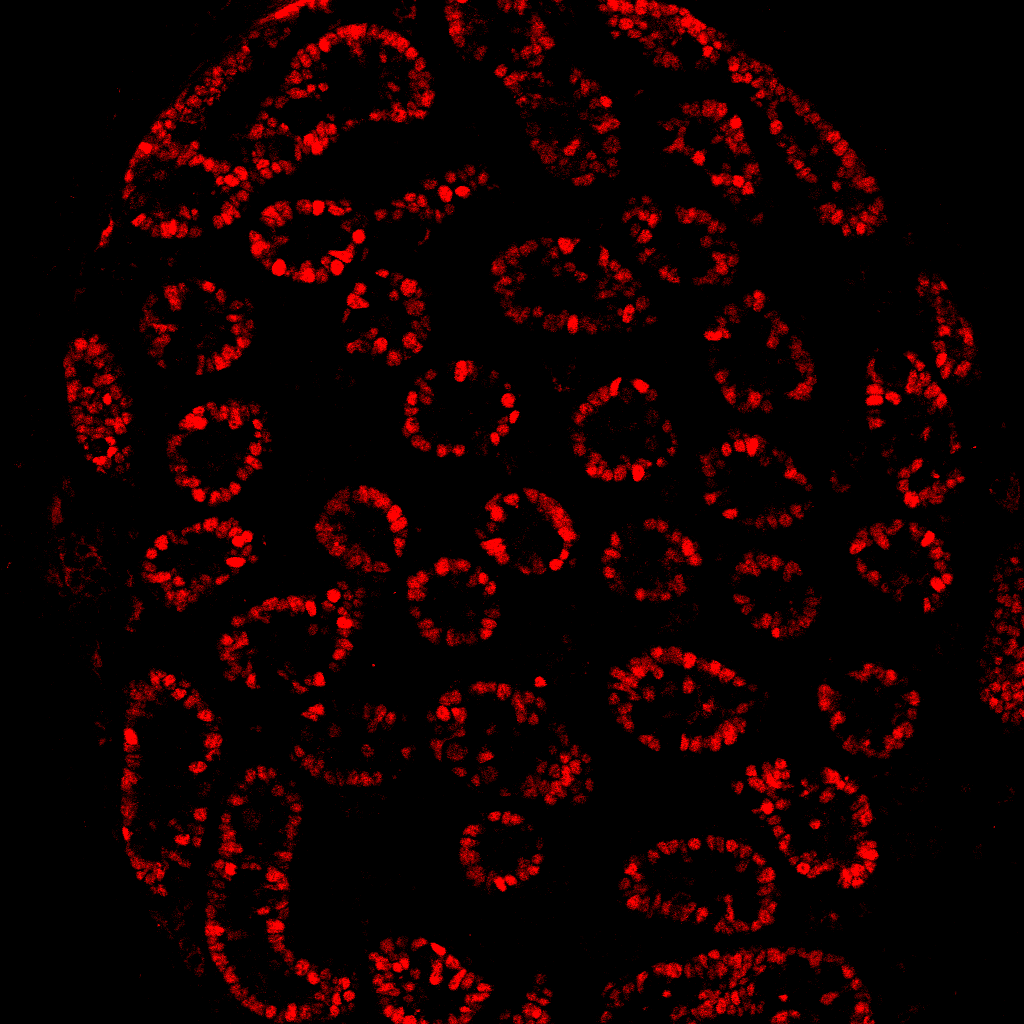

Supplement: Supplementary file 4 — Source data Fig. 1 [file 44319_2025_487_MOESM4_ESM.zip › Figure 1/1B/PD1 SOX9&γH2AX/PD1 WT testis SOX9.tif]

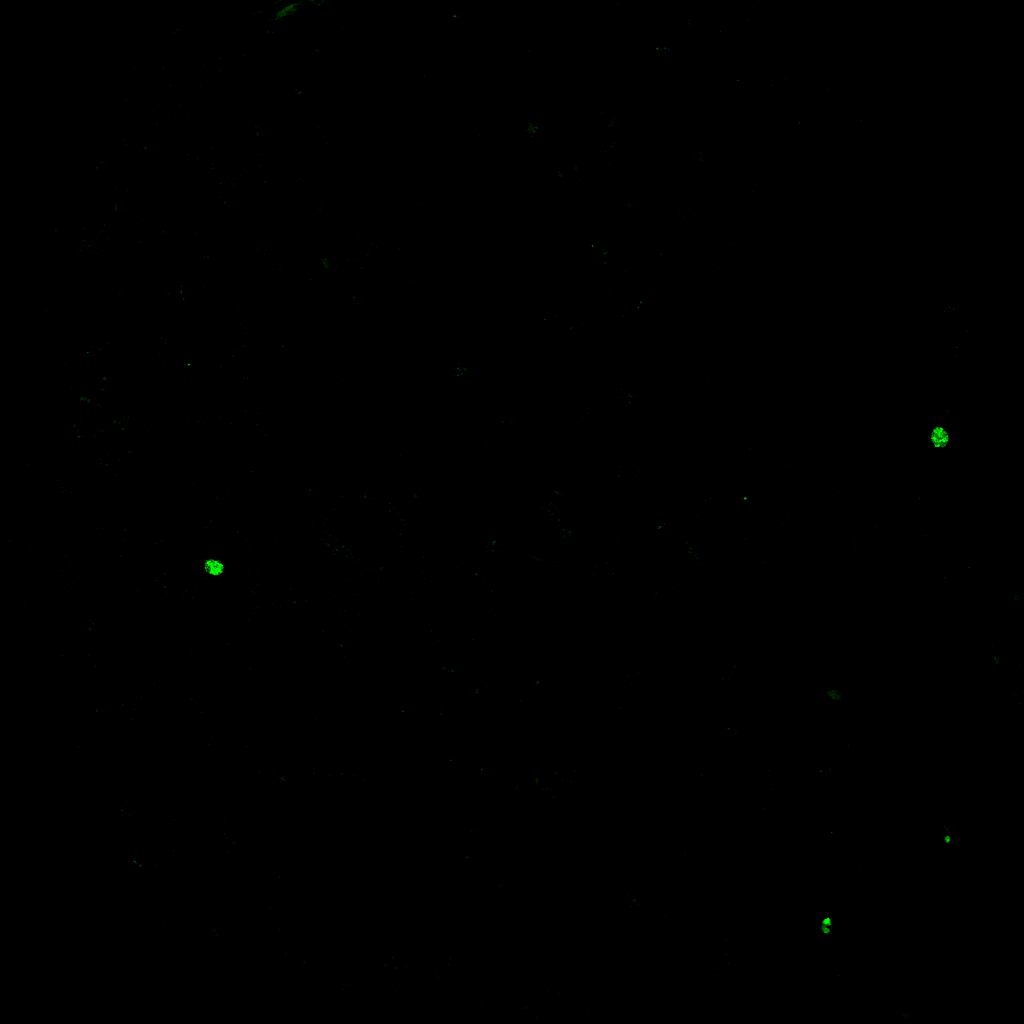

Supplement: Supplementary file 4 — Source data Fig. 1 [file 44319_2025_487_MOESM4_ESM.zip › Figure 1/1B/PD1 SOX9&γH2AX/PD1 WT testis γH2AX.tif]

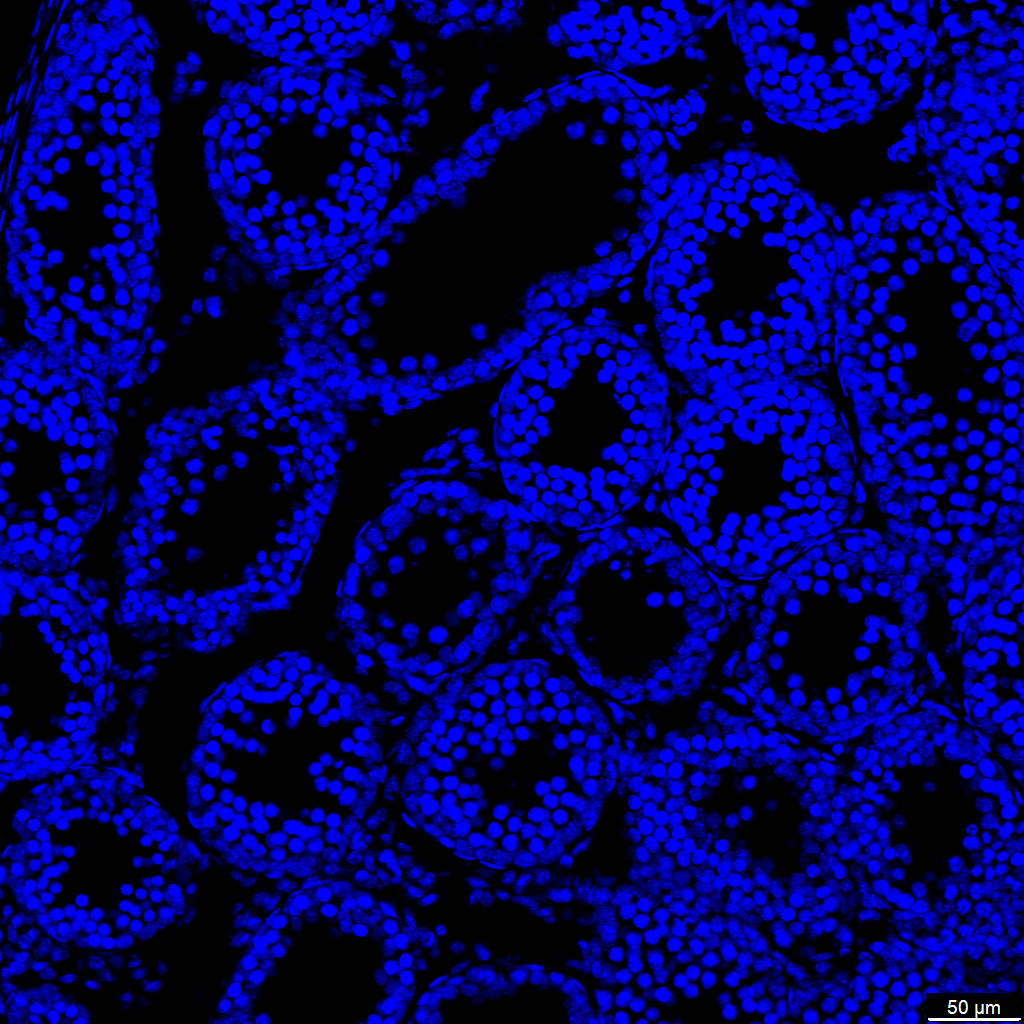

Supplement: Supplementary file 4 — Source data Fig. 1 [file 44319_2025_487_MOESM4_ESM.zip › Figure 1/1B/PD14 SOX9&γH2AX/PD14 WT testis SOX9&γH2AX Hoechst.tif]

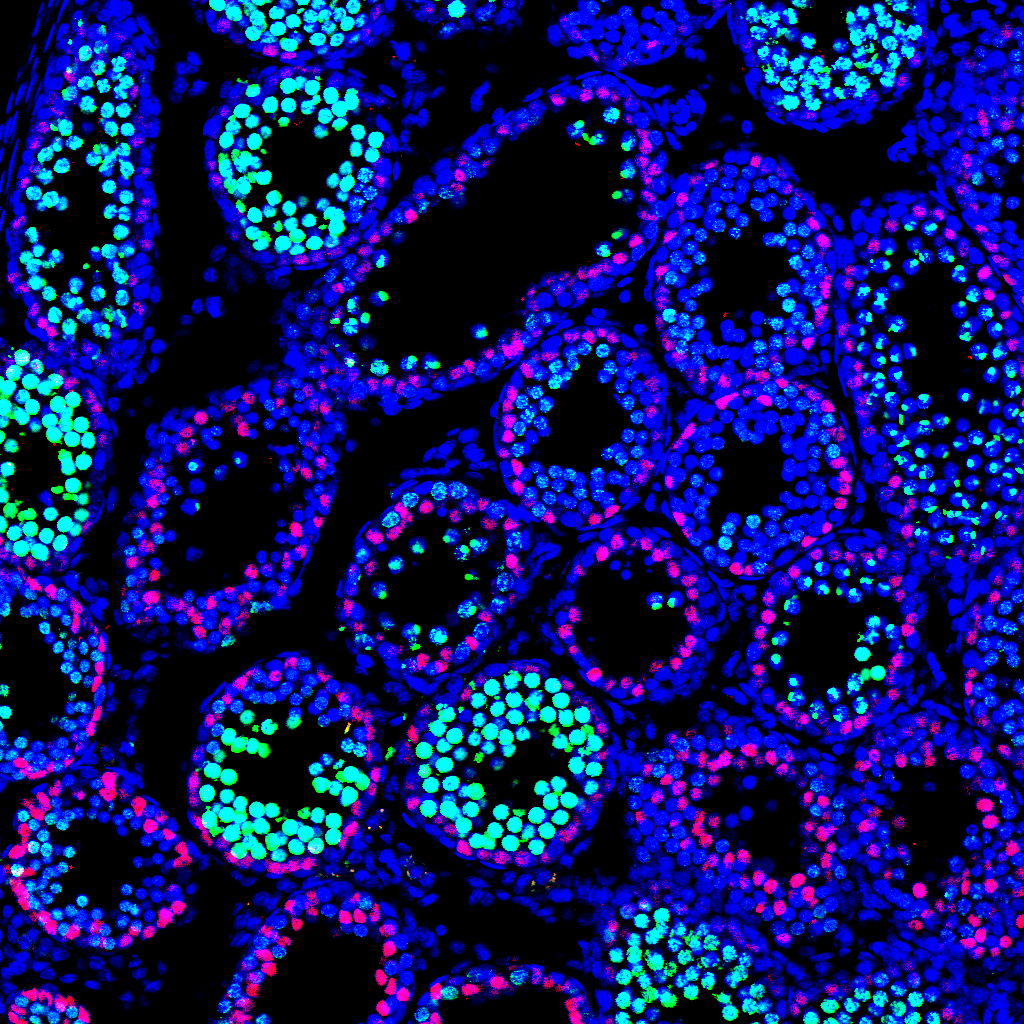

Supplement: Supplementary file 4 — Source data Fig. 1 [file 44319_2025_487_MOESM4_ESM.zip › Figure 1/1B/PD14 SOX9&γH2AX/PD14 WT testis SOX9&γH2AX Hoechst_overlay.tif]

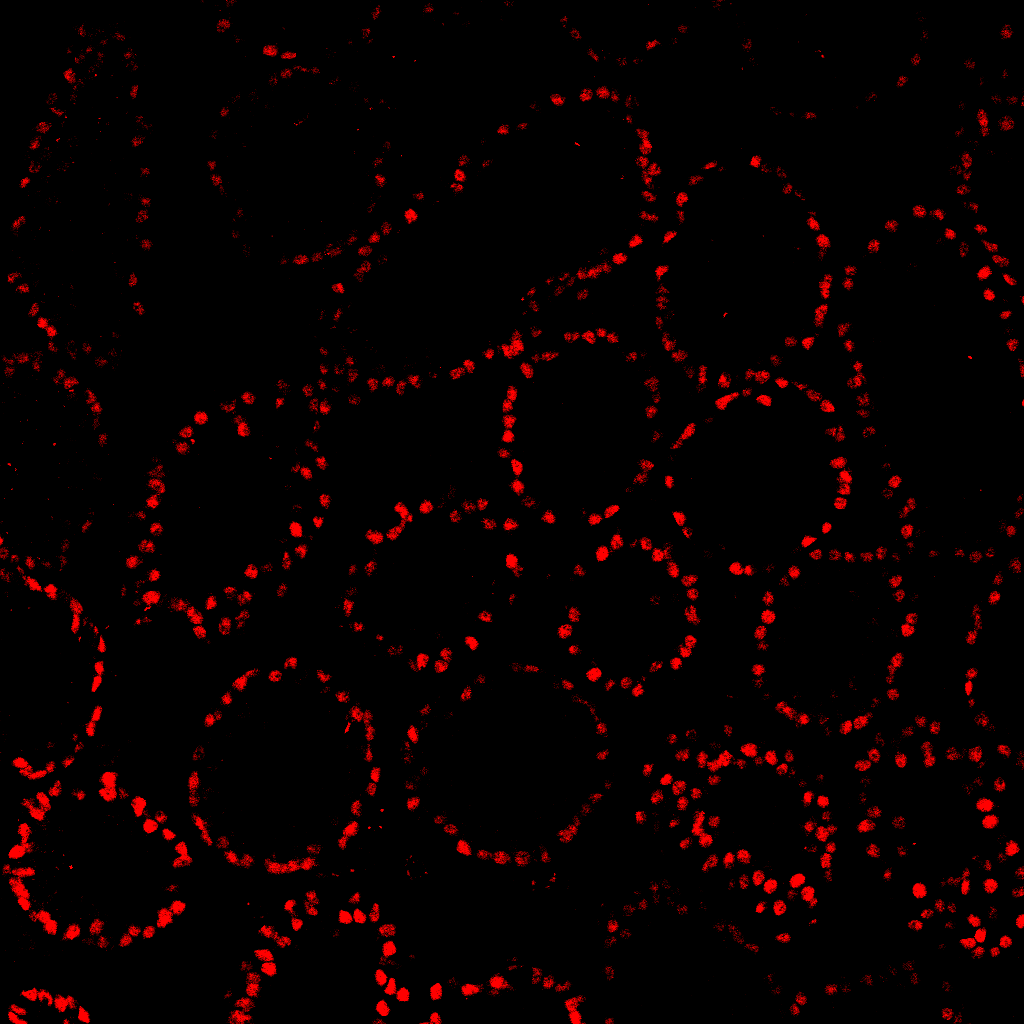

Supplement: Supplementary file 4 — Source data Fig. 1 [file 44319_2025_487_MOESM4_ESM.zip › Figure 1/1B/PD14 SOX9&γH2AX/PD14 WT testis SOX9.tif]

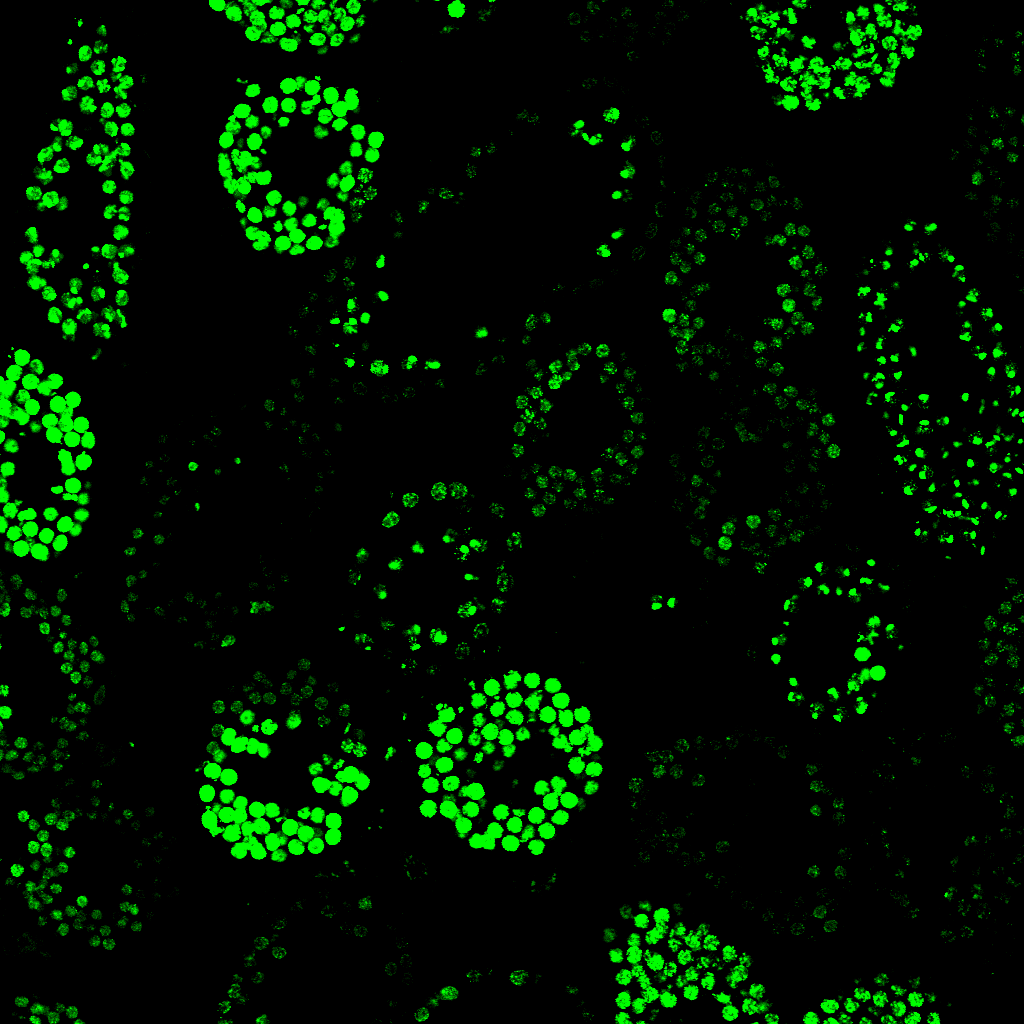

Supplement: Supplementary file 4 — Source data Fig. 1 [file 44319_2025_487_MOESM4_ESM.zip › Figure 1/1B/PD14 SOX9&γH2AX/PD14 WT testis γH2AX.tif]

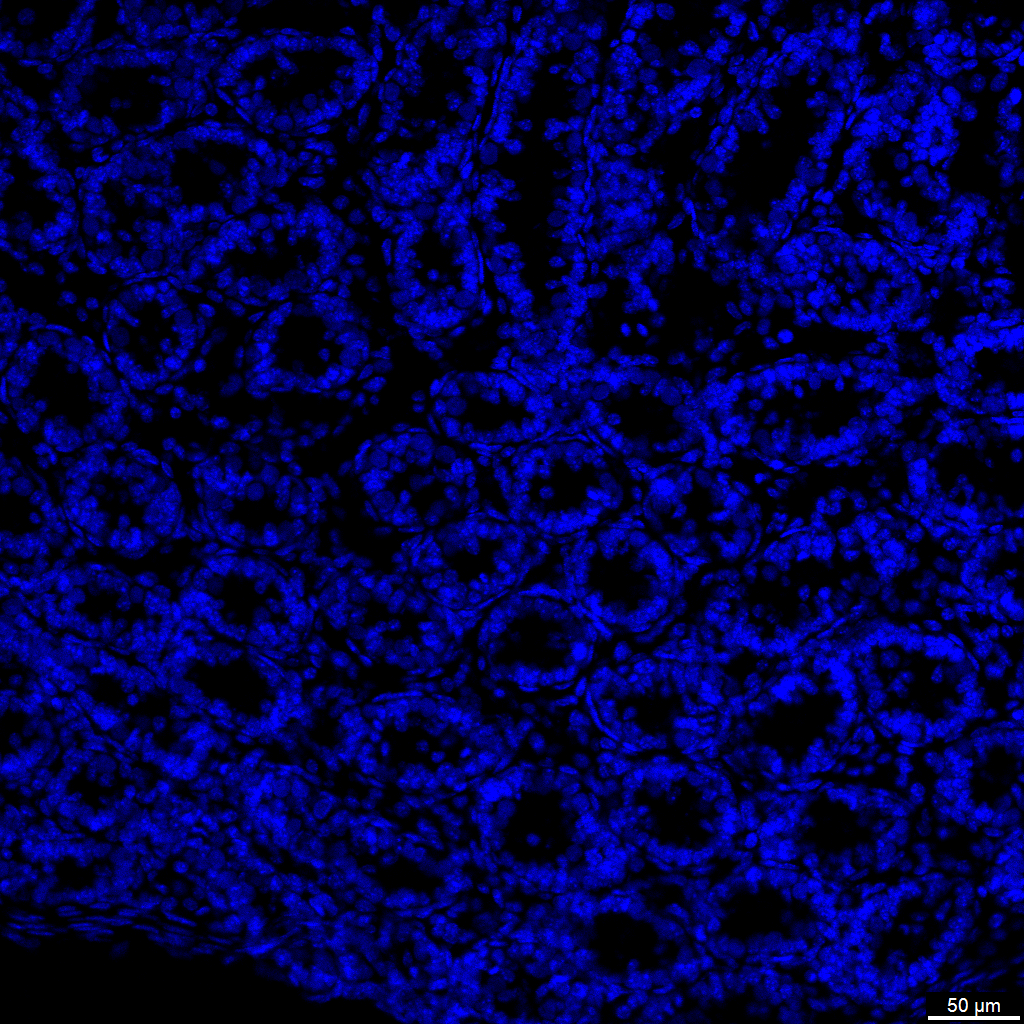

Supplement: Supplementary file 4 — Source data Fig. 1 [file 44319_2025_487_MOESM4_ESM.zip › Figure 1/1B/PD4 SOX9&γH2AX/PD4 WT testis SOX9&γH2AX Hoechst.tif]

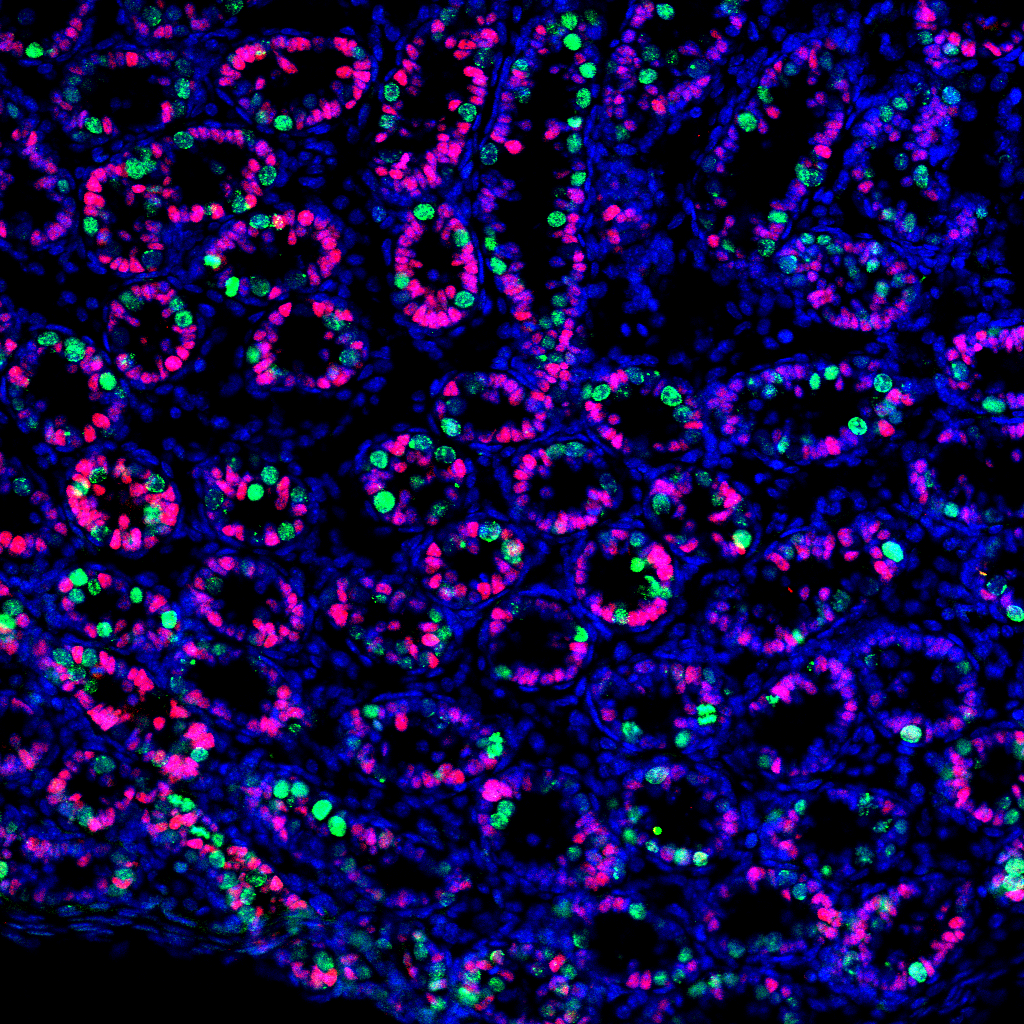

Supplement: Supplementary file 4 — Source data Fig. 1 [file 44319_2025_487_MOESM4_ESM.zip › Figure 1/1B/PD4 SOX9&γH2AX/PD4 WT testis SOX9&γH2AX_overlay.tif]

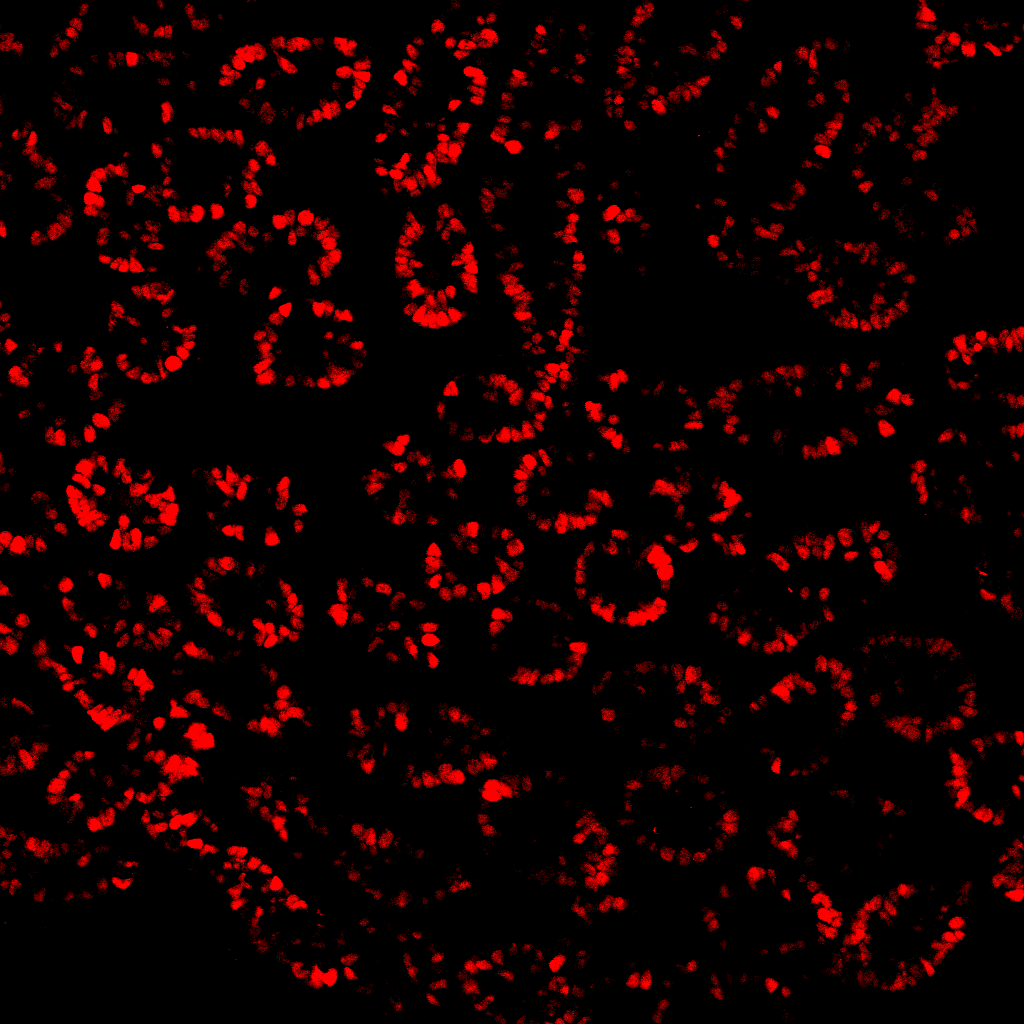

Supplement: Supplementary file 4 — Source data Fig. 1 [file 44319_2025_487_MOESM4_ESM.zip › Figure 1/1B/PD4 SOX9&γH2AX/PD4 WT testis SOX9.tif]

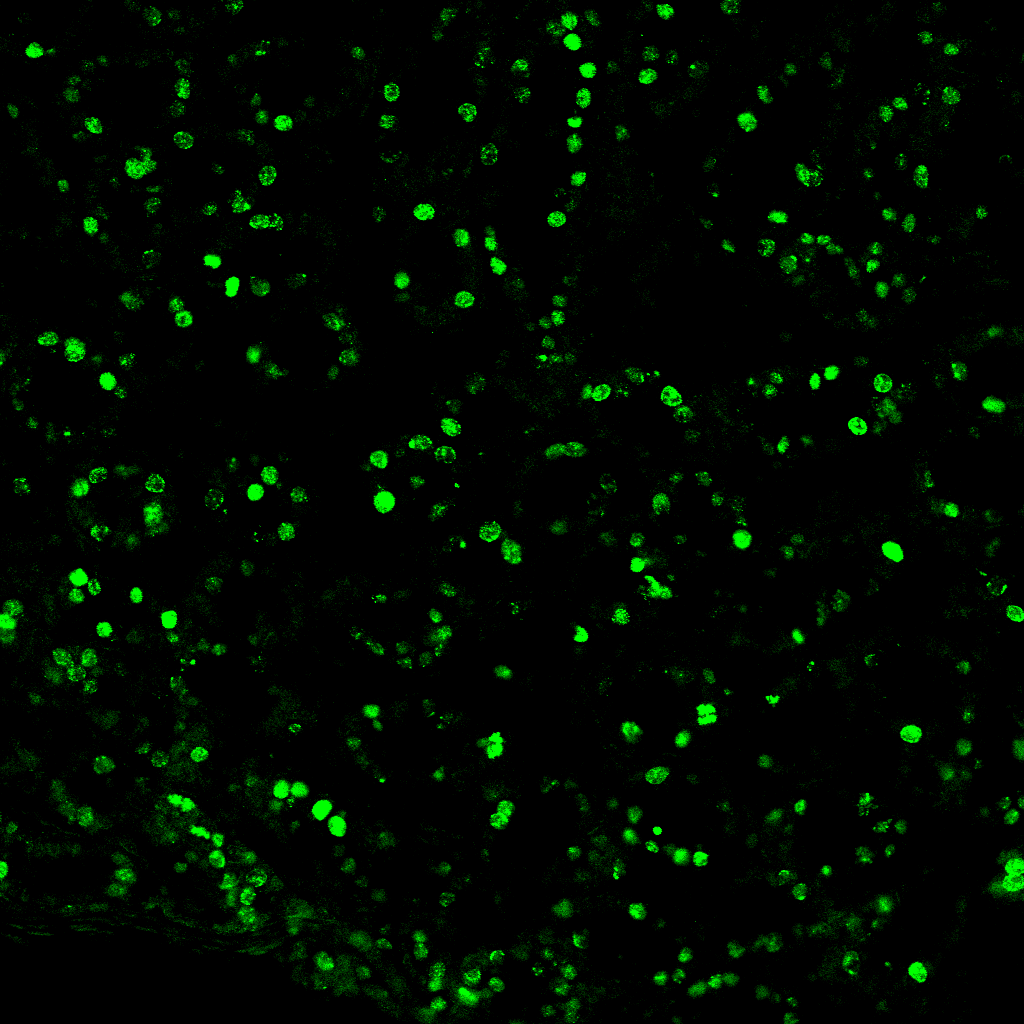

Supplement: Supplementary file 4 — Source data Fig. 1 [file 44319_2025_487_MOESM4_ESM.zip › Figure 1/1B/PD4 SOX9&γH2AX/PD4 WT testis γH2AX.tif]

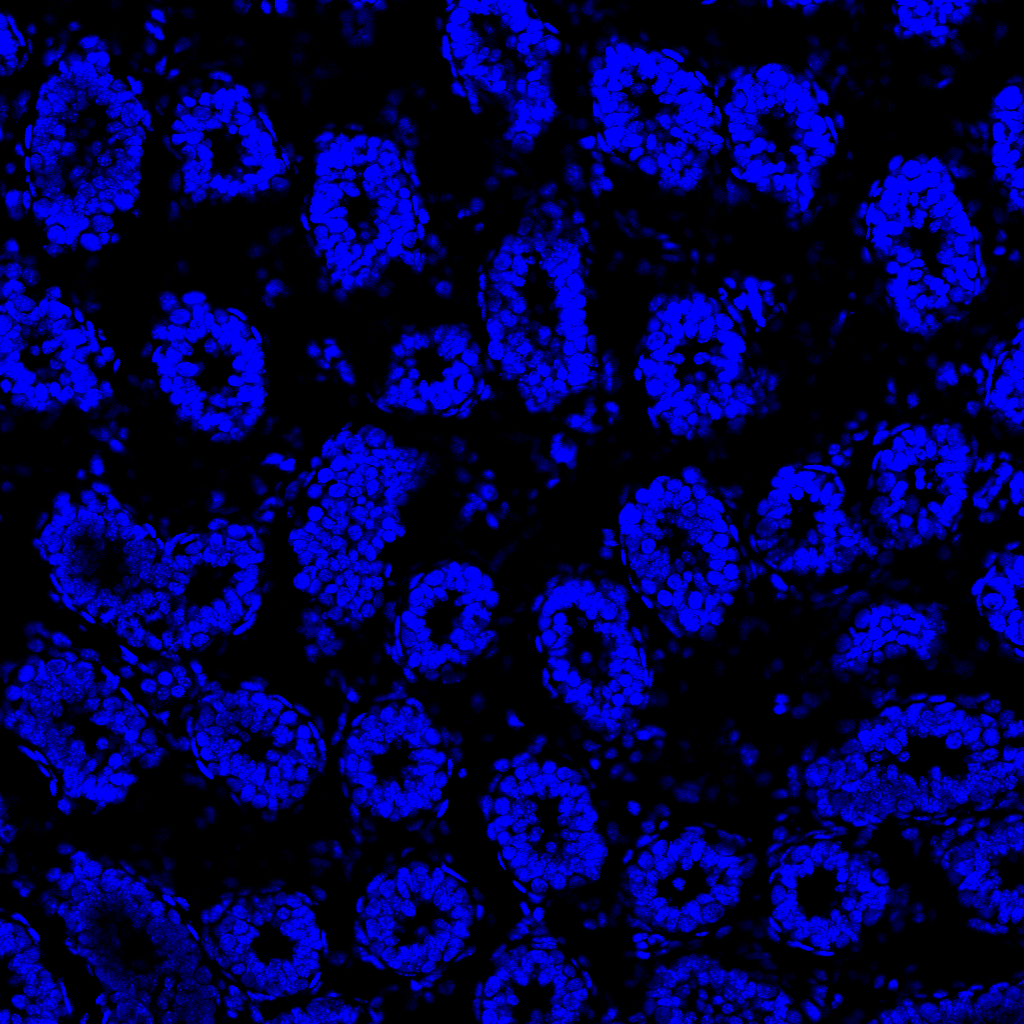

Supplement: Supplementary file 4 — Source data Fig. 1 [file 44319_2025_487_MOESM4_ESM.zip › Figure 1/1B/PD7 SOX9&γH2AX/PD7 WT testis SOX9&γH2AX Hoechst.tif]

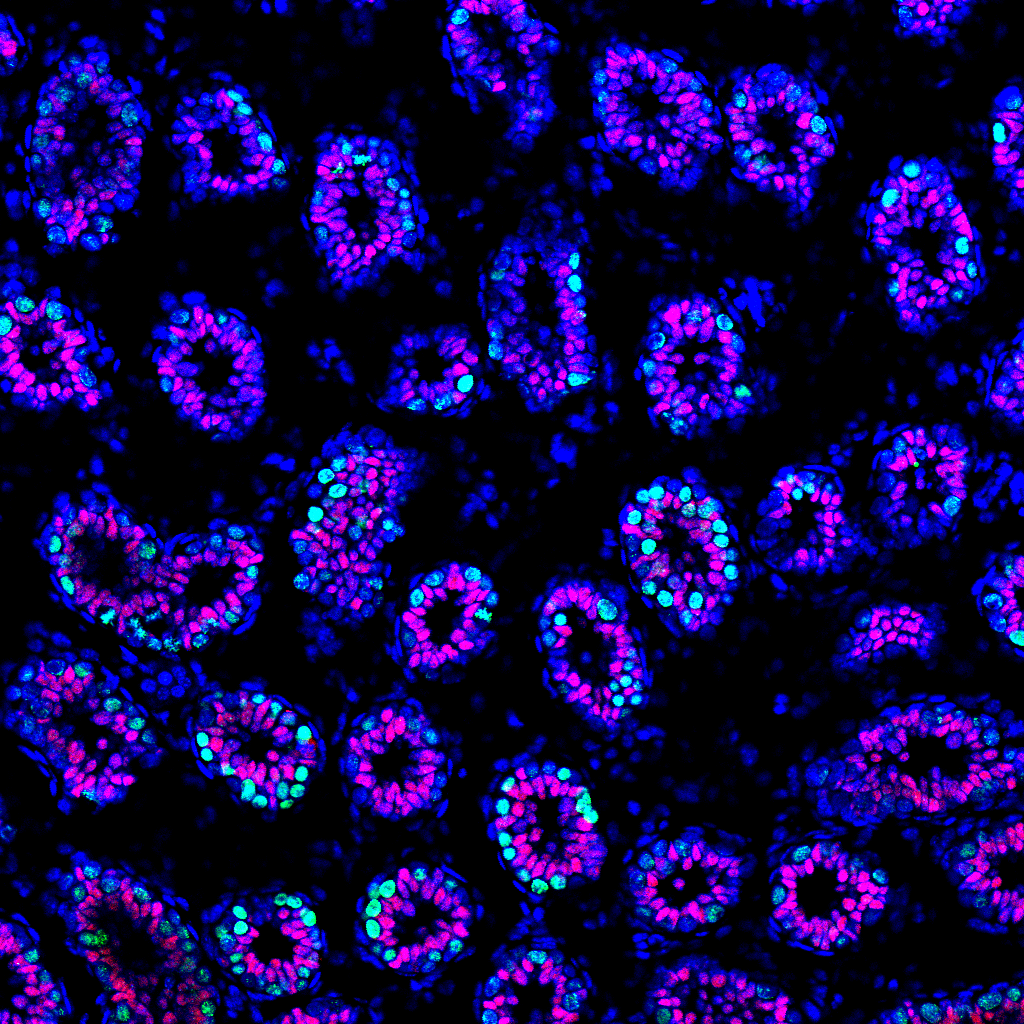

Supplement: Supplementary file 4 — Source data Fig. 1 [file 44319_2025_487_MOESM4_ESM.zip › Figure 1/1B/PD7 SOX9&γH2AX/PD7 WT testis SOX9&γH2AX Hoechst_overlay.tif]

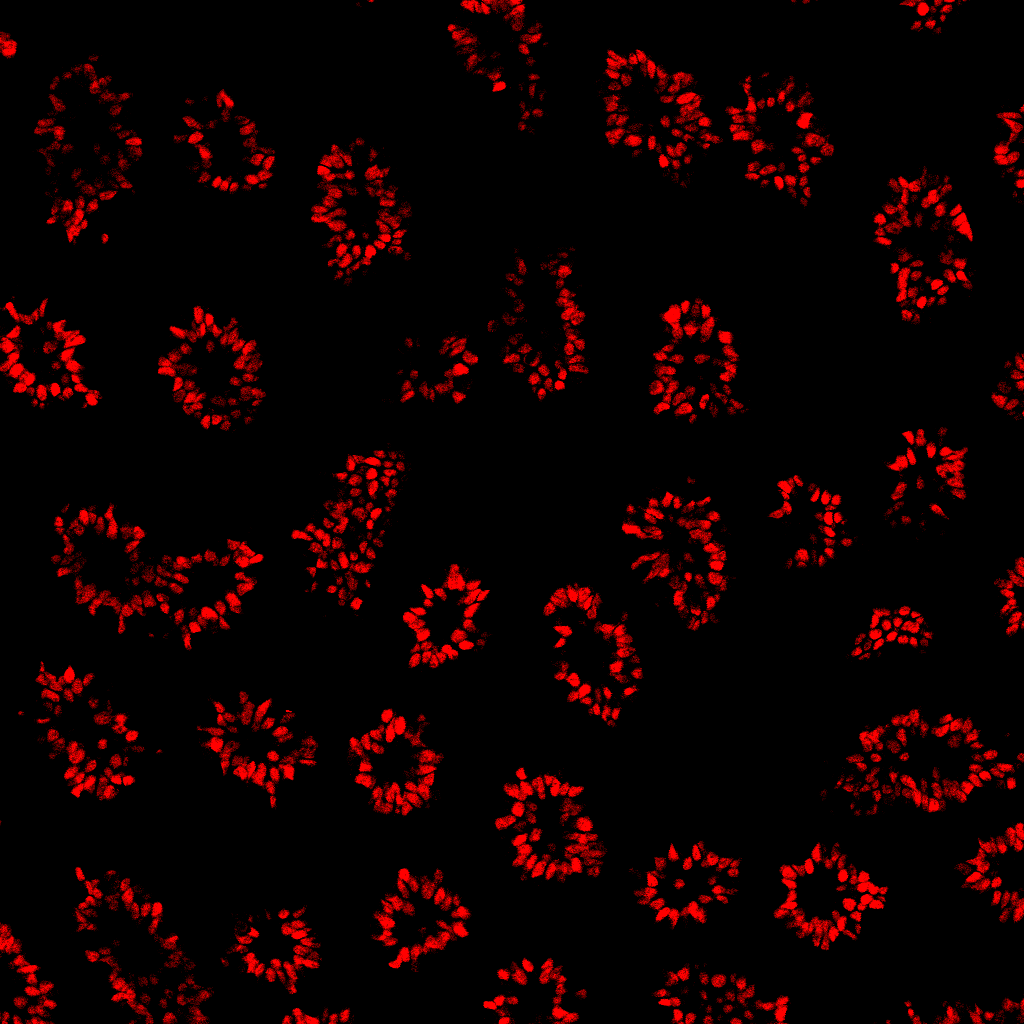

Supplement: Supplementary file 4 — Source data Fig. 1 [file 44319_2025_487_MOESM4_ESM.zip › Figure 1/1B/PD7 SOX9&γH2AX/PD7 WT testis SOX9.tif]

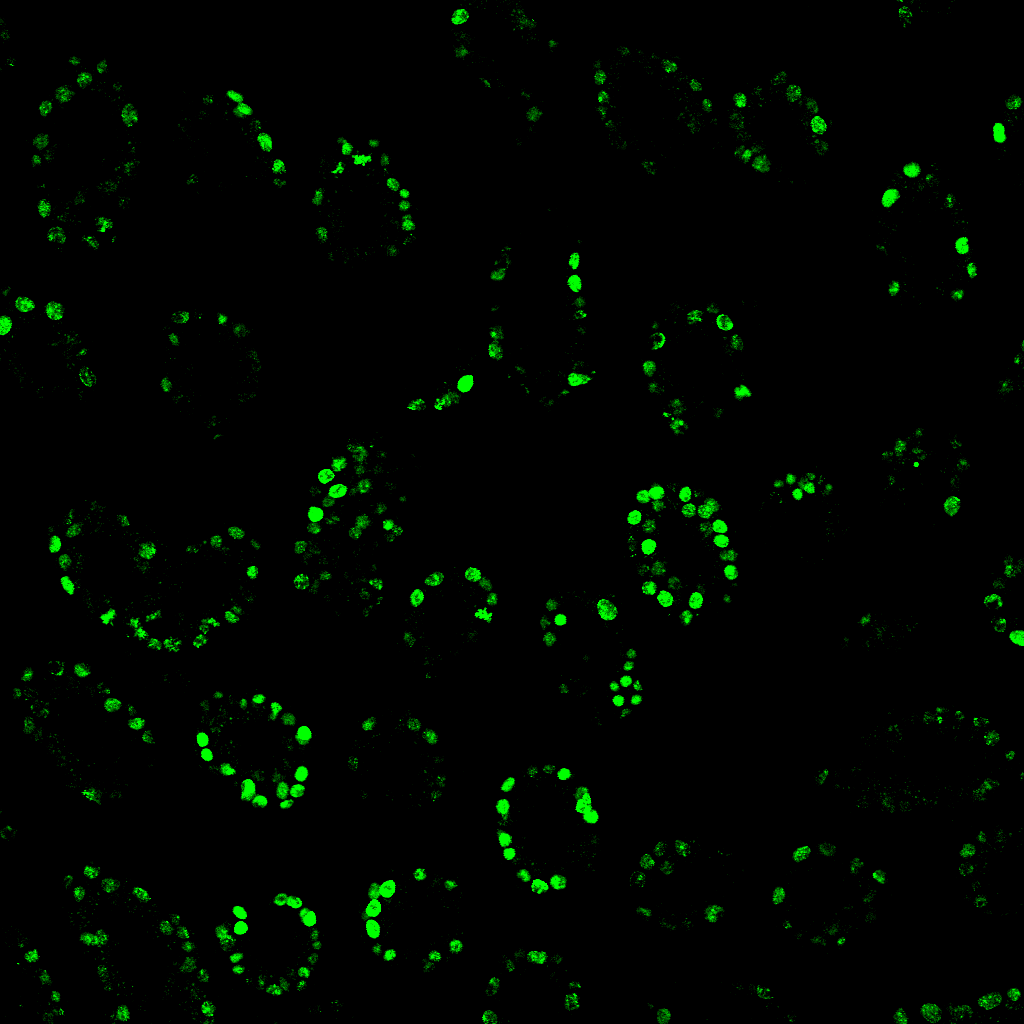

Supplement: Supplementary file 4 — Source data Fig. 1 [file 44319_2025_487_MOESM4_ESM.zip › Figure 1/1B/PD7 SOX9&γH2AX/PD7 WT testis γH2AX.tif]

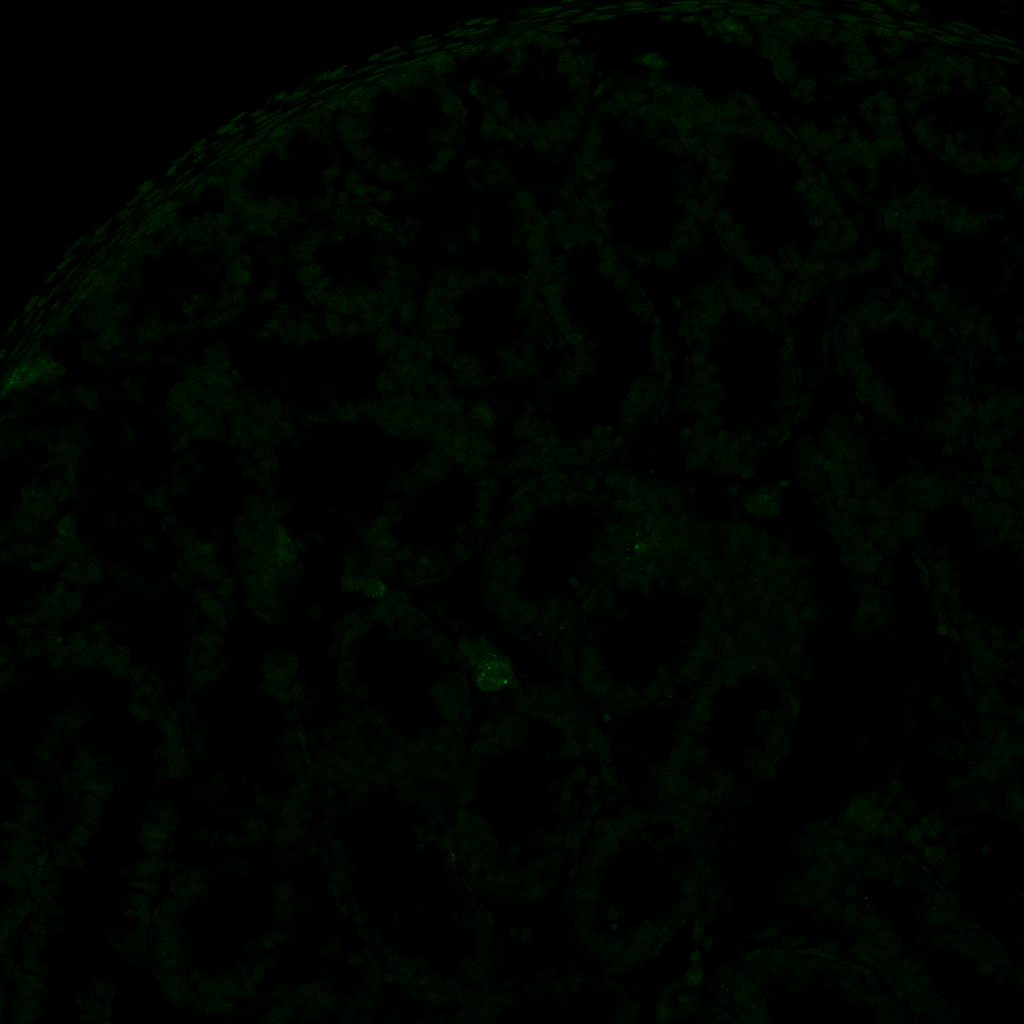

Supplement: Supplementary file 4 — Source data Fig. 1 [file 44319_2025_487_MOESM4_ESM.zip › Figure 1/1D/PD4 PLZF&cleaved PARP1/PD4 WT testis cleaved PARP1.tif]

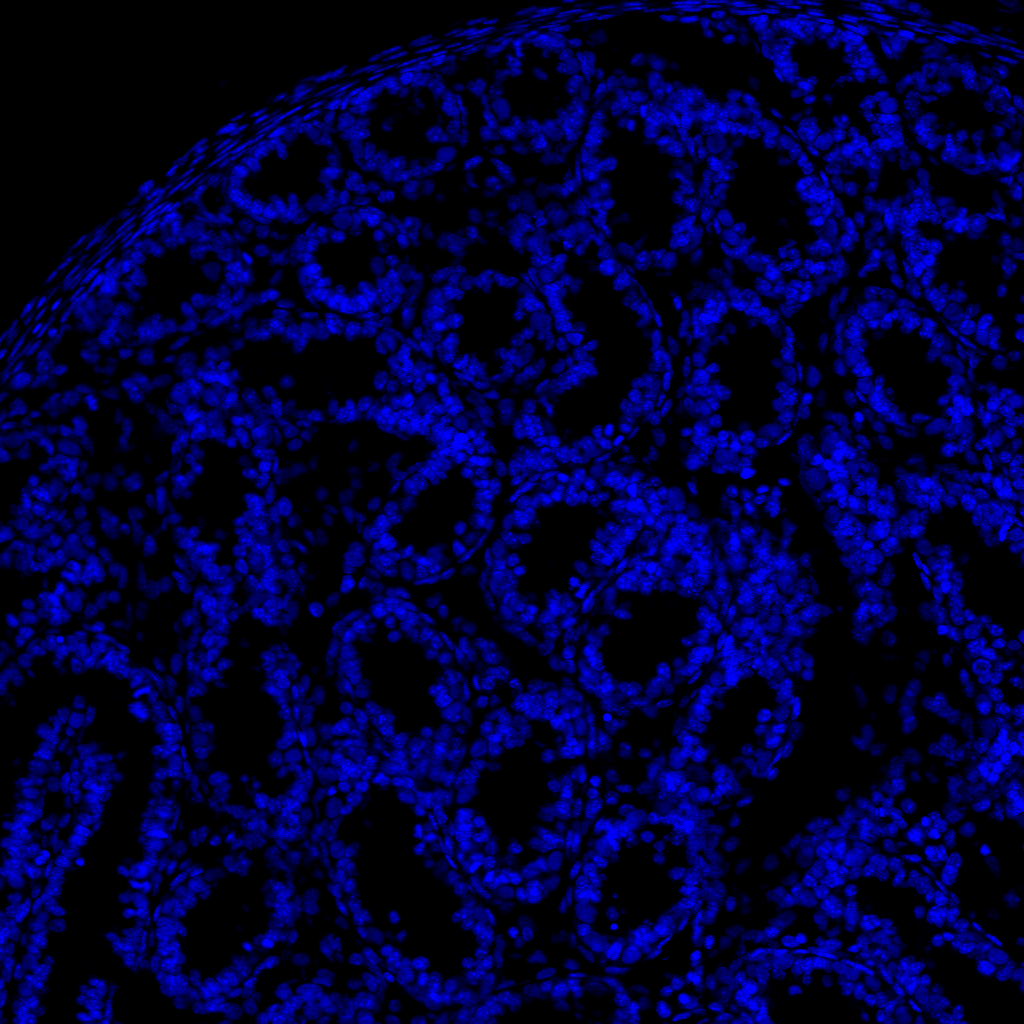

Supplement: Supplementary file 4 — Source data Fig. 1 [file 44319_2025_487_MOESM4_ESM.zip › Figure 1/1D/PD4 PLZF&cleaved PARP1/PD4 WT testis PLZF&cleaved PARP1 Hoechst.tif]

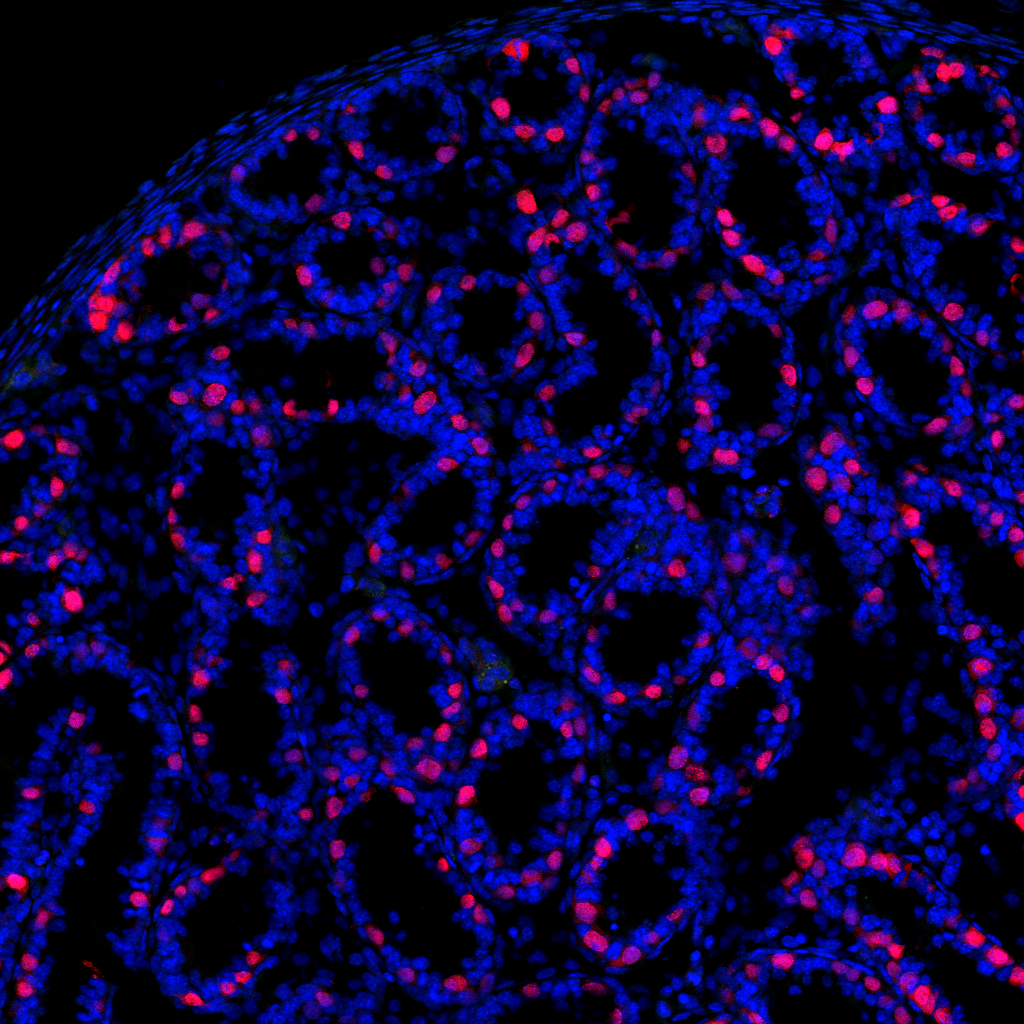

Supplement: Supplementary file 4 — Source data Fig. 1 [file 44319_2025_487_MOESM4_ESM.zip › Figure 1/1D/PD4 PLZF&cleaved PARP1/PD4 WT testis PLZF&cleaved PARP1 Hoechst_overlay.tif]

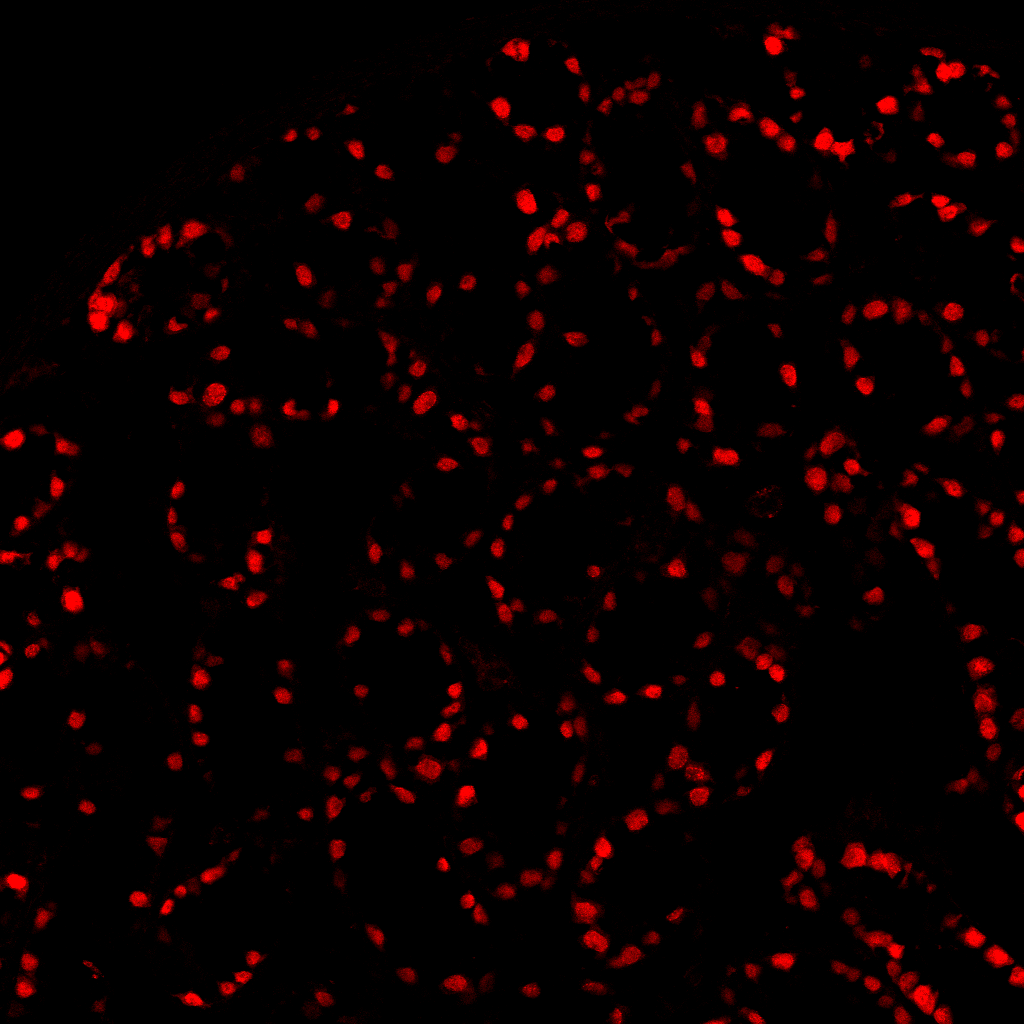

Supplement: Supplementary file 4 — Source data Fig. 1 [file 44319_2025_487_MOESM4_ESM.zip › Figure 1/1D/PD4 PLZF&cleaved PARP1/PD4 WT testis PLZF.tif]

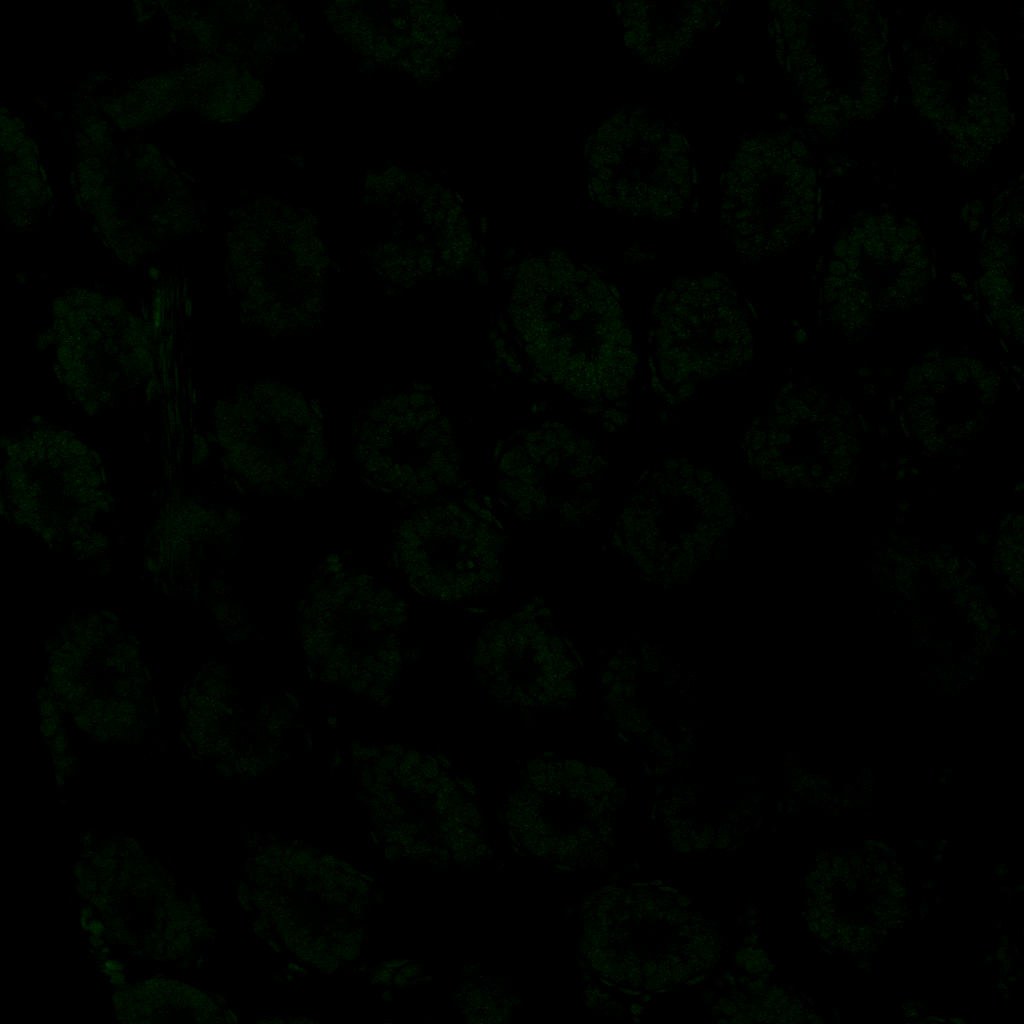

Supplement: Supplementary file 4 — Source data Fig. 1 [file 44319_2025_487_MOESM4_ESM.zip › Figure 1/1D/PD7 PLZF&cleaved PARP1/PD7 WT testis cleaved PARP1.tif]

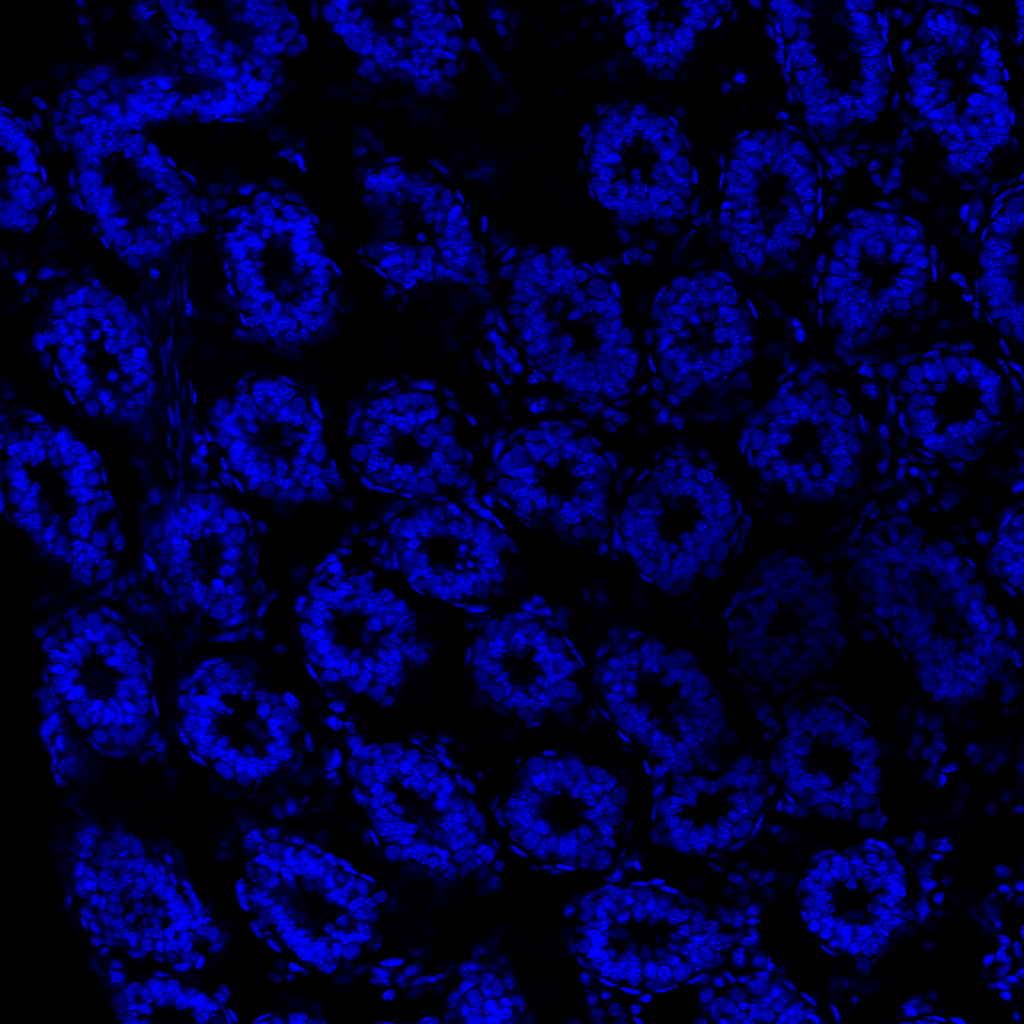

Supplement: Supplementary file 4 — Source data Fig. 1 [file 44319_2025_487_MOESM4_ESM.zip › Figure 1/1D/PD7 PLZF&cleaved PARP1/PD7 WT testis PLZF&cleaved PARP1 Hoechst.tif]

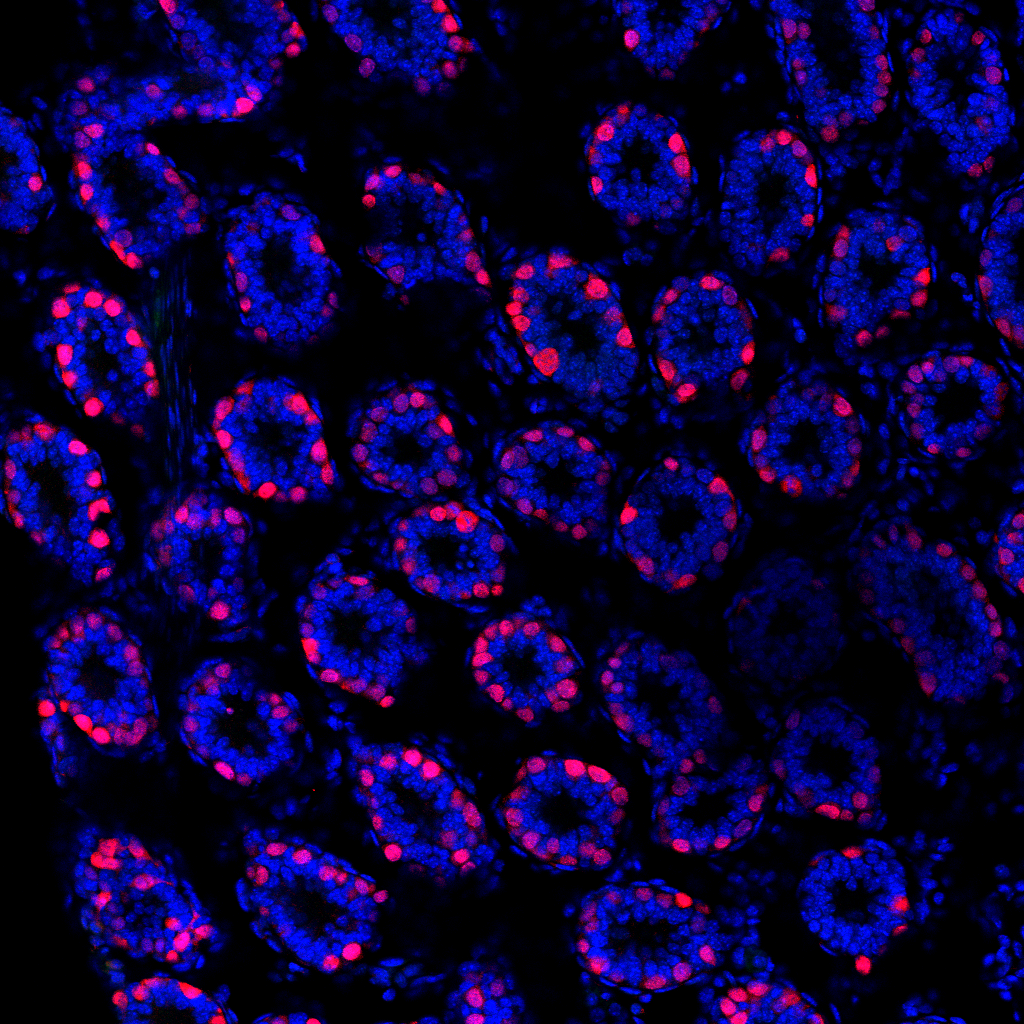

Supplement: Supplementary file 4 — Source data Fig. 1 [file 44319_2025_487_MOESM4_ESM.zip › Figure 1/1D/PD7 PLZF&cleaved PARP1/PD7 WT testis PLZF&cleaved PARP1 Hoechst_overlay.tif]

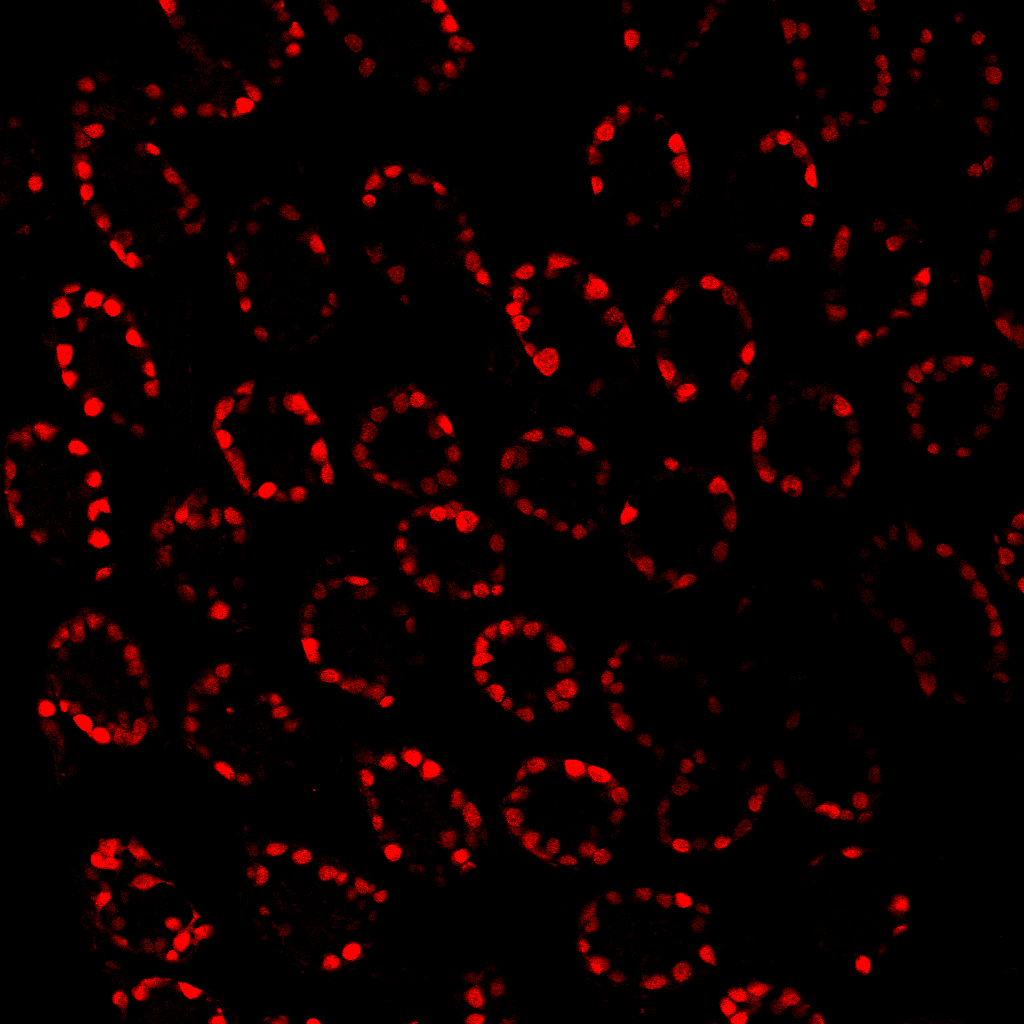

Supplement: Supplementary file 4 — Source data Fig. 1 [file 44319_2025_487_MOESM4_ESM.zip › Figure 1/1D/PD7 PLZF&cleaved PARP1/PD7 WT testis PLZF.tif]

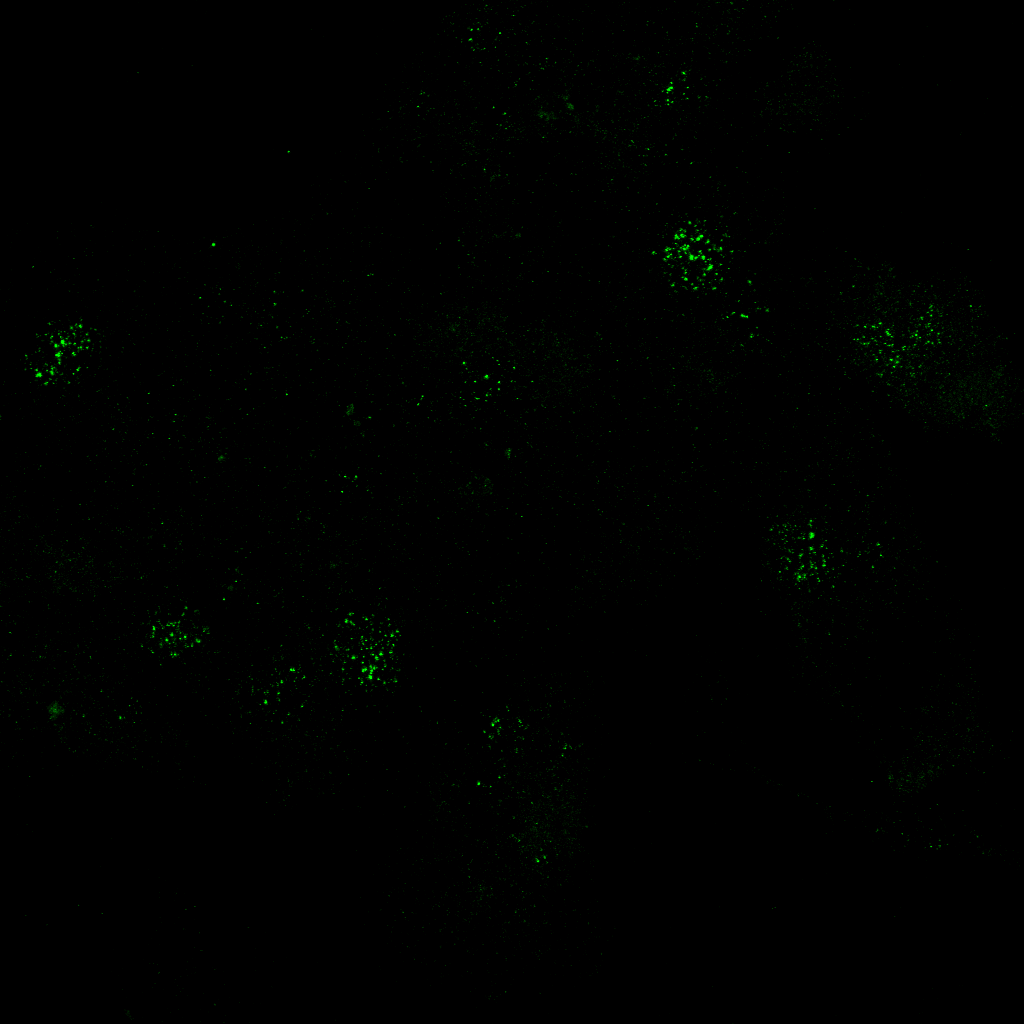

Supplement: Supplementary file 5 — Source data Fig. 2 [file 44319_2025_487_MOESM5_ESM.zip › Figure 2/2A/PD1 Brca1 vKO testicular cell IR treatment anti-GCNA&BRCA1/Anti-BRCA1.tif]

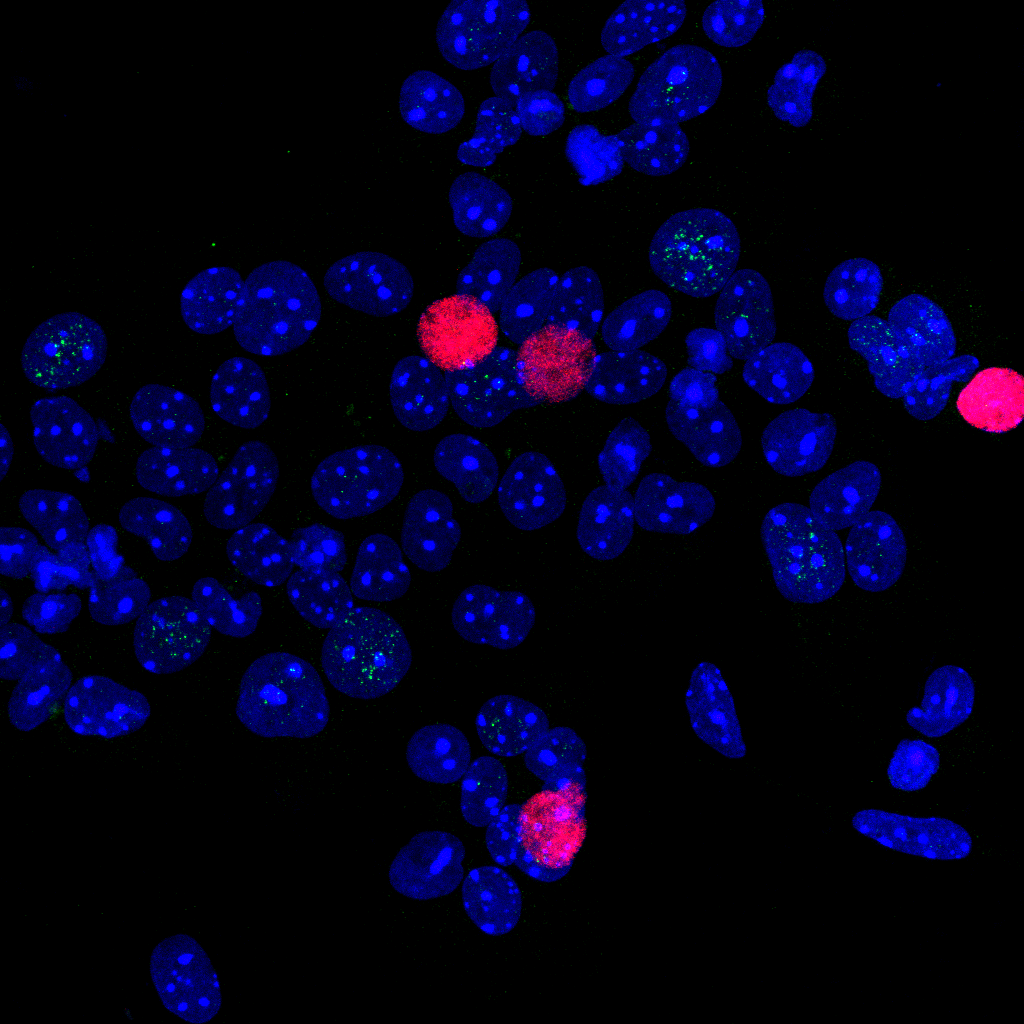

Supplement: Supplementary file 5 — Source data Fig. 2 [file 44319_2025_487_MOESM5_ESM.zip › Figure 2/2A/PD1 Brca1 vKO testicular cell IR treatment anti-GCNA&BRCA1/Anti-GCNA&BRCA overlay.tif]

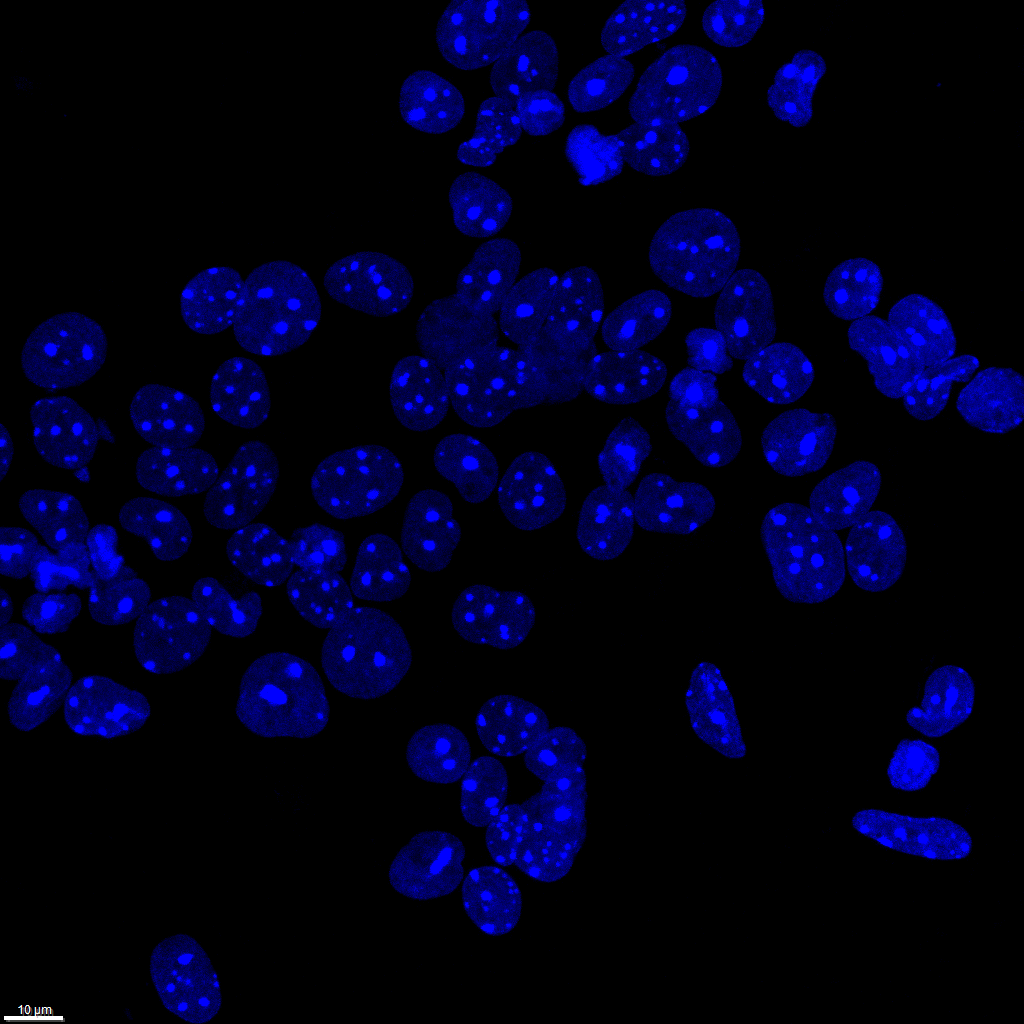

Supplement: Supplementary file 5 — Source data Fig. 2 [file 44319_2025_487_MOESM5_ESM.zip › Figure 2/2A/PD1 Brca1 vKO testicular cell IR treatment anti-GCNA&BRCA1/Anti-GCNA&BRCA1 Hoechst.tif]

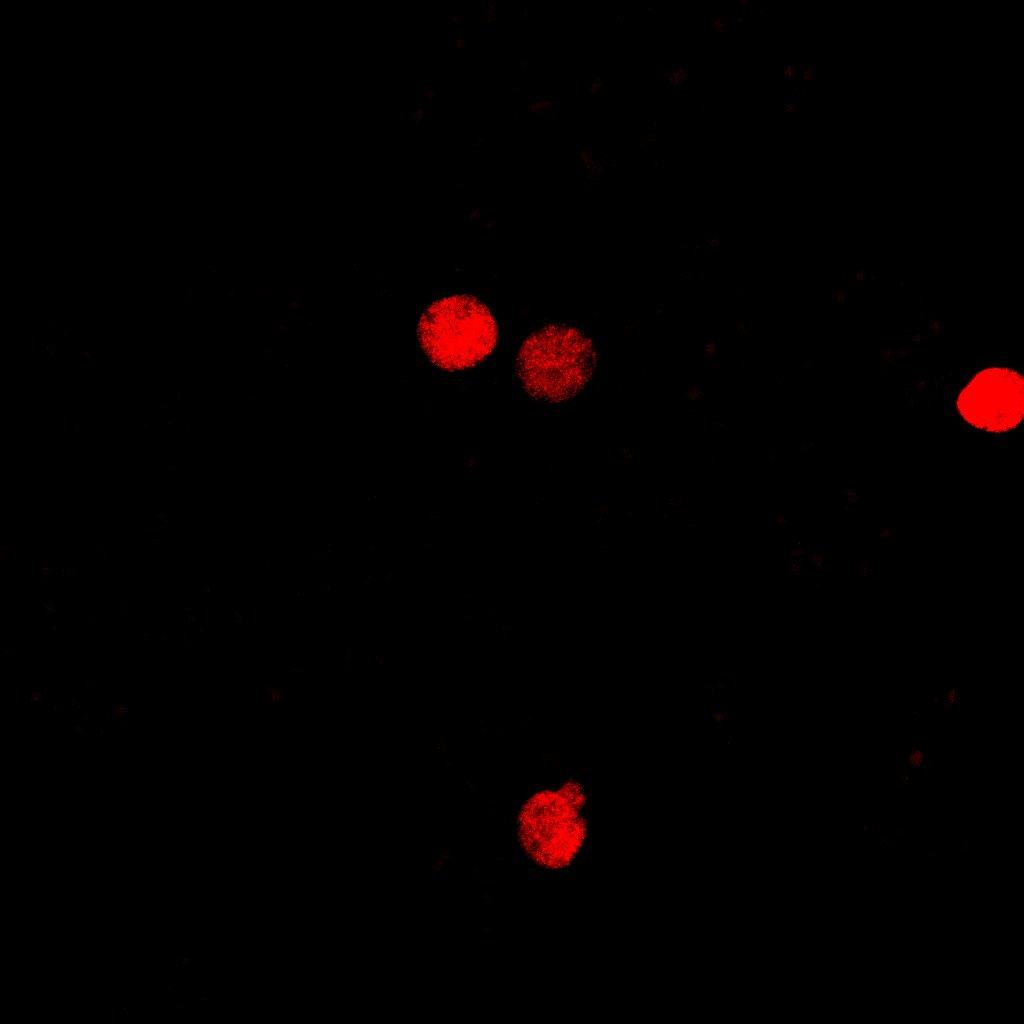

Supplement: Supplementary file 5 — Source data Fig. 2 [file 44319_2025_487_MOESM5_ESM.zip › Figure 2/2A/PD1 Brca1 vKO testicular cell IR treatment anti-GCNA&BRCA1/Anti-GCNA.tif]

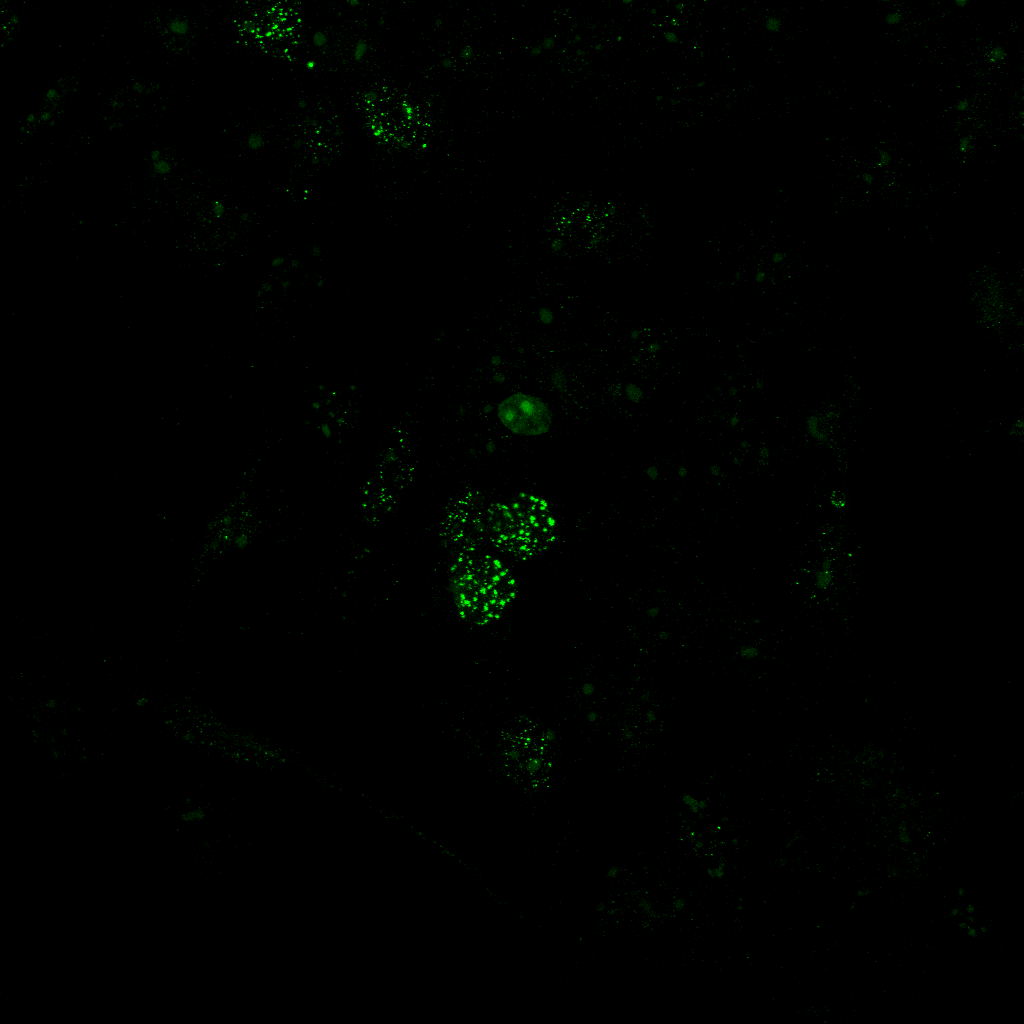

Supplement: Supplementary file 5 — Source data Fig. 2 [file 44319_2025_487_MOESM5_ESM.zip › Figure 2/2A/PD1 Control testicular cell IR treatment GCNA&BRCA1/Anti-BRCA1.tif]

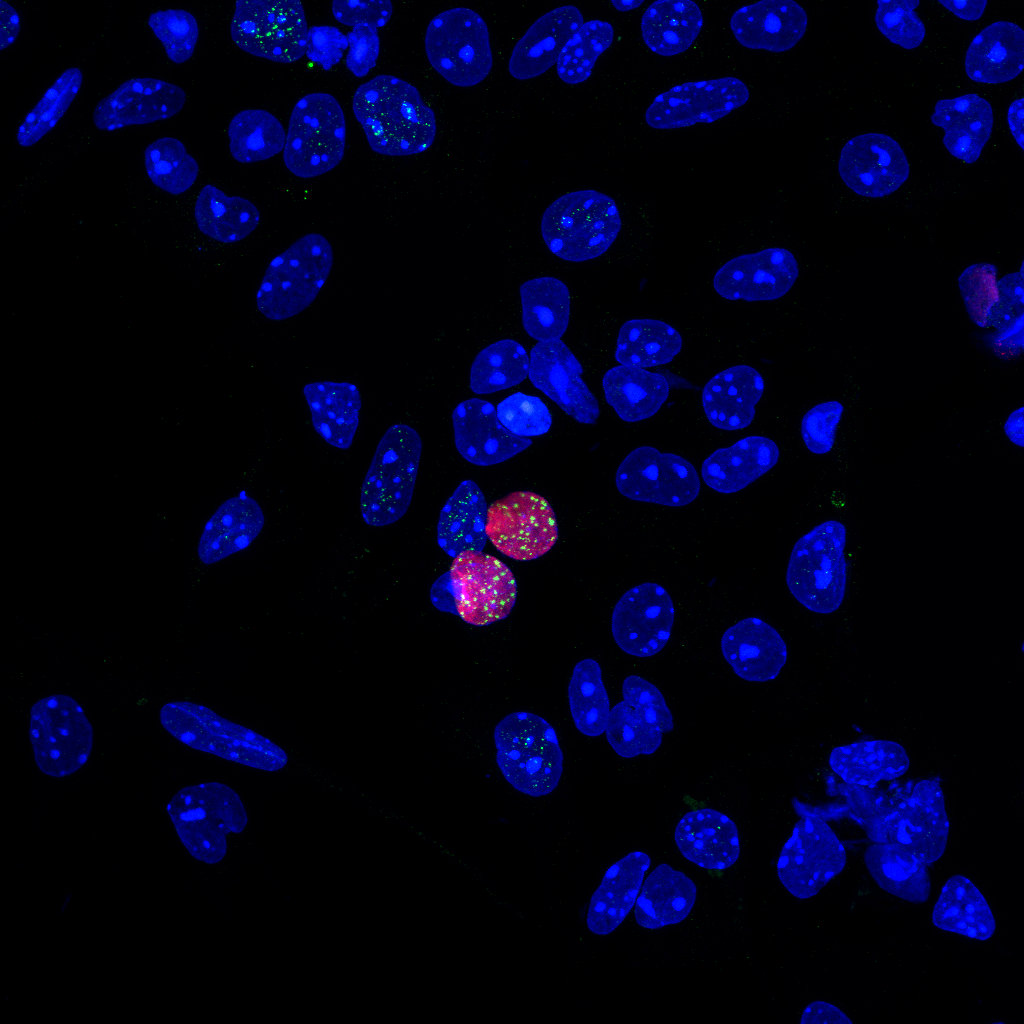

Supplement: Supplementary file 5 — Source data Fig. 2 [file 44319_2025_487_MOESM5_ESM.zip › Figure 2/2A/PD1 Control testicular cell IR treatment GCNA&BRCA1/Anti-GCNA&BRCA overlay.tif]

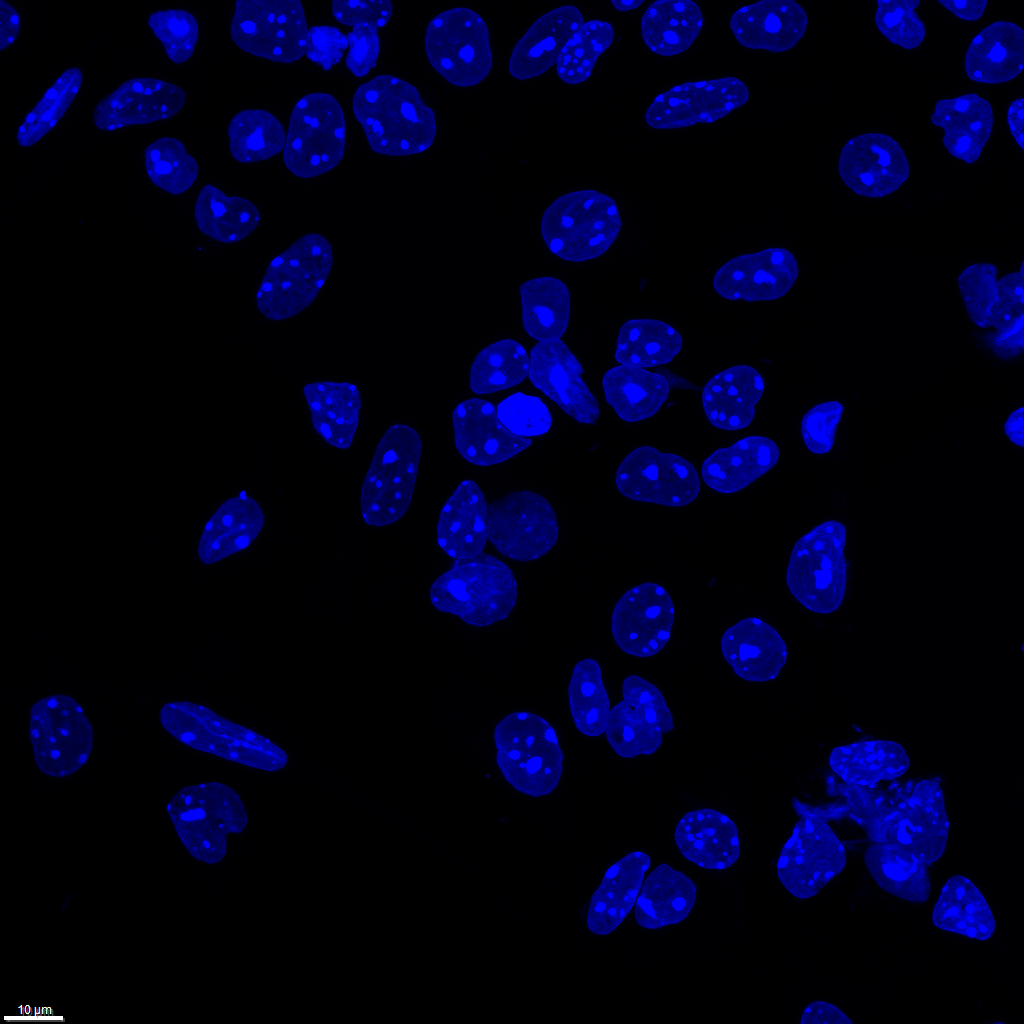

Supplement: Supplementary file 5 — Source data Fig. 2 [file 44319_2025_487_MOESM5_ESM.zip › Figure 2/2A/PD1 Control testicular cell IR treatment GCNA&BRCA1/Anti-GCNA&BRCA1 Hoechst.tif]

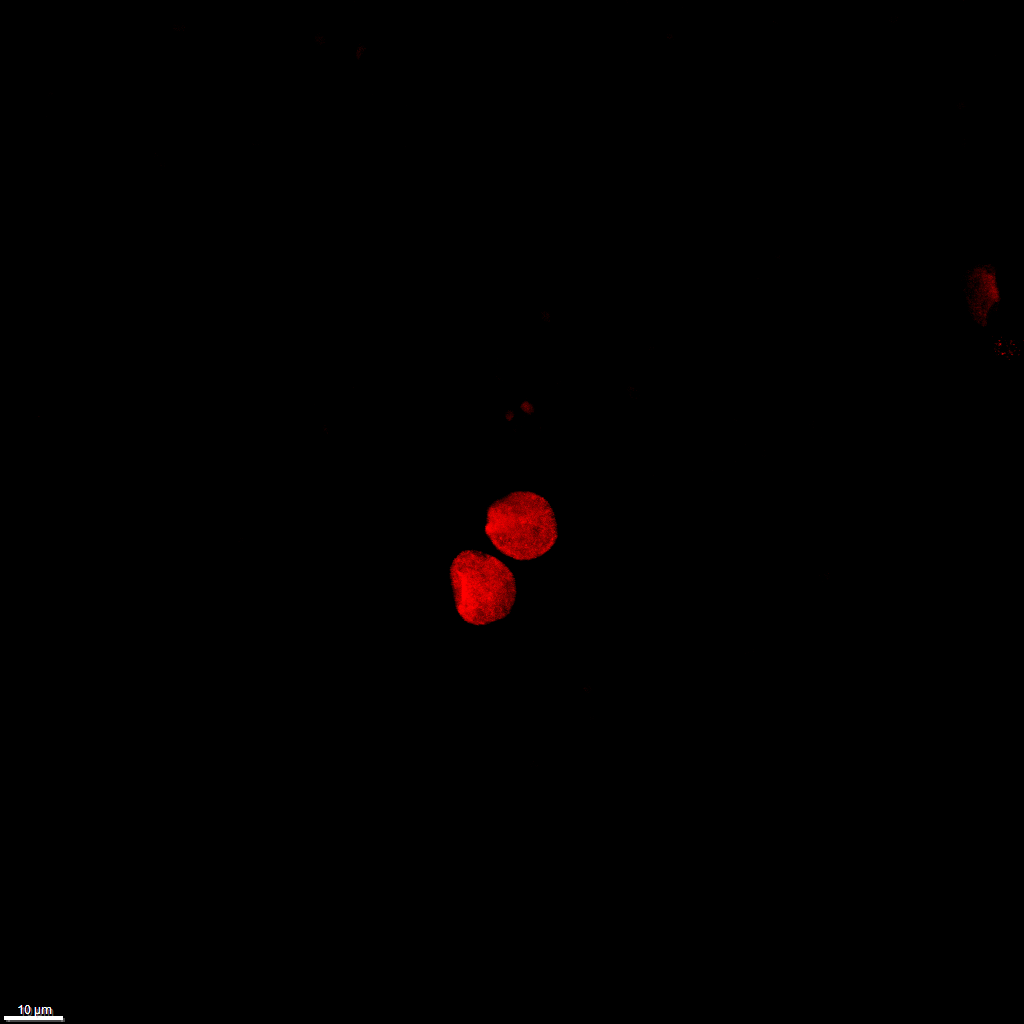

Supplement: Supplementary file 5 — Source data Fig. 2 [file 44319_2025_487_MOESM5_ESM.zip › Figure 2/2A/PD1 Control testicular cell IR treatment GCNA&BRCA1/Anti-GCNA.tif]

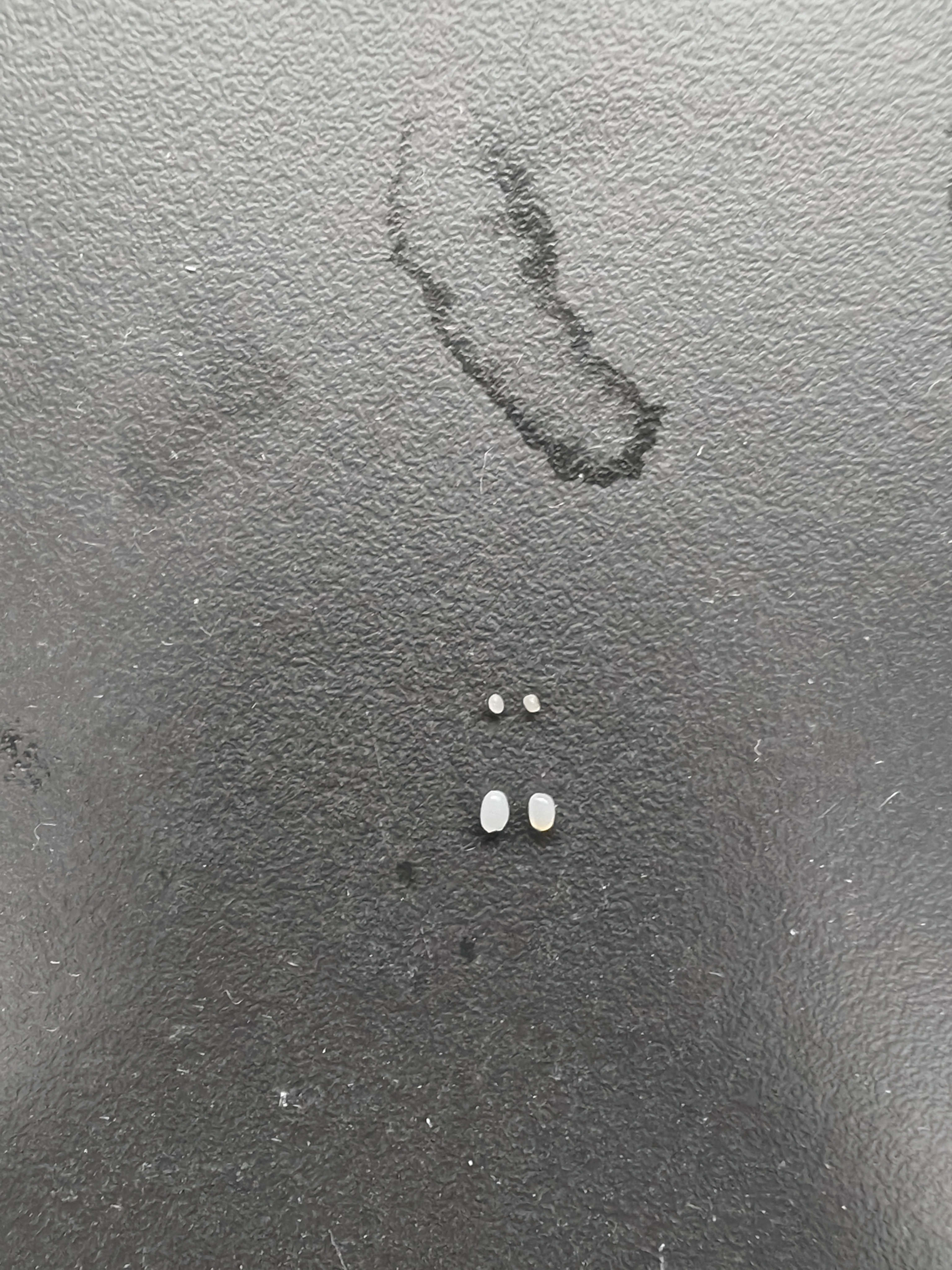

Supplement: Supplementary file 5 — Source data Fig. 2 [file 44319_2025_487_MOESM5_ESM.zip › Figure 2/2B/PD1 and PD7 BRCA1 vasa-cre male testis.jpg]

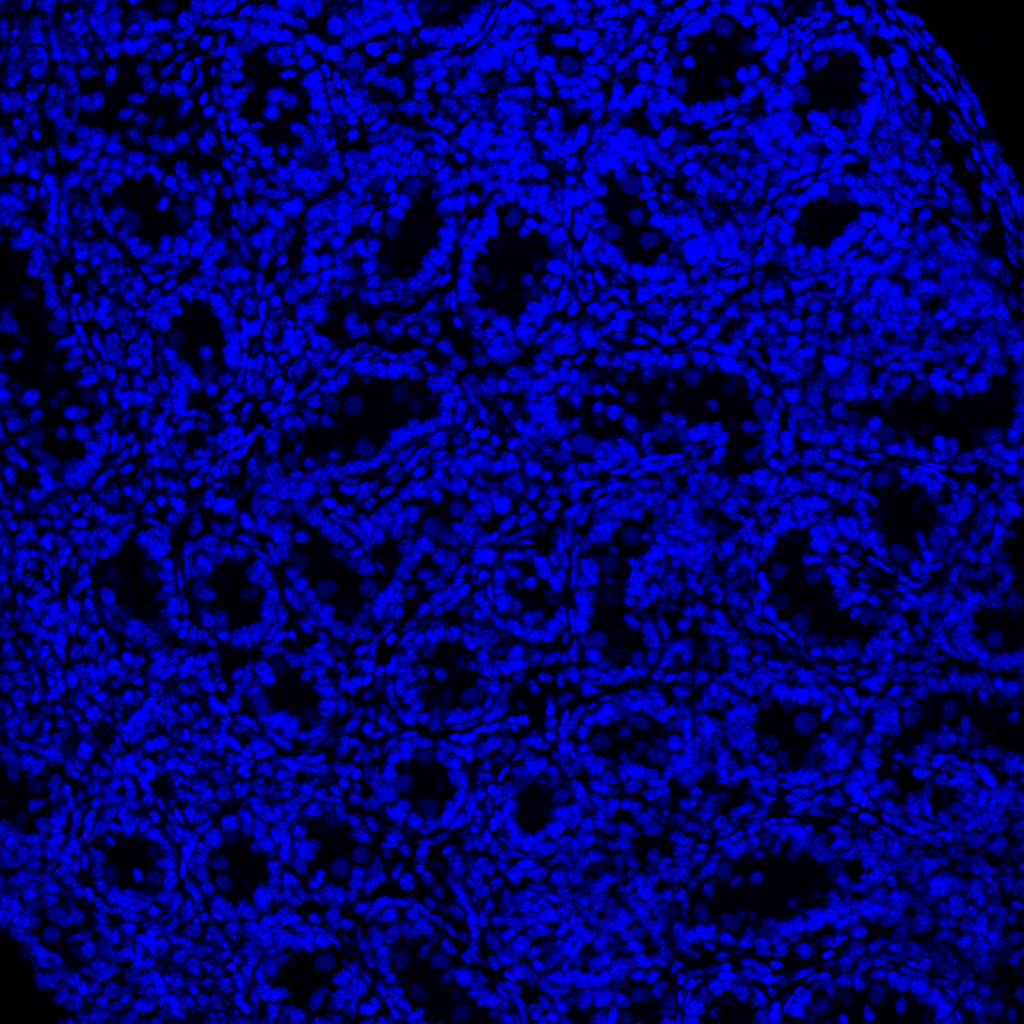

Supplement: Supplementary file 5 — Source data Fig. 2 [file 44319_2025_487_MOESM5_ESM.zip › Figure 2/2H/PD1 MVH/PD1 Brca1 vKO testis anti-MVH Hoechst.tif]

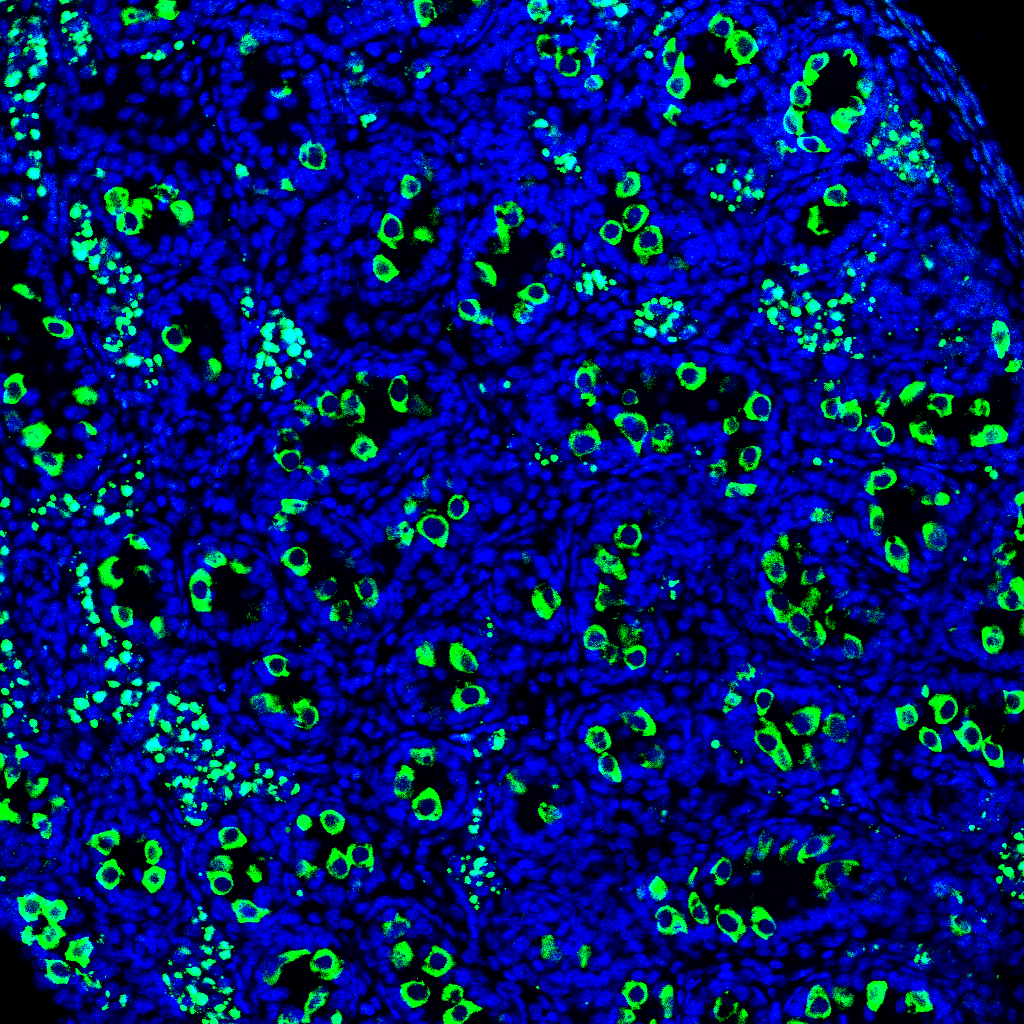

Supplement: Supplementary file 5 — Source data Fig. 2 [file 44319_2025_487_MOESM5_ESM.zip › Figure 2/2H/PD1 MVH/PD1 Brca1 vKO testis anti-MVH Hoechst_overlay.tif]

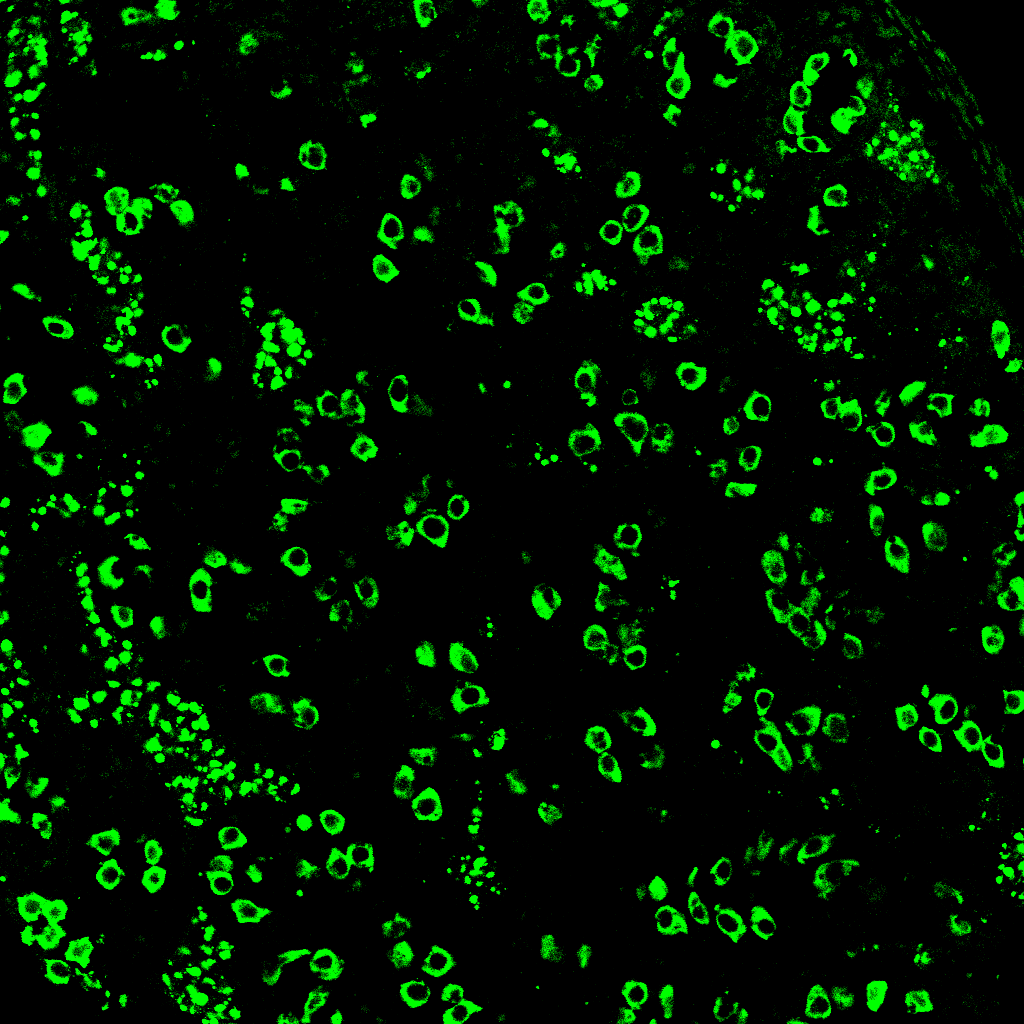

Supplement: Supplementary file 5 — Source data Fig. 2 [file 44319_2025_487_MOESM5_ESM.zip › Figure 2/2H/PD1 MVH/PD1 Brca1 vKO testis anti-MVH.tif]

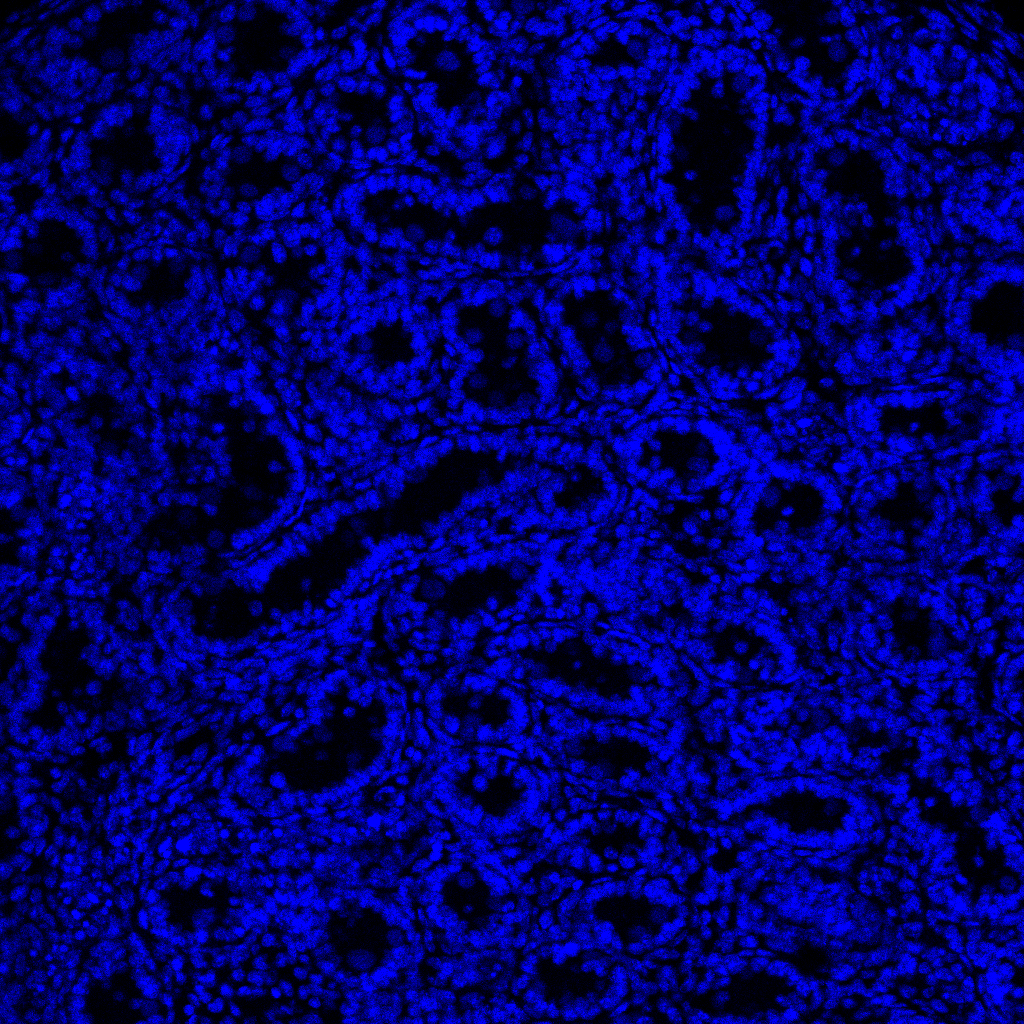

Supplement: Supplementary file 5 — Source data Fig. 2 [file 44319_2025_487_MOESM5_ESM.zip › Figure 2/2H/PD1 MVH/PD1 Control testis anti-MVH Hoechst.tif]

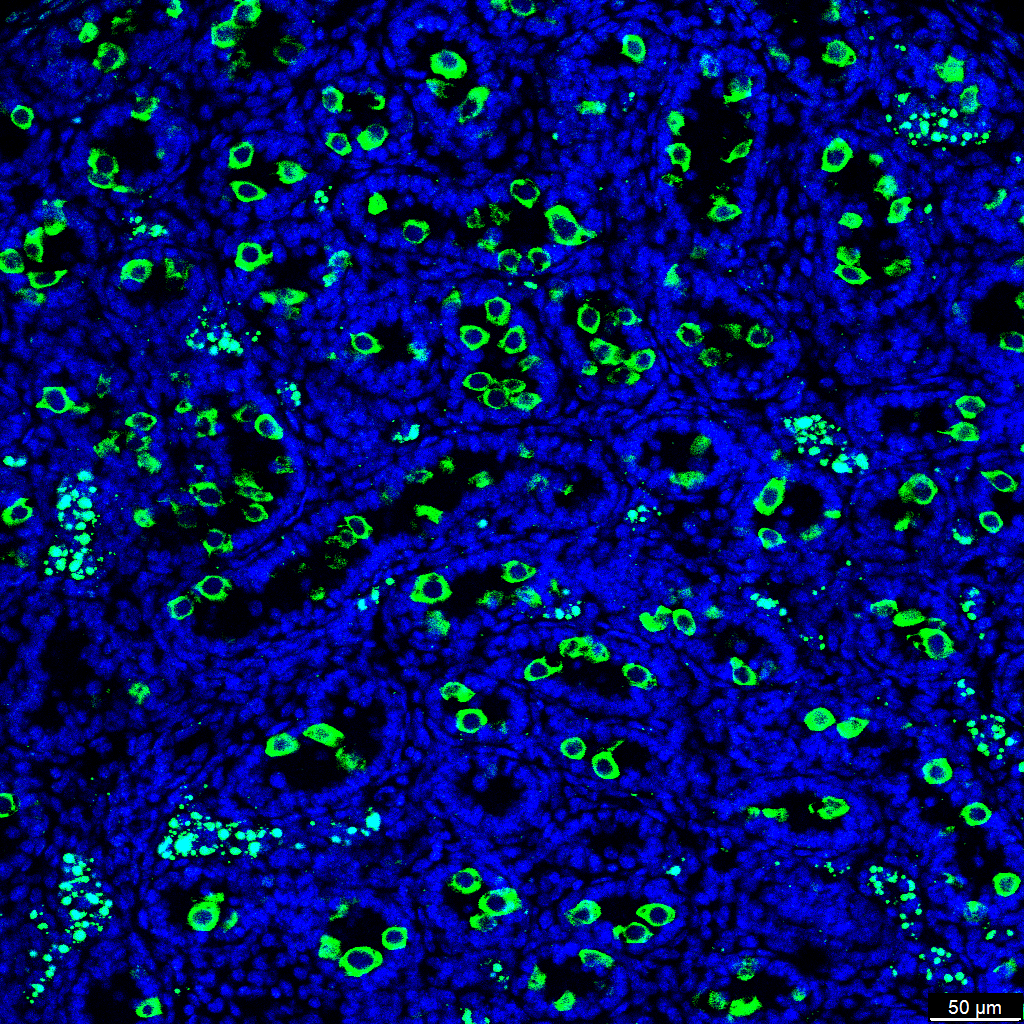

Supplement: Supplementary file 5 — Source data Fig. 2 [file 44319_2025_487_MOESM5_ESM.zip › Figure 2/2H/PD1 MVH/PD1 Control testis anti-MVH Hoechst_overlay.tif]

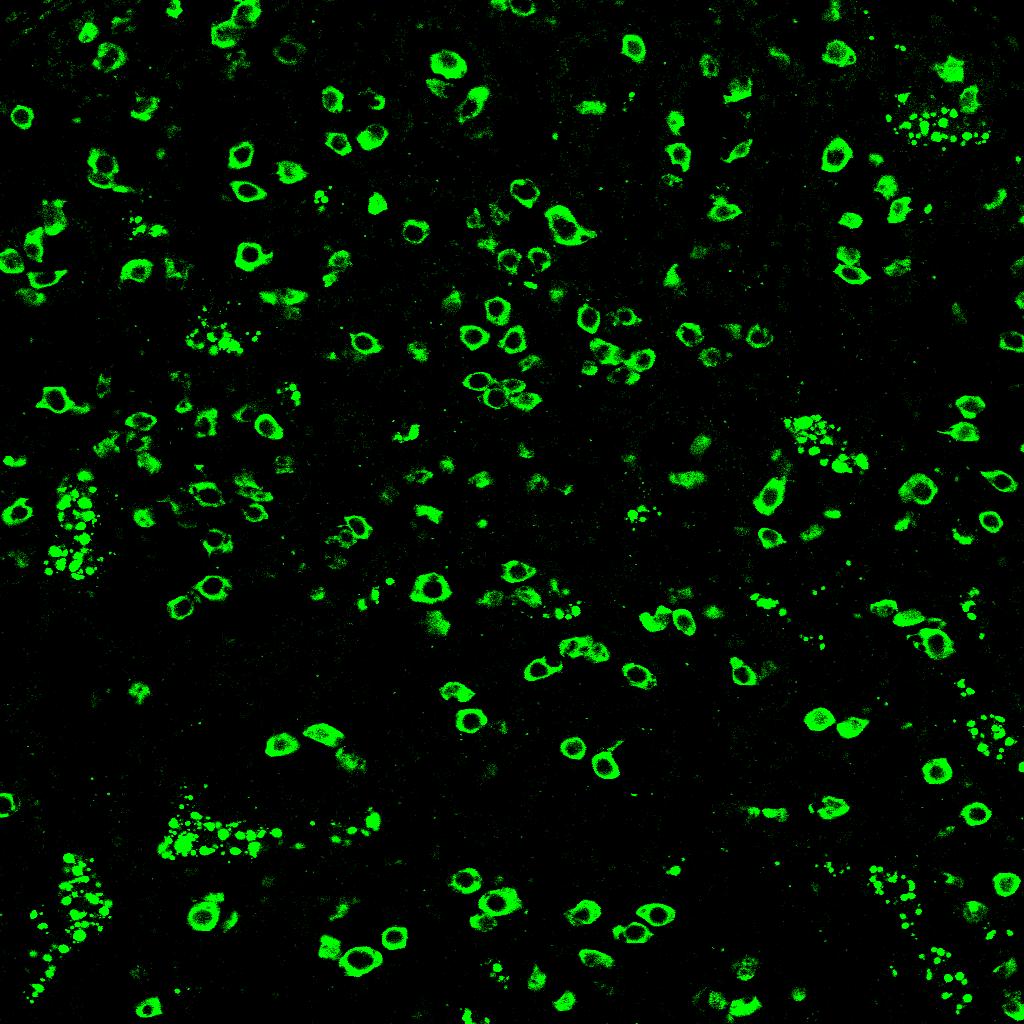

Supplement: Supplementary file 5 — Source data Fig. 2 [file 44319_2025_487_MOESM5_ESM.zip › Figure 2/2H/PD1 MVH/PD1 Control testis anti-MVH.tif]

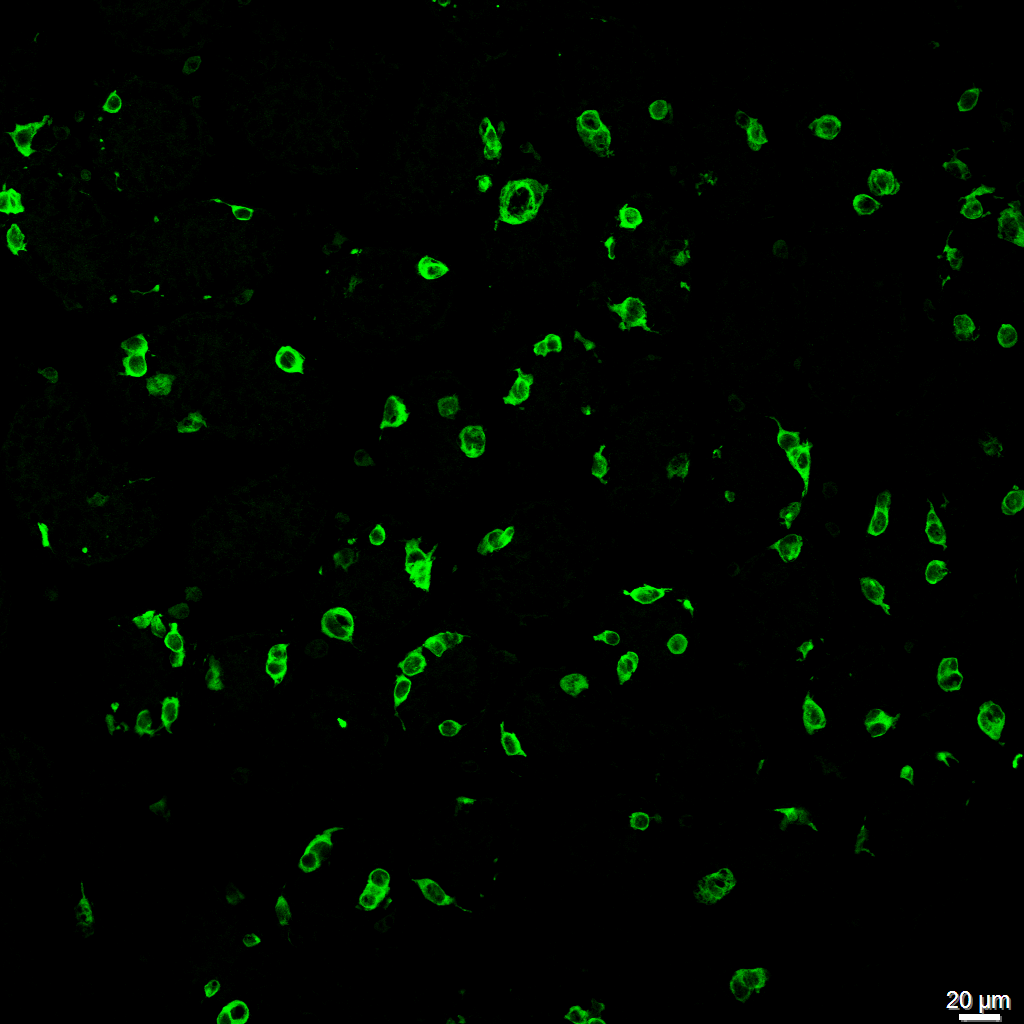

Supplement: Supplementary file 5 — Source data Fig. 2 [file 44319_2025_487_MOESM5_ESM.zip › Figure 2/2H/PD7 MVH/PD7 Brca1 vKO testis anti-MVH 20X.tif]

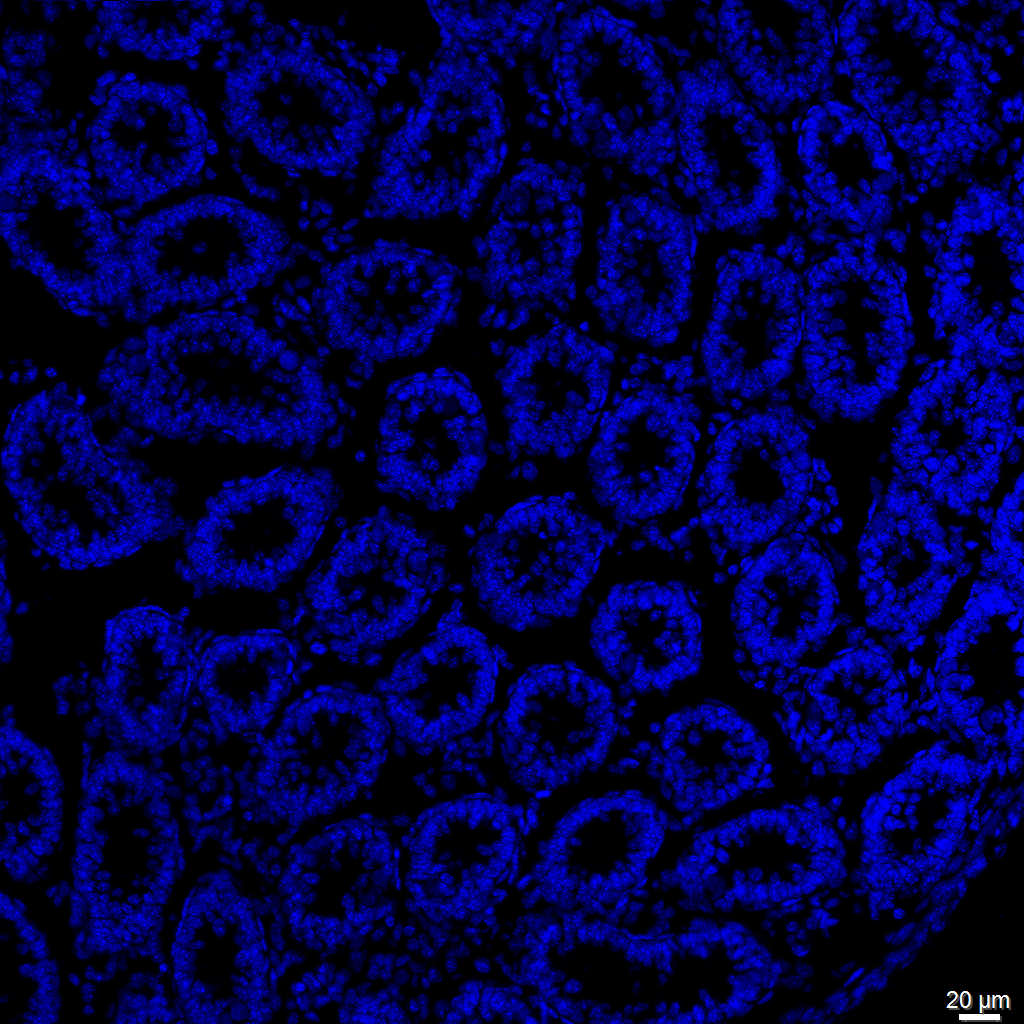

Supplement: Supplementary file 5 — Source data Fig. 2 [file 44319_2025_487_MOESM5_ESM.zip › Figure 2/2H/PD7 MVH/PD7 Brca1 vKO testis anti-MVH Hoechst 20X.tif]

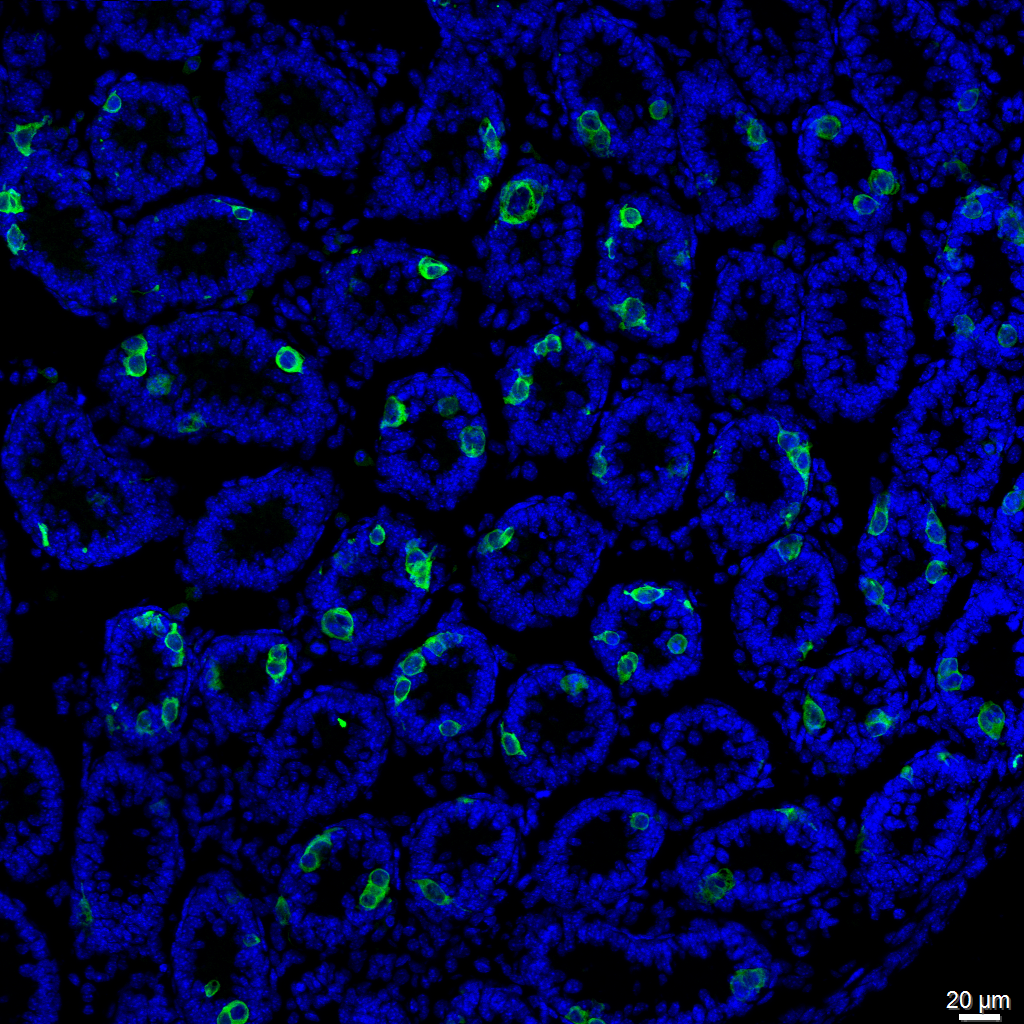

Supplement: Supplementary file 5 — Source data Fig. 2 [file 44319_2025_487_MOESM5_ESM.zip › Figure 2/2H/PD7 MVH/PD7 Brca1 vKO testis anti-MVH Hoechst overlay.tif]

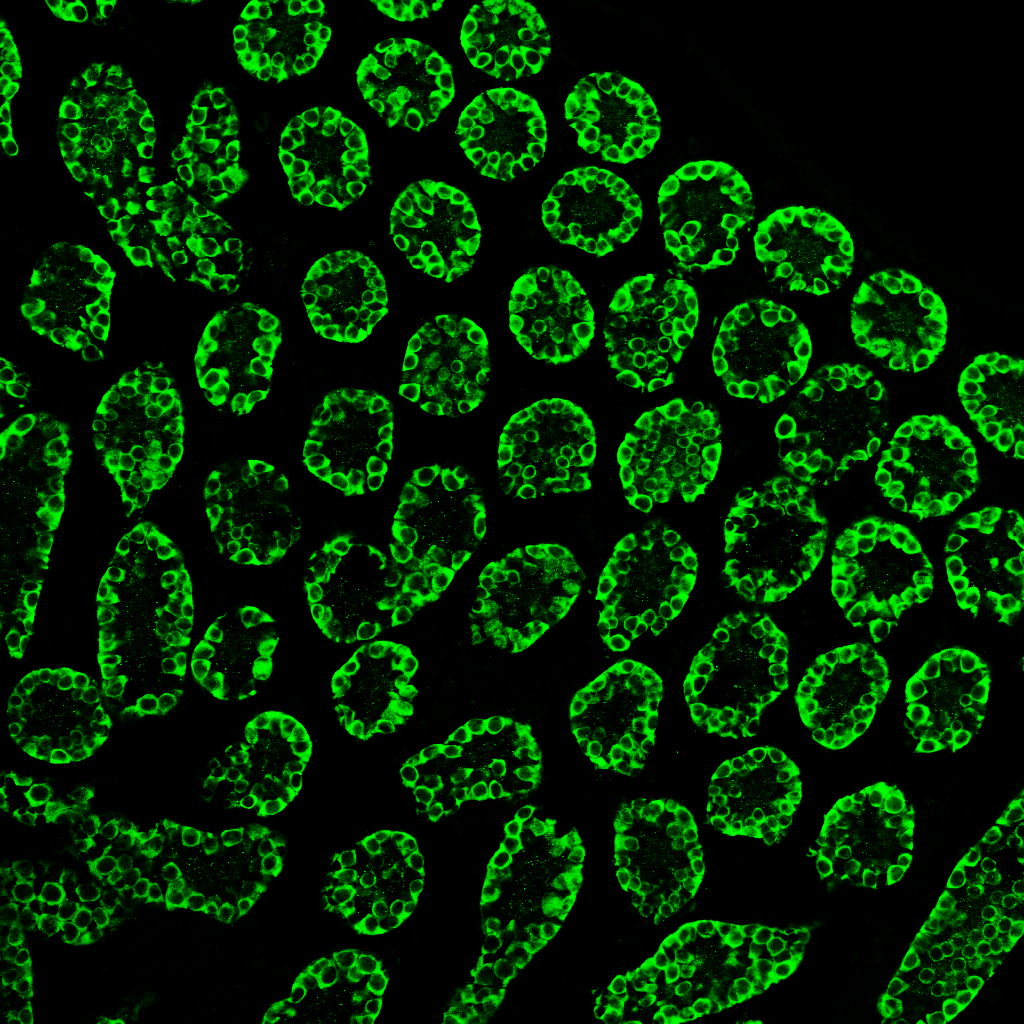

Supplement: Supplementary file 5 — Source data Fig. 2 [file 44319_2025_487_MOESM5_ESM.zip › Figure 2/2H/PD7 MVH/PD7 Control testis anti-MVH.tif]

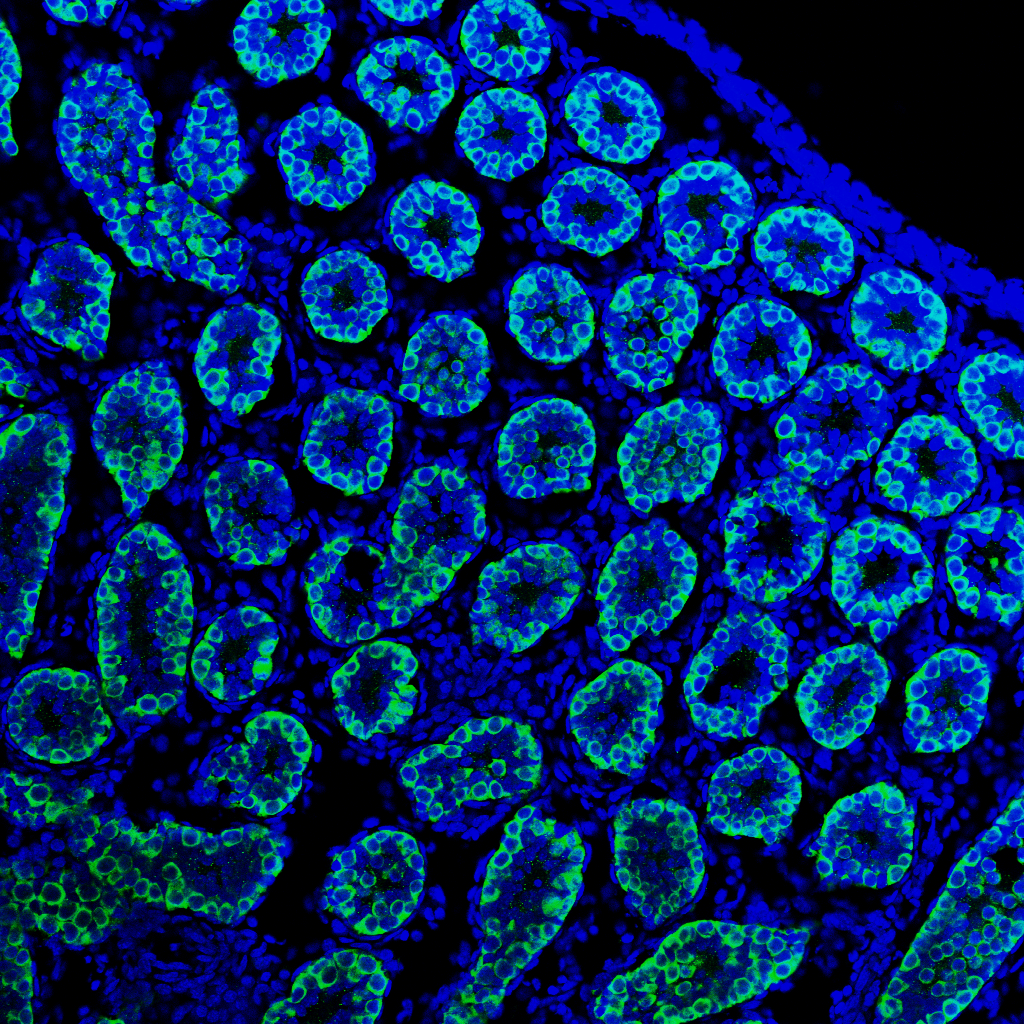

Supplement: Supplementary file 5 — Source data Fig. 2 [file 44319_2025_487_MOESM5_ESM.zip › Figure 2/2H/PD7 MVH/PD7 Control testis anti-MVH Hoechst overlay.tif]

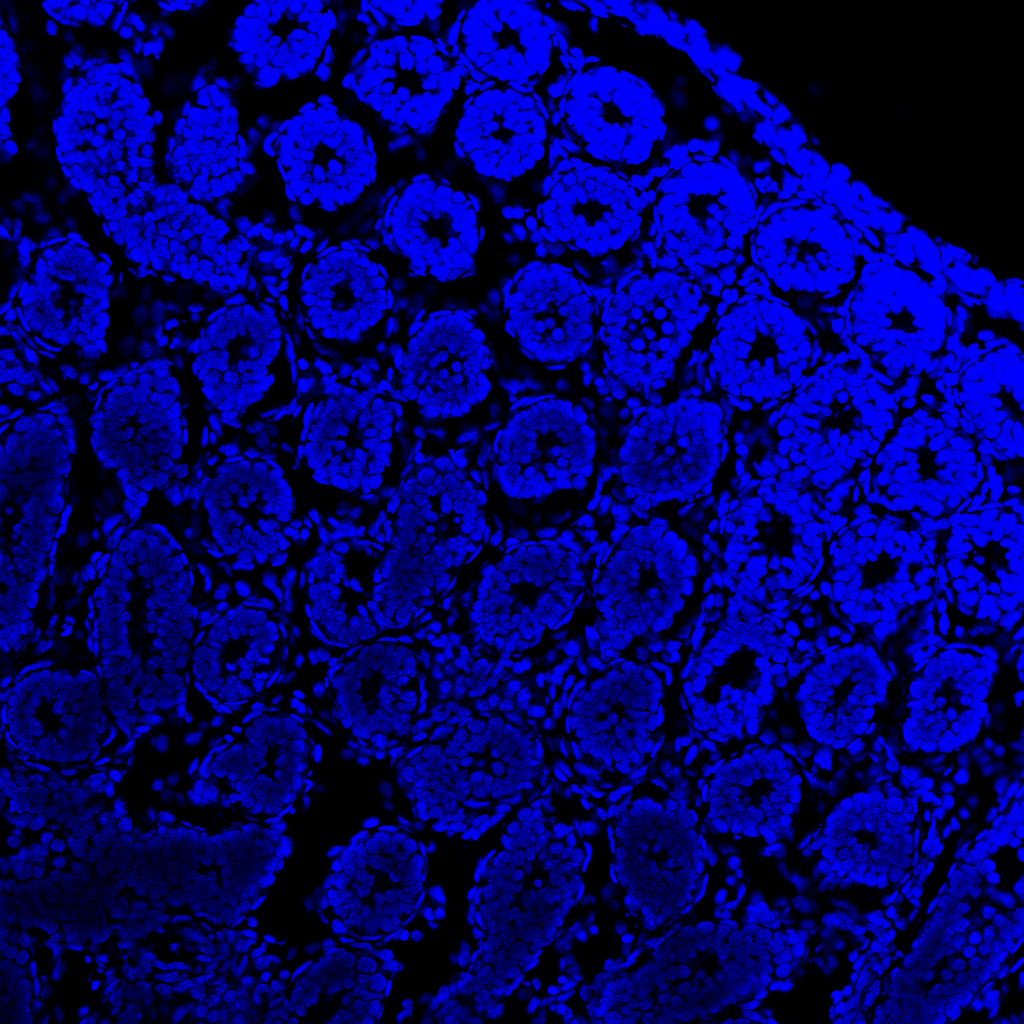

Supplement: Supplementary file 5 — Source data Fig. 2 [file 44319_2025_487_MOESM5_ESM.zip › Figure 2/2H/PD7 MVH/PD7 Control testis anti-MVH Hoechst.tif]

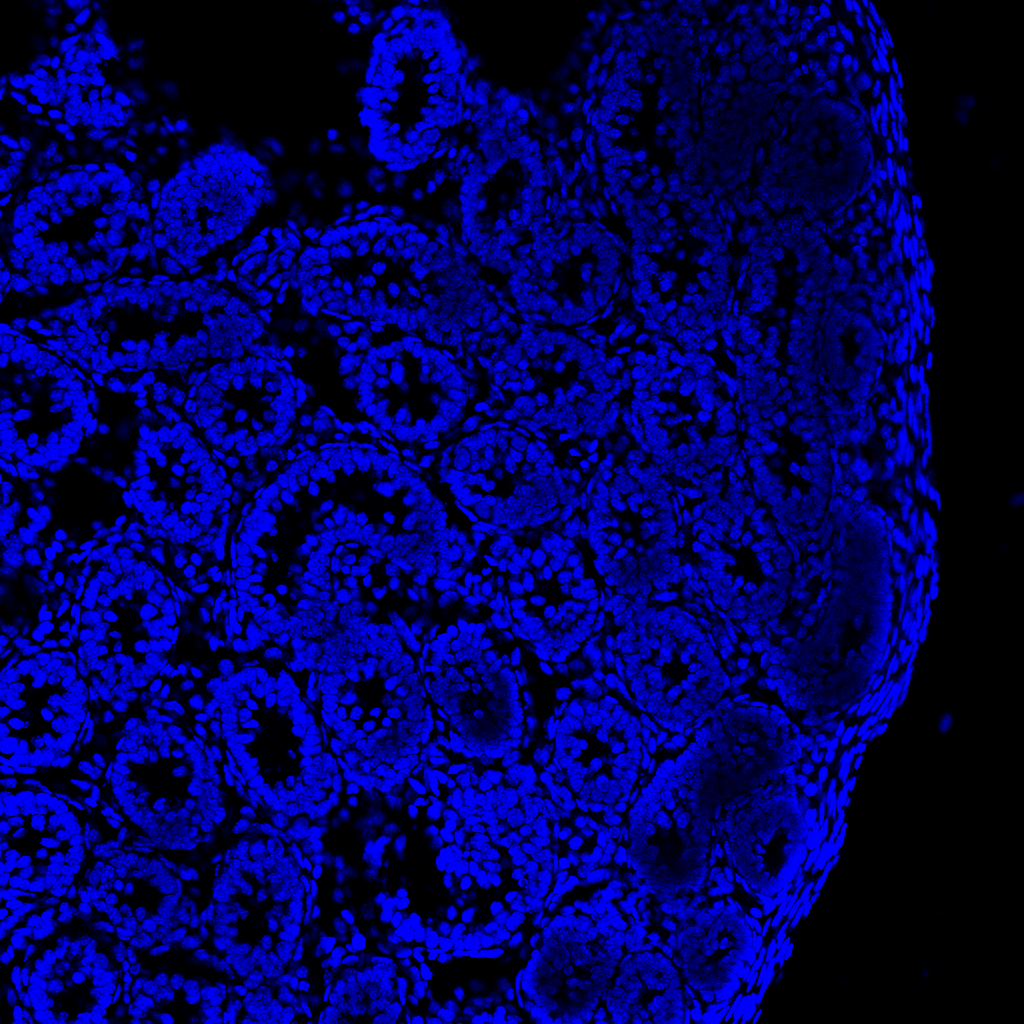

Supplement: Supplementary file 6 — Source data Fig. 3 [file 44319_2025_487_MOESM6_ESM.zip › Figure 3/3D/PD7 Brca1 vKO testis anti-MVH&PLZF Hoechst.tif]

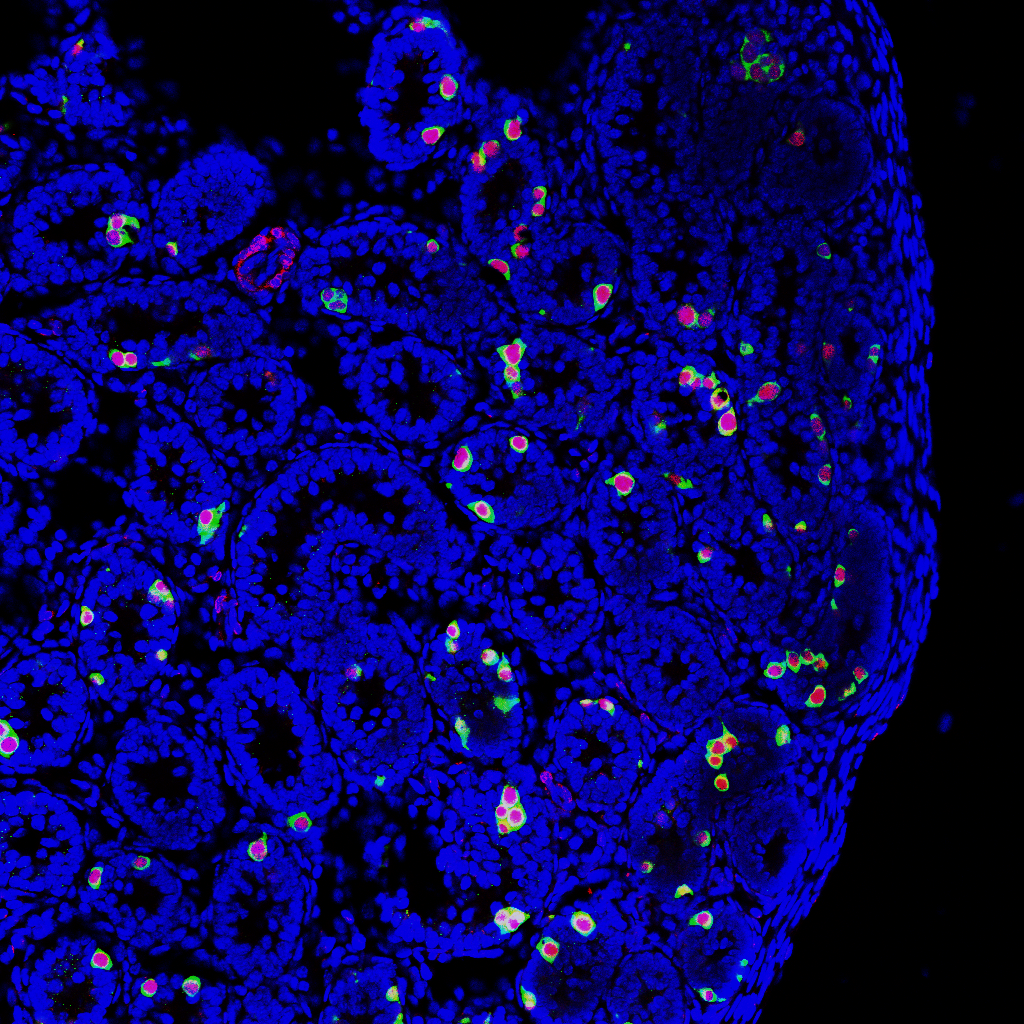

Supplement: Supplementary file 6 — Source data Fig. 3 [file 44319_2025_487_MOESM6_ESM.zip › Figure 3/3D/PD7 Brca1 vKO testis anti-MVH&PLZF Hoechst_overlay.tif]

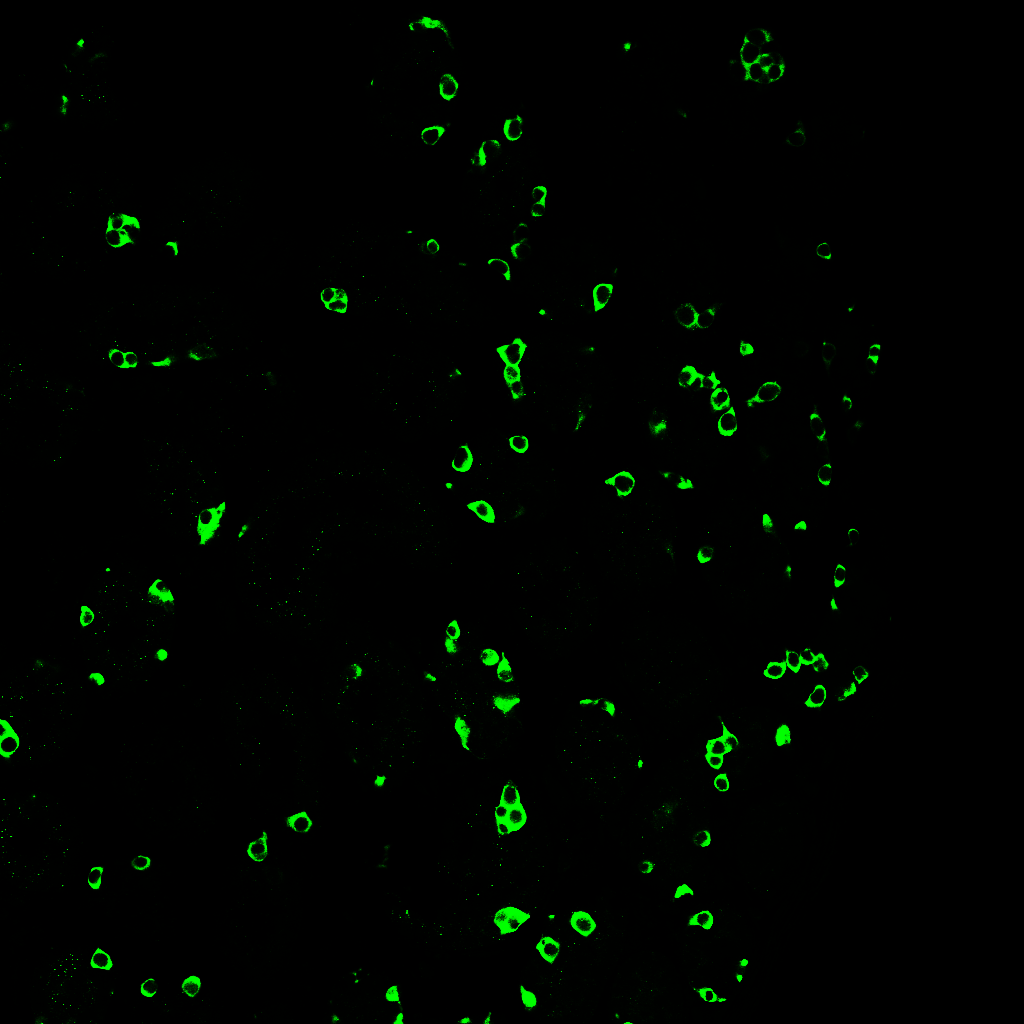

Supplement: Supplementary file 6 — Source data Fig. 3 [file 44319_2025_487_MOESM6_ESM.zip › Figure 3/3D/PD7 Brca1 vKO testis anti-MVH.tif]

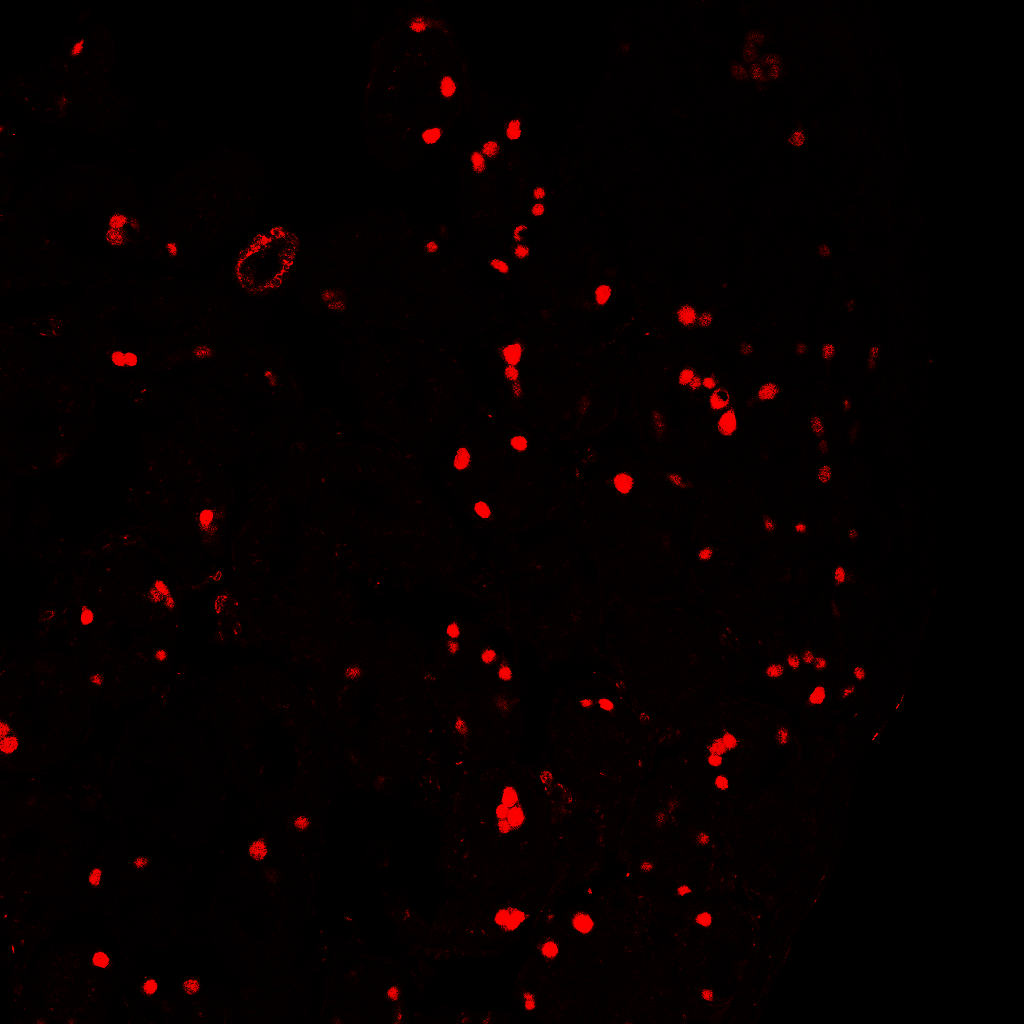

Supplement: Supplementary file 6 — Source data Fig. 3 [file 44319_2025_487_MOESM6_ESM.zip › Figure 3/3D/PD7 Brca1 vKO testis anti-PLZF.tif]

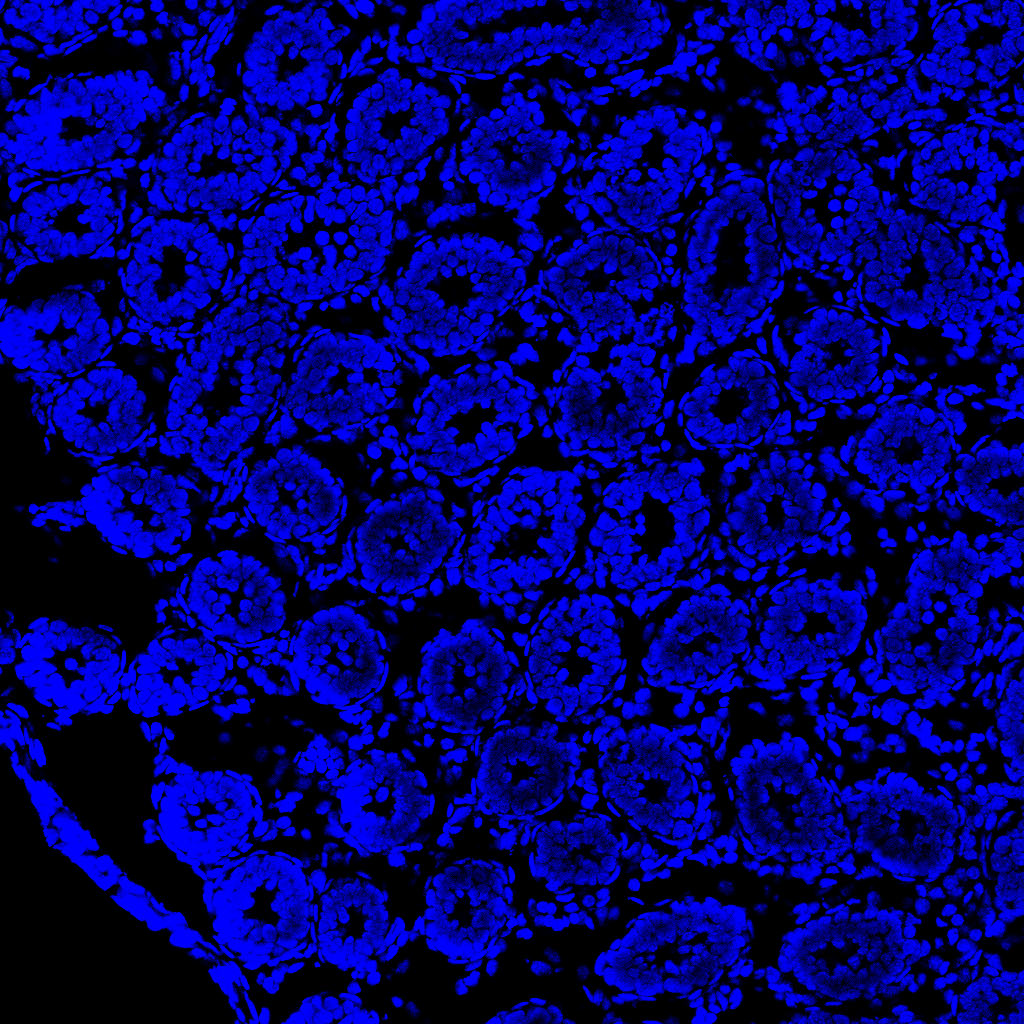

Supplement: Supplementary file 6 — Source data Fig. 3 [file 44319_2025_487_MOESM6_ESM.zip › Figure 3/3D/PD7 Control testis anti-MVH&PLZF Hoechst.tif]

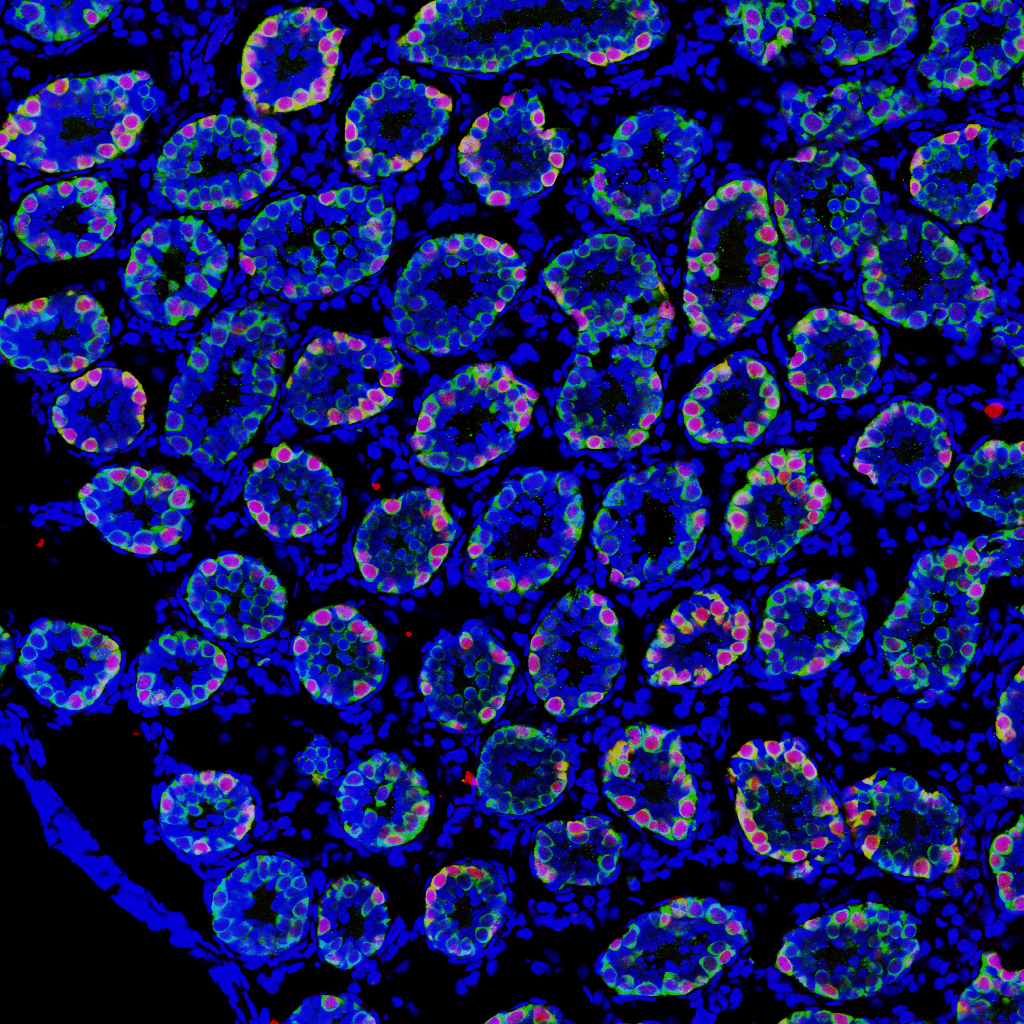

Supplement: Supplementary file 6 — Source data Fig. 3 [file 44319_2025_487_MOESM6_ESM.zip › Figure 3/3D/PD7 Control testis anti-MVH&PLZF Hoechst_overlay.tif]

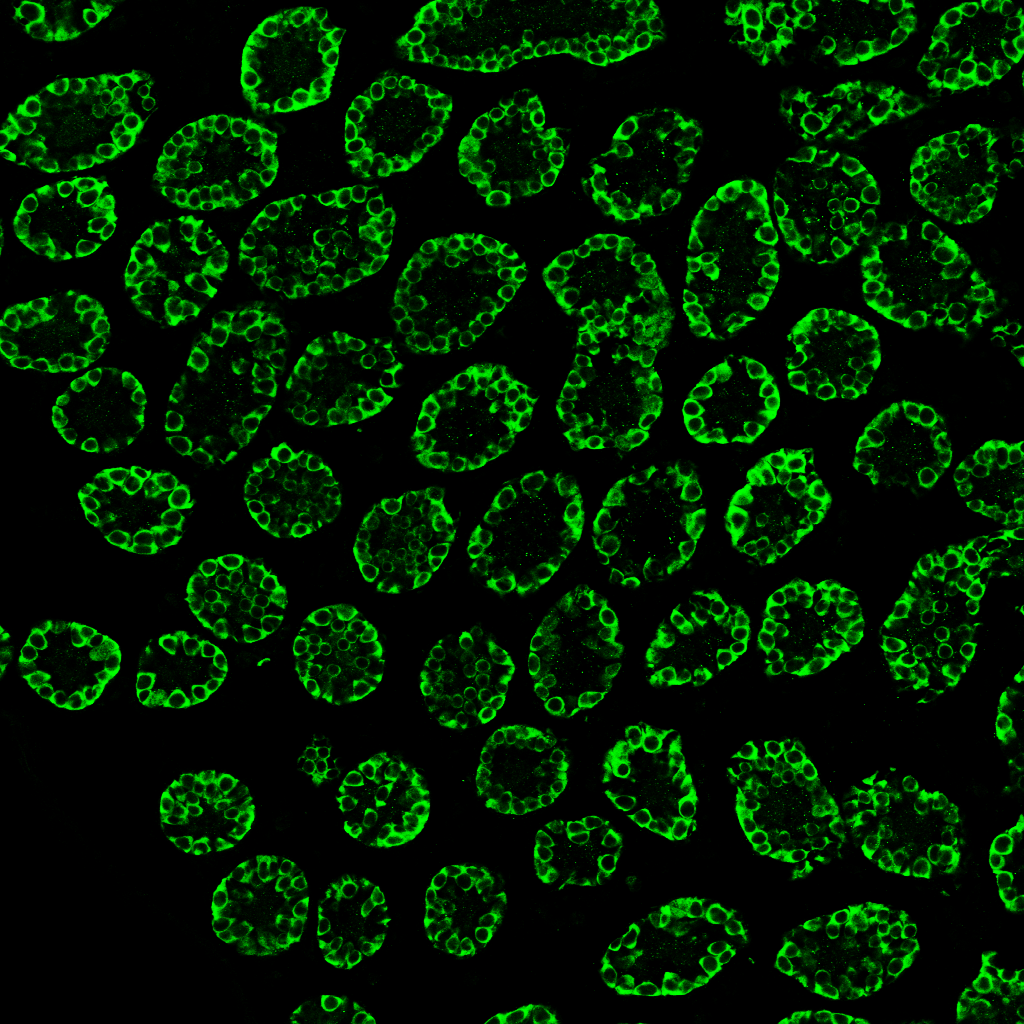

Supplement: Supplementary file 6 — Source data Fig. 3 [file 44319_2025_487_MOESM6_ESM.zip › Figure 3/3D/PD7 Control testis anti-MVH.tif]

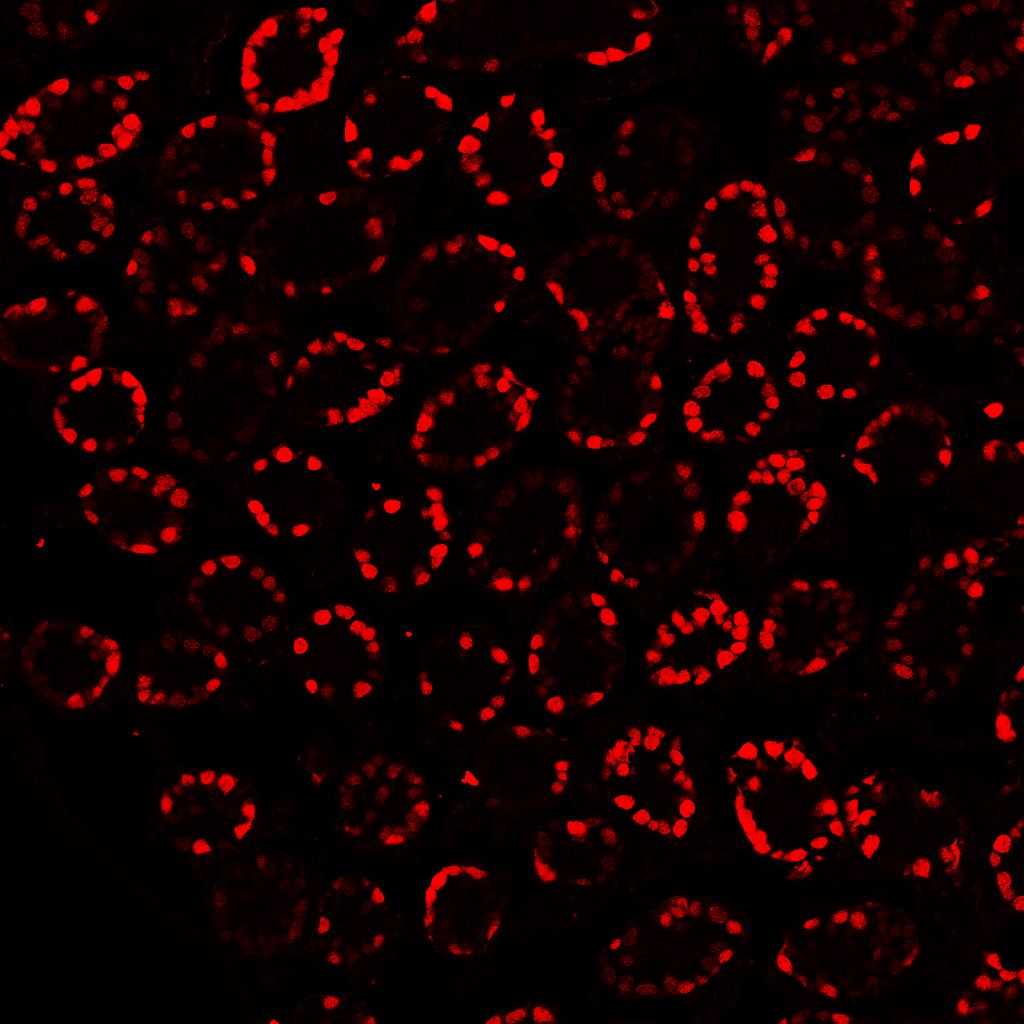

Supplement: Supplementary file 6 — Source data Fig. 3 [file 44319_2025_487_MOESM6_ESM.zip › Figure 3/3D/PD7 Control testis anti-PLZF.tif]

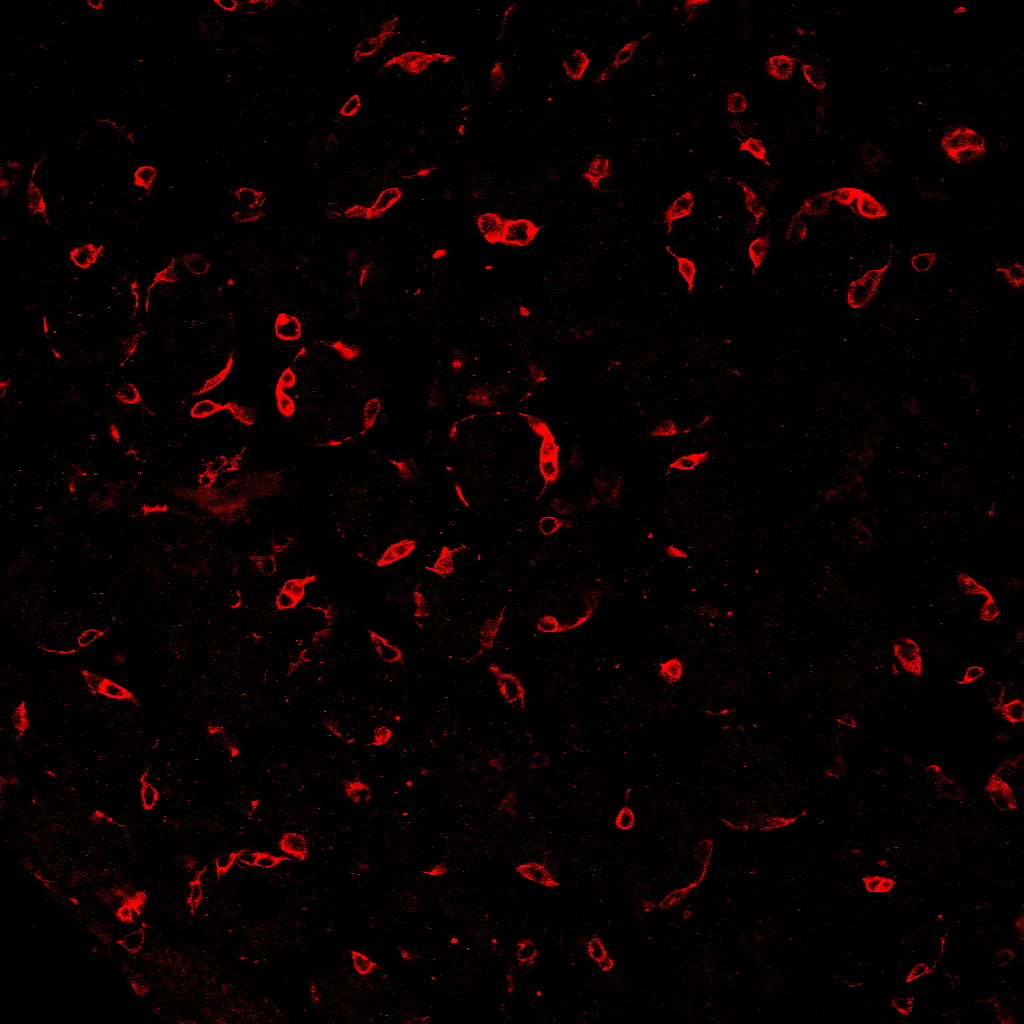

Supplement: Supplementary file 6 — Source data Fig. 3 [file 44319_2025_487_MOESM6_ESM.zip › Figure 3/3E/PD7 Brca1 Vasa-cre testis anti-PLZF&GFRa1/PD7 Brca1 vKO testis anti-GFRa1.tif]

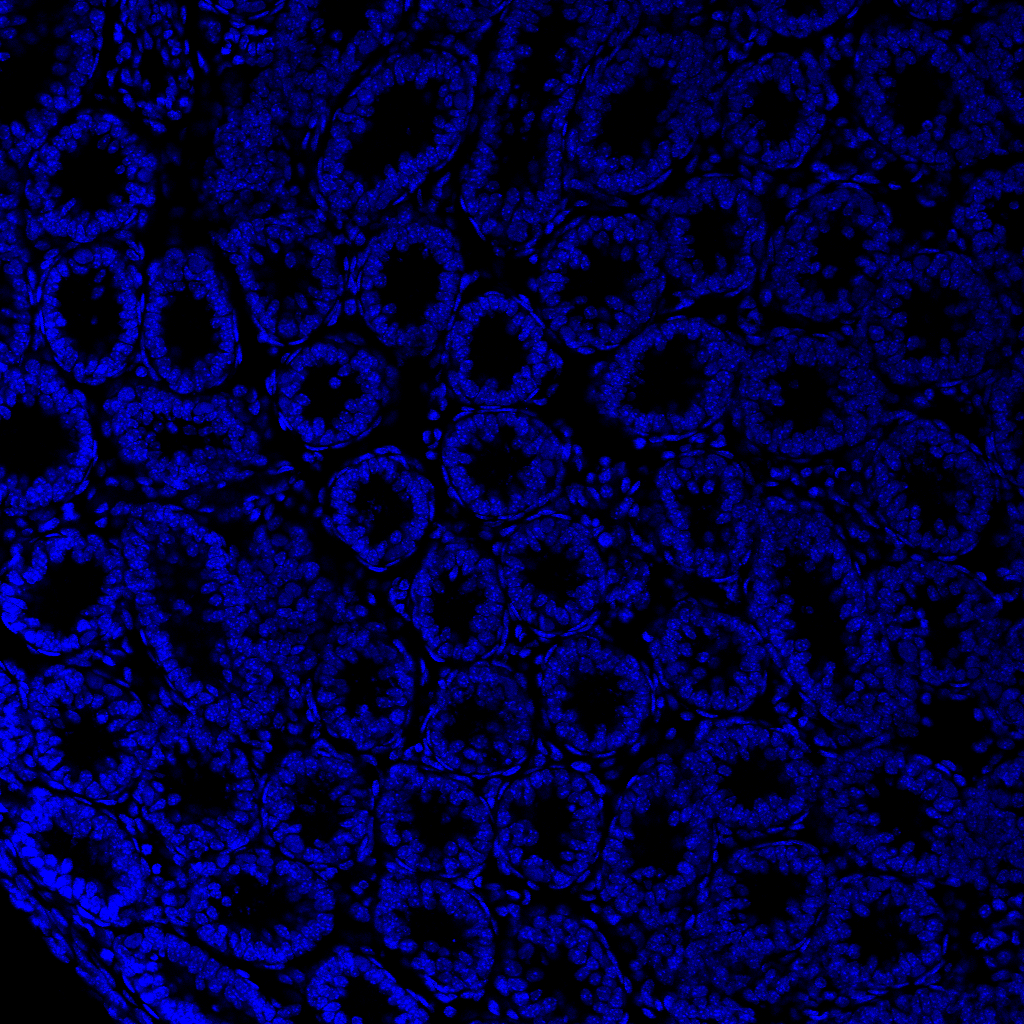

Supplement: Supplementary file 6 — Source data Fig. 3 [file 44319_2025_487_MOESM6_ESM.zip › Figure 3/3E/PD7 Brca1 Vasa-cre testis anti-PLZF&GFRa1/PD7 Brca1 vKO testis anti-PLZF&GFRa1 Hoechst.tif]

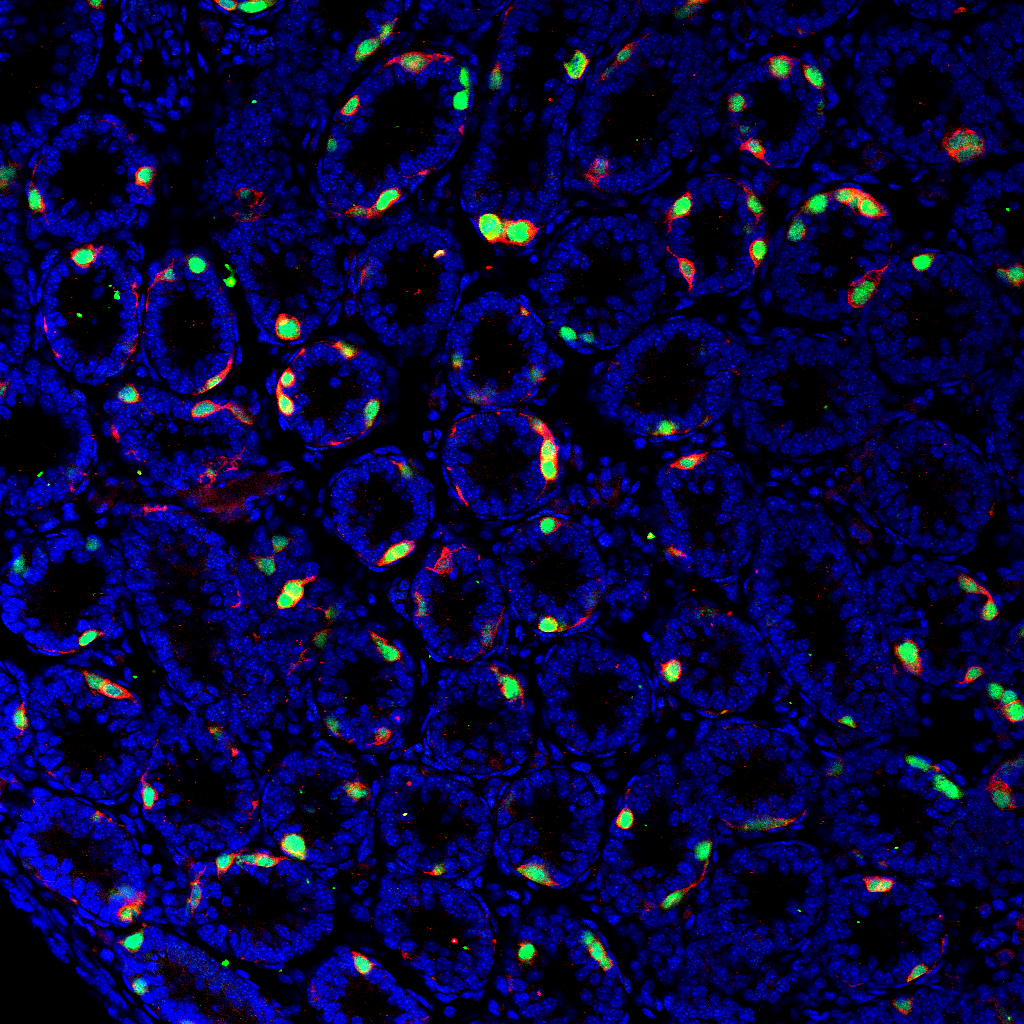

Supplement: Supplementary file 6 — Source data Fig. 3 [file 44319_2025_487_MOESM6_ESM.zip › Figure 3/3E/PD7 Brca1 Vasa-cre testis anti-PLZF&GFRa1/PD7 Brca1 vKO testis anti-PLZF&GFRa1 Hoechst_overlay.tif]

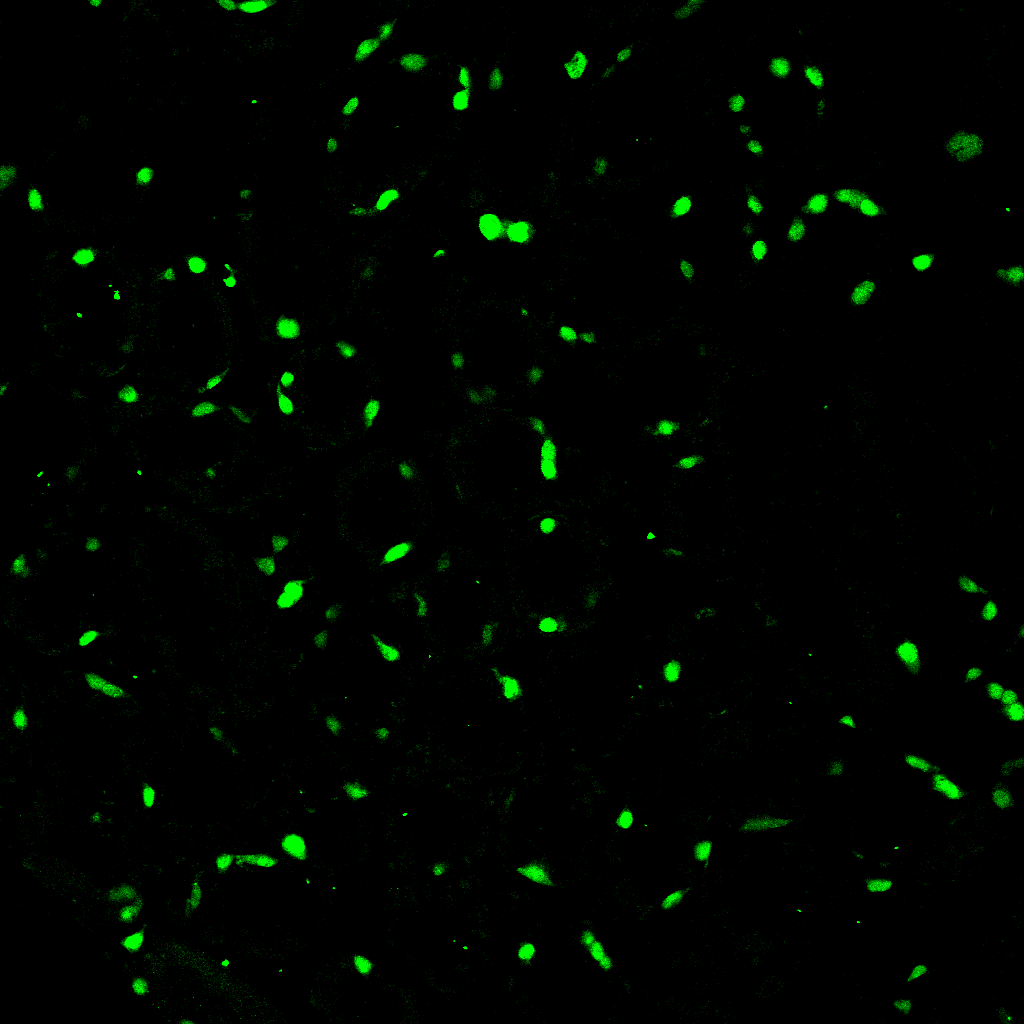

Supplement: Supplementary file 6 — Source data Fig. 3 [file 44319_2025_487_MOESM6_ESM.zip › Figure 3/3E/PD7 Brca1 Vasa-cre testis anti-PLZF&GFRa1/PD7 Brca1 vKO testis anti-PLZF.tif]

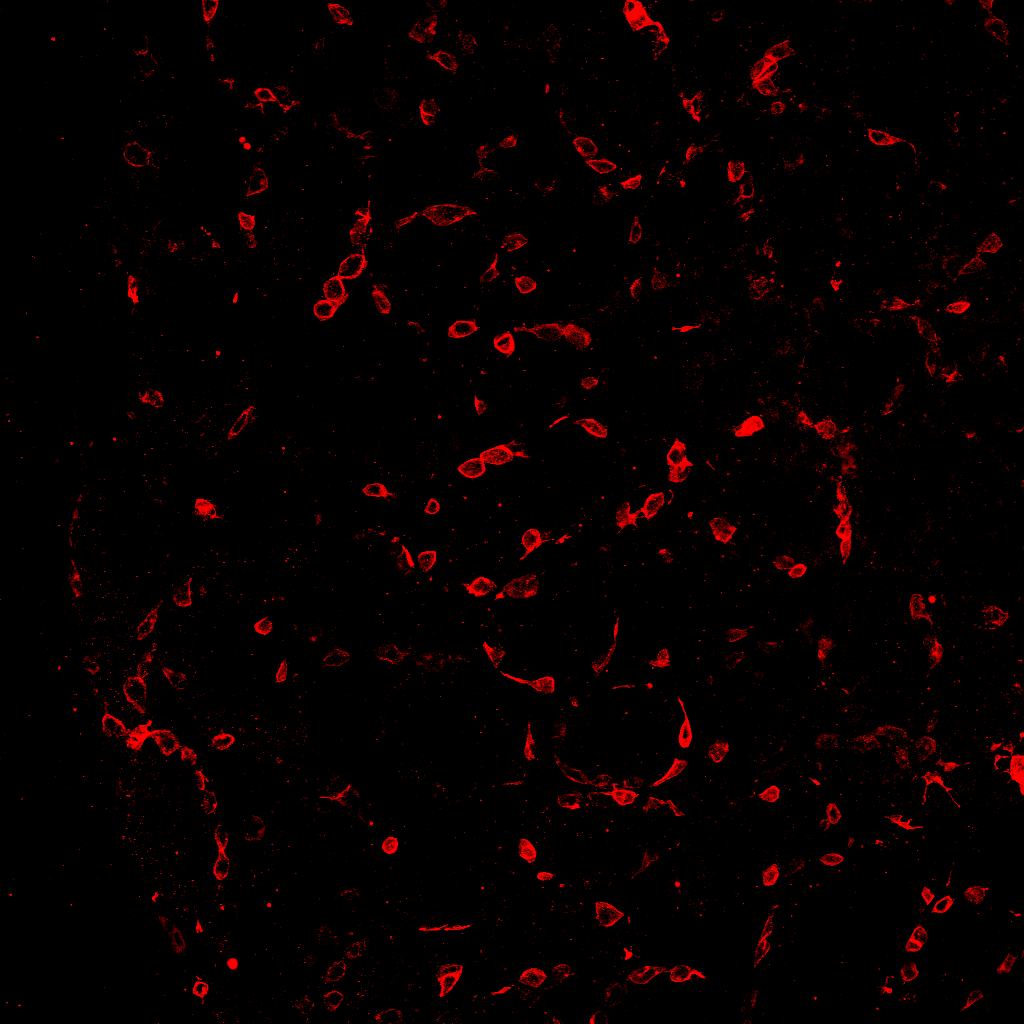

Supplement: Supplementary file 6 — Source data Fig. 3 [file 44319_2025_487_MOESM6_ESM.zip › Figure 3/3E/PD7 Brca1 Vasa-cre testis anti-PLZF&GFRa1/PD7 Control testis anti-GFRa1.tif]

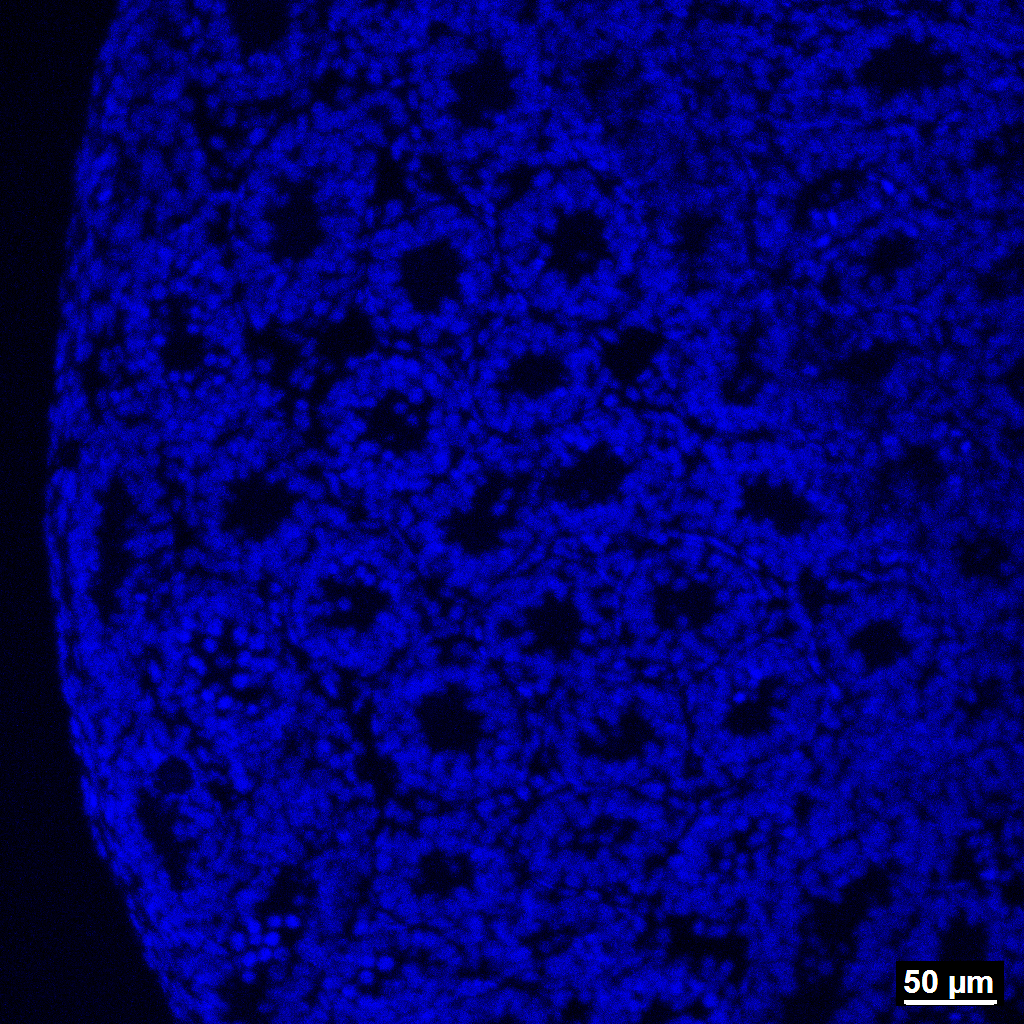

Supplement: Supplementary file 6 — Source data Fig. 3 [file 44319_2025_487_MOESM6_ESM.zip › Figure 3/3E/PD7 Brca1 Vasa-cre testis anti-PLZF&GFRa1/PD7 Control testis anti-PLZF&GFRa1 Hoechst.tif]

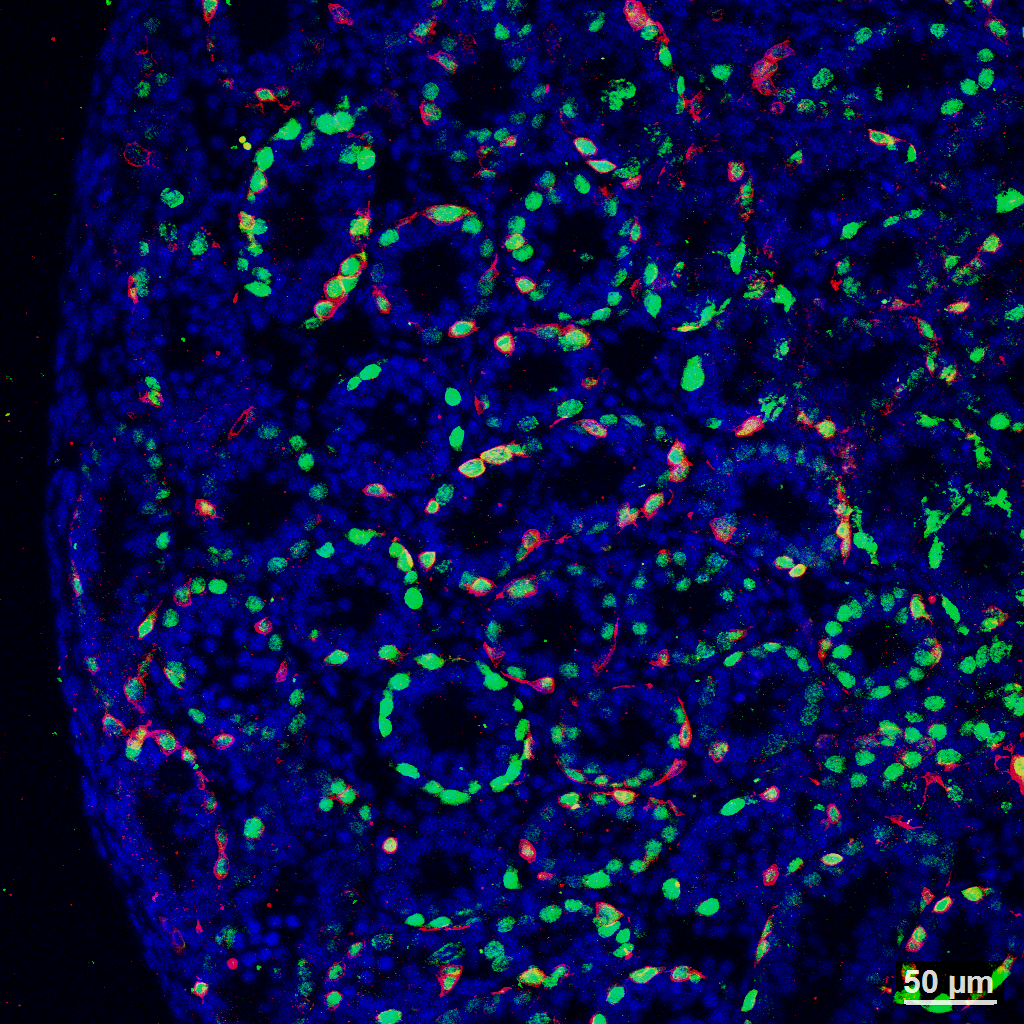

Supplement: Supplementary file 6 — Source data Fig. 3 [file 44319_2025_487_MOESM6_ESM.zip › Figure 3/3E/PD7 Brca1 Vasa-cre testis anti-PLZF&GFRa1/PD7 Control testis anti-PLZF&GFRa1 Hoechst_overlay.tif]

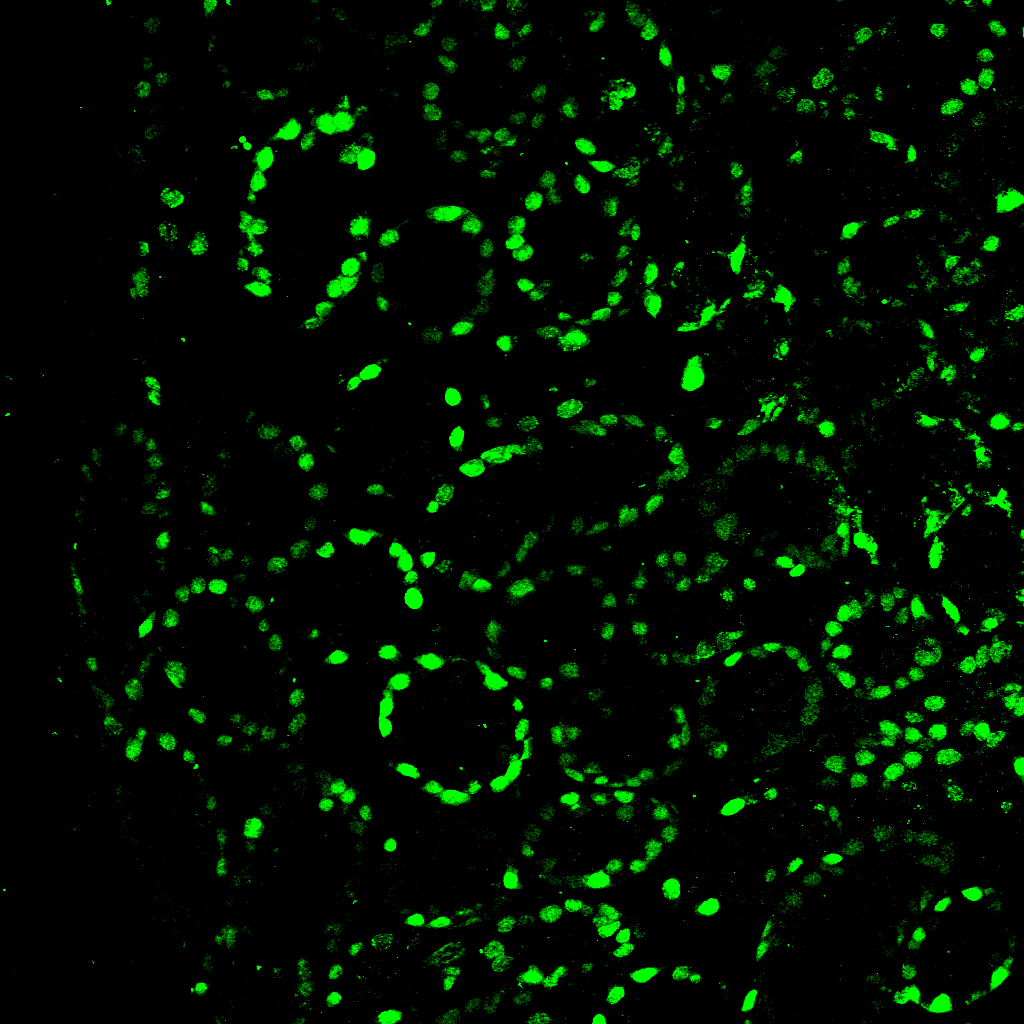

Supplement: Supplementary file 6 — Source data Fig. 3 [file 44319_2025_487_MOESM6_ESM.zip › Figure 3/3E/PD7 Brca1 Vasa-cre testis anti-PLZF&GFRa1/PD7 Control testis anti-PLZF.tif]

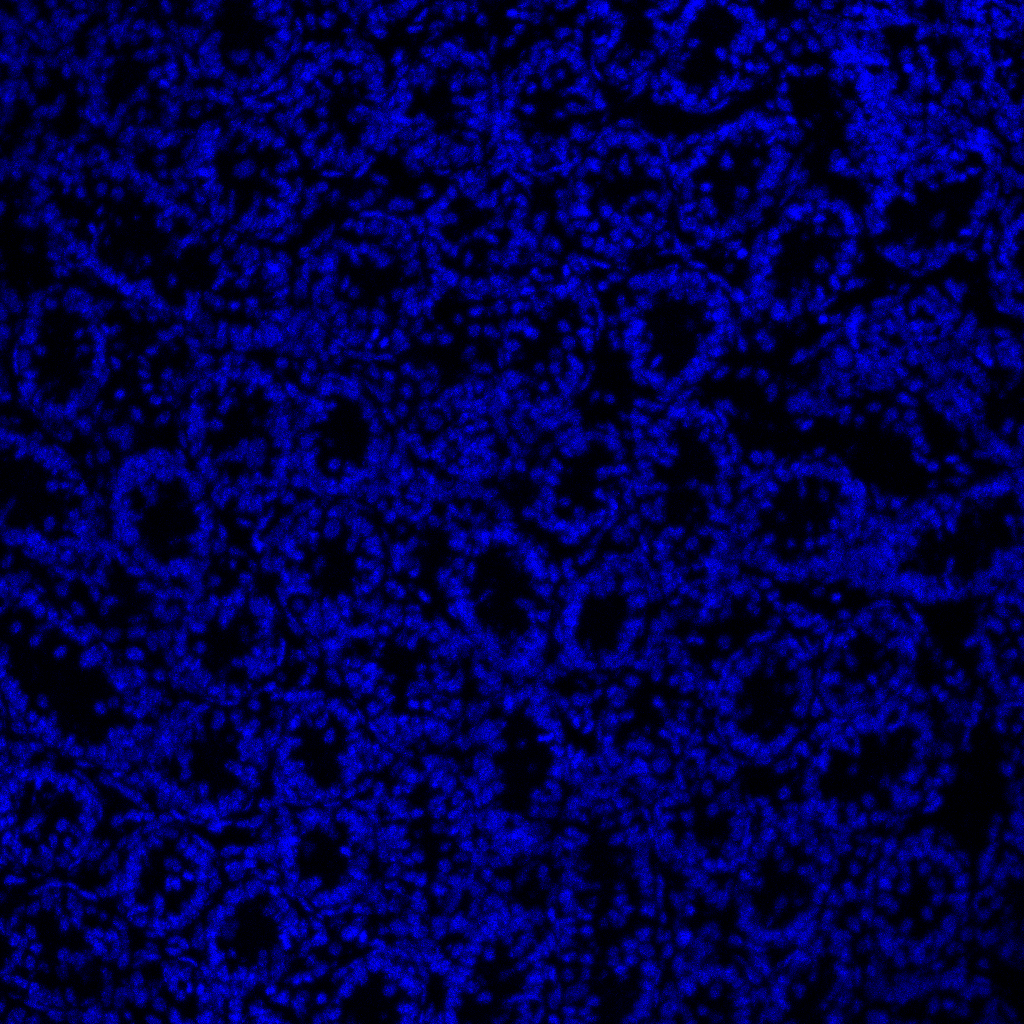

Supplement: Supplementary file 6 — Source data Fig. 3 [file 44319_2025_487_MOESM6_ESM.zip › Figure 3/3E/PD7 Brca1 Vasa-cre testis anti-PLZF&SOX3/PD7 Brca1 vKO testis anti-PLZF&SOX3 Hoechst.tif]

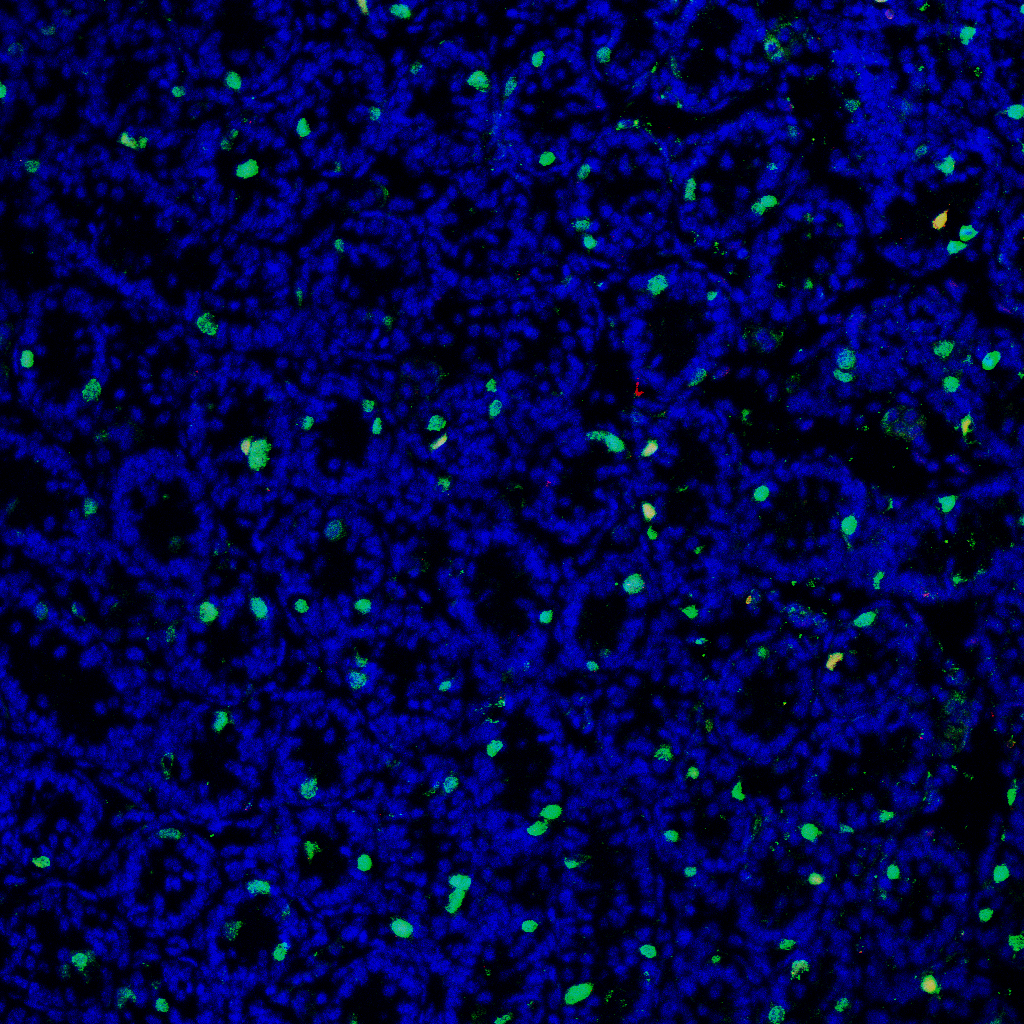

Supplement: Supplementary file 6 — Source data Fig. 3 [file 44319_2025_487_MOESM6_ESM.zip › Figure 3/3E/PD7 Brca1 Vasa-cre testis anti-PLZF&SOX3/PD7 Brca1 vKO testis anti-PLZF&SOX3 Hoechst_overlay.tif]

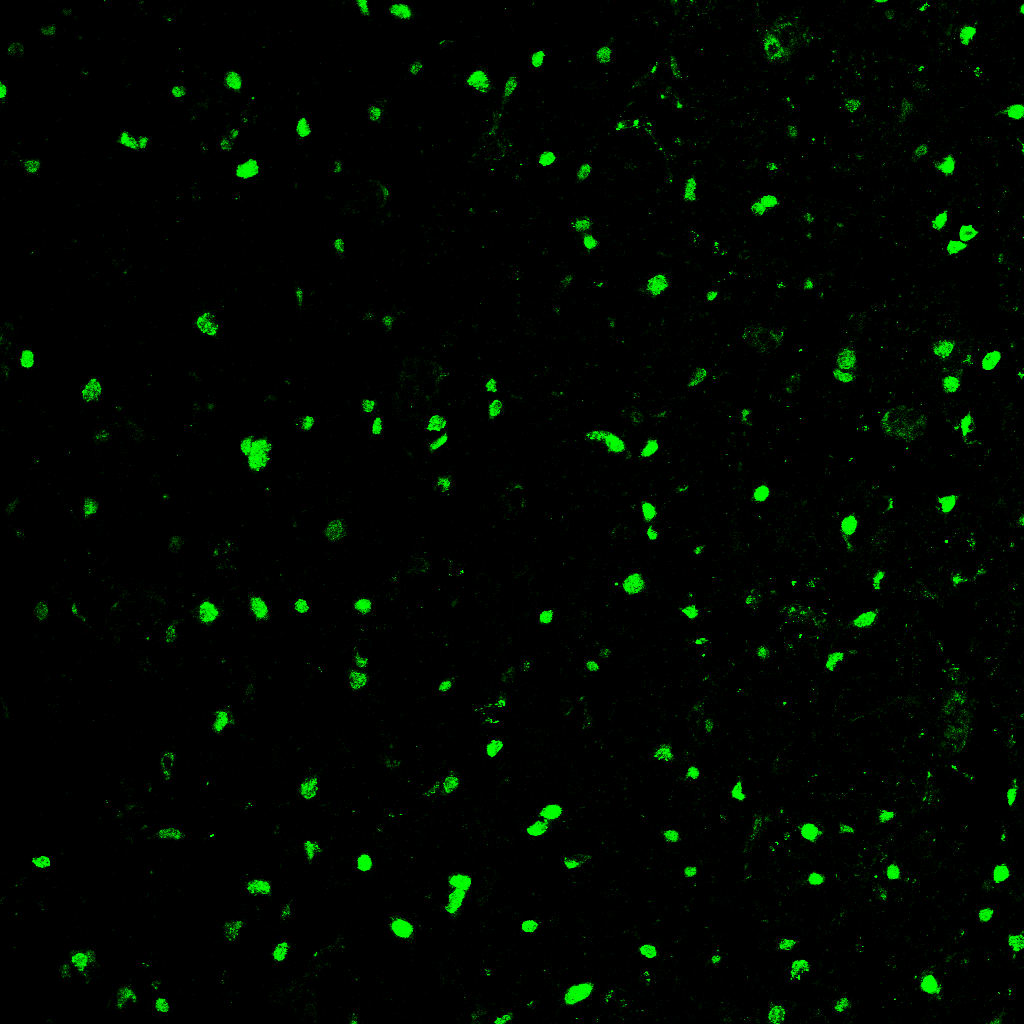

Supplement: Supplementary file 6 — Source data Fig. 3 [file 44319_2025_487_MOESM6_ESM.zip › Figure 3/3E/PD7 Brca1 Vasa-cre testis anti-PLZF&SOX3/PD7 Brca1 vKO testis anti-PLZF.tif]

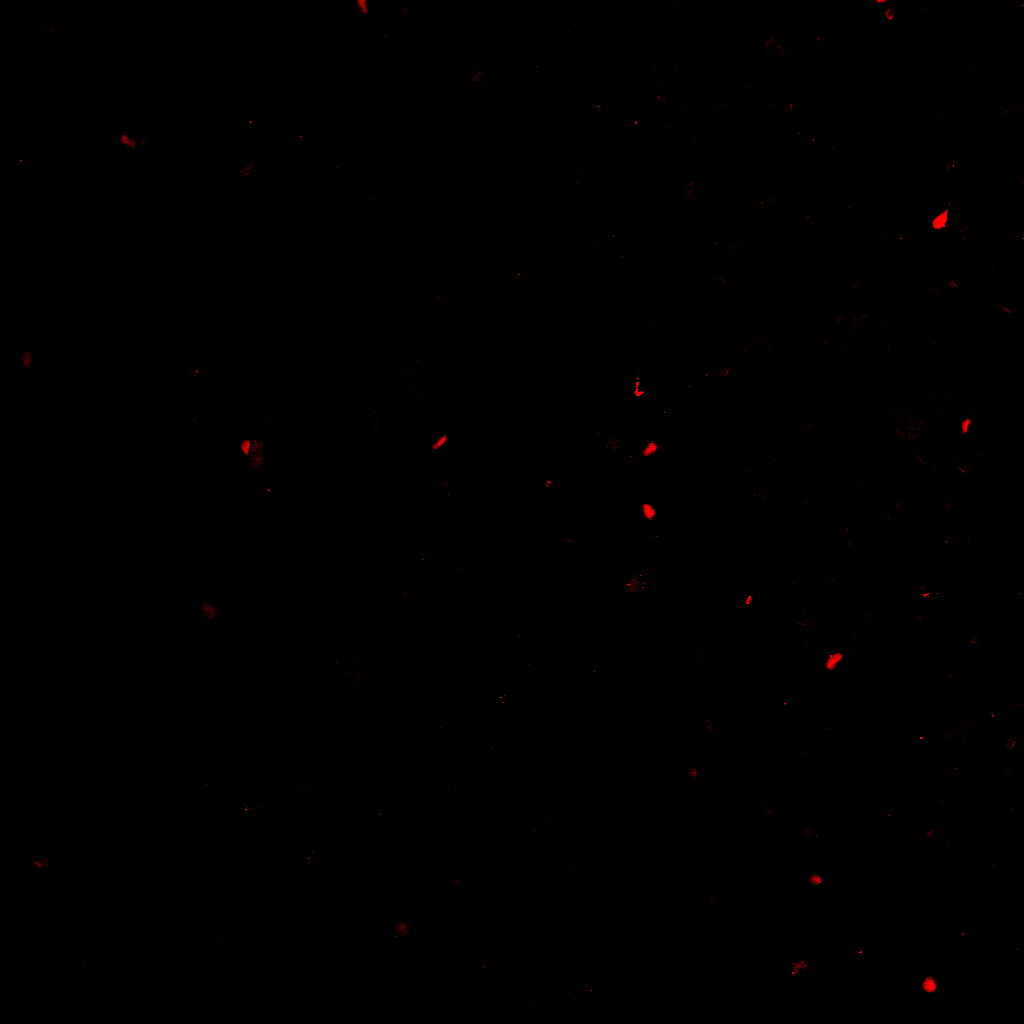

Supplement: Supplementary file 6 — Source data Fig. 3 [file 44319_2025_487_MOESM6_ESM.zip › Figure 3/3E/PD7 Brca1 Vasa-cre testis anti-PLZF&SOX3/PD7 Brca1 vKO testis anti-SOX3.tif]

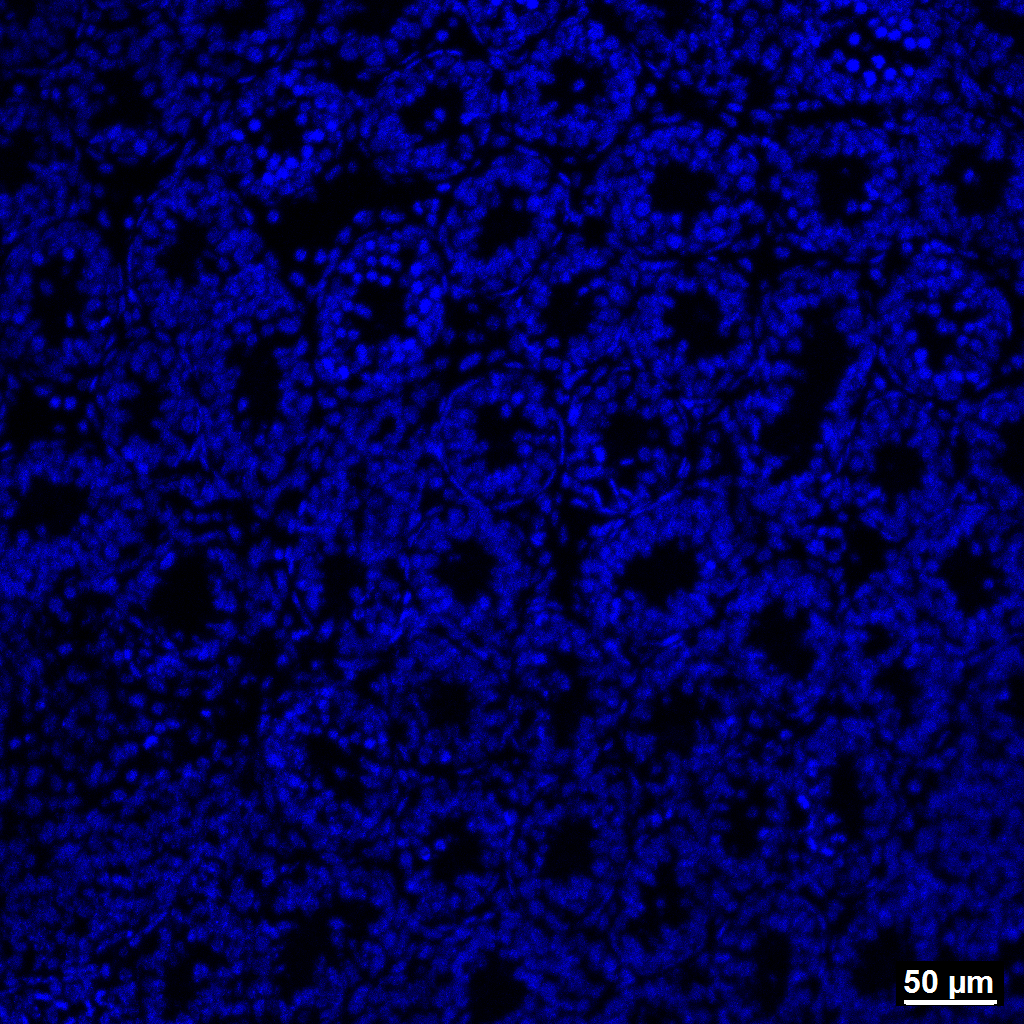

Supplement: Supplementary file 6 — Source data Fig. 3 [file 44319_2025_487_MOESM6_ESM.zip › Figure 3/3E/PD7 Brca1 Vasa-cre testis anti-PLZF&SOX3/PD7 Control testis anti-PLZF&SOX3 Hoechst.tif]

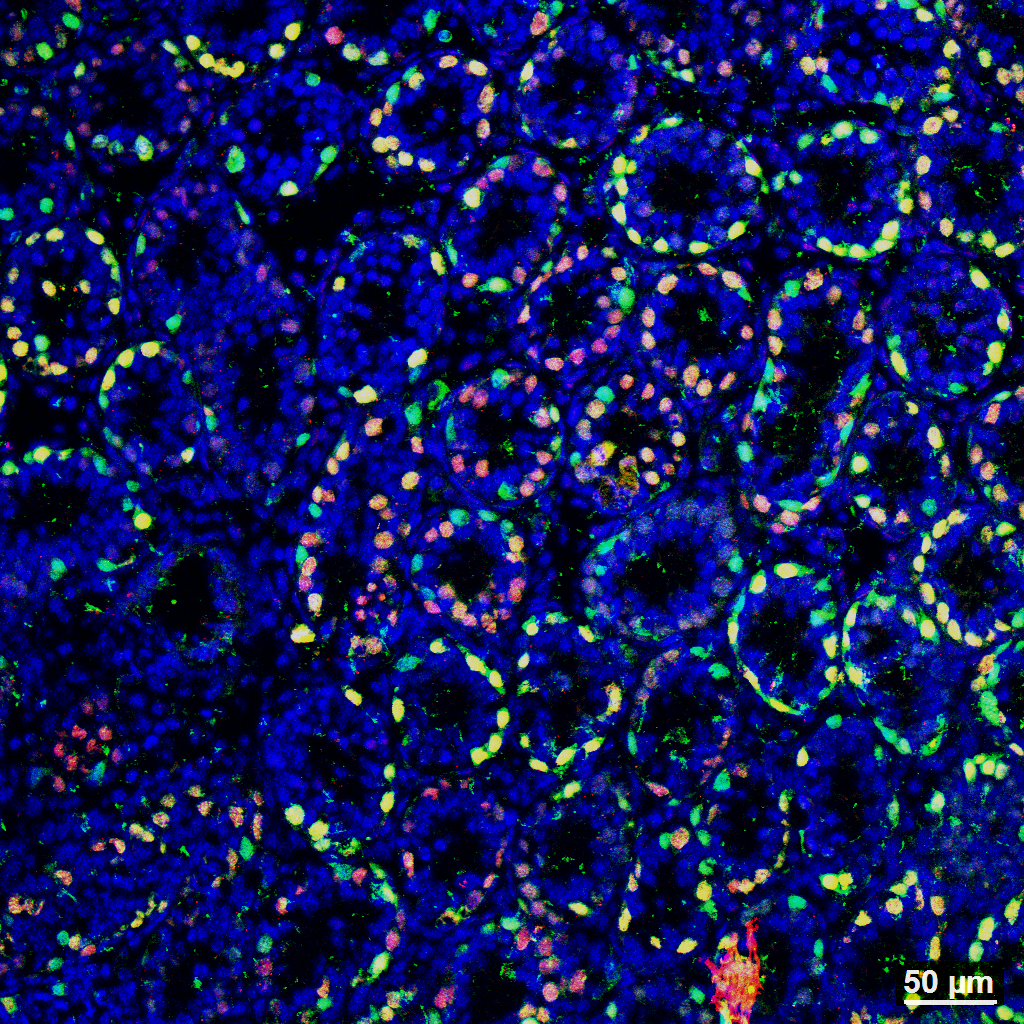

Supplement: Supplementary file 6 — Source data Fig. 3 [file 44319_2025_487_MOESM6_ESM.zip › Figure 3/3E/PD7 Brca1 Vasa-cre testis anti-PLZF&SOX3/PD7 Control testis anti-PLZF&SOX3 Hoechst_overlay.tif]

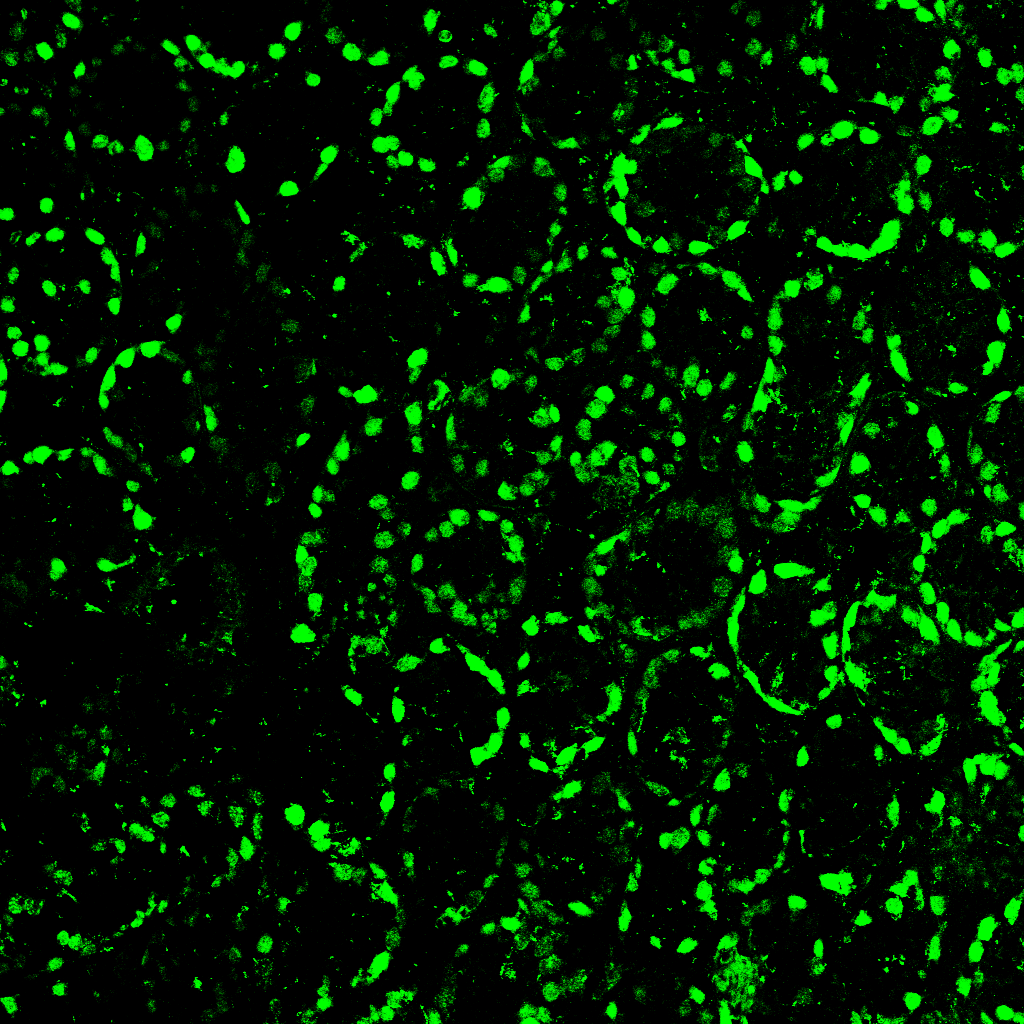

Supplement: Supplementary file 6 — Source data Fig. 3 [file 44319_2025_487_MOESM6_ESM.zip › Figure 3/3E/PD7 Brca1 Vasa-cre testis anti-PLZF&SOX3/PD7 Control testis anti-PLZF.tif]

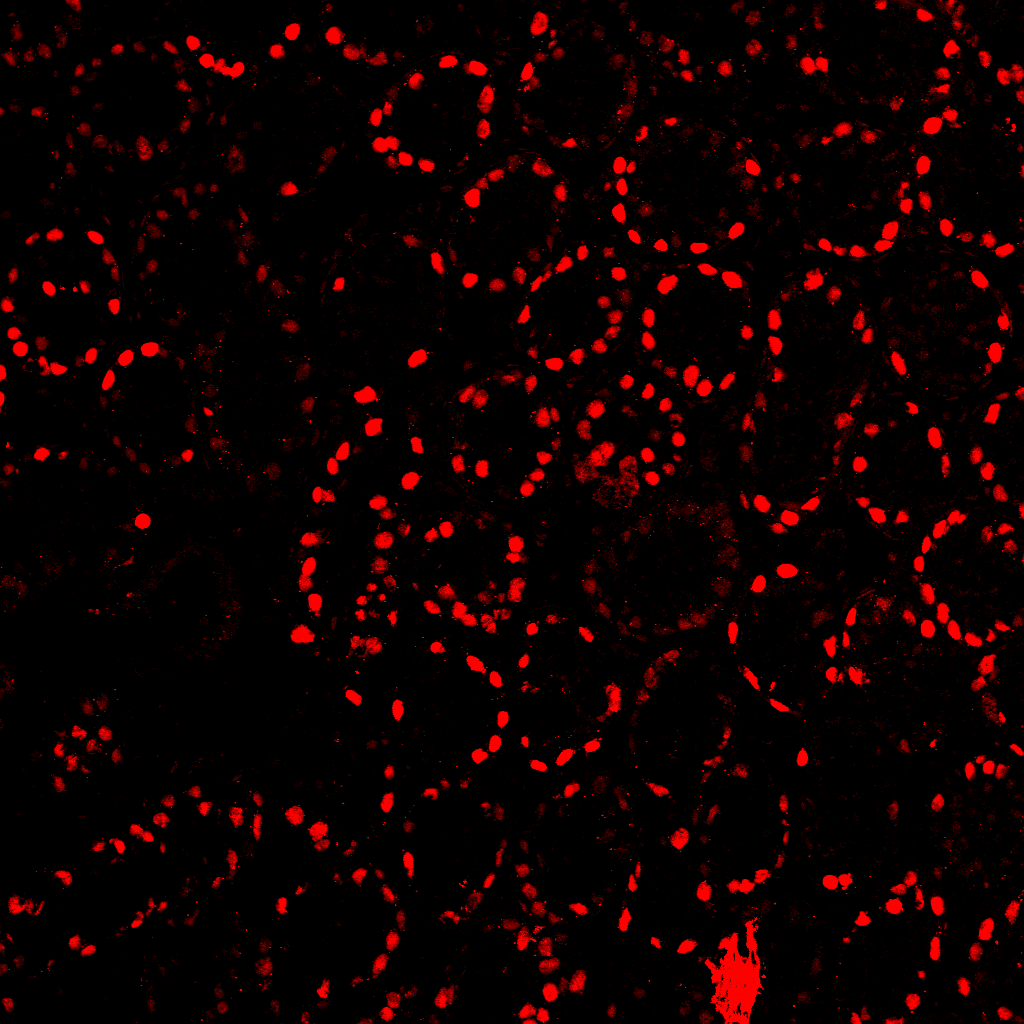

Supplement: Supplementary file 6 — Source data Fig. 3 [file 44319_2025_487_MOESM6_ESM.zip › Figure 3/3E/PD7 Brca1 Vasa-cre testis anti-PLZF&SOX3/PD7 Control testis anti-SOX3.tif]

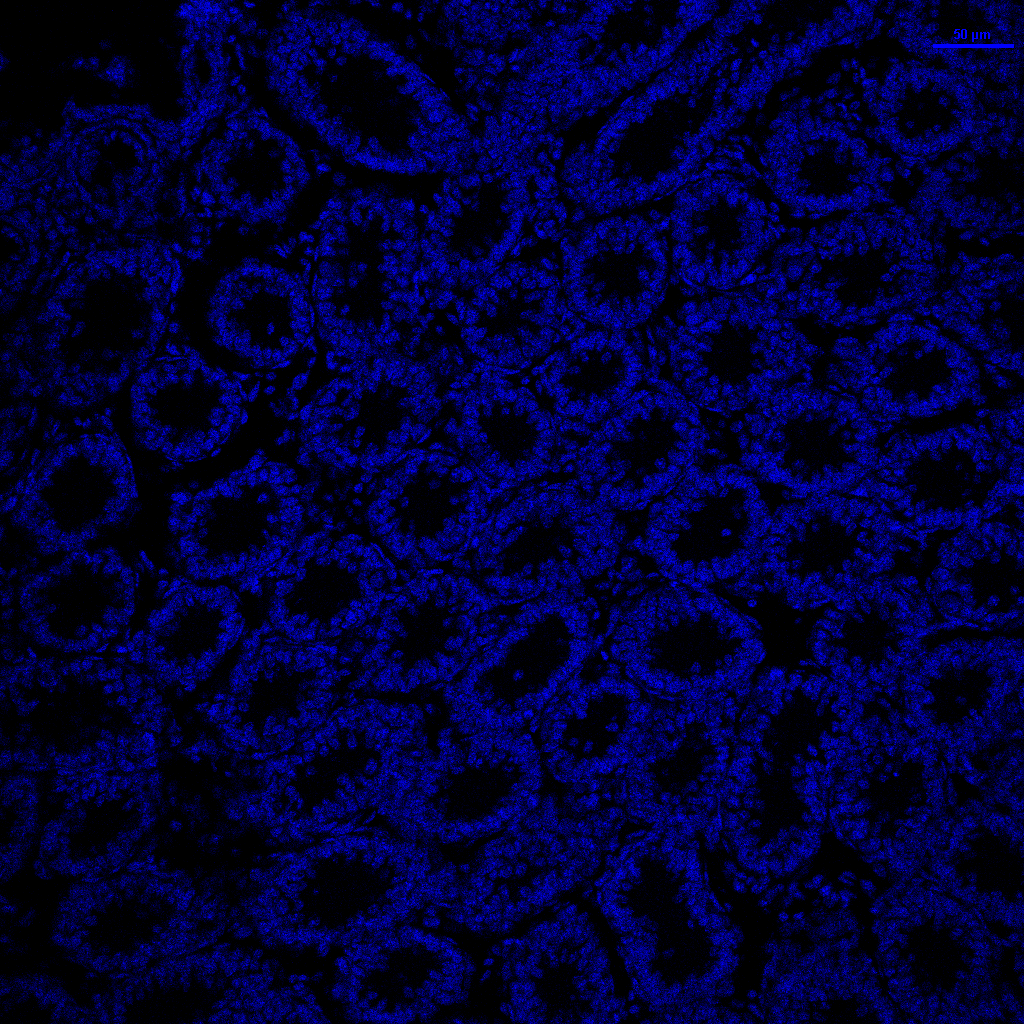

Supplement: Supplementary file 7 — Source data Fig. 4 [file 44319_2025_487_MOESM7_ESM.zip › Figure 4/4A/PD7 Brca1 vKO testis anti-GFRa1&p-H3(S10) Hoechst.tif]

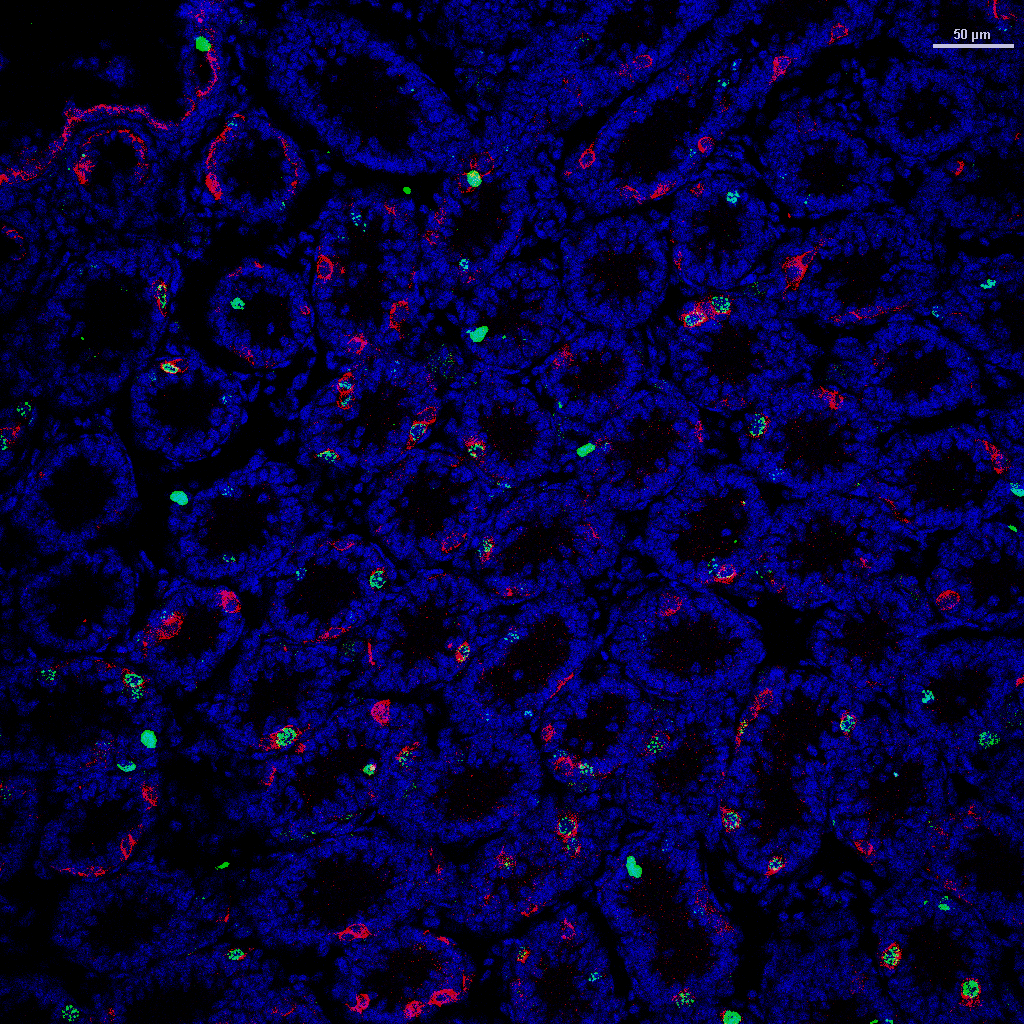

Supplement: Supplementary file 7 — Source data Fig. 4 [file 44319_2025_487_MOESM7_ESM.zip › Figure 4/4A/PD7 Brca1 vKO testis anti-GFRa1&p-H3(S10) Hoechst_overlay.tif]

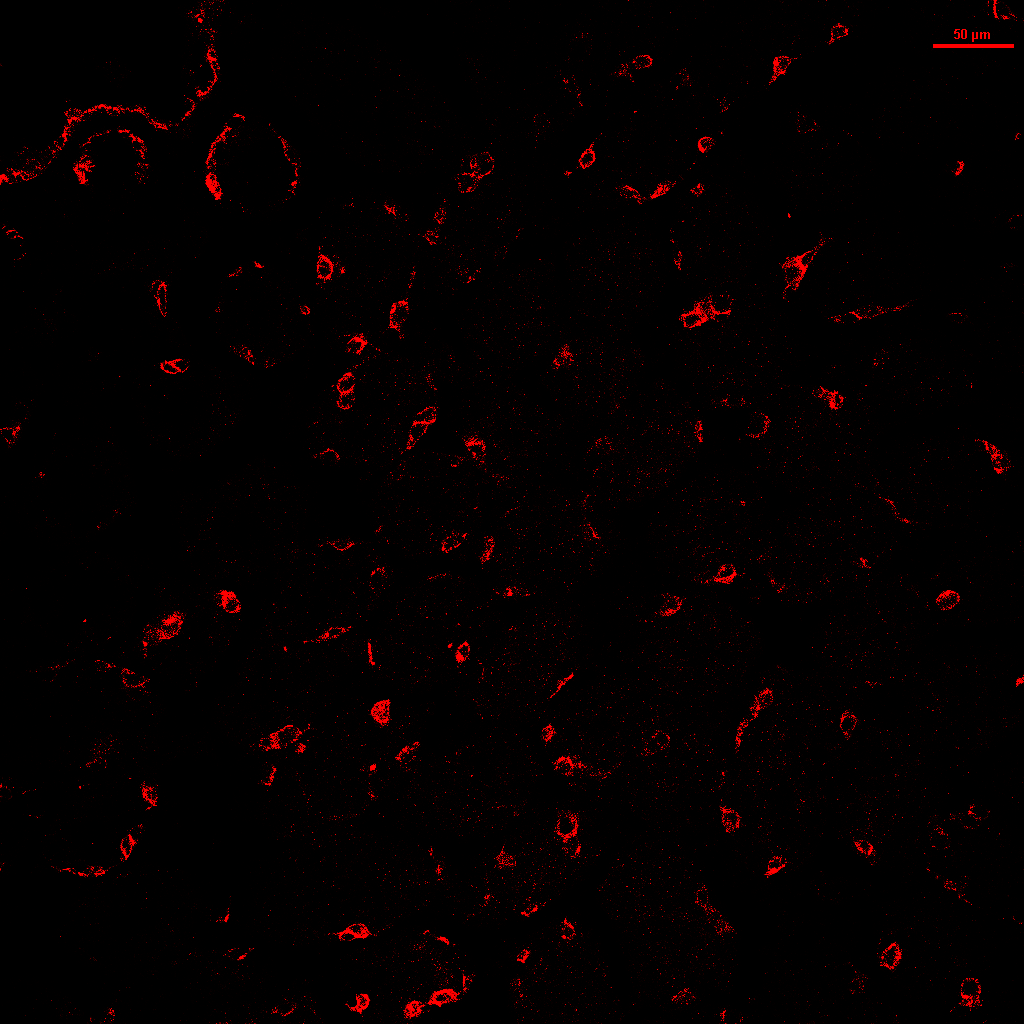

Supplement: Supplementary file 7 — Source data Fig. 4 [file 44319_2025_487_MOESM7_ESM.zip › Figure 4/4A/PD7 Brca1 vKO testis anti-GFRa1.tif]

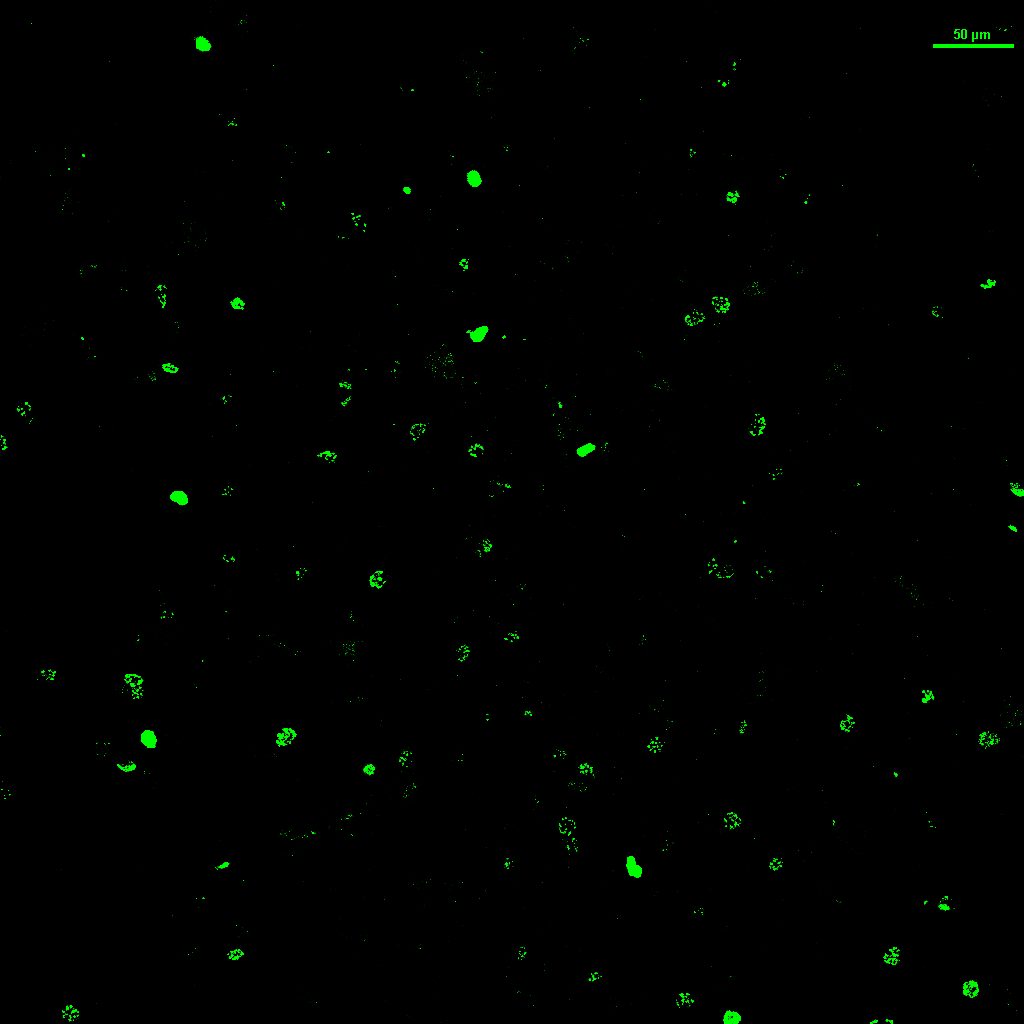

Supplement: Supplementary file 7 — Source data Fig. 4 [file 44319_2025_487_MOESM7_ESM.zip › Figure 4/4A/PD7 Brca1 vKO testis anti-p-H3(S10).tif]

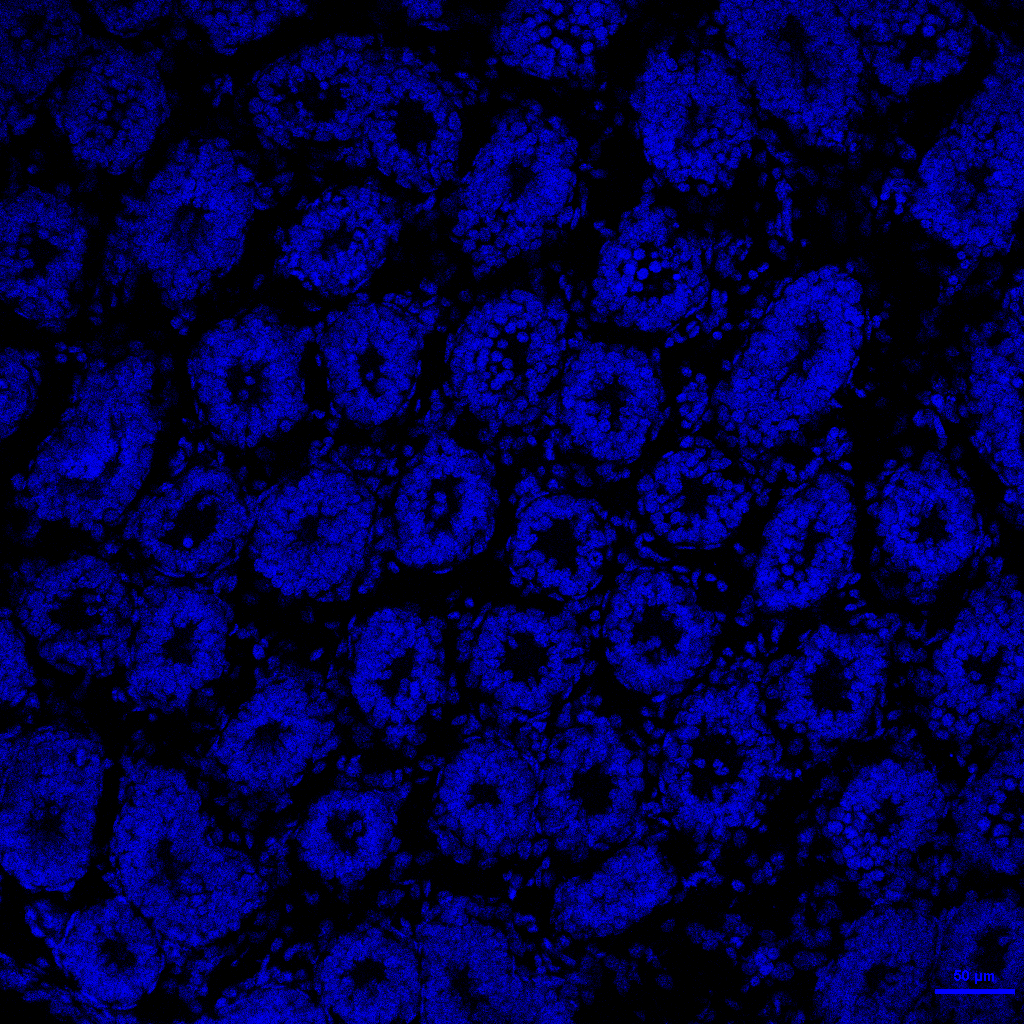

Supplement: Supplementary file 7 — Source data Fig. 4 [file 44319_2025_487_MOESM7_ESM.zip › Figure 4/4A/PD7 Control testis anti-GFRa1&p-H3(S10) Hoechst.tif]

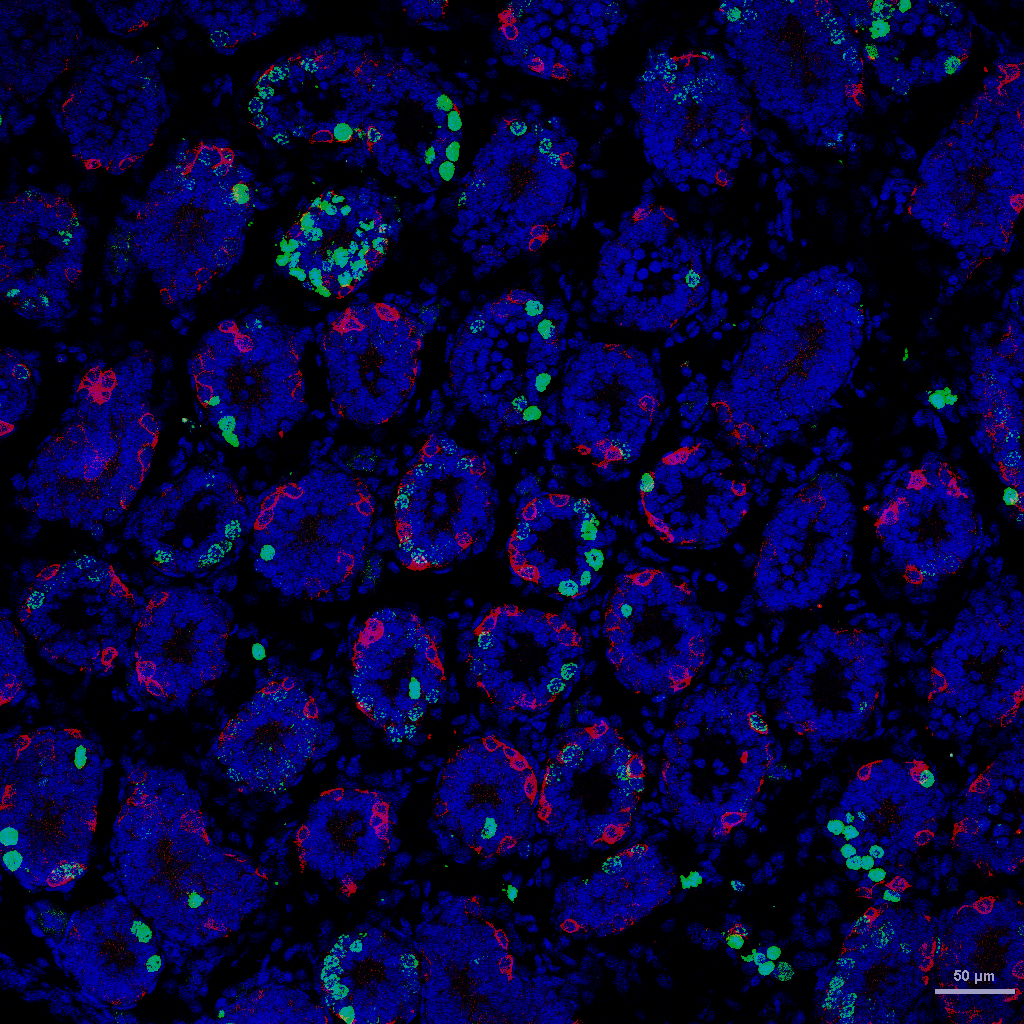

Supplement: Supplementary file 7 — Source data Fig. 4 [file 44319_2025_487_MOESM7_ESM.zip › Figure 4/4A/PD7 Control testis anti-GFRa1&p-H3(S10) Hoechst_overlay.tif]

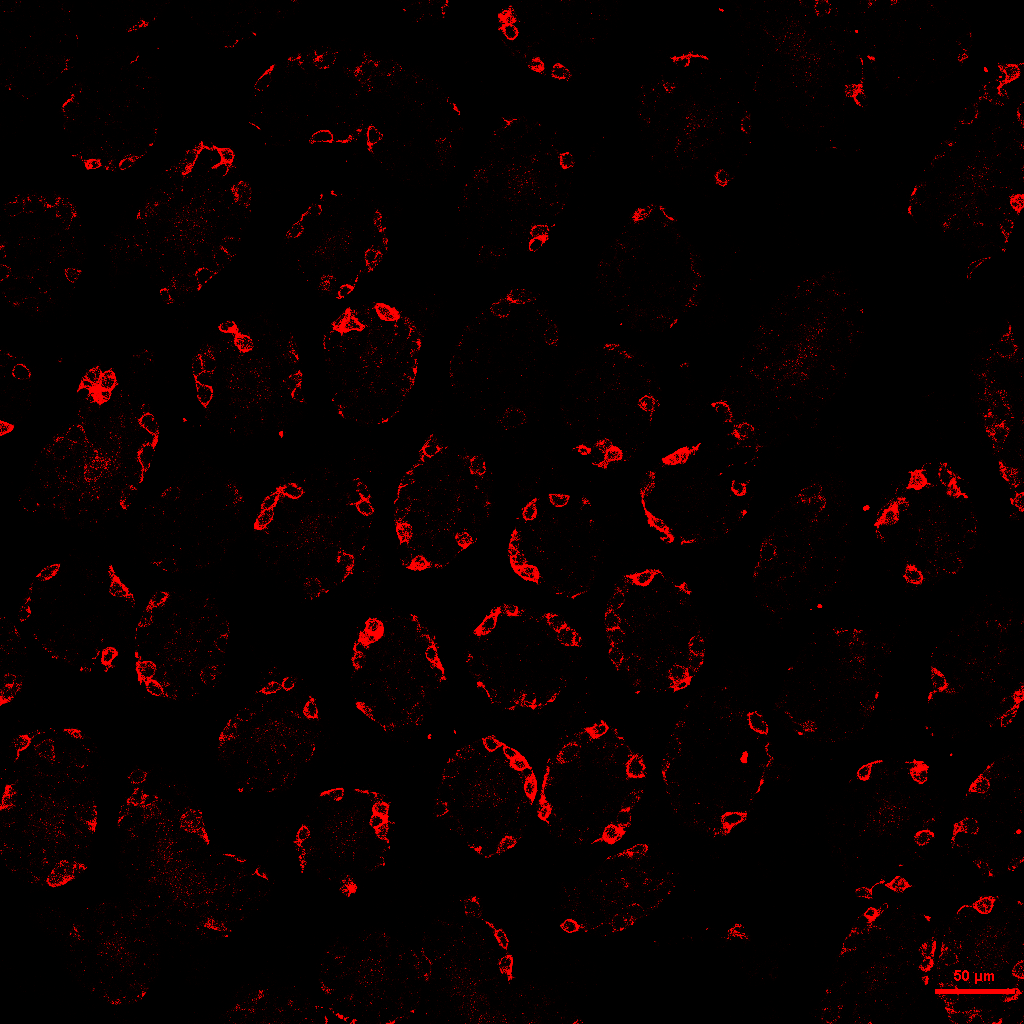

Supplement: Supplementary file 7 — Source data Fig. 4 [file 44319_2025_487_MOESM7_ESM.zip › Figure 4/4A/PD7 Control testis anti-GFRa1.tif]
