# Supplementary figures and images for: BRCA1 preserves genome integrity during the formation of undifferentiated spermatogonia (part 2 of 3)
Source: EMBO Rep. 2025 May 28;26(15):3747–72. doi: 10.1038/s44319-025-00487-5 (PMC12332178; doi:10.1038/s44319-025-00487-5)

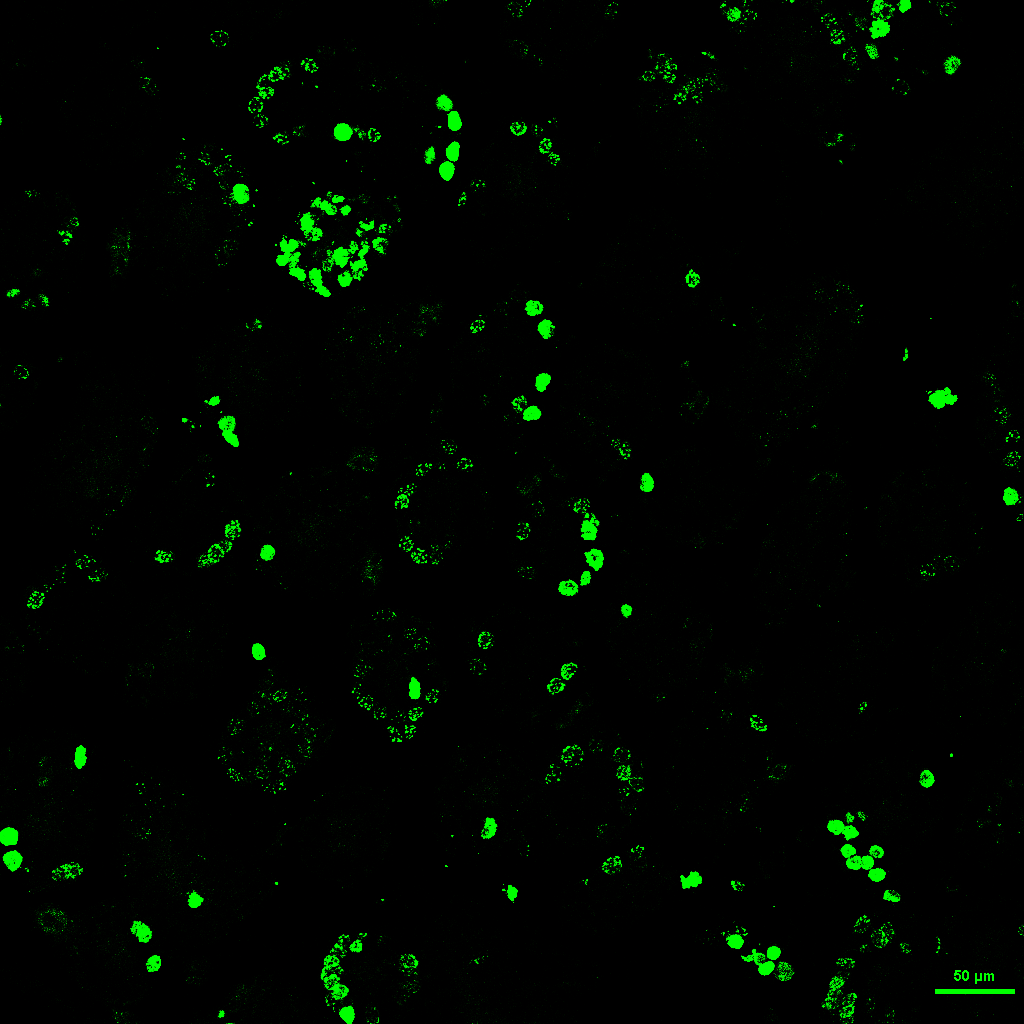

Supplement: Supplementary file 7 — Source data Fig. 4 [file 44319_2025_487_MOESM7_ESM.zip › Figure 4/4A/PD7 Control testis anti-p-H3(S10).tif]

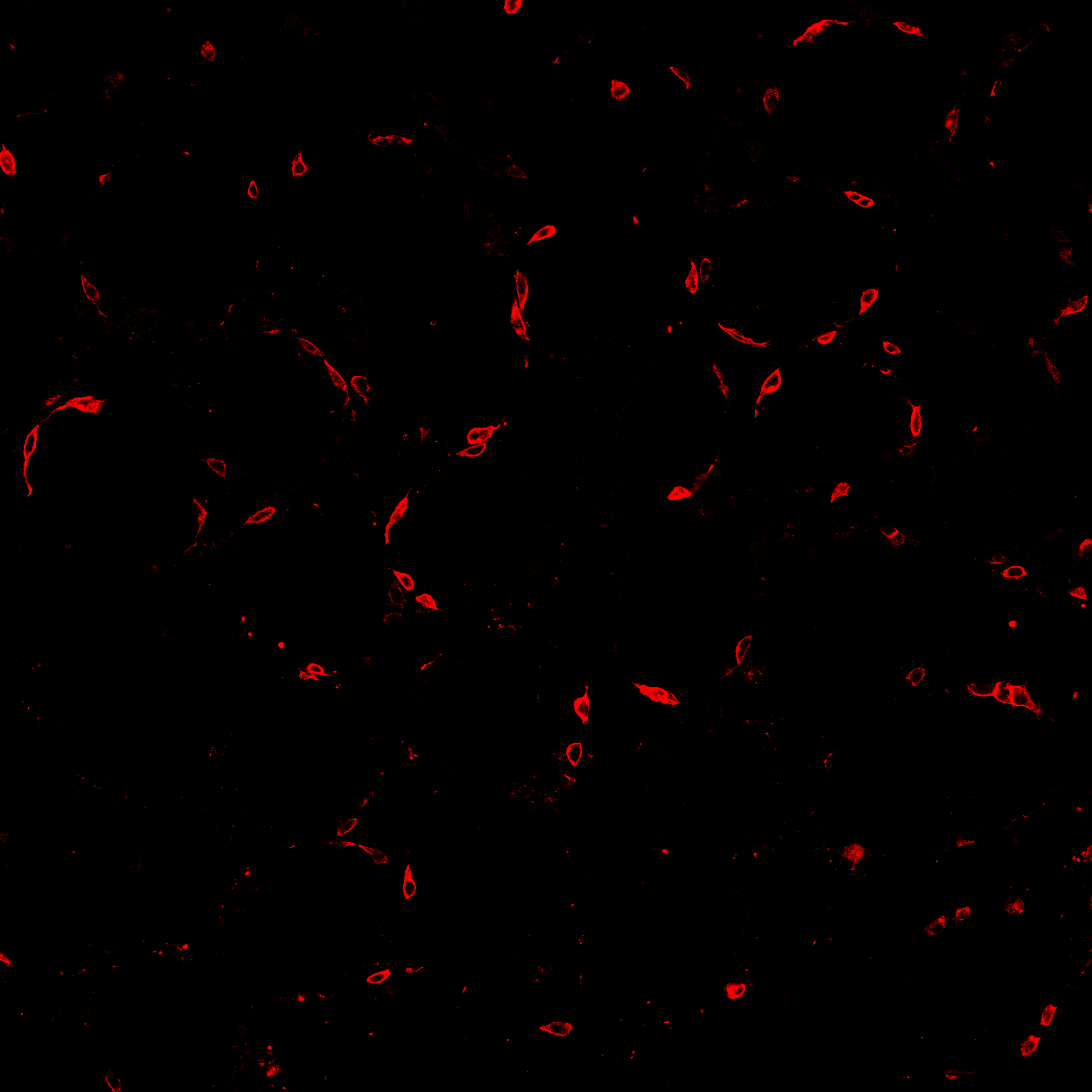

Supplement: Supplementary file 7 — Source data Fig. 4 [file 44319_2025_487_MOESM7_ESM.zip › Figure 4/4C/PD14 Brca1 Vasa-cre testis anti-PLZF&GFRa1/PD14 Brca1 vKO testis anti-GFRa1.tif]

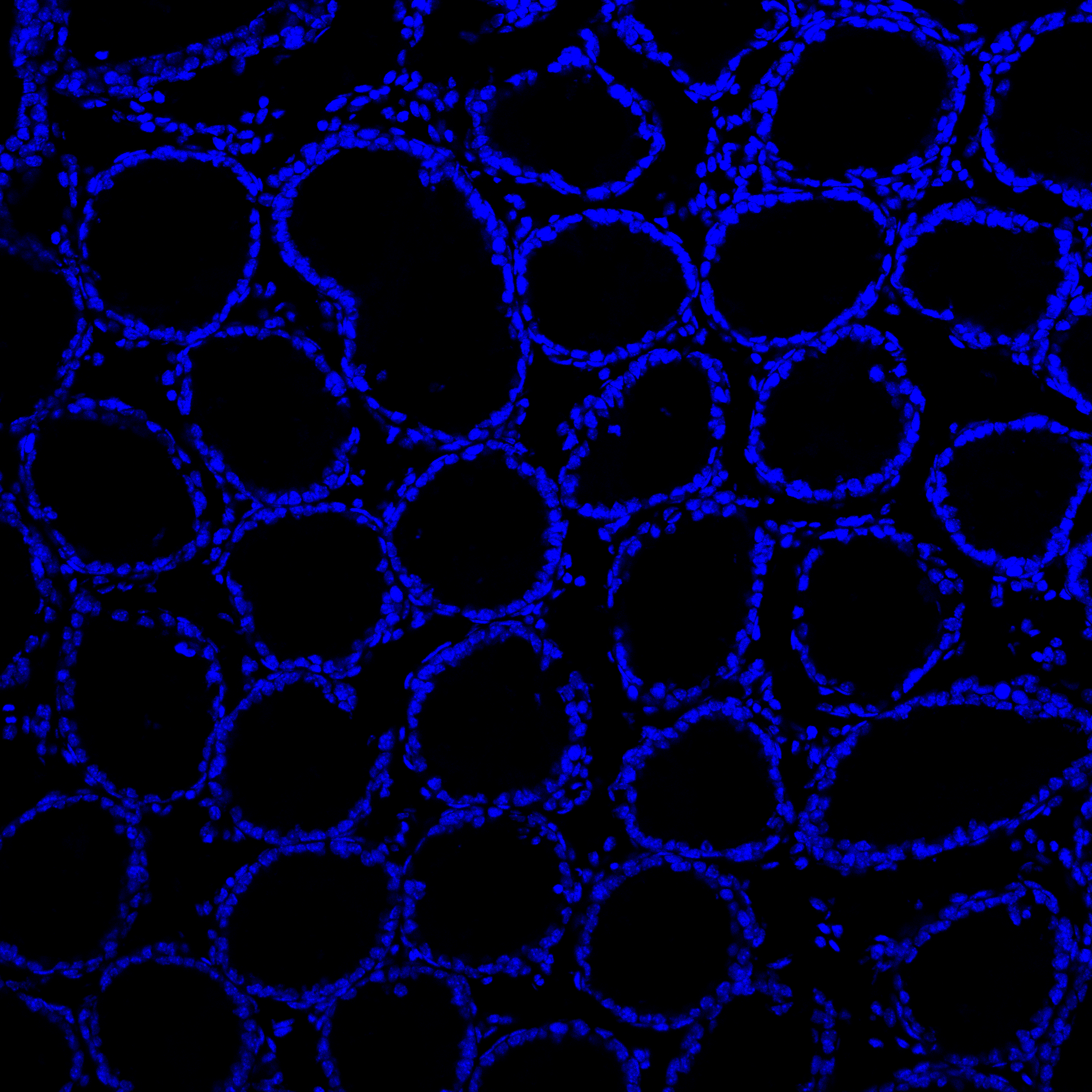

Supplement: Supplementary file 7 — Source data Fig. 4 [file 44319_2025_487_MOESM7_ESM.zip › Figure 4/4C/PD14 Brca1 Vasa-cre testis anti-PLZF&GFRa1/PD14 Brca1 vKO testis anti-PLZF&GFRa1 Hoechst.tif]

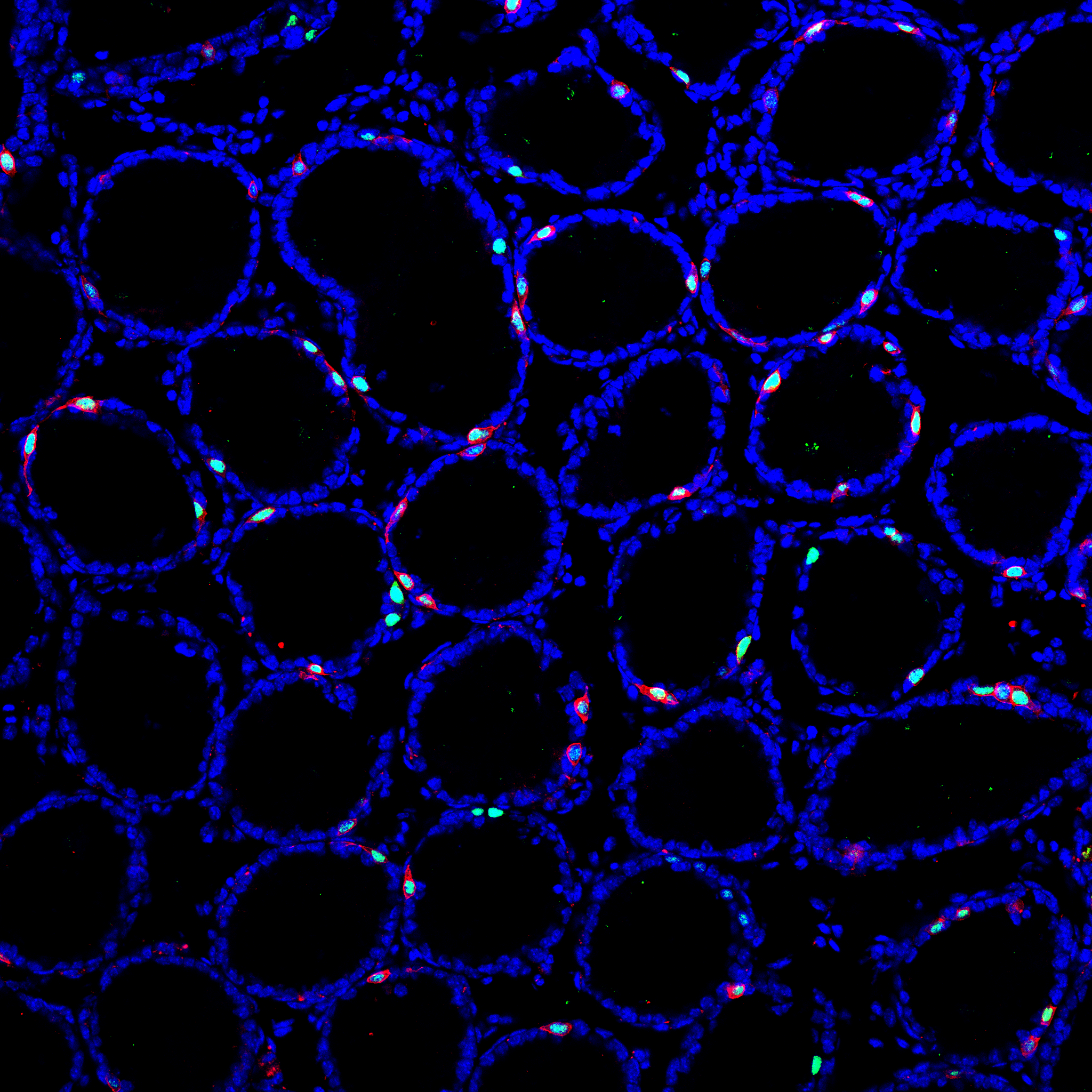

Supplement: Supplementary file 7 — Source data Fig. 4 [file 44319_2025_487_MOESM7_ESM.zip › Figure 4/4C/PD14 Brca1 Vasa-cre testis anti-PLZF&GFRa1/PD14 Brca1 vKO testis anti-PLZF&GFRa1 Hoechst_overlay.tif]

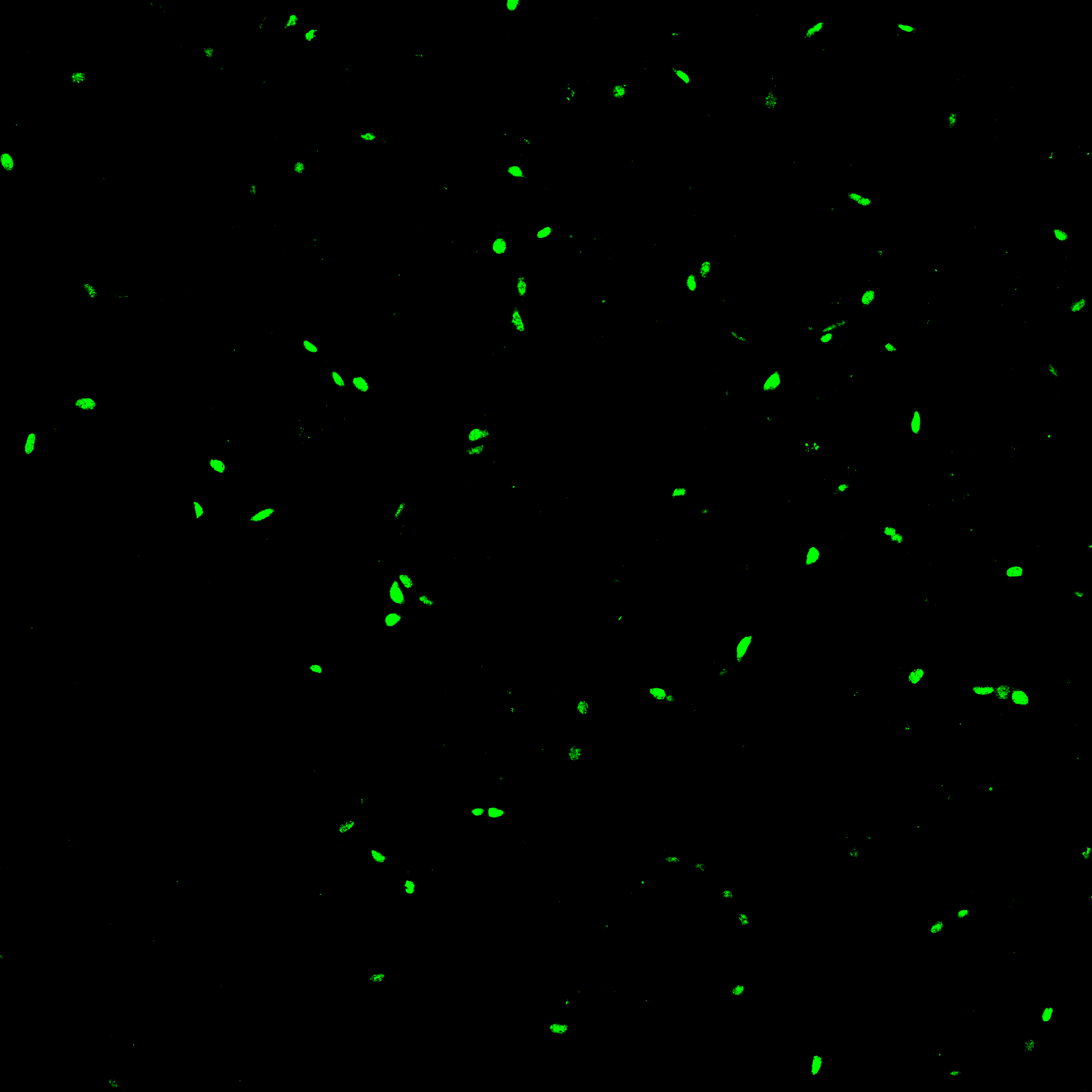

Supplement: Supplementary file 7 — Source data Fig. 4 [file 44319_2025_487_MOESM7_ESM.zip › Figure 4/4C/PD14 Brca1 Vasa-cre testis anti-PLZF&GFRa1/PD14 Brca1 vKO testis anti-PLZF.tif]

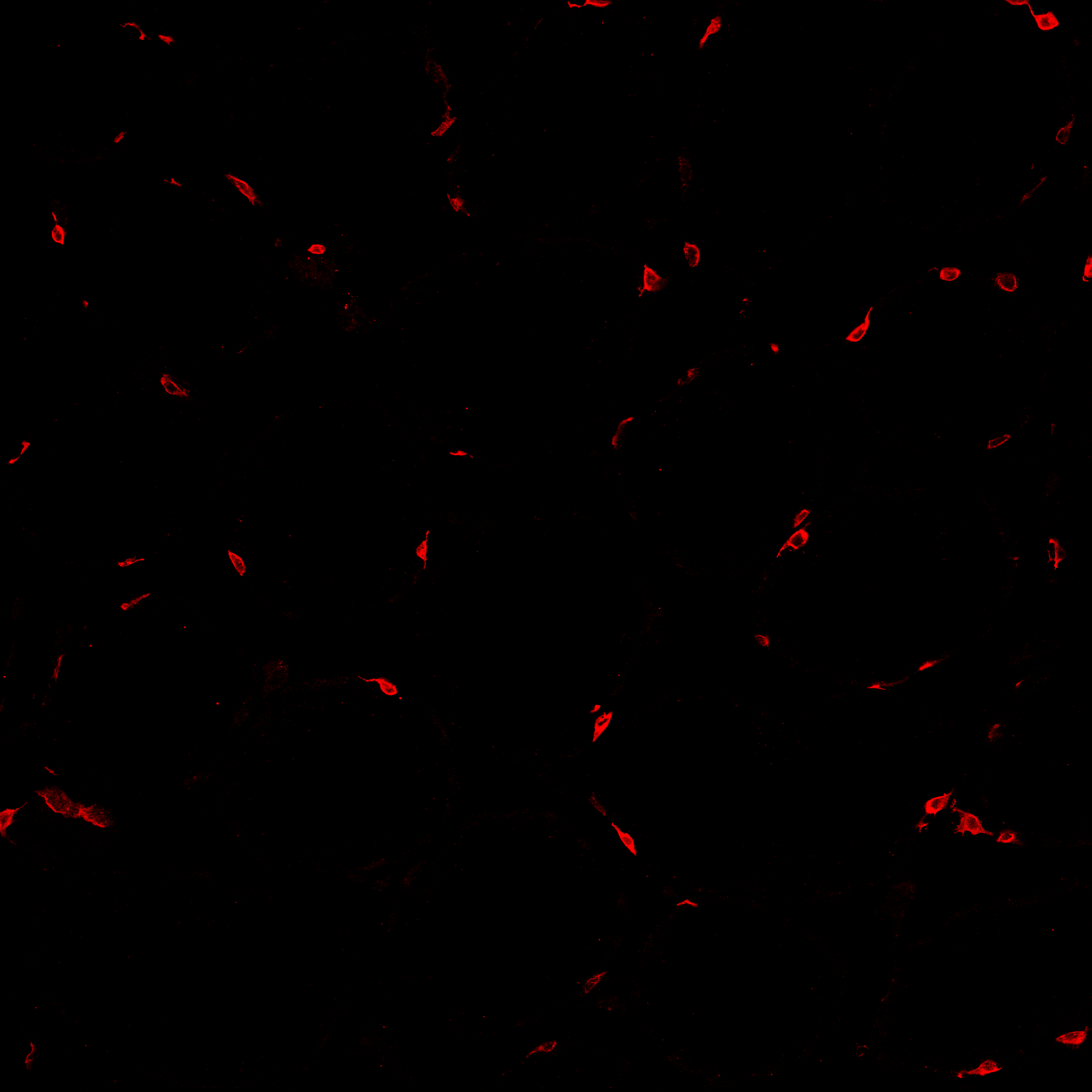

Supplement: Supplementary file 7 — Source data Fig. 4 [file 44319_2025_487_MOESM7_ESM.zip › Figure 4/4C/PD14 Brca1 Vasa-cre testis anti-PLZF&GFRa1/PD14 Control testis anti-GFRa1.tif]

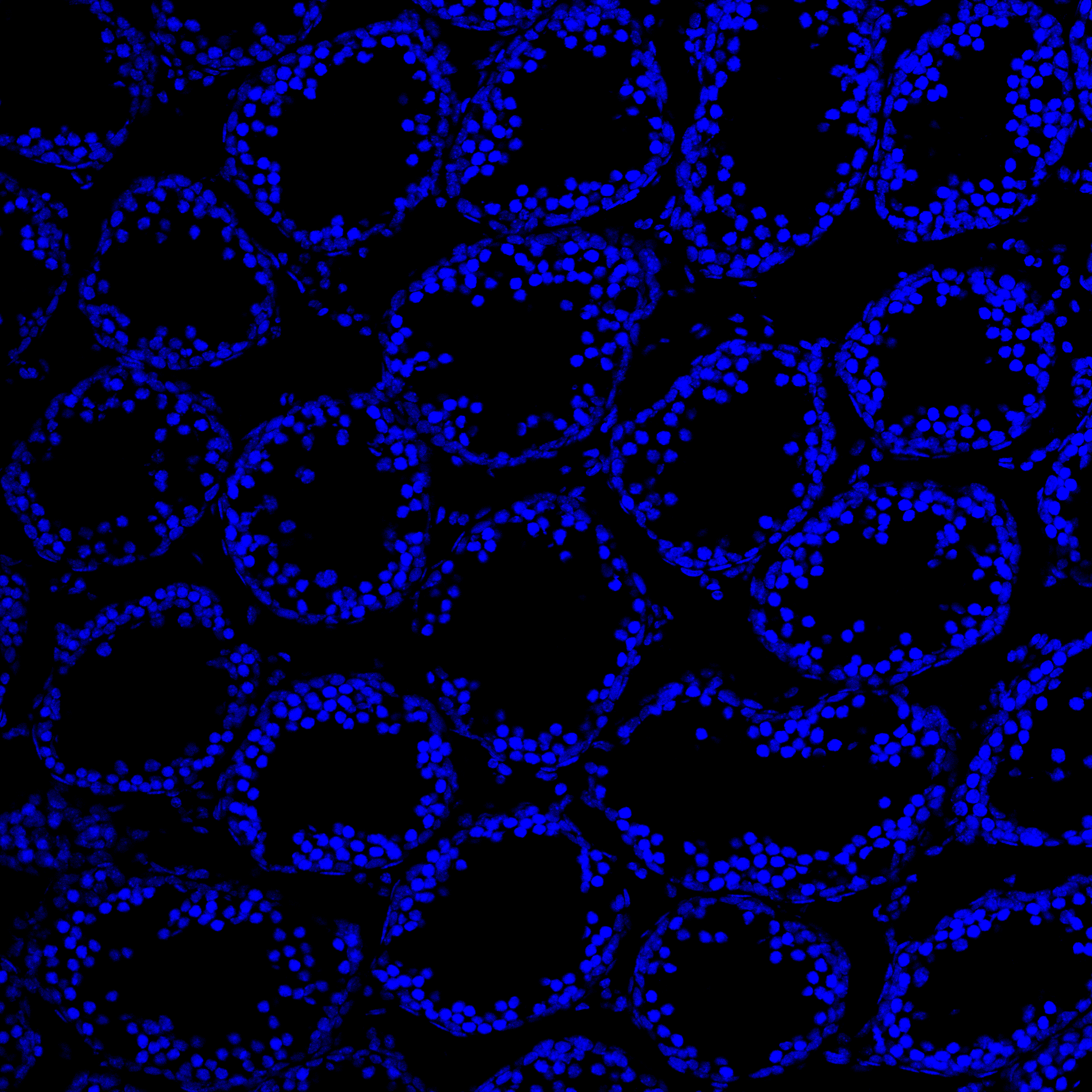

Supplement: Supplementary file 7 — Source data Fig. 4 [file 44319_2025_487_MOESM7_ESM.zip › Figure 4/4C/PD14 Brca1 Vasa-cre testis anti-PLZF&GFRa1/PD14 Control testis anti-PLZF&GFRa1 Hoechst.tif]

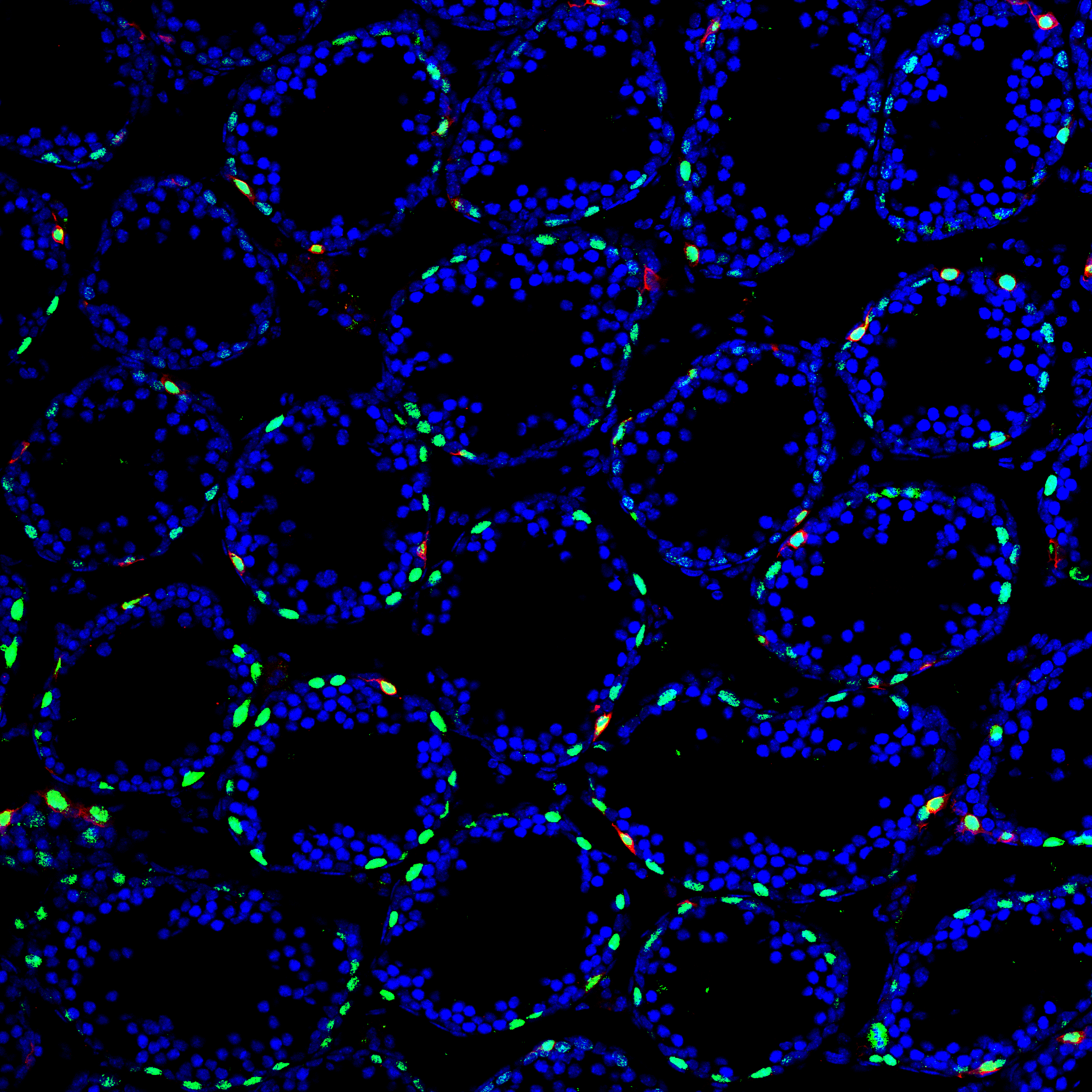

Supplement: Supplementary file 7 — Source data Fig. 4 [file 44319_2025_487_MOESM7_ESM.zip › Figure 4/4C/PD14 Brca1 Vasa-cre testis anti-PLZF&GFRa1/PD14 Control testis anti-PLZF&GFRa1 Hoechst_overlay.tif]

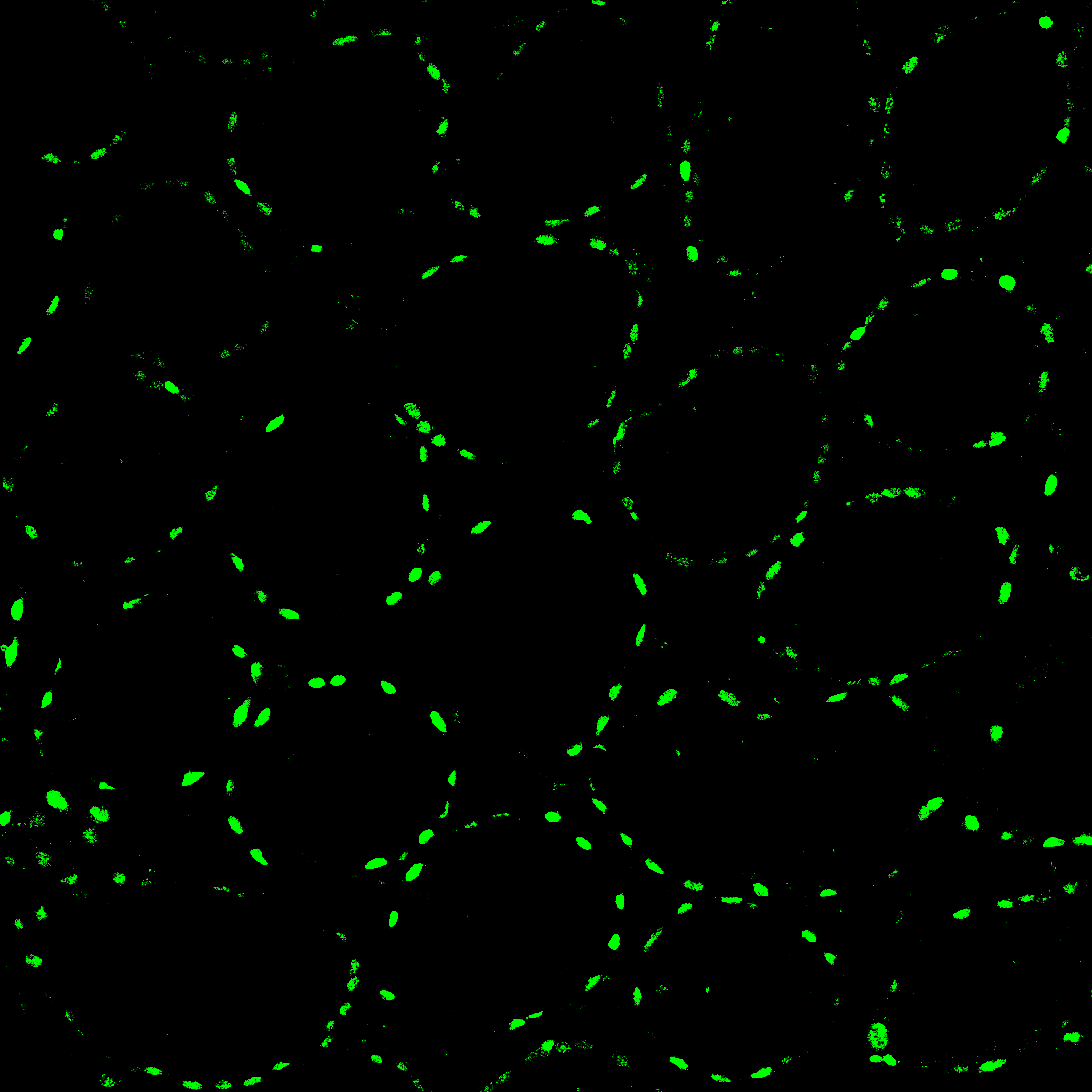

Supplement: Supplementary file 7 — Source data Fig. 4 [file 44319_2025_487_MOESM7_ESM.zip › Figure 4/4C/PD14 Brca1 Vasa-cre testis anti-PLZF&GFRa1/PD14 Control testis anti-PLZF.tif]

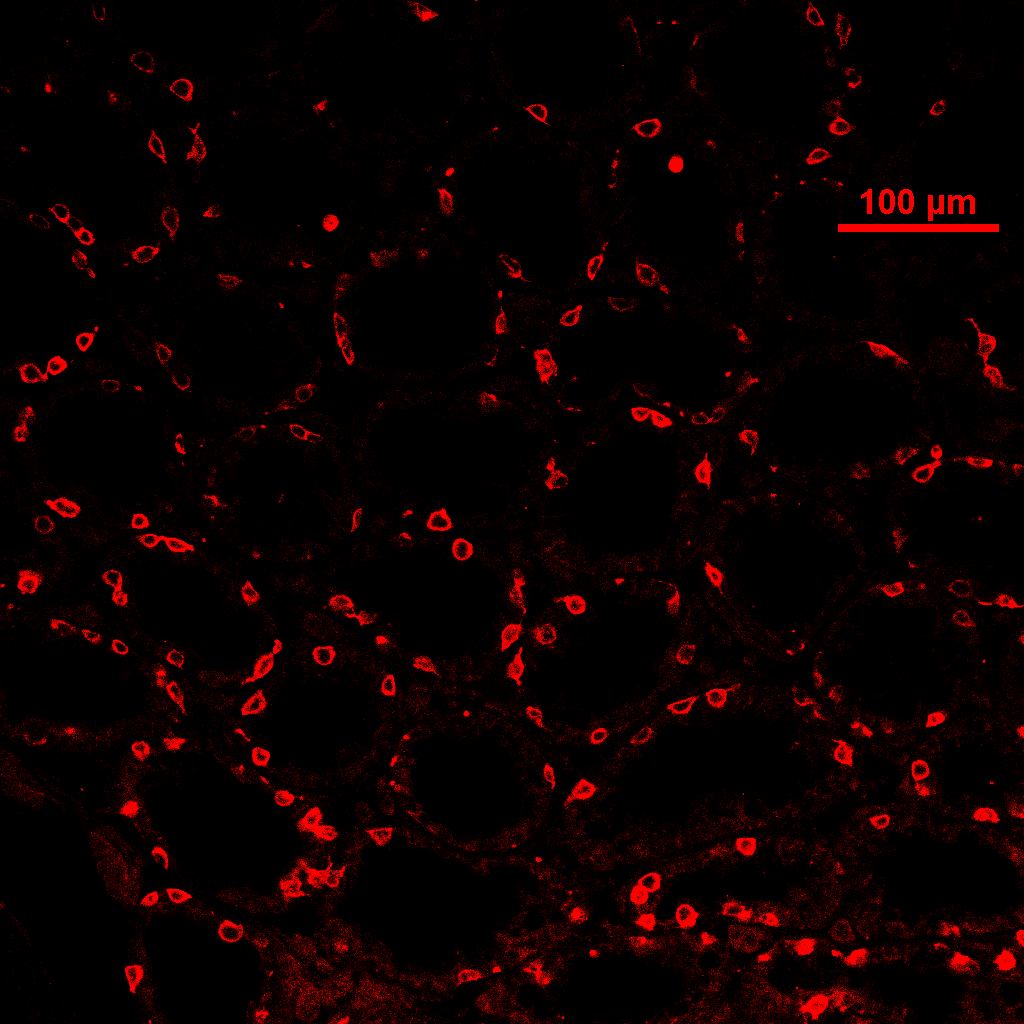

Supplement: Supplementary file 7 — Source data Fig. 4 [file 44319_2025_487_MOESM7_ESM.zip › Figure 4/4C/PD21 Brca1 Vasa-cre testis anti-PLZF&GFRa1/PD21 Brca1 vKO testis anti-GFRa1.tif]

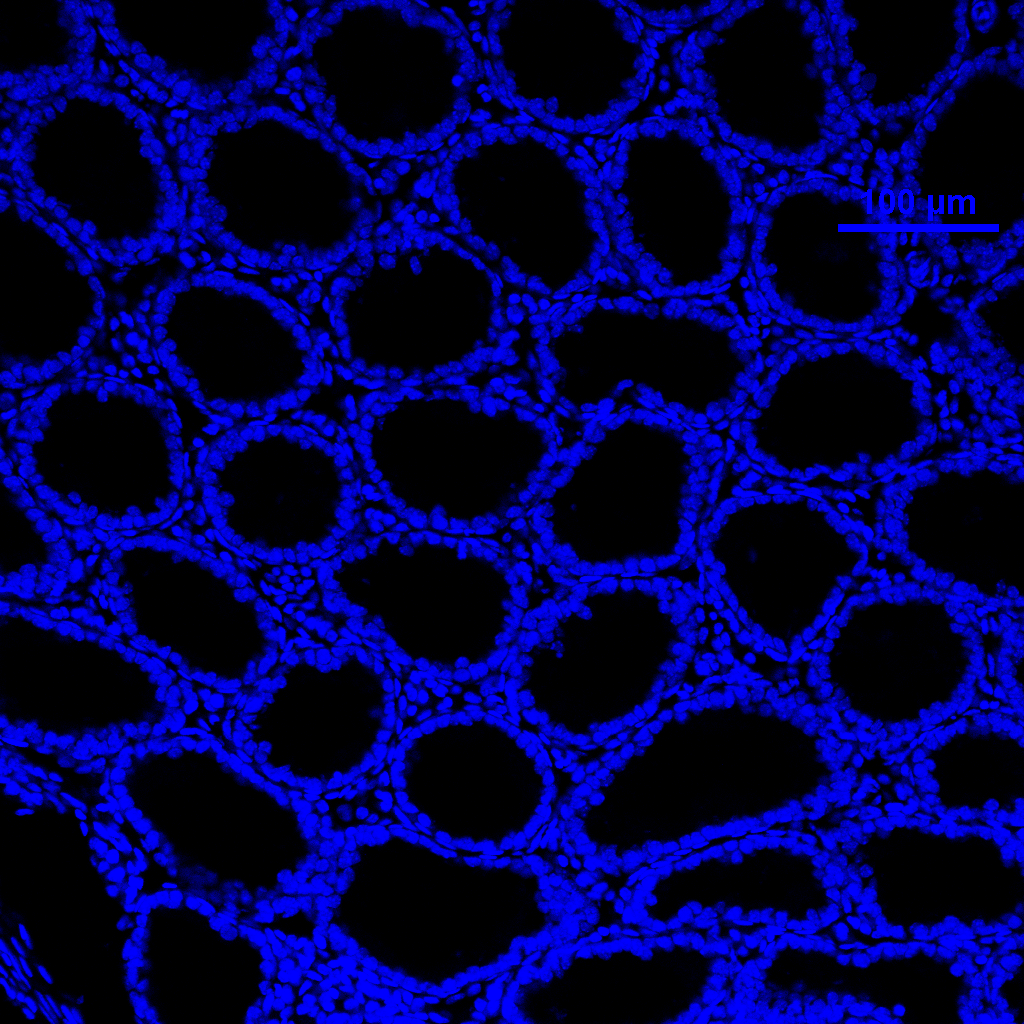

Supplement: Supplementary file 7 — Source data Fig. 4 [file 44319_2025_487_MOESM7_ESM.zip › Figure 4/4C/PD21 Brca1 Vasa-cre testis anti-PLZF&GFRa1/PD21 Brca1 vKO testis anti-PLZF&GFRa1 Hoechst.tif]

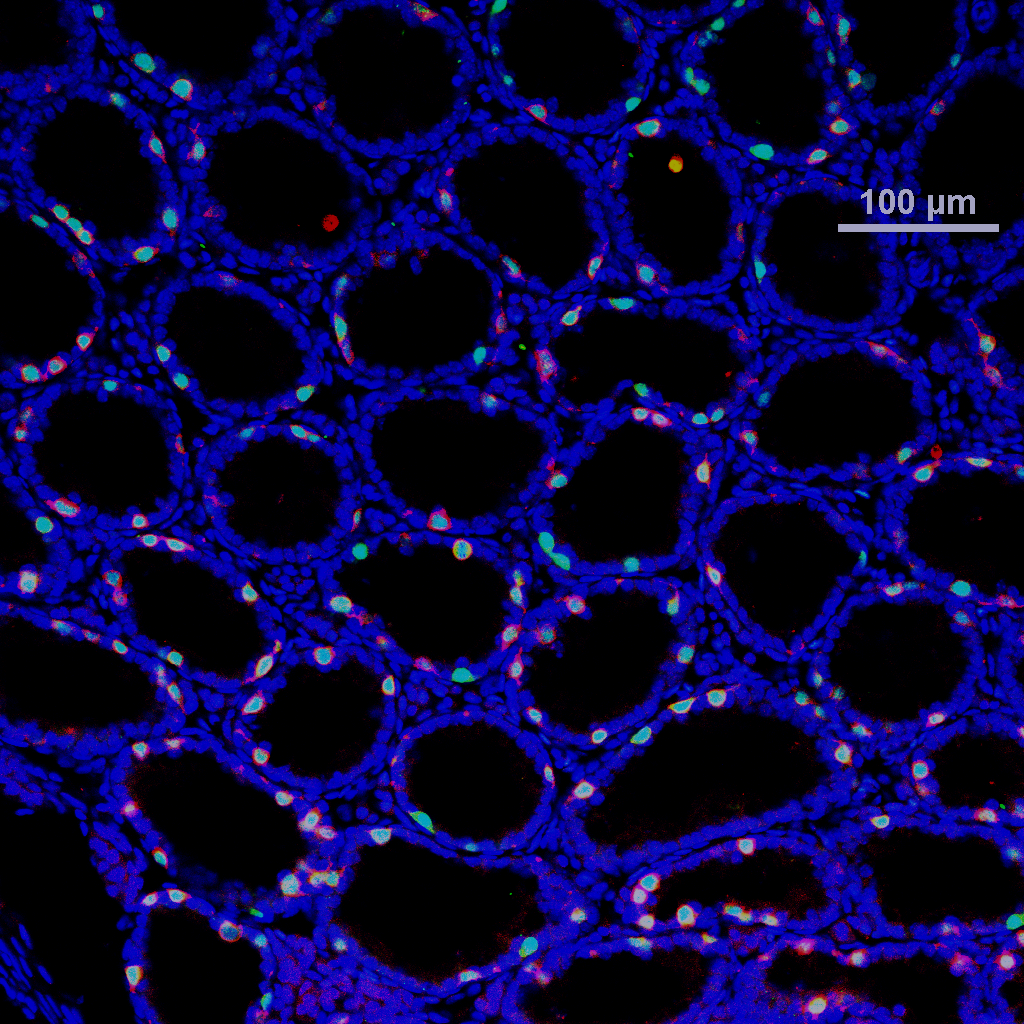

Supplement: Supplementary file 7 — Source data Fig. 4 [file 44319_2025_487_MOESM7_ESM.zip › Figure 4/4C/PD21 Brca1 Vasa-cre testis anti-PLZF&GFRa1/PD21 Brca1 vKO testis anti-PLZF&GFRa1 Hoechst_overlay.tif]

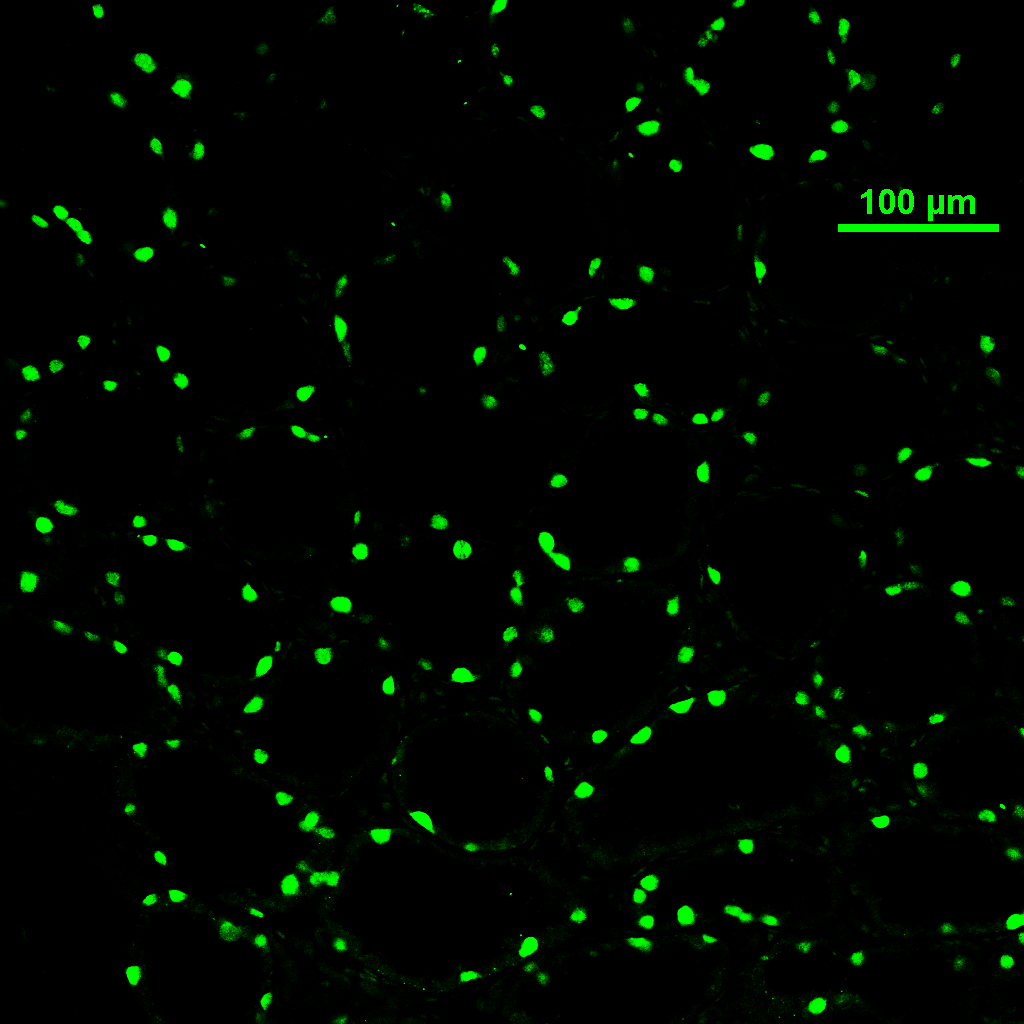

Supplement: Supplementary file 7 — Source data Fig. 4 [file 44319_2025_487_MOESM7_ESM.zip › Figure 4/4C/PD21 Brca1 Vasa-cre testis anti-PLZF&GFRa1/PD21 Brca1 vKO testis anti-PLZF.tif]

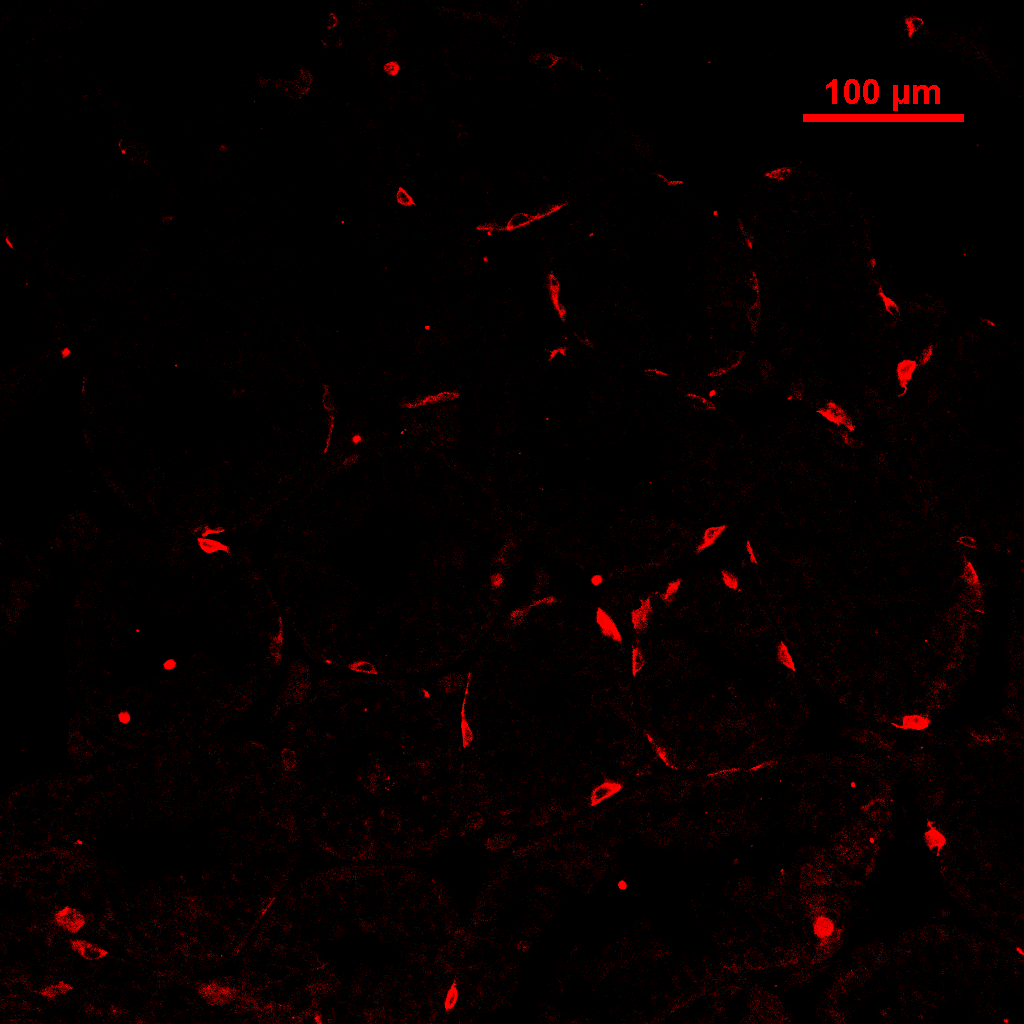

Supplement: Supplementary file 7 — Source data Fig. 4 [file 44319_2025_487_MOESM7_ESM.zip › Figure 4/4C/PD21 Brca1 Vasa-cre testis anti-PLZF&GFRa1/PD21 Control testis anti-GFRa1.tif]

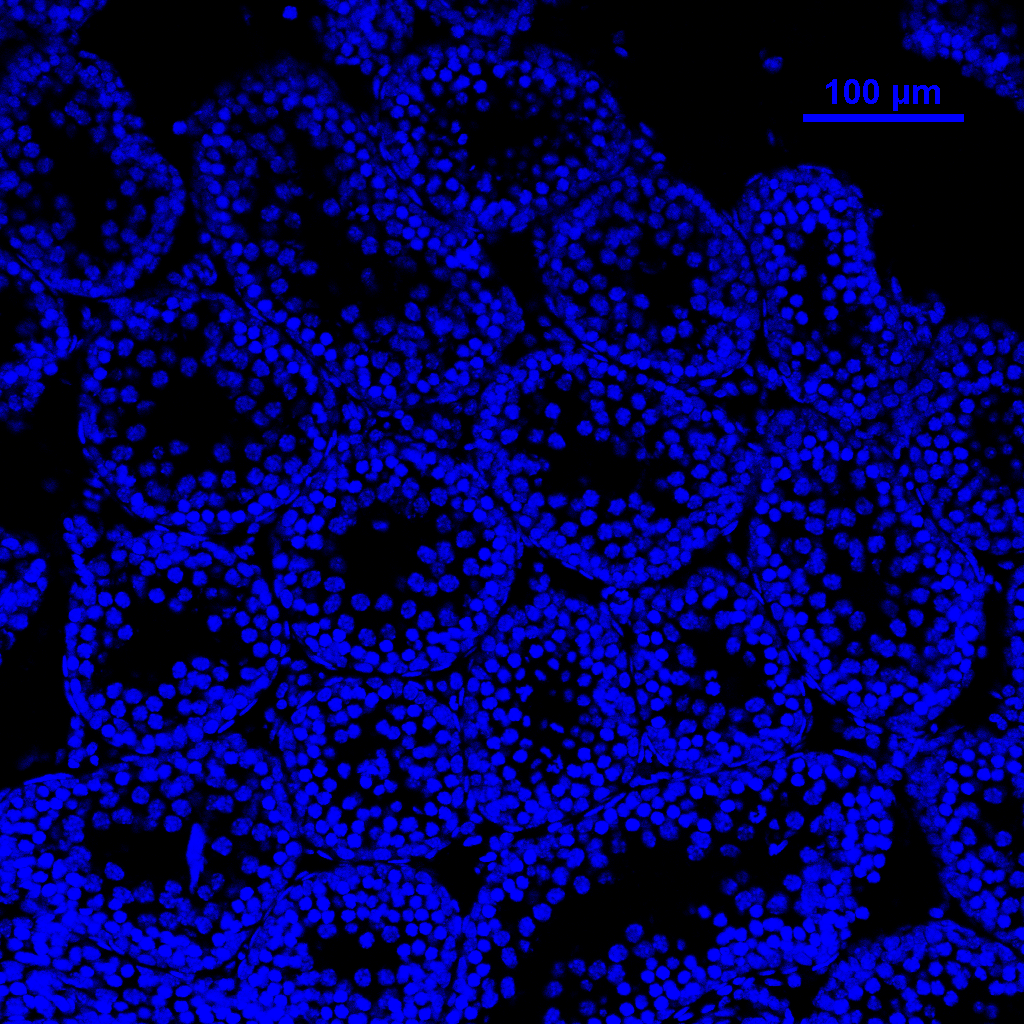

Supplement: Supplementary file 7 — Source data Fig. 4 [file 44319_2025_487_MOESM7_ESM.zip › Figure 4/4C/PD21 Brca1 Vasa-cre testis anti-PLZF&GFRa1/PD21 Control testis anti-PLZF&GFRa1 Hoechst.tif]

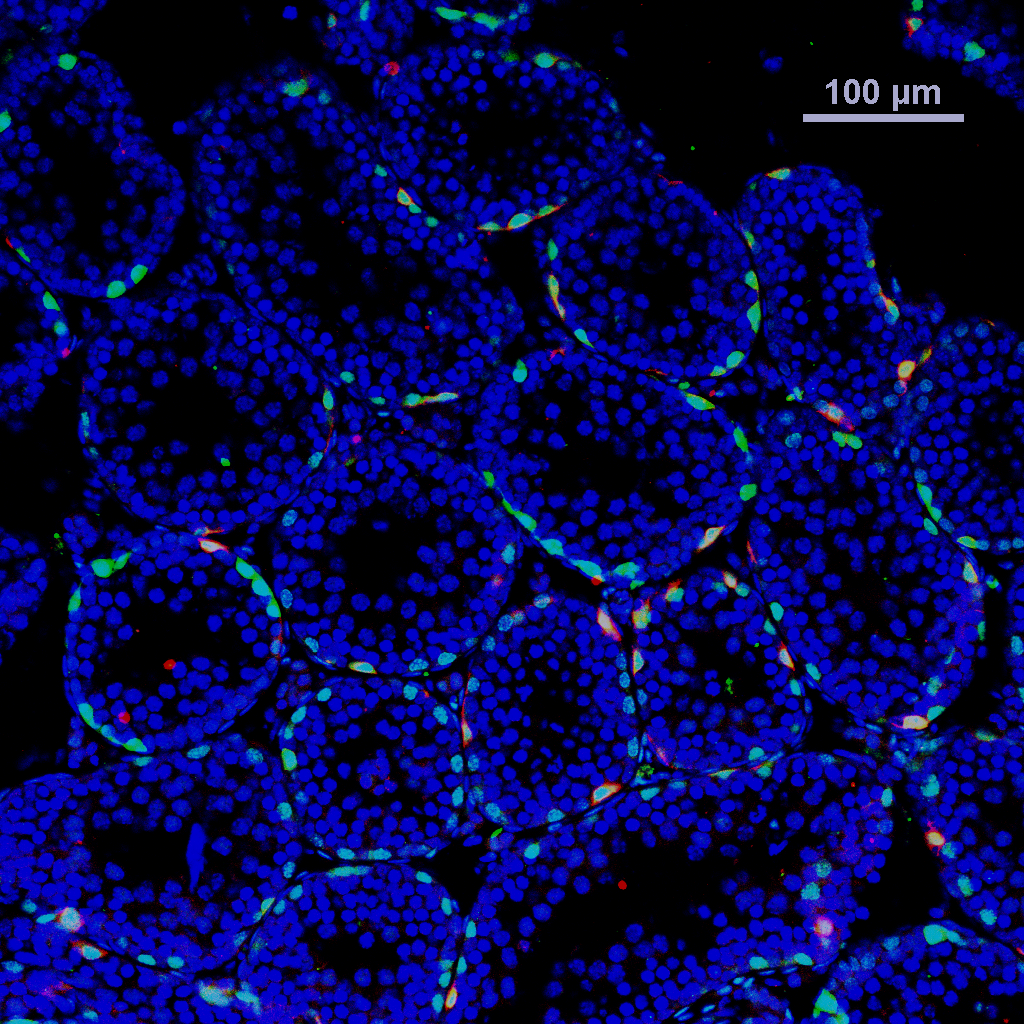

Supplement: Supplementary file 7 — Source data Fig. 4 [file 44319_2025_487_MOESM7_ESM.zip › Figure 4/4C/PD21 Brca1 Vasa-cre testis anti-PLZF&GFRa1/PD21 Control testis anti-PLZF&GFRa1 Hoechst_overlay.tif]

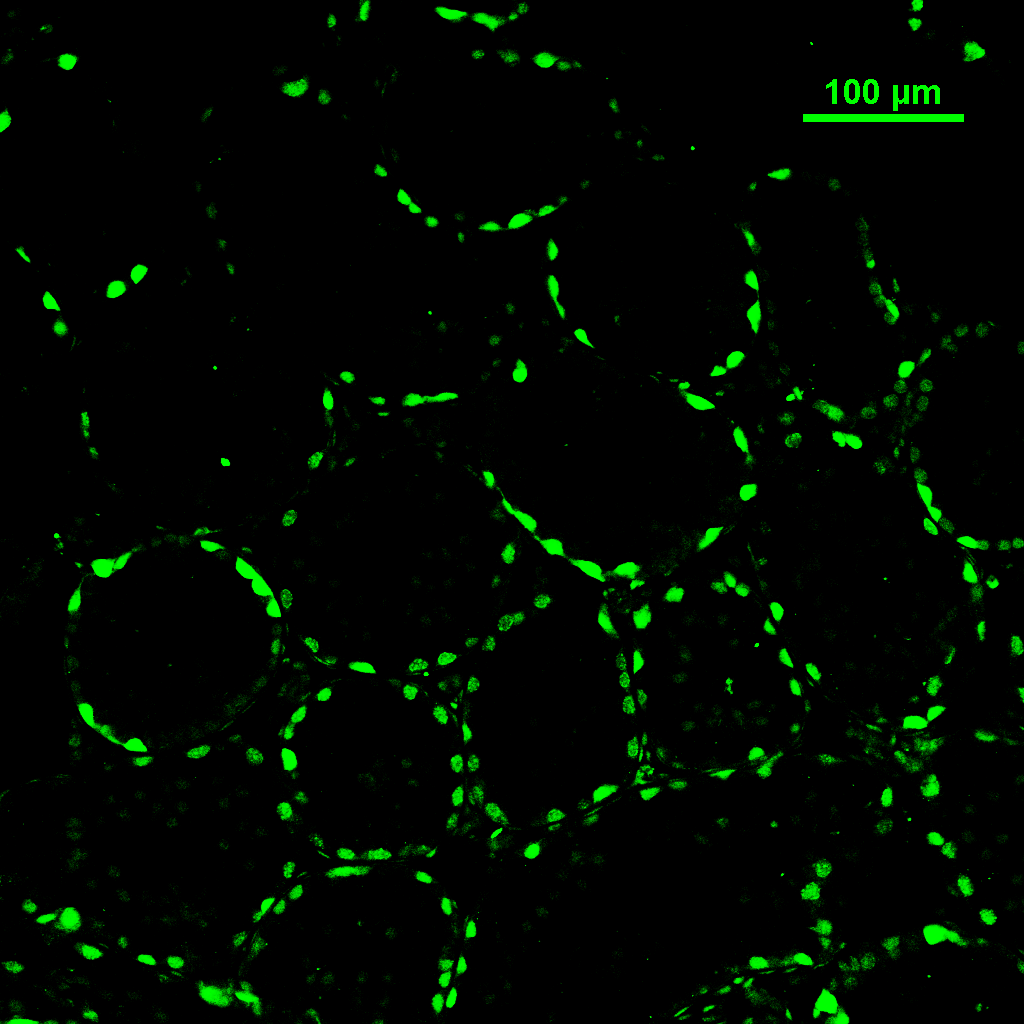

Supplement: Supplementary file 7 — Source data Fig. 4 [file 44319_2025_487_MOESM7_ESM.zip › Figure 4/4C/PD21 Brca1 Vasa-cre testis anti-PLZF&GFRa1/PD21 Control testis anti-PLZF.tif]

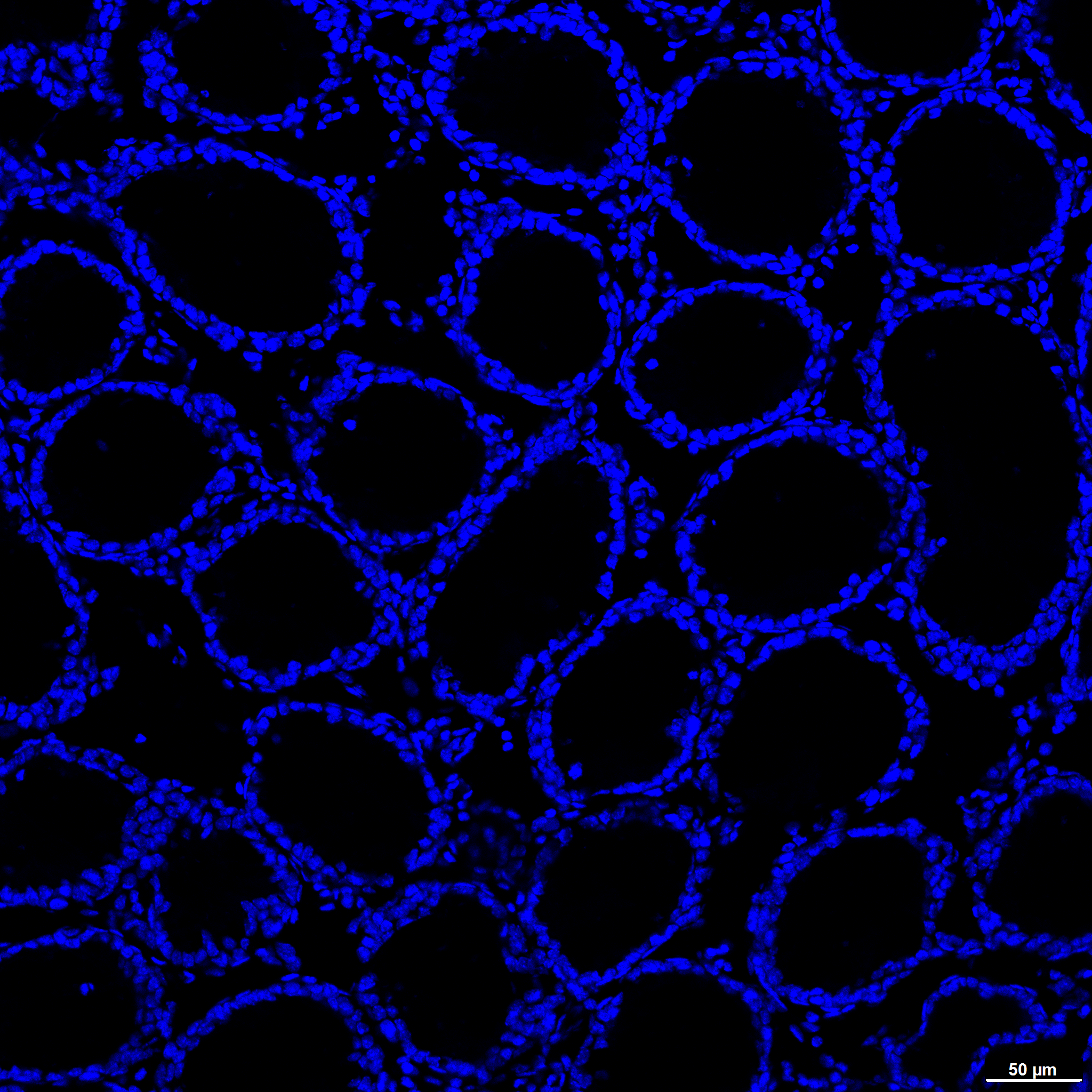

Supplement: Supplementary file 7 — Source data Fig. 4 [file 44319_2025_487_MOESM7_ESM.zip › Figure 4/4E/PD14 Brca1 Vasa-cre testis anti-PLZF&SOX3/PD14 Brca1 vKO testis anti-PLZF&SOX3 Hoechst.tif]

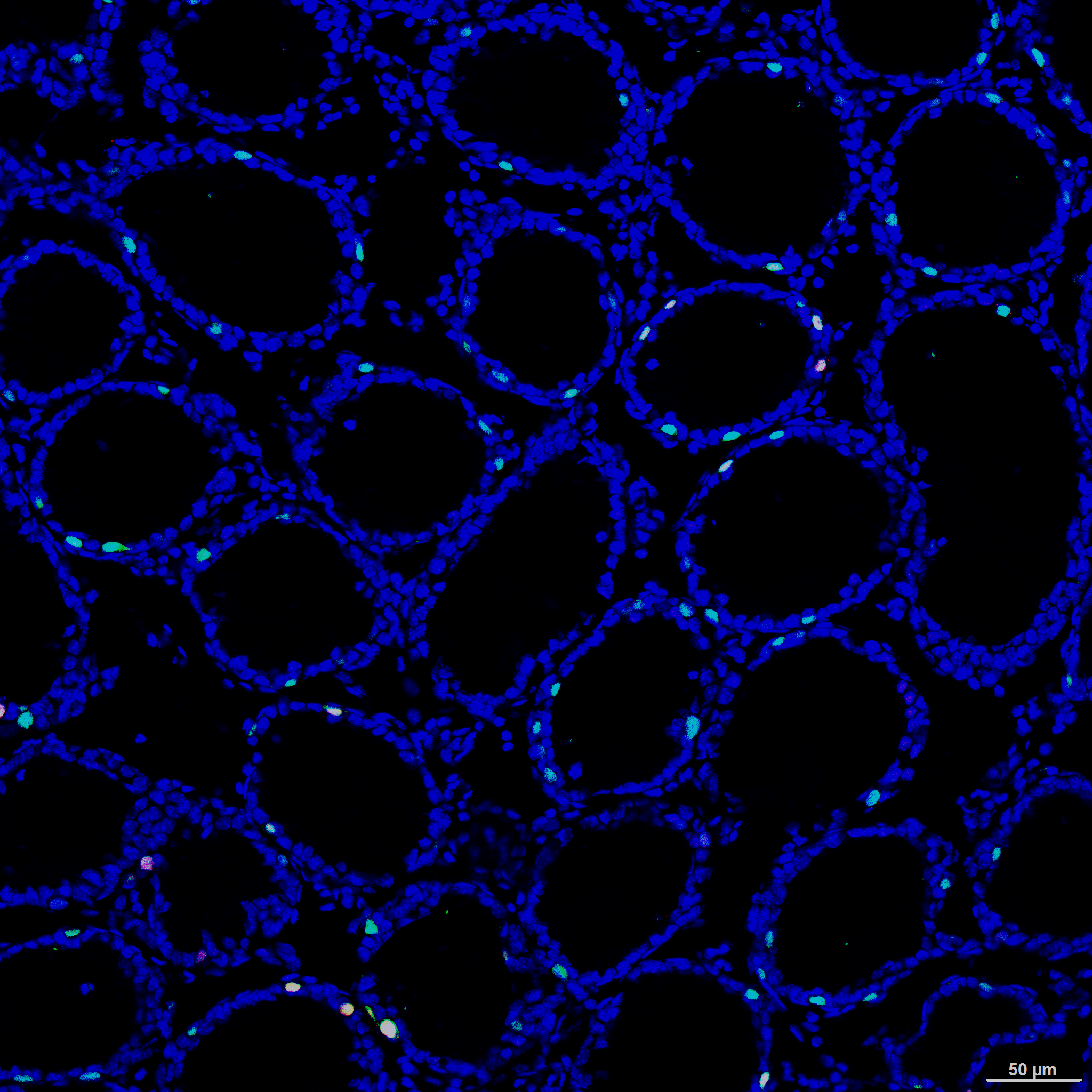

Supplement: Supplementary file 7 — Source data Fig. 4 [file 44319_2025_487_MOESM7_ESM.zip › Figure 4/4E/PD14 Brca1 Vasa-cre testis anti-PLZF&SOX3/PD14 Brca1 vKO testis anti-PLZF&SOX3 Hoechst_overlay.tif]

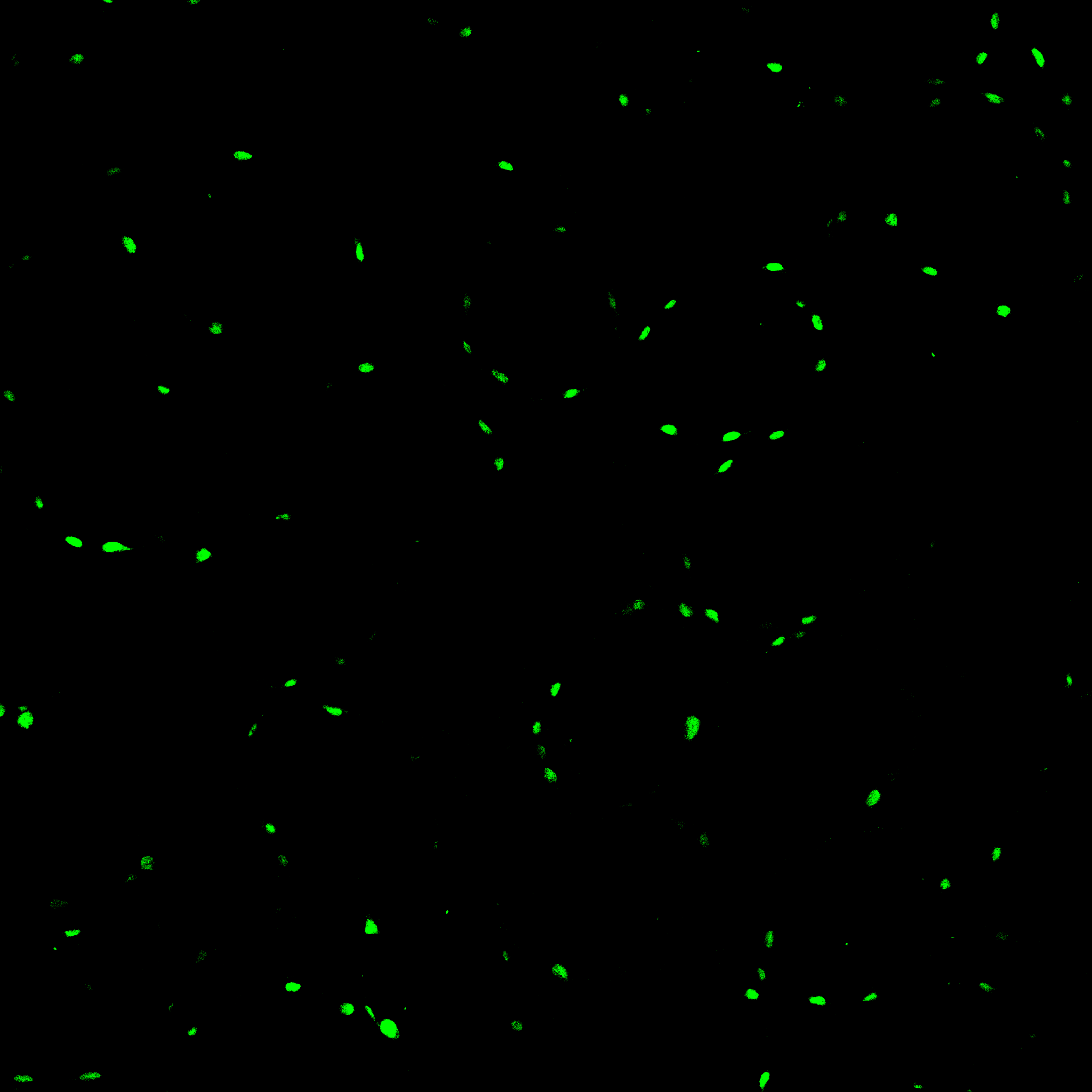

Supplement: Supplementary file 7 — Source data Fig. 4 [file 44319_2025_487_MOESM7_ESM.zip › Figure 4/4E/PD14 Brca1 Vasa-cre testis anti-PLZF&SOX3/PD14 Brca1 vKO testis anti-PLZF.tif]

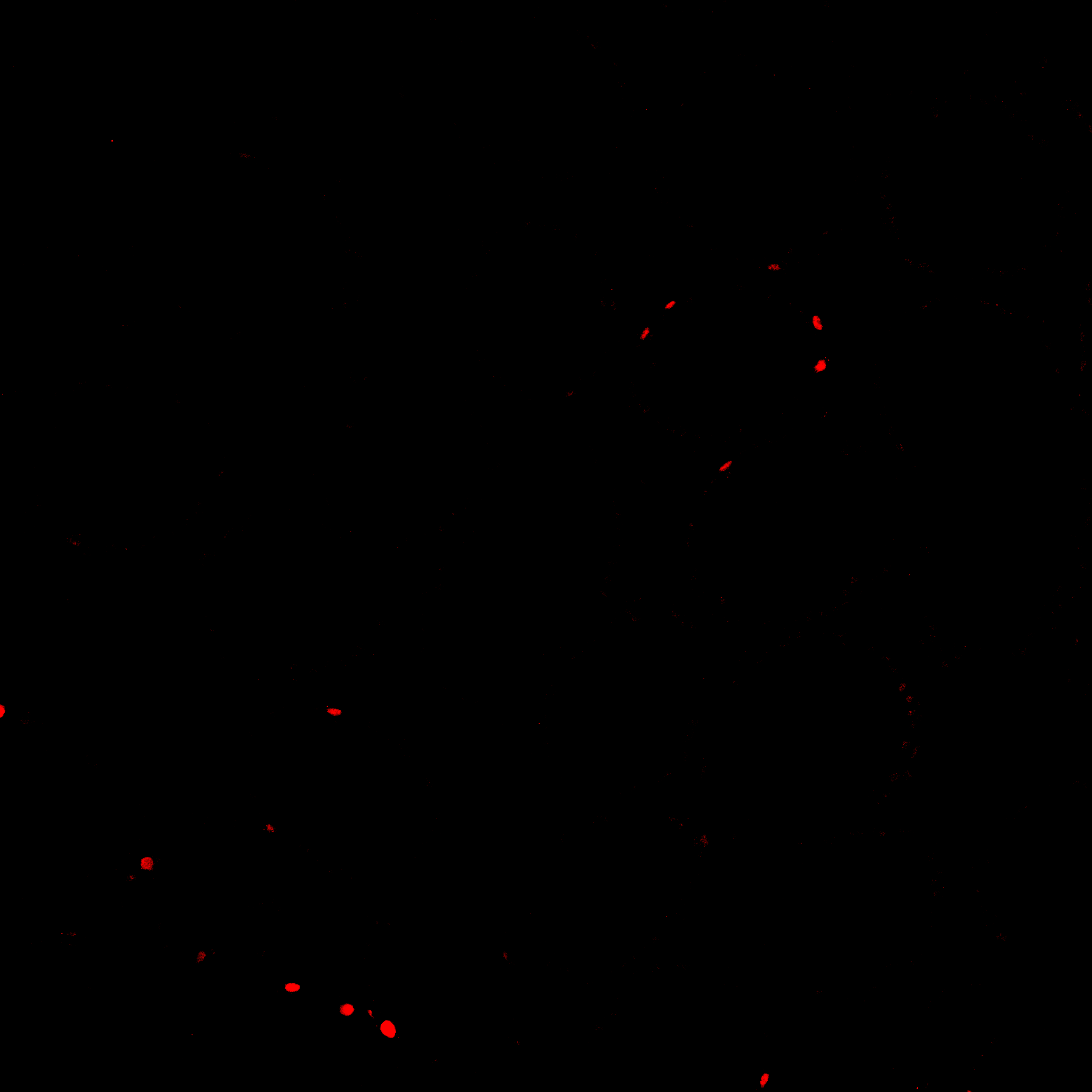

Supplement: Supplementary file 7 — Source data Fig. 4 [file 44319_2025_487_MOESM7_ESM.zip › Figure 4/4E/PD14 Brca1 Vasa-cre testis anti-PLZF&SOX3/PD14 Brca1 vKO testis anti-SOX3.tif]

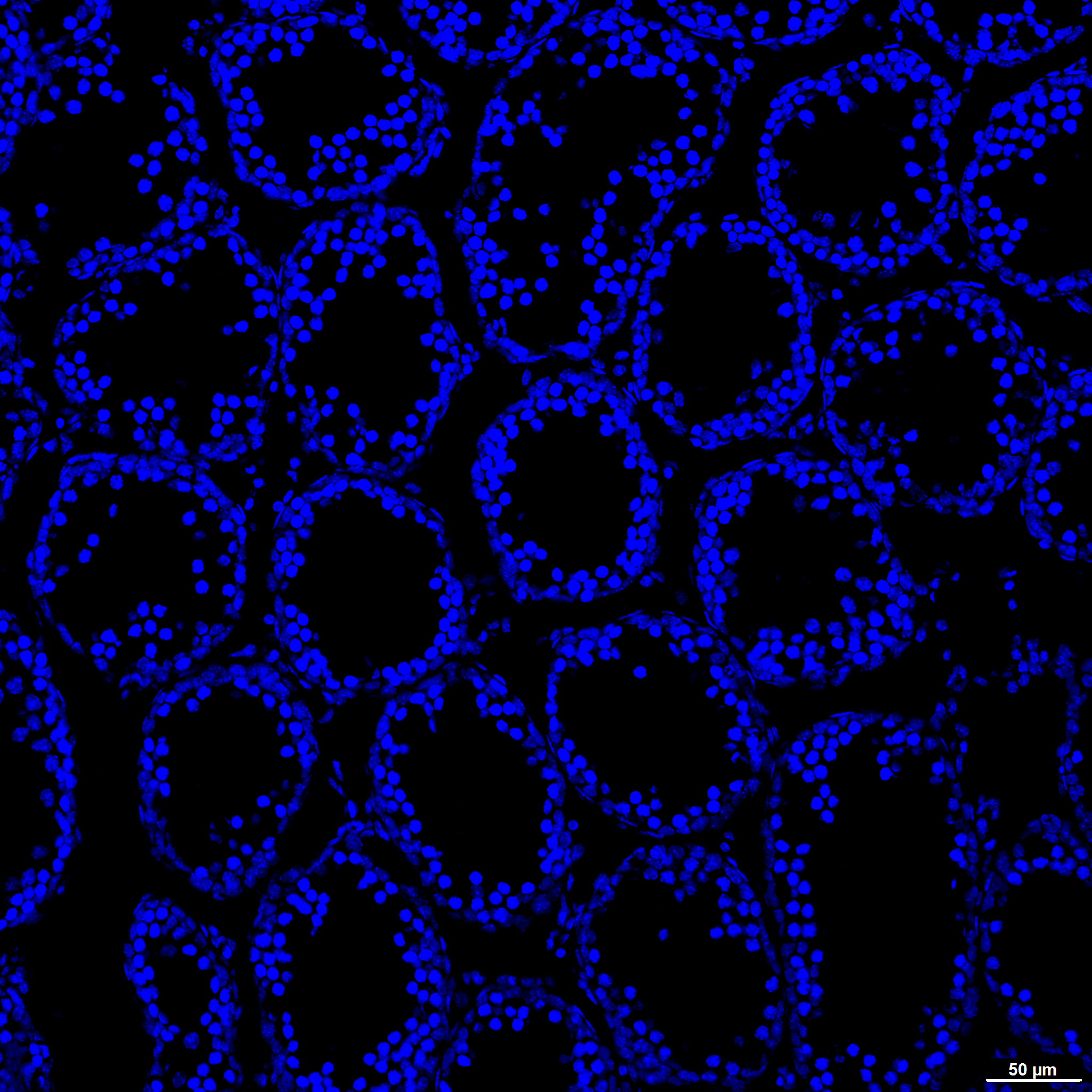

Supplement: Supplementary file 7 — Source data Fig. 4 [file 44319_2025_487_MOESM7_ESM.zip › Figure 4/4E/PD14 Brca1 Vasa-cre testis anti-PLZF&SOX3/PD14 Control testis anti-PLZF&SOX3 Hoechst.tif]

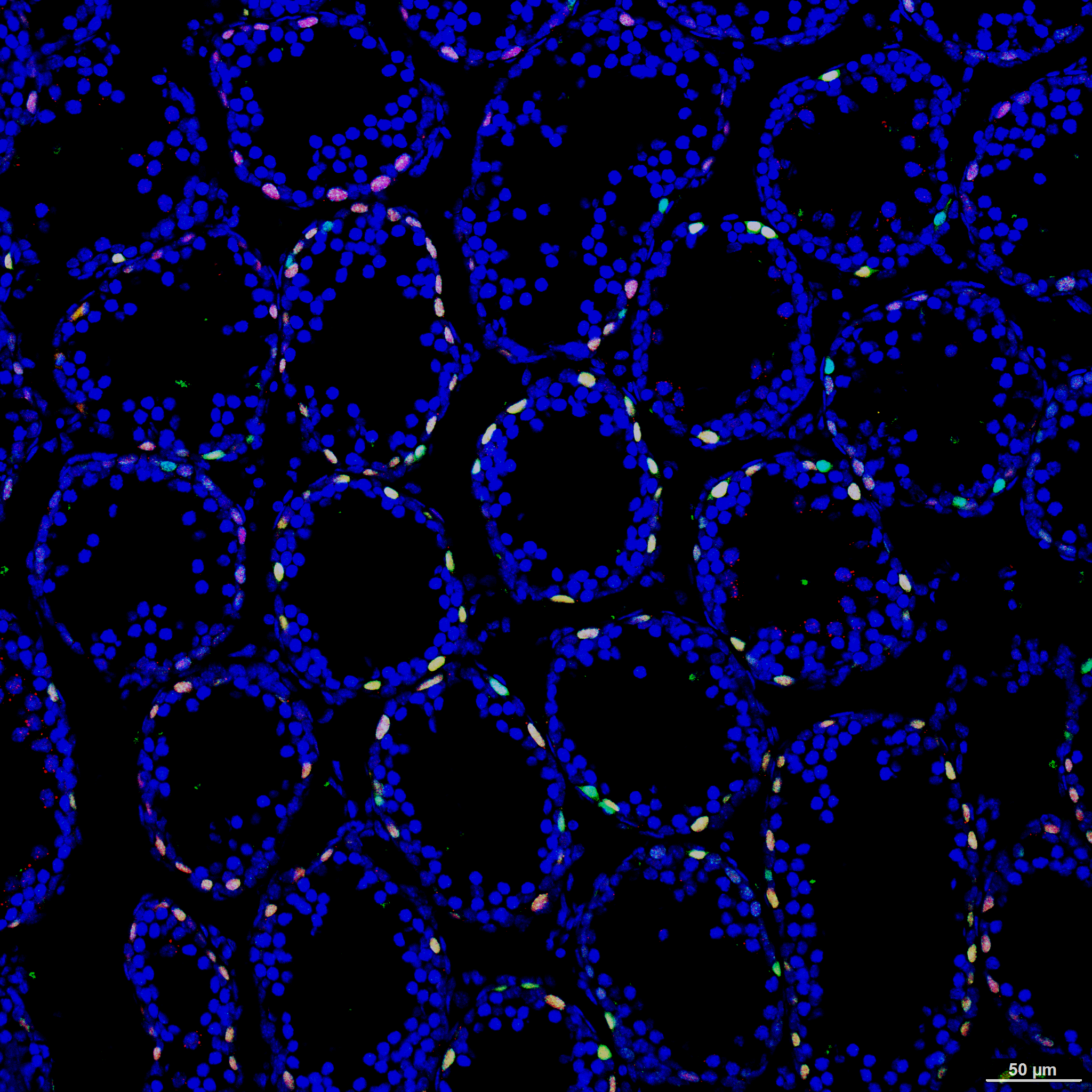

Supplement: Supplementary file 7 — Source data Fig. 4 [file 44319_2025_487_MOESM7_ESM.zip › Figure 4/4E/PD14 Brca1 Vasa-cre testis anti-PLZF&SOX3/PD14 Control testis anti-PLZF&SOX3 Hoechst_overlay.tif]

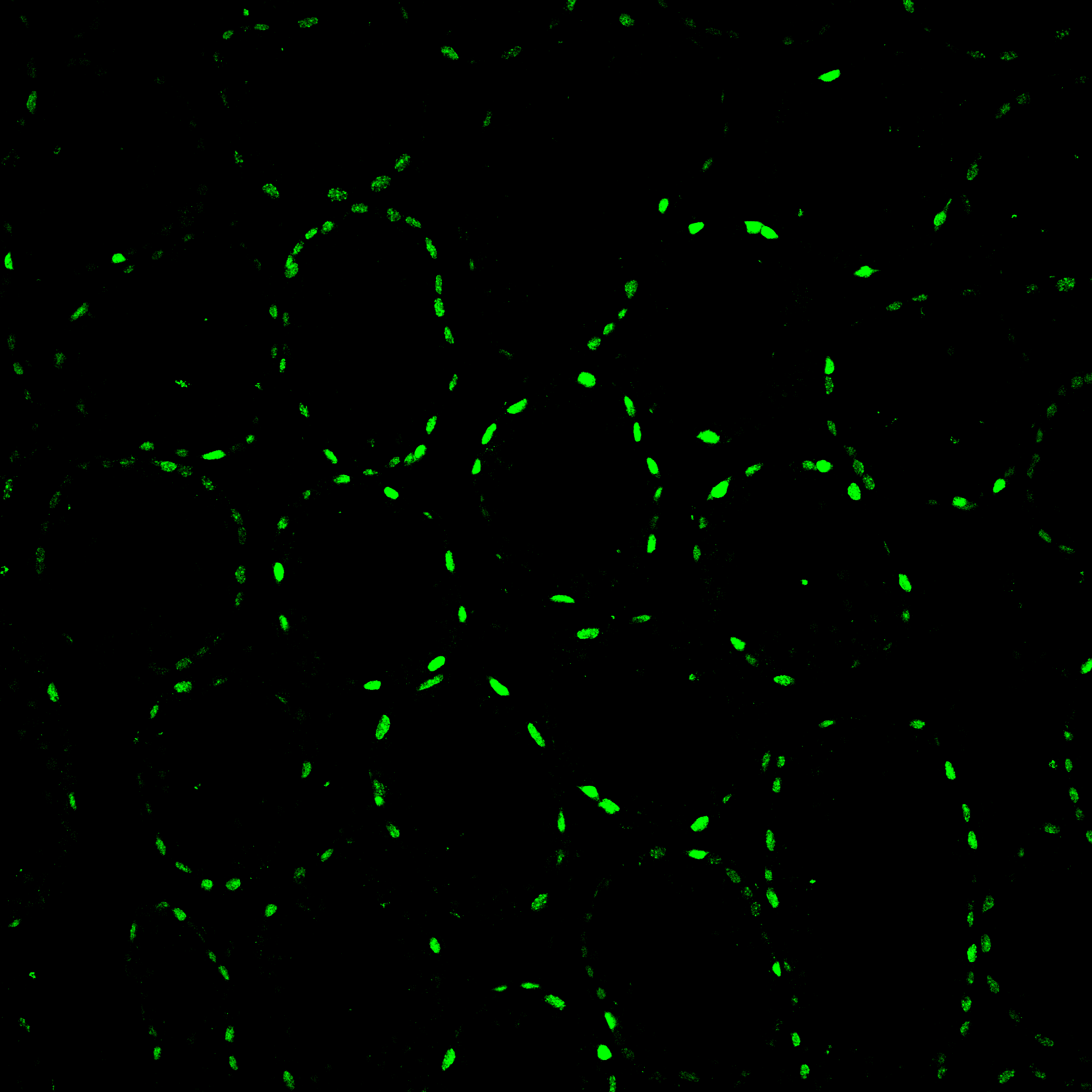

Supplement: Supplementary file 7 — Source data Fig. 4 [file 44319_2025_487_MOESM7_ESM.zip › Figure 4/4E/PD14 Brca1 Vasa-cre testis anti-PLZF&SOX3/PD14 Control testis anti-PLZF.tif]

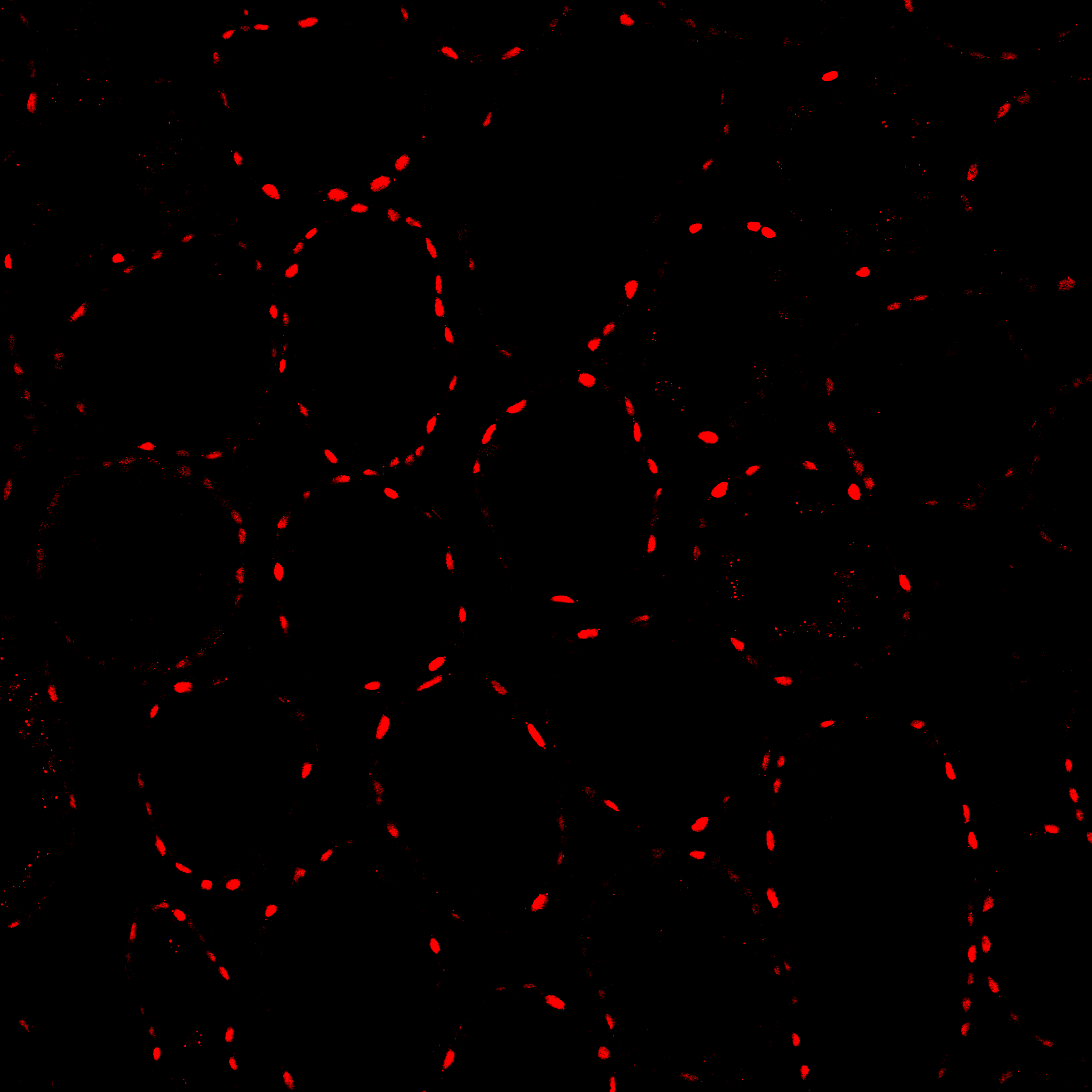

Supplement: Supplementary file 7 — Source data Fig. 4 [file 44319_2025_487_MOESM7_ESM.zip › Figure 4/4E/PD14 Brca1 Vasa-cre testis anti-PLZF&SOX3/PD14 Control testis anti-SOX3.tif]

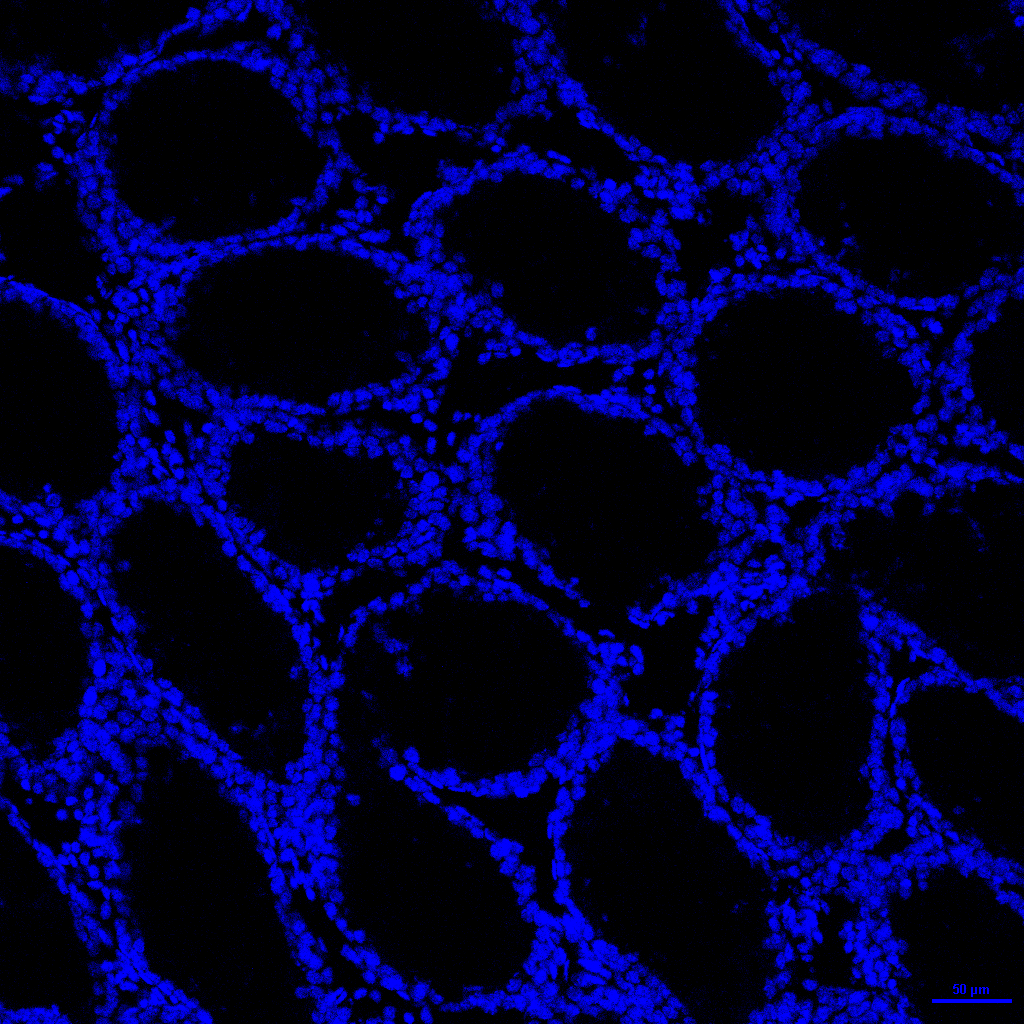

Supplement: Supplementary file 7 — Source data Fig. 4 [file 44319_2025_487_MOESM7_ESM.zip › Figure 4/4E/PD21 Brca1 Vasa-cre testis anti-PLZF&SOX3/PD21 Brca1 vKO testis anti-PLZF&SOX3 Hoechst.tif]

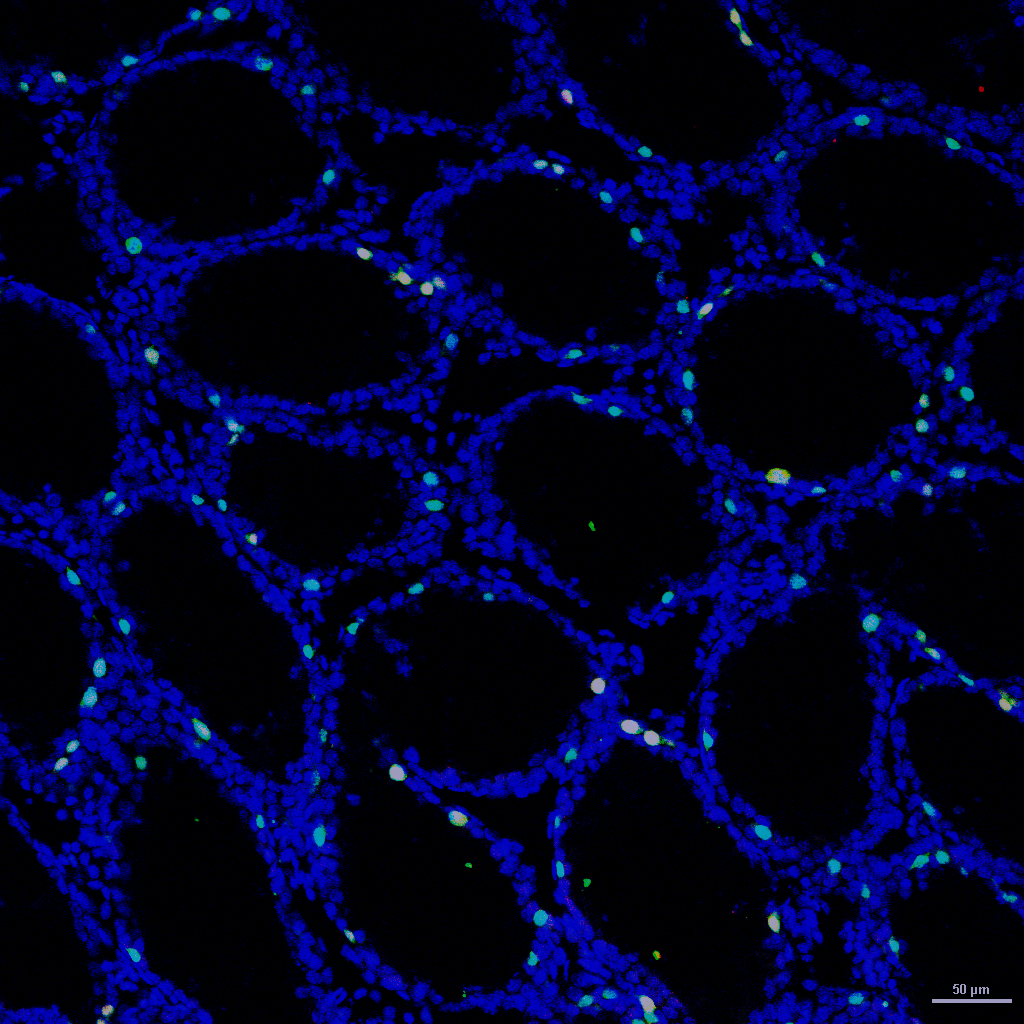

Supplement: Supplementary file 7 — Source data Fig. 4 [file 44319_2025_487_MOESM7_ESM.zip › Figure 4/4E/PD21 Brca1 Vasa-cre testis anti-PLZF&SOX3/PD21 Brca1 vKO testis anti-PLZF&SOX3 Hoechst_overlay.tif]

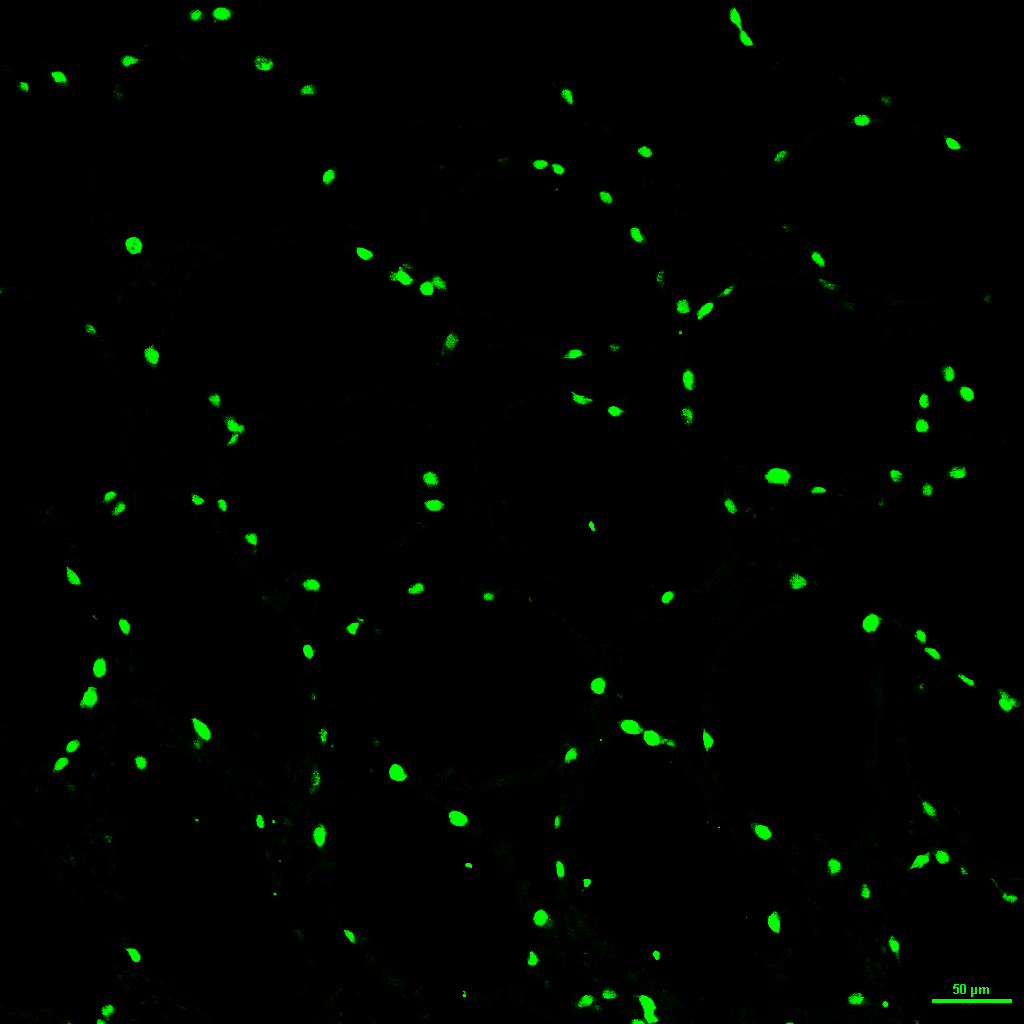

Supplement: Supplementary file 7 — Source data Fig. 4 [file 44319_2025_487_MOESM7_ESM.zip › Figure 4/4E/PD21 Brca1 Vasa-cre testis anti-PLZF&SOX3/PD21 Brca1 vKO testis anti-PLZF.tif]

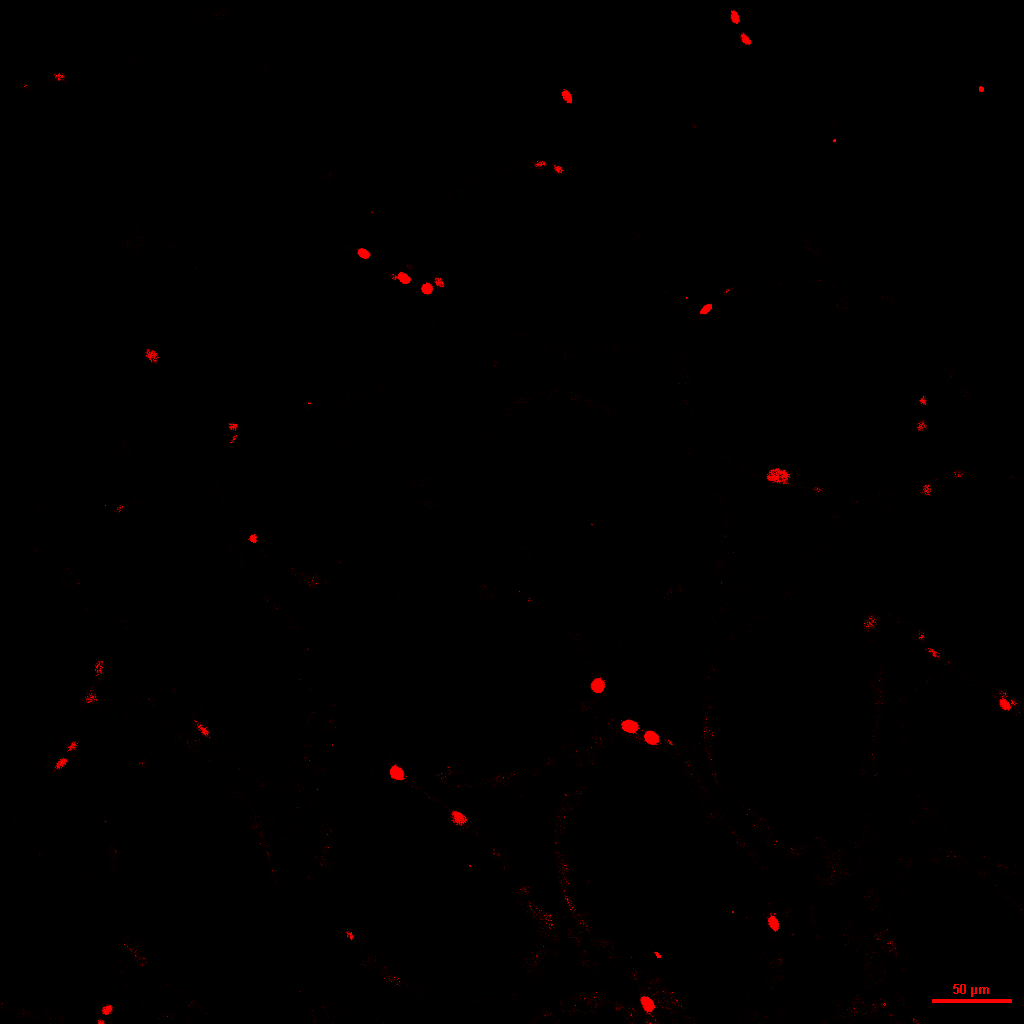

Supplement: Supplementary file 7 — Source data Fig. 4 [file 44319_2025_487_MOESM7_ESM.zip › Figure 4/4E/PD21 Brca1 Vasa-cre testis anti-PLZF&SOX3/PD21 Brca1 vKO testis anti-SOX3.tif]

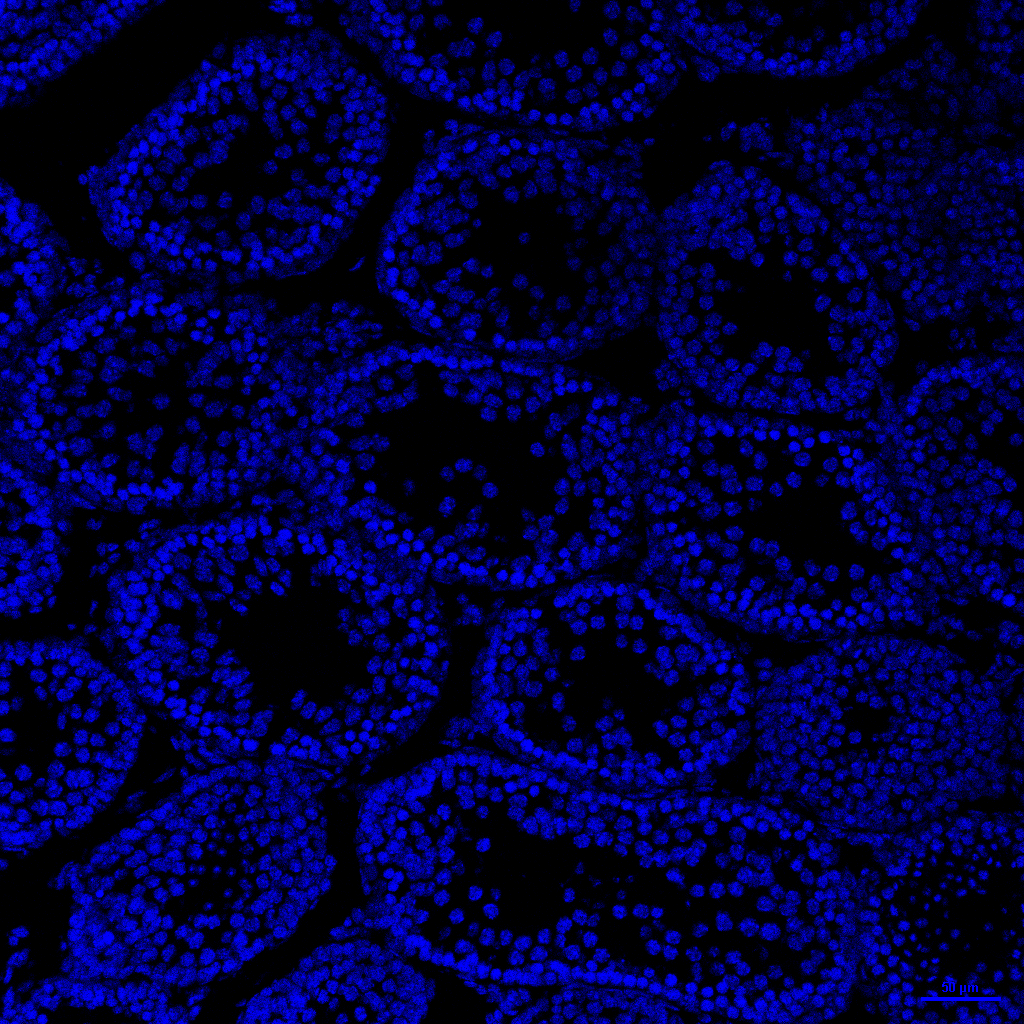

Supplement: Supplementary file 7 — Source data Fig. 4 [file 44319_2025_487_MOESM7_ESM.zip › Figure 4/4E/PD21 Brca1 Vasa-cre testis anti-PLZF&SOX3/PD21 Control testis anti-PLZF&SOX3 Hoechst.tif]

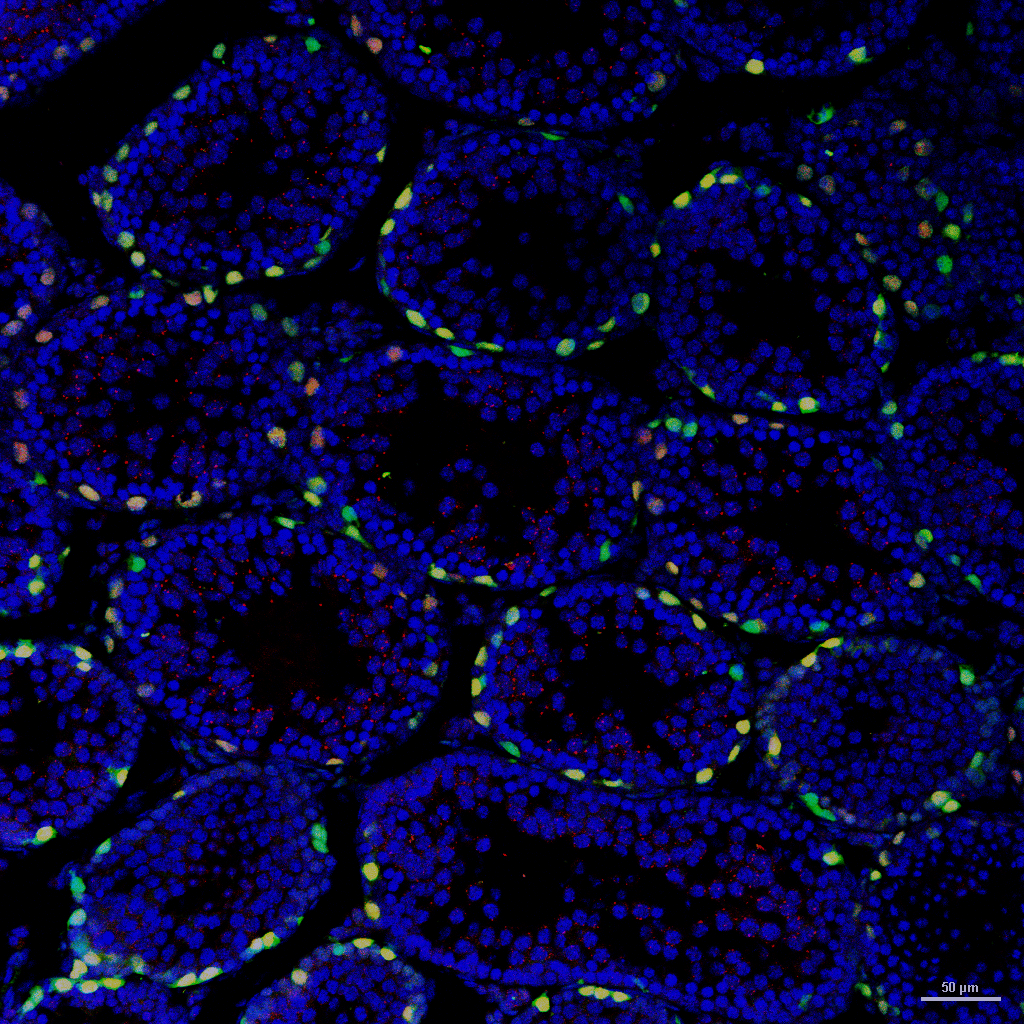

Supplement: Supplementary file 7 — Source data Fig. 4 [file 44319_2025_487_MOESM7_ESM.zip › Figure 4/4E/PD21 Brca1 Vasa-cre testis anti-PLZF&SOX3/PD21 Control testis anti-PLZF&SOX3 Hoechst_overlay.tif]

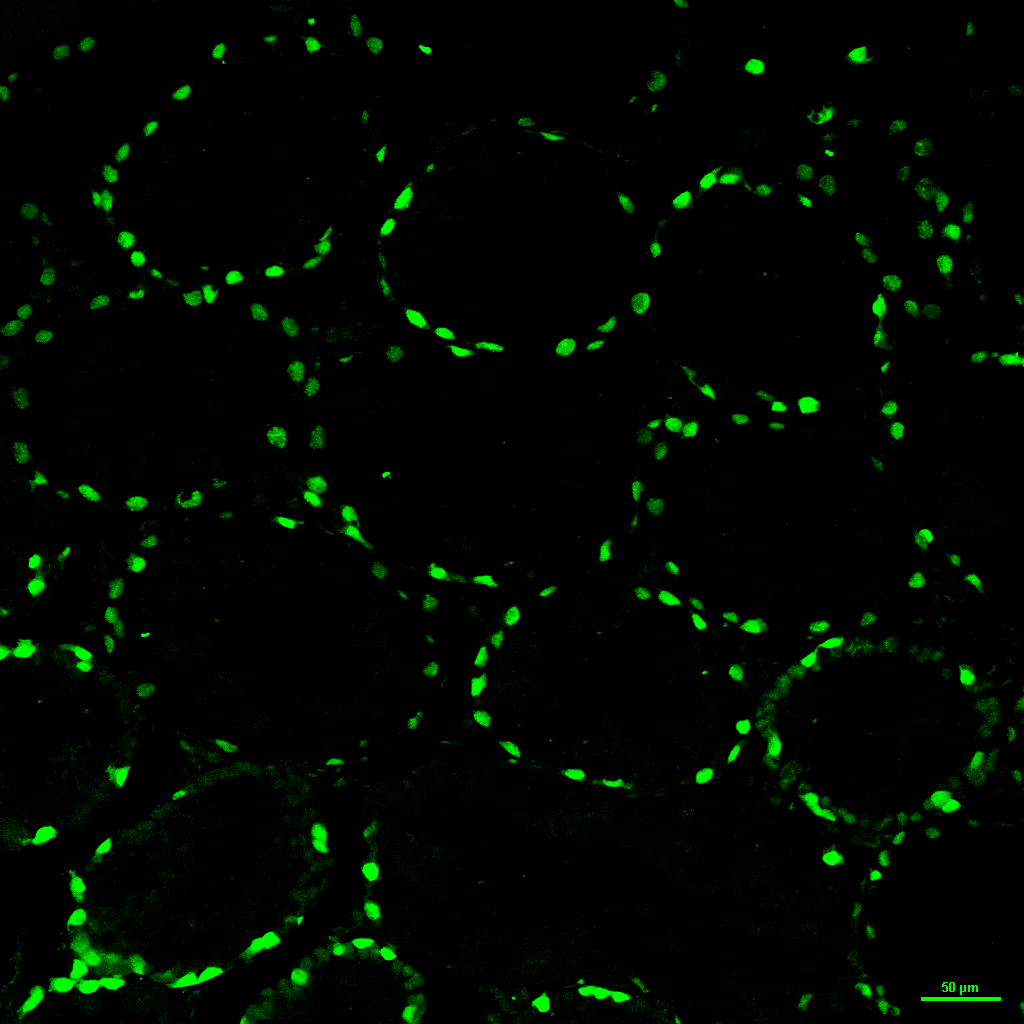

Supplement: Supplementary file 7 — Source data Fig. 4 [file 44319_2025_487_MOESM7_ESM.zip › Figure 4/4E/PD21 Brca1 Vasa-cre testis anti-PLZF&SOX3/PD21 Control testis anti-PLZF.tif]

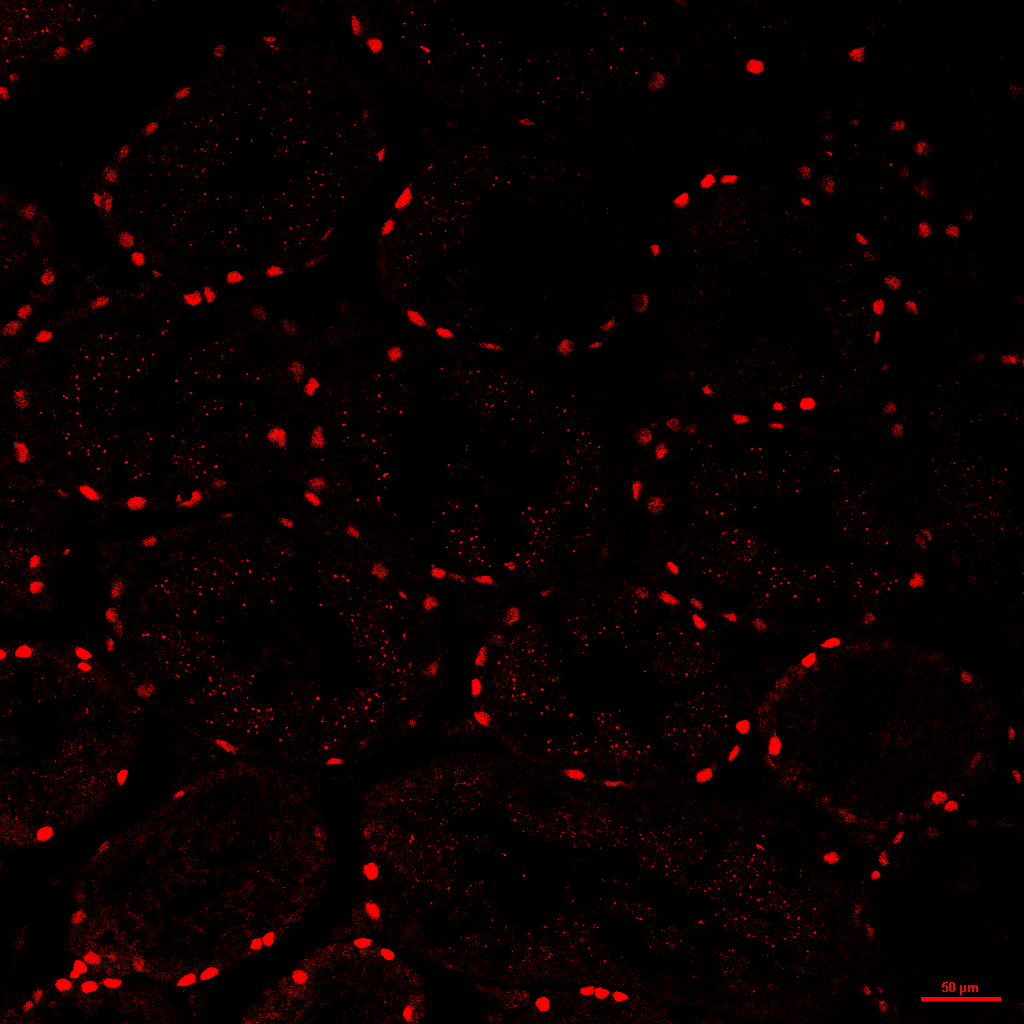

Supplement: Supplementary file 7 — Source data Fig. 4 [file 44319_2025_487_MOESM7_ESM.zip › Figure 4/4E/PD21 Brca1 Vasa-cre testis anti-PLZF&SOX3/PD21 Control testis anti-SOX3.tif]

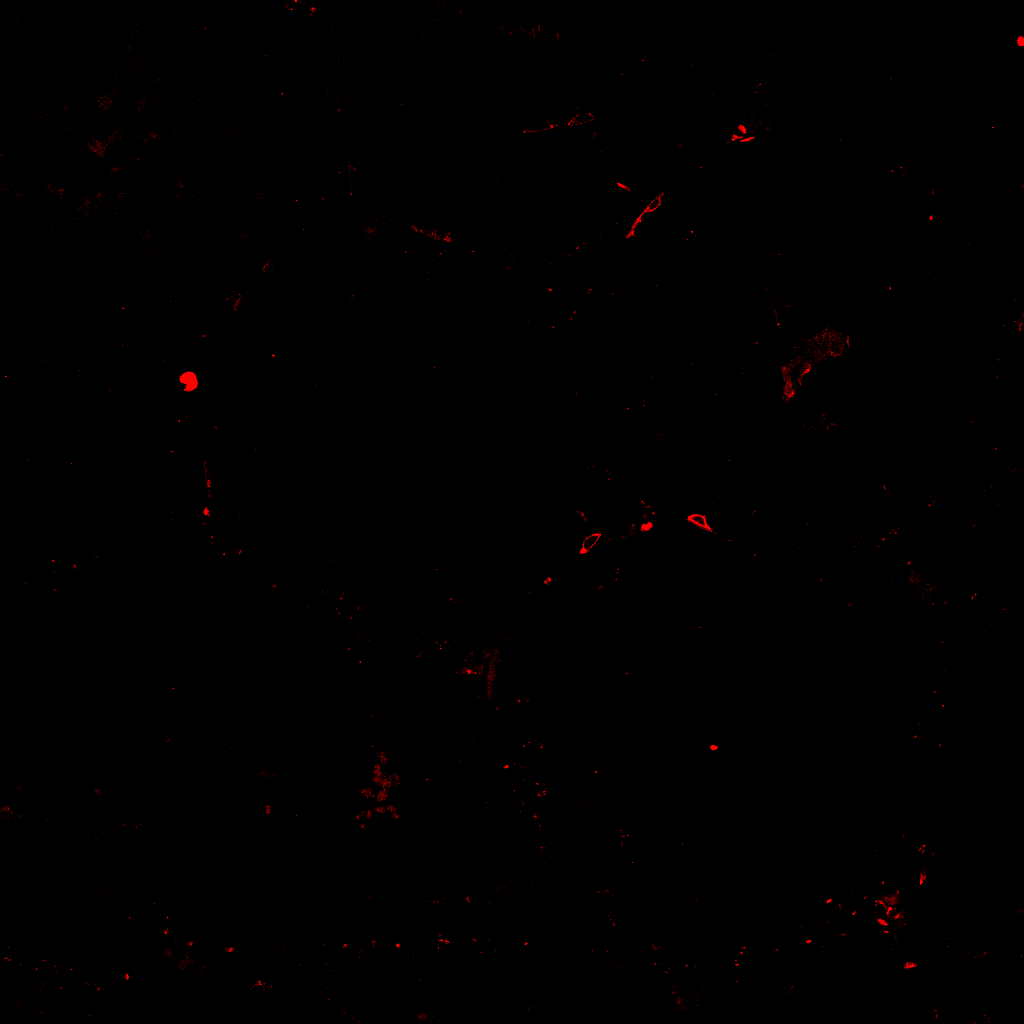

Supplement: Supplementary file 8 — Source data Fig. 5 [file 44319_2025_487_MOESM8_ESM.zip › Figure 5/5A/PD180 PLZF&GFRa1/PD180 Control testis anti-GFRa1.tif]

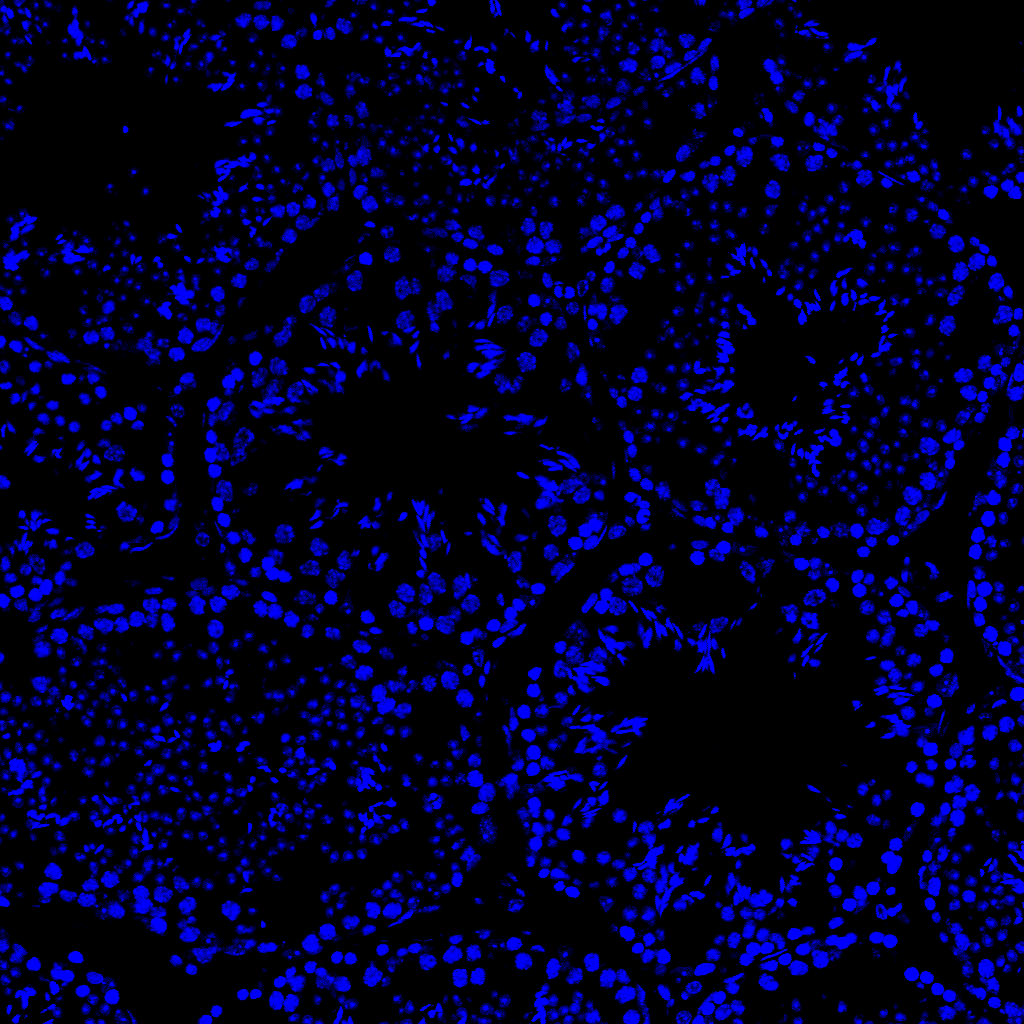

Supplement: Supplementary file 8 — Source data Fig. 5 [file 44319_2025_487_MOESM8_ESM.zip › Figure 5/5A/PD180 PLZF&GFRa1/PD180 Control testis anti-PLZF&GFRa1 Hoechst.tif]

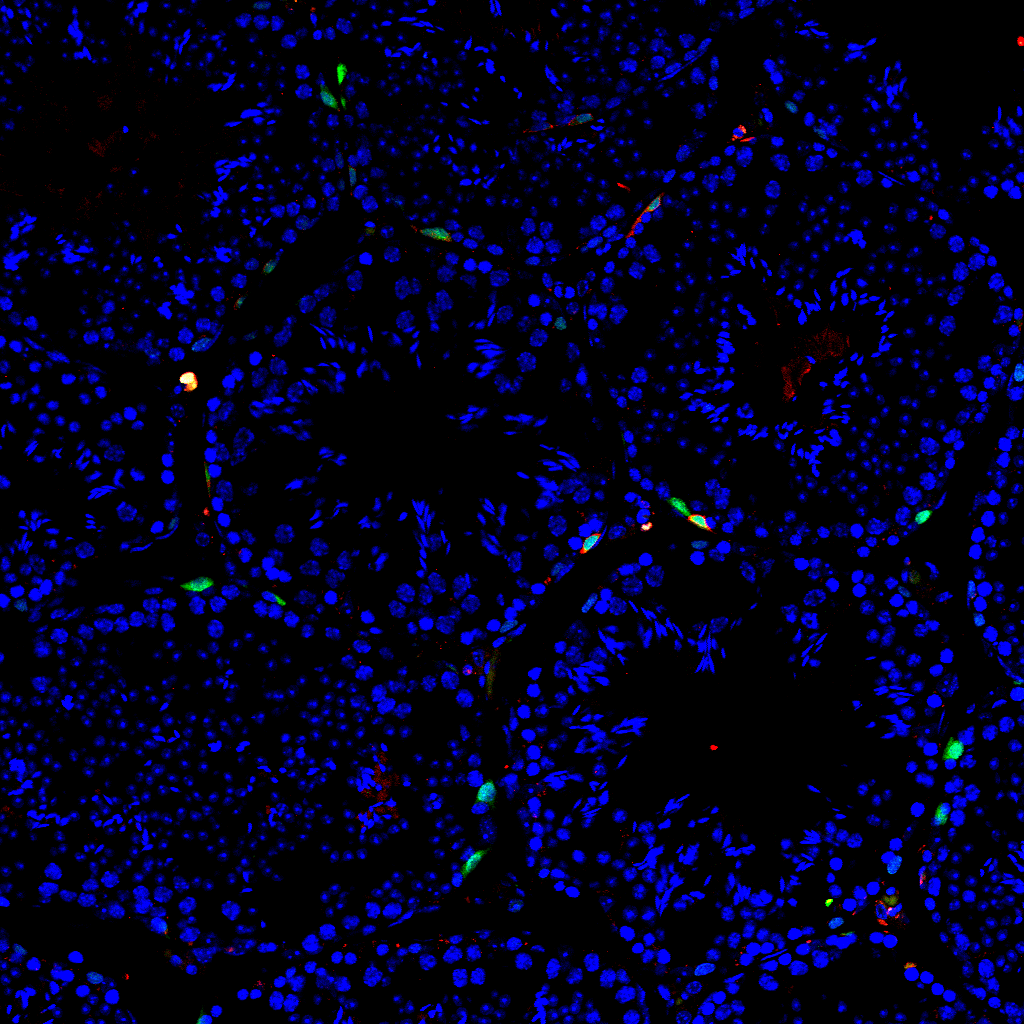

Supplement: Supplementary file 8 — Source data Fig. 5 [file 44319_2025_487_MOESM8_ESM.zip › Figure 5/5A/PD180 PLZF&GFRa1/PD180 Control testis anti-PLZF&GFRa1_overlay.tif]

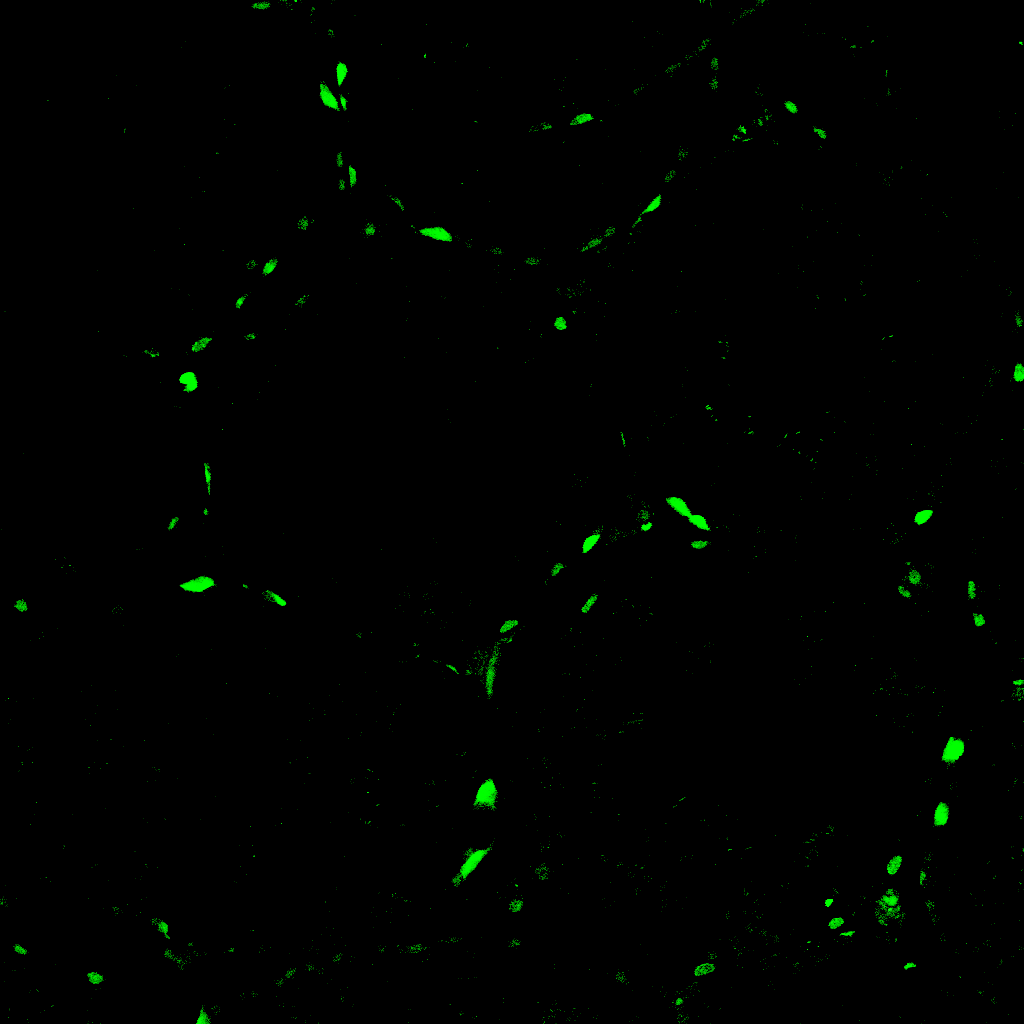

Supplement: Supplementary file 8 — Source data Fig. 5 [file 44319_2025_487_MOESM8_ESM.zip › Figure 5/5A/PD180 PLZF&GFRa1/PD180 Control testis anti-PLZF.tif]

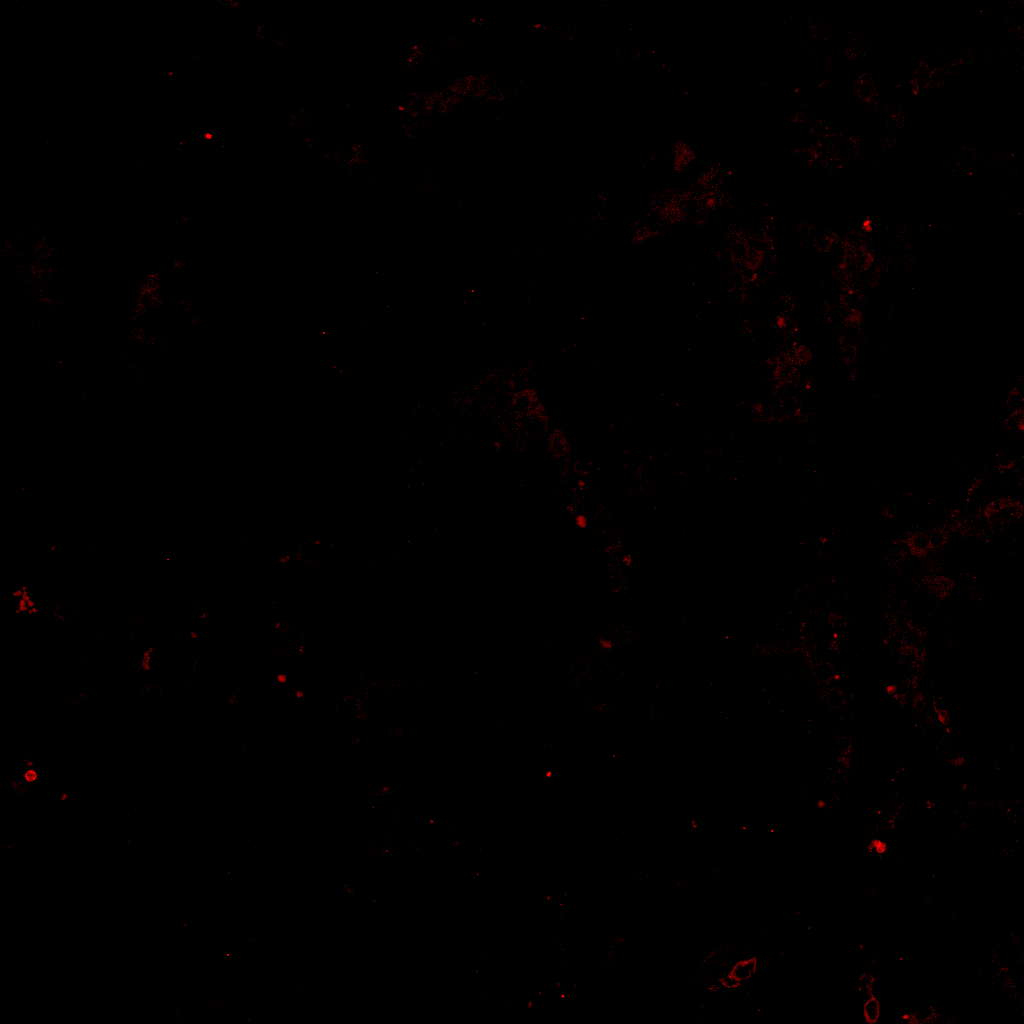

Supplement: Supplementary file 8 — Source data Fig. 5 [file 44319_2025_487_MOESM8_ESM.zip › Figure 5/5A/PD180 PLZF&GFRa1/PND180 Brca1 vKO testis anti-GFRa1.tif]

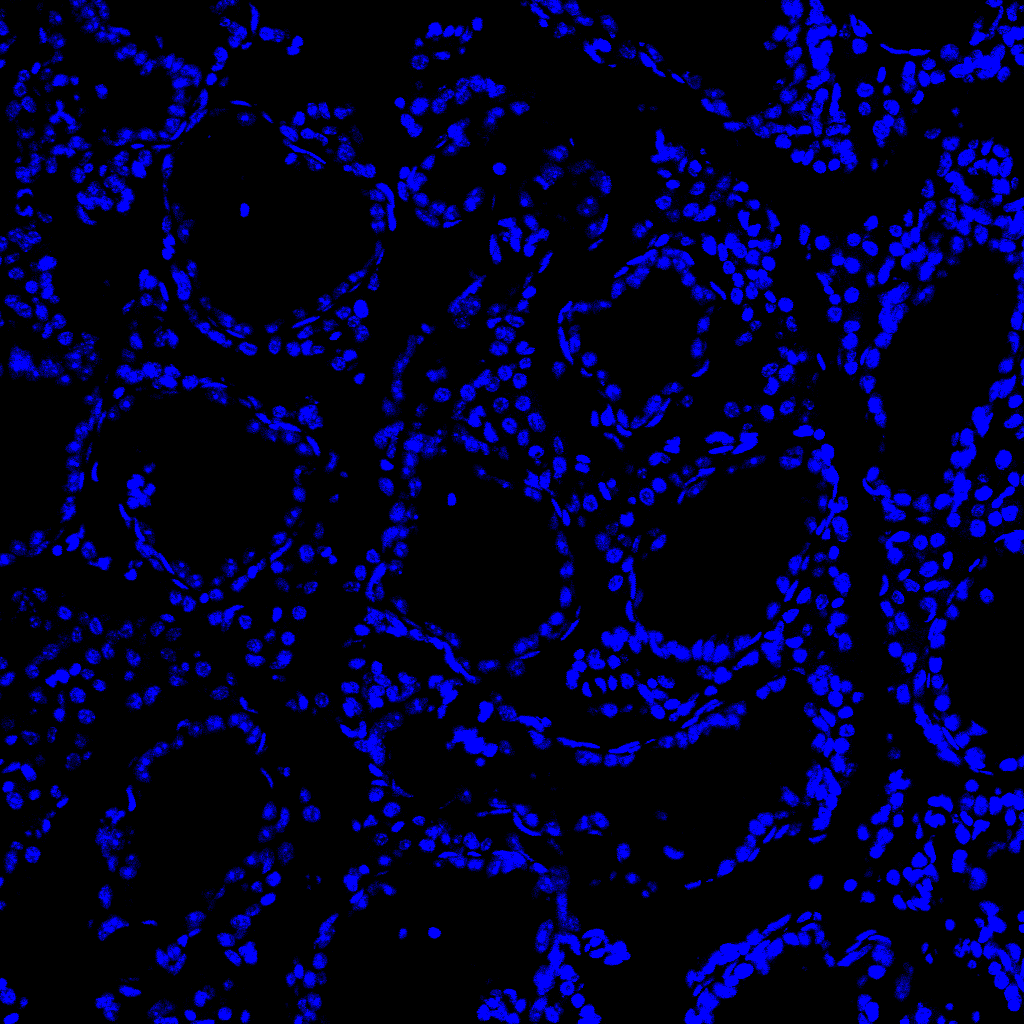

Supplement: Supplementary file 8 — Source data Fig. 5 [file 44319_2025_487_MOESM8_ESM.zip › Figure 5/5A/PD180 PLZF&GFRa1/PND180 Brca1 vKO testis anti-PLZF&GFRa1 Hoechst.tif]

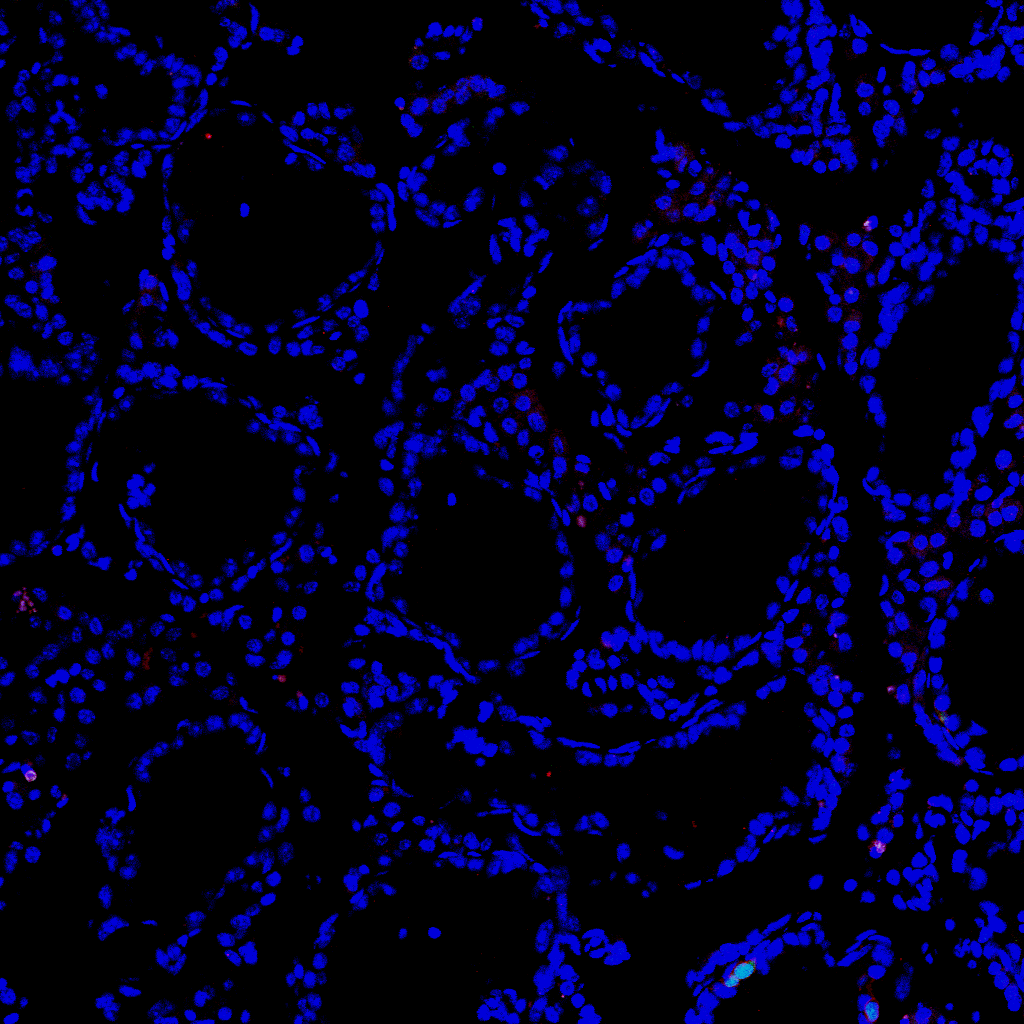

Supplement: Supplementary file 8 — Source data Fig. 5 [file 44319_2025_487_MOESM8_ESM.zip › Figure 5/5A/PD180 PLZF&GFRa1/PND180 Brca1 vKO testis anti-PLZF&GFRa1 Hoechst_overlay.tif]

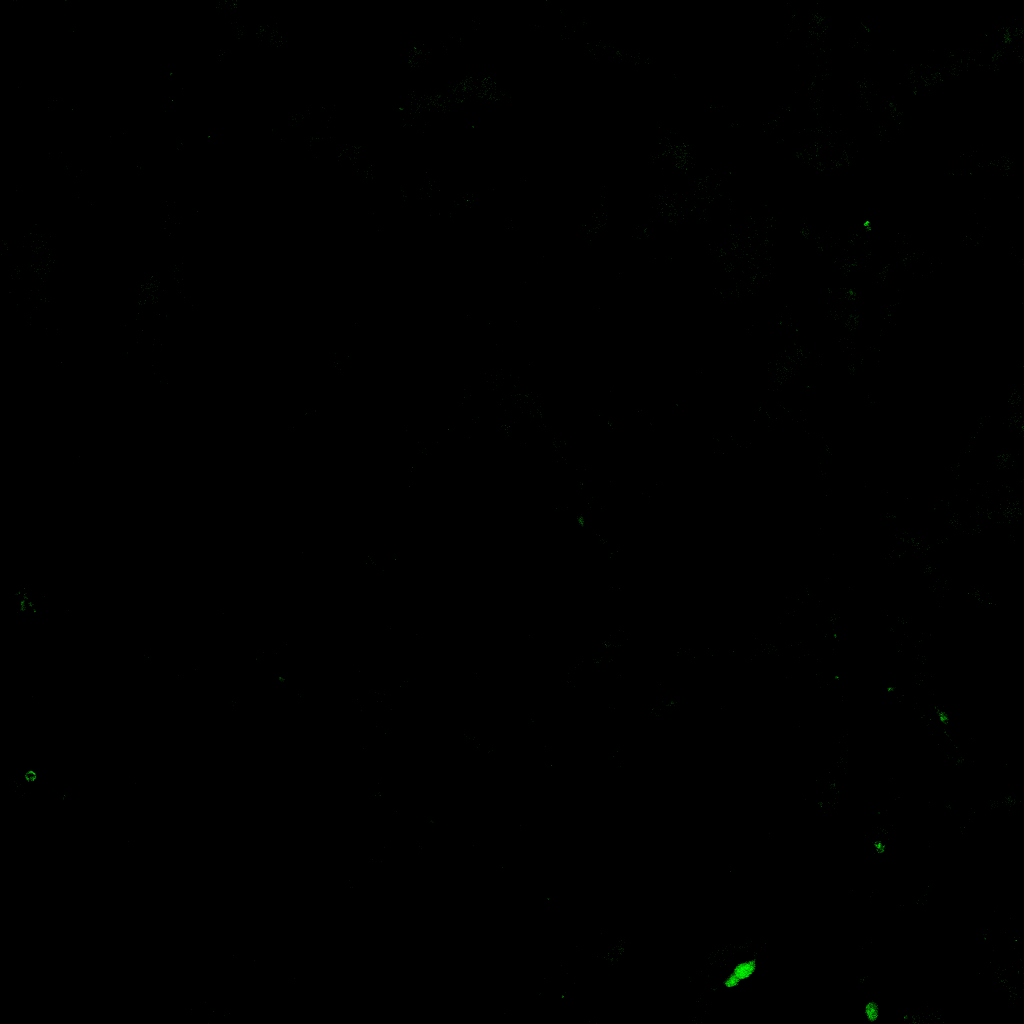

Supplement: Supplementary file 8 — Source data Fig. 5 [file 44319_2025_487_MOESM8_ESM.zip › Figure 5/5A/PD180 PLZF&GFRa1/PND180 Brca1 vKO testis anti-PLZF.tif]

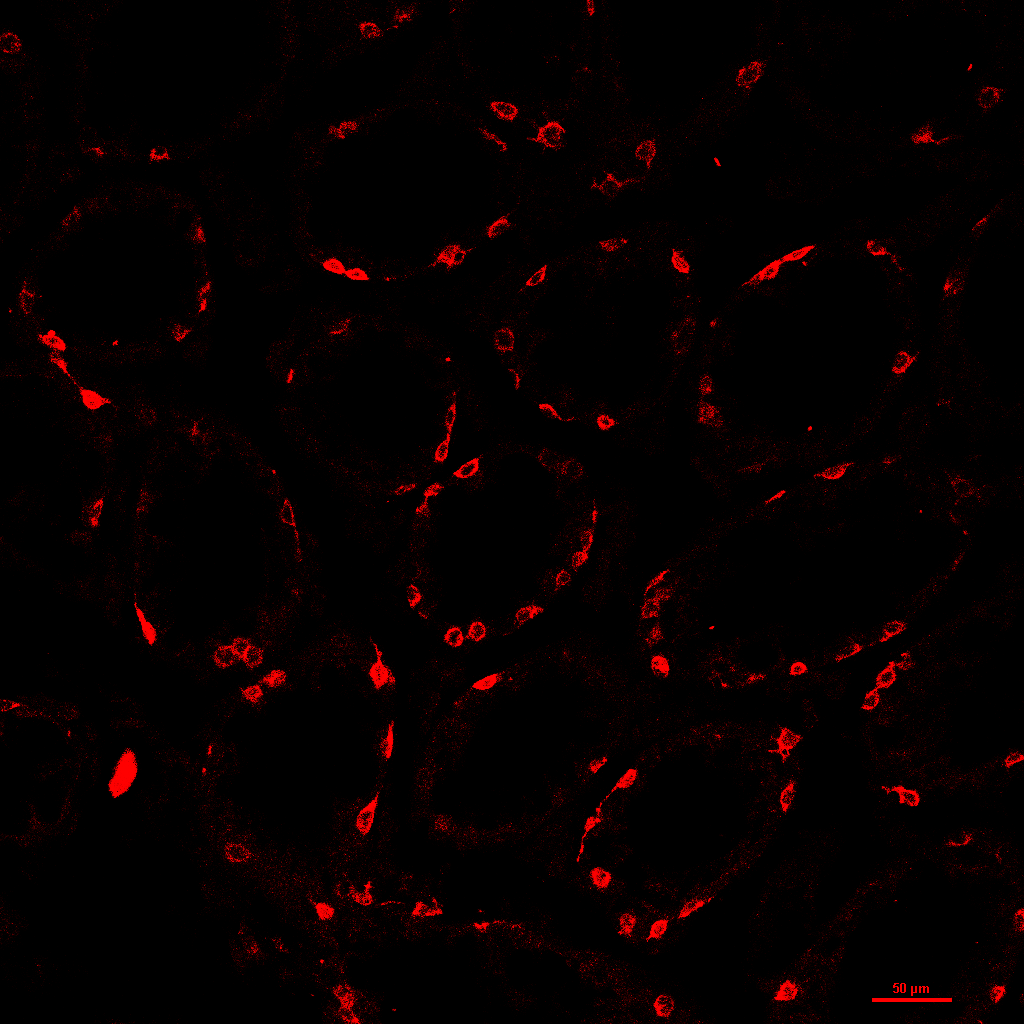

Supplement: Supplementary file 8 — Source data Fig. 5 [file 44319_2025_487_MOESM8_ESM.zip › Figure 5/5A/PD21 PLZF&GFRa1/PD21 Brca1 vKO testis anti-GFRa1.tif]

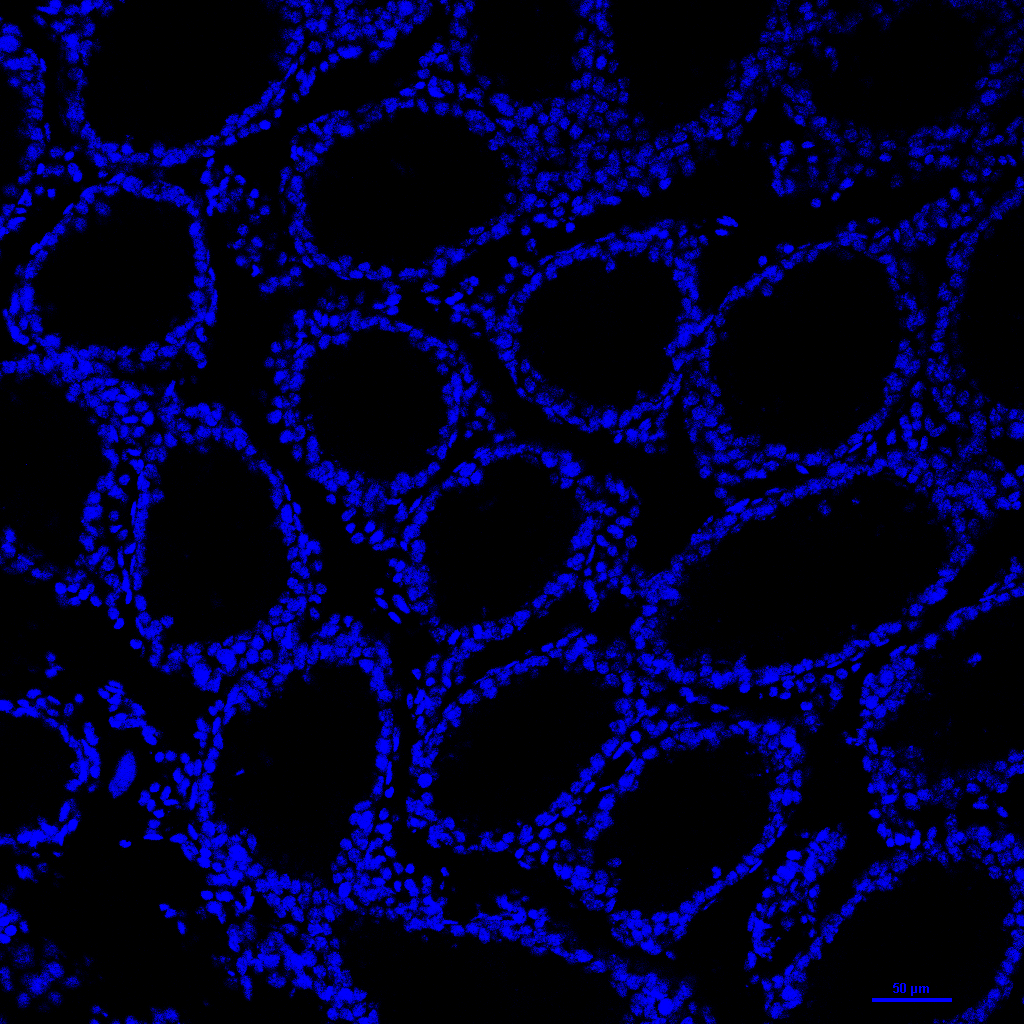

Supplement: Supplementary file 8 — Source data Fig. 5 [file 44319_2025_487_MOESM8_ESM.zip › Figure 5/5A/PD21 PLZF&GFRa1/PD21 Brca1 vKO testis anti-PLZF&GFRa1 Hoechst.tif]

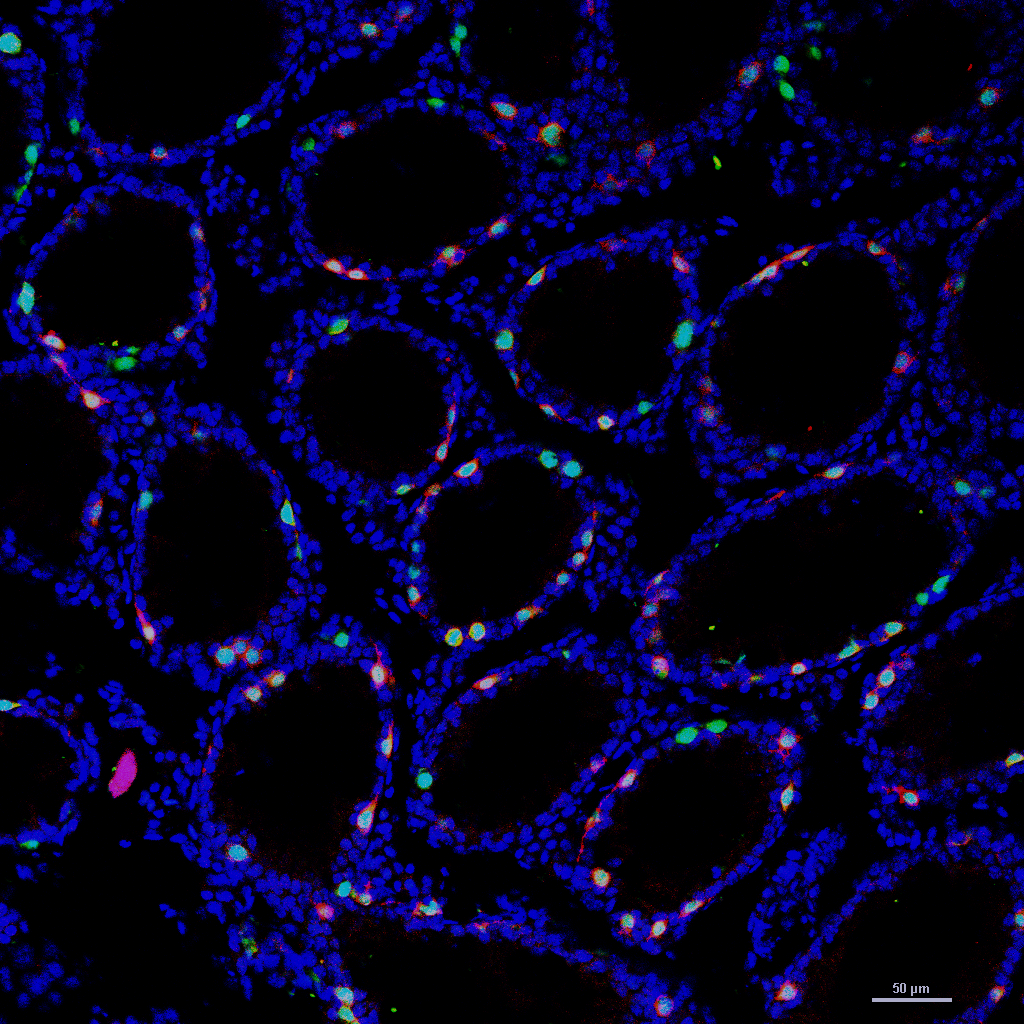

Supplement: Supplementary file 8 — Source data Fig. 5 [file 44319_2025_487_MOESM8_ESM.zip › Figure 5/5A/PD21 PLZF&GFRa1/PD21 Brca1 vKO testis anti-PLZF&GFRa1 Hoechst_overlay.tif]

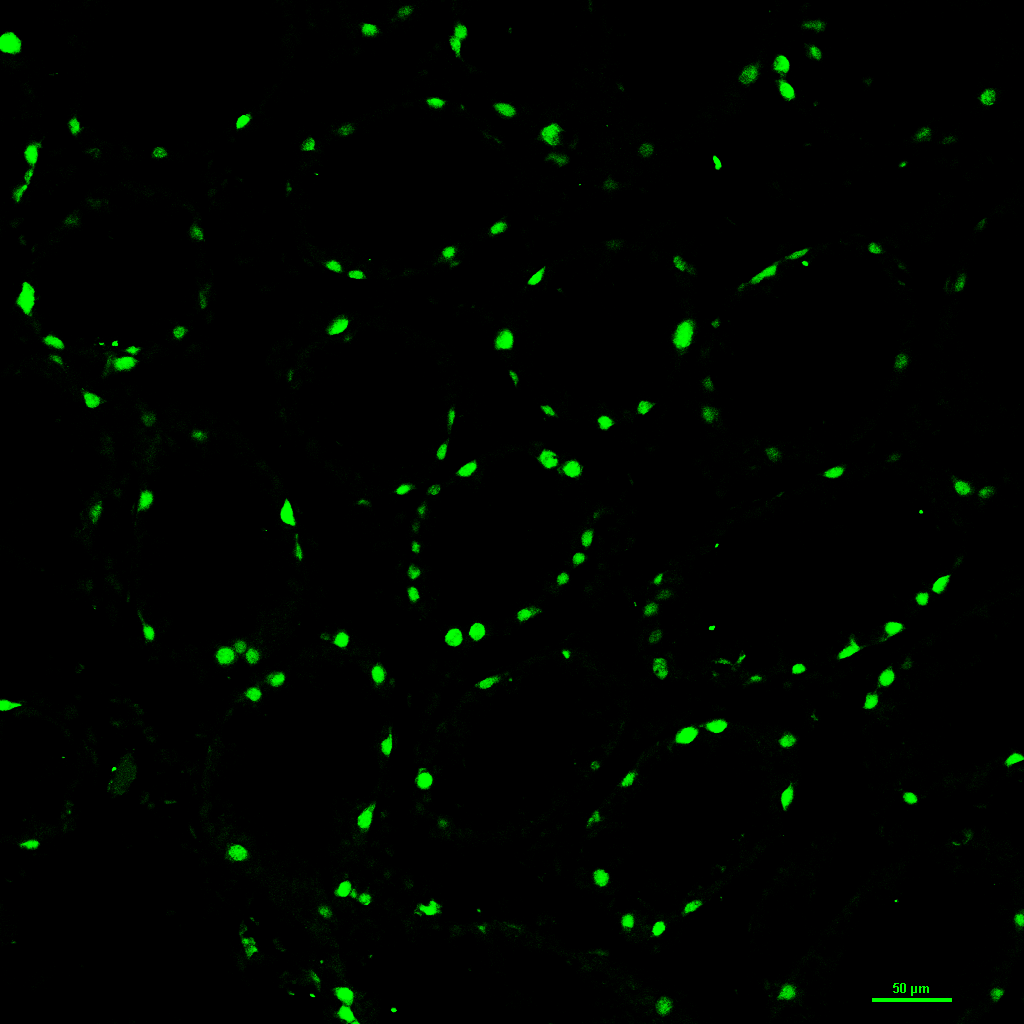

Supplement: Supplementary file 8 — Source data Fig. 5 [file 44319_2025_487_MOESM8_ESM.zip › Figure 5/5A/PD21 PLZF&GFRa1/PD21 Brca1 vKO testis anti-PLZF.tif]

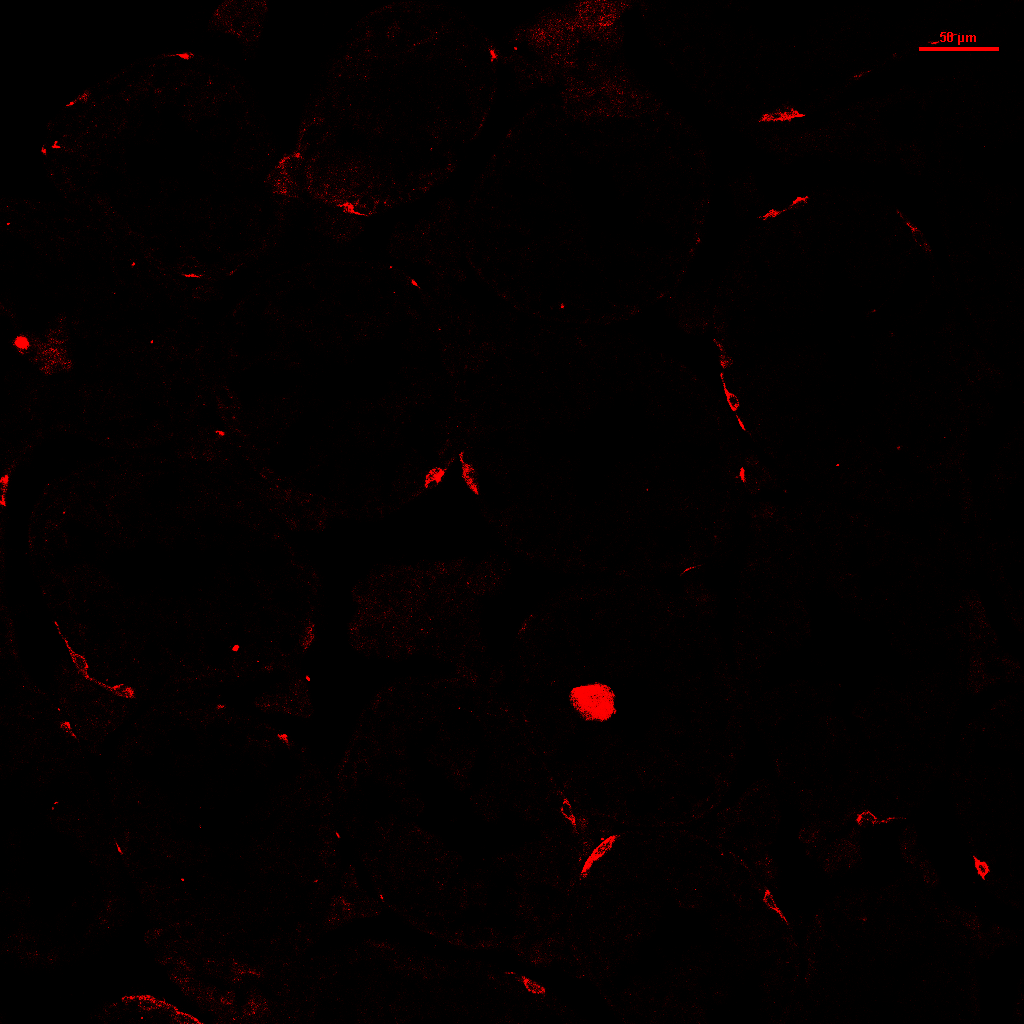

Supplement: Supplementary file 8 — Source data Fig. 5 [file 44319_2025_487_MOESM8_ESM.zip › Figure 5/5A/PD21 PLZF&GFRa1/PD21 Control testis anti-GFRa1.tif]

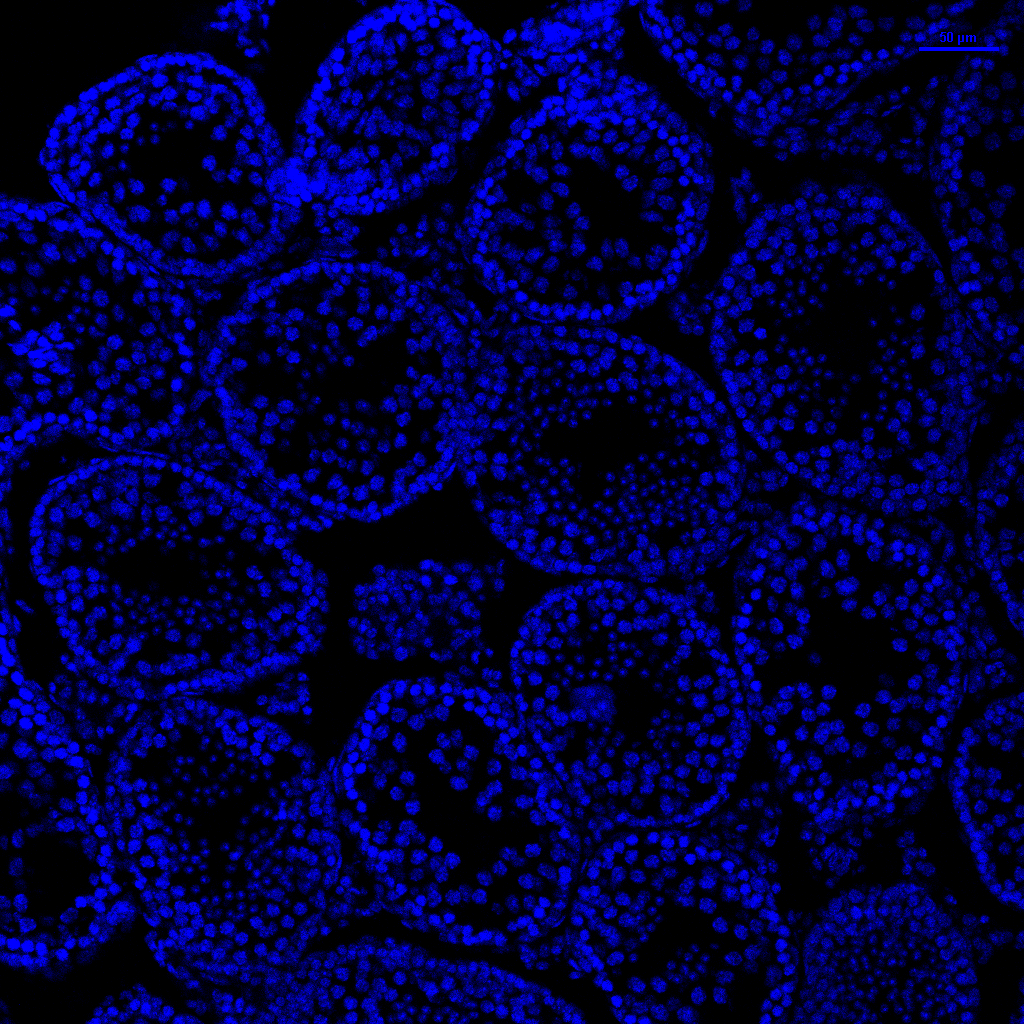

Supplement: Supplementary file 8 — Source data Fig. 5 [file 44319_2025_487_MOESM8_ESM.zip › Figure 5/5A/PD21 PLZF&GFRa1/PD21 Control testis anti-PLZF&GFRa1 Hoechst.tif]

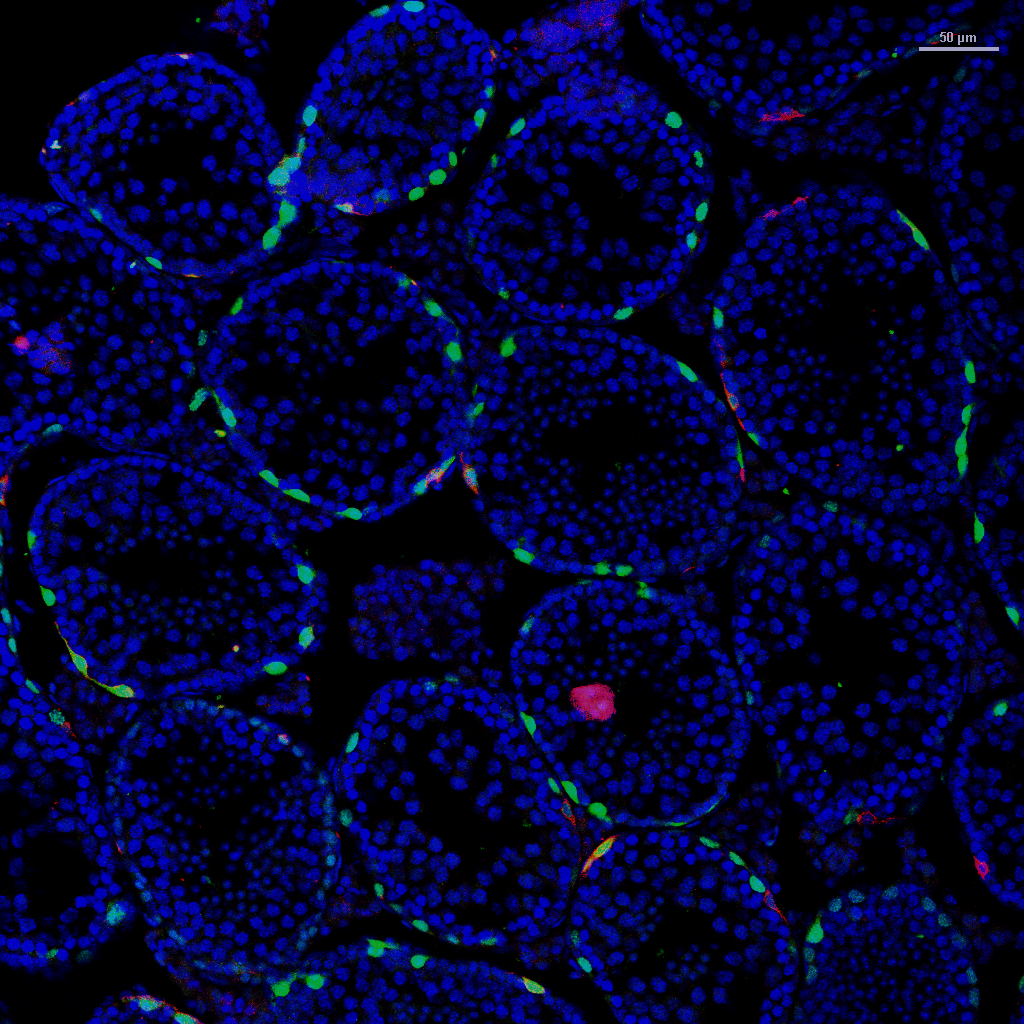

Supplement: Supplementary file 8 — Source data Fig. 5 [file 44319_2025_487_MOESM8_ESM.zip › Figure 5/5A/PD21 PLZF&GFRa1/PD21 Control testis anti-PLZF&GFRa1 Hoechst_overlay.tif]

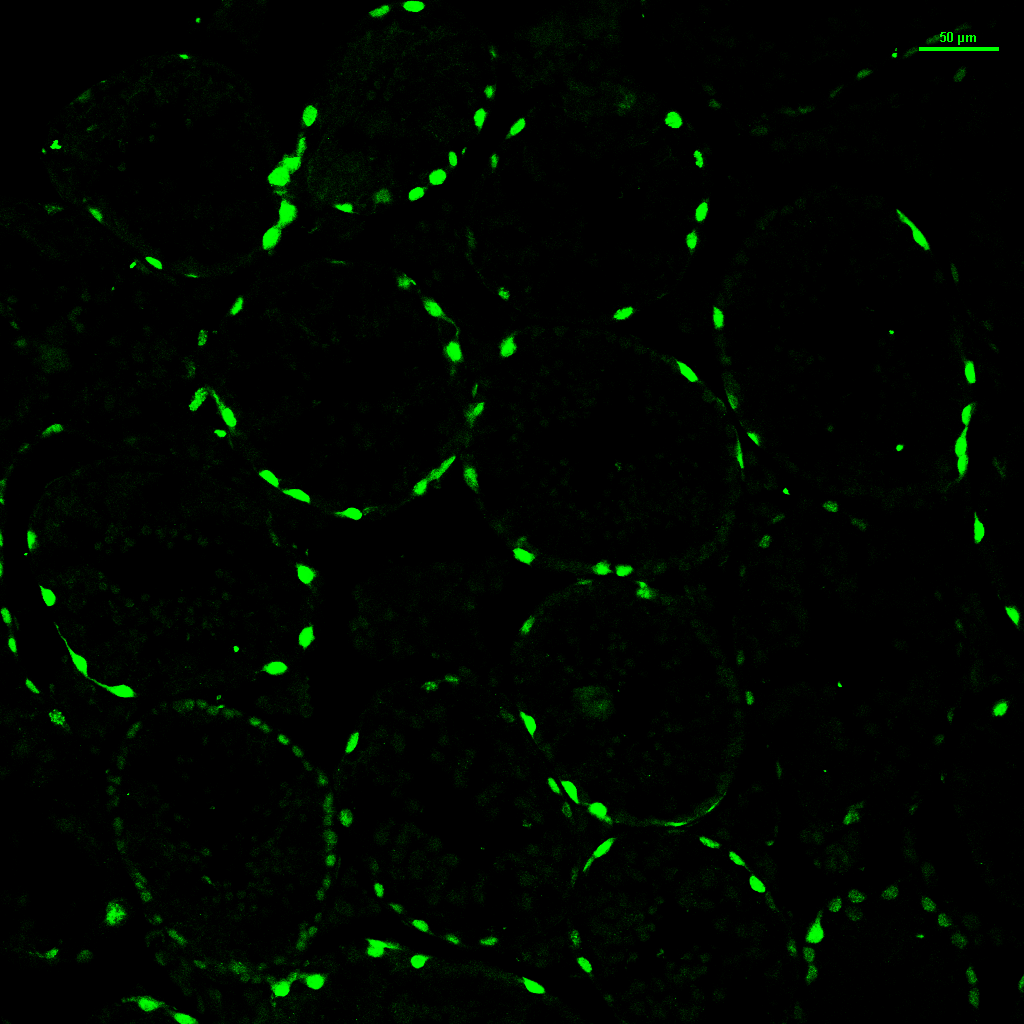

Supplement: Supplementary file 8 — Source data Fig. 5 [file 44319_2025_487_MOESM8_ESM.zip › Figure 5/5A/PD21 PLZF&GFRa1/PD21 Control testis anti-PLZF.tif]

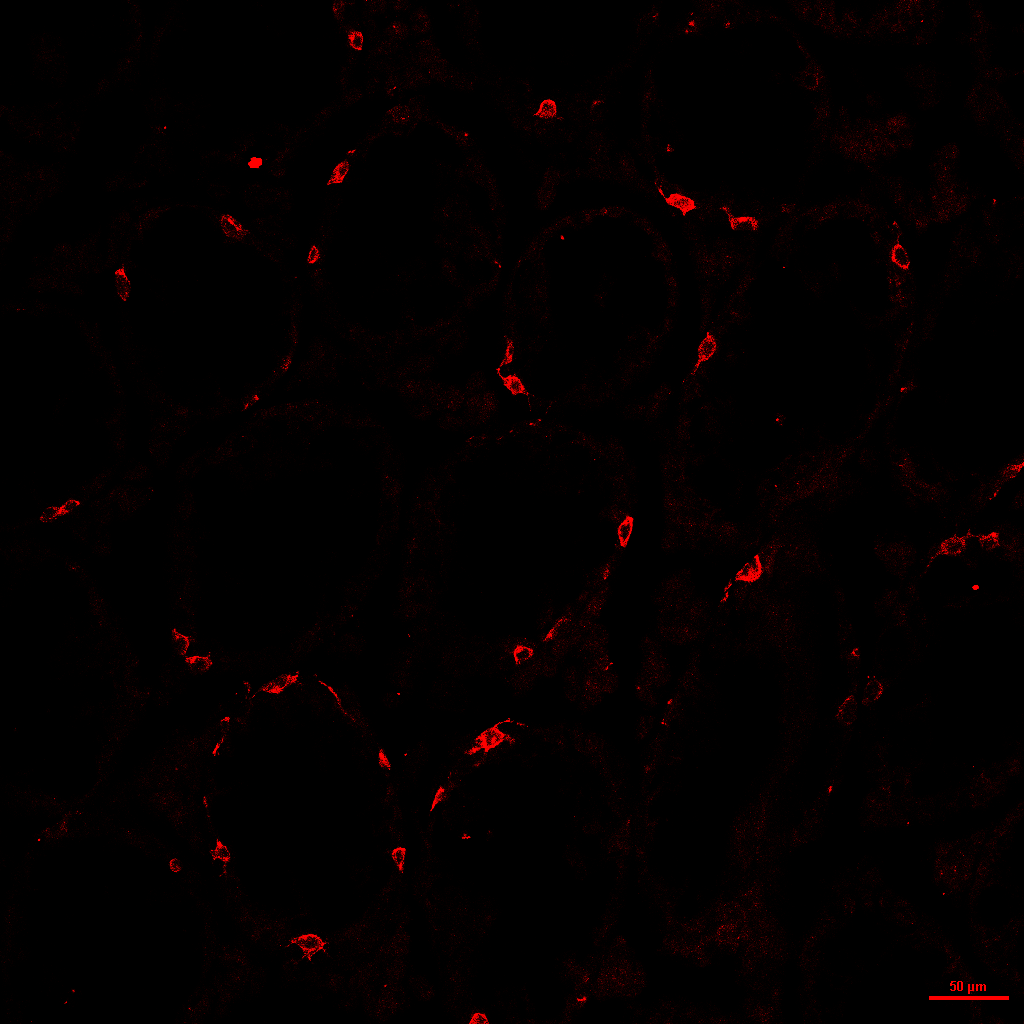

Supplement: Supplementary file 8 — Source data Fig. 5 [file 44319_2025_487_MOESM8_ESM.zip › Figure 5/5A/PD42 PLZF&GFRa1/PD42 Brca1 vKO testis anti-GFRa1.tif]

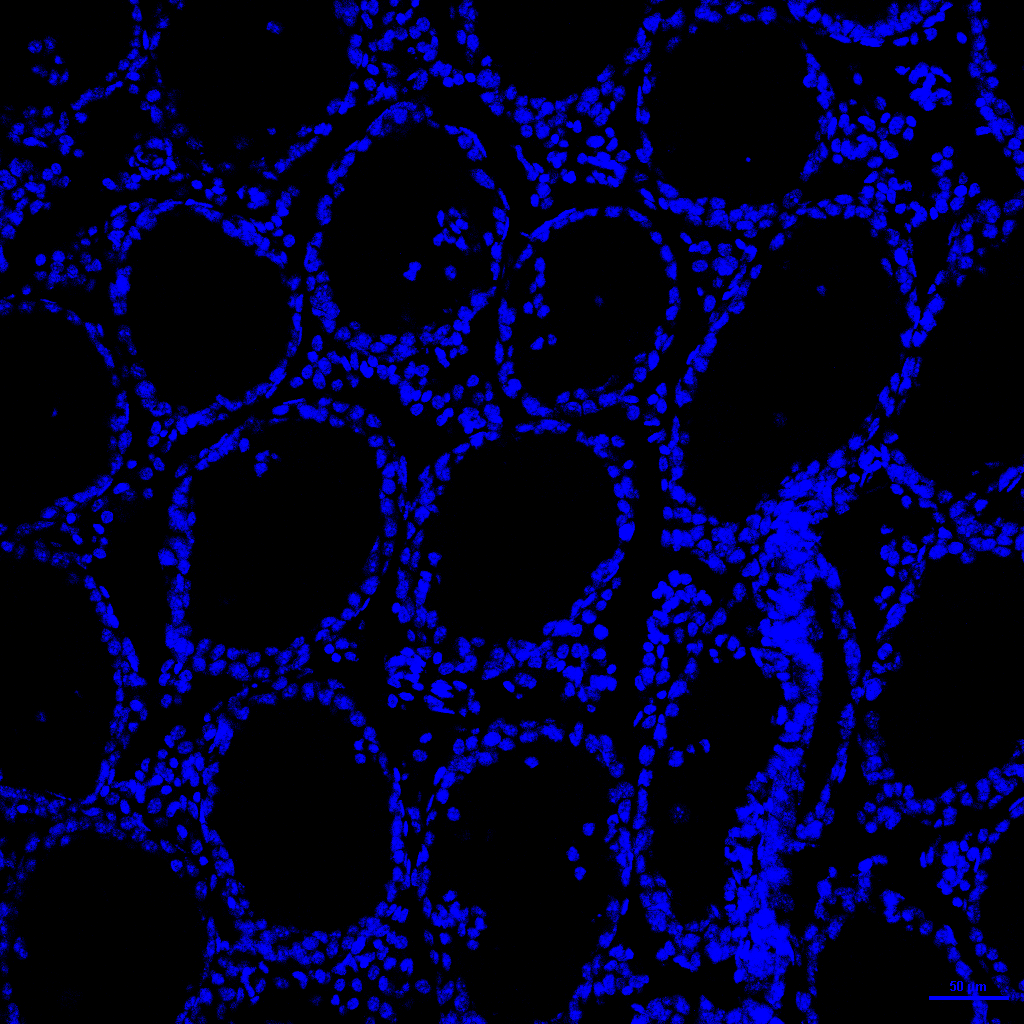

Supplement: Supplementary file 8 — Source data Fig. 5 [file 44319_2025_487_MOESM8_ESM.zip › Figure 5/5A/PD42 PLZF&GFRa1/PD42 Brca1 vKO testis anti-PLZF&GFRa1 Hoechst.tif]

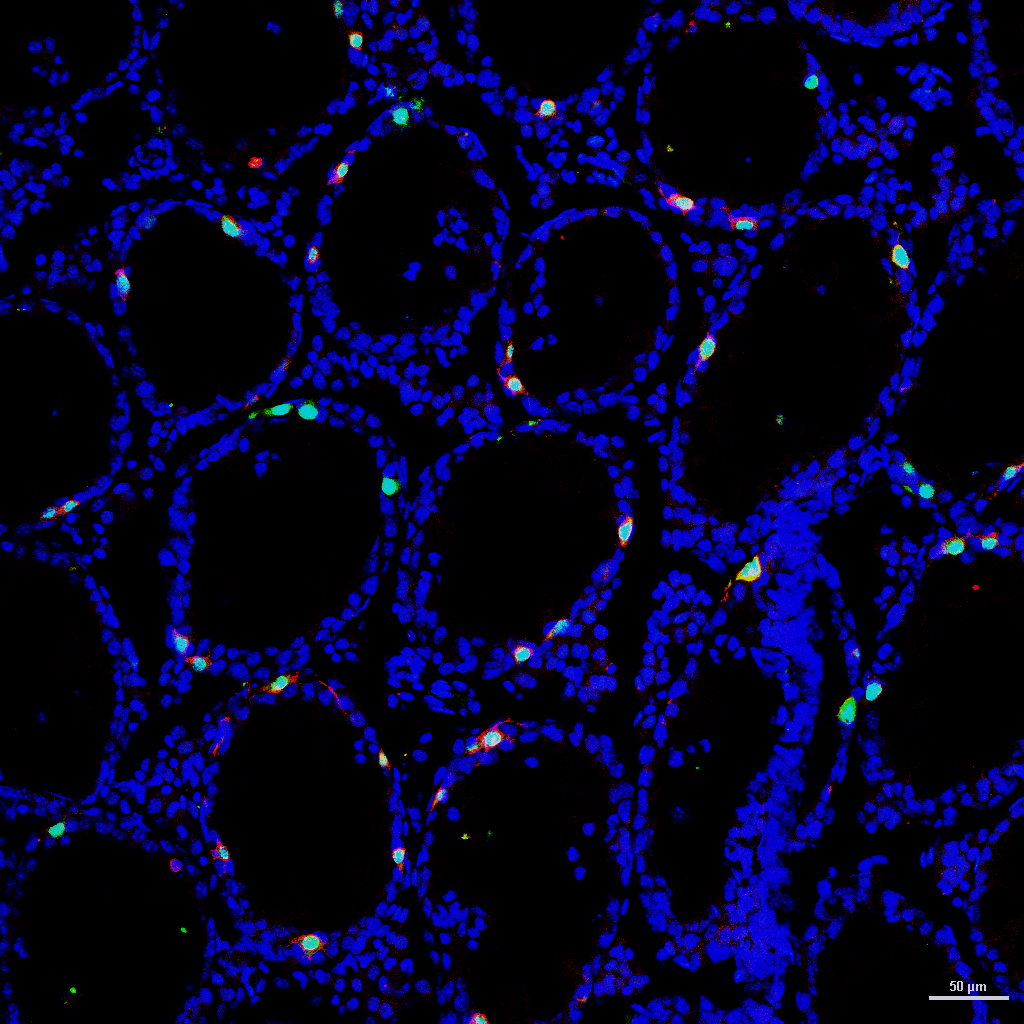

Supplement: Supplementary file 8 — Source data Fig. 5 [file 44319_2025_487_MOESM8_ESM.zip › Figure 5/5A/PD42 PLZF&GFRa1/PD42 Brca1 vKO testis anti-PLZF&GFRa1 Hoechst_overlay.tif]

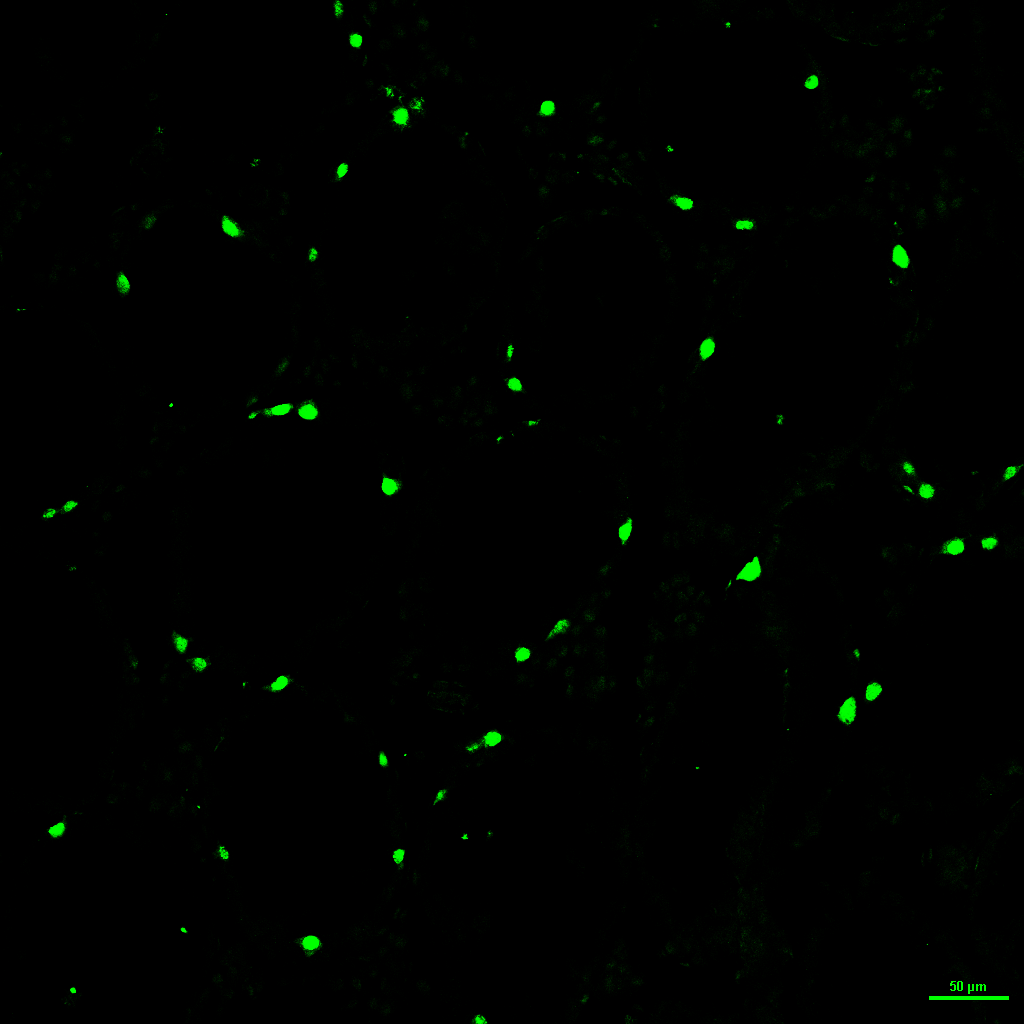

Supplement: Supplementary file 8 — Source data Fig. 5 [file 44319_2025_487_MOESM8_ESM.zip › Figure 5/5A/PD42 PLZF&GFRa1/PD42 Brca1 vKO testis anti-PLZF.tif]

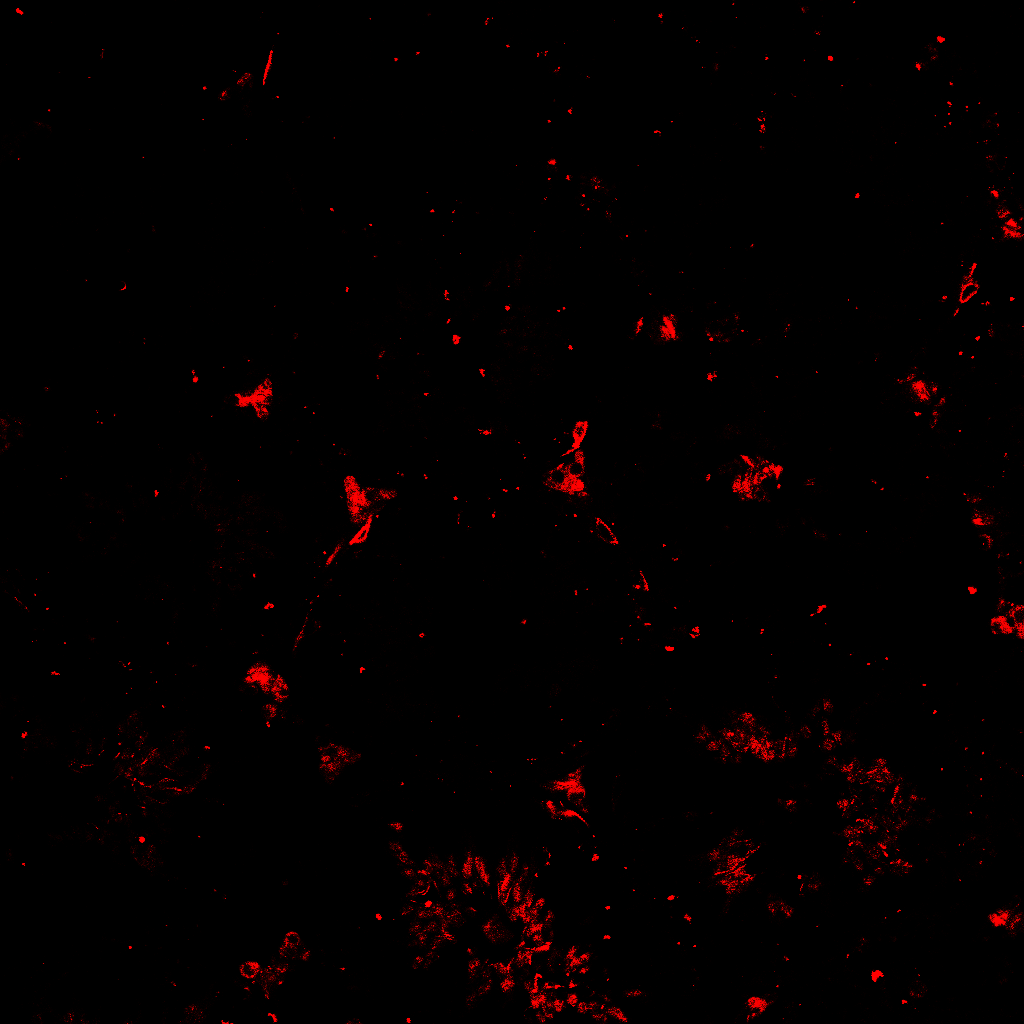

Supplement: Supplementary file 8 — Source data Fig. 5 [file 44319_2025_487_MOESM8_ESM.zip › Figure 5/5A/PD42 PLZF&GFRa1/PD42 Control testis anti-GFRa1.tif]

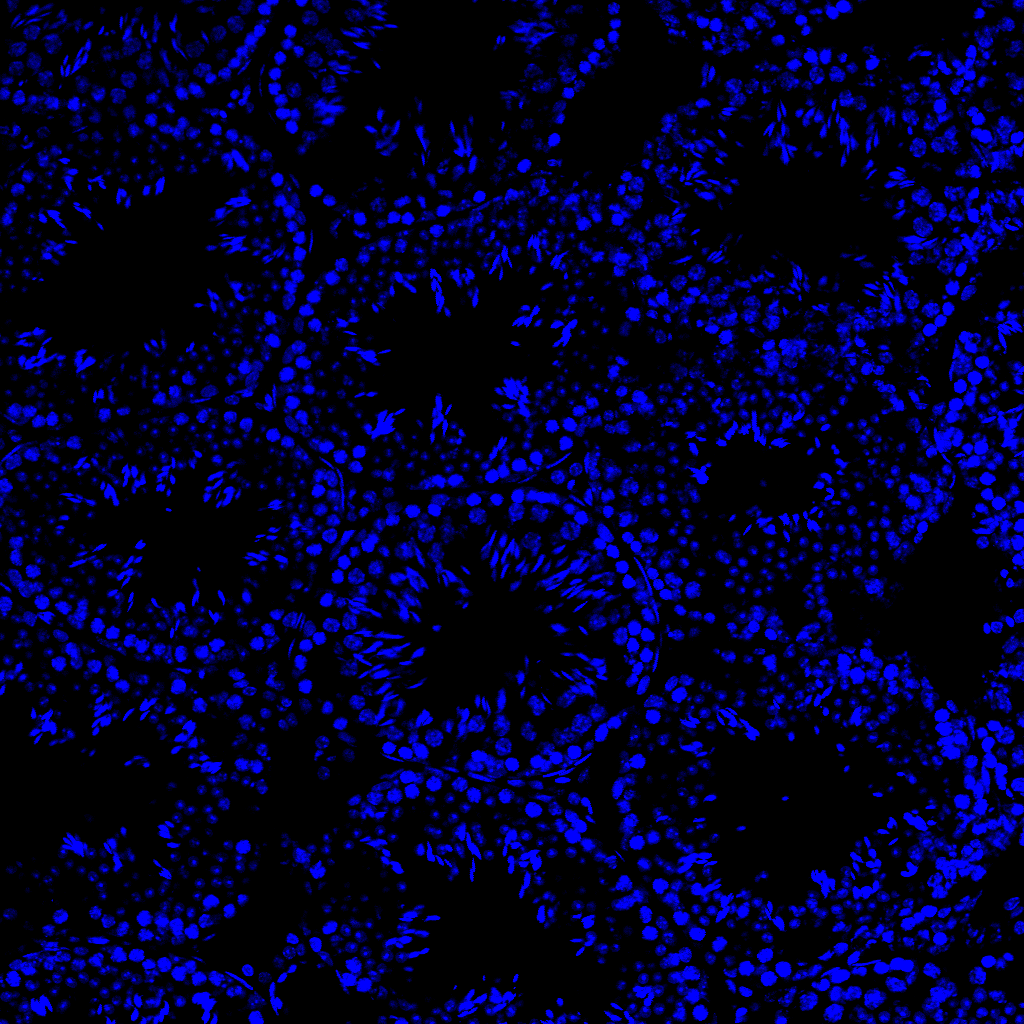

Supplement: Supplementary file 8 — Source data Fig. 5 [file 44319_2025_487_MOESM8_ESM.zip › Figure 5/5A/PD42 PLZF&GFRa1/PD42 Control testis anti-PLZF&GFRa1 Hoechst.tif]

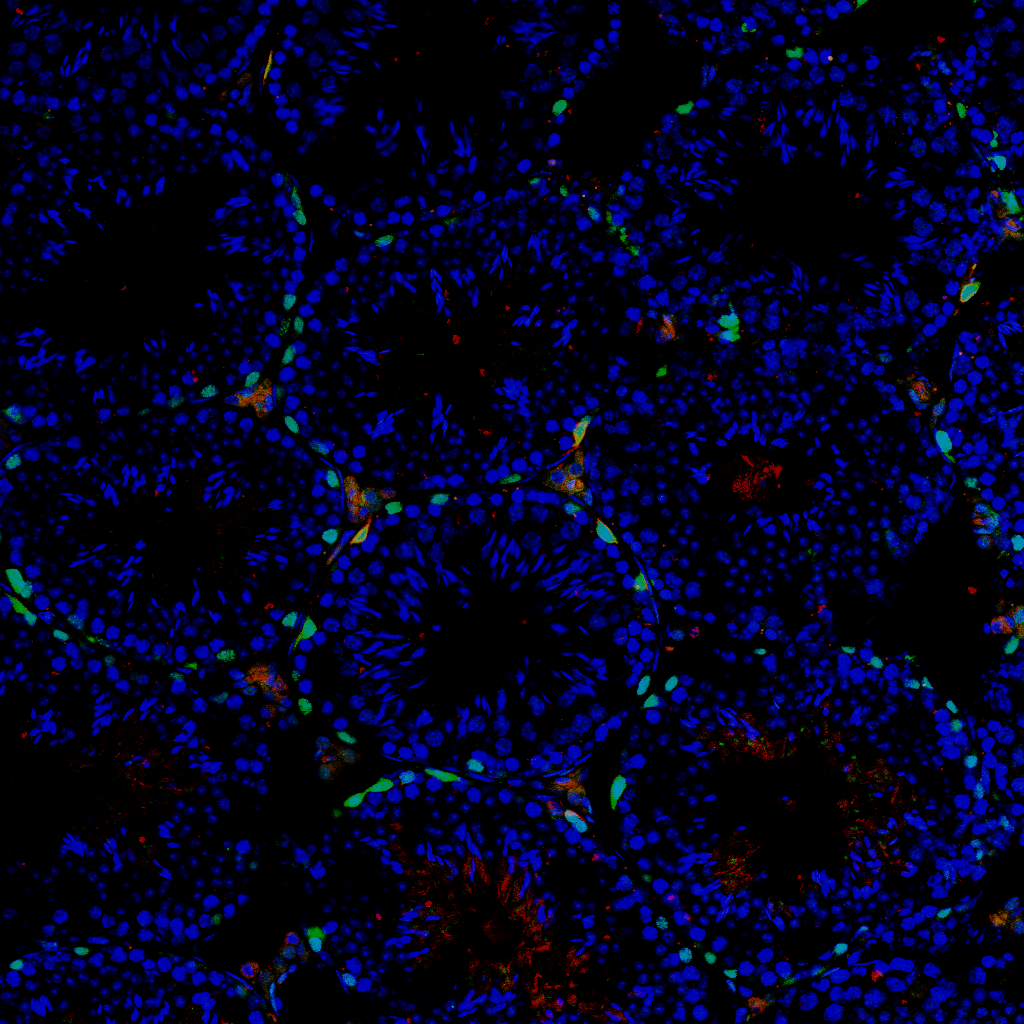

Supplement: Supplementary file 8 — Source data Fig. 5 [file 44319_2025_487_MOESM8_ESM.zip › Figure 5/5A/PD42 PLZF&GFRa1/PD42 Control testis anti-PLZF&GFRa1 Hoechst_overlay.tif]

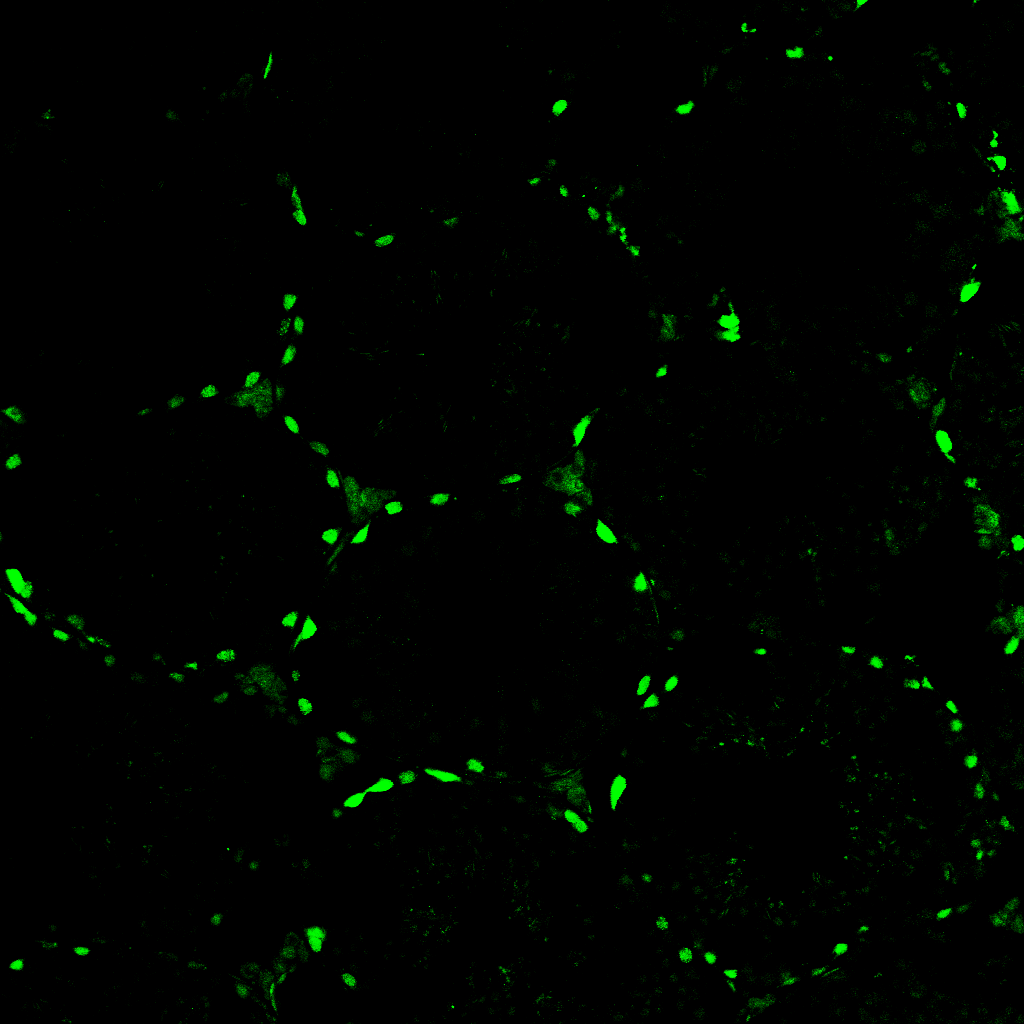

Supplement: Supplementary file 8 — Source data Fig. 5 [file 44319_2025_487_MOESM8_ESM.zip › Figure 5/5A/PD42 PLZF&GFRa1/PD42 Control testis anti-PLZF.tif]

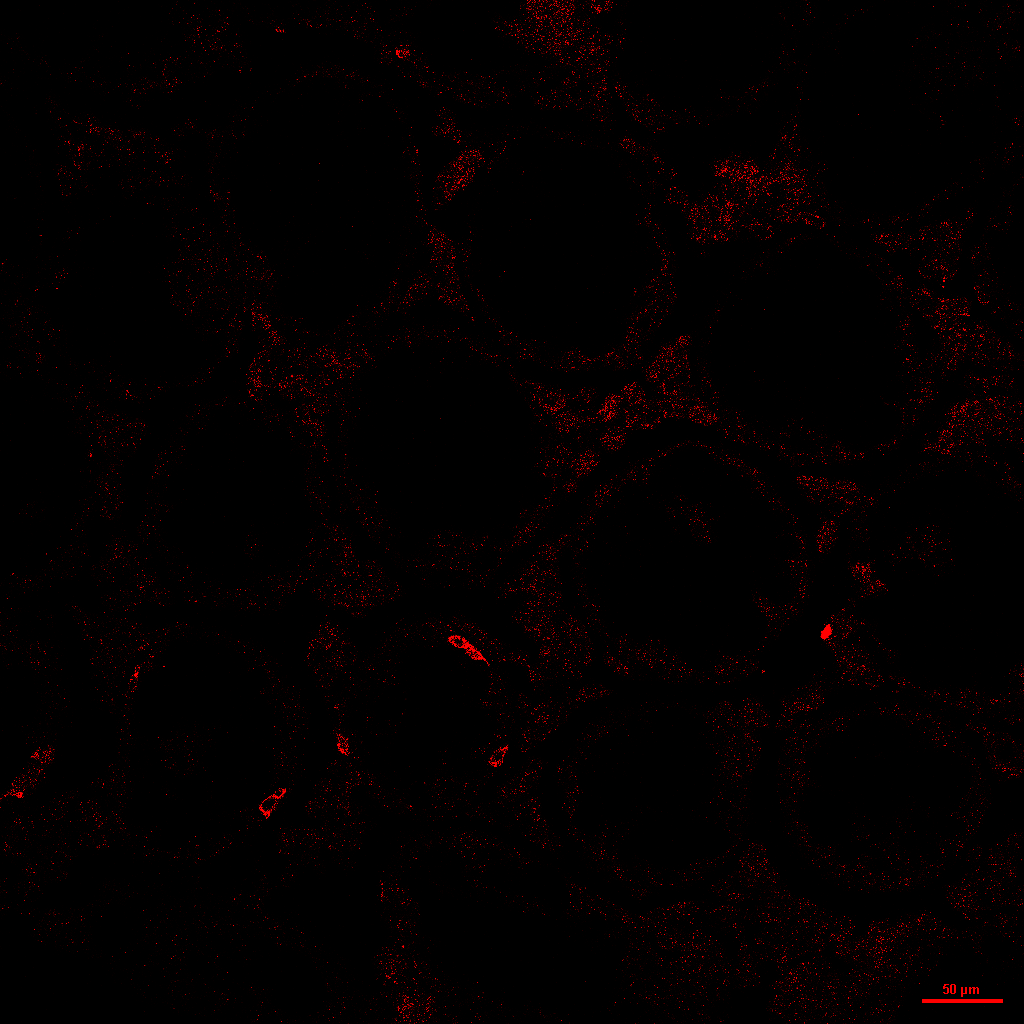

Supplement: Supplementary file 8 — Source data Fig. 5 [file 44319_2025_487_MOESM8_ESM.zip › Figure 5/5A/PD90 PLZF&GFRa1/PD90 Brca1 vKO testis anti-GFRa1.tif]

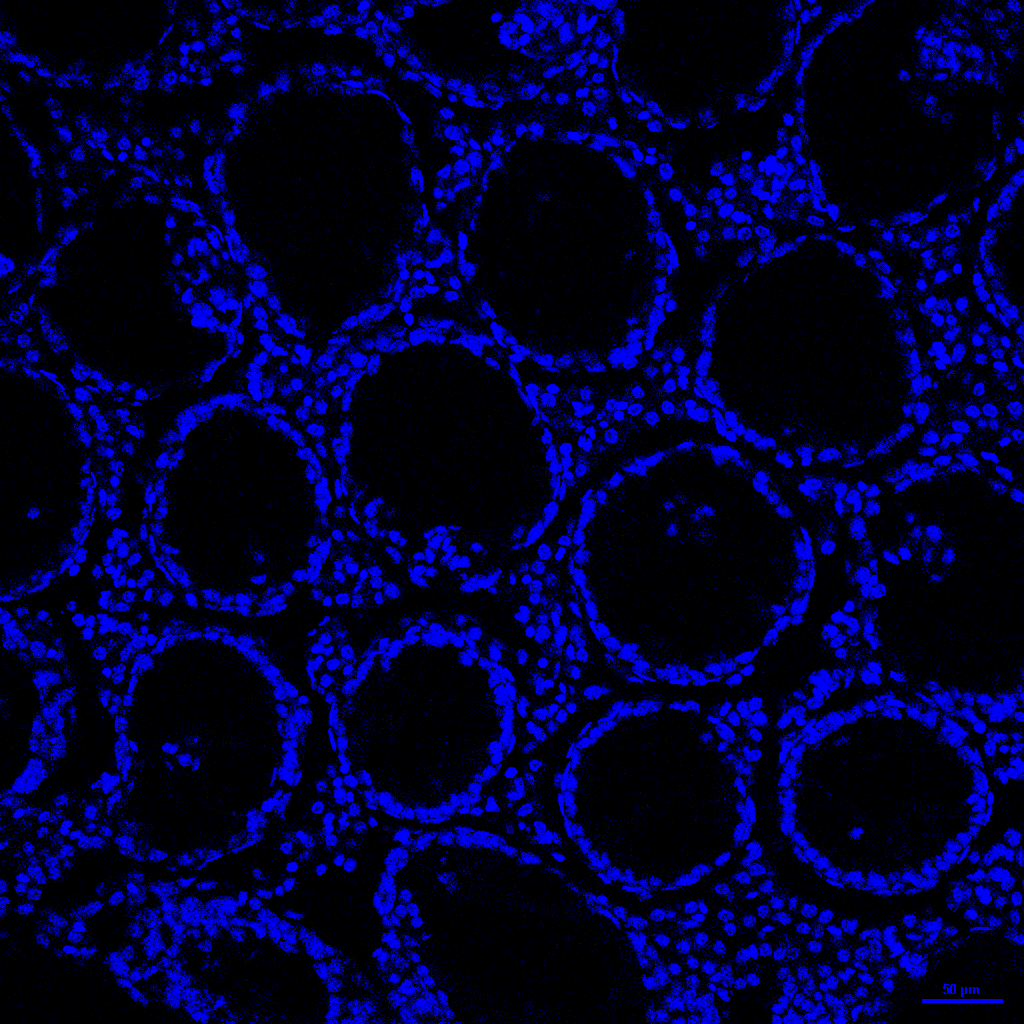

Supplement: Supplementary file 8 — Source data Fig. 5 [file 44319_2025_487_MOESM8_ESM.zip › Figure 5/5A/PD90 PLZF&GFRa1/PD90 Brca1 vKO testis anti-PLZF&GFRa1 Hoechst.tif]

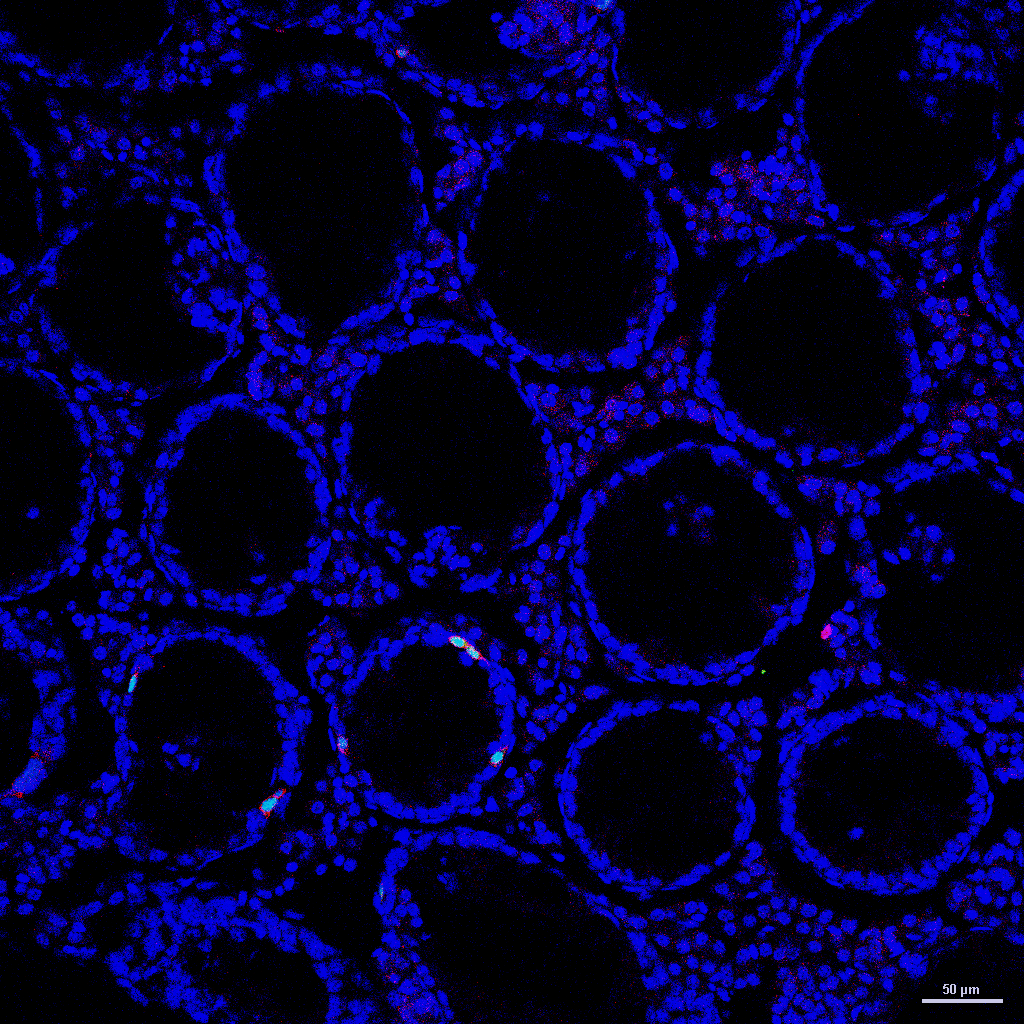

Supplement: Supplementary file 8 — Source data Fig. 5 [file 44319_2025_487_MOESM8_ESM.zip › Figure 5/5A/PD90 PLZF&GFRa1/PD90 Brca1 vKO testis anti-PLZF&GFRa1 Hoechst_overlay.tif]

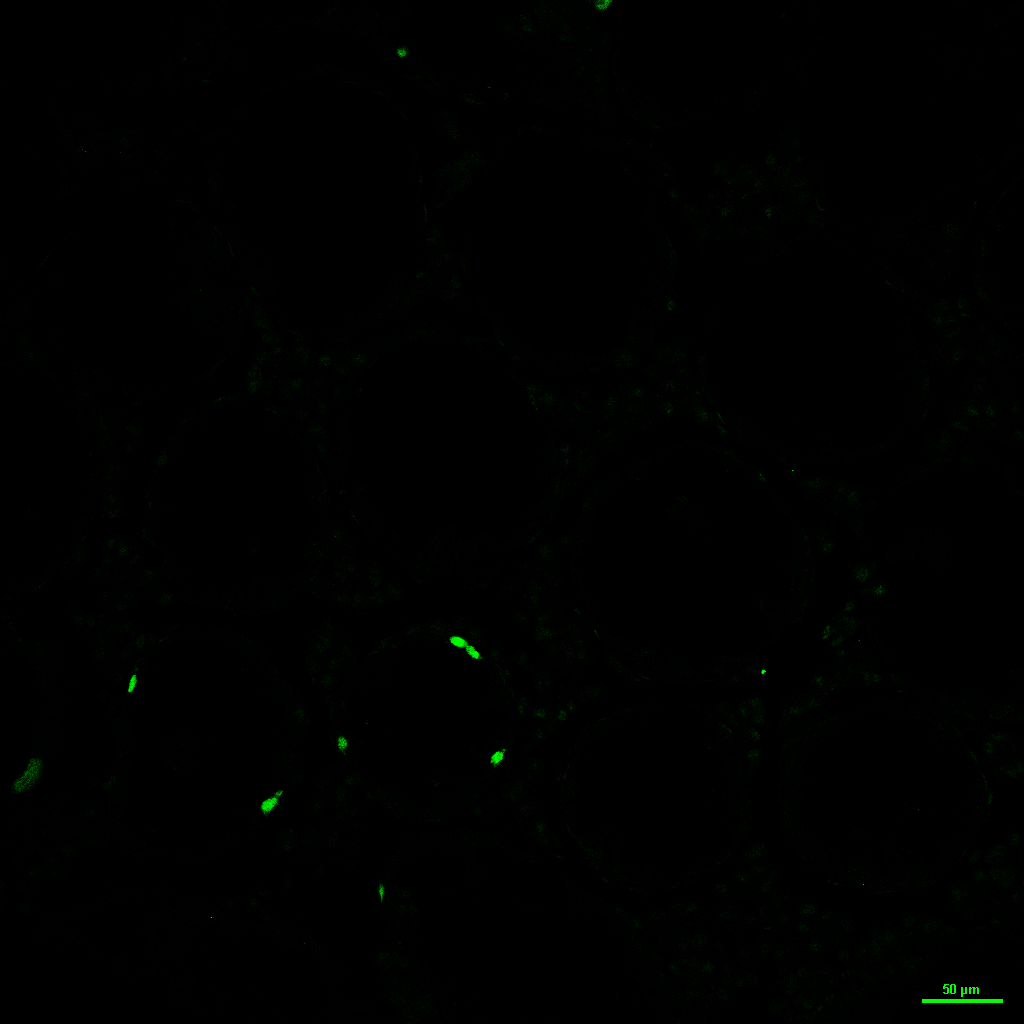

Supplement: Supplementary file 8 — Source data Fig. 5 [file 44319_2025_487_MOESM8_ESM.zip › Figure 5/5A/PD90 PLZF&GFRa1/PD90 Brca1 vKO testis anti-PLZF.tif]

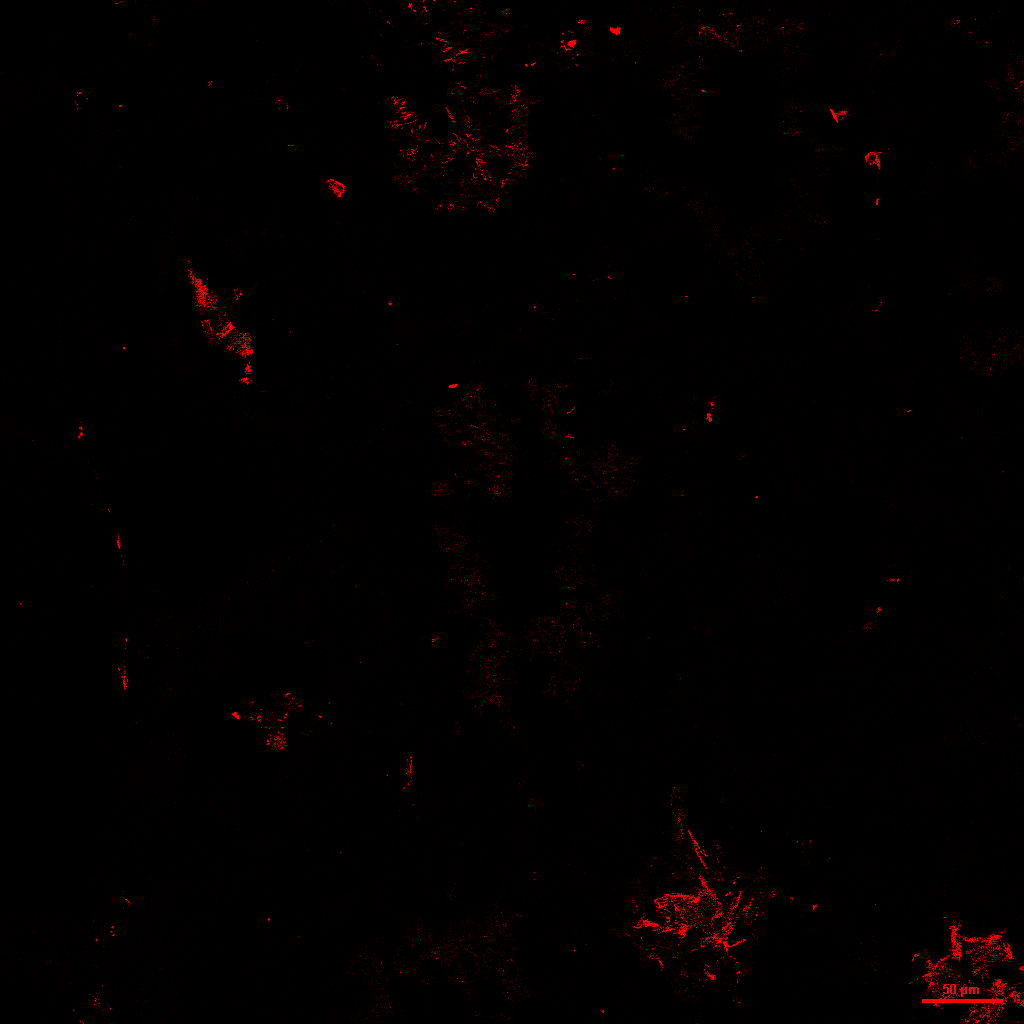

Supplement: Supplementary file 8 — Source data Fig. 5 [file 44319_2025_487_MOESM8_ESM.zip › Figure 5/5A/PD90 PLZF&GFRa1/PD90 Control testis anti-GFRa1.tif]

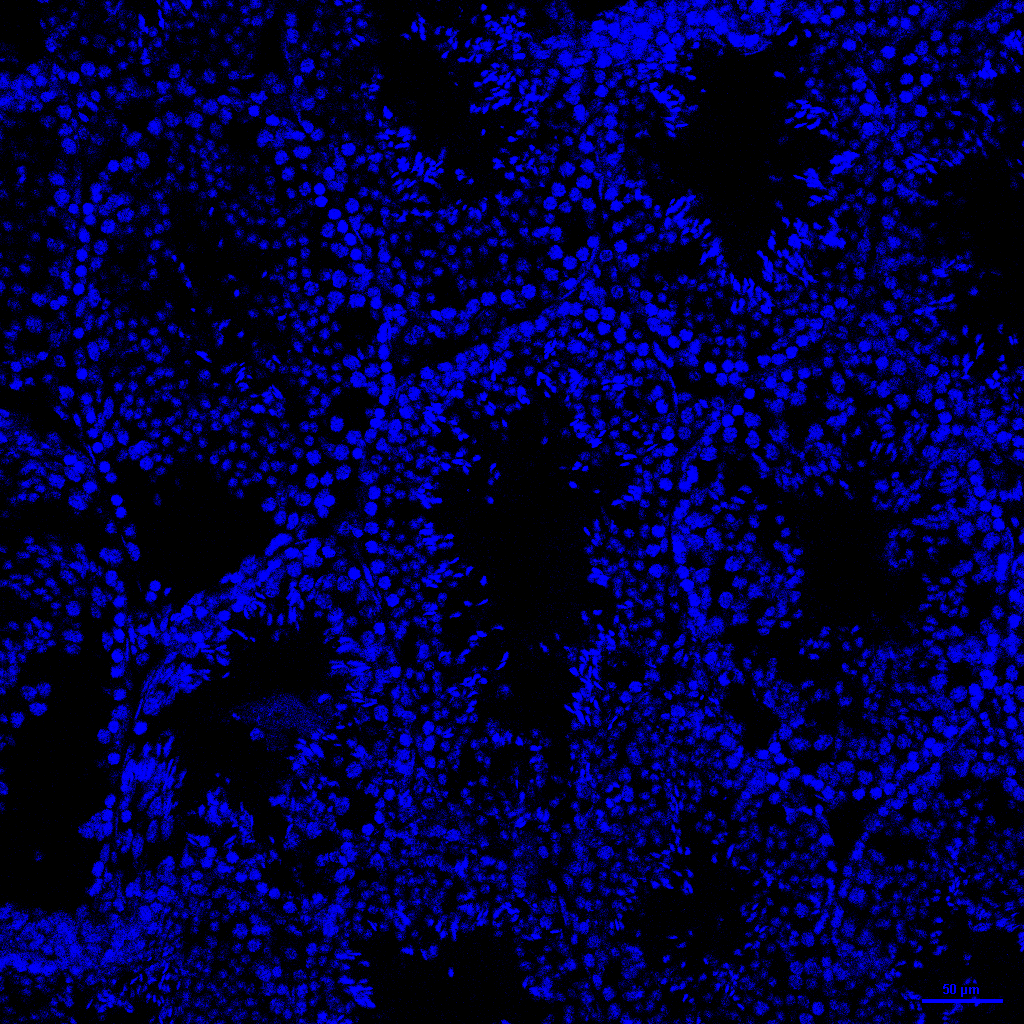

Supplement: Supplementary file 8 — Source data Fig. 5 [file 44319_2025_487_MOESM8_ESM.zip › Figure 5/5A/PD90 PLZF&GFRa1/PD90 Control testis anti-PLZF&GFRa1 Hoechst.tif]

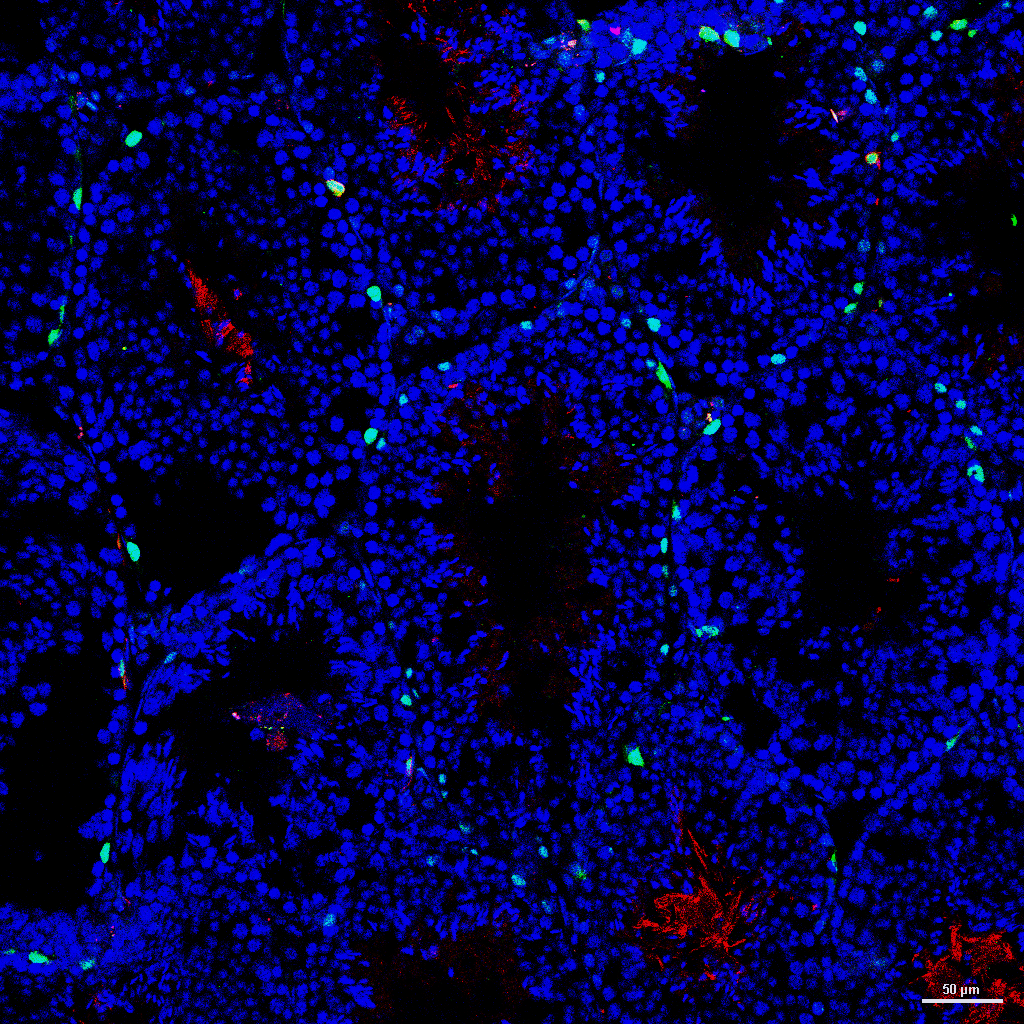

Supplement: Supplementary file 8 — Source data Fig. 5 [file 44319_2025_487_MOESM8_ESM.zip › Figure 5/5A/PD90 PLZF&GFRa1/PD90 Control testis anti-PLZF&GFRa1 Hoechst_overlay.tif]

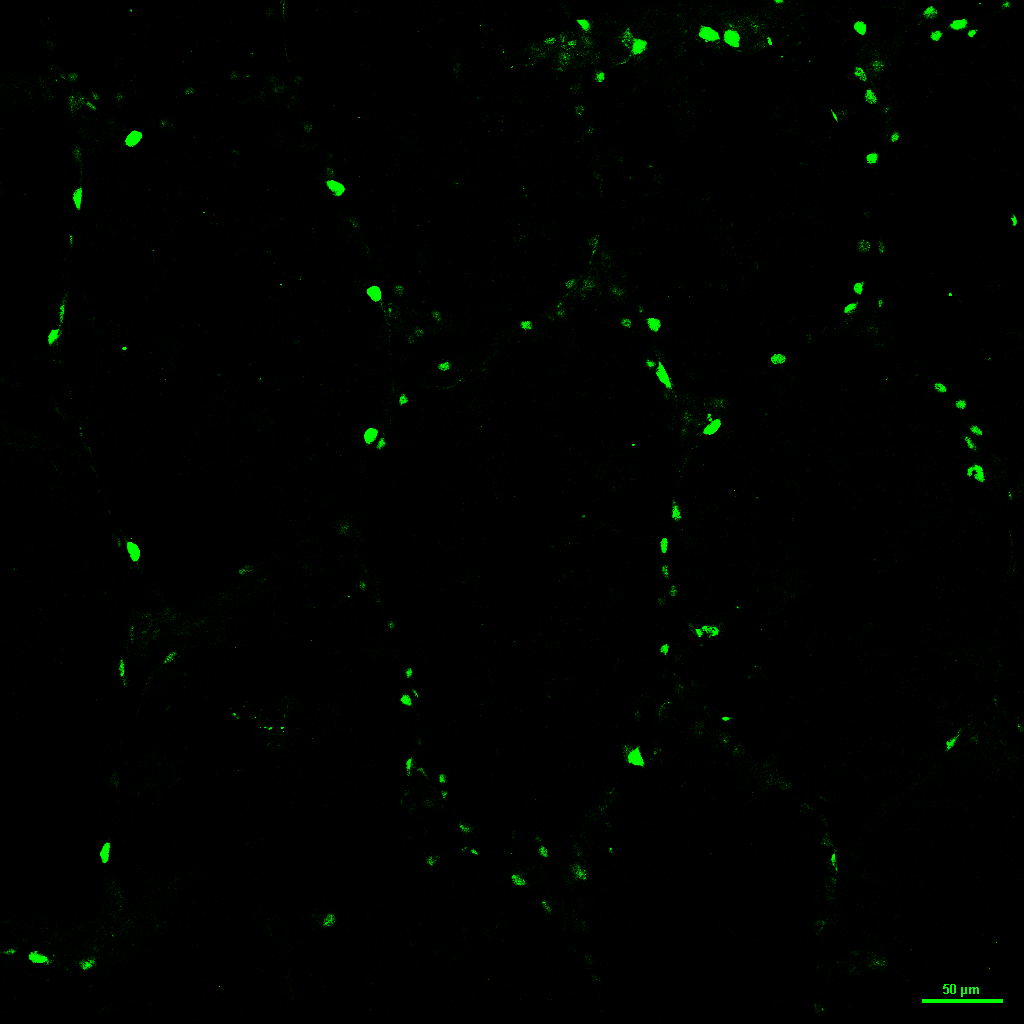

Supplement: Supplementary file 8 — Source data Fig. 5 [file 44319_2025_487_MOESM8_ESM.zip › Figure 5/5A/PD90 PLZF&GFRa1/PD90 Control testis anti-PLZF.tif]

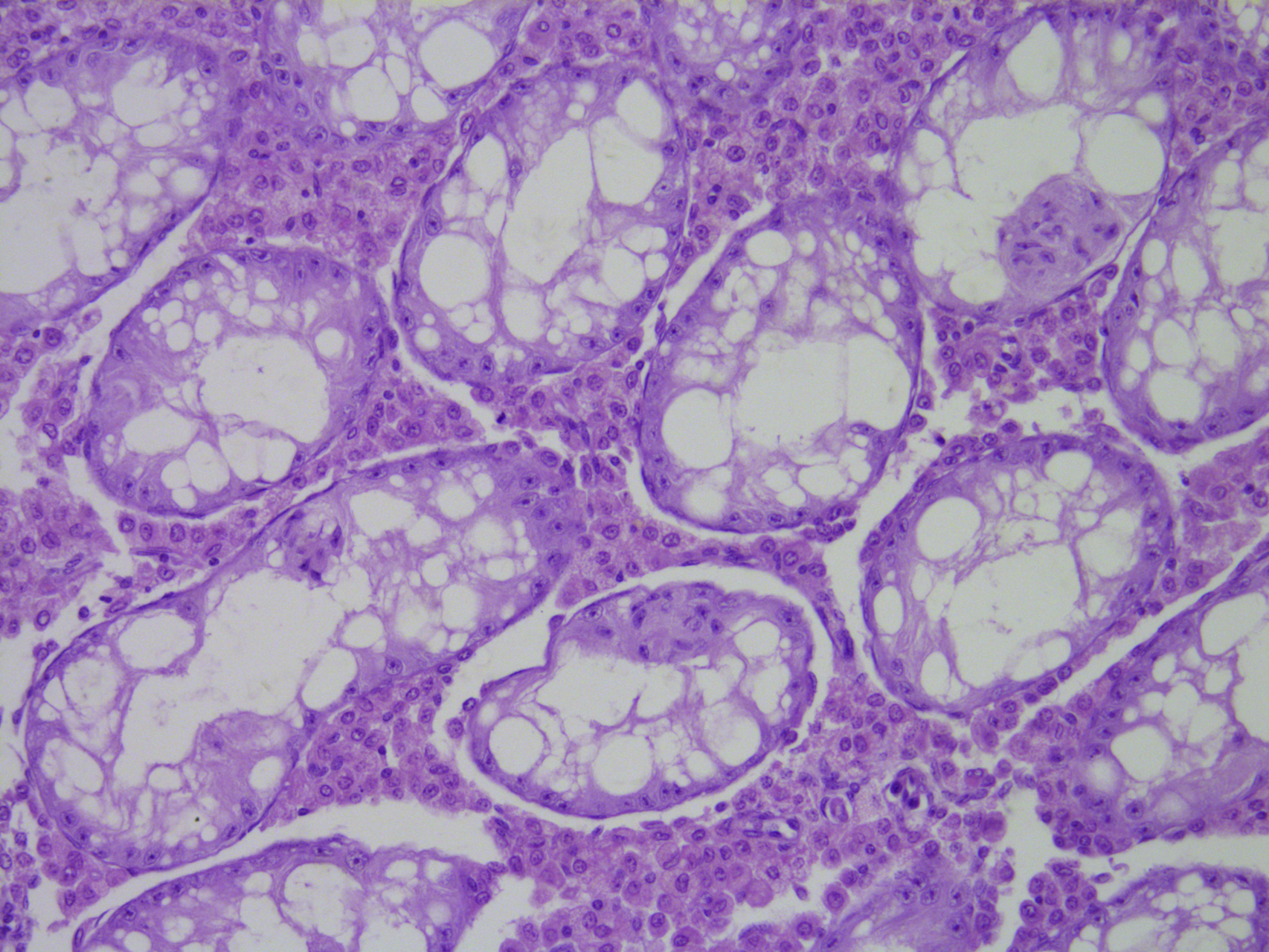

Supplement: Supplementary file 8 — Source data Fig. 5 [file 44319_2025_487_MOESM8_ESM.zip › Figure 5/5D/PD180 H&E/PD180 Brca1 vKO testis.tif]

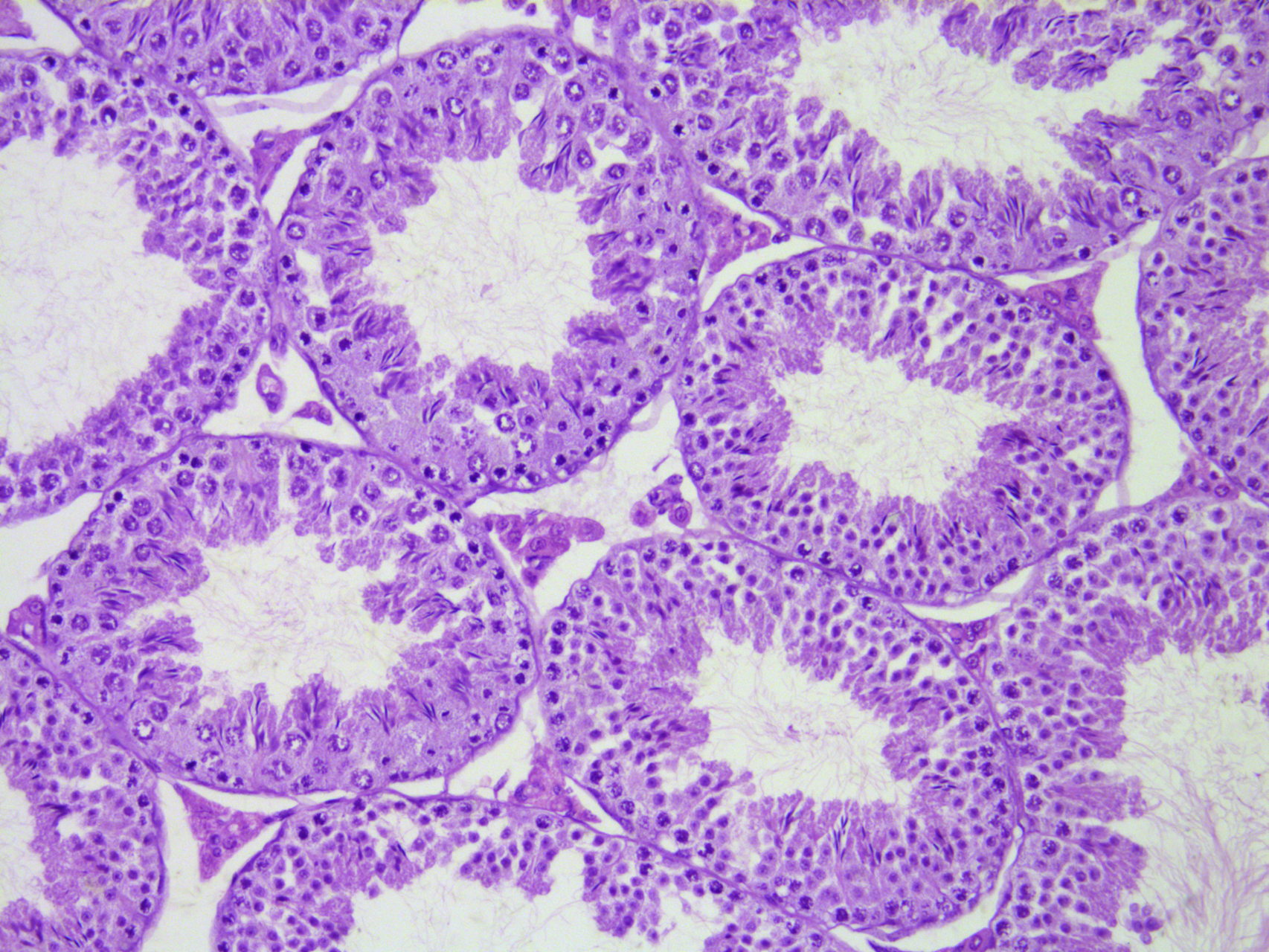

Supplement: Supplementary file 8 — Source data Fig. 5 [file 44319_2025_487_MOESM8_ESM.zip › Figure 5/5D/PD180 H&E/PD180 Control testis.tif]

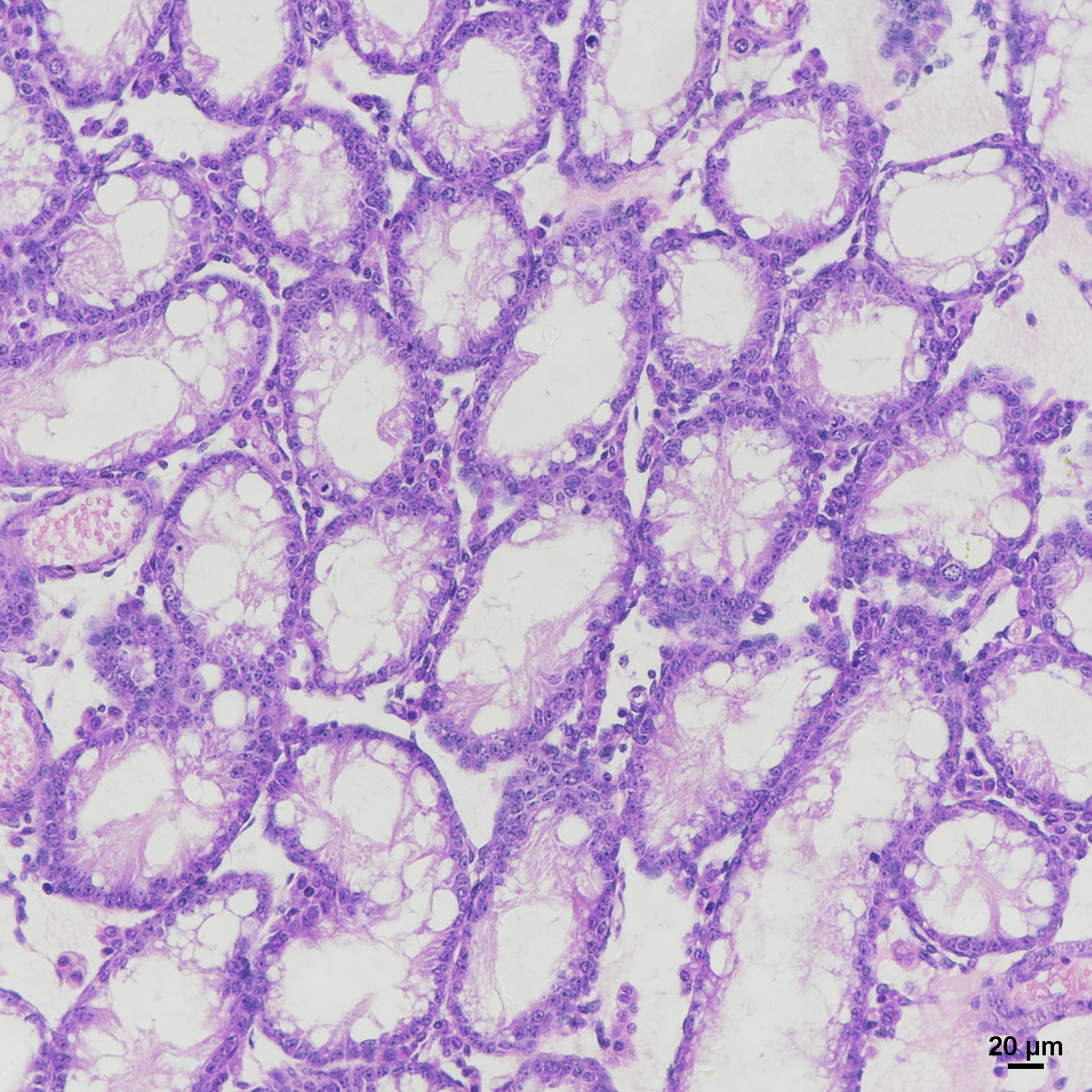

Supplement: Supplementary file 8 — Source data Fig. 5 [file 44319_2025_487_MOESM8_ESM.zip › Figure 5/5D/PD21 H&E/PD21 Brca1 vKO testis.tif]

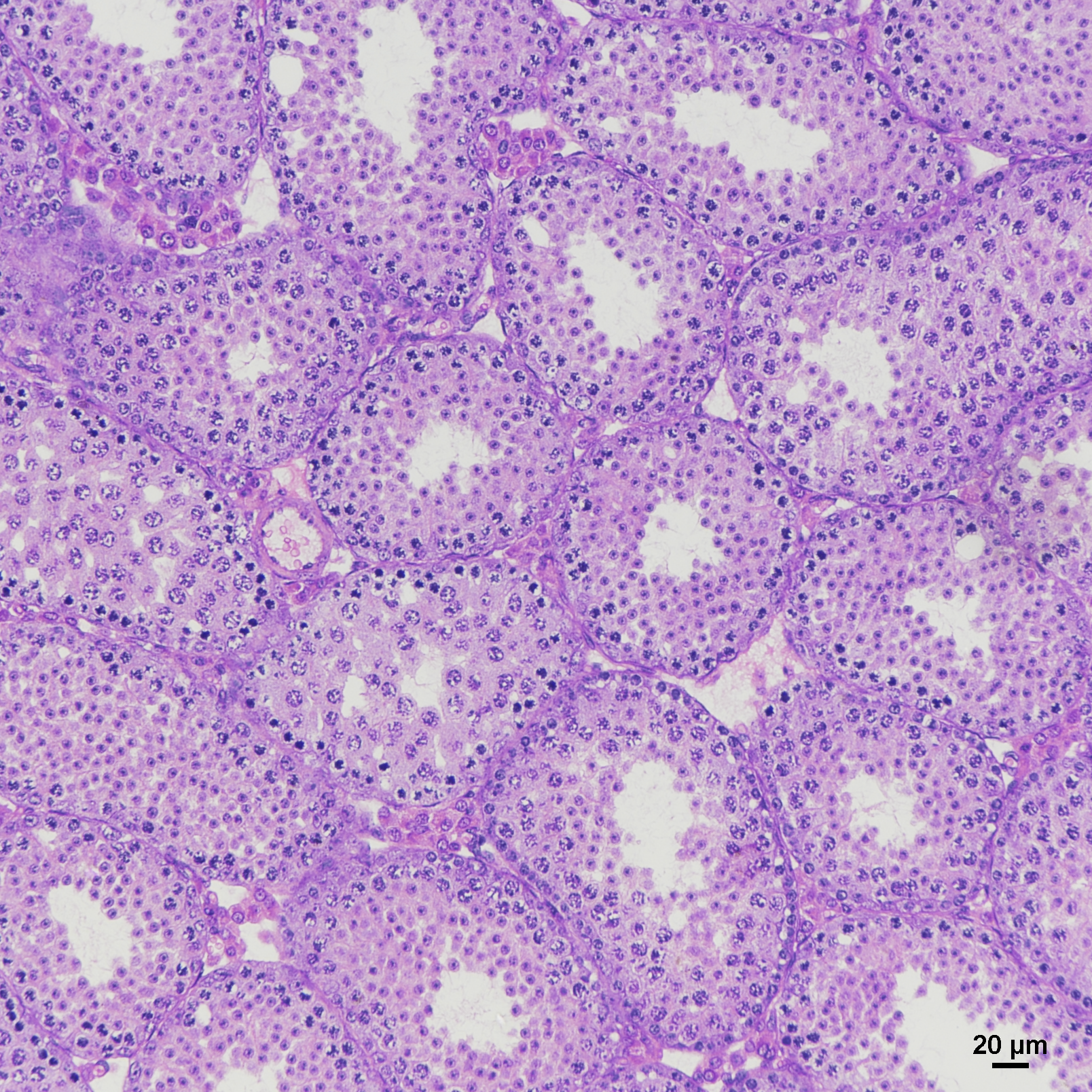

Supplement: Supplementary file 8 — Source data Fig. 5 [file 44319_2025_487_MOESM8_ESM.zip › Figure 5/5D/PD21 H&E/PD21 Control testis.tif]

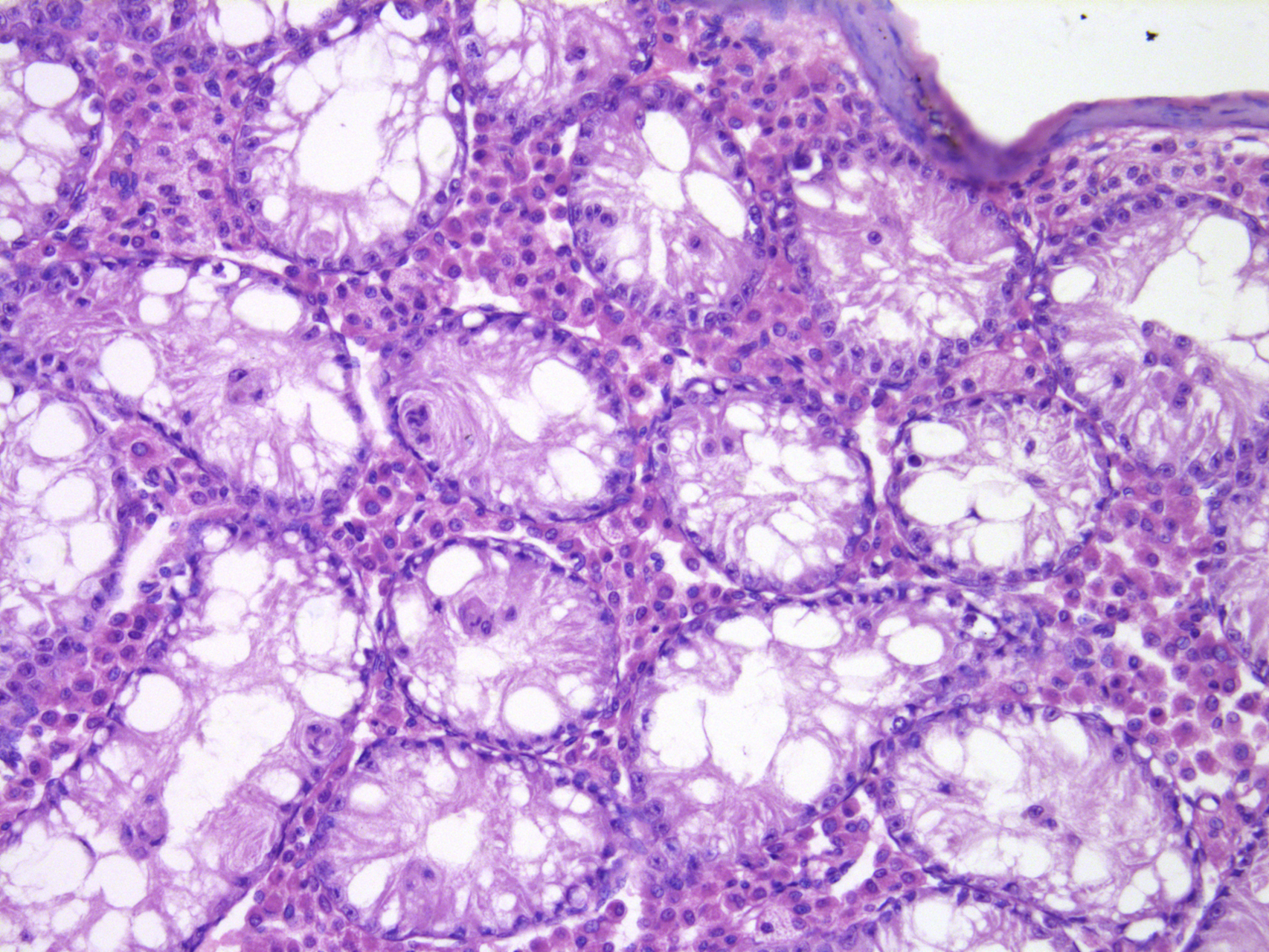

Supplement: Supplementary file 8 — Source data Fig. 5 [file 44319_2025_487_MOESM8_ESM.zip › Figure 5/5D/PD42 H&E/PD42 Brca1 vKO testis.tif]

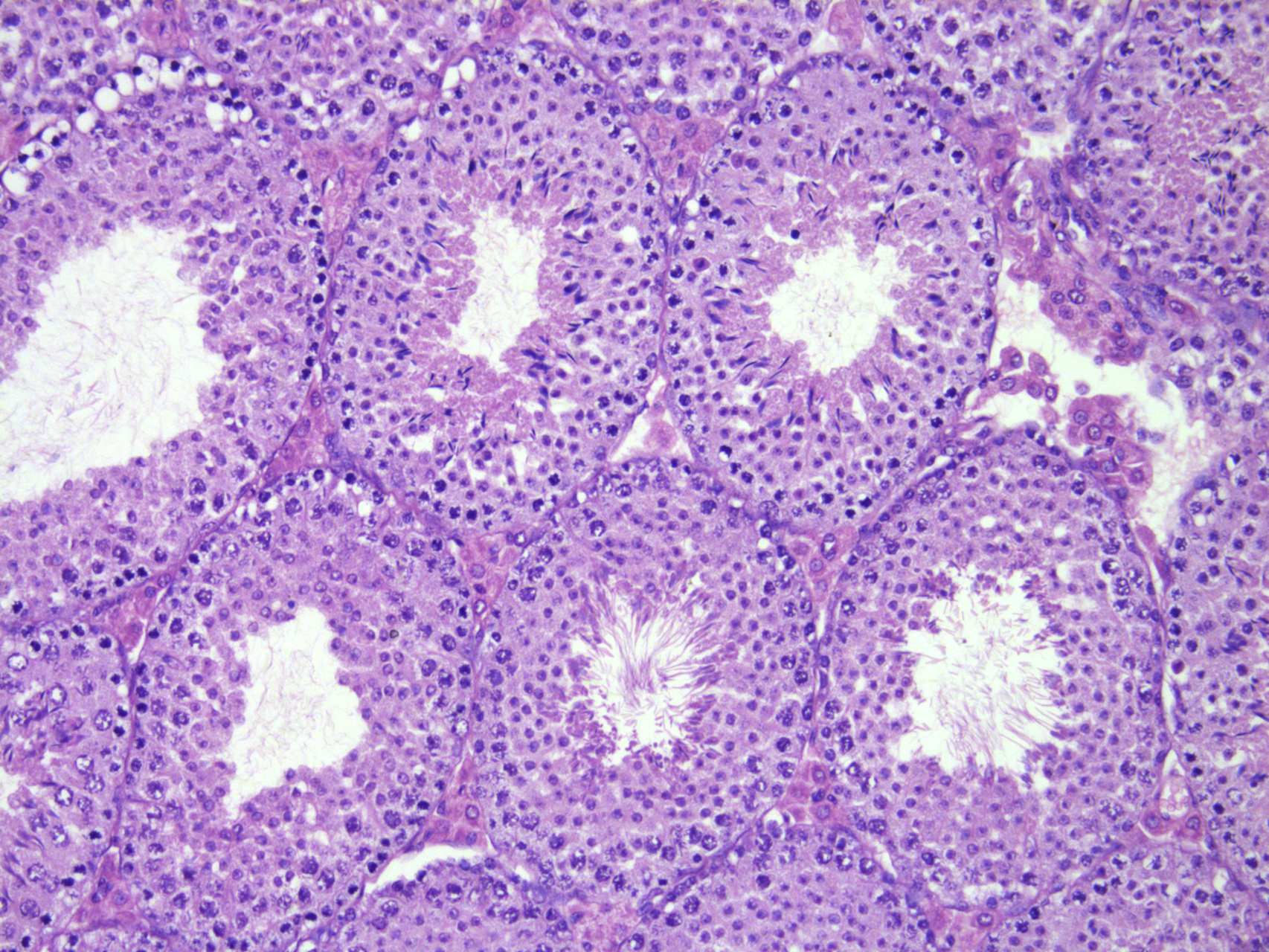

Supplement: Supplementary file 8 — Source data Fig. 5 [file 44319_2025_487_MOESM8_ESM.zip › Figure 5/5D/PD42 H&E/PD42 Control testis.tif]

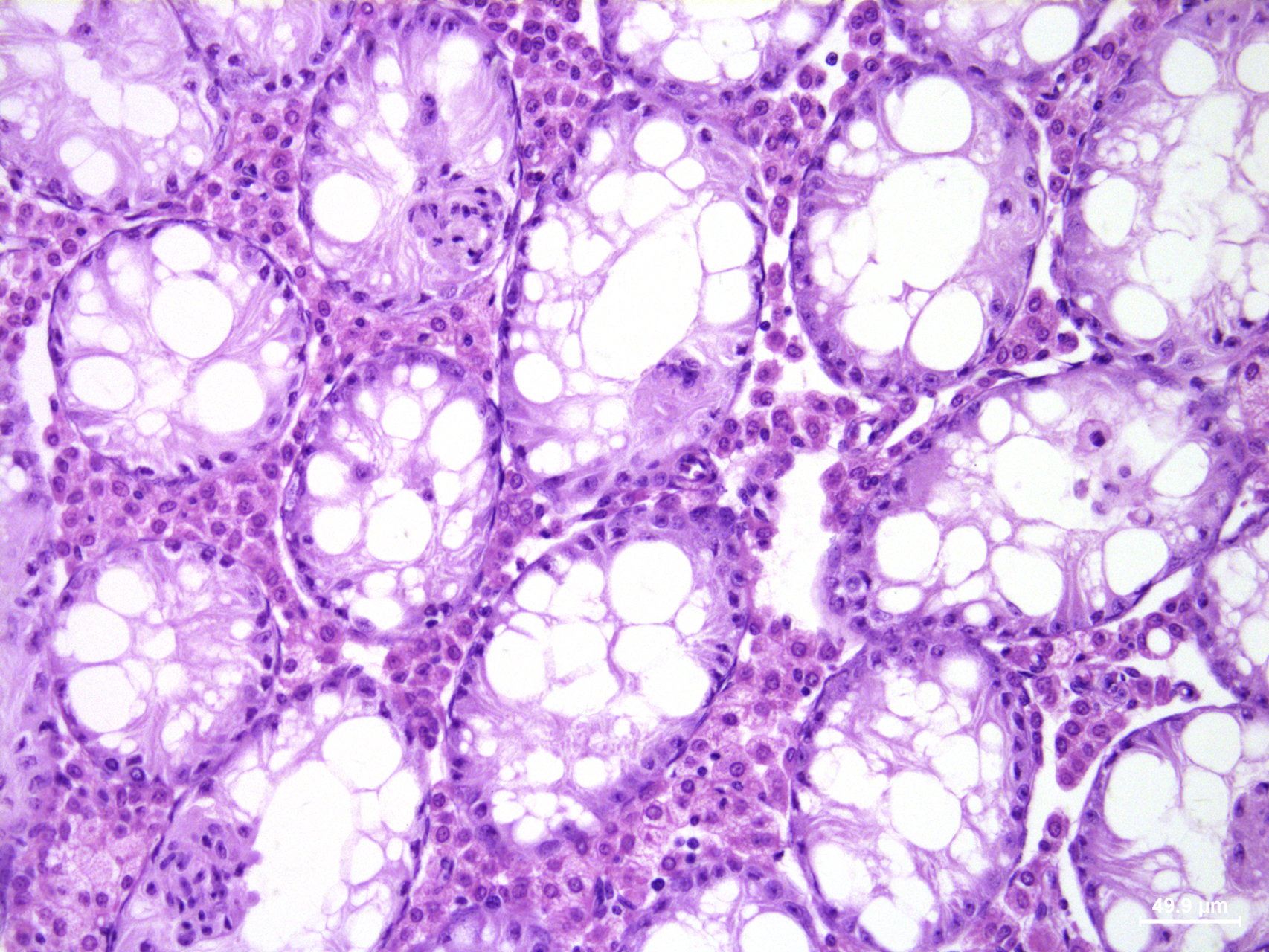

Supplement: Supplementary file 8 — Source data Fig. 5 [file 44319_2025_487_MOESM8_ESM.zip › Figure 5/5D/PD90 H&E/PD90 Brca1 vKO testis.tif]

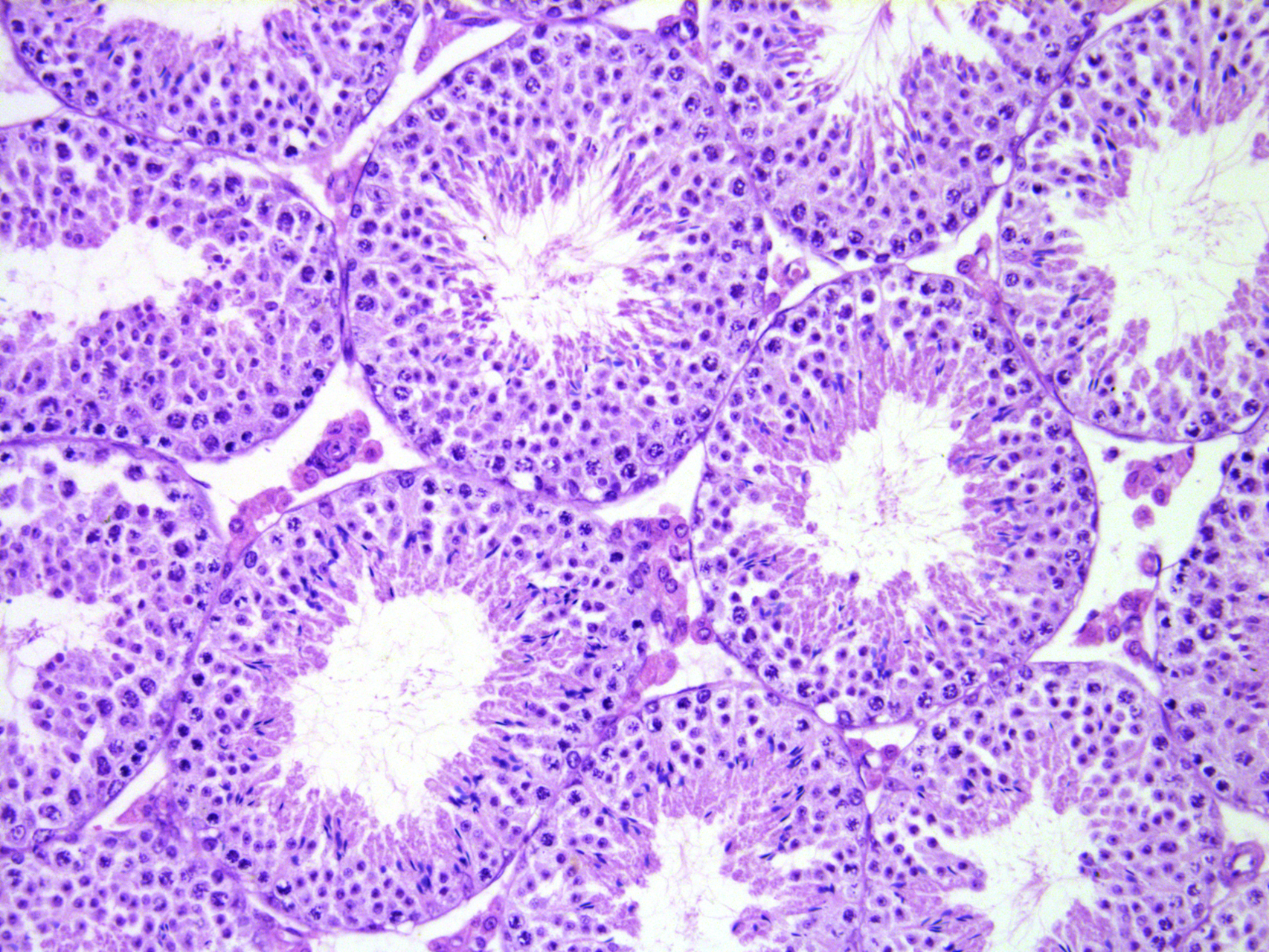

Supplement: Supplementary file 8 — Source data Fig. 5 [file 44319_2025_487_MOESM8_ESM.zip › Figure 5/5D/PD90 H&E/PD90 Control testis.tif]

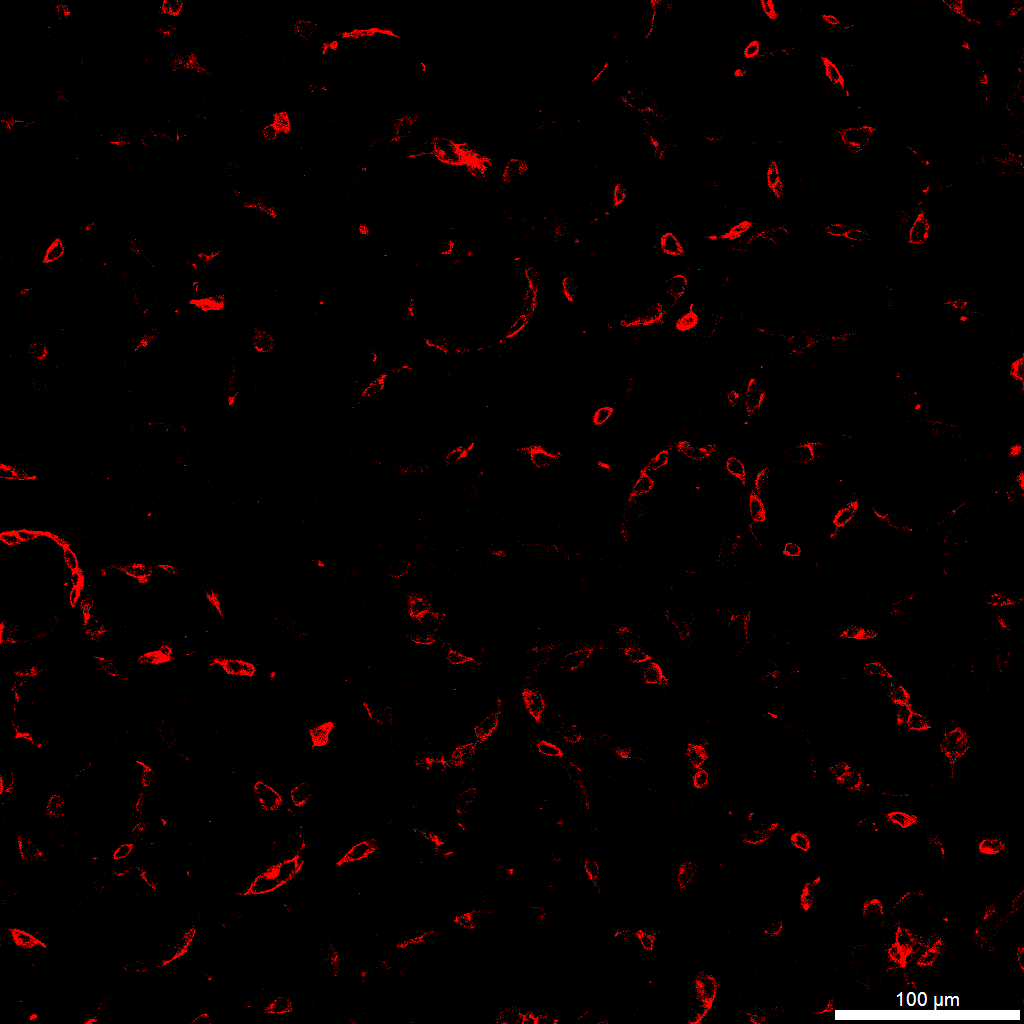

Supplement: Supplementary file 9 — Source data Fig. 6 [file 44319_2025_487_MOESM9_ESM.zip › Figure 6/6A/PD7 Brca1-p53 vDKO testis anti-GFRa1.tif]

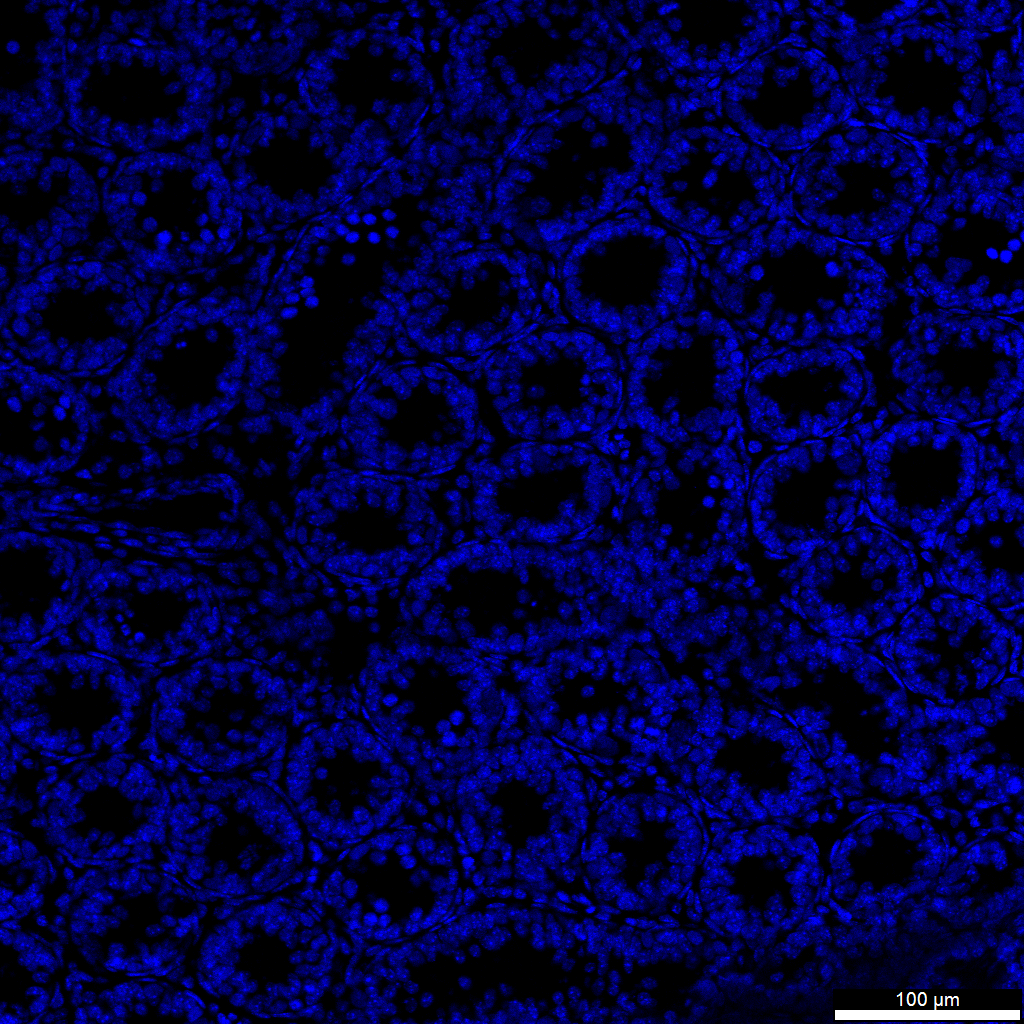

Supplement: Supplementary file 9 — Source data Fig. 6 [file 44319_2025_487_MOESM9_ESM.zip › Figure 6/6A/PD7 Brca1-p53 vDKO testis anti-PLZF&GFRa1 Hoechst.tif]

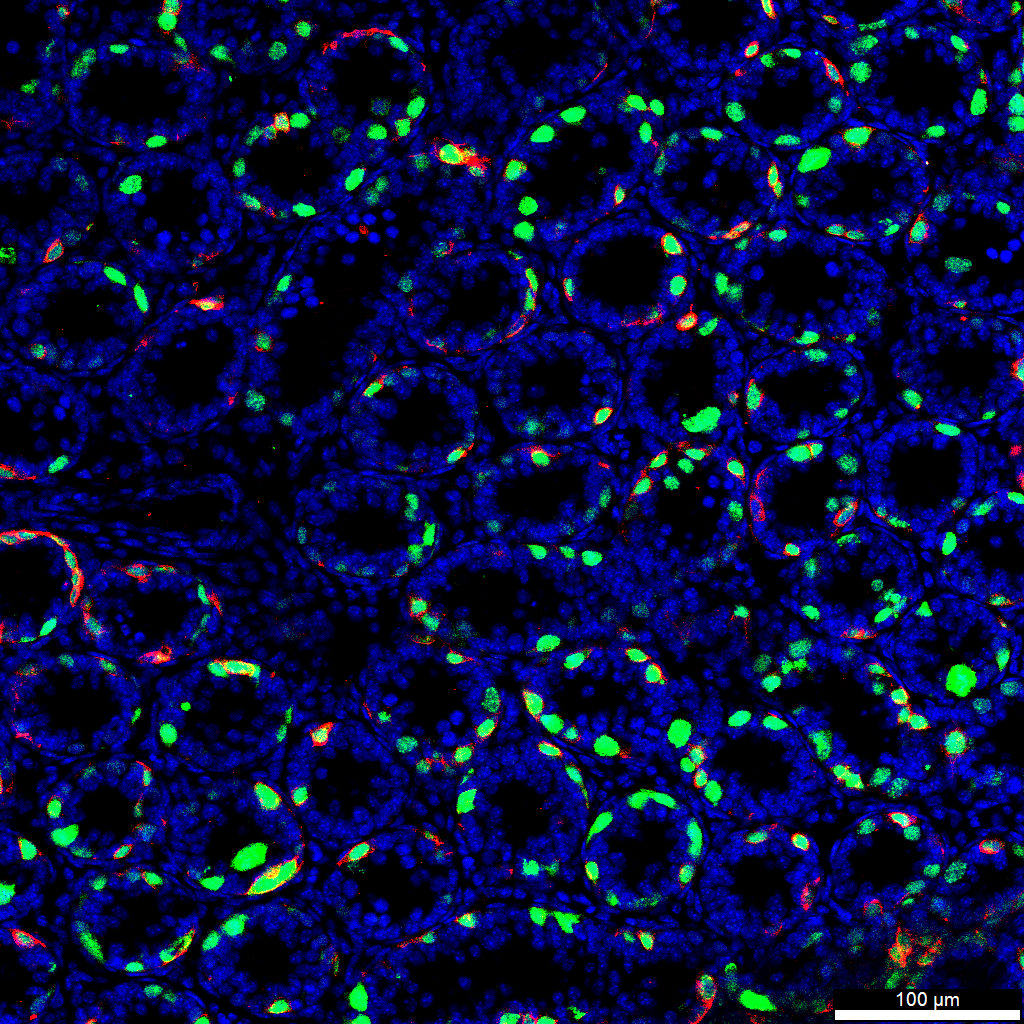

Supplement: Supplementary file 9 — Source data Fig. 6 [file 44319_2025_487_MOESM9_ESM.zip › Figure 6/6A/PD7 Brca1-p53 vDKO testis anti-PLZF&GFRa1 Hoechst_overlay.tif]

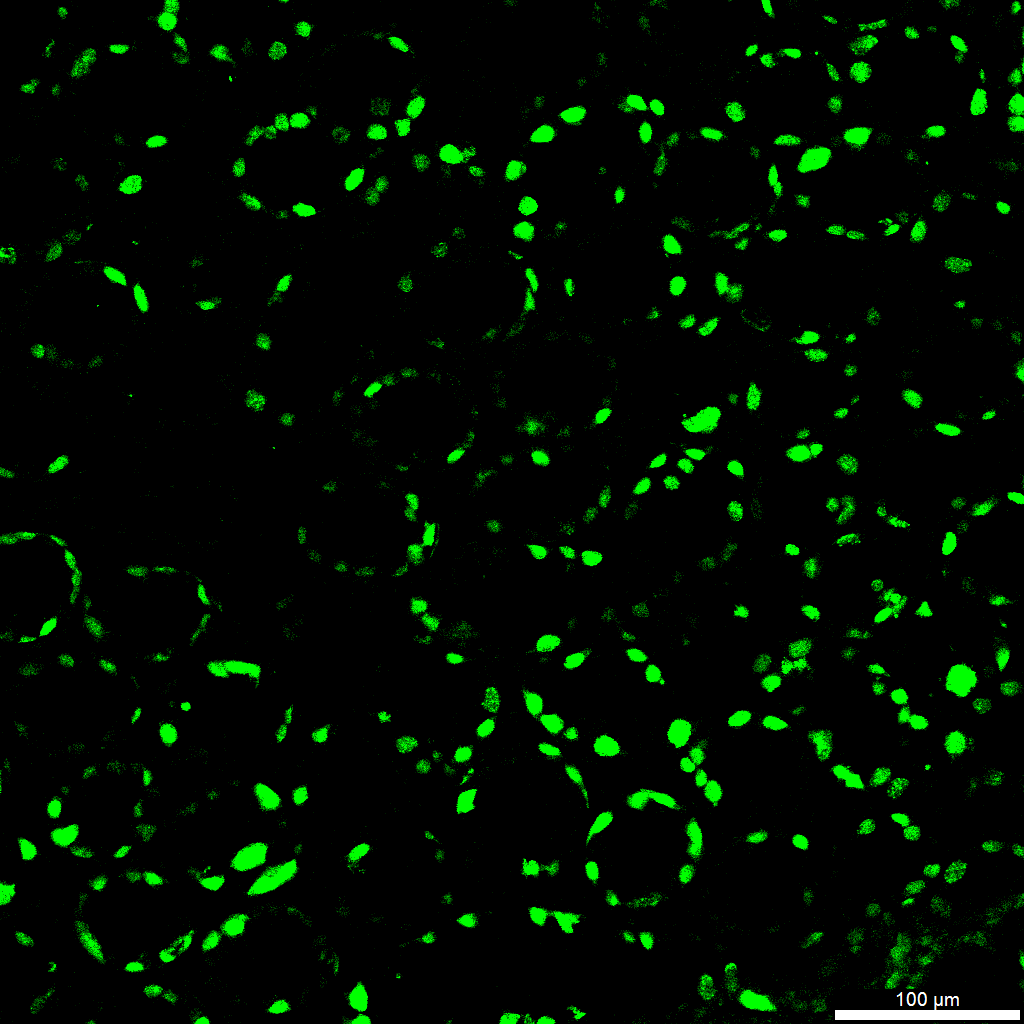

Supplement: Supplementary file 9 — Source data Fig. 6 [file 44319_2025_487_MOESM9_ESM.zip › Figure 6/6A/PD7 Brca1-p53 vDKO testis anti-PLZF.tif]

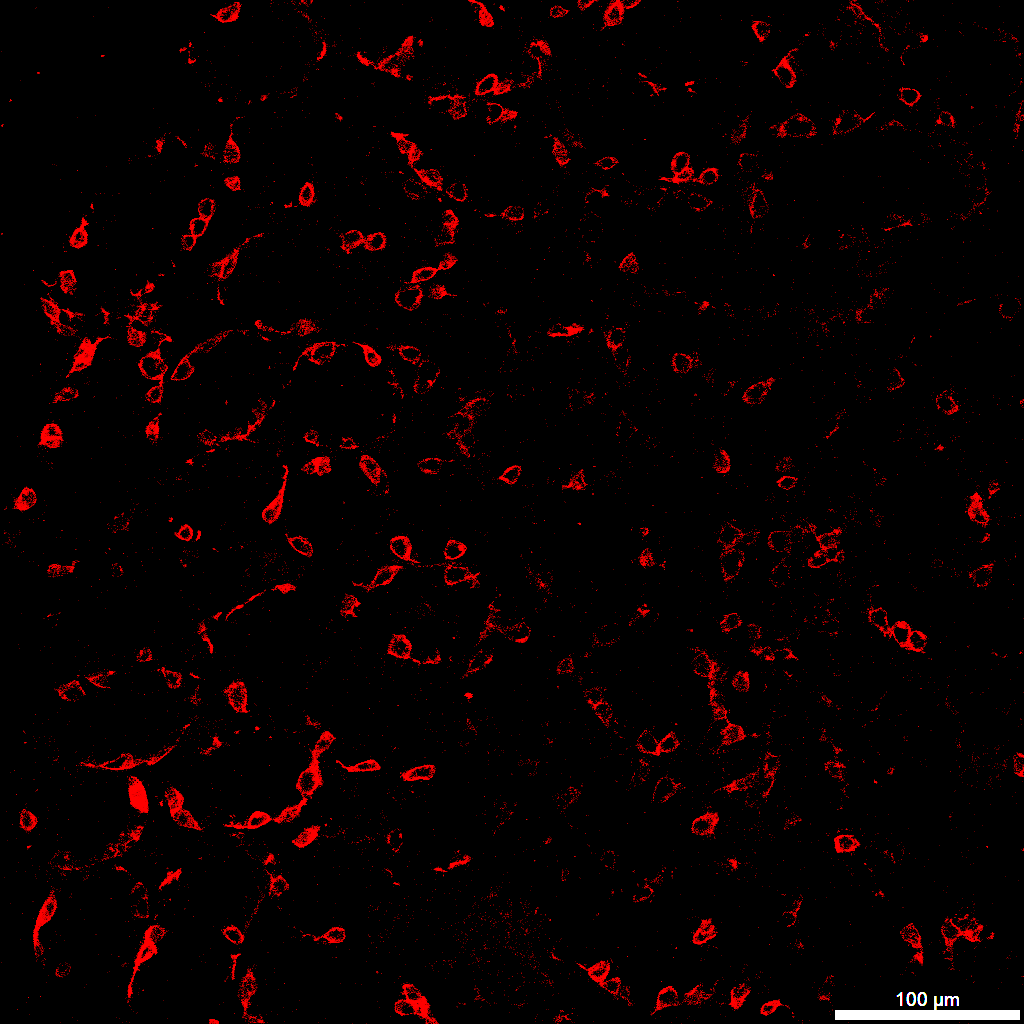

Supplement: Supplementary file 9 — Source data Fig. 6 [file 44319_2025_487_MOESM9_ESM.zip › Figure 6/6A/PD7 Control testis anti-GFRa1.tif]

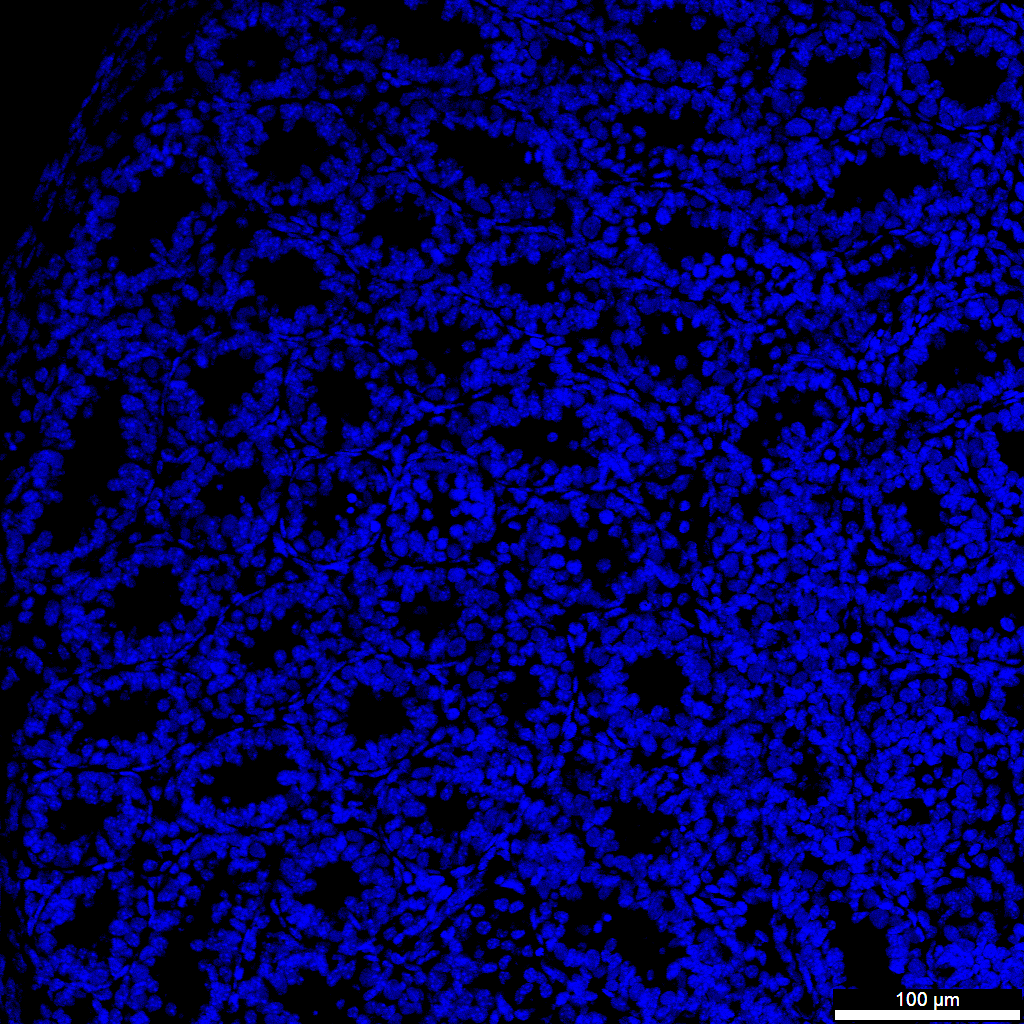

Supplement: Supplementary file 9 — Source data Fig. 6 [file 44319_2025_487_MOESM9_ESM.zip › Figure 6/6A/PD7 Control testis anti-PLZF&GFRa1 Hoechst.tif]

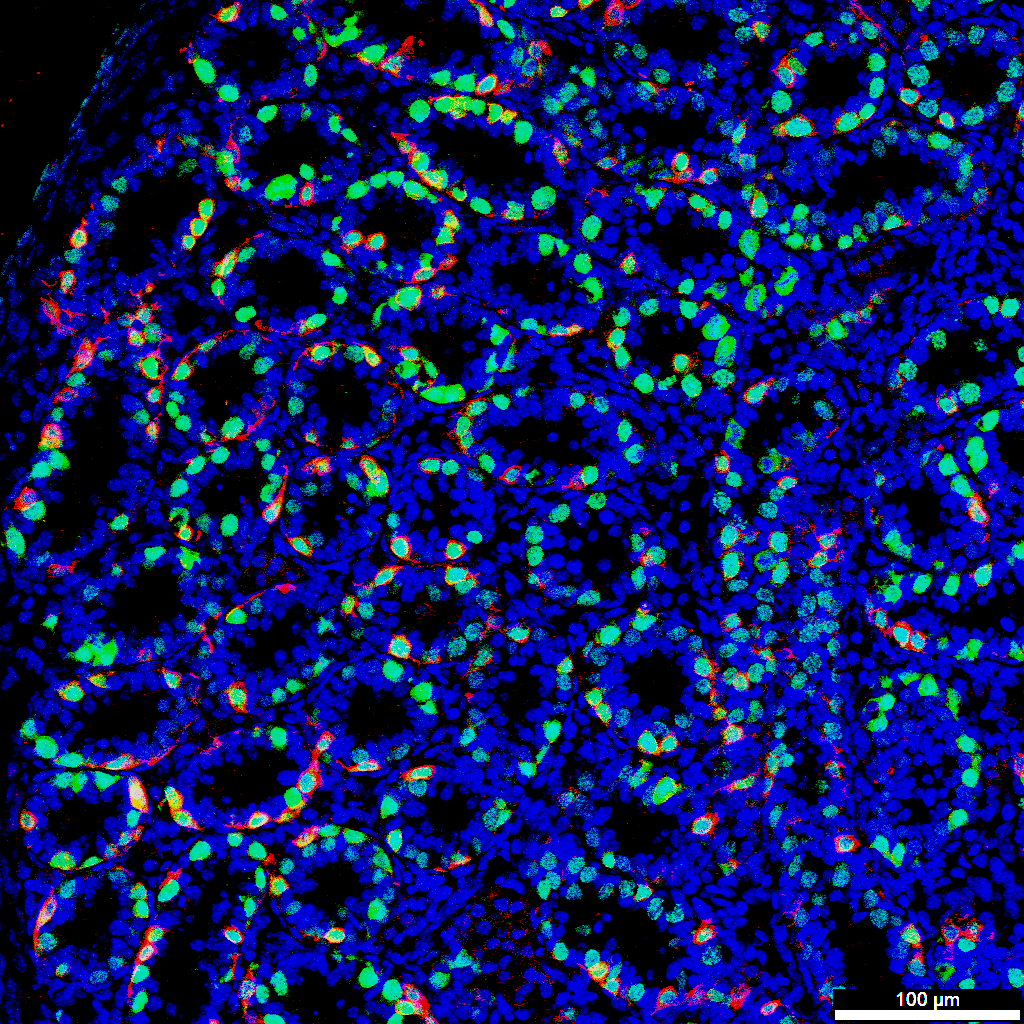

Supplement: Supplementary file 9 — Source data Fig. 6 [file 44319_2025_487_MOESM9_ESM.zip › Figure 6/6A/PD7 Control testis anti-PLZF&GFRa1 Hoechst_overlay.tif]

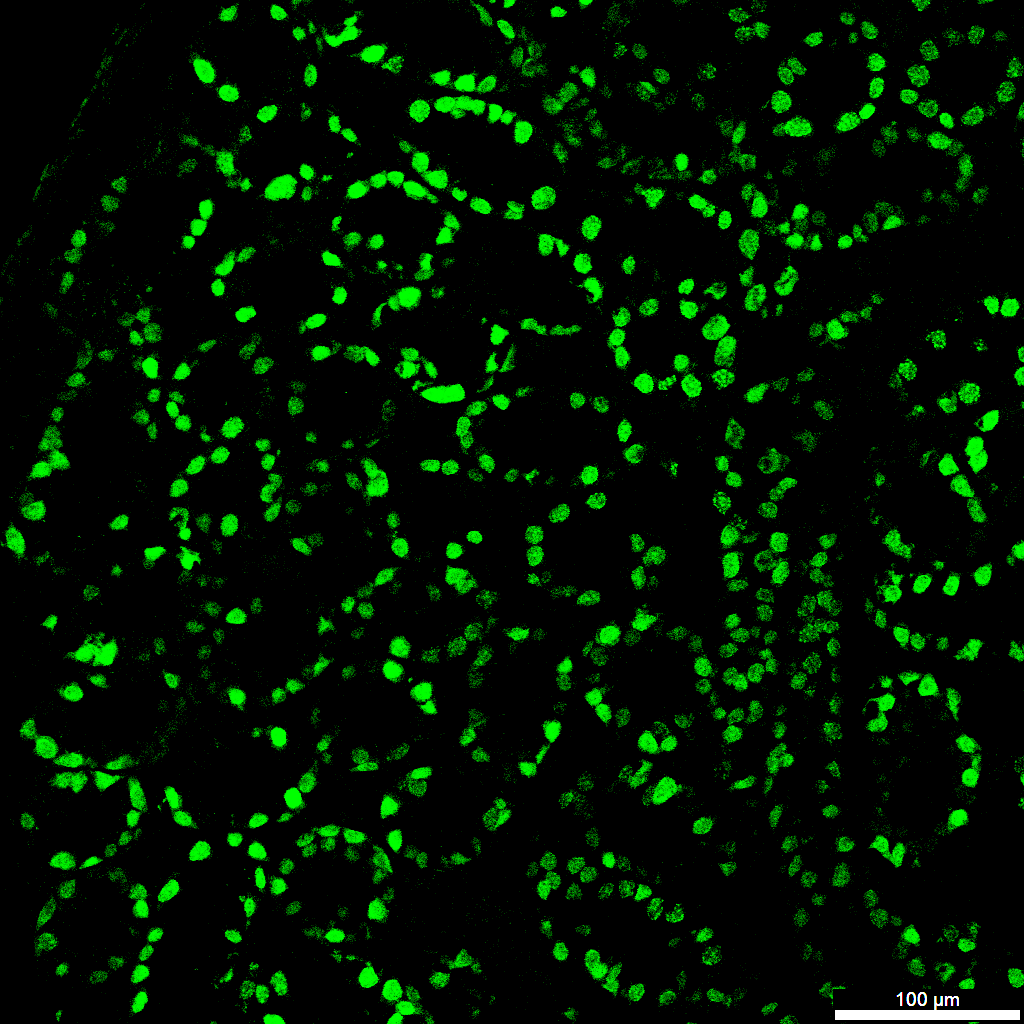

Supplement: Supplementary file 9 — Source data Fig. 6 [file 44319_2025_487_MOESM9_ESM.zip › Figure 6/6A/PD7 Control testis anti-PLZF.tif]

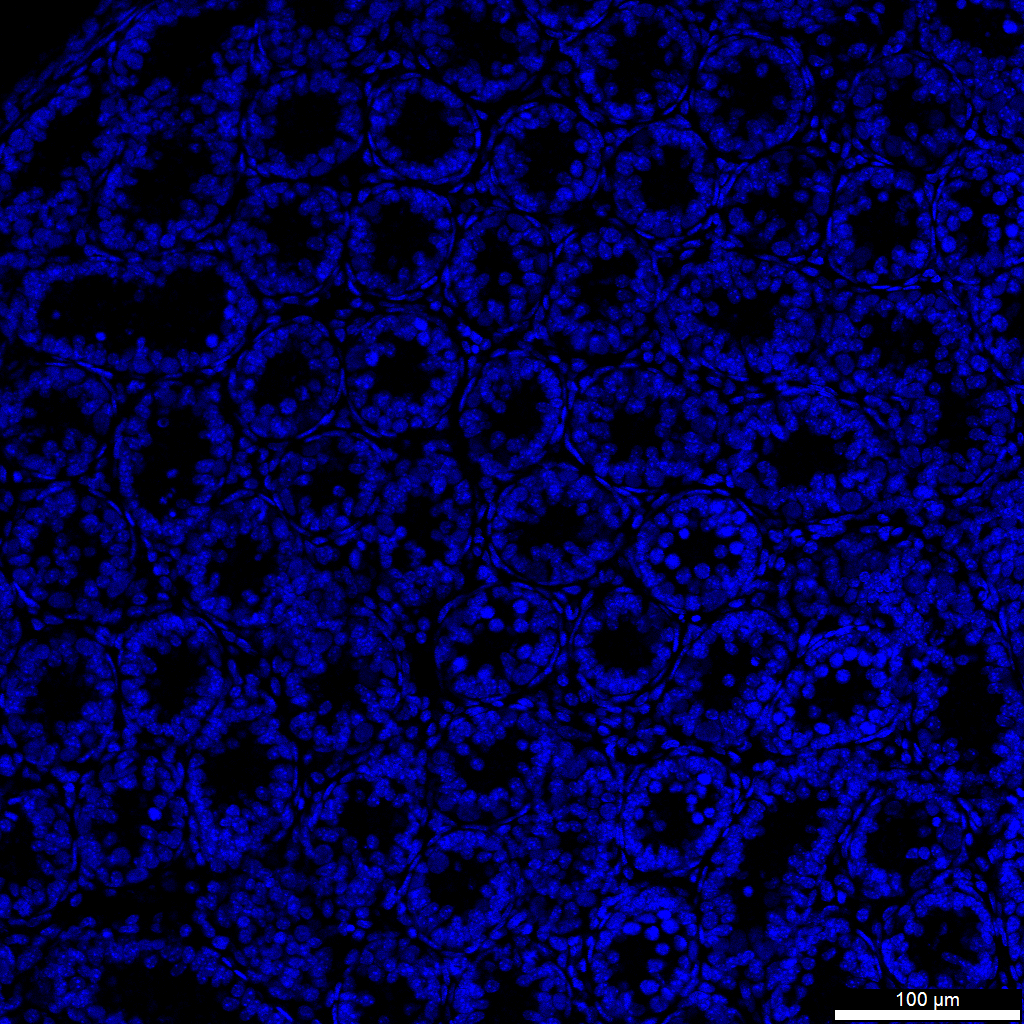

Supplement: Supplementary file 9 — Source data Fig. 6 [file 44319_2025_487_MOESM9_ESM.zip › Figure 6/6B/PD7 Brca1-p53 vDKO testis anti-PLZF&SOX3 Hoechst.tif]

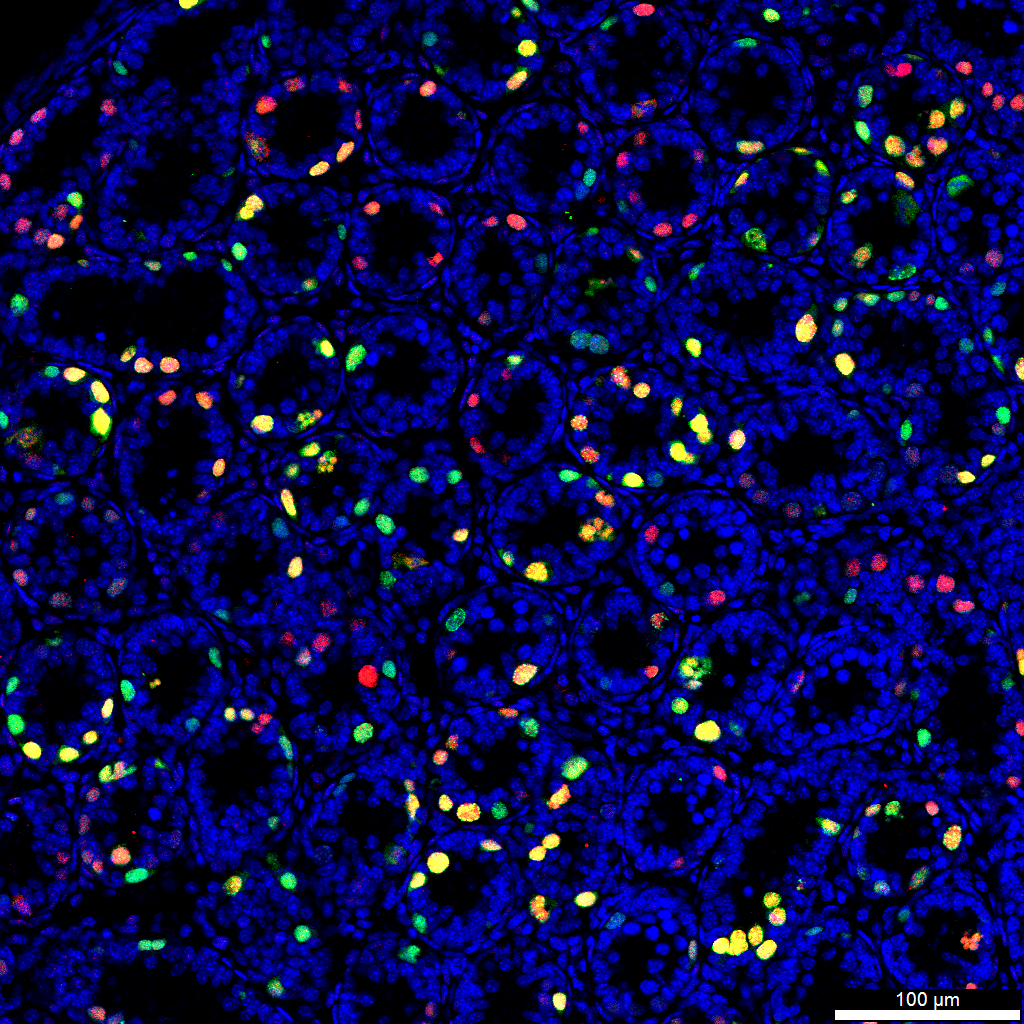

Supplement: Supplementary file 9 — Source data Fig. 6 [file 44319_2025_487_MOESM9_ESM.zip › Figure 6/6B/PD7 Brca1-p53 vDKO testis anti-PLZF&SOX3 Hoechst_overlay.tif]

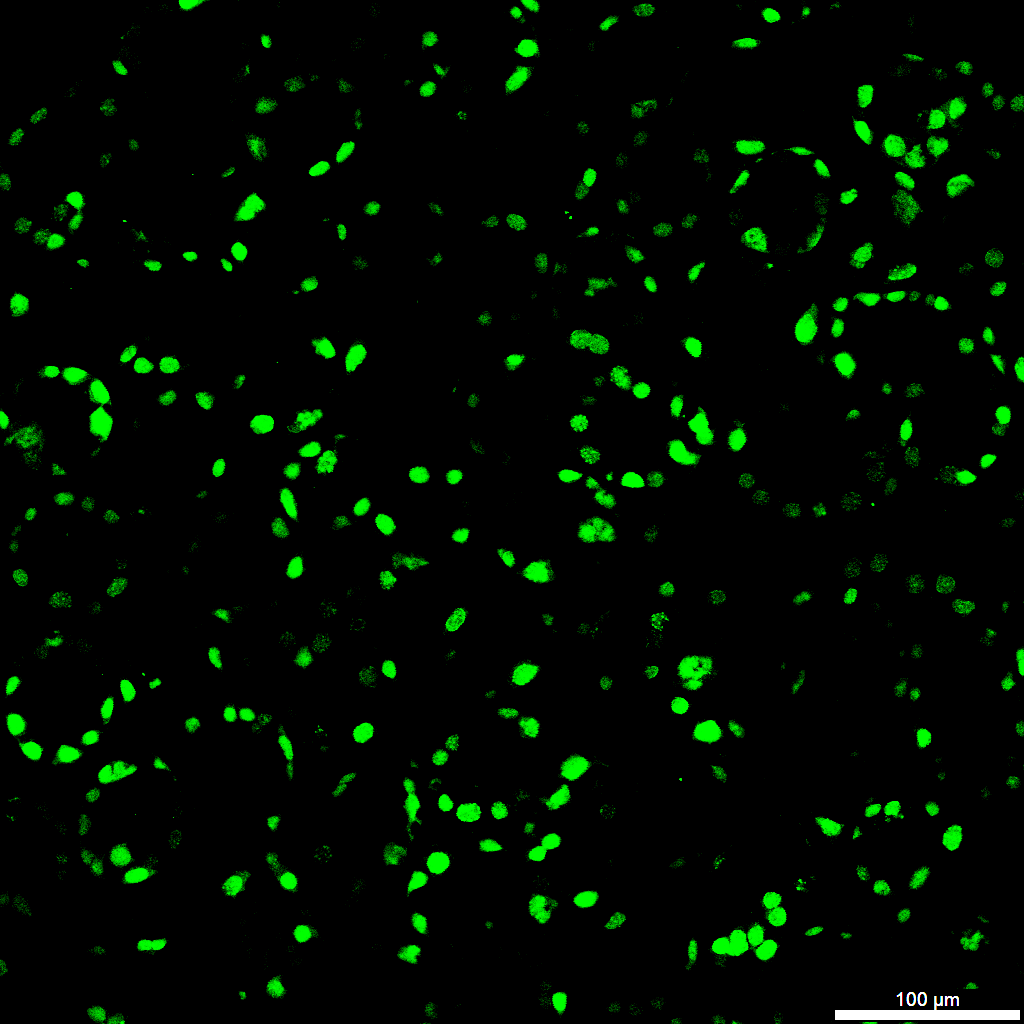

Supplement: Supplementary file 9 — Source data Fig. 6 [file 44319_2025_487_MOESM9_ESM.zip › Figure 6/6B/PD7 Brca1-p53 vDKO testis anti-PLZF.tif]

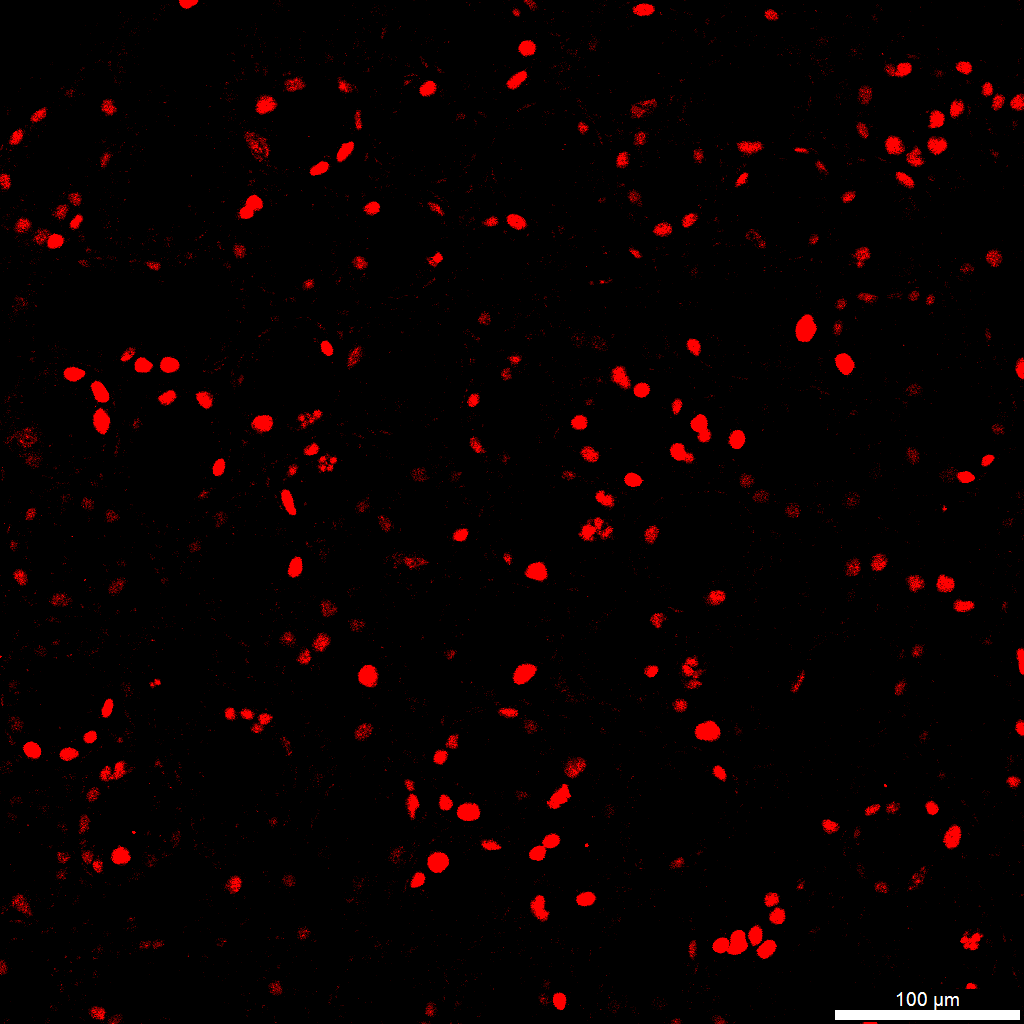

Supplement: Supplementary file 9 — Source data Fig. 6 [file 44319_2025_487_MOESM9_ESM.zip › Figure 6/6B/PD7 Brca1-p53 vDKO testis anti-SOX3.tif]

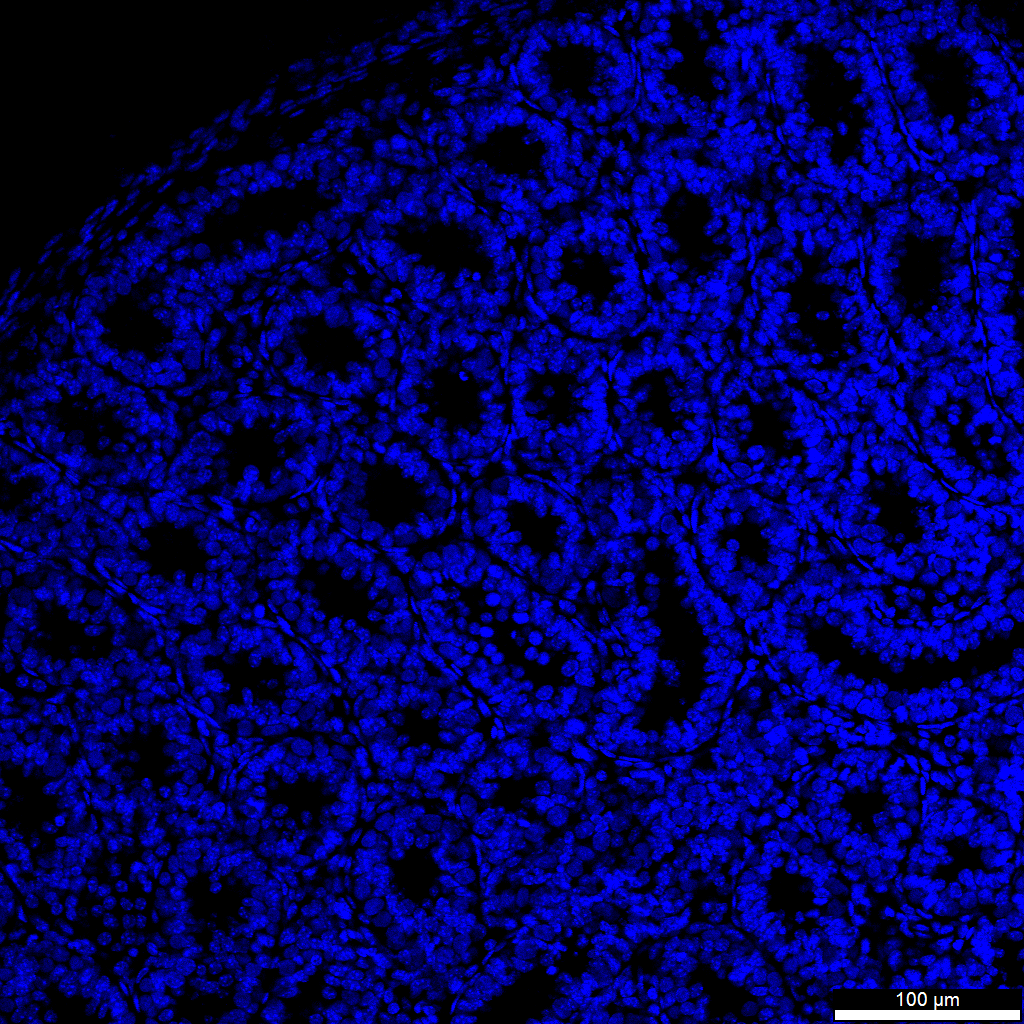

Supplement: Supplementary file 9 — Source data Fig. 6 [file 44319_2025_487_MOESM9_ESM.zip › Figure 6/6B/PD7 Control testis anti-PLZF&SOX3 Hoechst.tif]

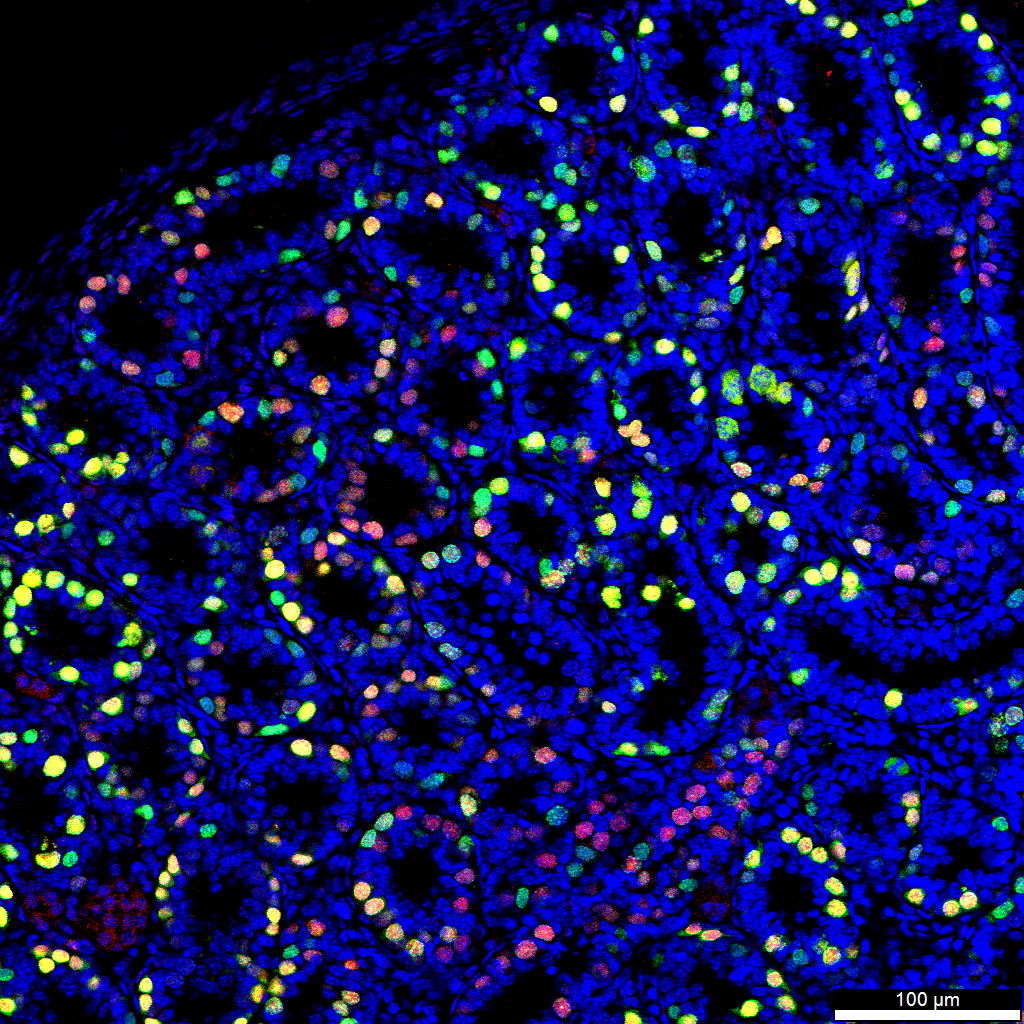

Supplement: Supplementary file 9 — Source data Fig. 6 [file 44319_2025_487_MOESM9_ESM.zip › Figure 6/6B/PD7 Control testis anti-PLZF&SOX3 Hoechst_overlay.tif]

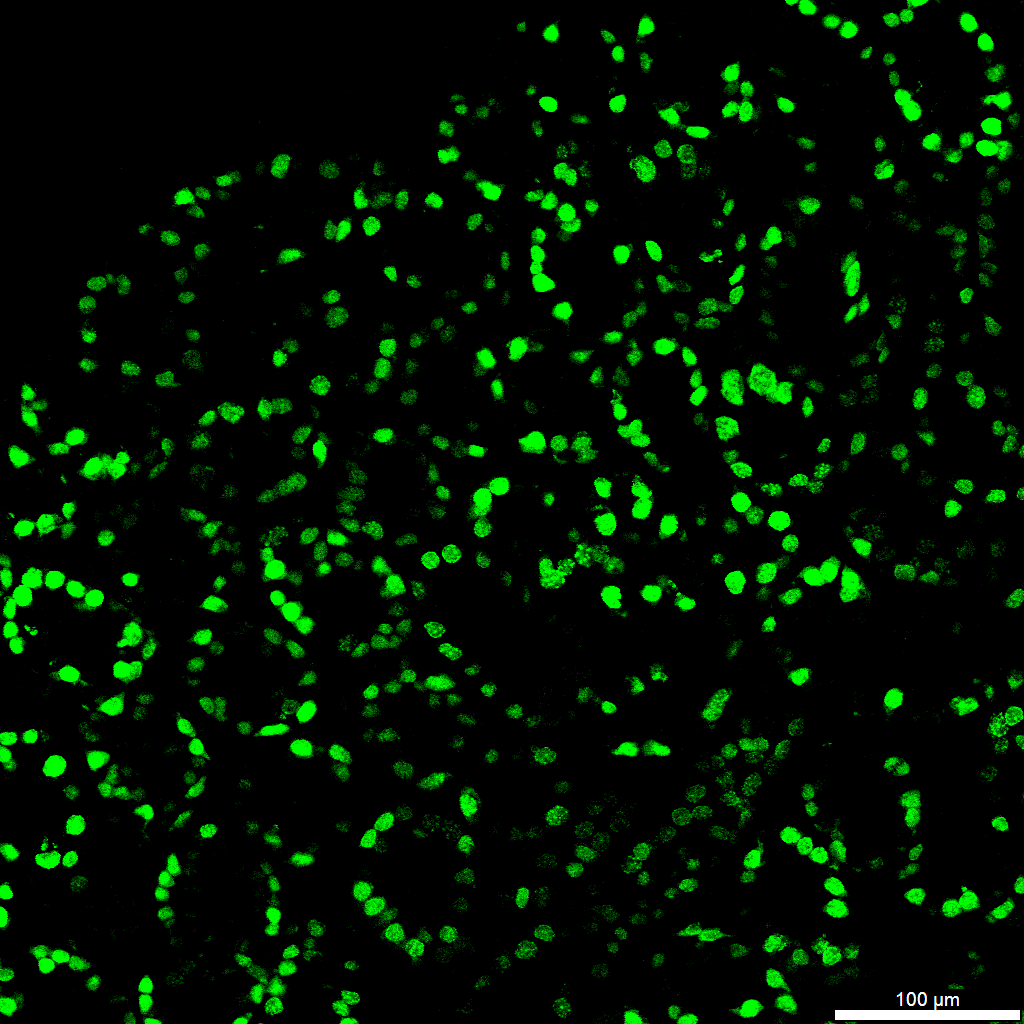

Supplement: Supplementary file 9 — Source data Fig. 6 [file 44319_2025_487_MOESM9_ESM.zip › Figure 6/6B/PD7 Control testis anti-PLZF.tif]

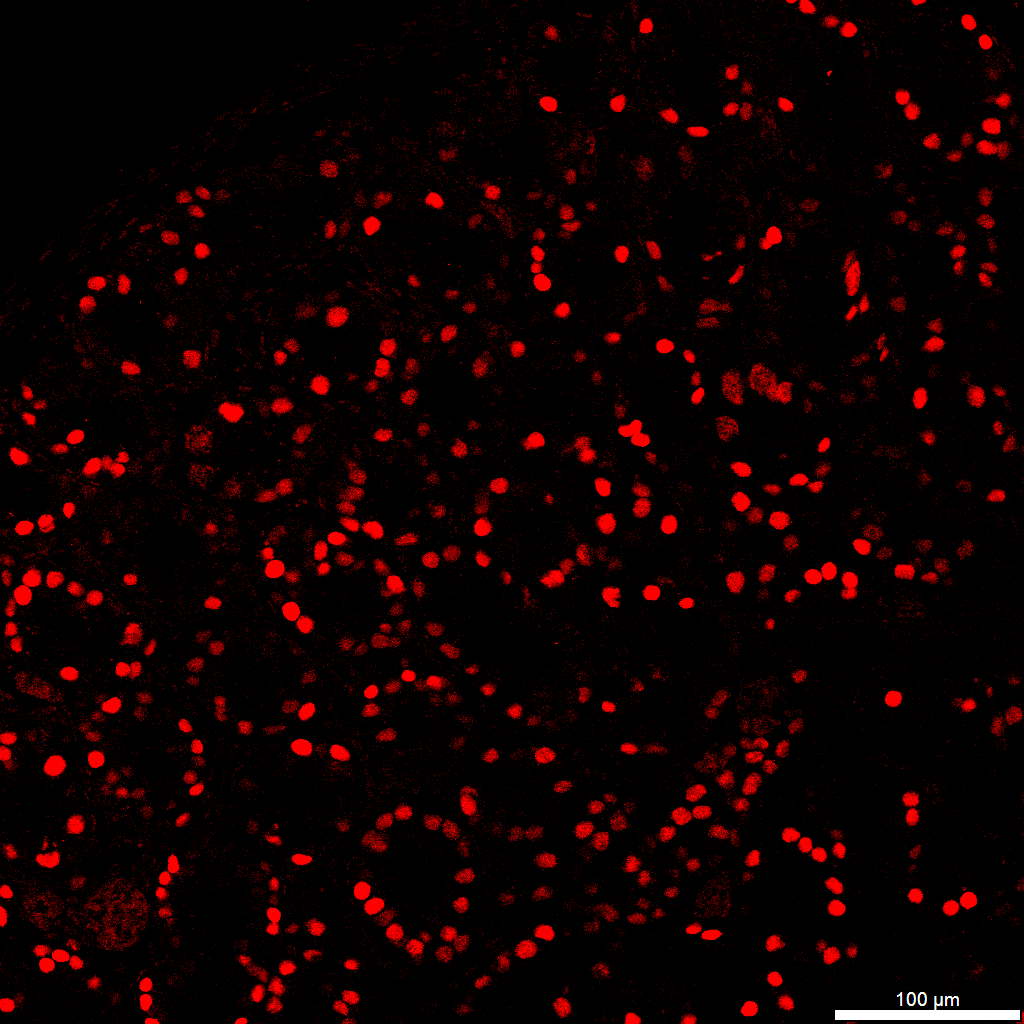

Supplement: Supplementary file 9 — Source data Fig. 6 [file 44319_2025_487_MOESM9_ESM.zip › Figure 6/6B/PD7 Control testis anti-SOX3.tif]

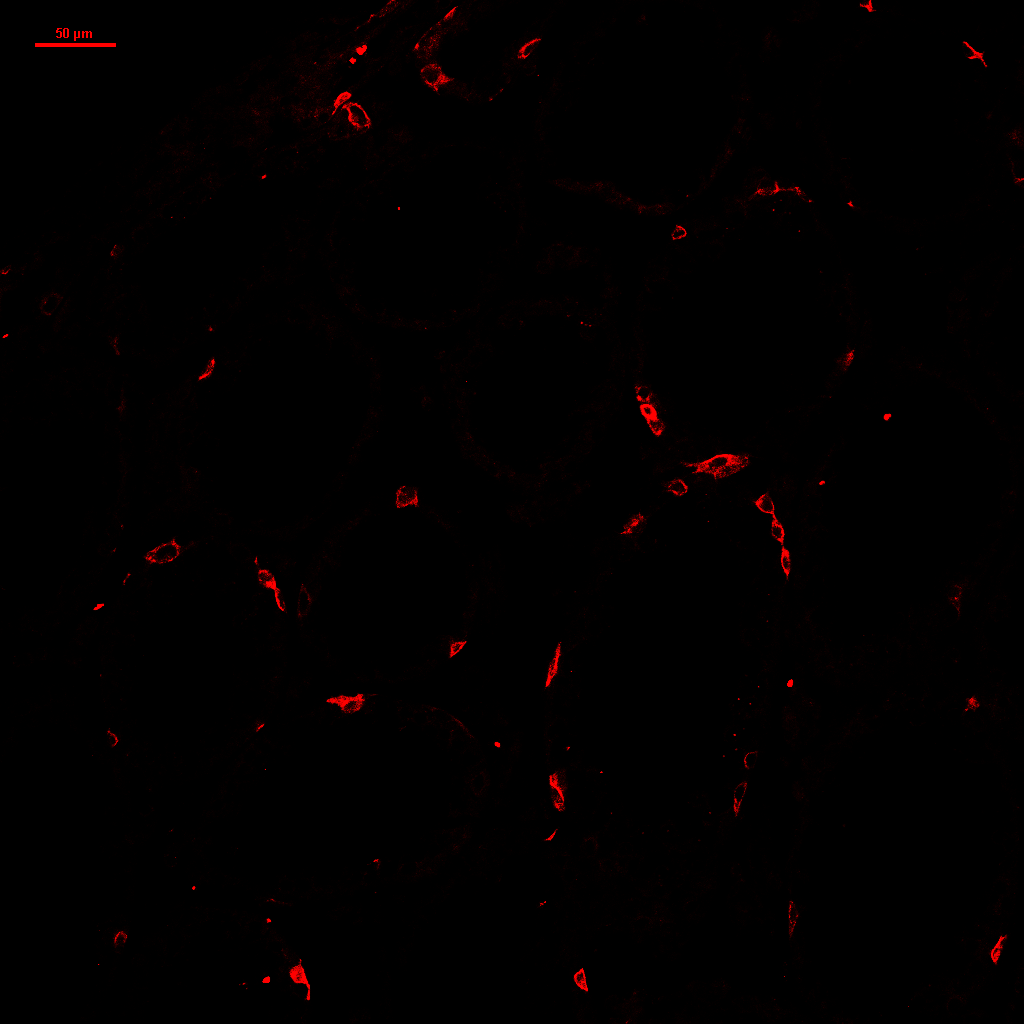

Supplement: Supplementary file 9 — Source data Fig. 6 [file 44319_2025_487_MOESM9_ESM.zip › Figure 6/6E/PD21 Brca1-p53 vDKO testis anti-GFRa1.tif]

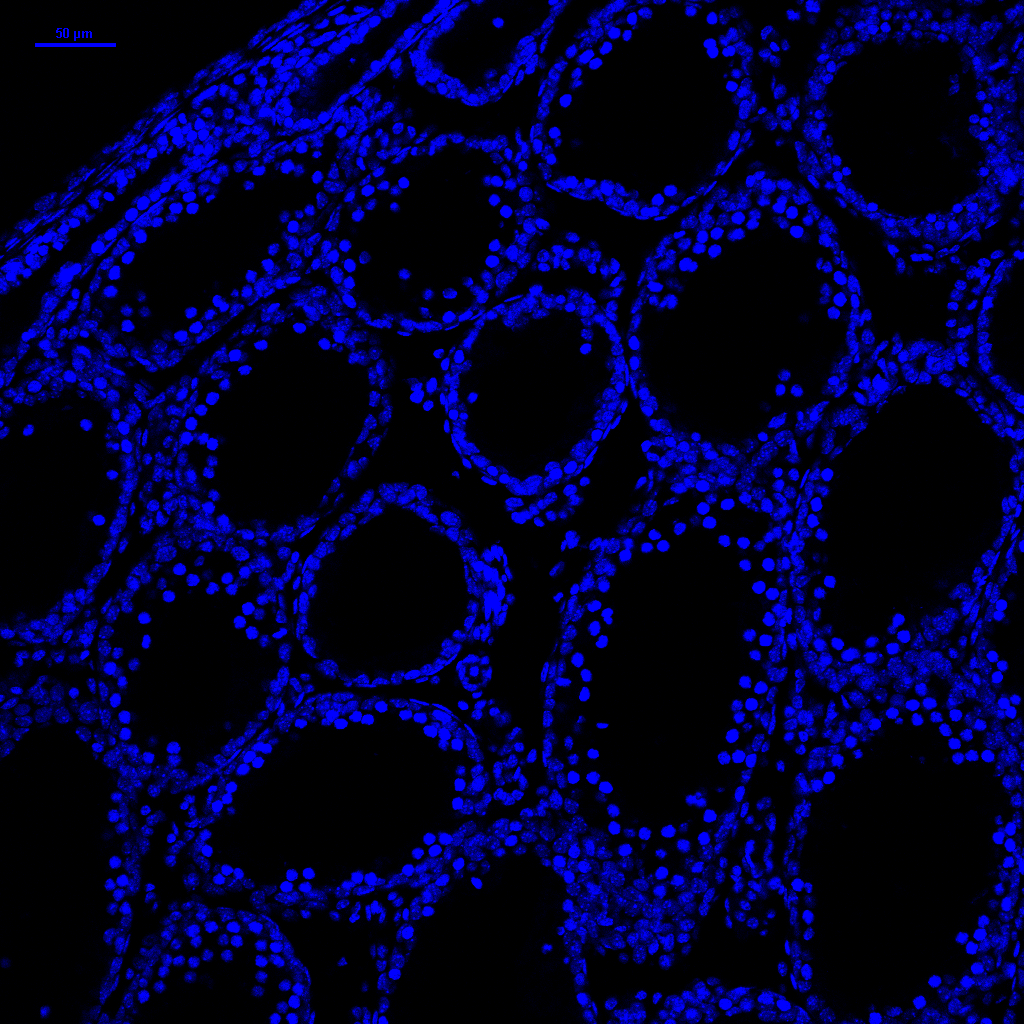

Supplement: Supplementary file 9 — Source data Fig. 6 [file 44319_2025_487_MOESM9_ESM.zip › Figure 6/6E/PD21 Brca1-p53 vDKO testis anti-PLZF&GFRa1 Hoechst.tif]

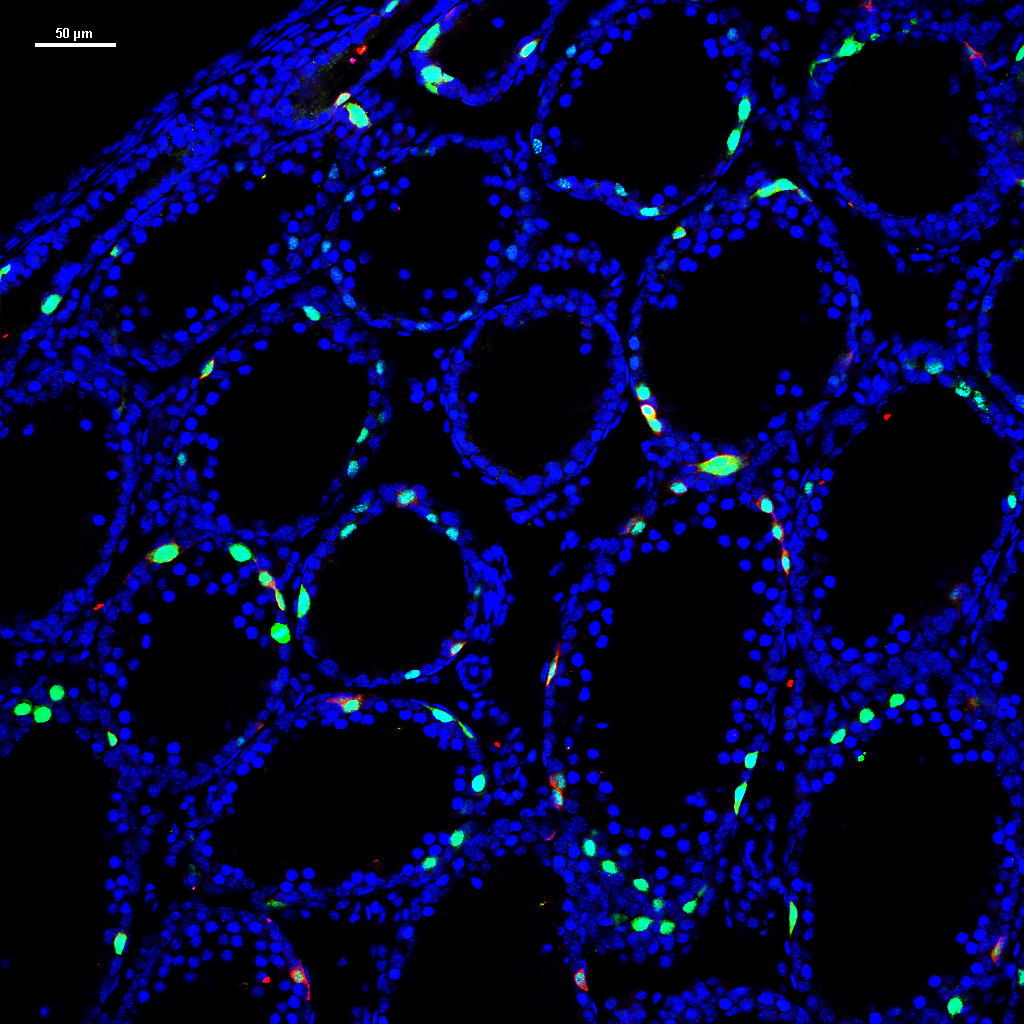

Supplement: Supplementary file 9 — Source data Fig. 6 [file 44319_2025_487_MOESM9_ESM.zip › Figure 6/6E/PD21 Brca1-p53 vDKO testis anti-PLZF&GFRa1 Hoechst_overlay.tif]

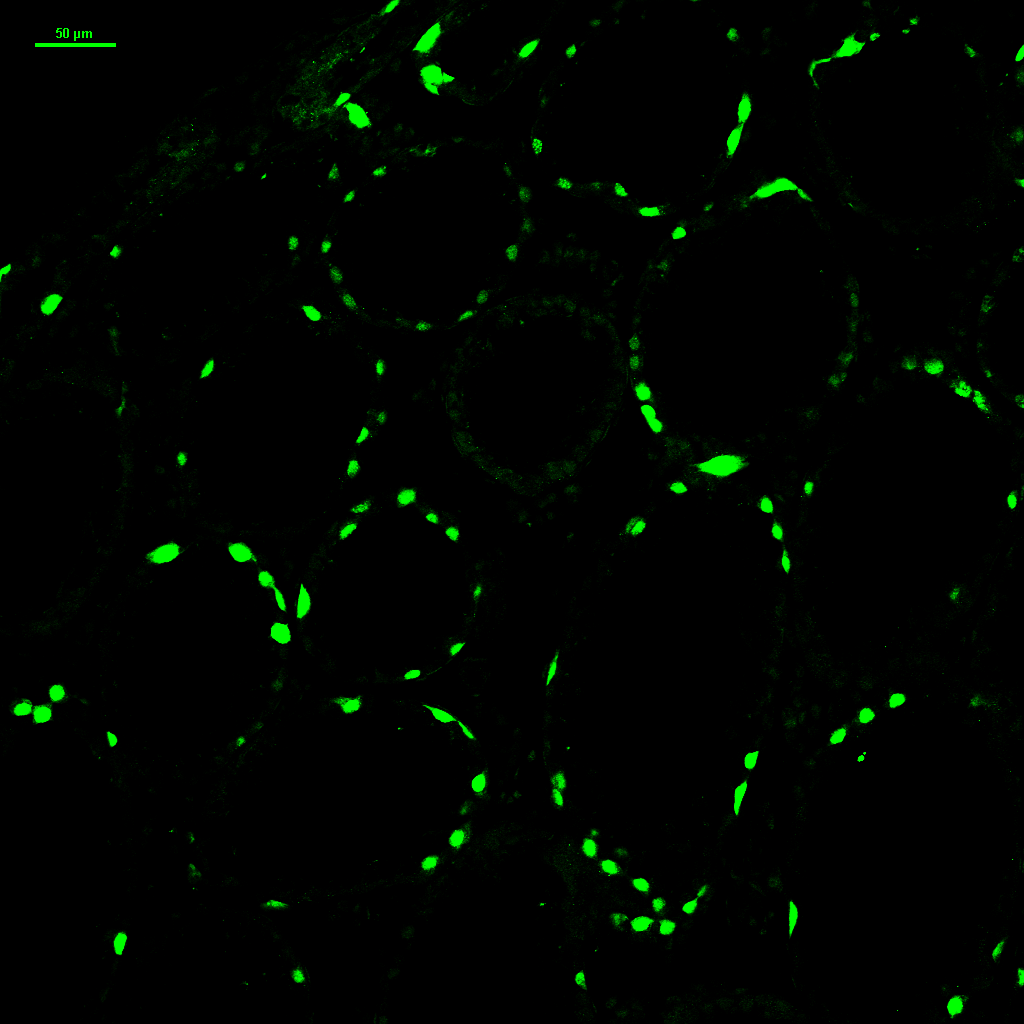

Supplement: Supplementary file 9 — Source data Fig. 6 [file 44319_2025_487_MOESM9_ESM.zip › Figure 6/6E/PD21 Brca1-p53 vDKO testis anti-PLZF.tif]

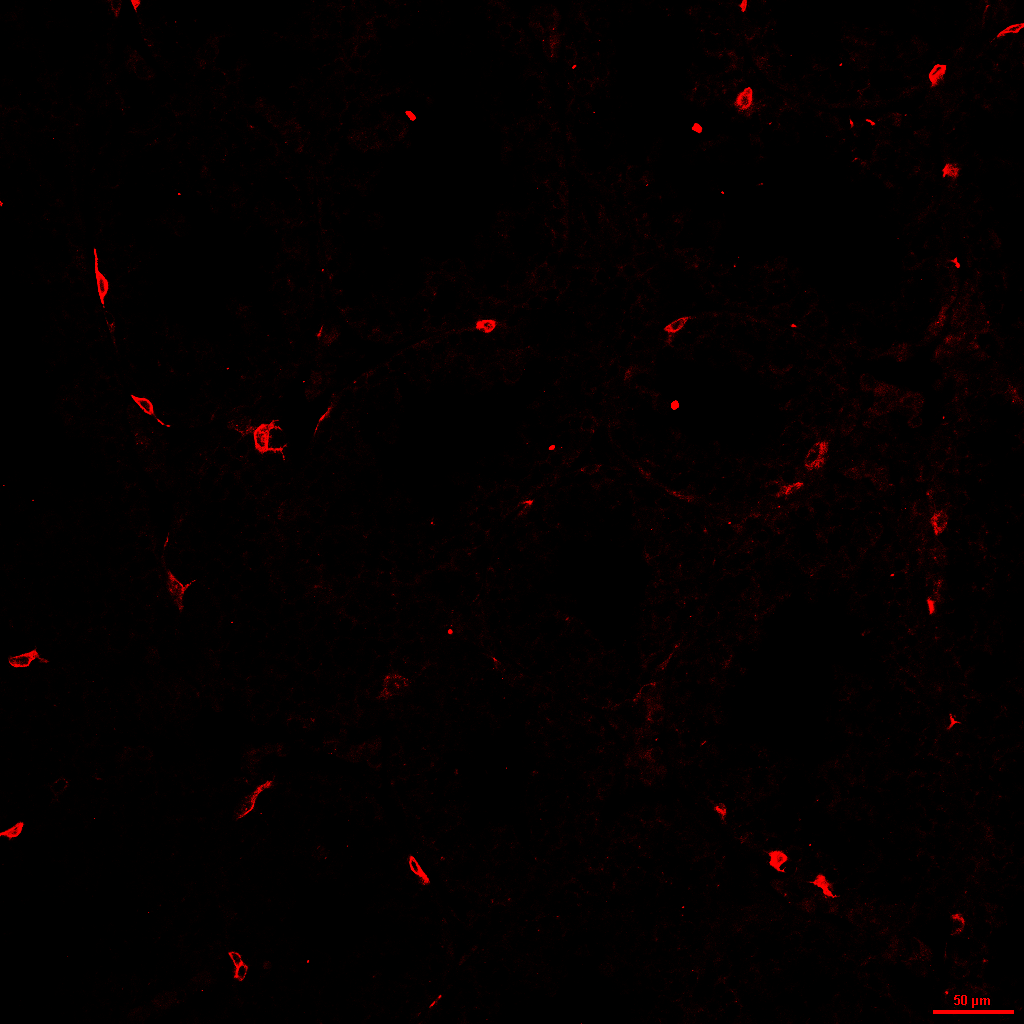

Supplement: Supplementary file 9 — Source data Fig. 6 [file 44319_2025_487_MOESM9_ESM.zip › Figure 6/6E/PD21 Control testis anti-GFRa1.tif]

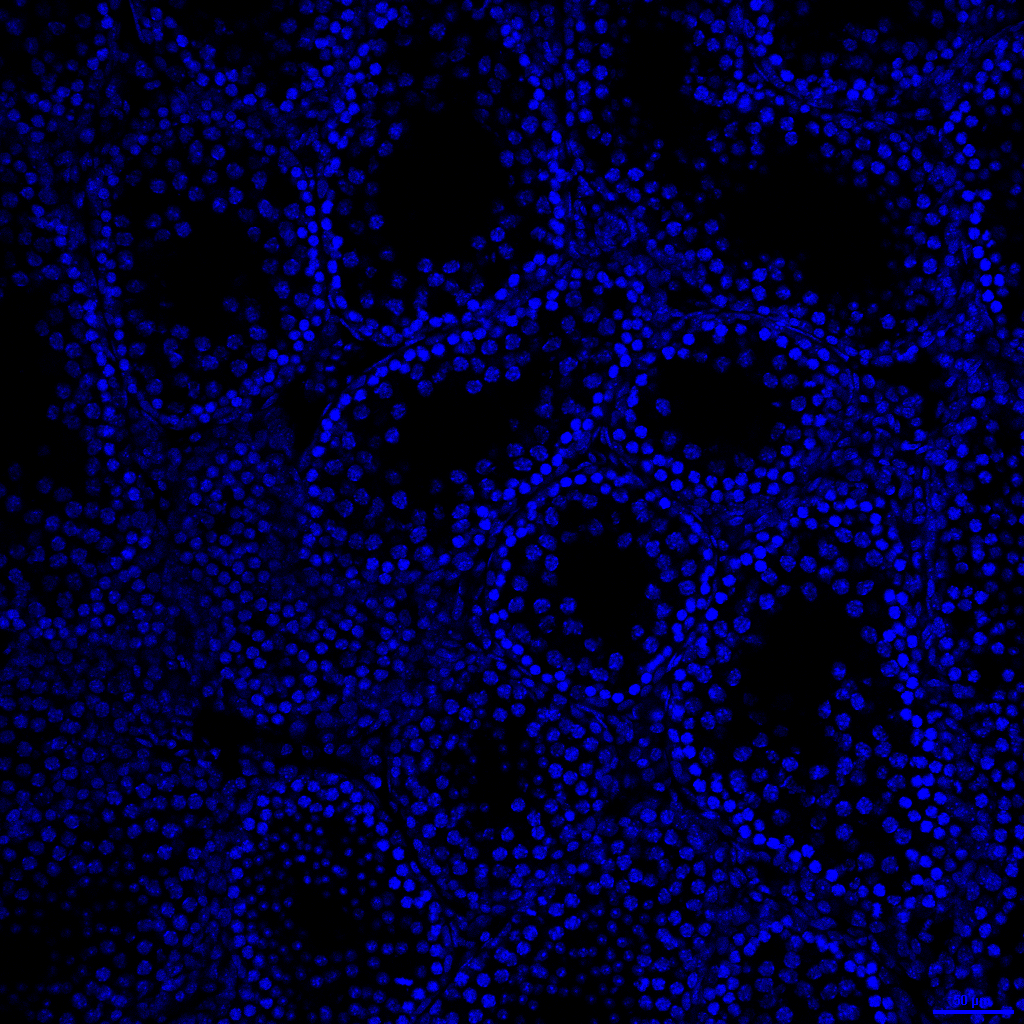

Supplement: Supplementary file 9 — Source data Fig. 6 [file 44319_2025_487_MOESM9_ESM.zip › Figure 6/6E/PD21 Control testis anti-PLZF&GFRa1 Hoechst.tif]

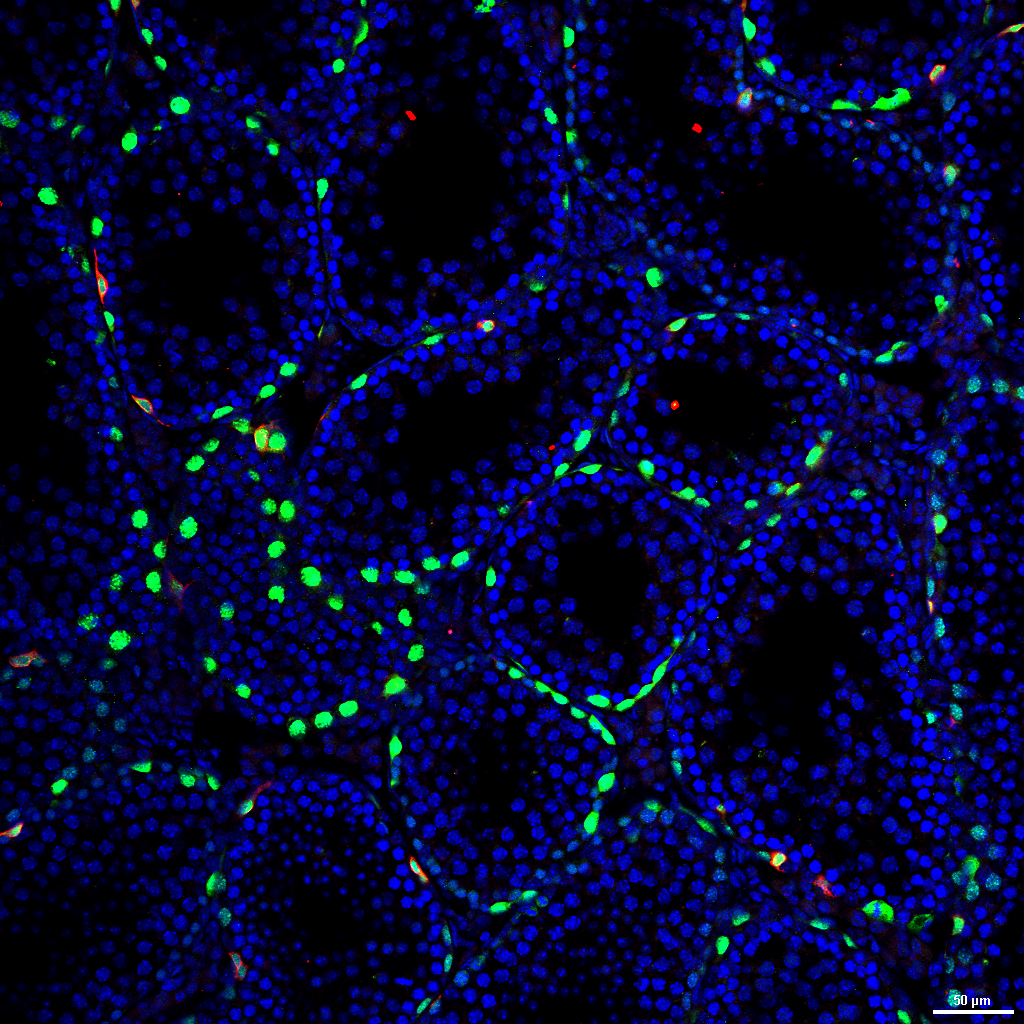

Supplement: Supplementary file 9 — Source data Fig. 6 [file 44319_2025_487_MOESM9_ESM.zip › Figure 6/6E/PD21 Control testis anti-PLZF&GFRa1 Hoechst_overlay.tif]

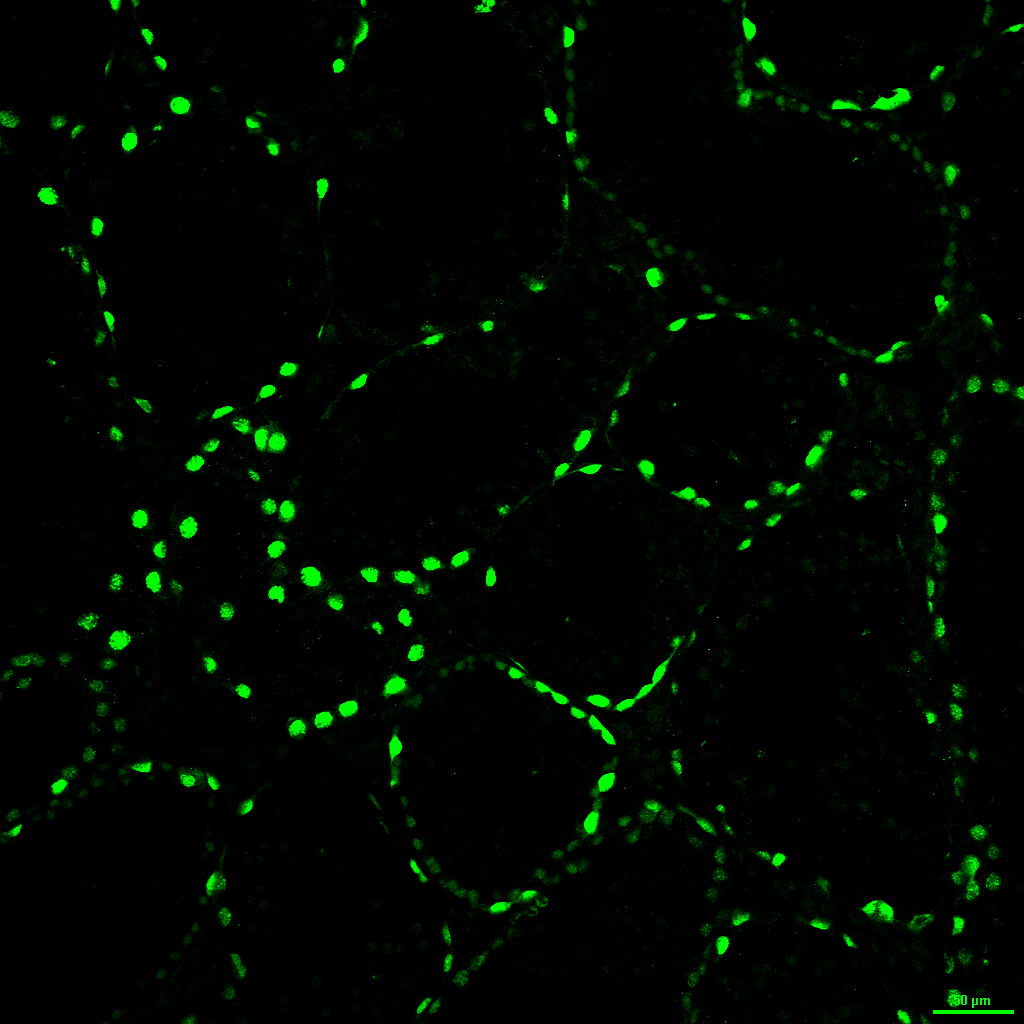

Supplement: Supplementary file 9 — Source data Fig. 6 [file 44319_2025_487_MOESM9_ESM.zip › Figure 6/6E/PD21 Control testis anti-PLZF.tif]

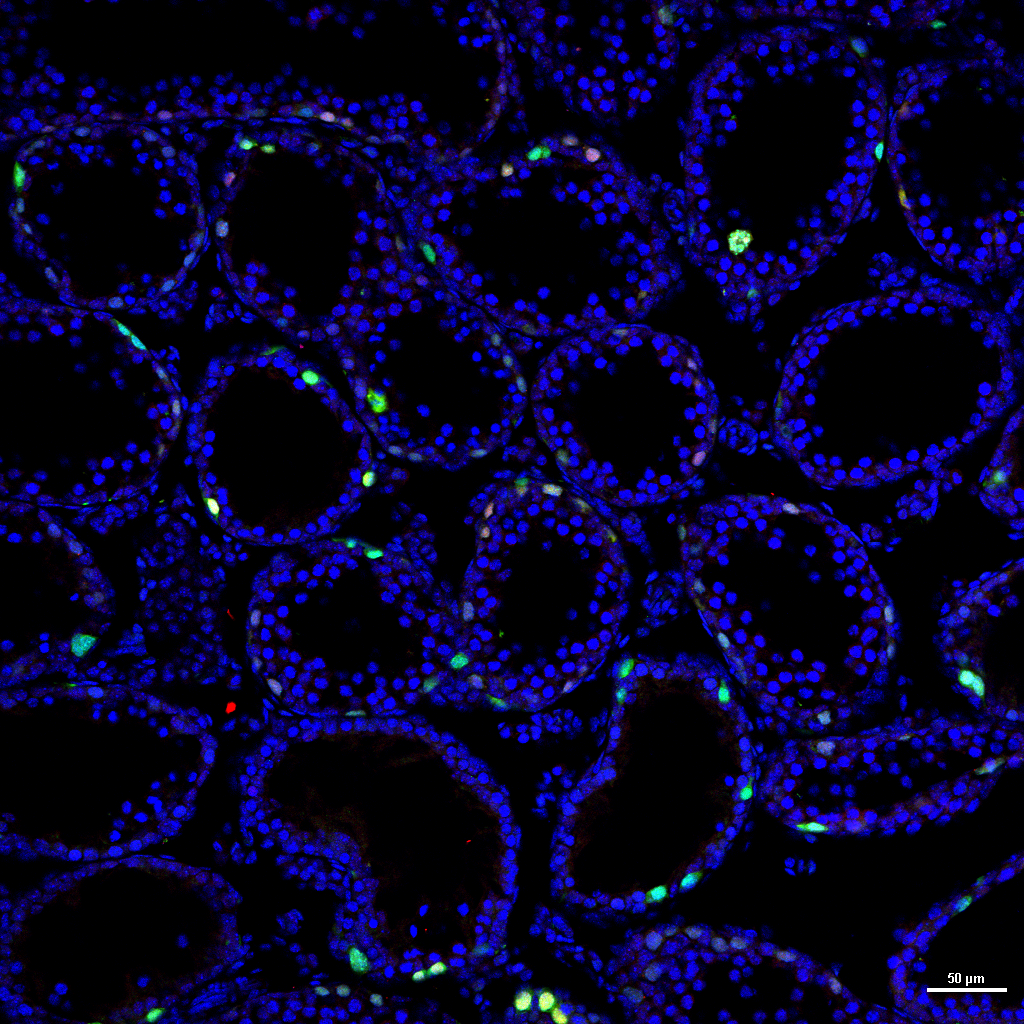

Supplement: Supplementary file 9 — Source data Fig. 6 [file 44319_2025_487_MOESM9_ESM.zip › Figure 6/6F/3wk BRCA1 vasa-cre p53 041619 VDKO anti-PLZF&SOX3 20x-1.tif]

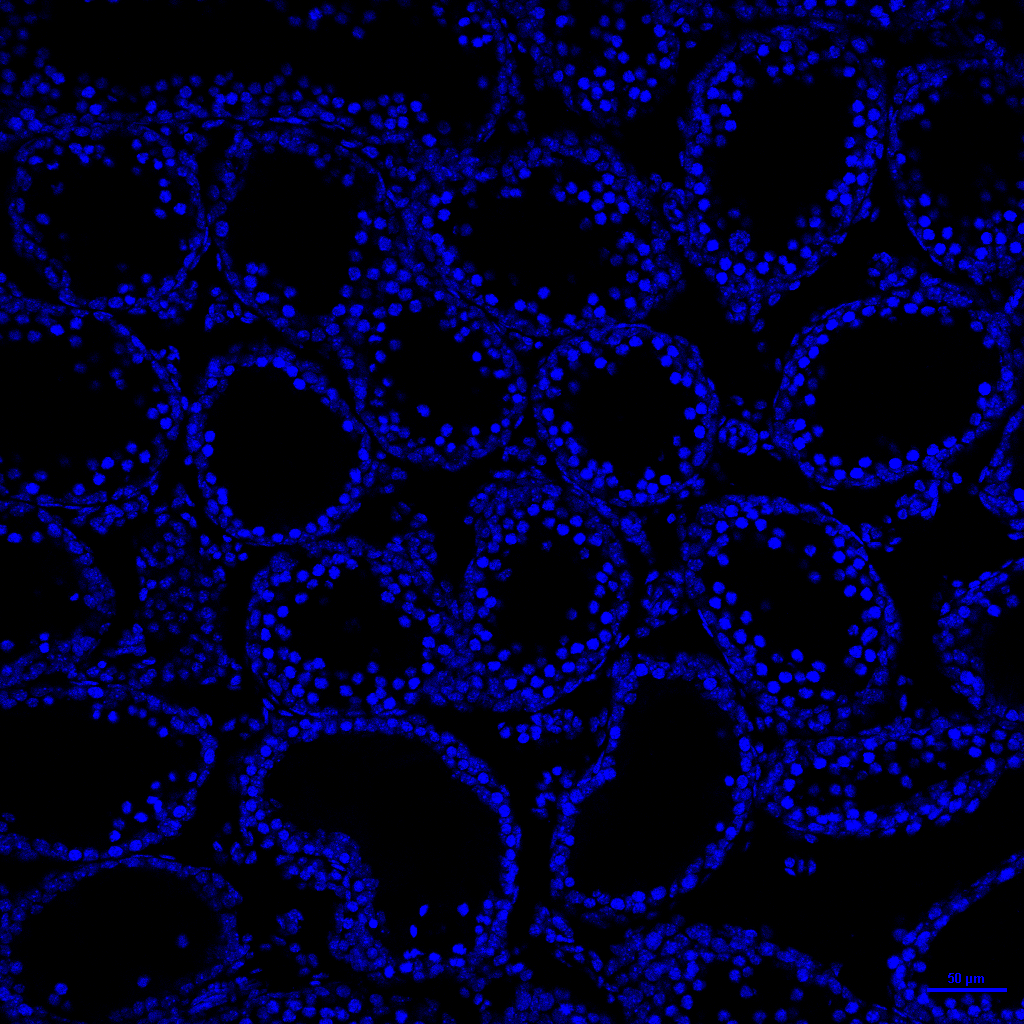

Supplement: Supplementary file 9 — Source data Fig. 6 [file 44319_2025_487_MOESM9_ESM.zip › Figure 6/6F/3wk BRCA1 vasa-cre p53 041619 VDKO anti-PLZF&SOX3 20x-1c1.tif]

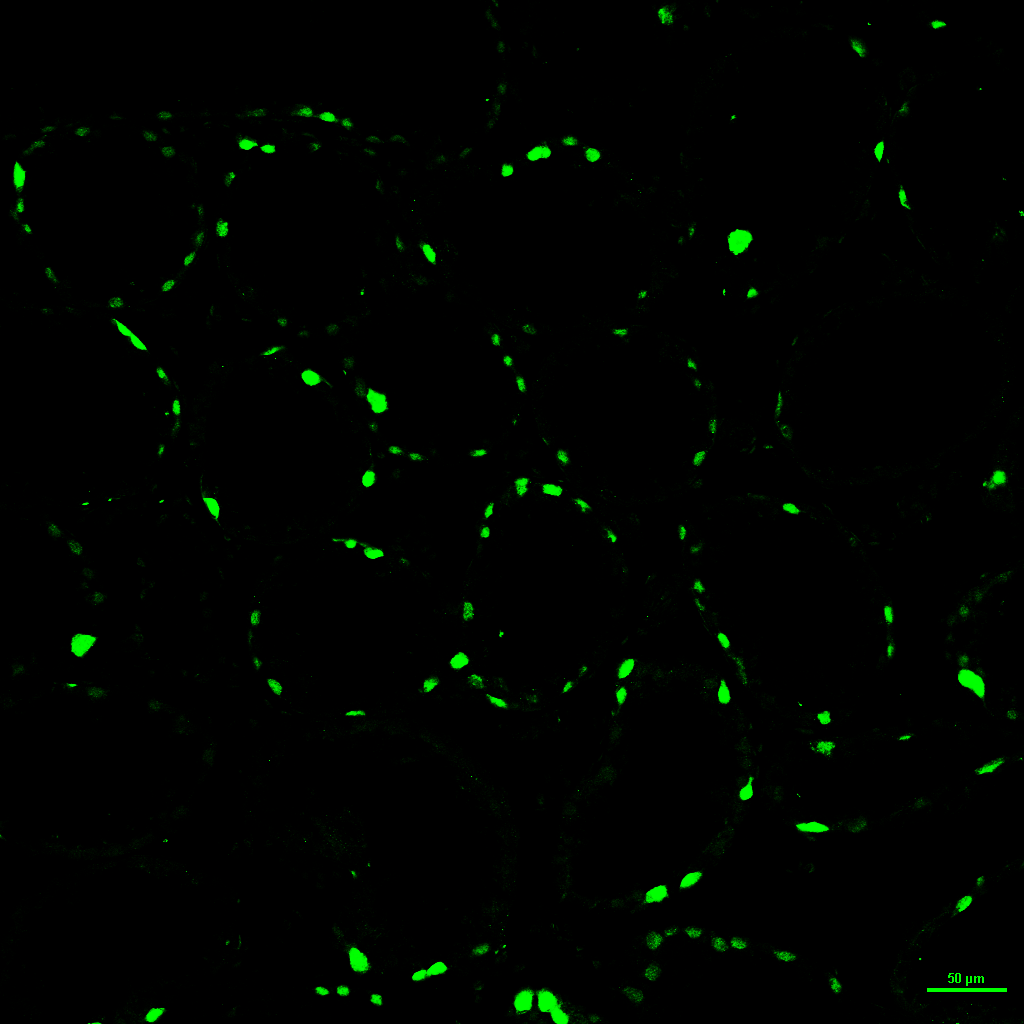

Supplement: Supplementary file 9 — Source data Fig. 6 [file 44319_2025_487_MOESM9_ESM.zip › Figure 6/6F/3wk BRCA1 vasa-cre p53 041619 VDKO anti-PLZF&SOX3 20x-1c2.tif]
