# Supplementary figures and images for: BRCA1 preserves genome integrity during the formation of undifferentiated spermatogonia (part 3 of 3)
Source: EMBO Rep. 2025 May 28;26(15):3747–72. doi: 10.1038/s44319-025-00487-5 (PMC12332178; doi:10.1038/s44319-025-00487-5)

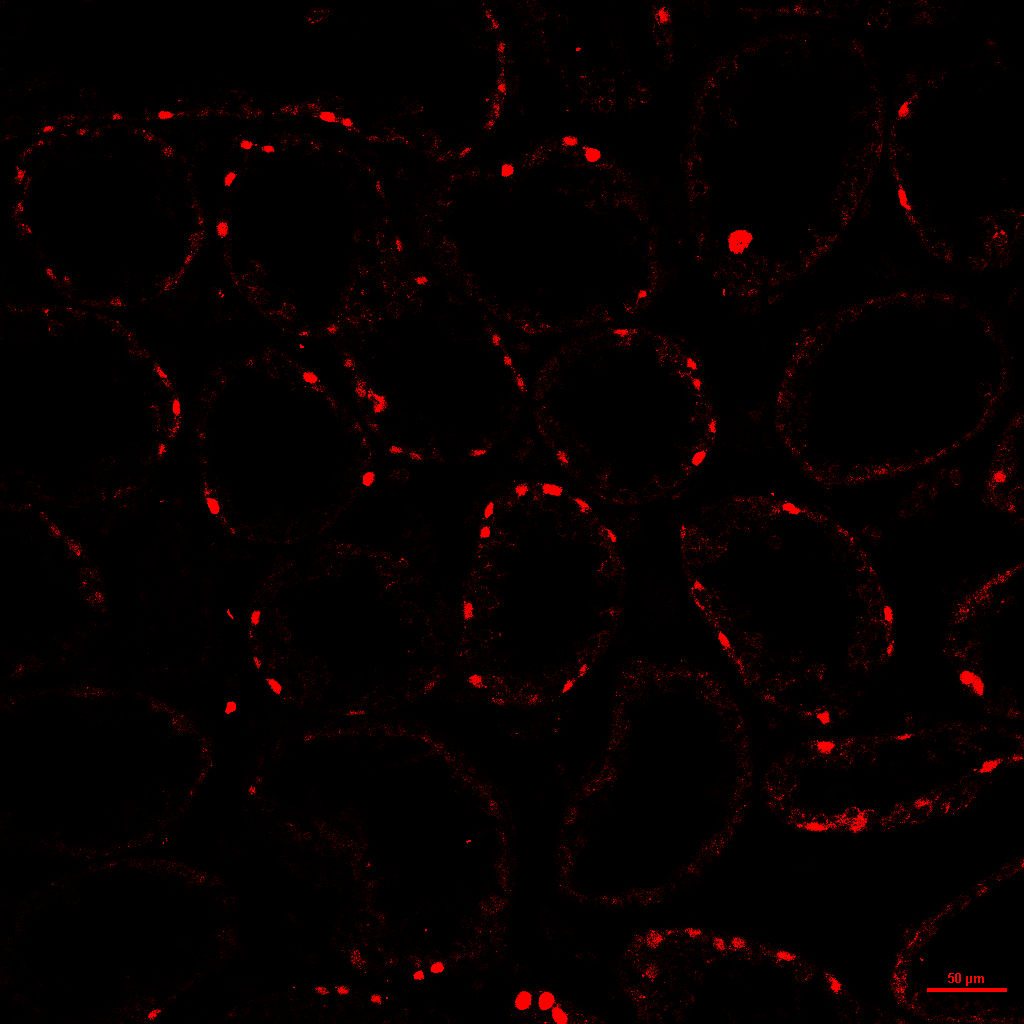

Supplement: Supplementary file 9 — Source data Fig. 6 [file 44319_2025_487_MOESM9_ESM.zip › Figure 6/6F/3wk BRCA1 vasa-cre p53 VDKO anti-PLZF&SOX3 20x-1c3.tif]

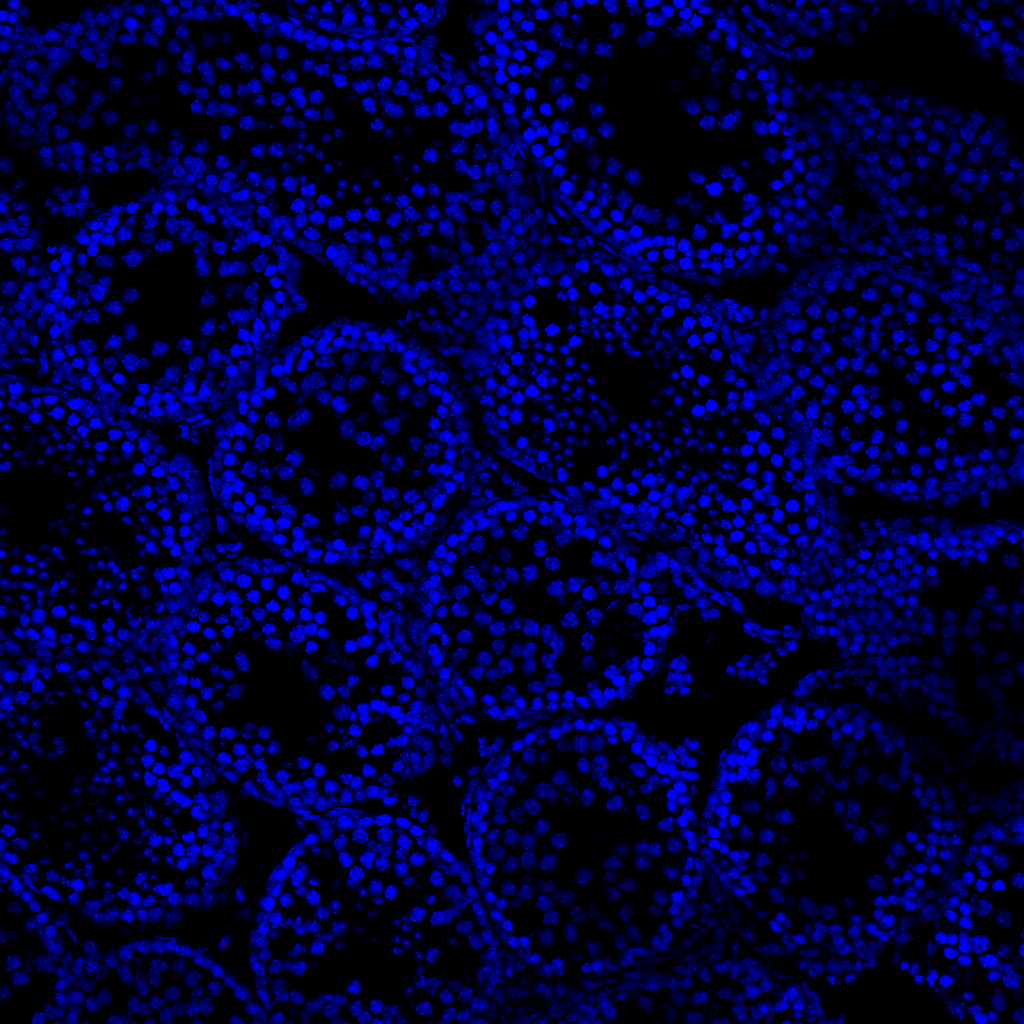

Supplement: Supplementary file 9 — Source data Fig. 6 [file 44319_2025_487_MOESM9_ESM.zip › Figure 6/6F/PD21 Control testis anti-PLZF&SOX3 Hoechst.tif]

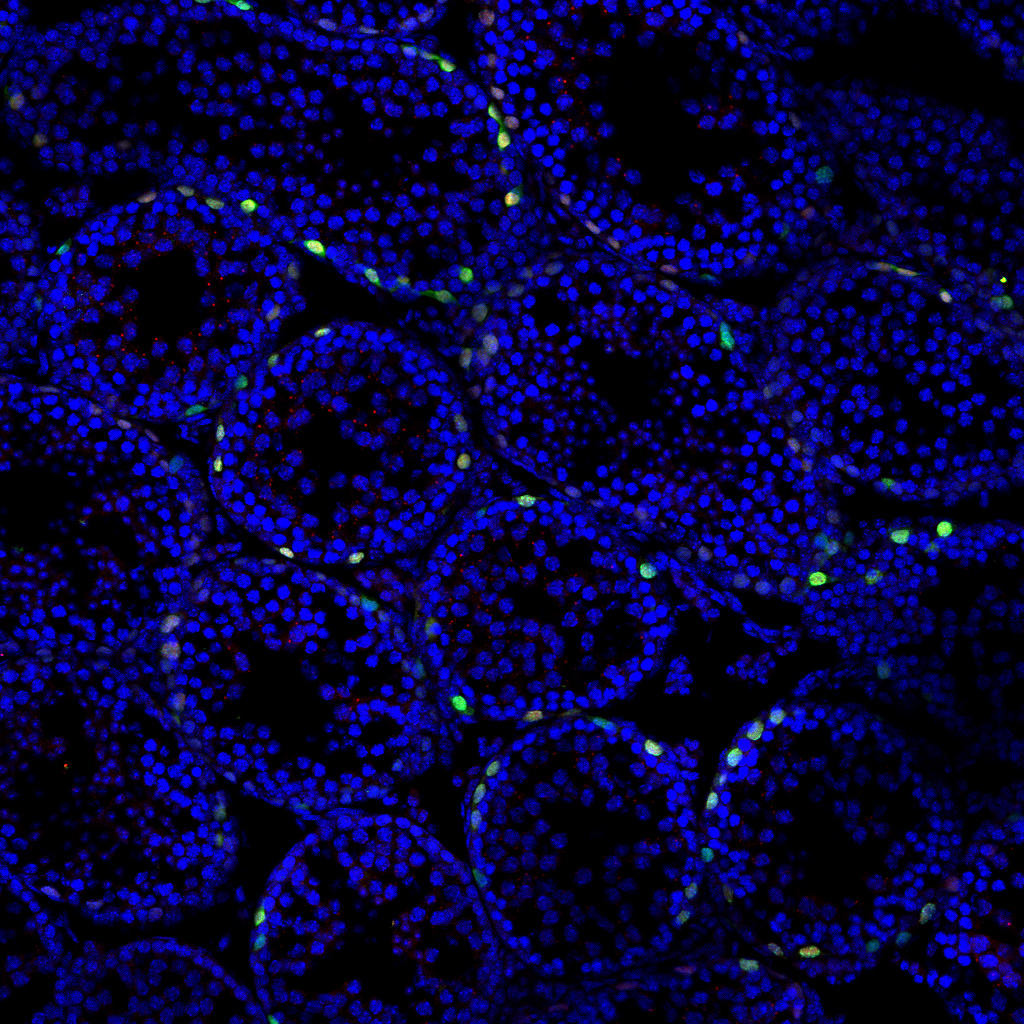

Supplement: Supplementary file 9 — Source data Fig. 6 [file 44319_2025_487_MOESM9_ESM.zip › Figure 6/6F/PD21 Control testis anti-PLZF&SOX3 Hoechst_overlay.tif]

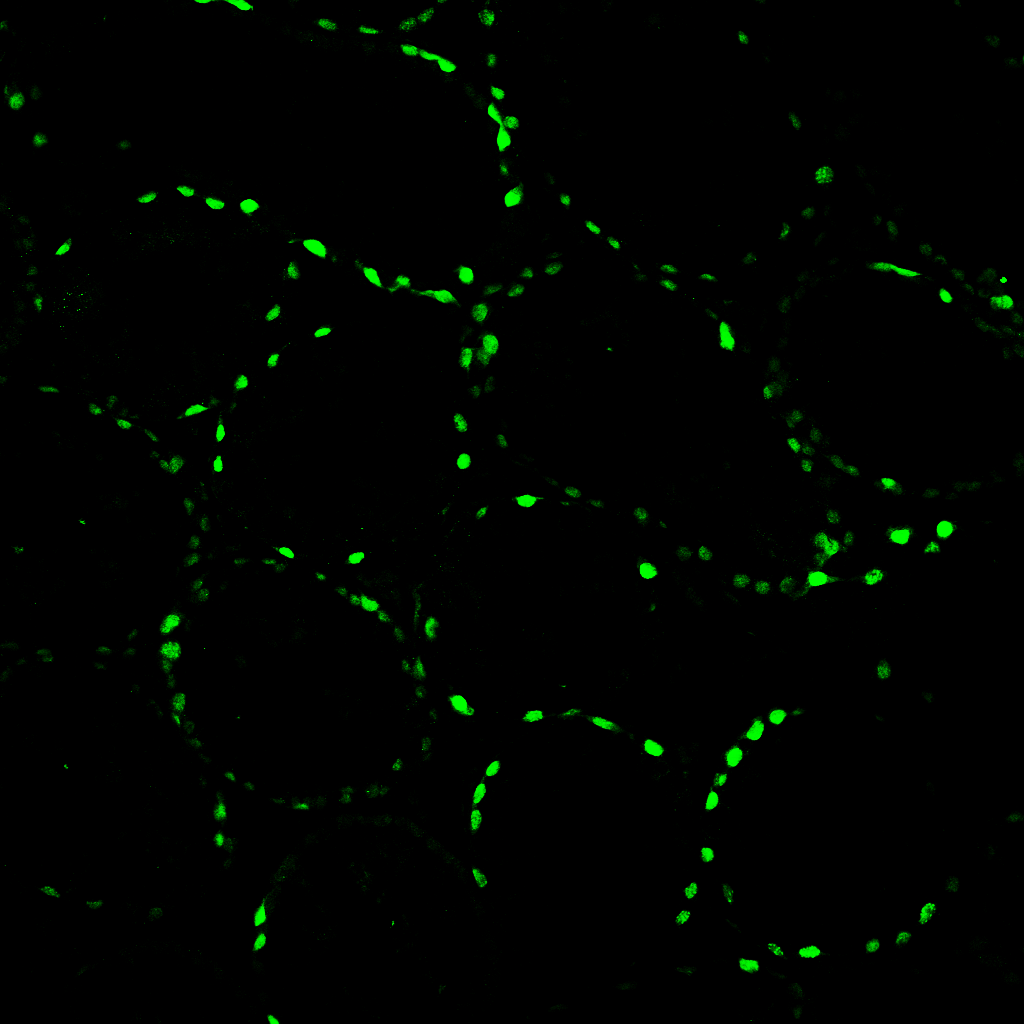

Supplement: Supplementary file 9 — Source data Fig. 6 [file 44319_2025_487_MOESM9_ESM.zip › Figure 6/6F/PD21 Control testis anti-PLZF.tif]

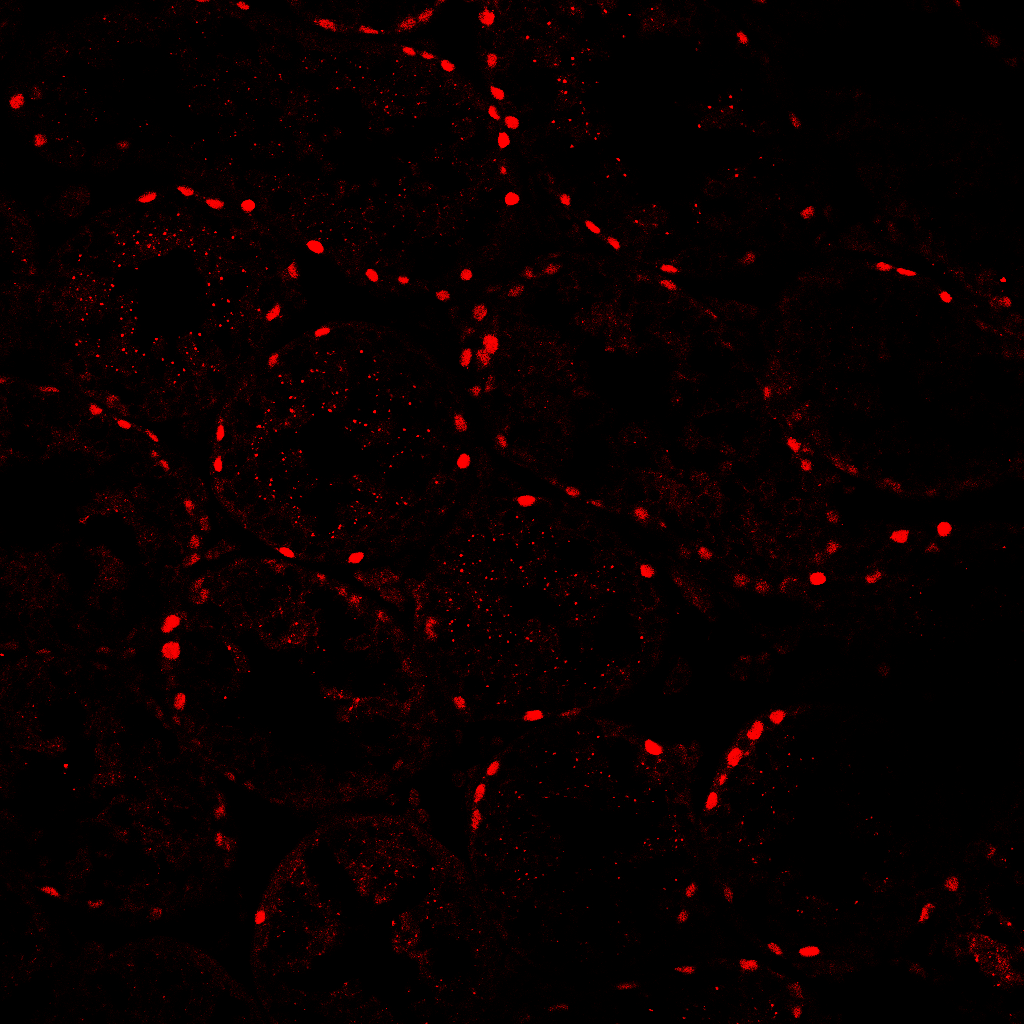

Supplement: Supplementary file 9 — Source data Fig. 6 [file 44319_2025_487_MOESM9_ESM.zip › Figure 6/6F/PD21 Control testis anti-SOX3.tif]

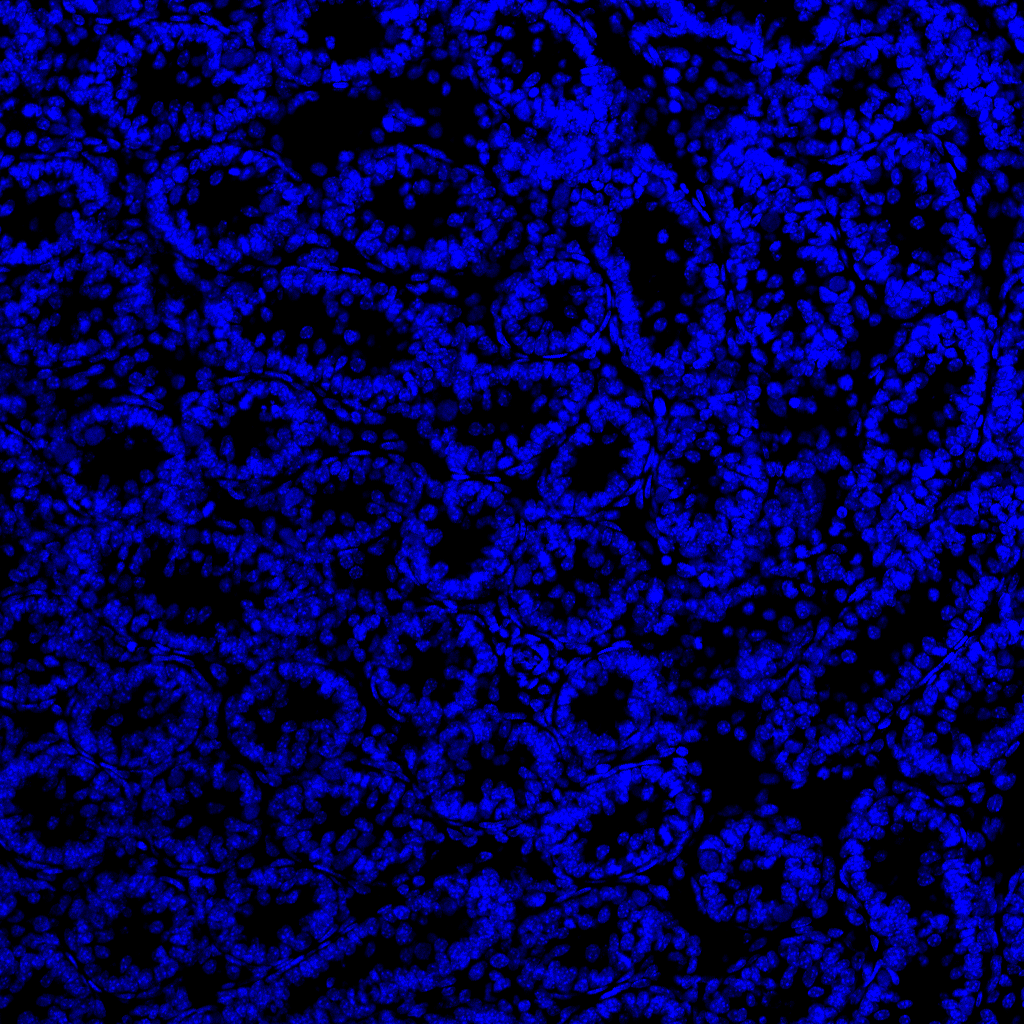

Supplement: Supplementary file 10 — Source data Fig. 7 [file 44319_2025_487_MOESM10_ESM.zip › Figure 7/7A/PD7 testis anti-GFRα1&γH2AX/PD7 Brca1 vKO testis anti-GFRα1&γH2AX Hoechst.tif]

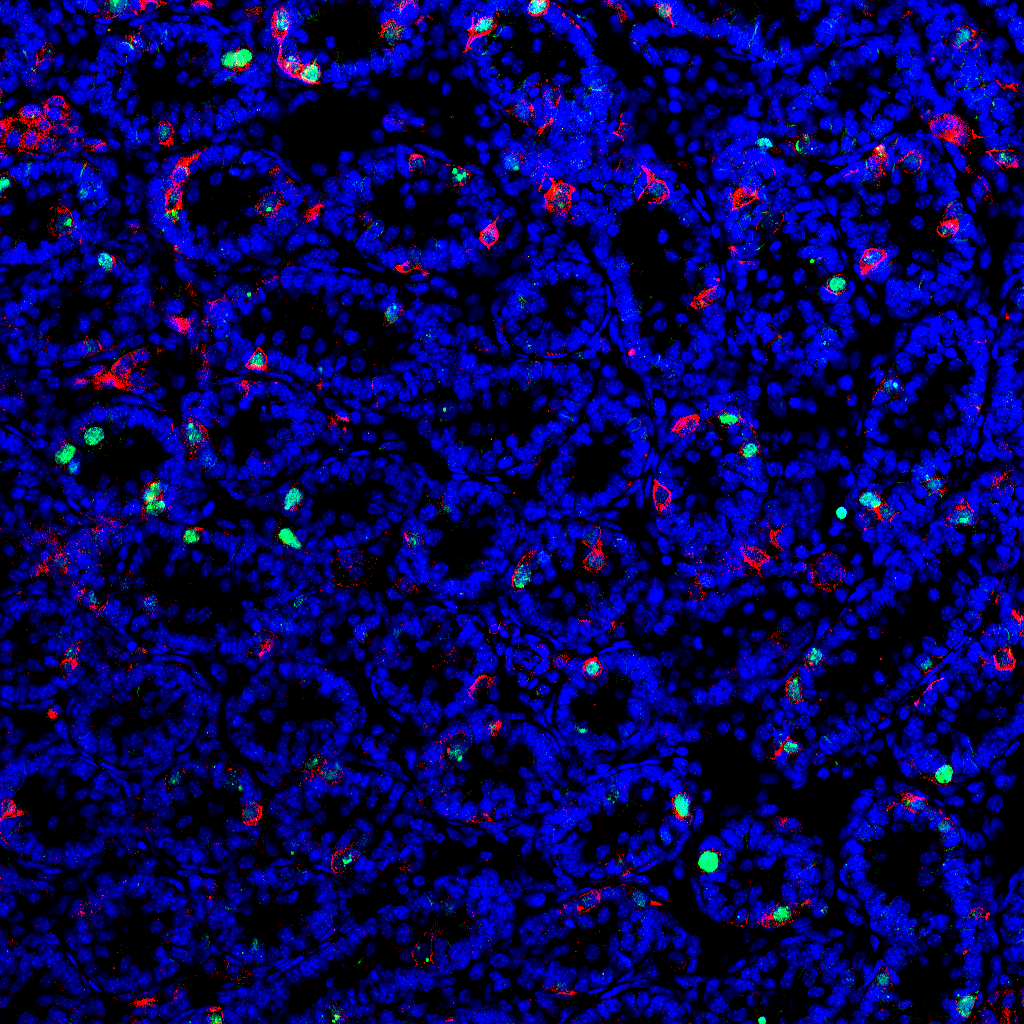

Supplement: Supplementary file 10 — Source data Fig. 7 [file 44319_2025_487_MOESM10_ESM.zip › Figure 7/7A/PD7 testis anti-GFRα1&γH2AX/PD7 Brca1 vKO testis anti-GFRα1&γH2AX Hoechst_overlay.tif]

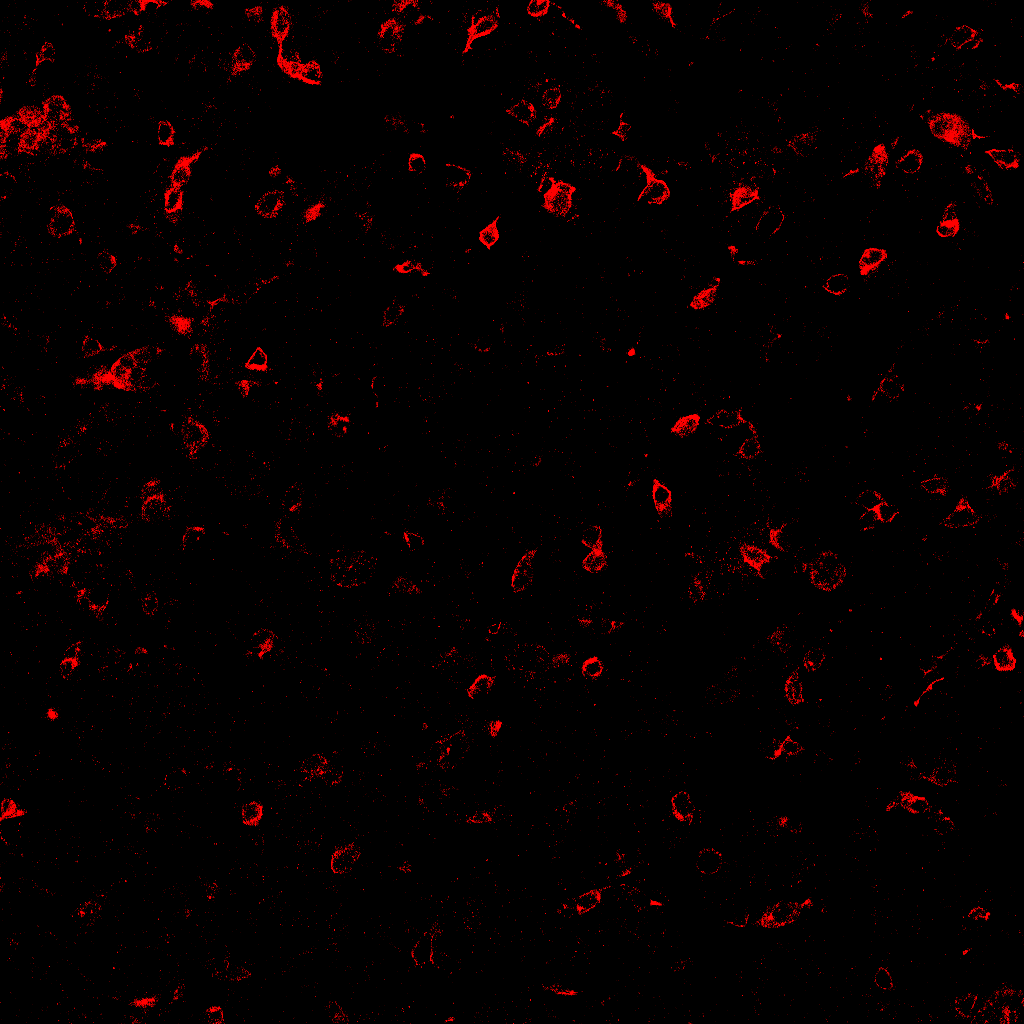

Supplement: Supplementary file 10 — Source data Fig. 7 [file 44319_2025_487_MOESM10_ESM.zip › Figure 7/7A/PD7 testis anti-GFRα1&γH2AX/PD7 Brca1 vKO testis anti-GFRα1.tif]

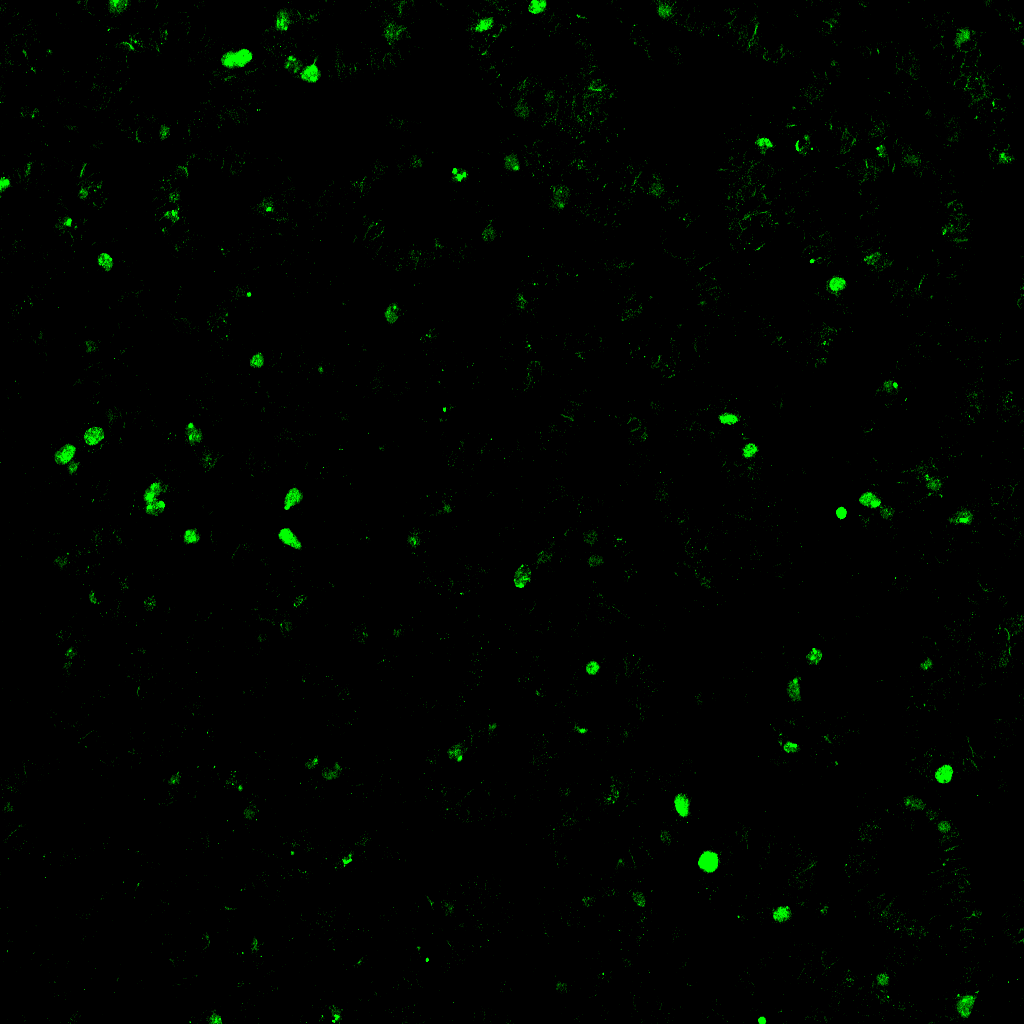

Supplement: Supplementary file 10 — Source data Fig. 7 [file 44319_2025_487_MOESM10_ESM.zip › Figure 7/7A/PD7 testis anti-GFRα1&γH2AX/PD7 Brca1 vKO testis anti-γH2AX.tif]

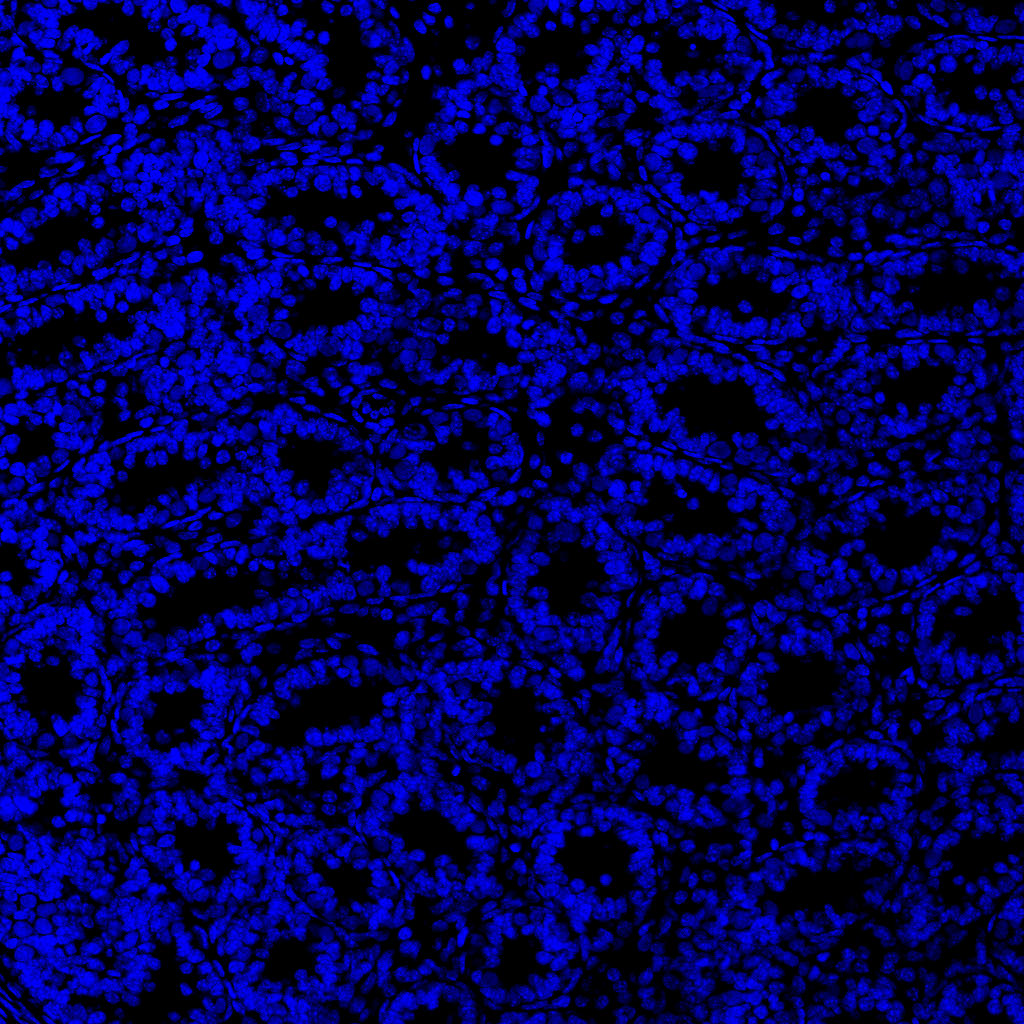

Supplement: Supplementary file 10 — Source data Fig. 7 [file 44319_2025_487_MOESM10_ESM.zip › Figure 7/7A/PD7 testis anti-GFRα1&γH2AX/PD7 Control testis anti-GFRα1&γH2AX Hoechst.tif]

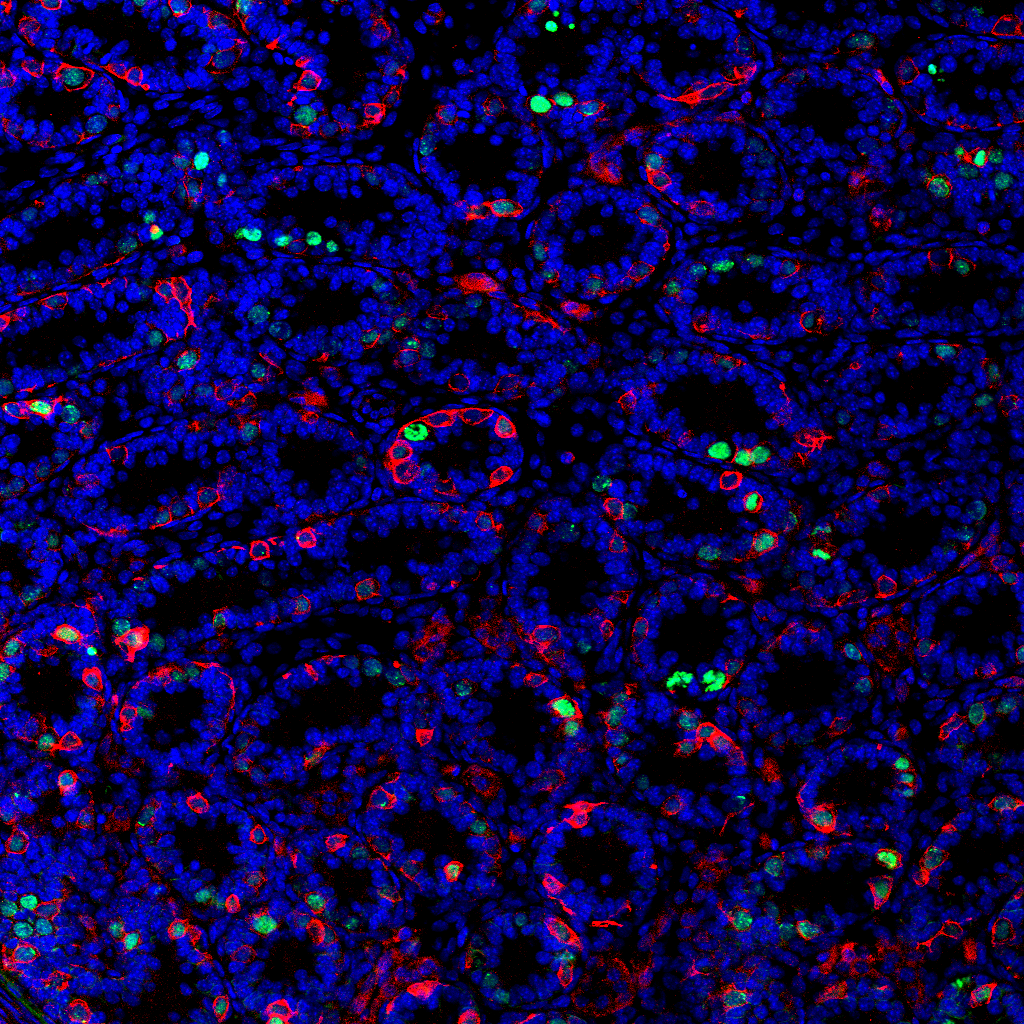

Supplement: Supplementary file 10 — Source data Fig. 7 [file 44319_2025_487_MOESM10_ESM.zip › Figure 7/7A/PD7 testis anti-GFRα1&γH2AX/PD7 Control testis anti-GFRα1&γH2AX Hoechst_overlay.tif]

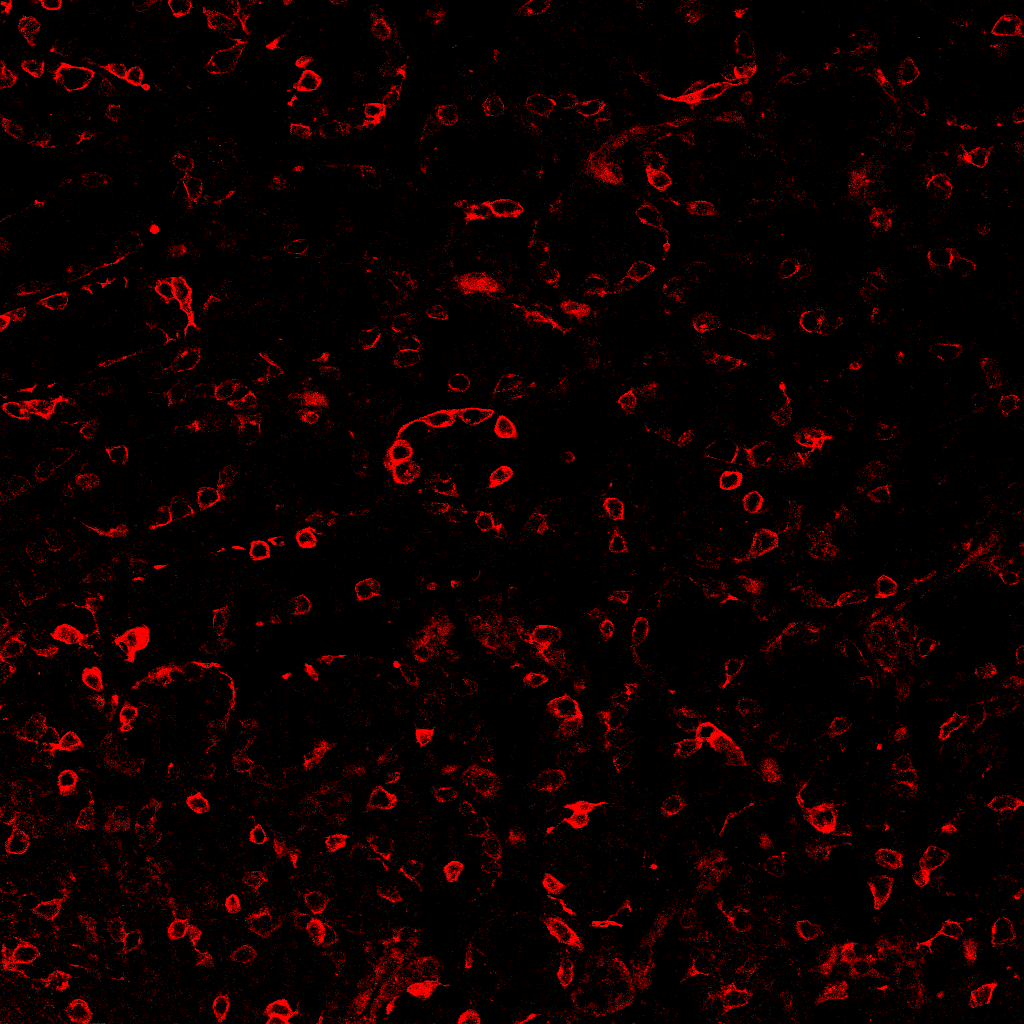

Supplement: Supplementary file 10 — Source data Fig. 7 [file 44319_2025_487_MOESM10_ESM.zip › Figure 7/7A/PD7 testis anti-GFRα1&γH2AX/PD7 Control testis anti-GFRα1.tif]

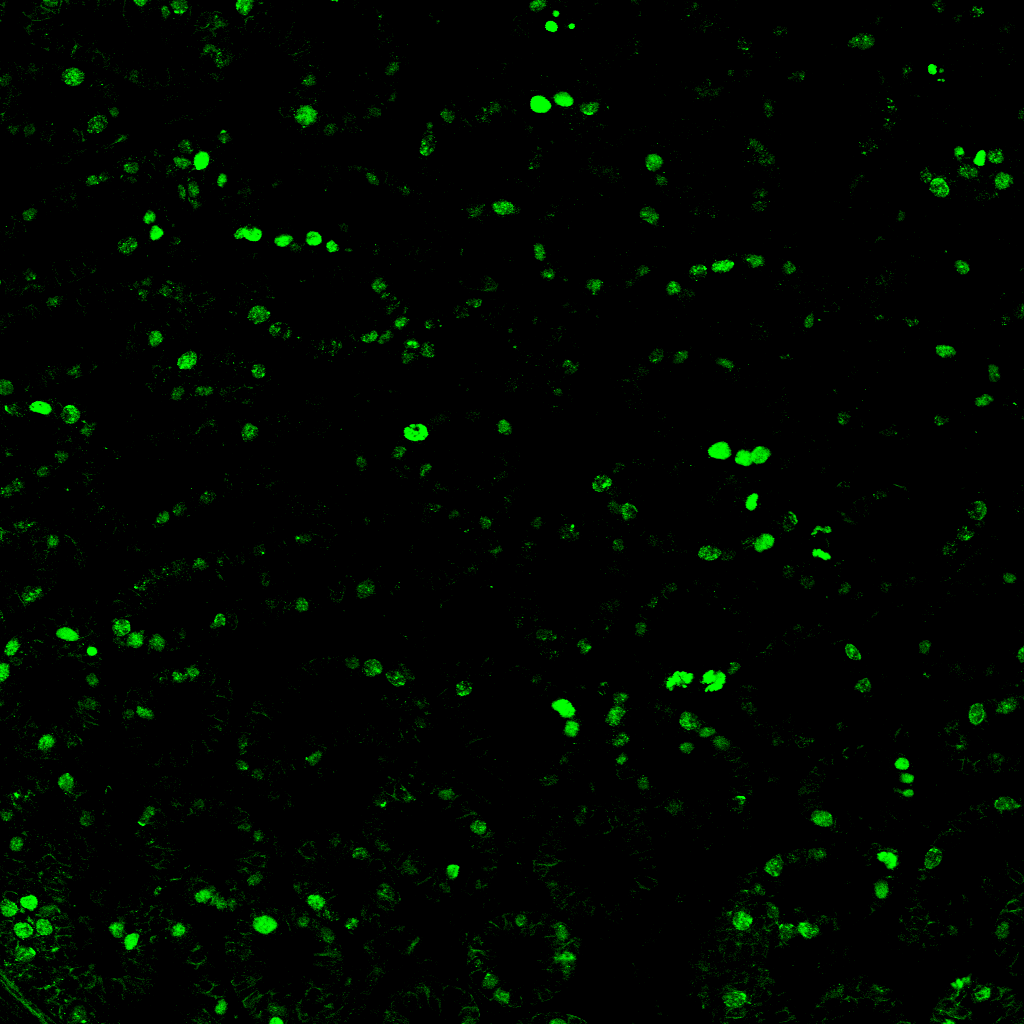

Supplement: Supplementary file 10 — Source data Fig. 7 [file 44319_2025_487_MOESM10_ESM.zip › Figure 7/7A/PD7 testis anti-GFRα1&γH2AX/PD7 Control testis anti-γH2AX.tif]

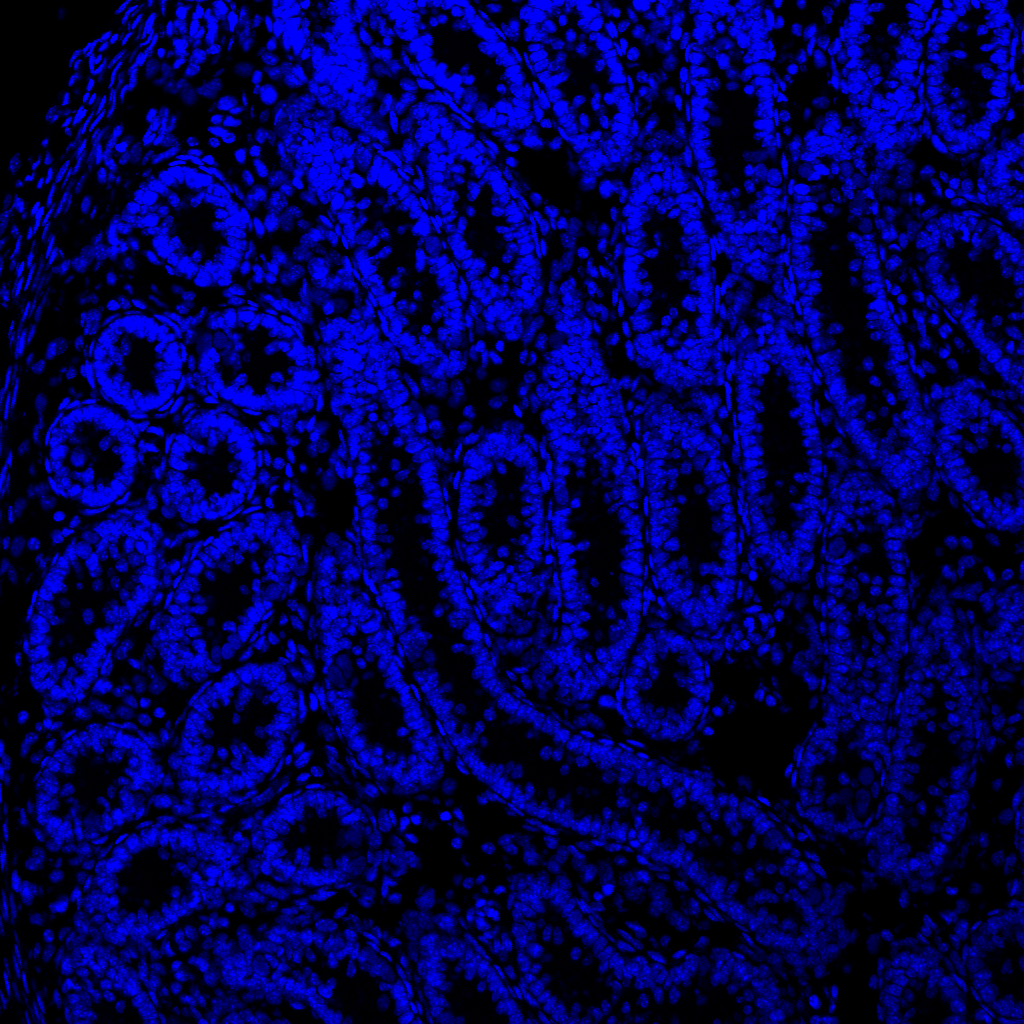

Supplement: Supplementary file 10 — Source data Fig. 7 [file 44319_2025_487_MOESM10_ESM.zip › Figure 7/7A/PD7 testis anti-SOX3&γH2AX/PD7 Brca1 vKO testis anti-SOX3&γH2AX Hoechst.tif]

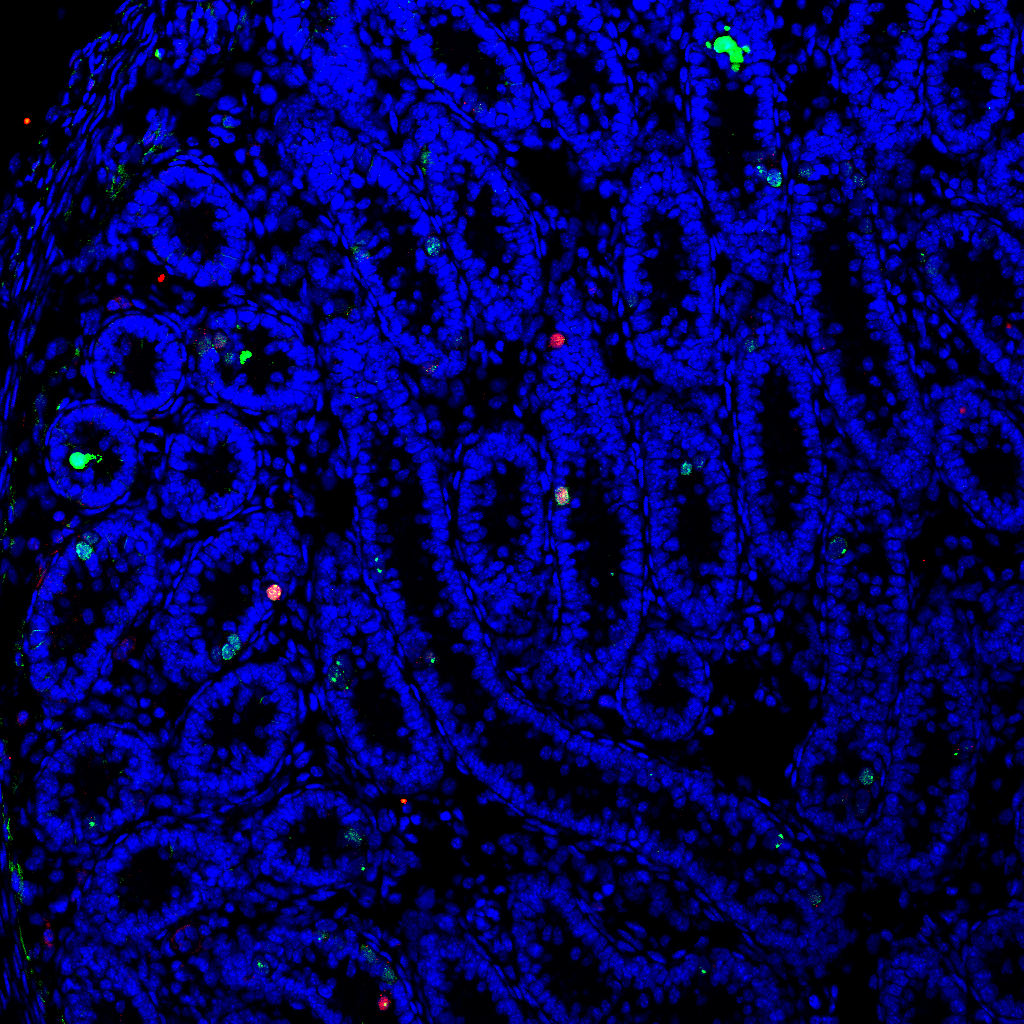

Supplement: Supplementary file 10 — Source data Fig. 7 [file 44319_2025_487_MOESM10_ESM.zip › Figure 7/7A/PD7 testis anti-SOX3&γH2AX/PD7 Brca1 vKO testis anti-SOX3&γH2AX Hoechst_overlay.tif]

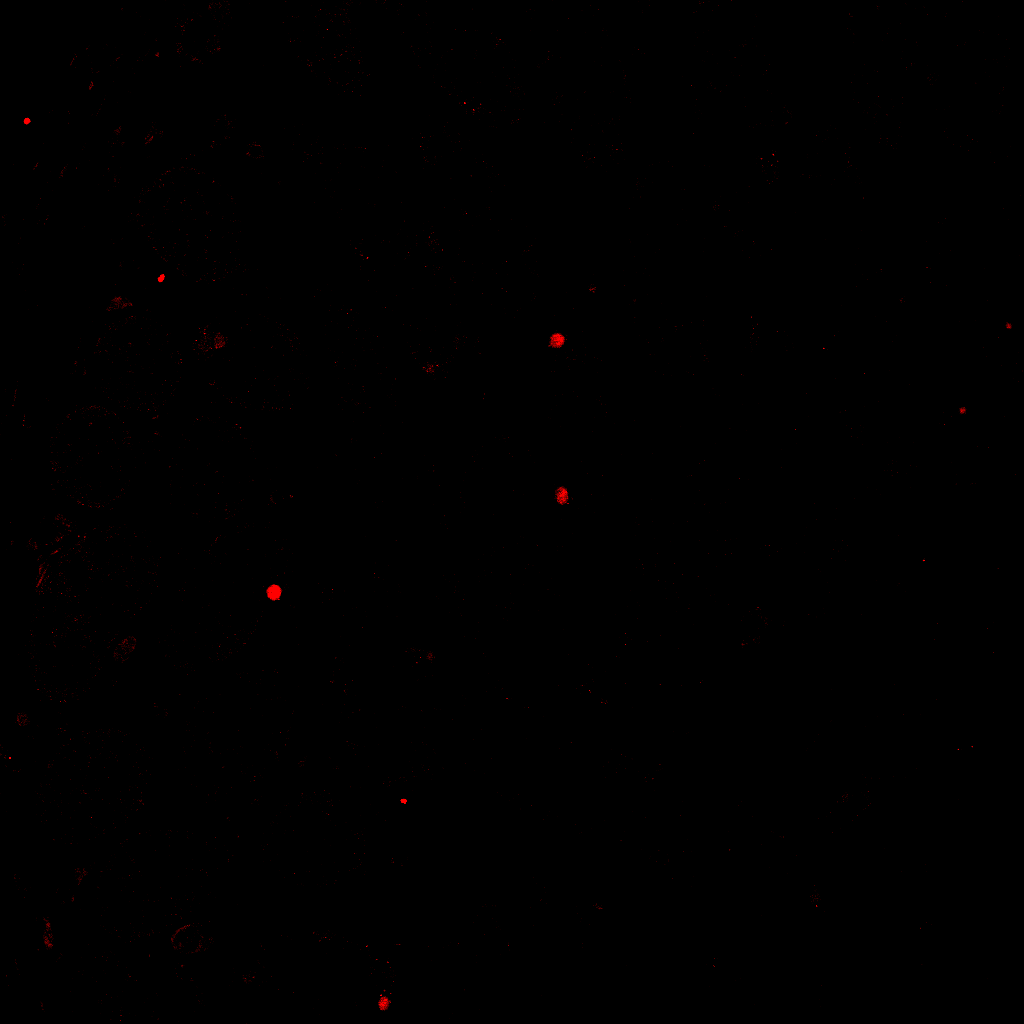

Supplement: Supplementary file 10 — Source data Fig. 7 [file 44319_2025_487_MOESM10_ESM.zip › Figure 7/7A/PD7 testis anti-SOX3&γH2AX/PD7 Brca1 vKO testis anti-SOX3.tif]

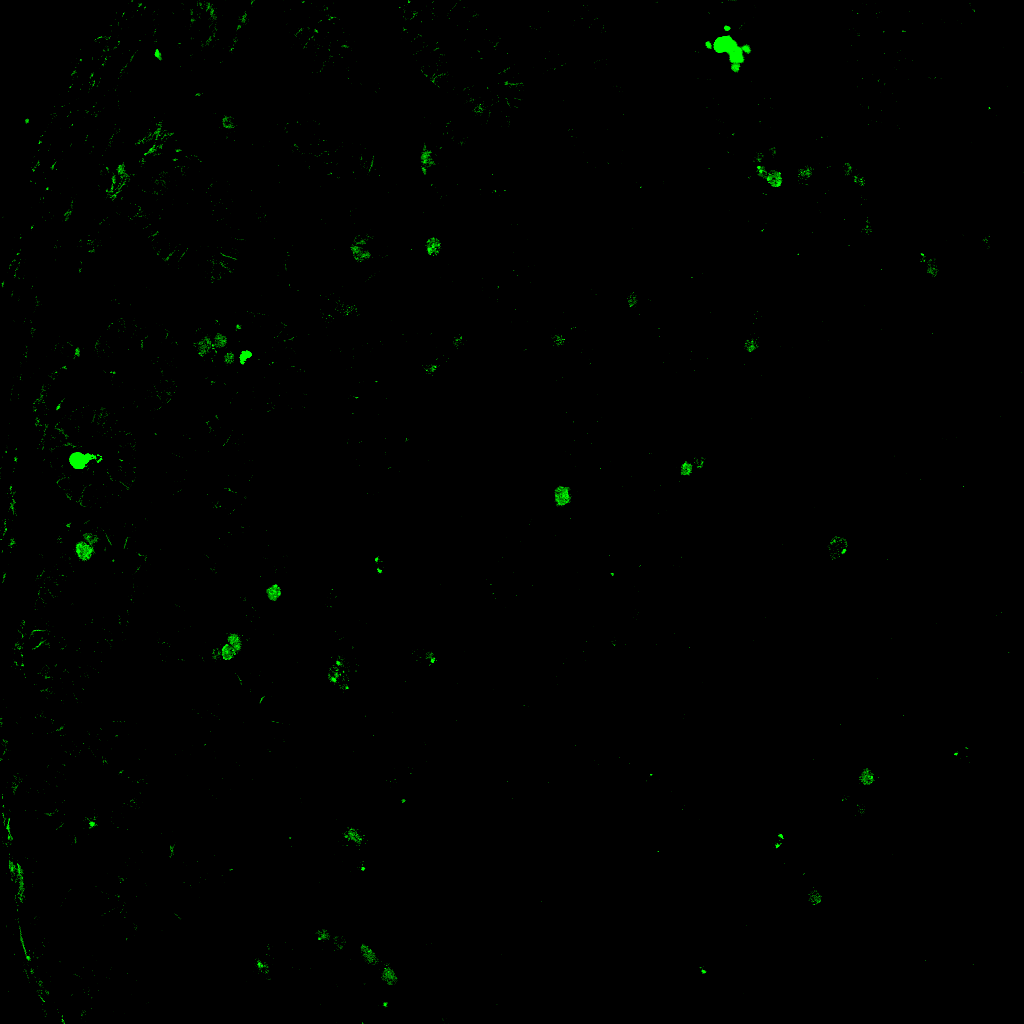

Supplement: Supplementary file 10 — Source data Fig. 7 [file 44319_2025_487_MOESM10_ESM.zip › Figure 7/7A/PD7 testis anti-SOX3&γH2AX/PD7 Brca1 vKO testis anti-γH2AX.tif]

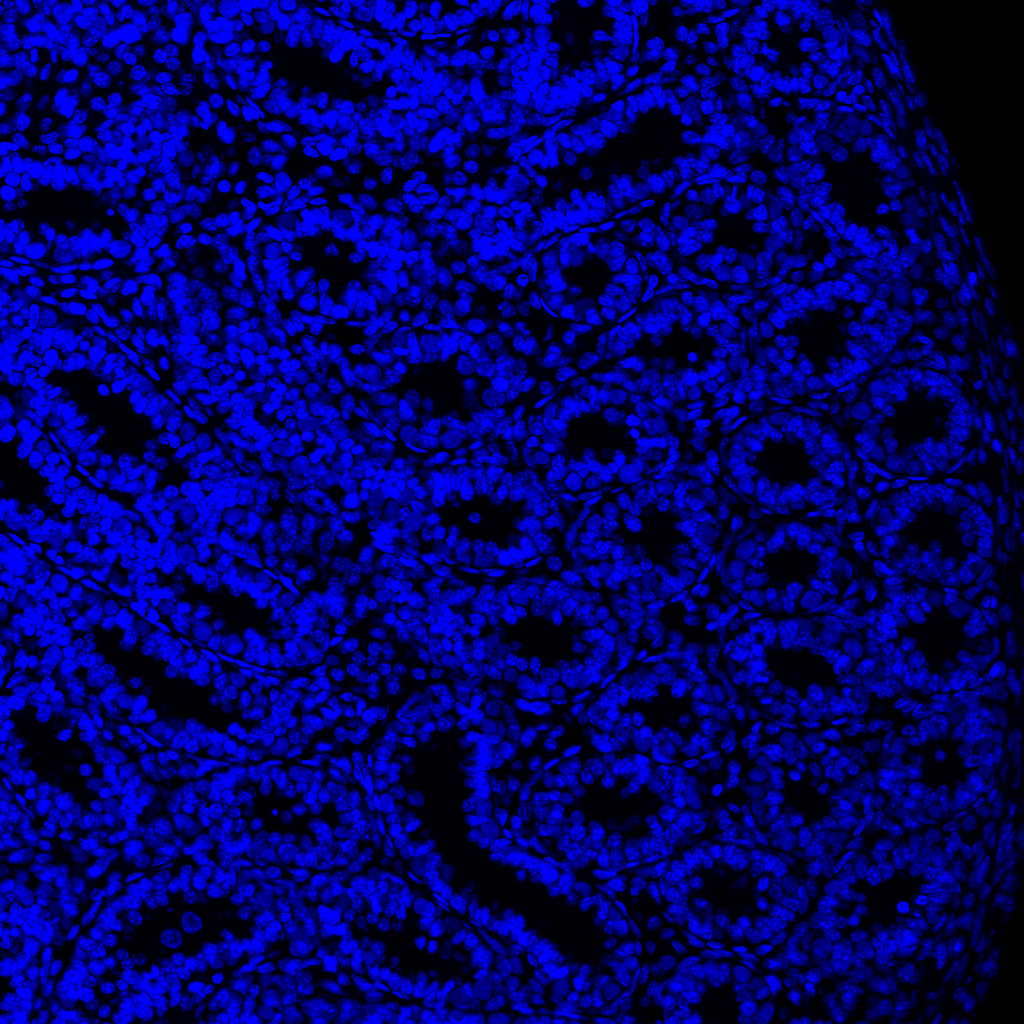

Supplement: Supplementary file 10 — Source data Fig. 7 [file 44319_2025_487_MOESM10_ESM.zip › Figure 7/7A/PD7 testis anti-SOX3&γH2AX/PD7 Control testis anti-SOX3&γH2AX Hoechst.tif]

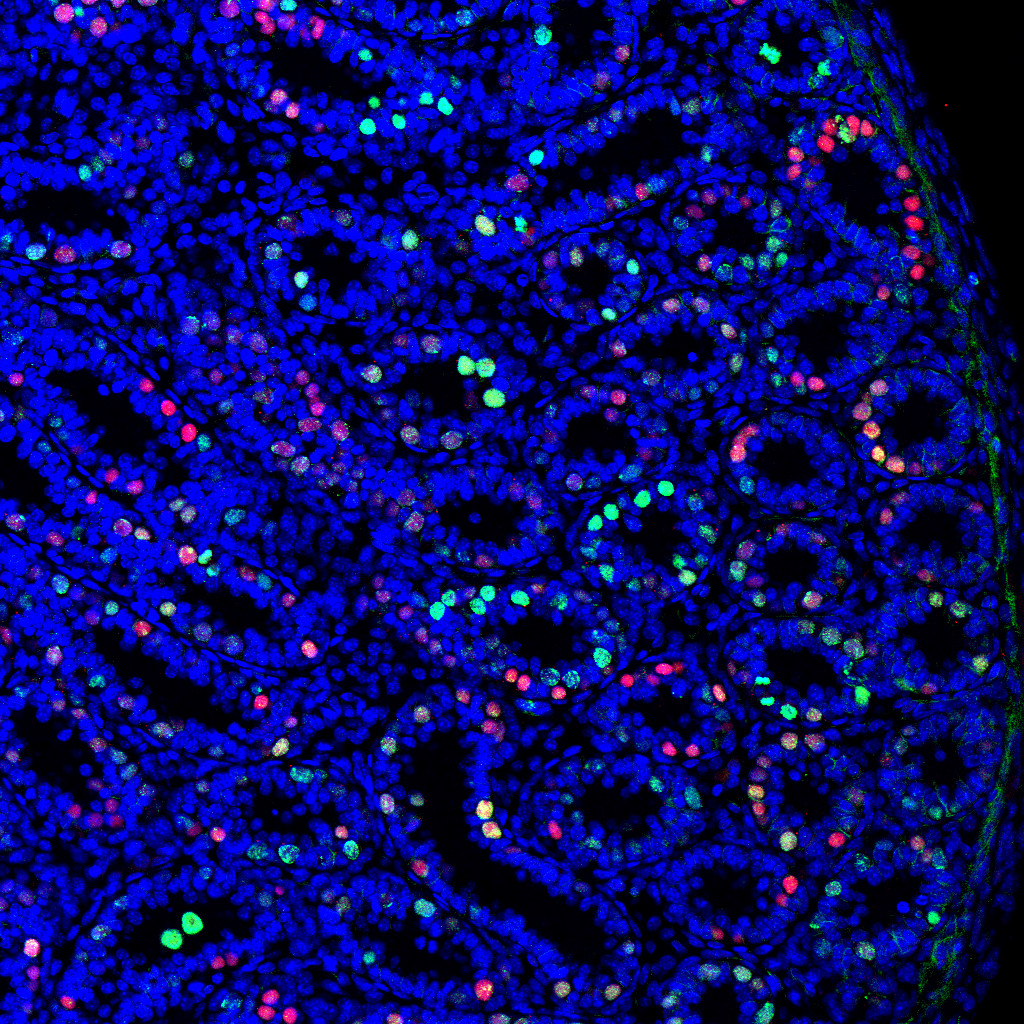

Supplement: Supplementary file 10 — Source data Fig. 7 [file 44319_2025_487_MOESM10_ESM.zip › Figure 7/7A/PD7 testis anti-SOX3&γH2AX/PD7 Control testis anti-SOX3&γH2AX Hoechst_overlay.tif]

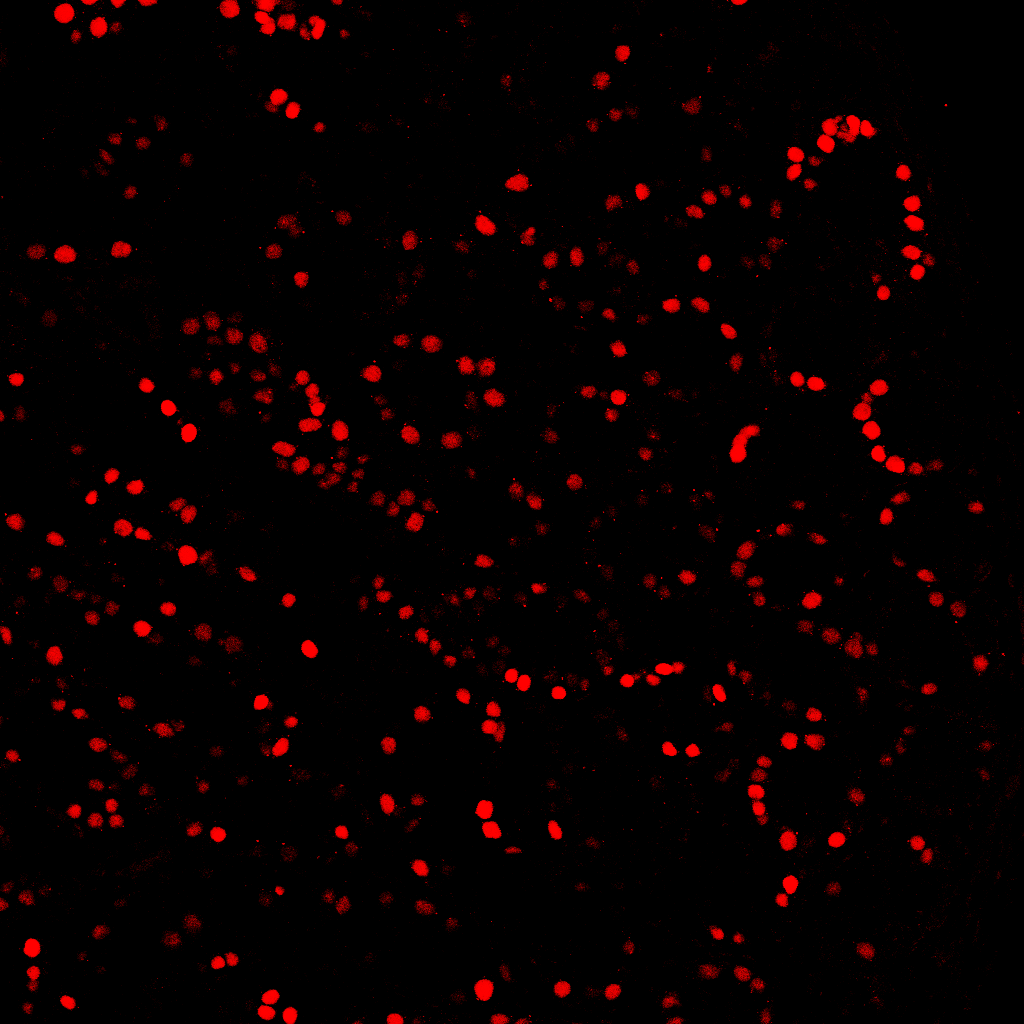

Supplement: Supplementary file 10 — Source data Fig. 7 [file 44319_2025_487_MOESM10_ESM.zip › Figure 7/7A/PD7 testis anti-SOX3&γH2AX/PD7 Control testis anti-SOX3.tif]

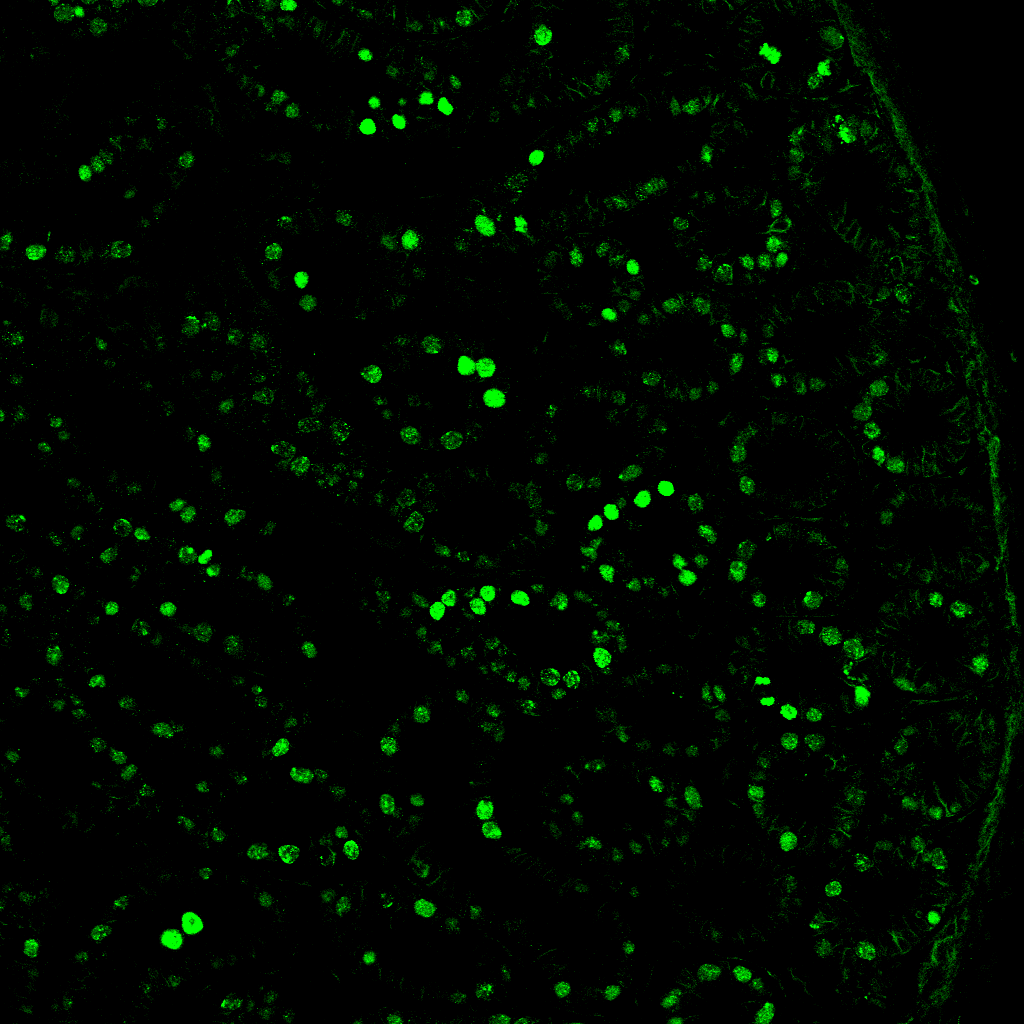

Supplement: Supplementary file 10 — Source data Fig. 7 [file 44319_2025_487_MOESM10_ESM.zip › Figure 7/7A/PD7 testis anti-SOX3&γH2AX/PD7 Control testis anti-γH2AX.tif]

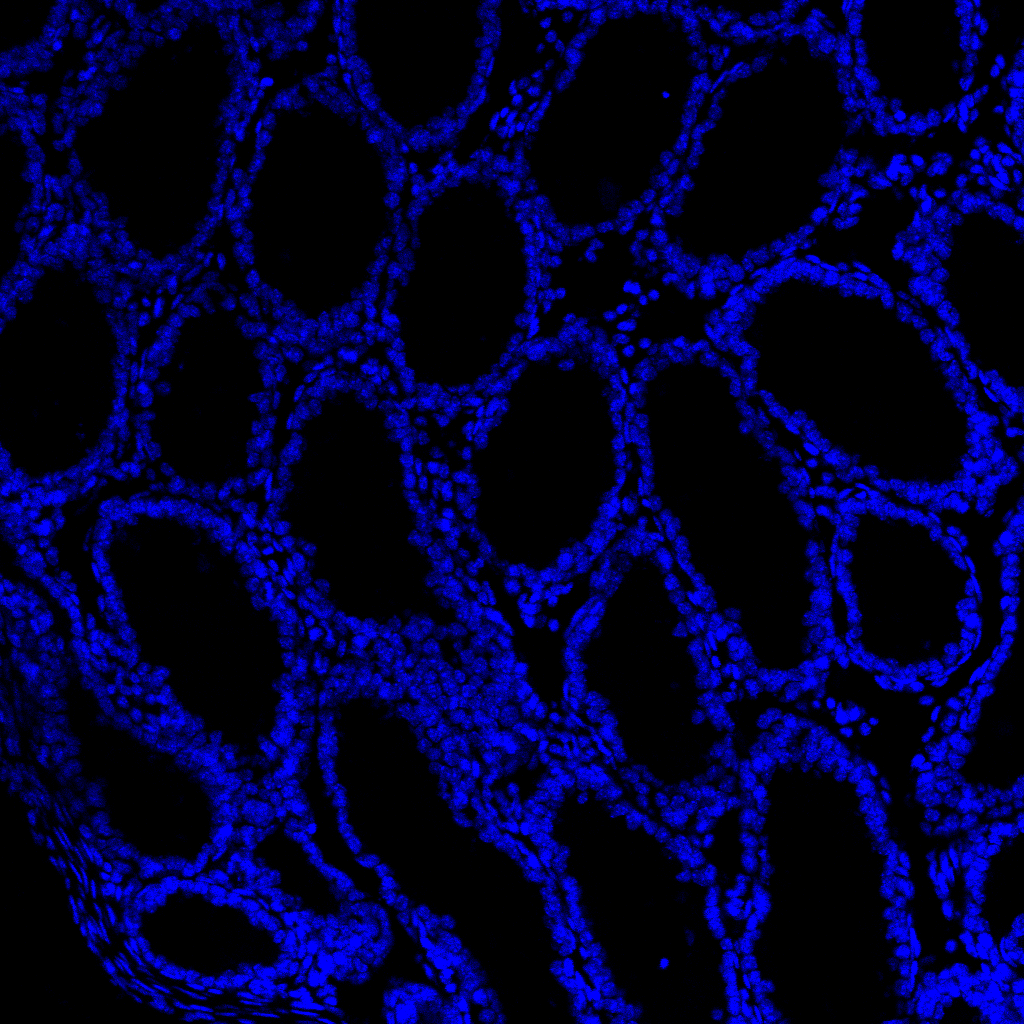

Supplement: Supplementary file 10 — Source data Fig. 7 [file 44319_2025_487_MOESM10_ESM.zip › Figure 7/7D/PD14 testis anti-GFRa1&γH2AX/PD14 Brca1 vKO testis anti-GFRa1&γH2AX Hoechst.tif]

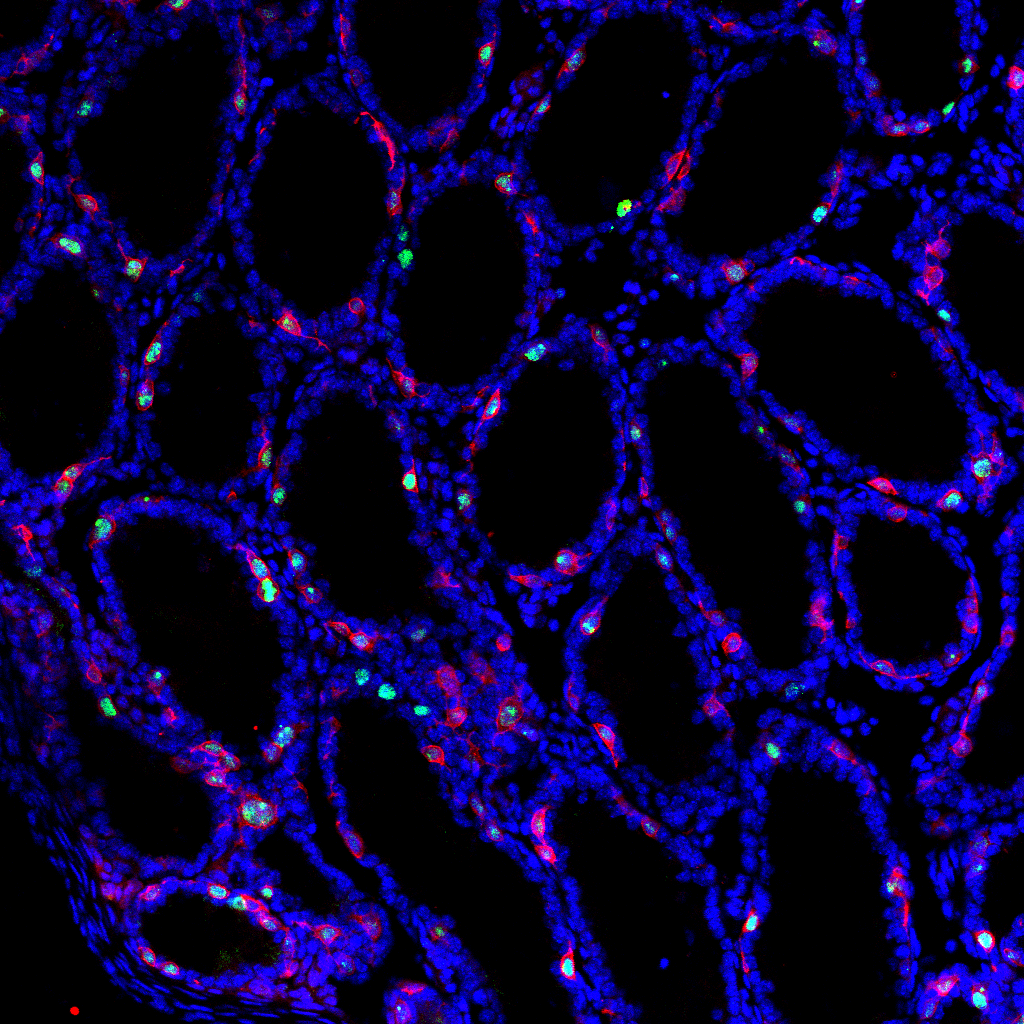

Supplement: Supplementary file 10 — Source data Fig. 7 [file 44319_2025_487_MOESM10_ESM.zip › Figure 7/7D/PD14 testis anti-GFRa1&γH2AX/PD14 Brca1 vKO testis anti-GFRa1&γH2AX Hoechst_overlay.tif]

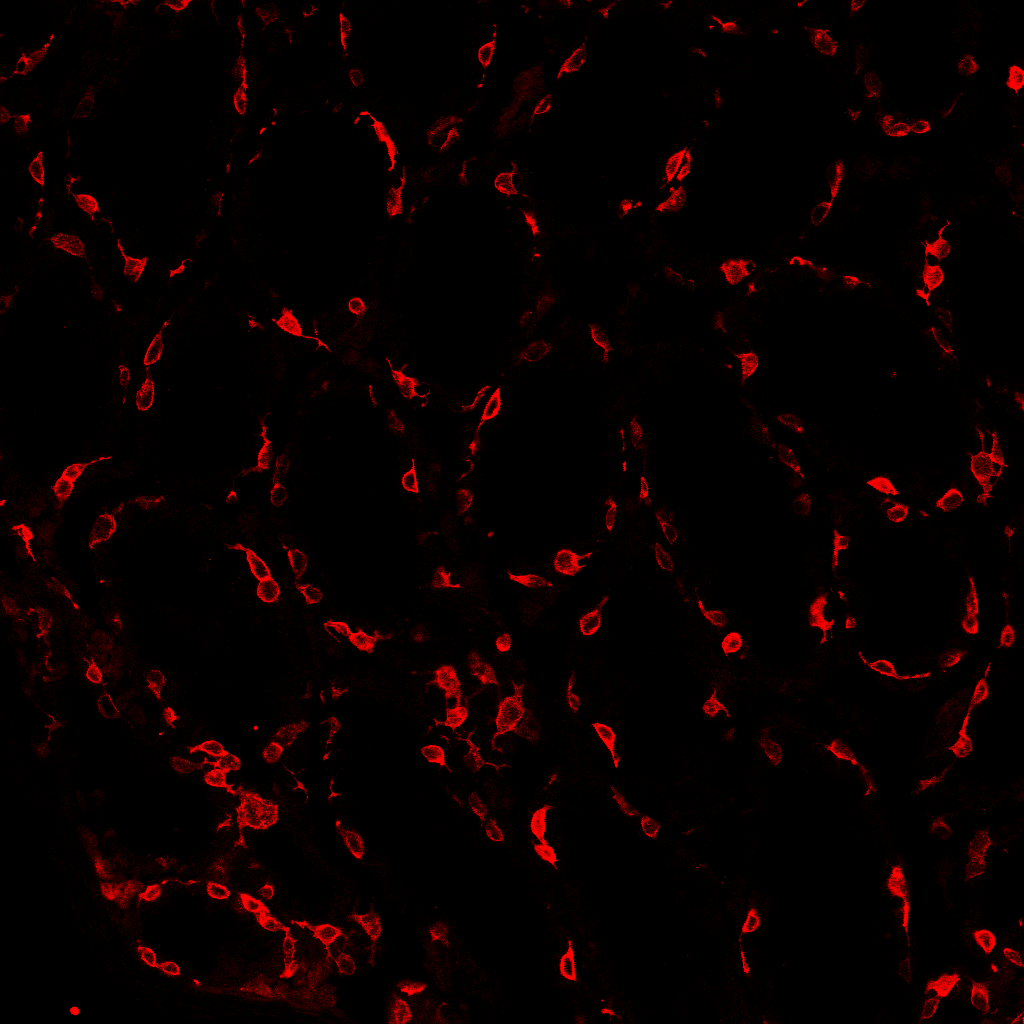

Supplement: Supplementary file 10 — Source data Fig. 7 [file 44319_2025_487_MOESM10_ESM.zip › Figure 7/7D/PD14 testis anti-GFRa1&γH2AX/PD14 Brca1 vKO testis anti-GFRa1.tif]

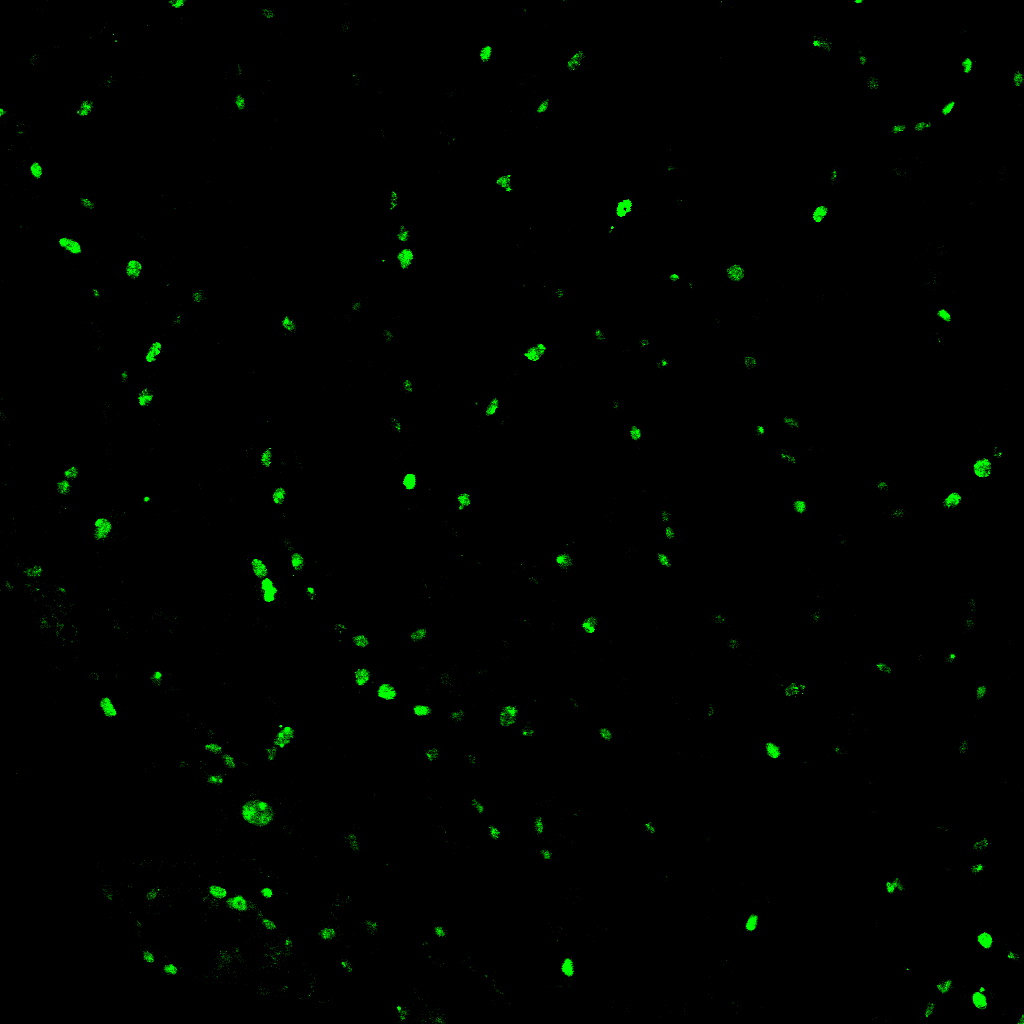

Supplement: Supplementary file 10 — Source data Fig. 7 [file 44319_2025_487_MOESM10_ESM.zip › Figure 7/7D/PD14 testis anti-GFRa1&γH2AX/PD14 Brca1 vKO testis anti-γH2AX.tif]

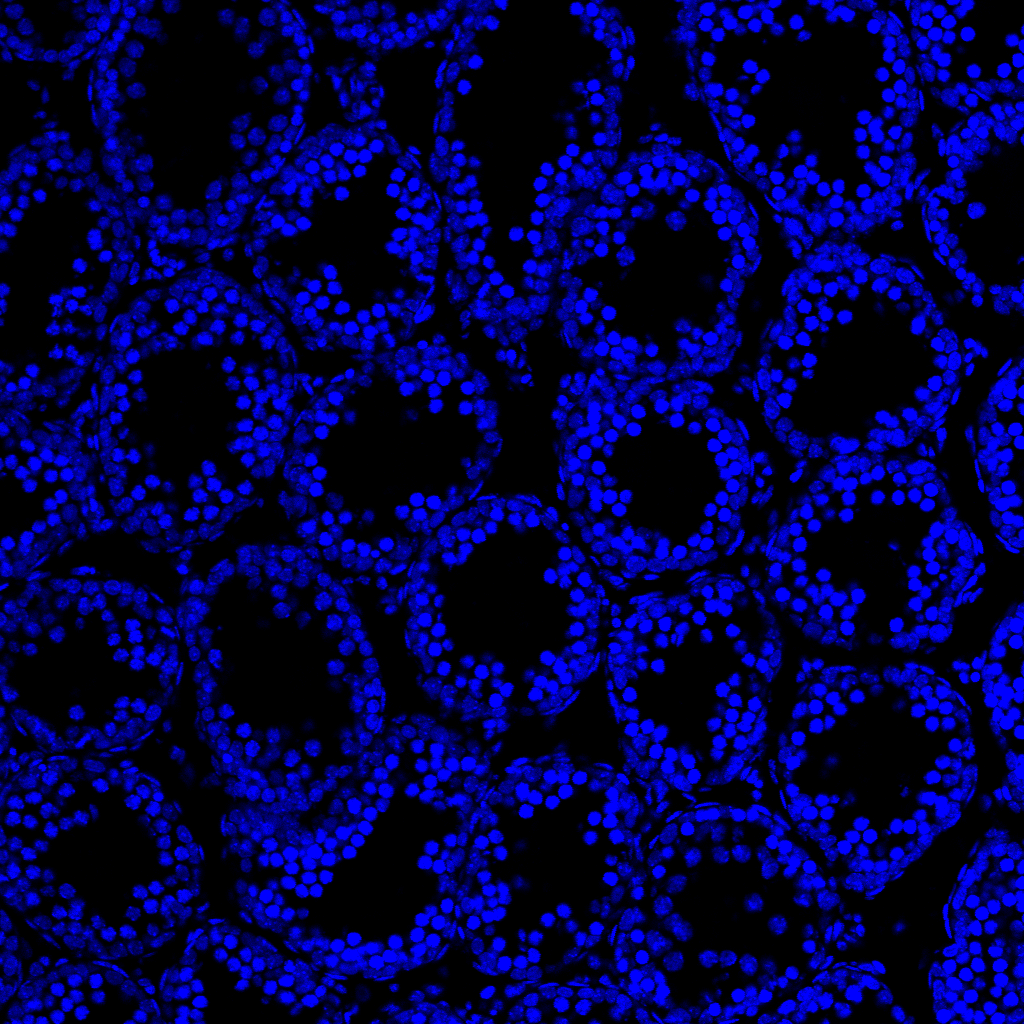

Supplement: Supplementary file 10 — Source data Fig. 7 [file 44319_2025_487_MOESM10_ESM.zip › Figure 7/7D/PD14 testis anti-GFRa1&γH2AX/PD14 Control testis anti-GFRa1&γH2AX Hoechst.tif]

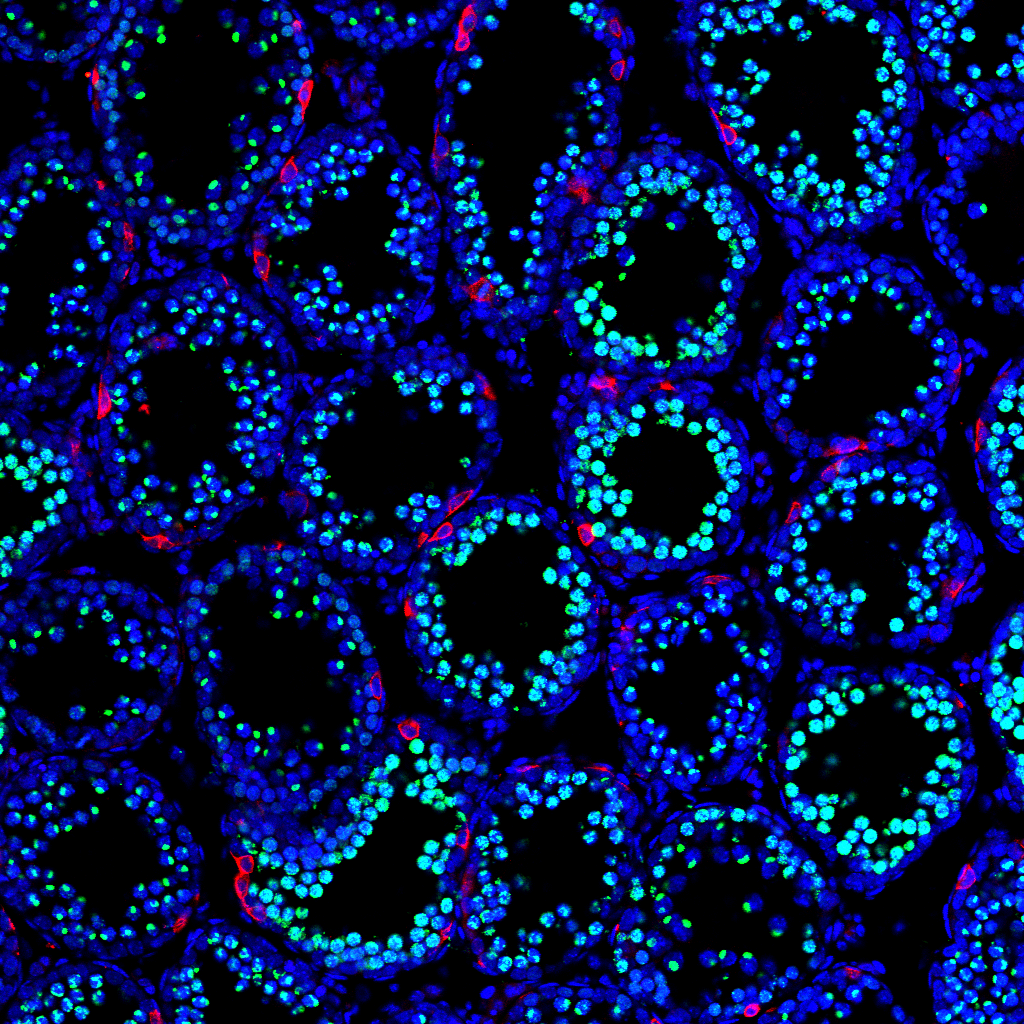

Supplement: Supplementary file 10 — Source data Fig. 7 [file 44319_2025_487_MOESM10_ESM.zip › Figure 7/7D/PD14 testis anti-GFRa1&γH2AX/PD14 Control testis anti-GFRa1&γH2AX Hoechst_overlay.tif]

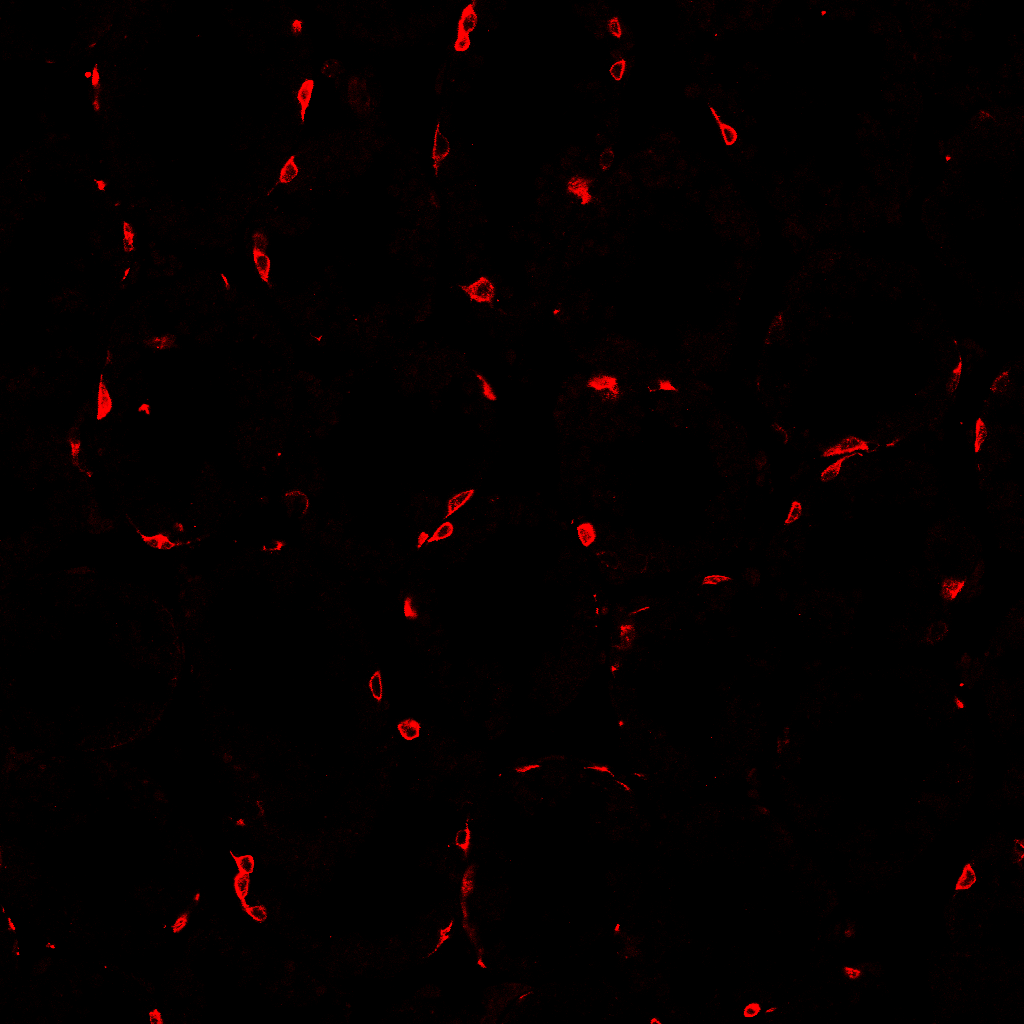

Supplement: Supplementary file 10 — Source data Fig. 7 [file 44319_2025_487_MOESM10_ESM.zip › Figure 7/7D/PD14 testis anti-GFRa1&γH2AX/PD14 Control testis anti-GFRa1.tif]

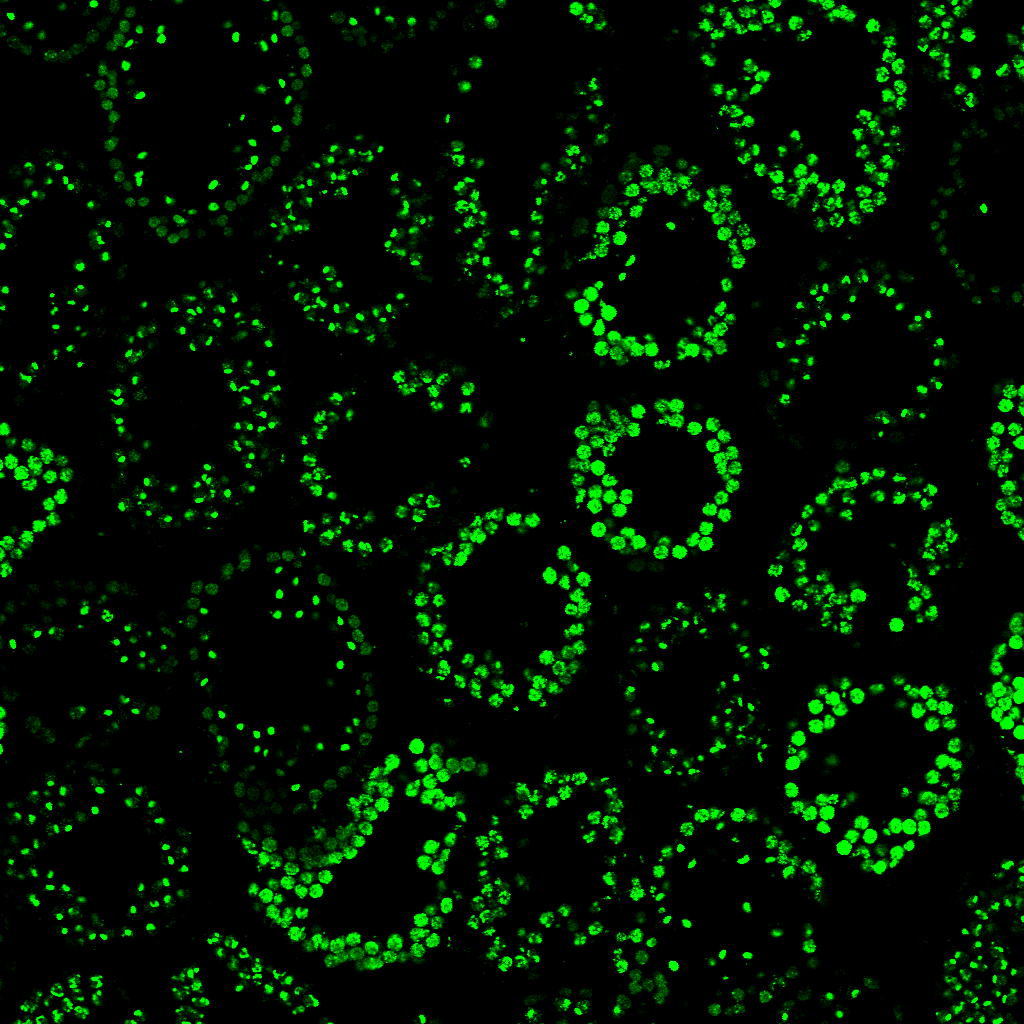

Supplement: Supplementary file 10 — Source data Fig. 7 [file 44319_2025_487_MOESM10_ESM.zip › Figure 7/7D/PD14 testis anti-GFRa1&γH2AX/PD14 Control testis anti-γH2AX.tif]

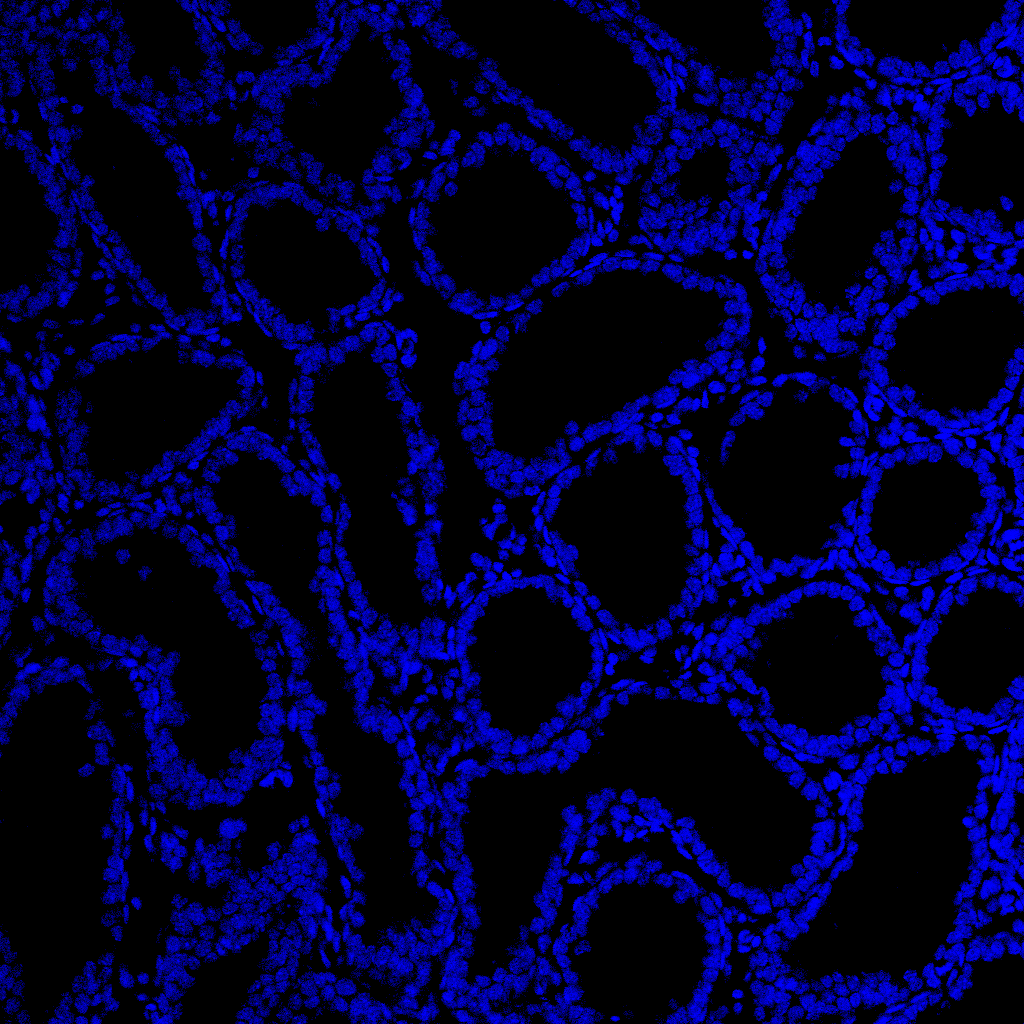

Supplement: Supplementary file 10 — Source data Fig. 7 [file 44319_2025_487_MOESM10_ESM.zip › Figure 7/7D/PD14 testis anti-SOX3&γH2AX/PD14 Brca1 vKO testis anti-SOX3&γH2AX Hoechst.tif]

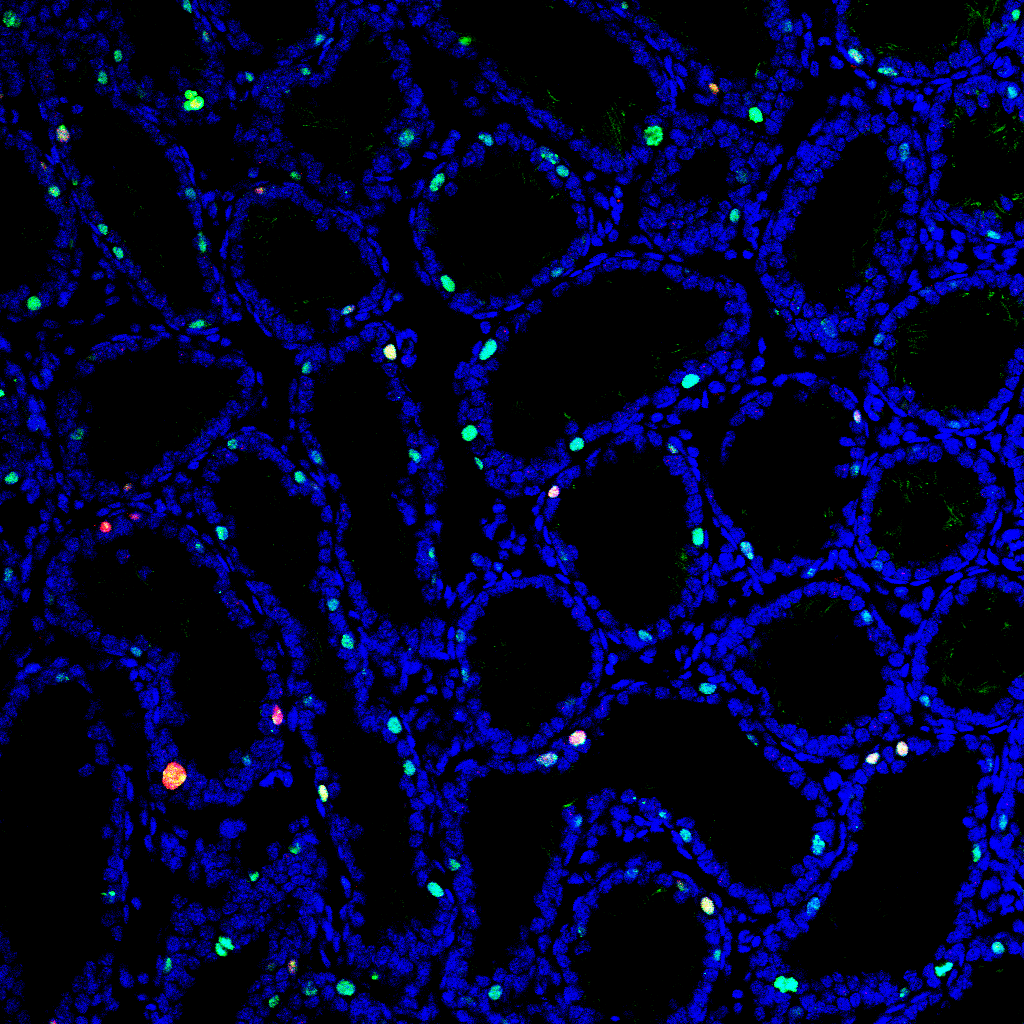

Supplement: Supplementary file 10 — Source data Fig. 7 [file 44319_2025_487_MOESM10_ESM.zip › Figure 7/7D/PD14 testis anti-SOX3&γH2AX/PD14 Brca1 vKO testis anti-SOX3&γH2AX Hoechst_overlay.tif]

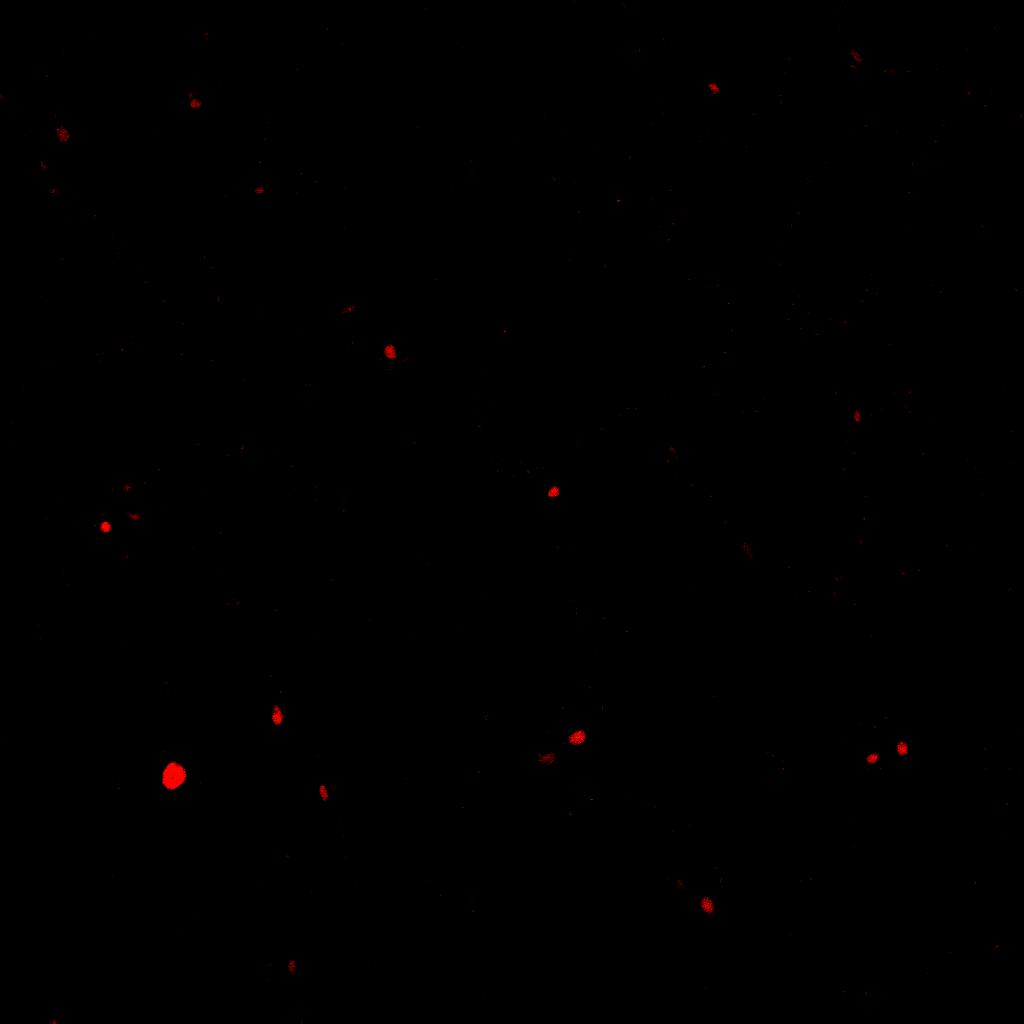

Supplement: Supplementary file 10 — Source data Fig. 7 [file 44319_2025_487_MOESM10_ESM.zip › Figure 7/7D/PD14 testis anti-SOX3&γH2AX/PD14 Brca1 vKO testis anti-SOX3.tif]

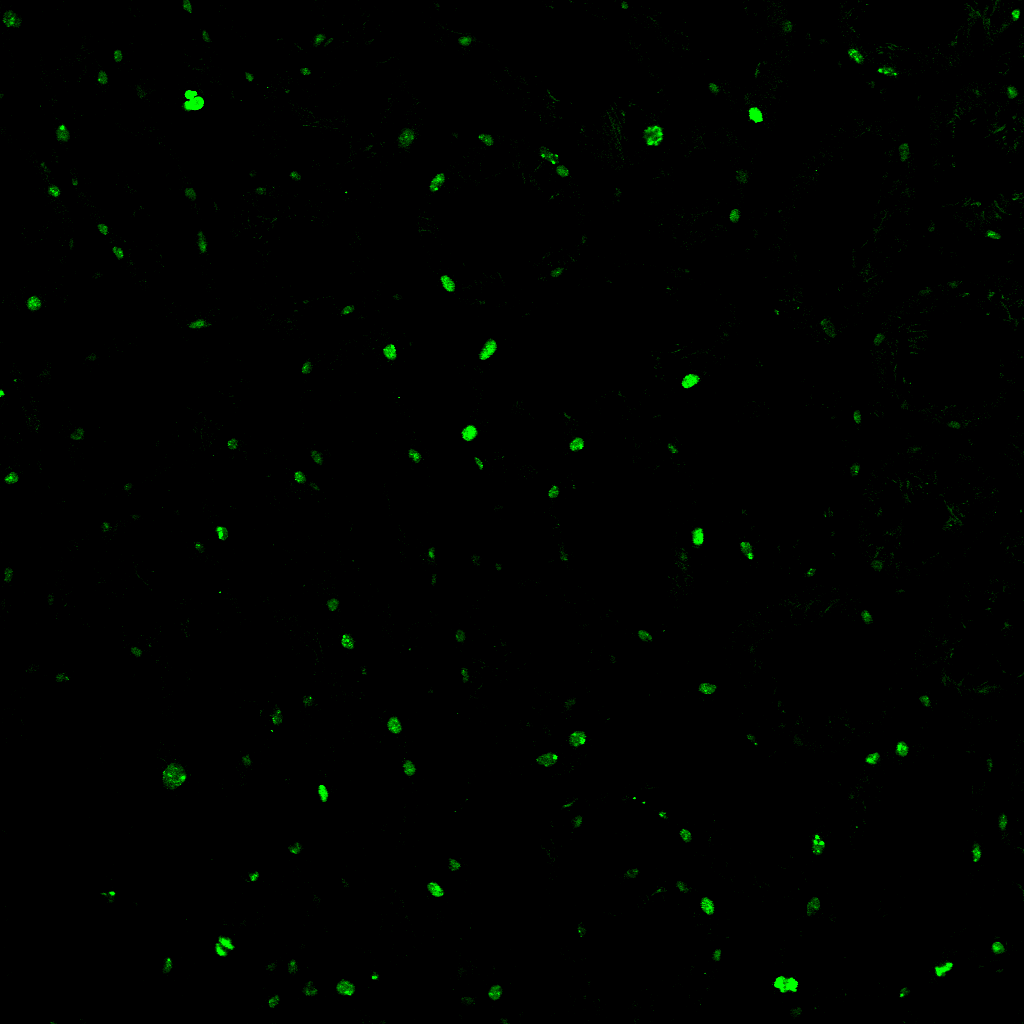

Supplement: Supplementary file 10 — Source data Fig. 7 [file 44319_2025_487_MOESM10_ESM.zip › Figure 7/7D/PD14 testis anti-SOX3&γH2AX/PD14 Brca1 vKO testis anti-γH2AX.tif]

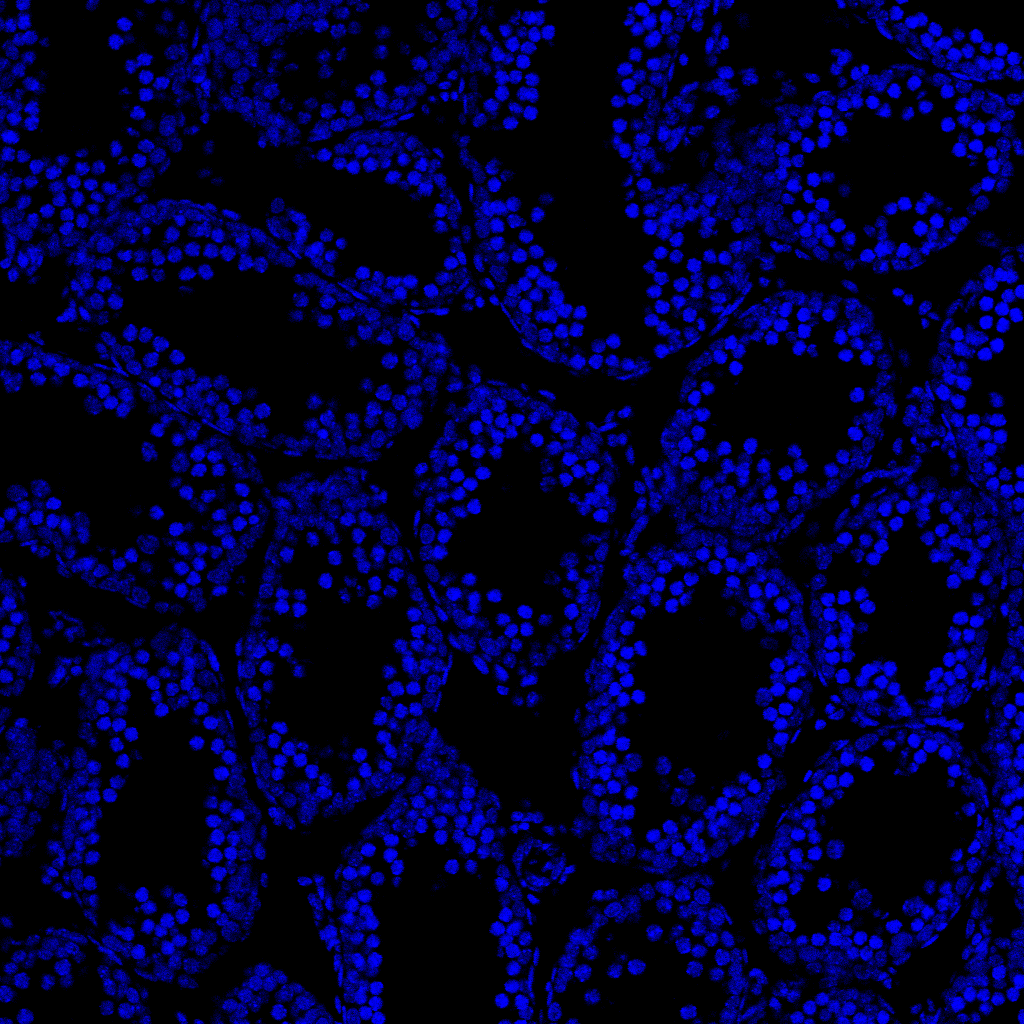

Supplement: Supplementary file 10 — Source data Fig. 7 [file 44319_2025_487_MOESM10_ESM.zip › Figure 7/7D/PD14 testis anti-SOX3&γH2AX/PD14 Control testis anti-SOX3&γH2AX Hoechst.tif]

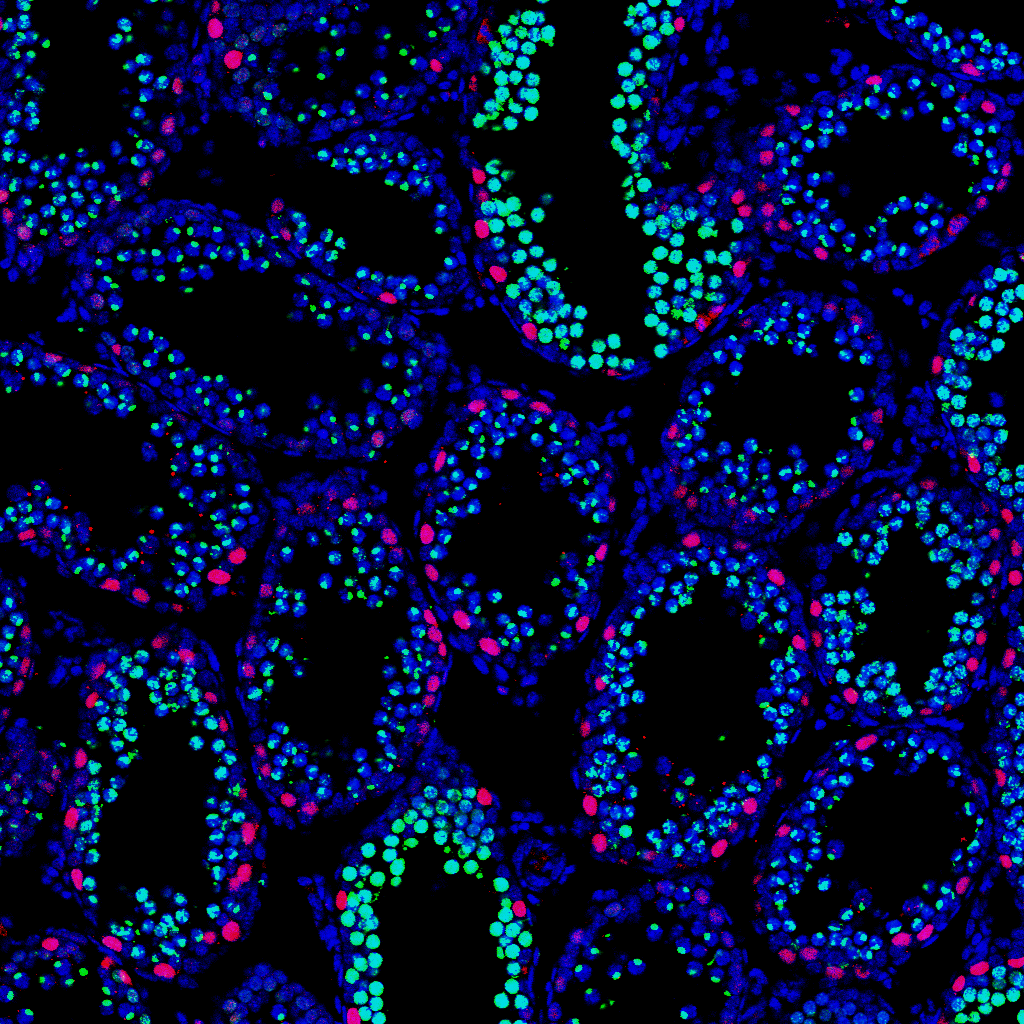

Supplement: Supplementary file 10 — Source data Fig. 7 [file 44319_2025_487_MOESM10_ESM.zip › Figure 7/7D/PD14 testis anti-SOX3&γH2AX/PD14 Control testis anti-SOX3&γH2AX Hoechst_overlay.tif]

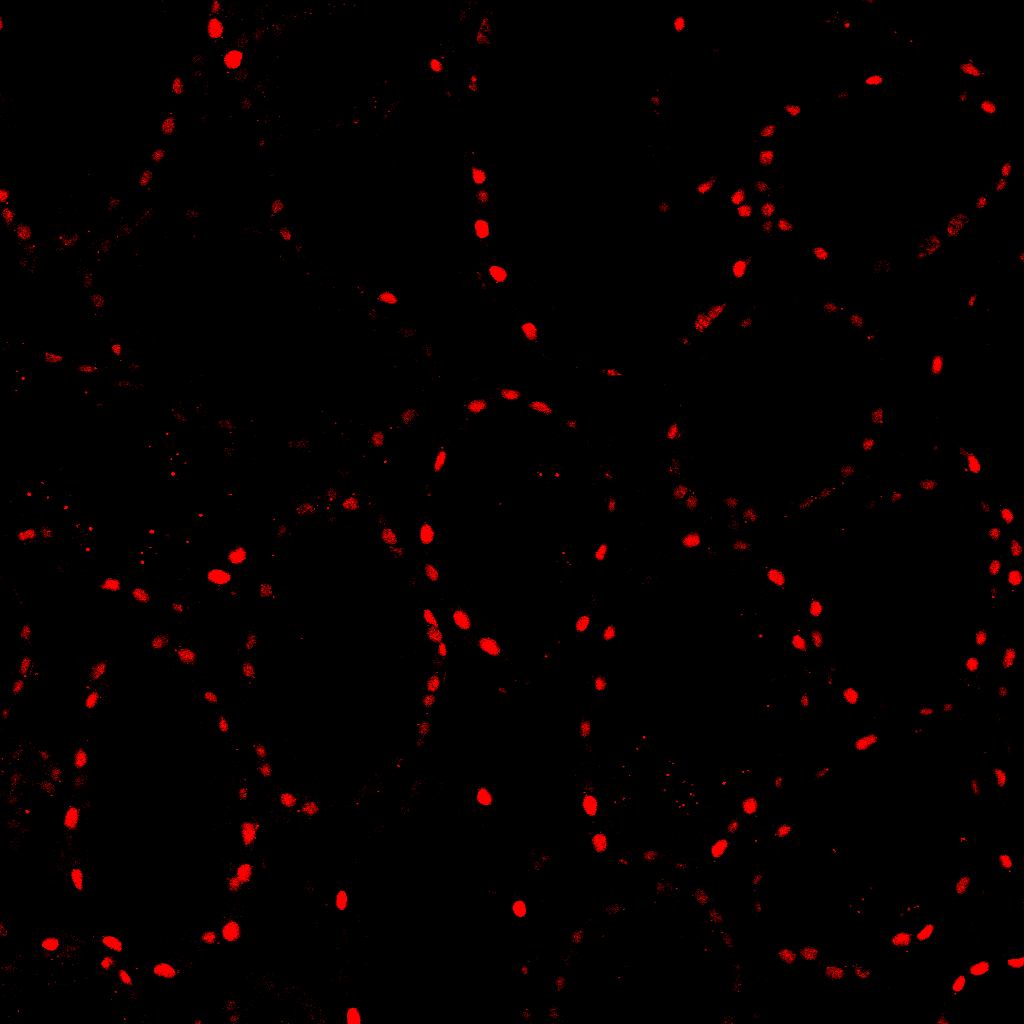

Supplement: Supplementary file 10 — Source data Fig. 7 [file 44319_2025_487_MOESM10_ESM.zip › Figure 7/7D/PD14 testis anti-SOX3&γH2AX/PD14 Control testis anti-SOX3.tif]

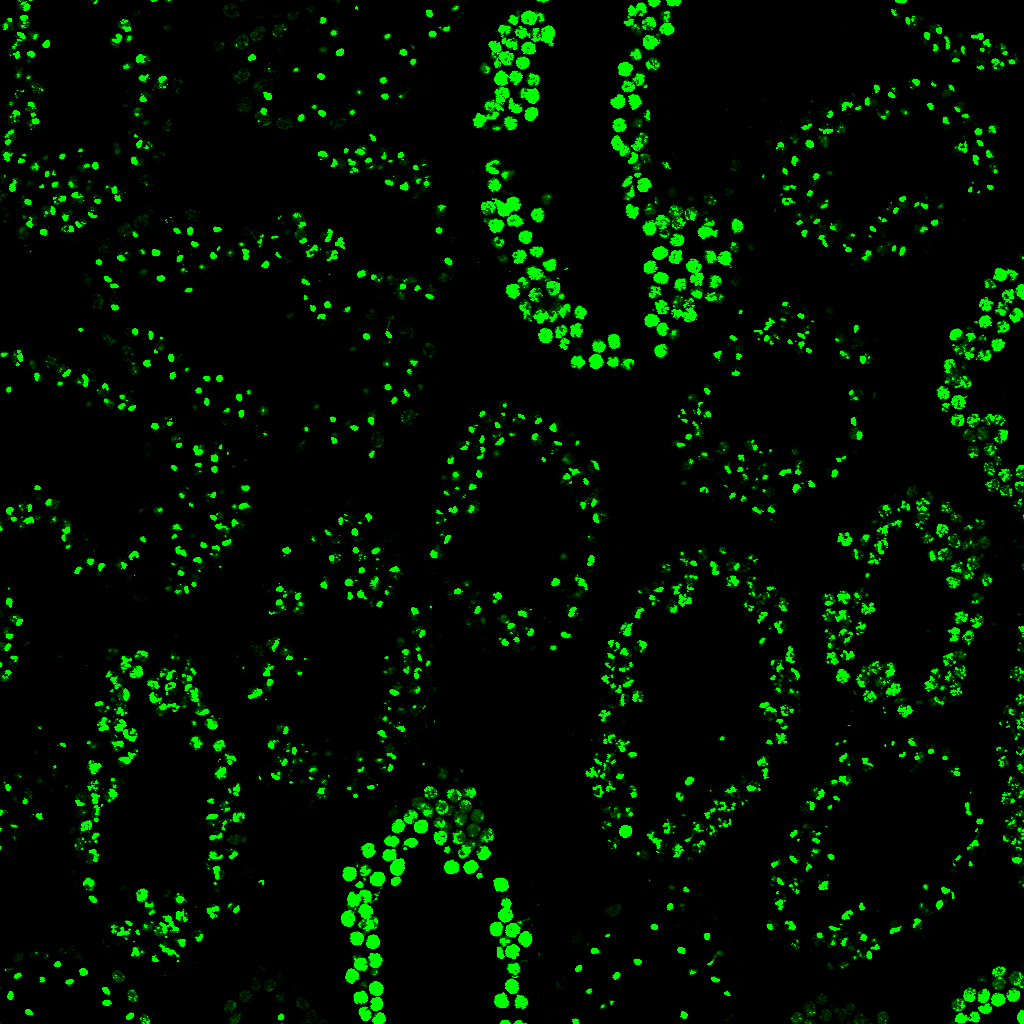

Supplement: Supplementary file 10 — Source data Fig. 7 [file 44319_2025_487_MOESM10_ESM.zip › Figure 7/7D/PD14 testis anti-SOX3&γH2AX/PD14 Control testis anti-γH2AX.tif]

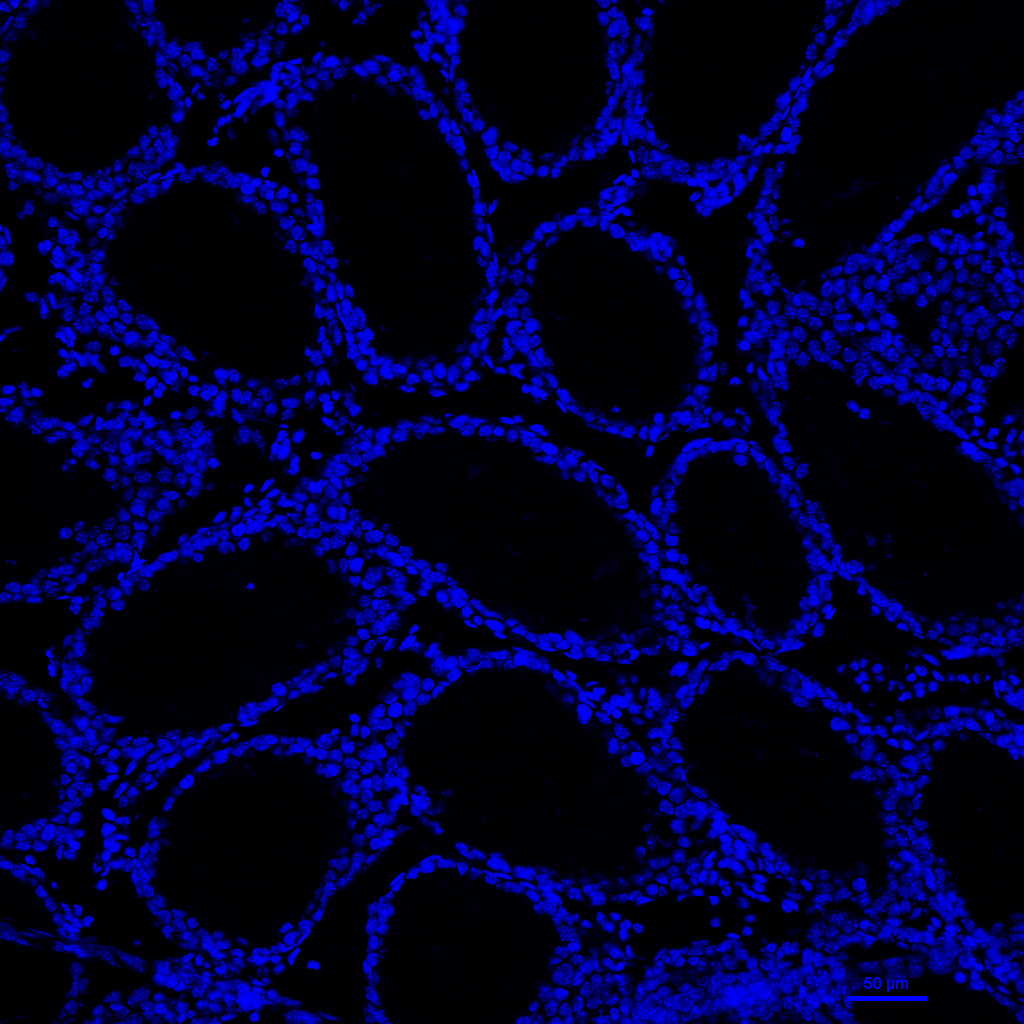

Supplement: Supplementary file 10 — Source data Fig. 7 [file 44319_2025_487_MOESM10_ESM.zip › Figure 7/7G/PD21 testis anti-GFRa1&γH2AX/PD21 Brca1 vKO testis anti-GFRa1&γH2AX Hoechst.tif]

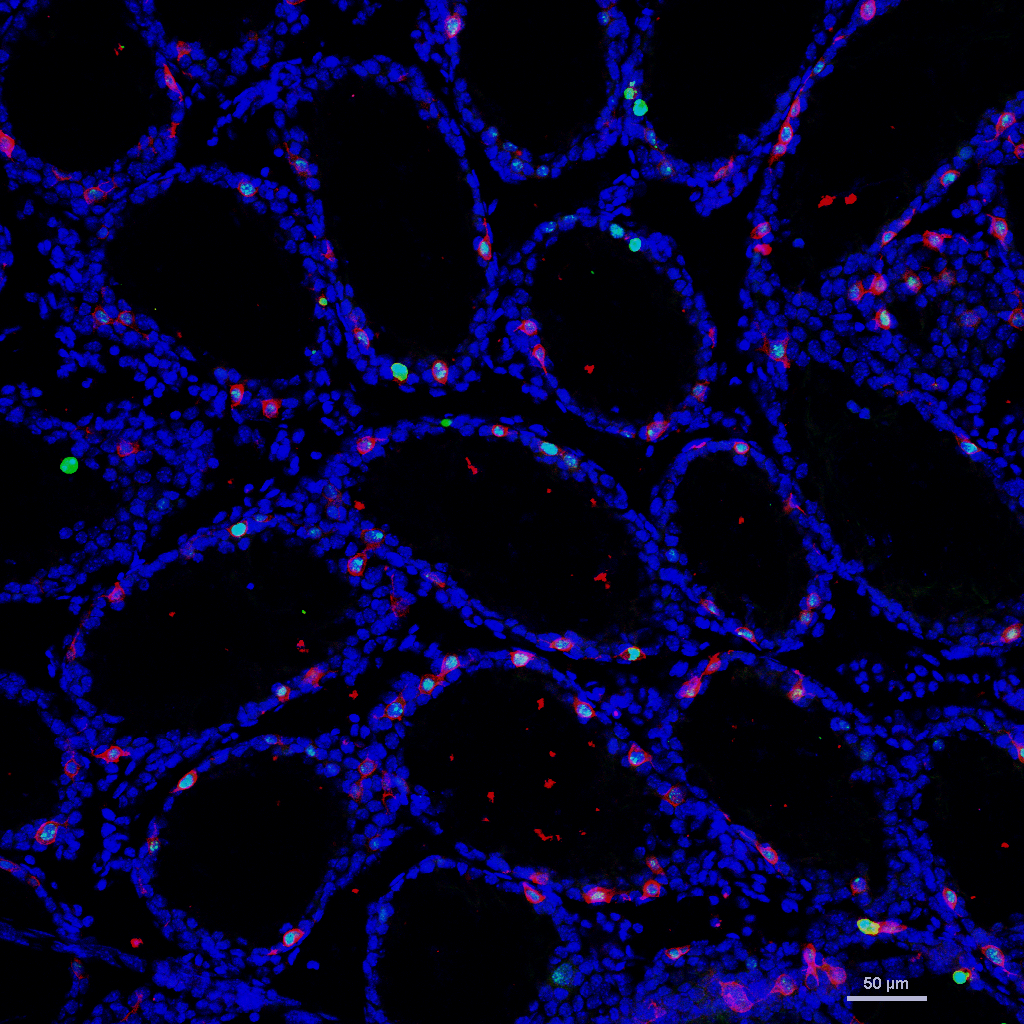

Supplement: Supplementary file 10 — Source data Fig. 7 [file 44319_2025_487_MOESM10_ESM.zip › Figure 7/7G/PD21 testis anti-GFRa1&γH2AX/PD21 Brca1 vKO testis anti-GFRa1&γH2AX Hoechst_overlay.tif]

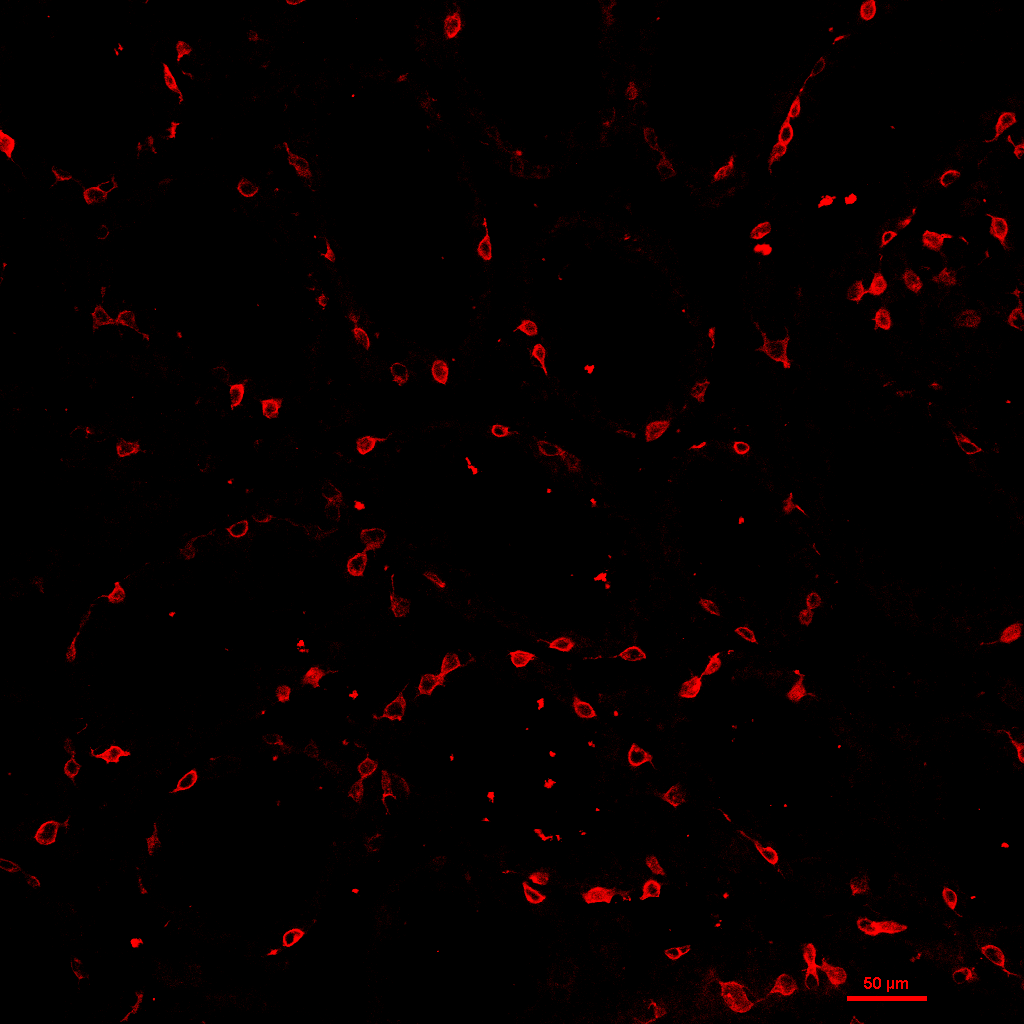

Supplement: Supplementary file 10 — Source data Fig. 7 [file 44319_2025_487_MOESM10_ESM.zip › Figure 7/7G/PD21 testis anti-GFRa1&γH2AX/PD21 Brca1 vKO testis anti-GFRa1.tif]

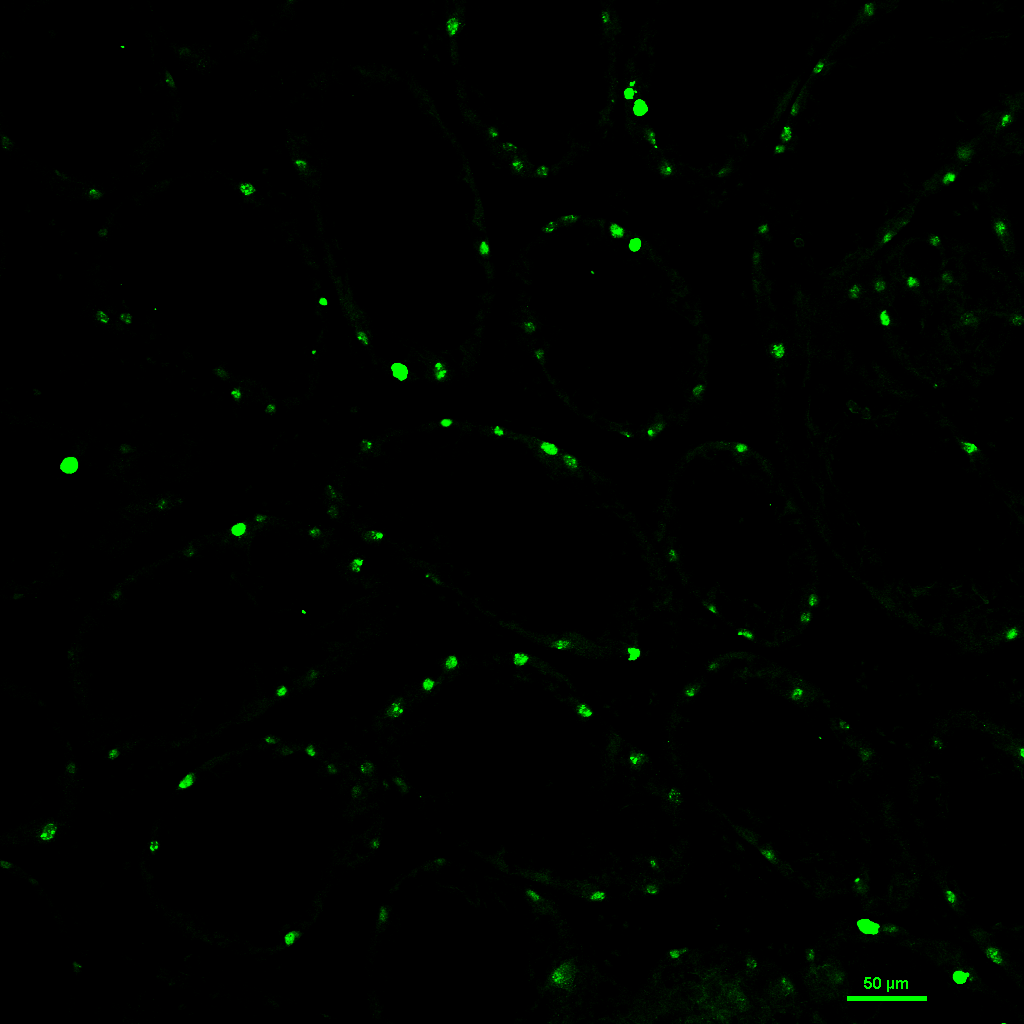

Supplement: Supplementary file 10 — Source data Fig. 7 [file 44319_2025_487_MOESM10_ESM.zip › Figure 7/7G/PD21 testis anti-GFRa1&γH2AX/PD21 Brca1 vKO testis anti-γH2AX.tif]

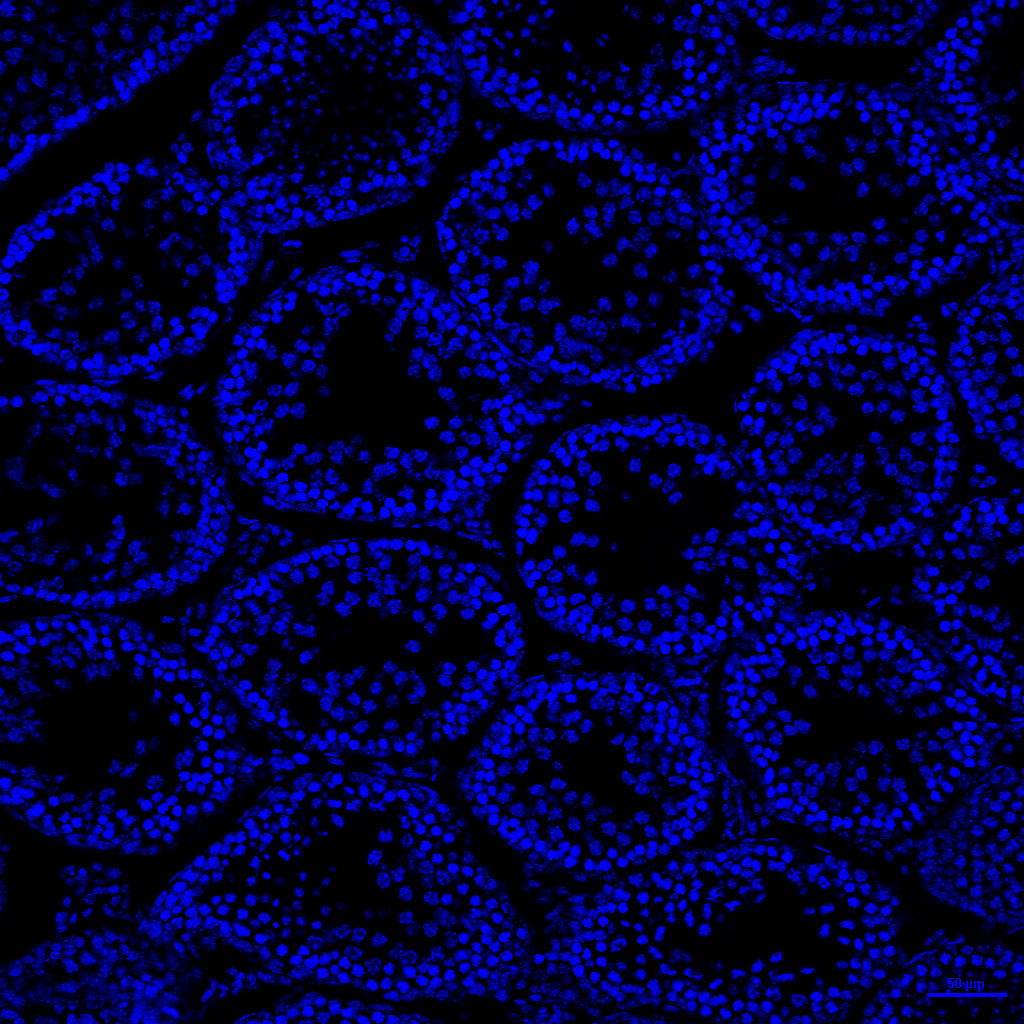

Supplement: Supplementary file 10 — Source data Fig. 7 [file 44319_2025_487_MOESM10_ESM.zip › Figure 7/7G/PD21 testis anti-GFRa1&γH2AX/PD21 Control testis anti-GFRa1&γH2AX Hoechst.tif]

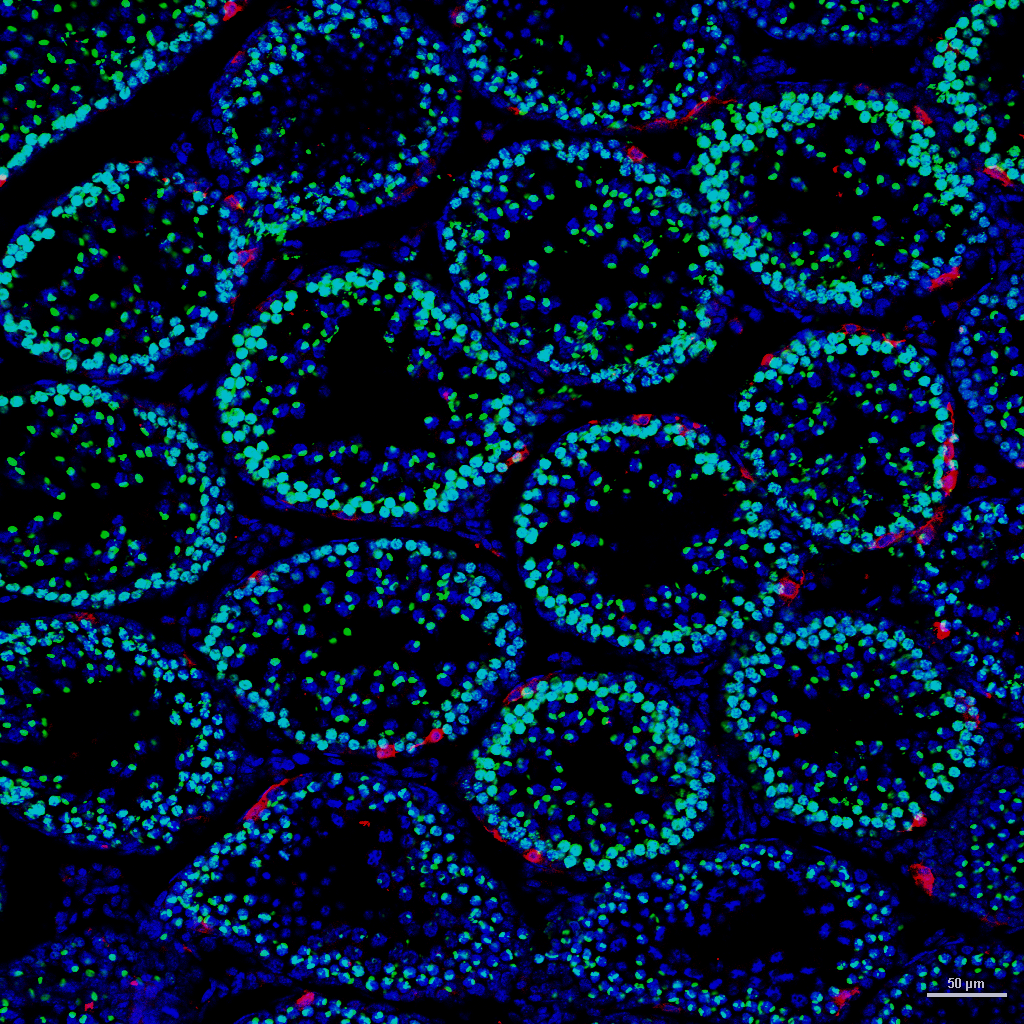

Supplement: Supplementary file 10 — Source data Fig. 7 [file 44319_2025_487_MOESM10_ESM.zip › Figure 7/7G/PD21 testis anti-GFRa1&γH2AX/PD21 Control testis anti-GFRa1&γH2AX Hoechst_overlay.tif]

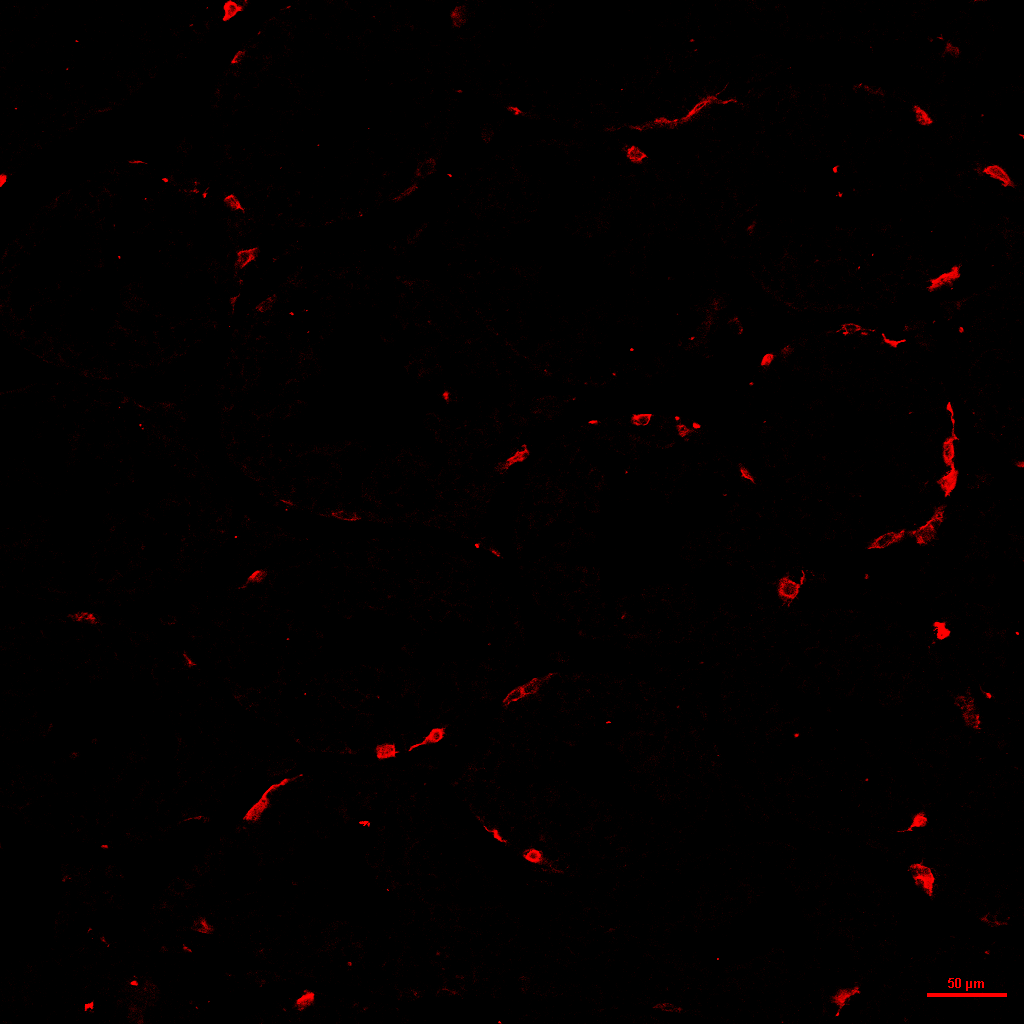

Supplement: Supplementary file 10 — Source data Fig. 7 [file 44319_2025_487_MOESM10_ESM.zip › Figure 7/7G/PD21 testis anti-GFRa1&γH2AX/PD21 Control testis anti-GFRa1.tif]

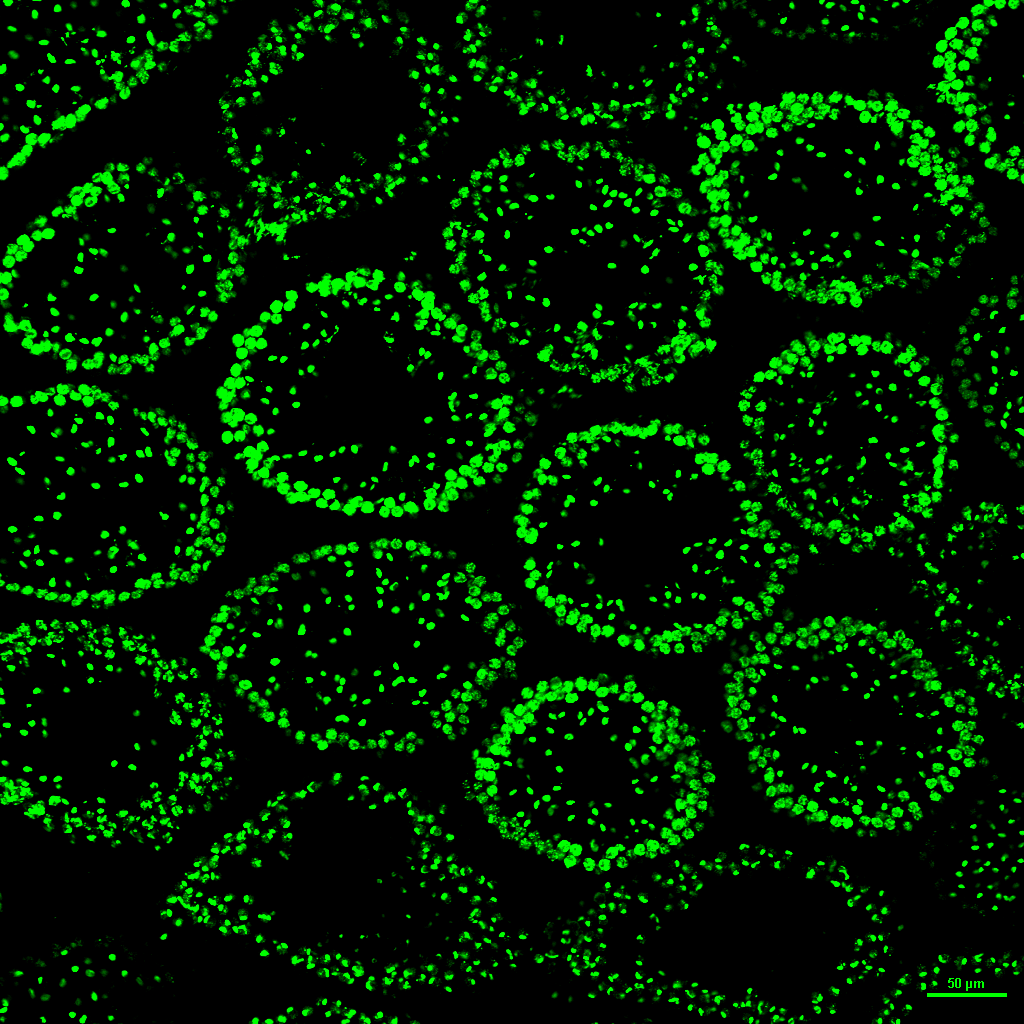

Supplement: Supplementary file 10 — Source data Fig. 7 [file 44319_2025_487_MOESM10_ESM.zip › Figure 7/7G/PD21 testis anti-GFRa1&γH2AX/PD21 Control testis anti-γH2AX.tif]

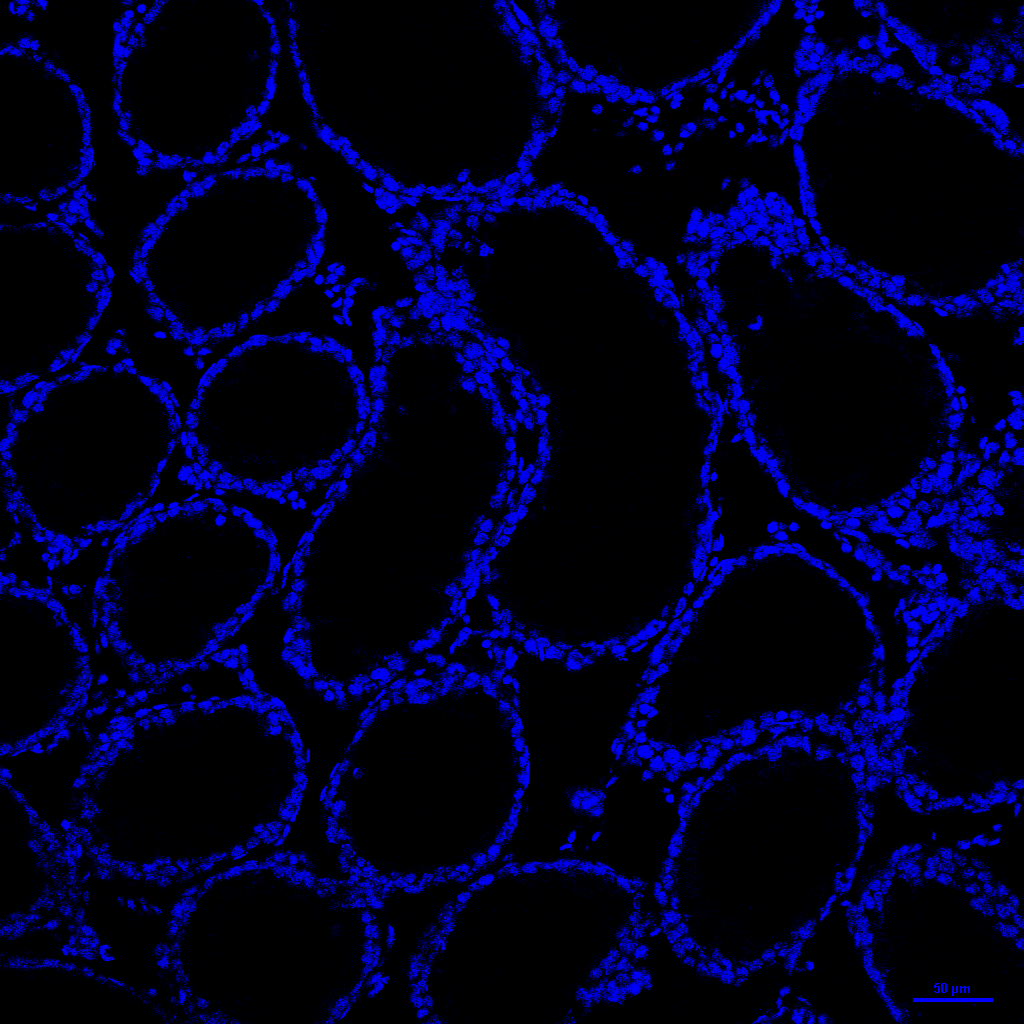

Supplement: Supplementary file 10 — Source data Fig. 7 [file 44319_2025_487_MOESM10_ESM.zip › Figure 7/7G/PD21 testis anti-SOX3&γH2AX/PD21 Brca1 vKO testis anti-SOX3&γH2AX Hoechst.tif]

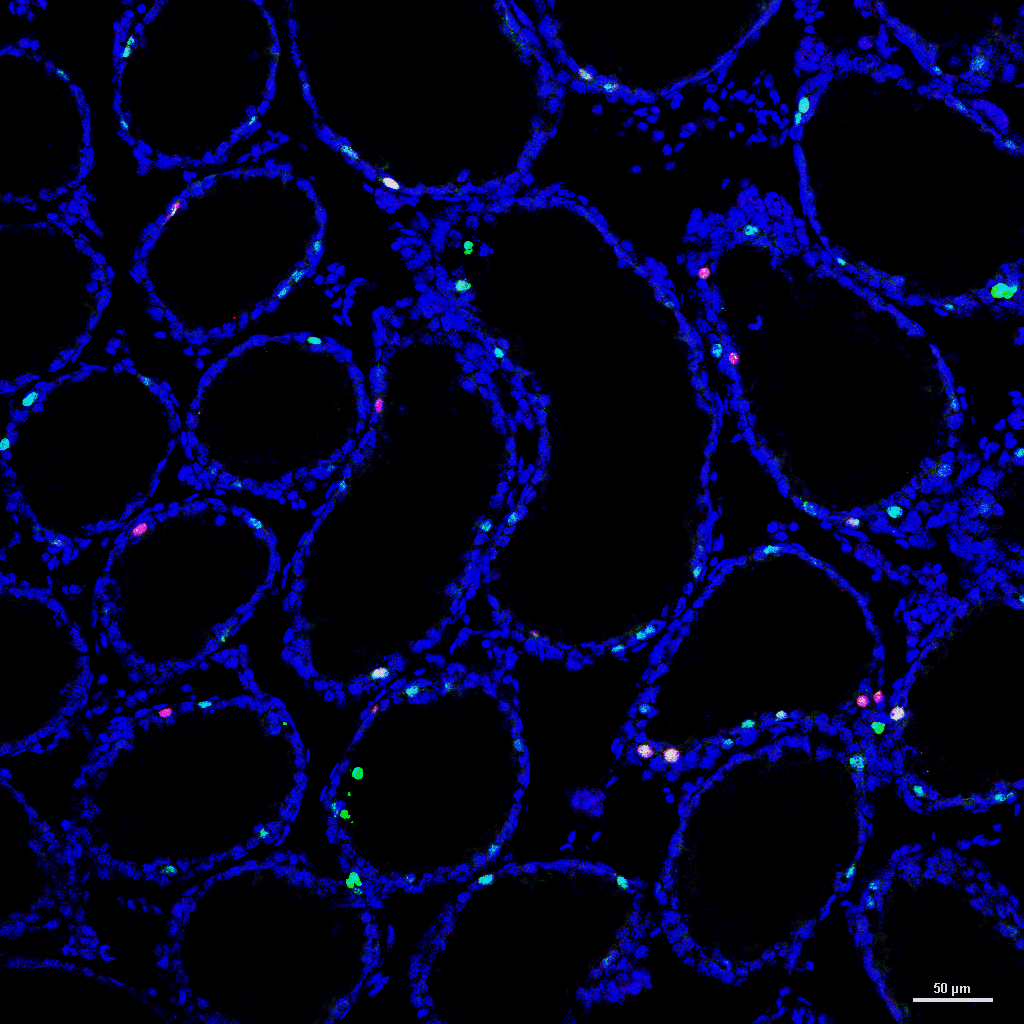

Supplement: Supplementary file 10 — Source data Fig. 7 [file 44319_2025_487_MOESM10_ESM.zip › Figure 7/7G/PD21 testis anti-SOX3&γH2AX/PD21 Brca1 vKO testis anti-SOX3&γH2AX Hoechst_overlay.tif]

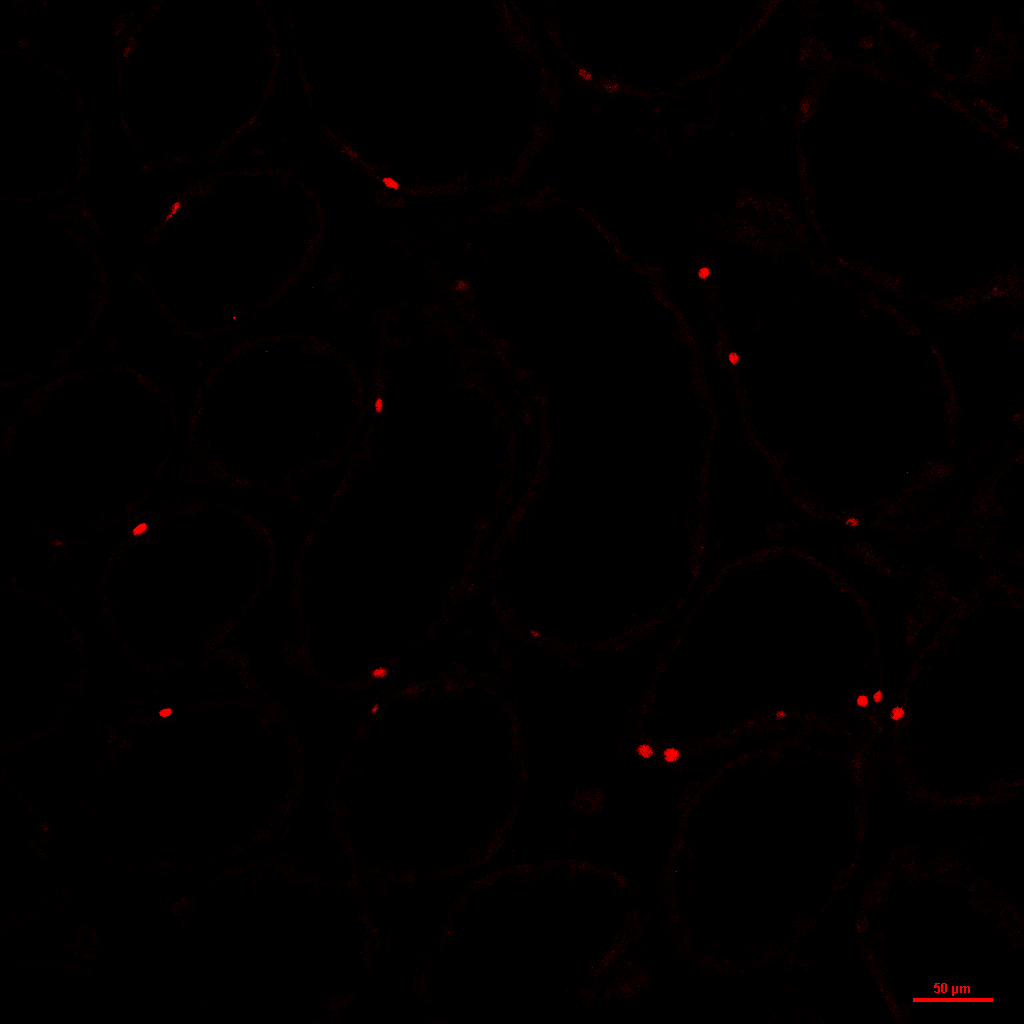

Supplement: Supplementary file 10 — Source data Fig. 7 [file 44319_2025_487_MOESM10_ESM.zip › Figure 7/7G/PD21 testis anti-SOX3&γH2AX/PD21 Brca1 vKO testis anti-SOX3.tif]

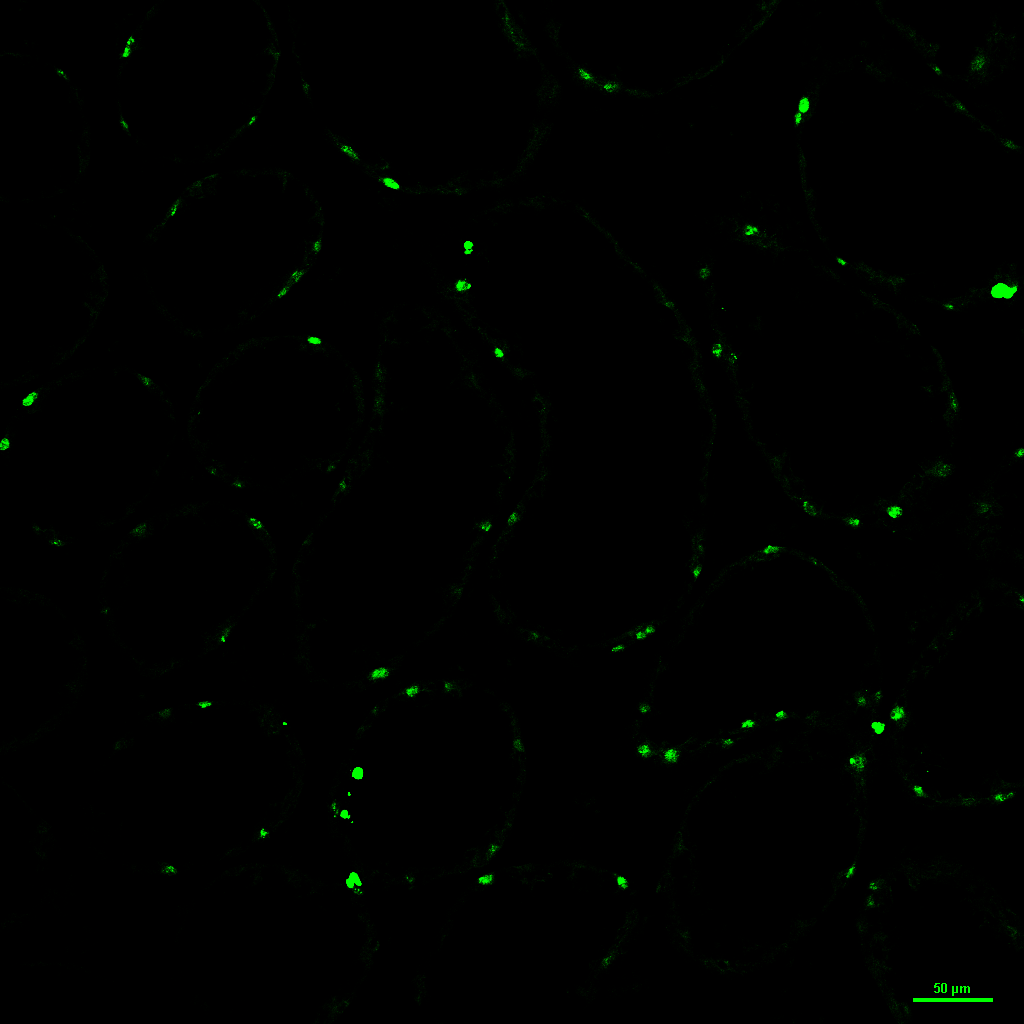

Supplement: Supplementary file 10 — Source data Fig. 7 [file 44319_2025_487_MOESM10_ESM.zip › Figure 7/7G/PD21 testis anti-SOX3&γH2AX/PD21 Brca1 vKO testis anti-γH2AX.tif]

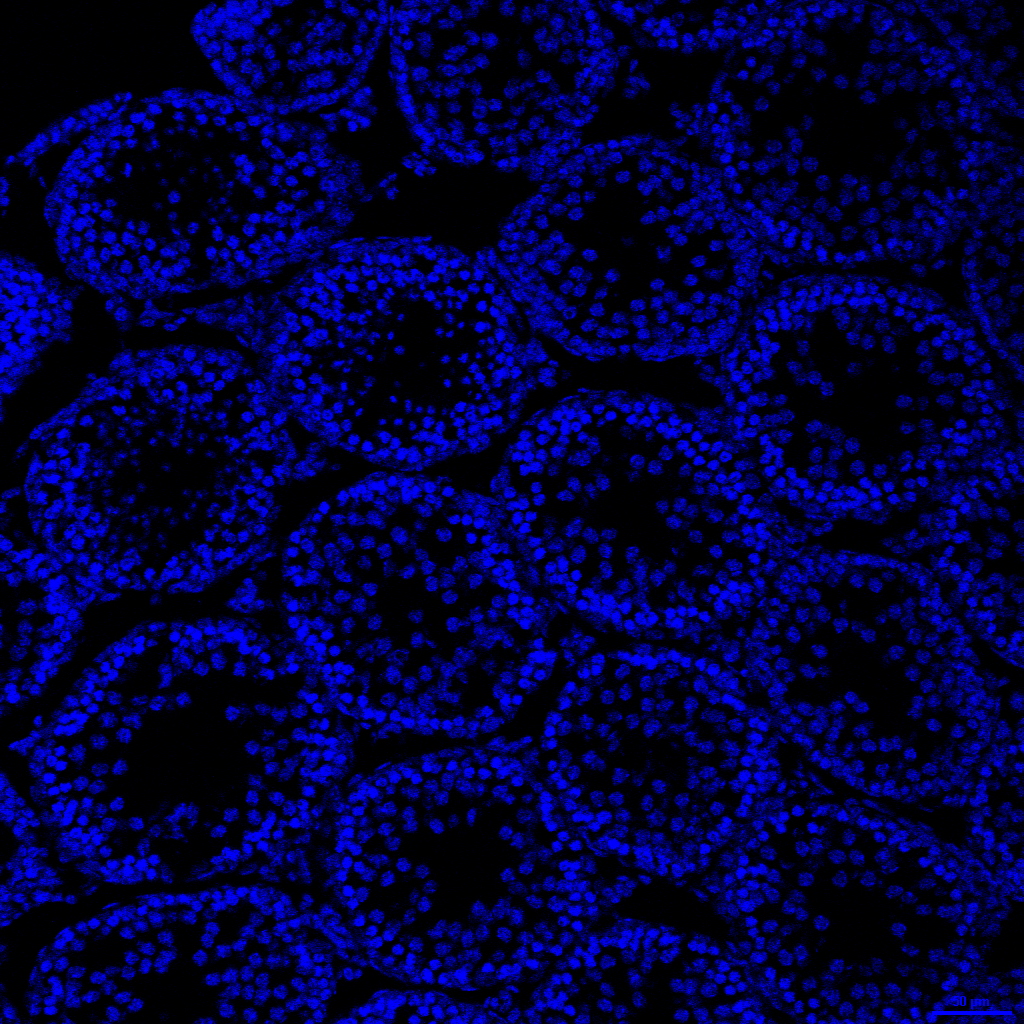

Supplement: Supplementary file 10 — Source data Fig. 7 [file 44319_2025_487_MOESM10_ESM.zip › Figure 7/7G/PD21 testis anti-SOX3&γH2AX/PD21 Control testis anti-SOX3&γH2AX Hoechst.tif]

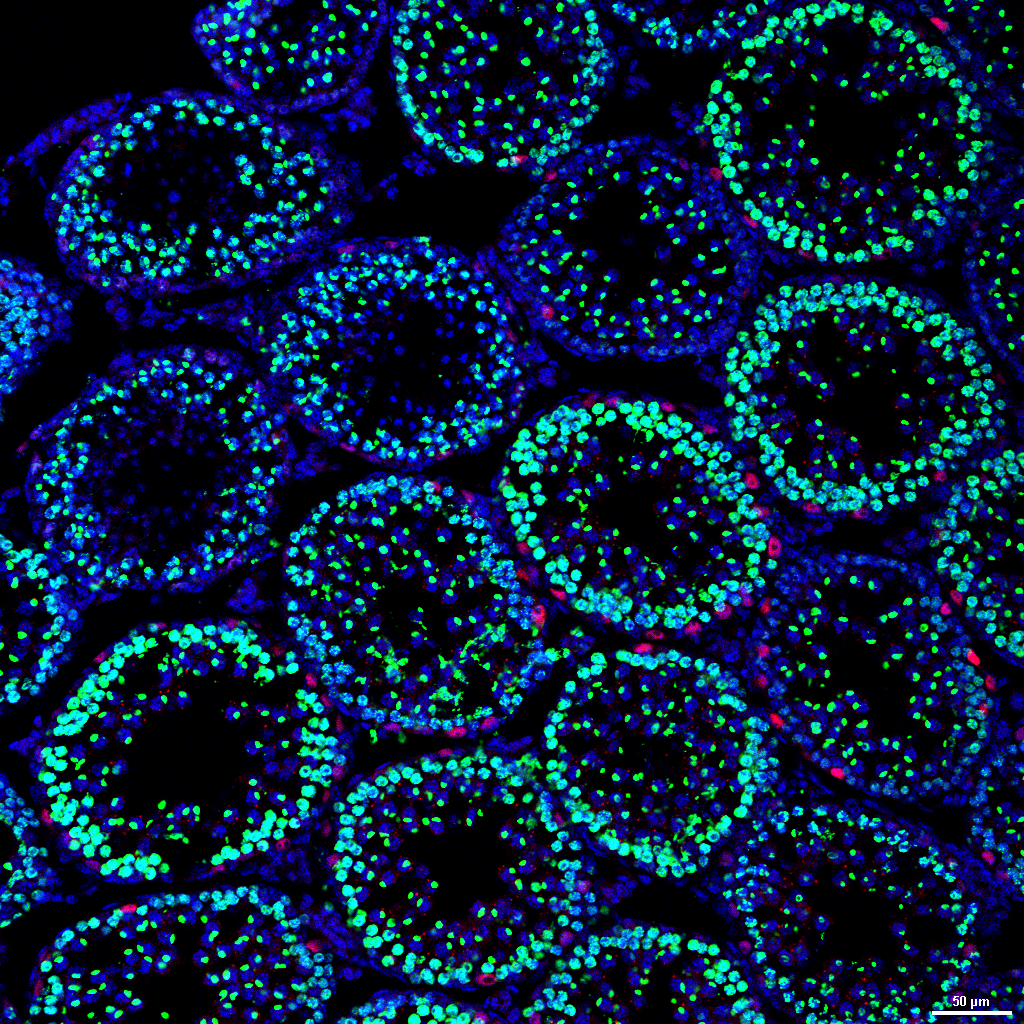

Supplement: Supplementary file 10 — Source data Fig. 7 [file 44319_2025_487_MOESM10_ESM.zip › Figure 7/7G/PD21 testis anti-SOX3&γH2AX/PD21 Control testis anti-SOX3&γH2AX Hoechst_overlay.tif]

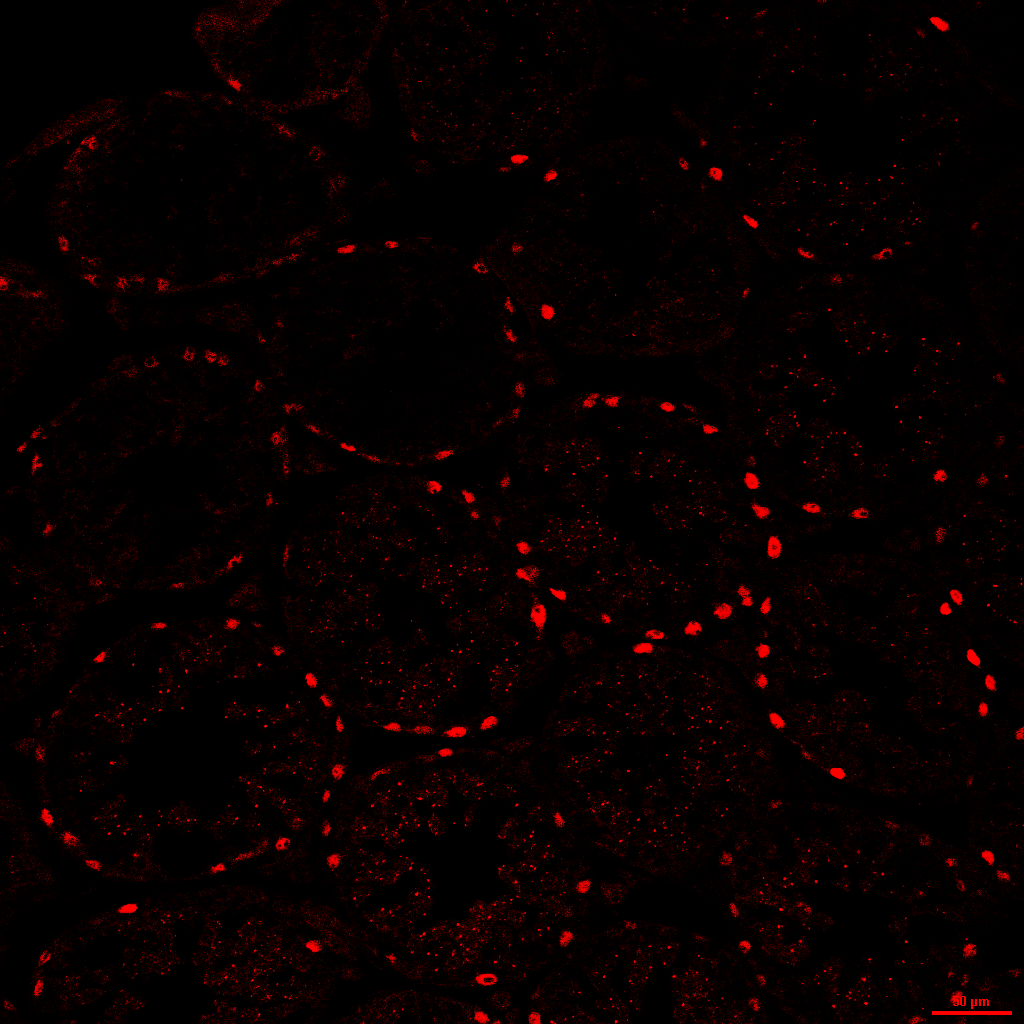

Supplement: Supplementary file 10 — Source data Fig. 7 [file 44319_2025_487_MOESM10_ESM.zip › Figure 7/7G/PD21 testis anti-SOX3&γH2AX/PD21 Control testis anti-SOX3.tif]

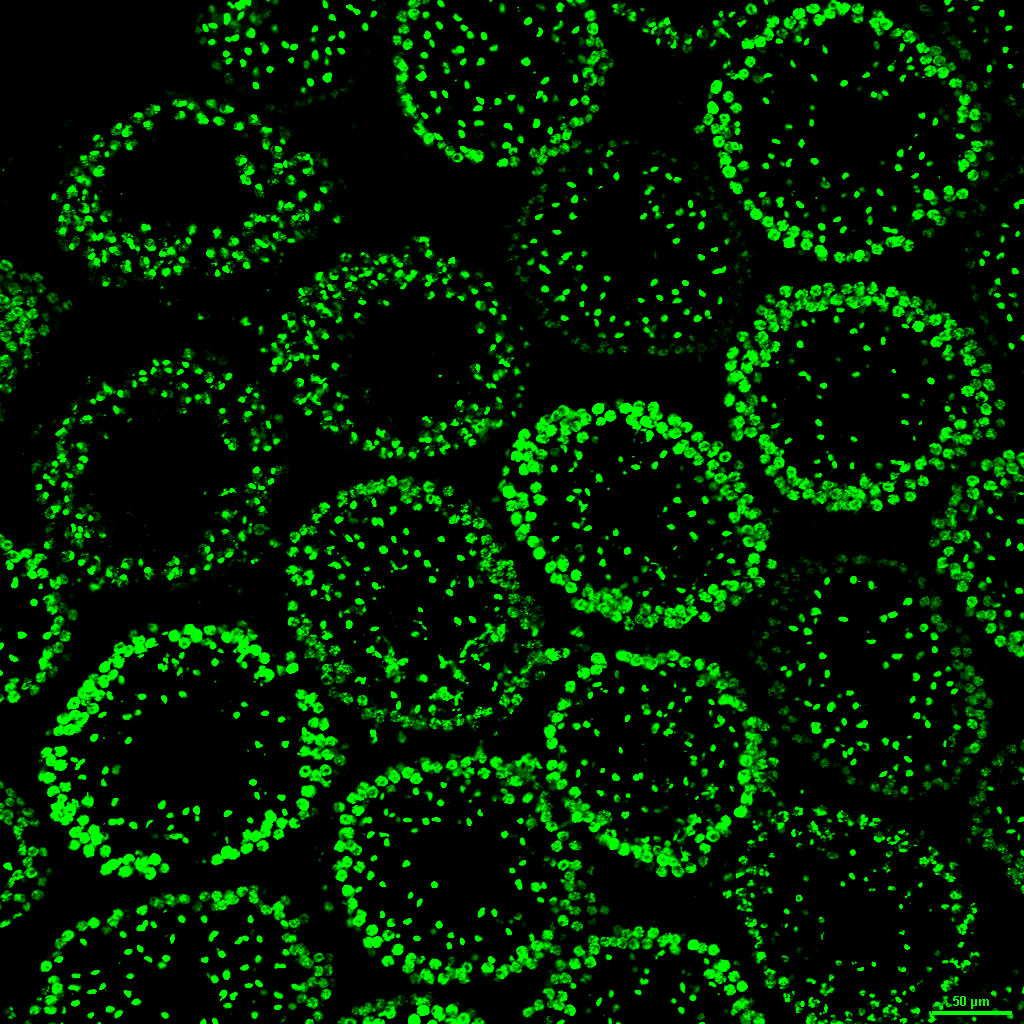

Supplement: Supplementary file 10 — Source data Fig. 7 [file 44319_2025_487_MOESM10_ESM.zip › Figure 7/7G/PD21 testis anti-SOX3&γH2AX/PD21 Control testis anti-γH2AX.tif]

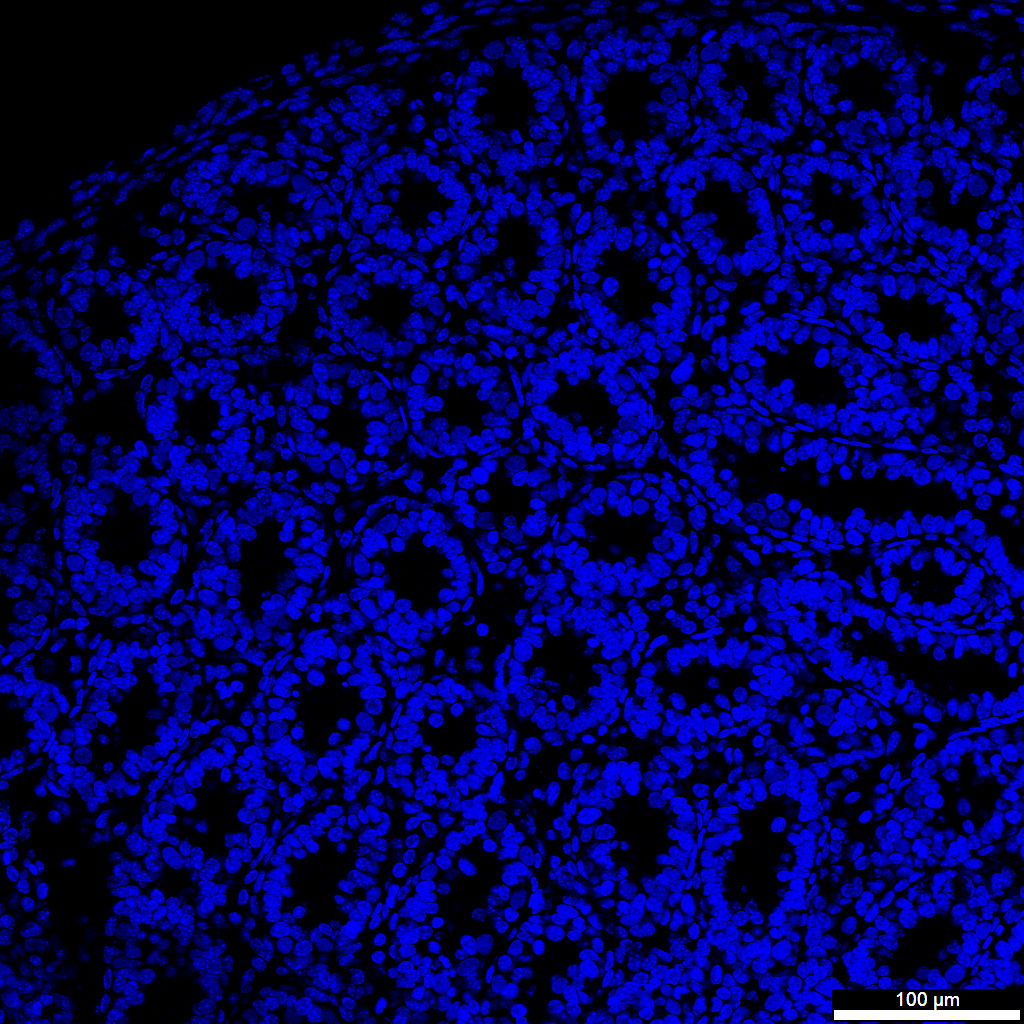

Supplement: Supplementary file 10 — Source data Fig. 7 [file 44319_2025_487_MOESM10_ESM.zip › Figure 7/7J/PD7 WT testis anti-GFRa1&p-H3(S10)/PD7 WT testis anti-GFRa1&p-H3(S10) Hoechst.tif]

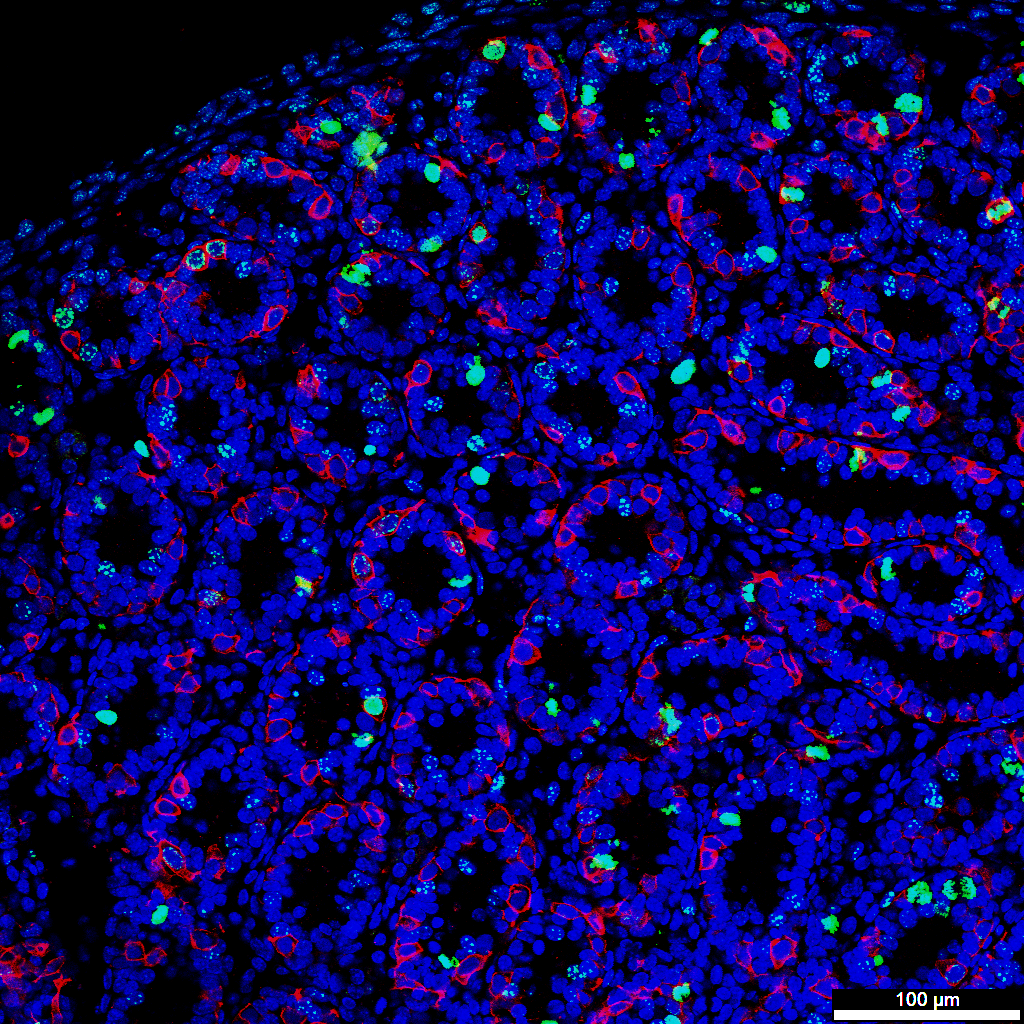

Supplement: Supplementary file 10 — Source data Fig. 7 [file 44319_2025_487_MOESM10_ESM.zip › Figure 7/7J/PD7 WT testis anti-GFRa1&p-H3(S10)/PD7 WT testis anti-GFRa1&p-H3(S10) Hoechst_overlay.tif]

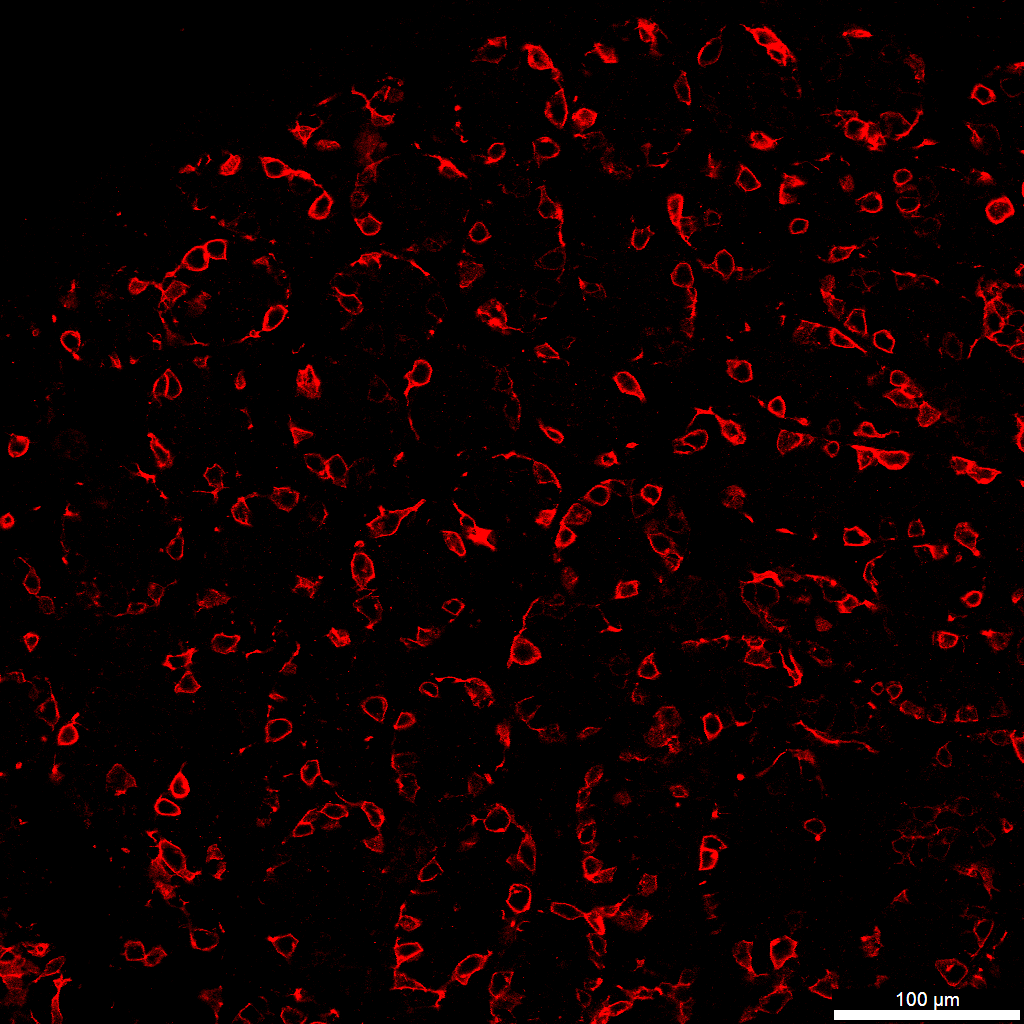

Supplement: Supplementary file 10 — Source data Fig. 7 [file 44319_2025_487_MOESM10_ESM.zip › Figure 7/7J/PD7 WT testis anti-GFRa1&p-H3(S10)/PD7 WT testis anti-GFRa1.tif]

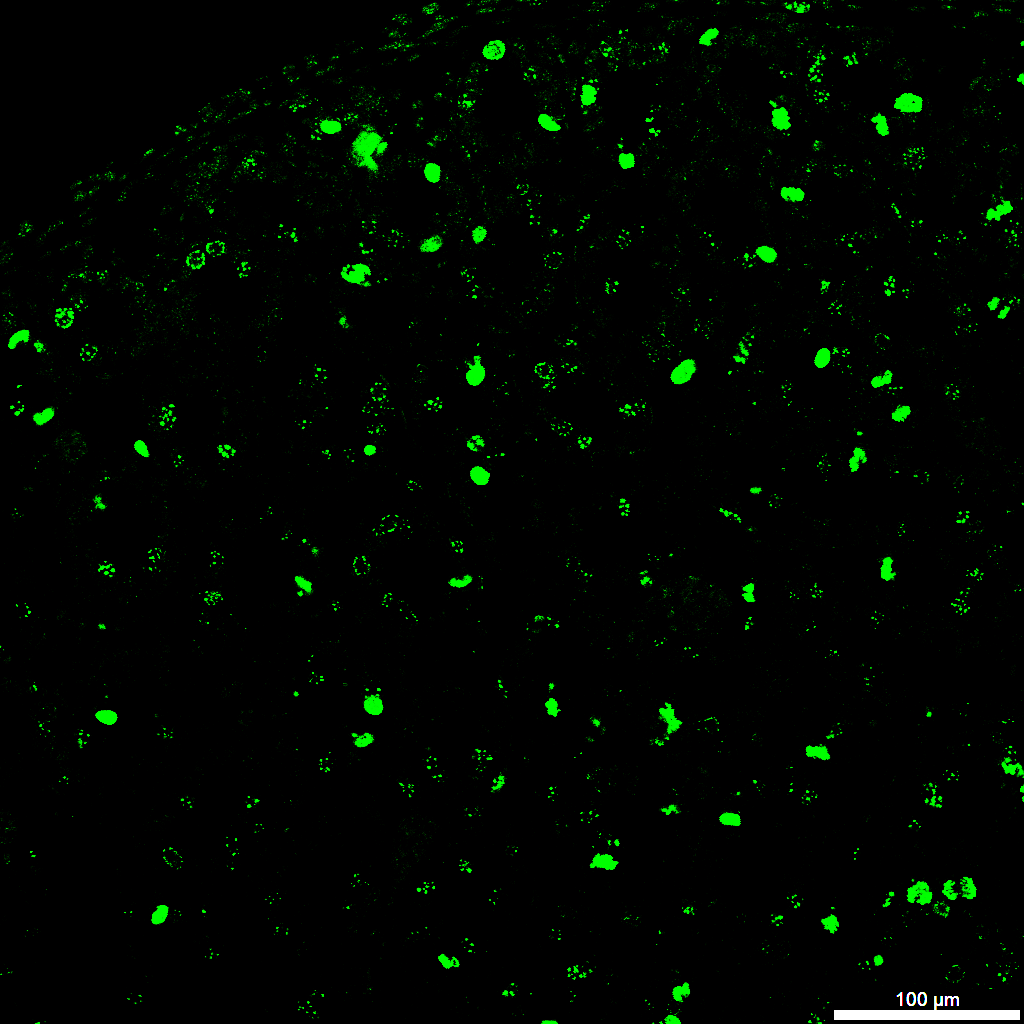

Supplement: Supplementary file 10 — Source data Fig. 7 [file 44319_2025_487_MOESM10_ESM.zip › Figure 7/7J/PD7 WT testis anti-GFRa1&p-H3(S10)/PD7 WT testis anti-p-H3(S10).tif]

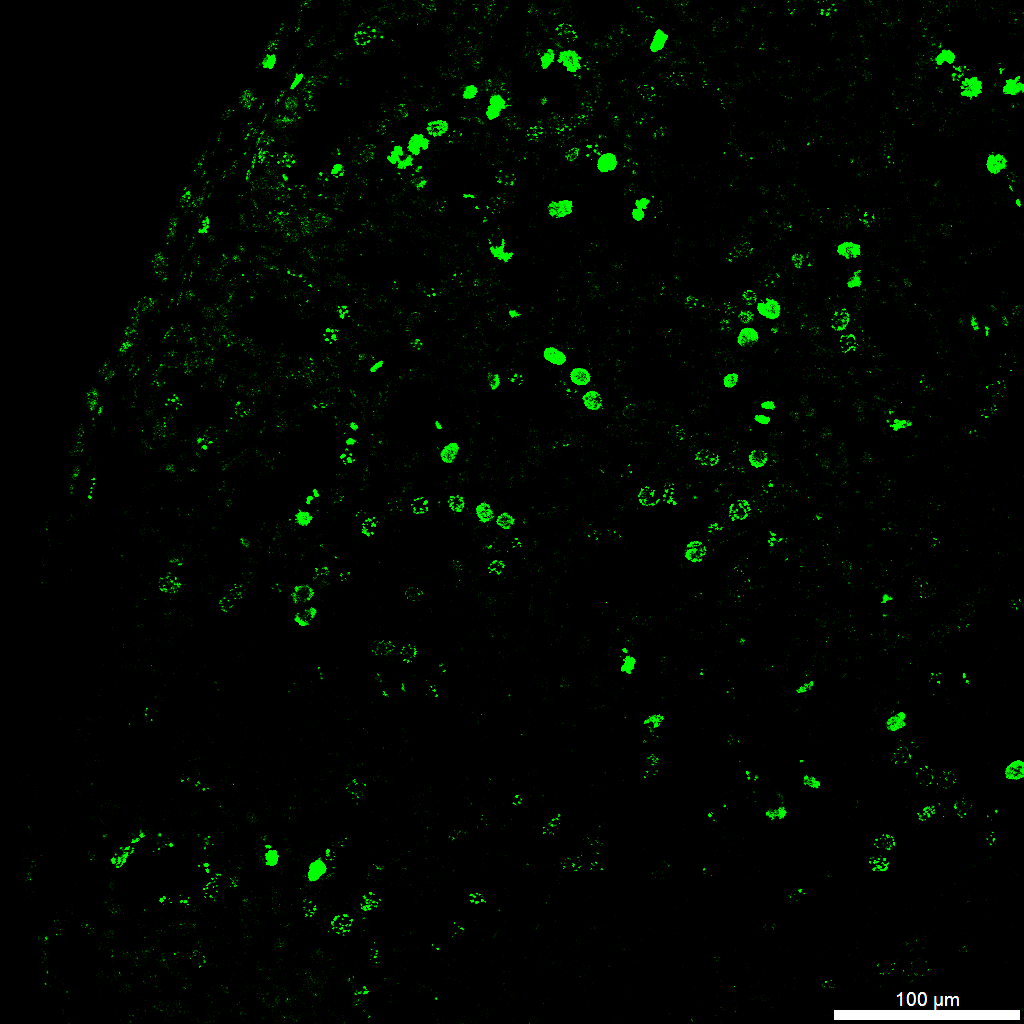

Supplement: Supplementary file 10 — Source data Fig. 7 [file 44319_2025_487_MOESM10_ESM.zip › Figure 7/7J/PD7 WT testis anti-SOX3&p-H3(S10)/PD7 WT testis anti-p-H3(S10).tif]

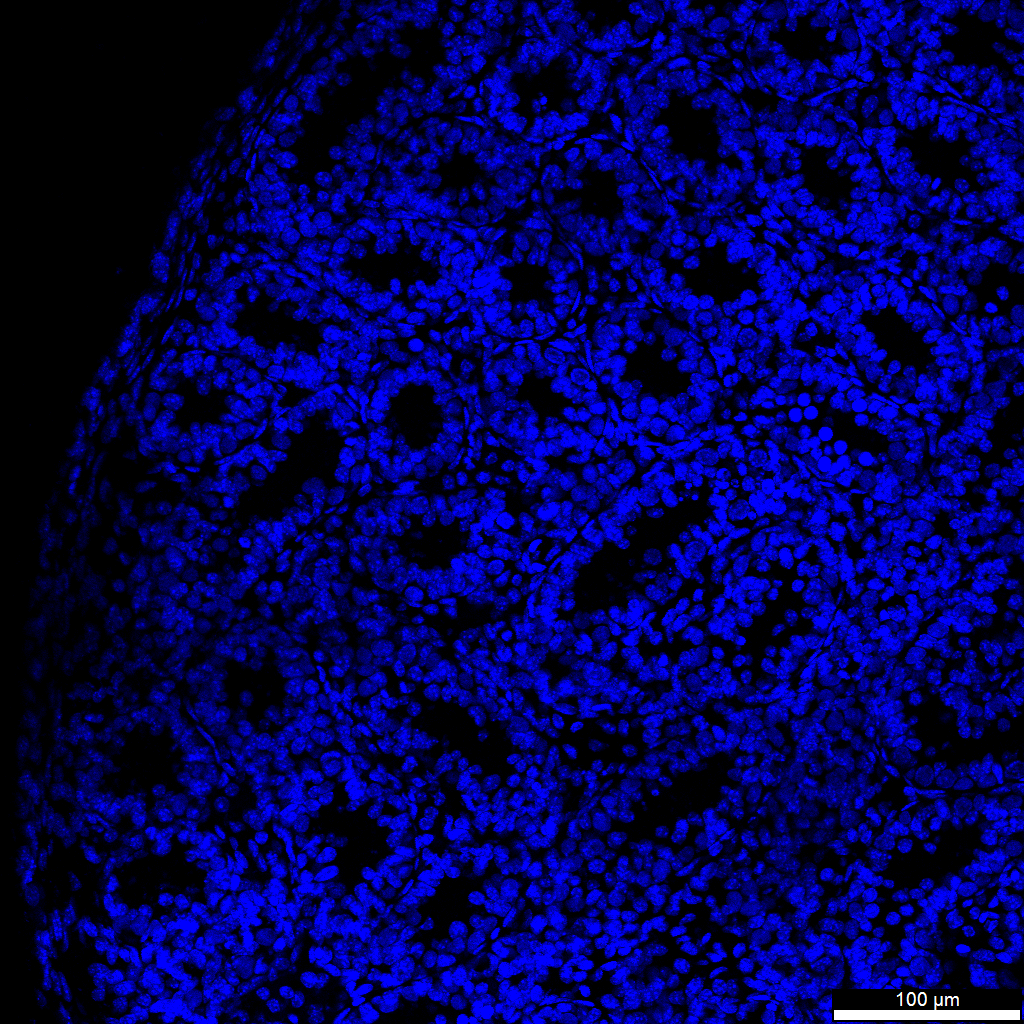

Supplement: Supplementary file 10 — Source data Fig. 7 [file 44319_2025_487_MOESM10_ESM.zip › Figure 7/7J/PD7 WT testis anti-SOX3&p-H3(S10)/PD7 WT testis anti-SOX3&p-H3(S10) Hoechst.tif]

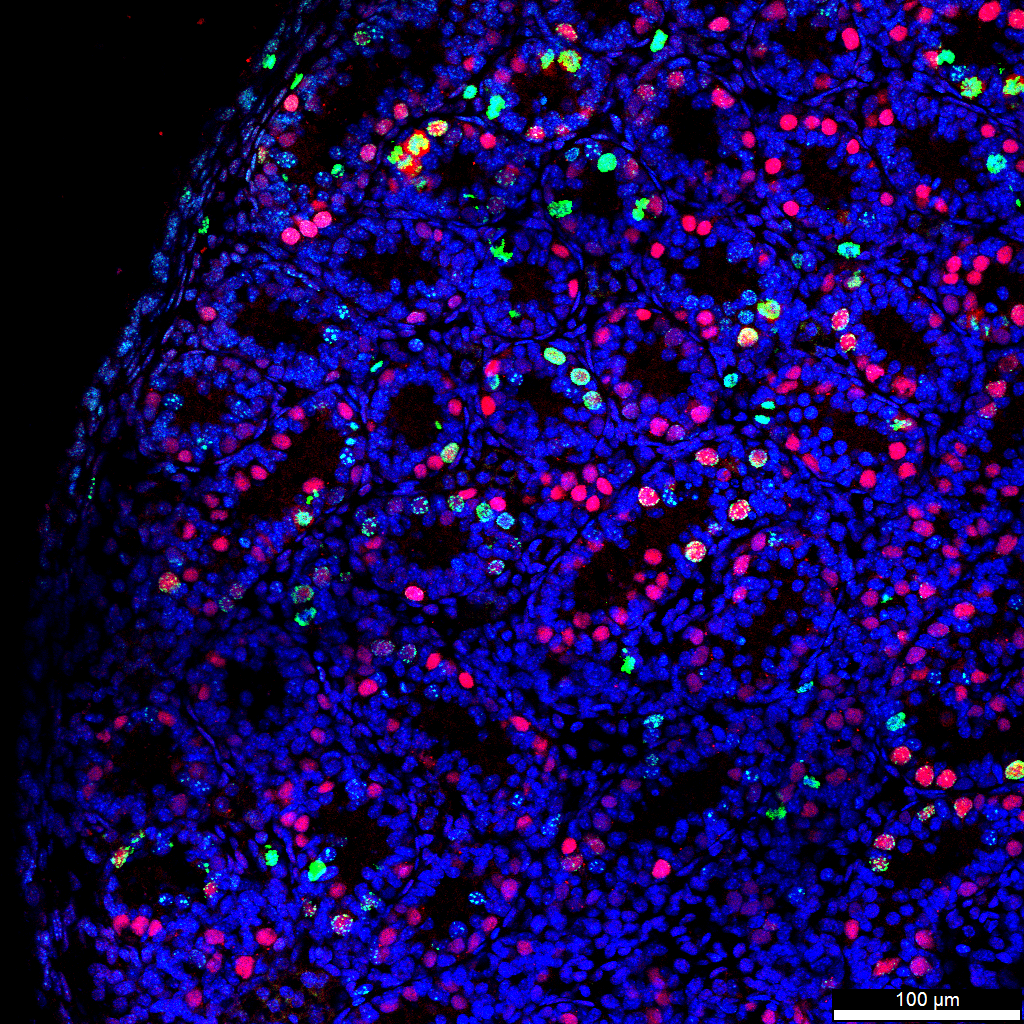

Supplement: Supplementary file 10 — Source data Fig. 7 [file 44319_2025_487_MOESM10_ESM.zip › Figure 7/7J/PD7 WT testis anti-SOX3&p-H3(S10)/PD7 WT testis anti-SOX3&p-H3(S10) Hoechst_overlay.tif]

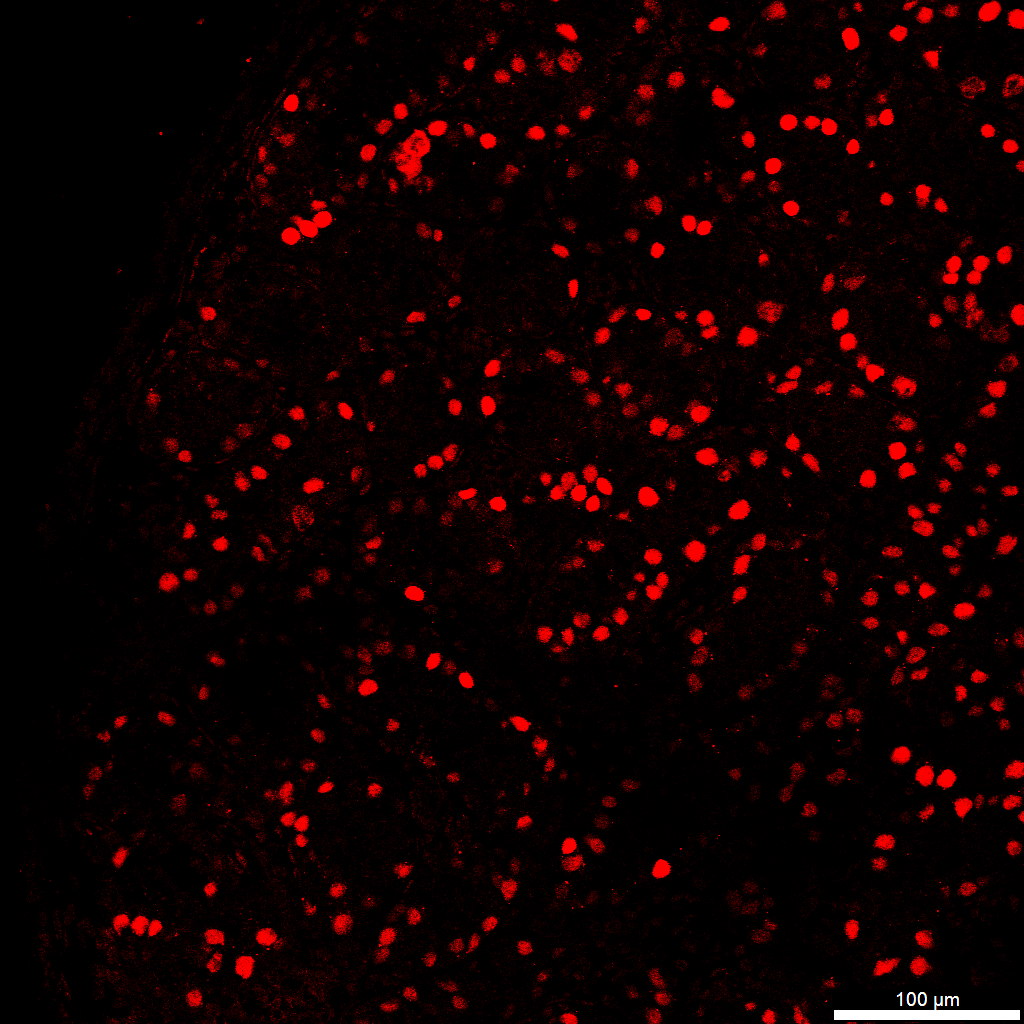

Supplement: Supplementary file 10 — Source data Fig. 7 [file 44319_2025_487_MOESM10_ESM.zip › Figure 7/7J/PD7 WT testis anti-SOX3&p-H3(S10)/PD7 WT testis anti-SOX3.tif]

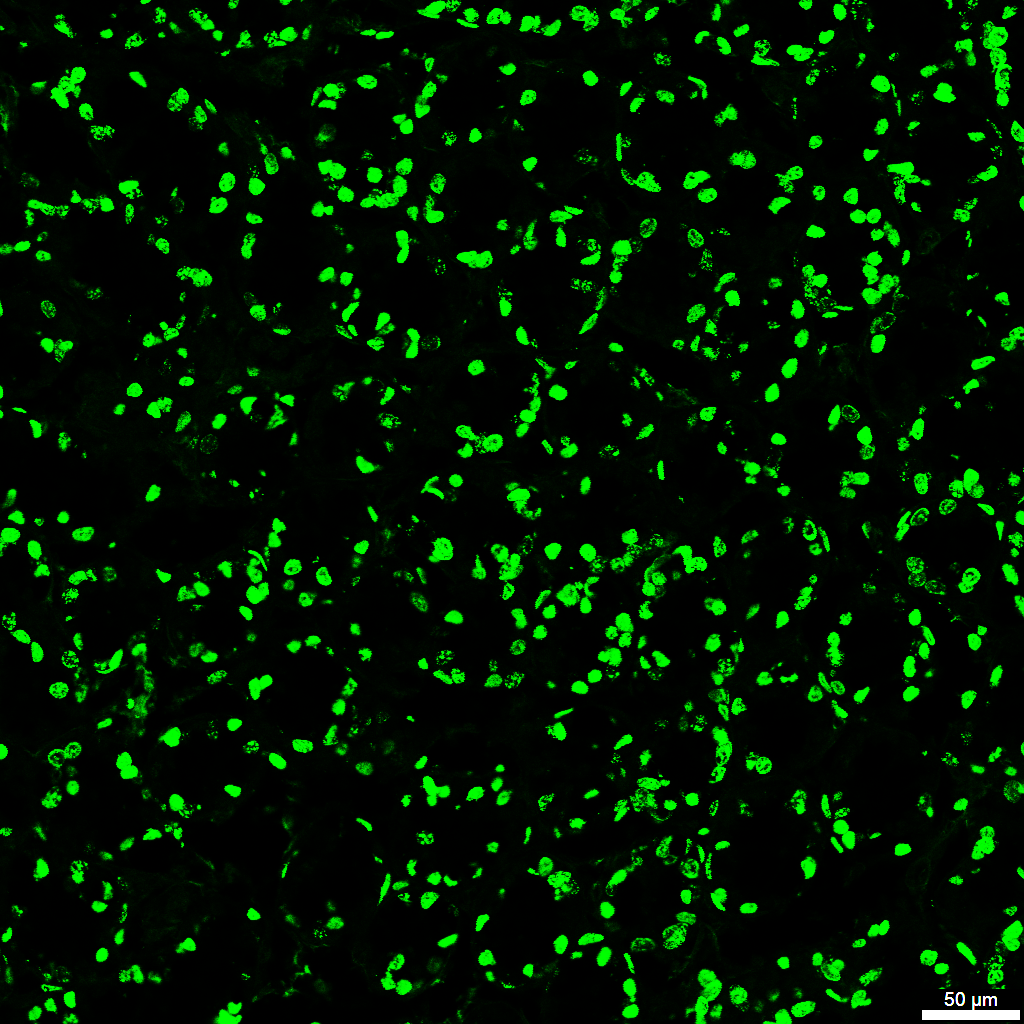

Supplement: Supplementary file 11 — Source data Fig. 8 [file 44319_2025_487_MOESM11_ESM.zip › Figure 8/8A/PD4 WT testis anti-PLZF&BrdU/PD4 WT testis anti-BrdU.tif]

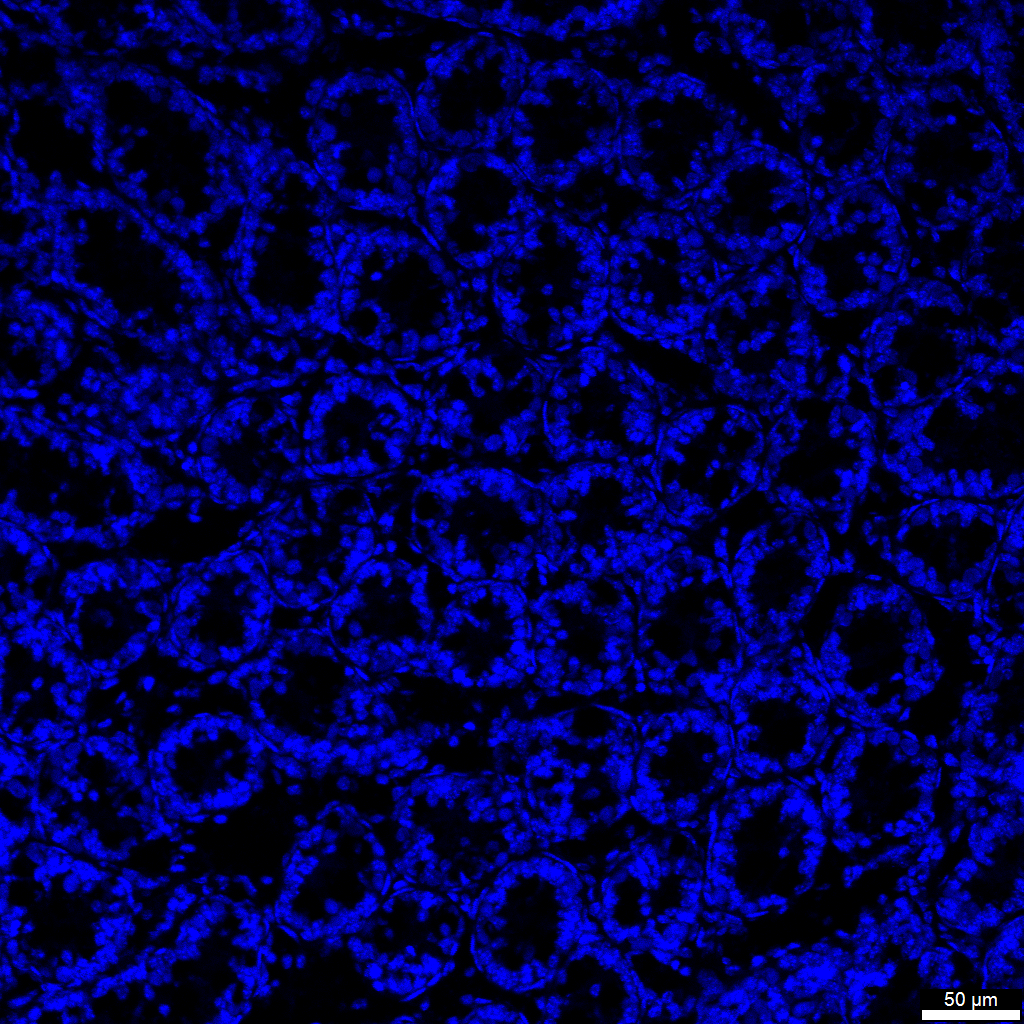

Supplement: Supplementary file 11 — Source data Fig. 8 [file 44319_2025_487_MOESM11_ESM.zip › Figure 8/8A/PD4 WT testis anti-PLZF&BrdU/PD4 WT testis anti-PLZF&BrdU Hoechst.tif]

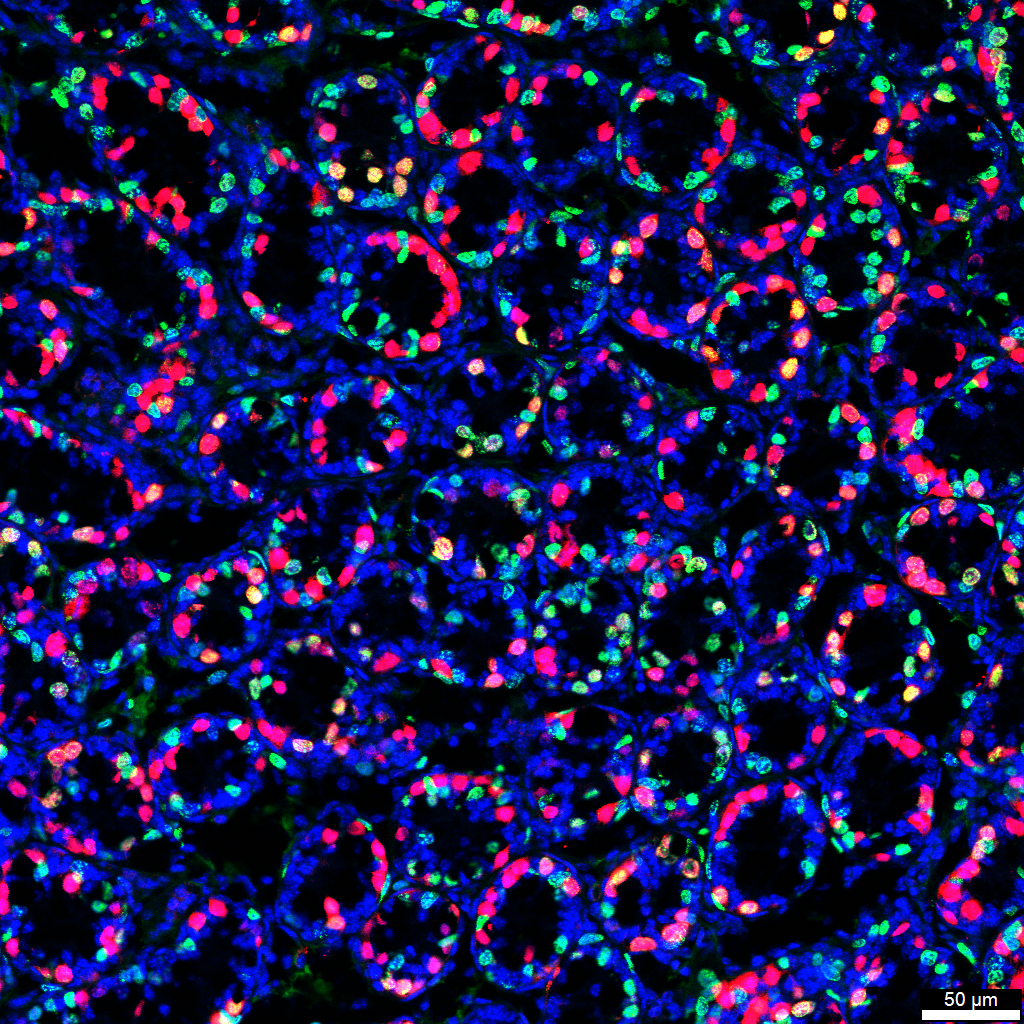

Supplement: Supplementary file 11 — Source data Fig. 8 [file 44319_2025_487_MOESM11_ESM.zip › Figure 8/8A/PD4 WT testis anti-PLZF&BrdU/PD4 WT testis anti-PLZF&BrdU Hoechst_overlay.tif]

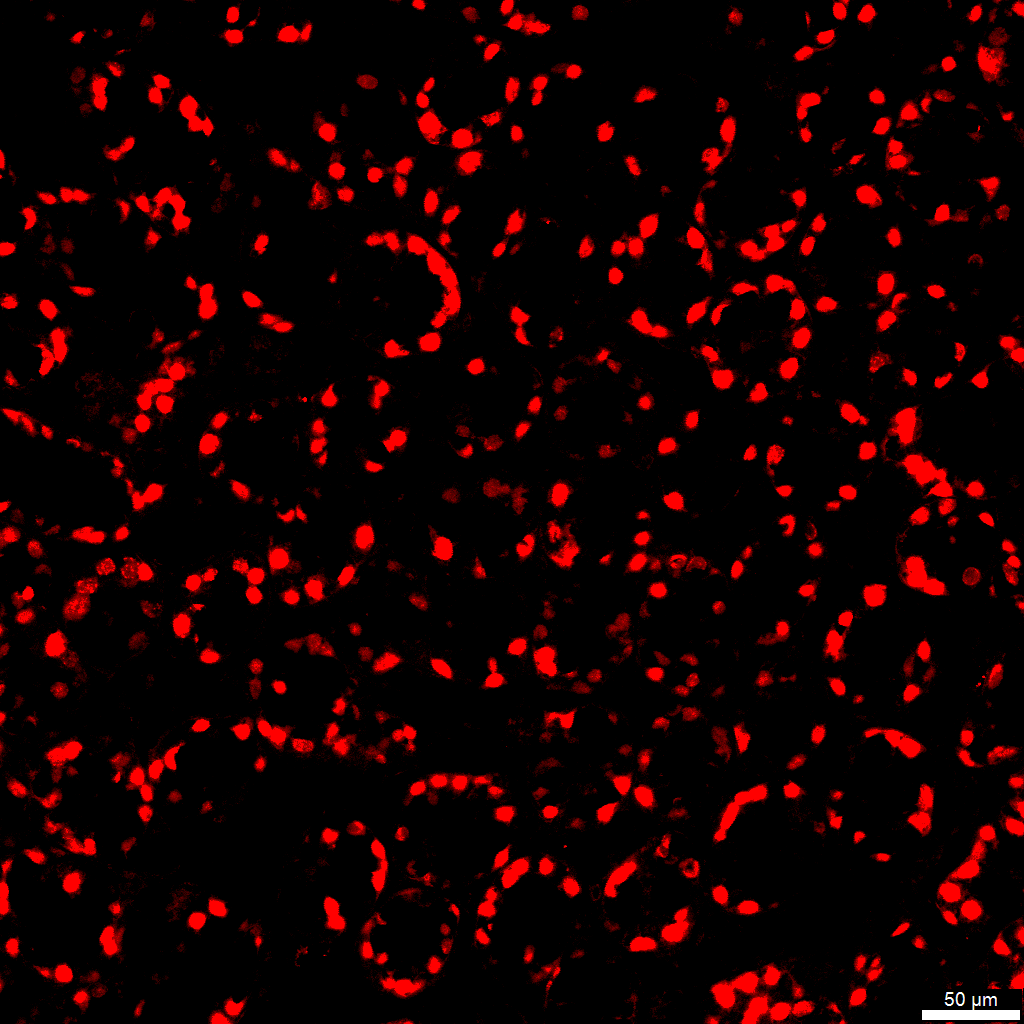

Supplement: Supplementary file 11 — Source data Fig. 8 [file 44319_2025_487_MOESM11_ESM.zip › Figure 8/8A/PD4 WT testis anti-PLZF&BrdU/PD4 WT testis anti-PLZF.tif]

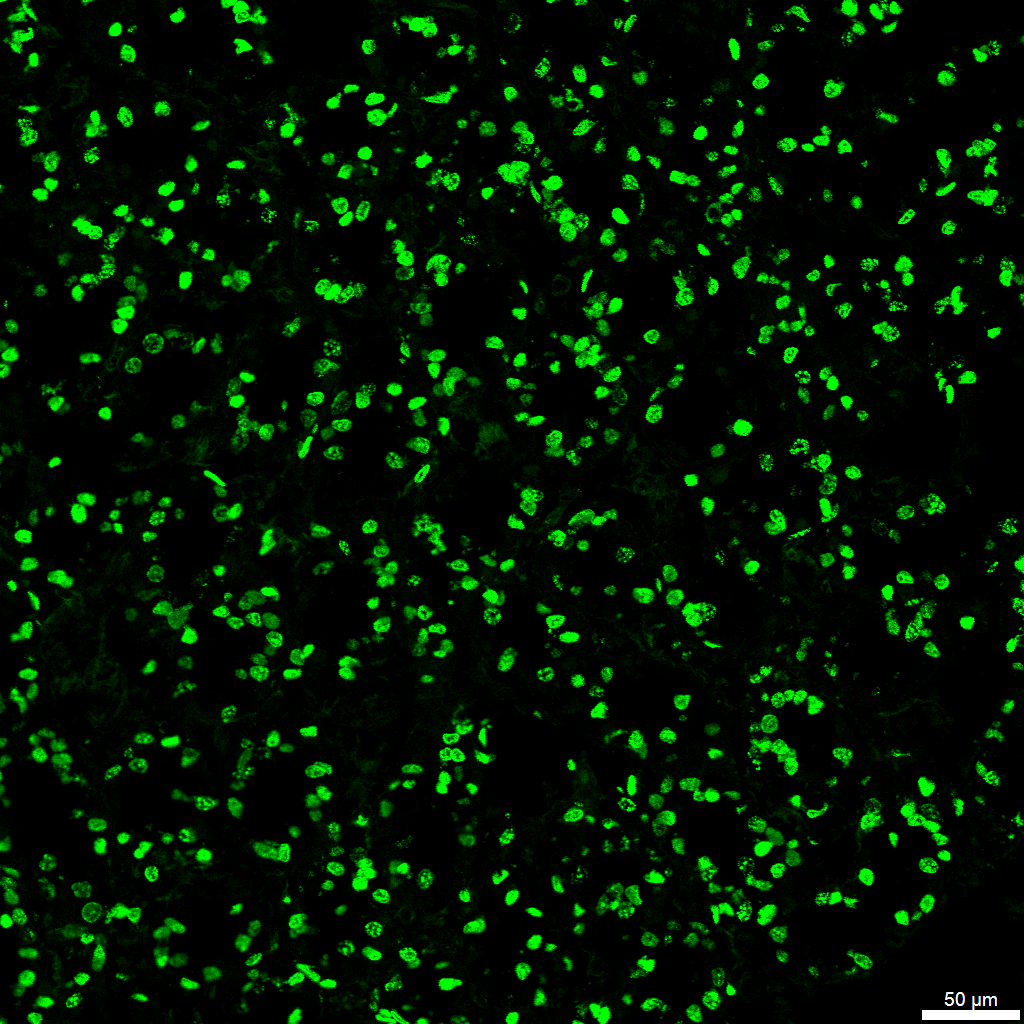

Supplement: Supplementary file 11 — Source data Fig. 8 [file 44319_2025_487_MOESM11_ESM.zip › Figure 8/8A/PD7 WT testis anti-PLZF&BrdU/PD7 WT testis anti-BrdU.tif]

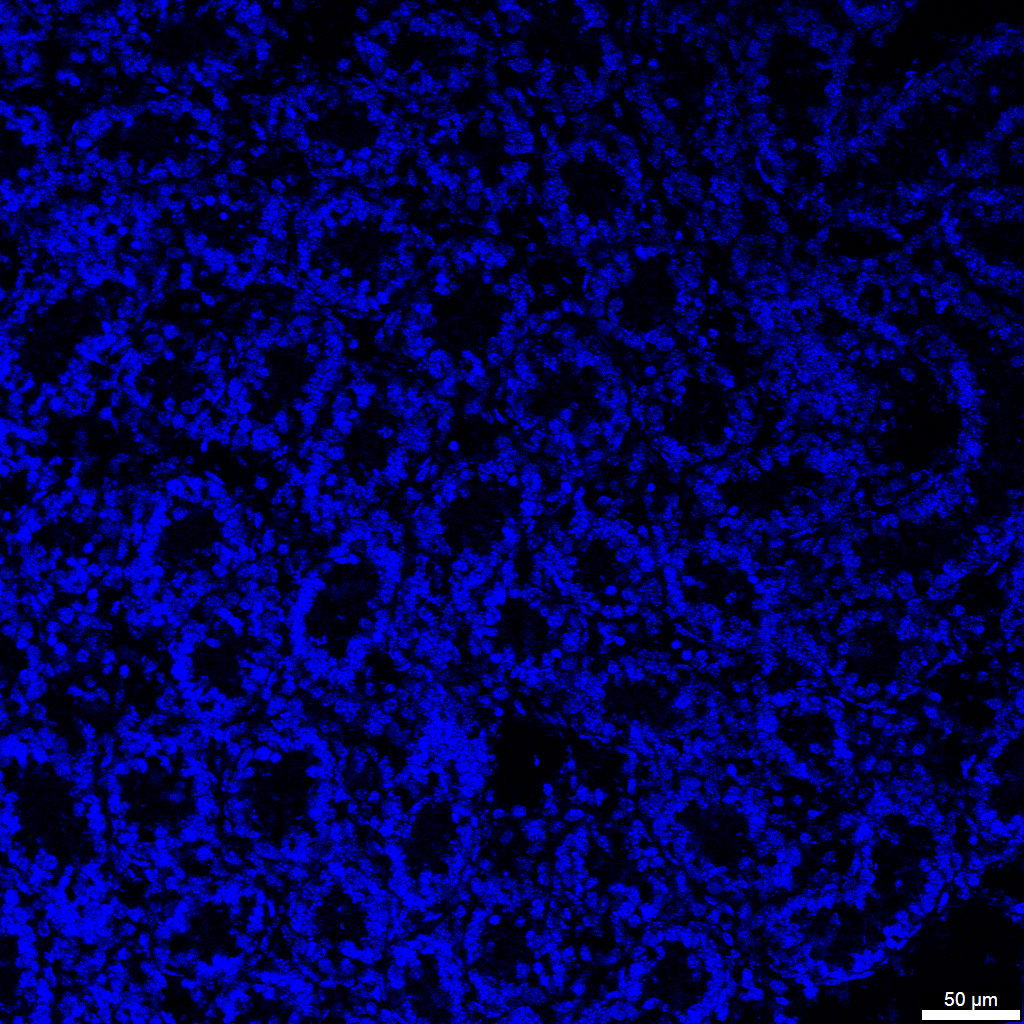

Supplement: Supplementary file 11 — Source data Fig. 8 [file 44319_2025_487_MOESM11_ESM.zip › Figure 8/8A/PD7 WT testis anti-PLZF&BrdU/PD7 WT testis anti-PLZF&BrdU Hoechst.tif]

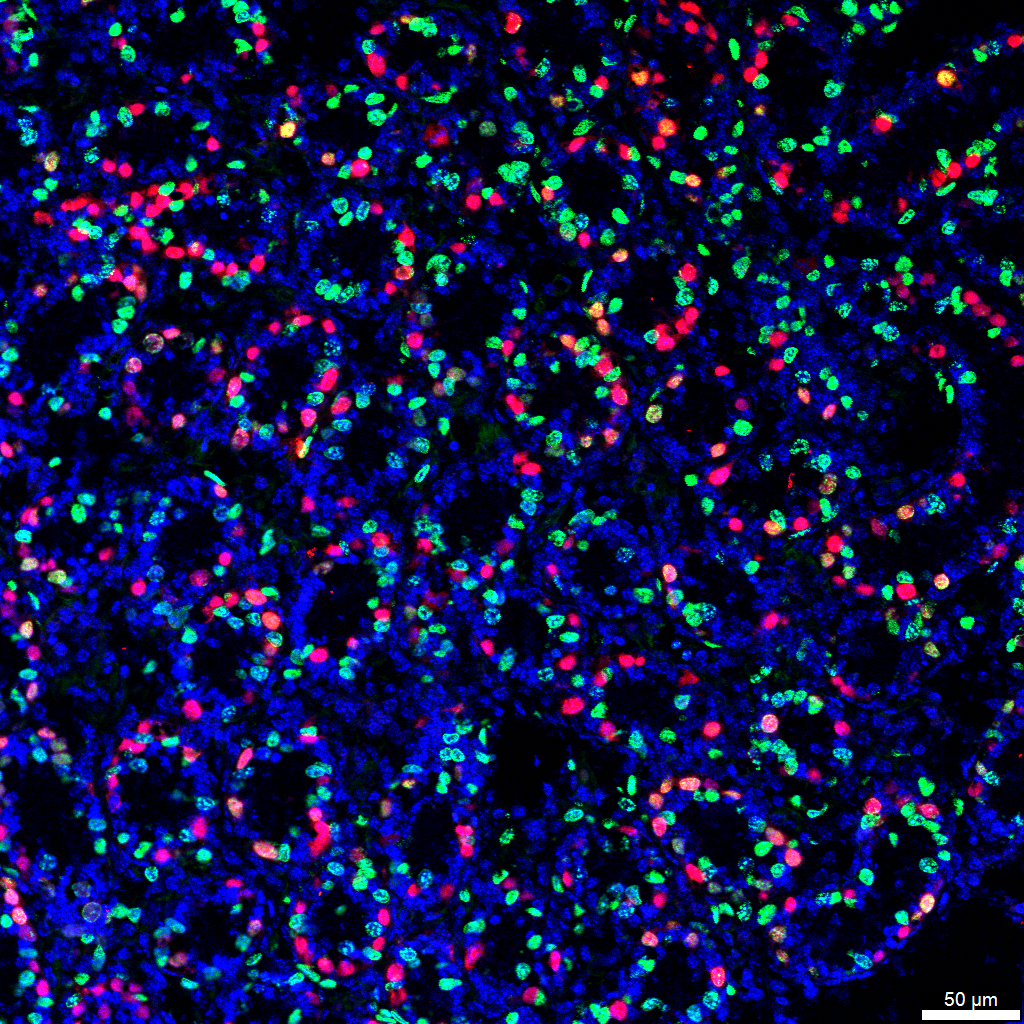

Supplement: Supplementary file 11 — Source data Fig. 8 [file 44319_2025_487_MOESM11_ESM.zip › Figure 8/8A/PD7 WT testis anti-PLZF&BrdU/PD7 WT testis anti-PLZF&BrdU Hoechst_overlay.tif]

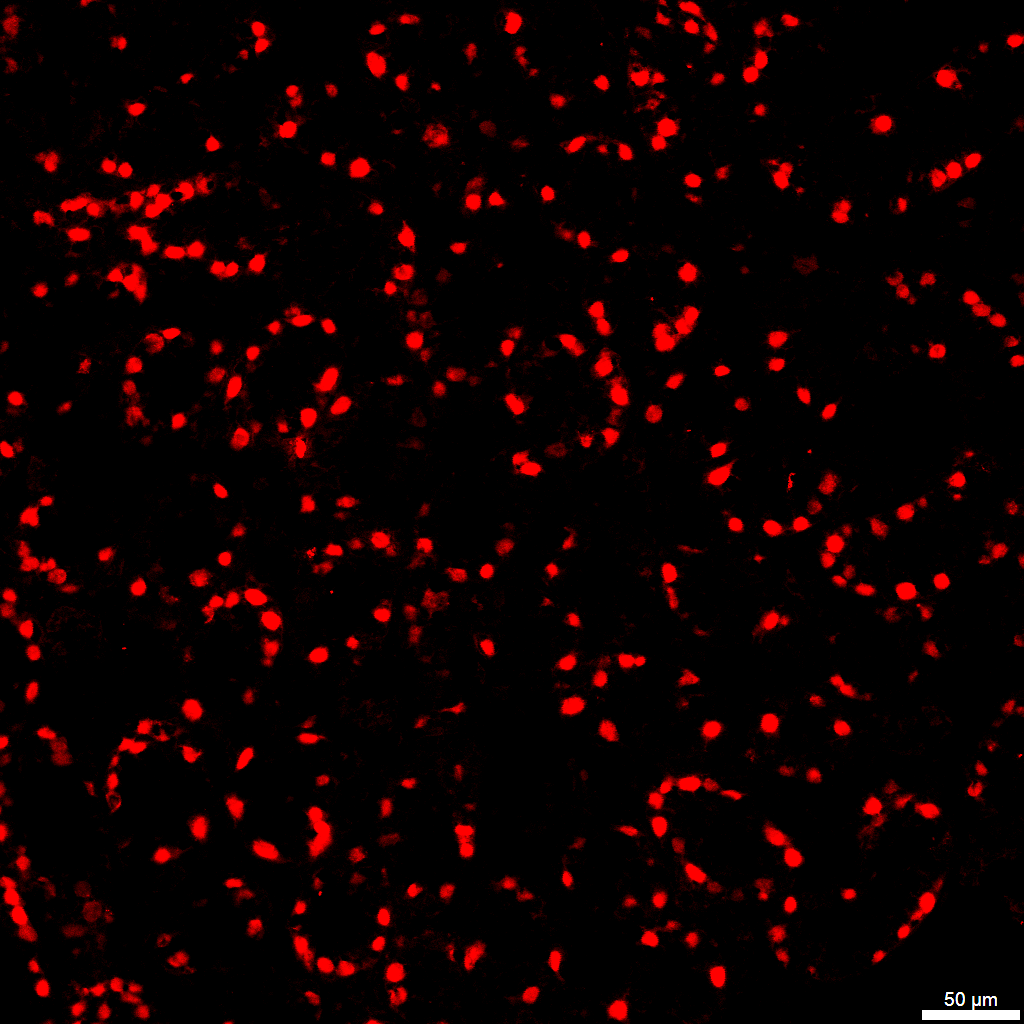

Supplement: Supplementary file 11 — Source data Fig. 8 [file 44319_2025_487_MOESM11_ESM.zip › Figure 8/8A/PD7 WT testis anti-PLZF&BrdU/PD7 WT testis anti-PLZF.tif]

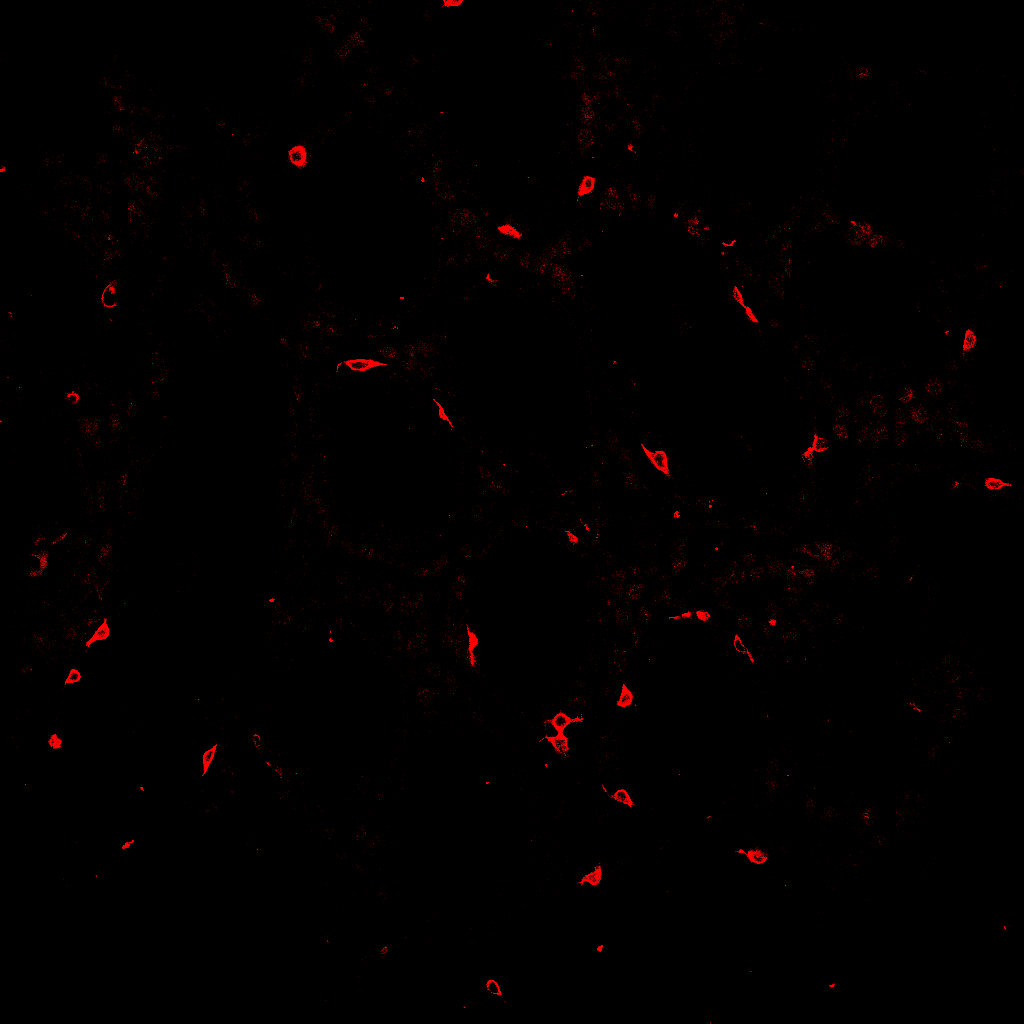

Supplement: Supplementary file 11 — Source data Fig. 8 [file 44319_2025_487_MOESM11_ESM.zip › Figure 8/8C/PD21 Brca1 vKO testis +NAC for three weeks anti-PLZF&GFRα1/PD21 Brca1 vKO testis +NAC anti-GFRα1.tif]

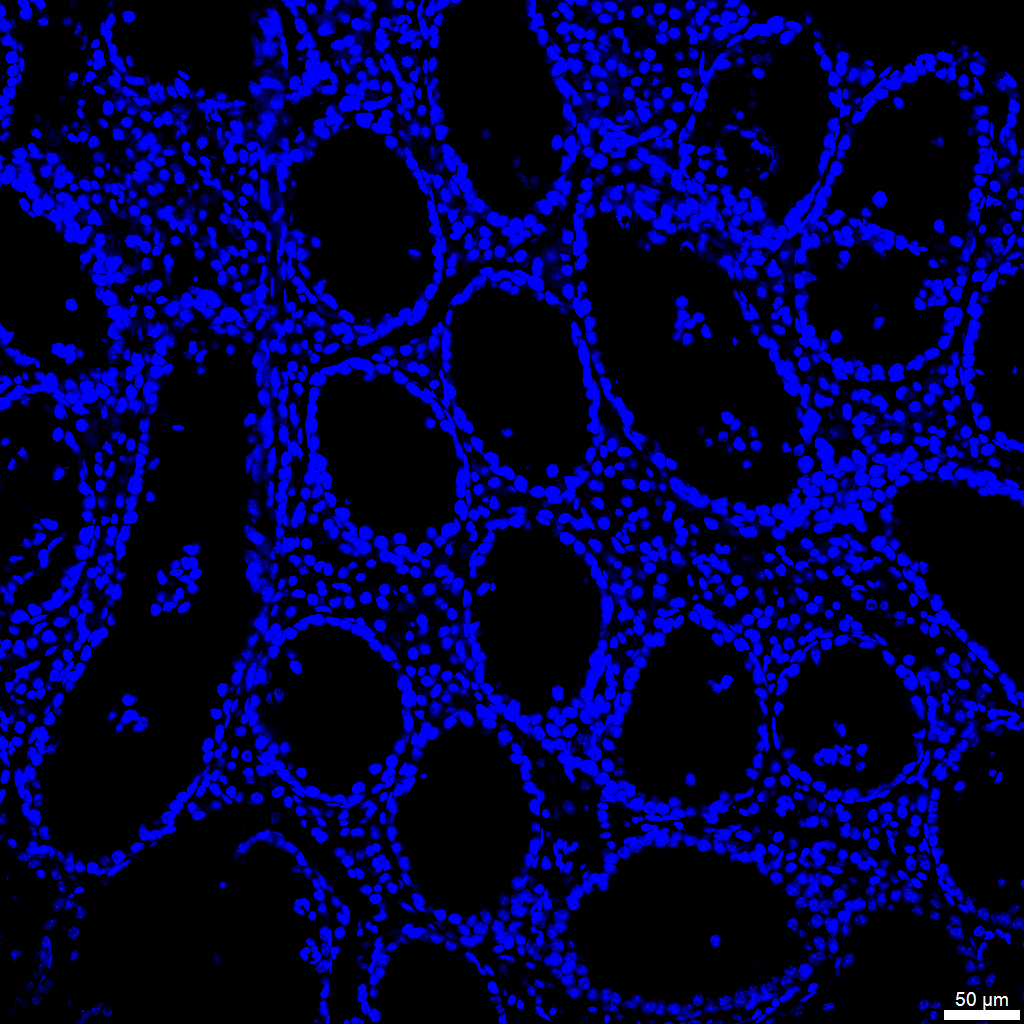

Supplement: Supplementary file 11 — Source data Fig. 8 [file 44319_2025_487_MOESM11_ESM.zip › Figure 8/8C/PD21 Brca1 vKO testis +NAC for three weeks anti-PLZF&GFRα1/PD21 Brca1 vKO testis +NAC anti-PLZF&GFRα1 Hoechst.tif]

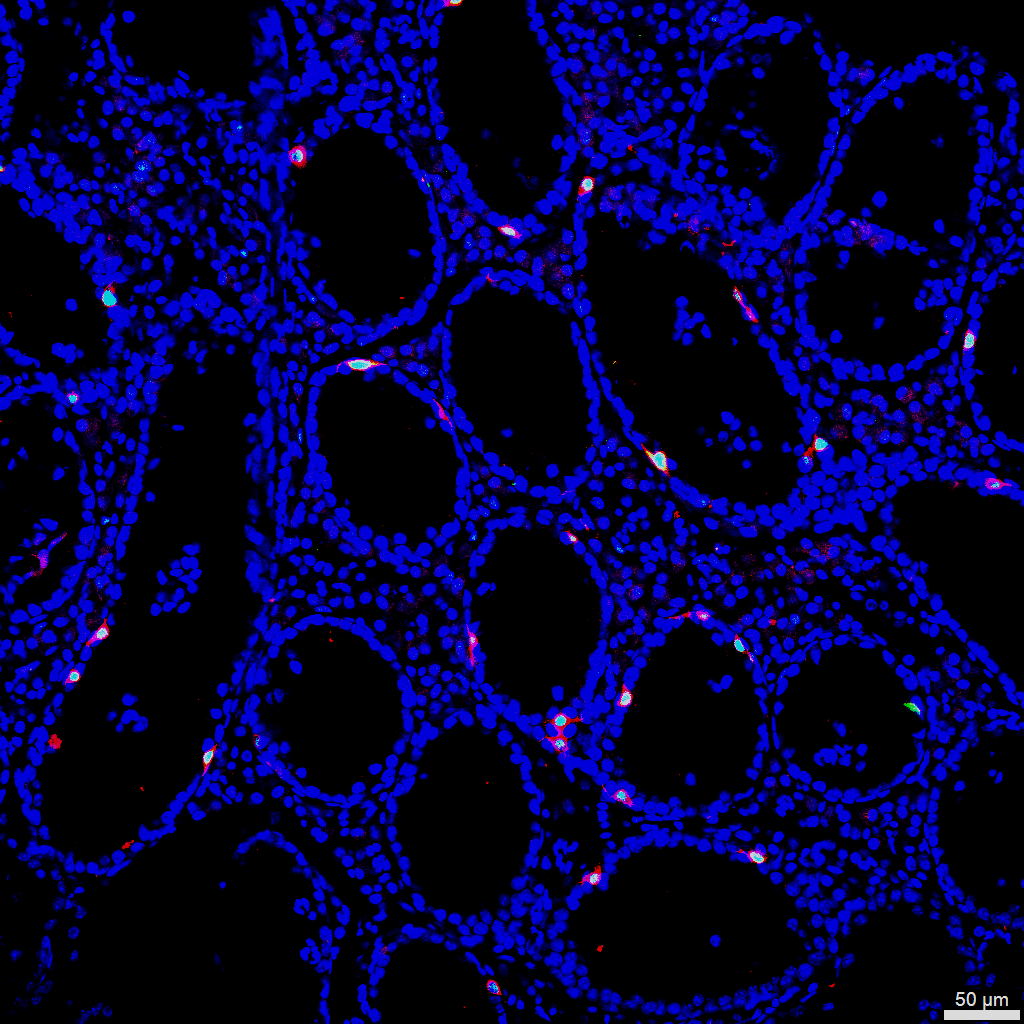

Supplement: Supplementary file 11 — Source data Fig. 8 [file 44319_2025_487_MOESM11_ESM.zip › Figure 8/8C/PD21 Brca1 vKO testis +NAC for three weeks anti-PLZF&GFRα1/PD21 Brca1 vKO testis +NAC anti-PLZF&GFRα1 Hoechst_overlay.tif]

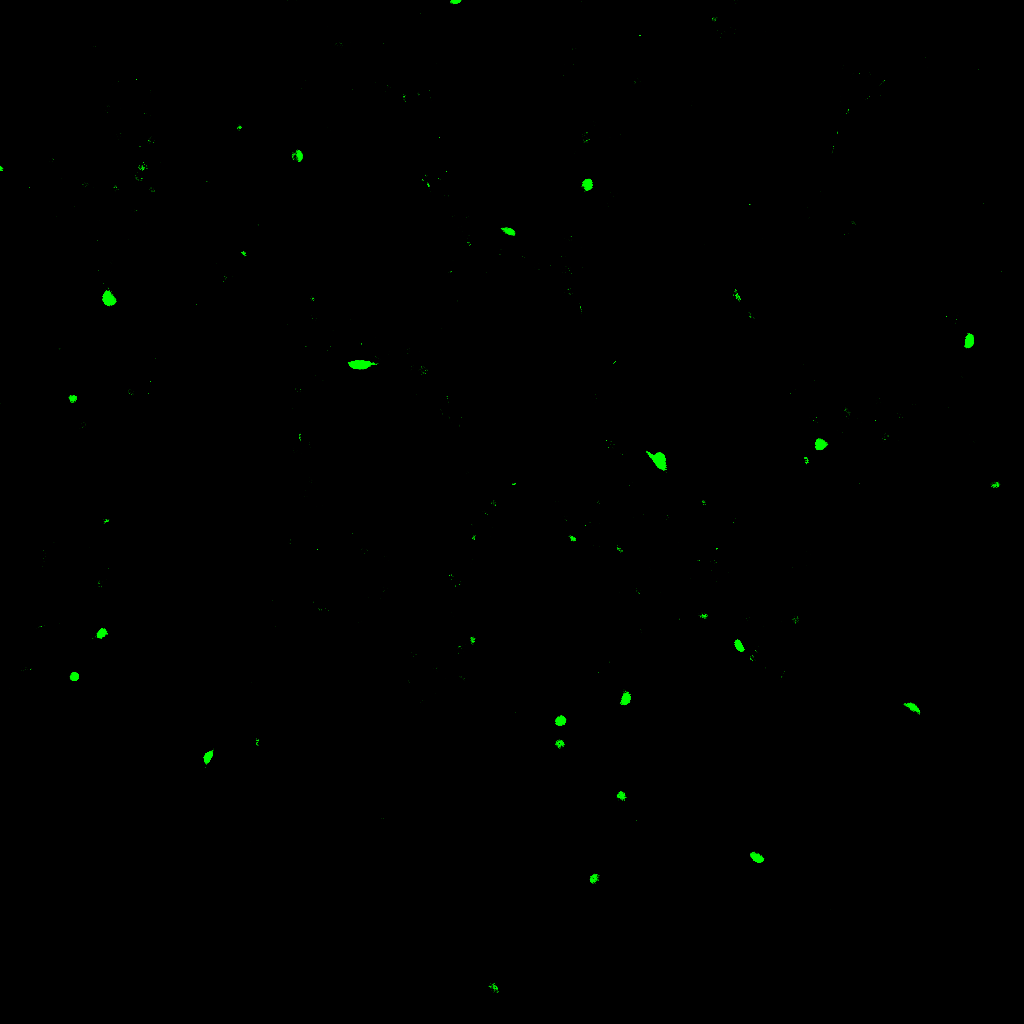

Supplement: Supplementary file 11 — Source data Fig. 8 [file 44319_2025_487_MOESM11_ESM.zip › Figure 8/8C/PD21 Brca1 vKO testis +NAC for three weeks anti-PLZF&GFRα1/PD21 Brca1 vKO testis +NAC anti-PLZF.tif]

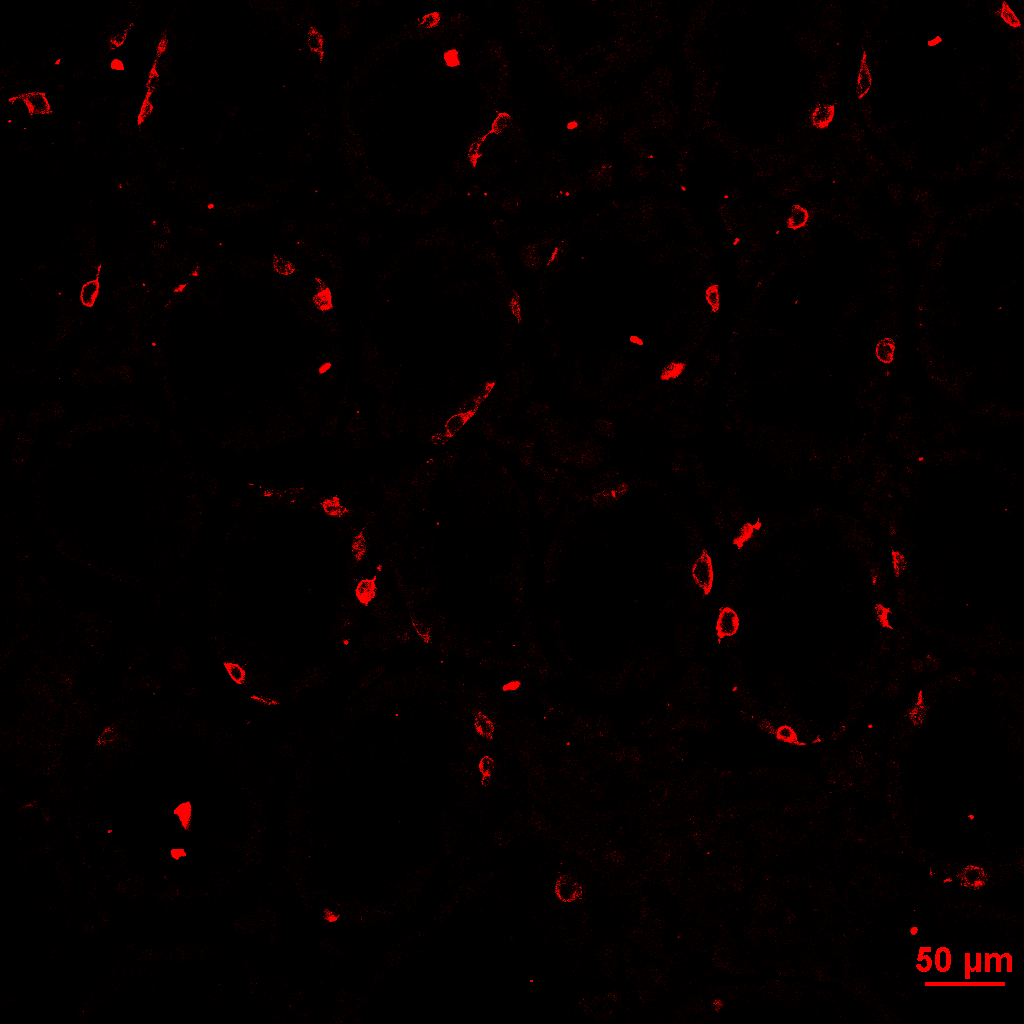

Supplement: Supplementary file 11 — Source data Fig. 8 [file 44319_2025_487_MOESM11_ESM.zip › Figure 8/8C/PD21 Brca1 vKO testis -NAC for three weeks anti-PLZF&GFRα1/PD21 Brca1 vKO testis -NAC anti-GFRα1.tif]

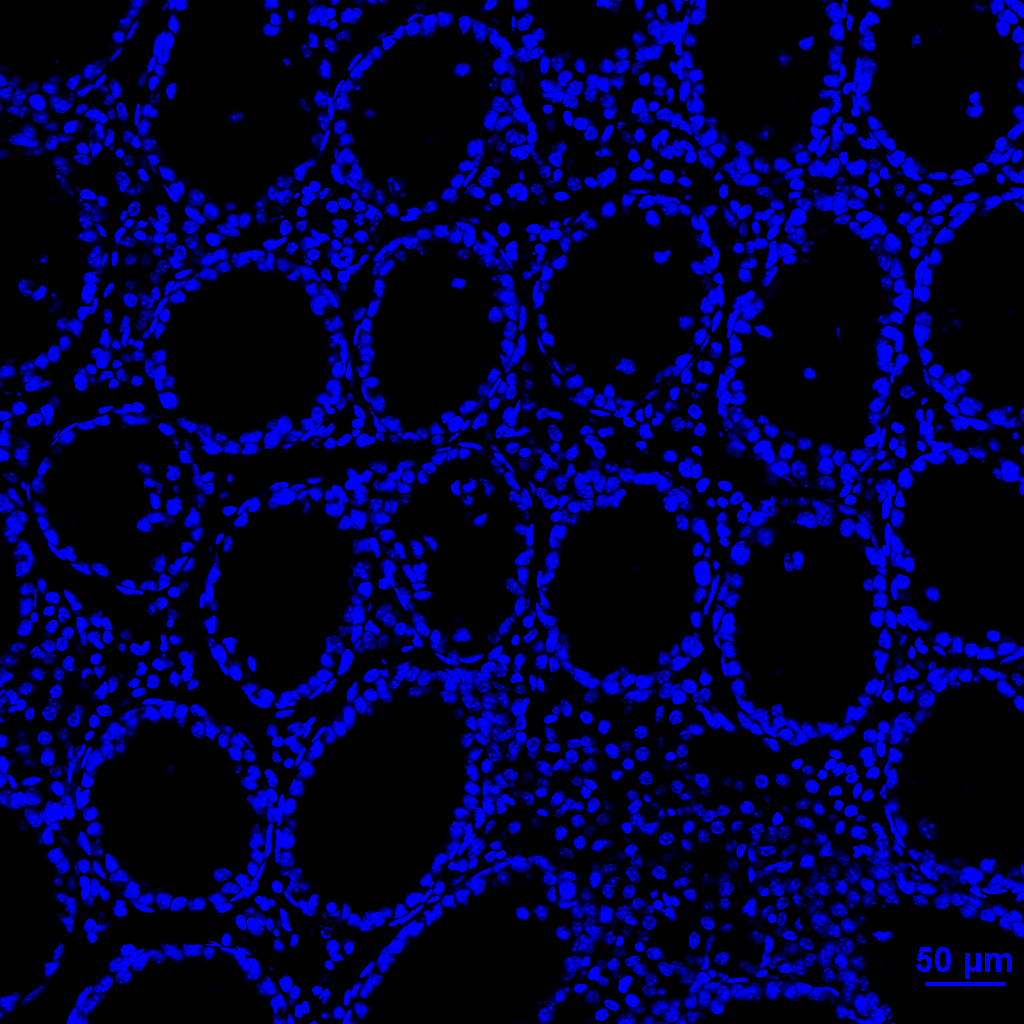

Supplement: Supplementary file 11 — Source data Fig. 8 [file 44319_2025_487_MOESM11_ESM.zip › Figure 8/8C/PD21 Brca1 vKO testis -NAC for three weeks anti-PLZF&GFRα1/PD21 Brca1 vKO testis -NAC anti-PLZF&GFRα1 Hoechst.tif]

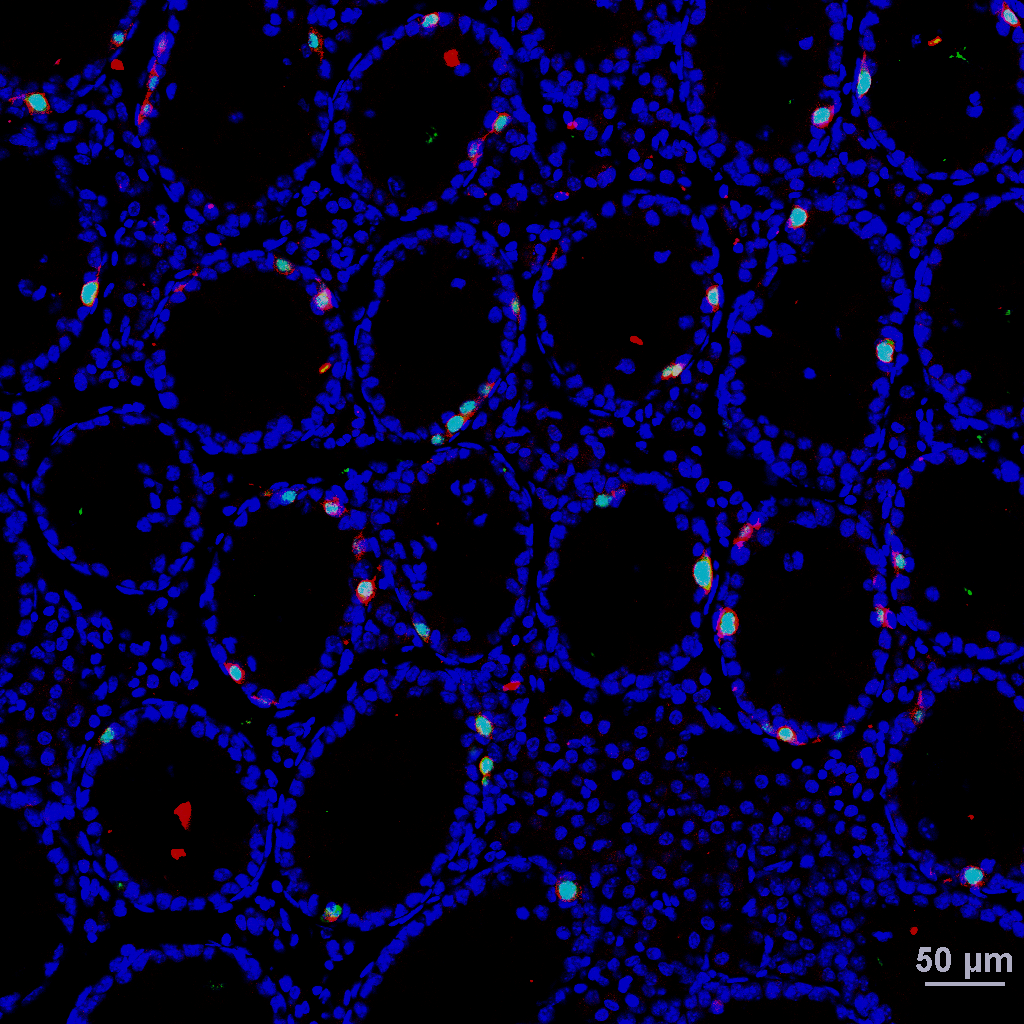

Supplement: Supplementary file 11 — Source data Fig. 8 [file 44319_2025_487_MOESM11_ESM.zip › Figure 8/8C/PD21 Brca1 vKO testis -NAC for three weeks anti-PLZF&GFRα1/PD21 Brca1 vKO testis -NAC anti-PLZF&GFRα1 Hoechst_overlay.tif]

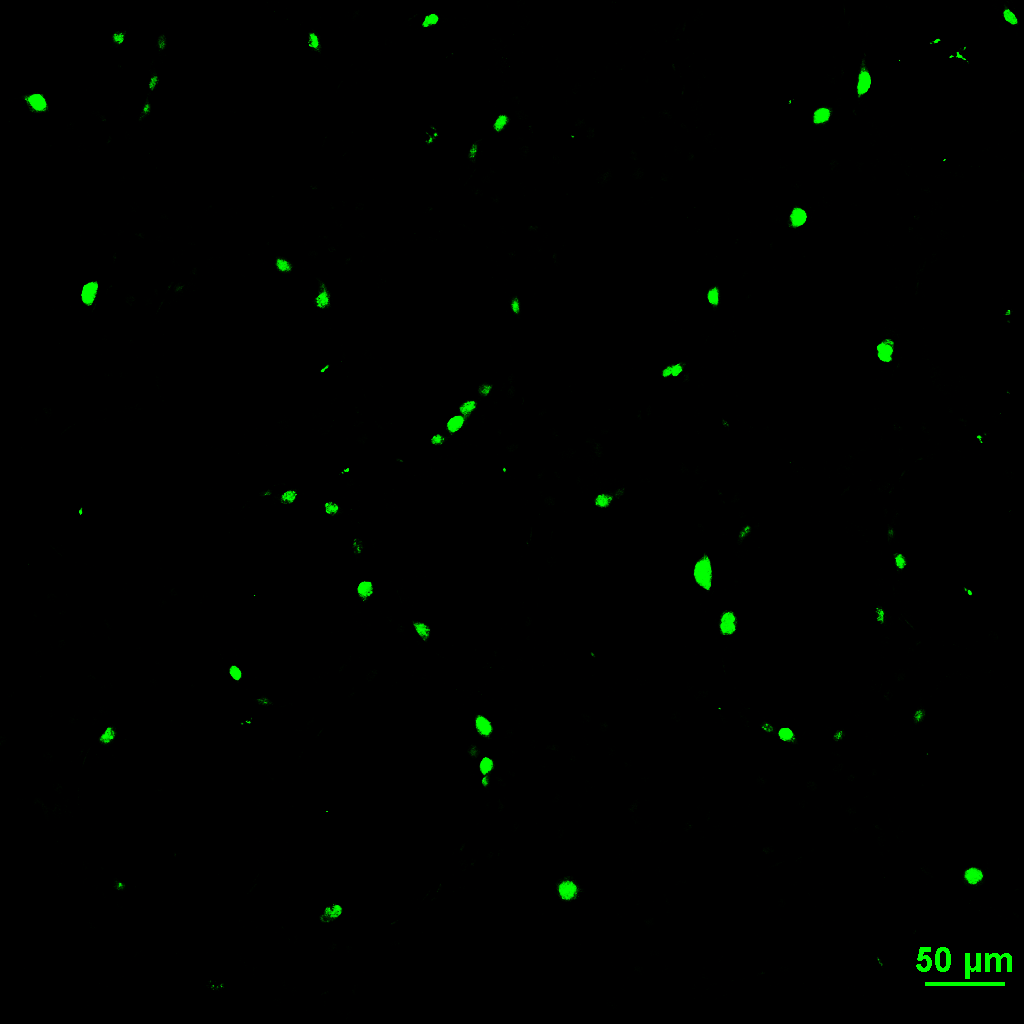

Supplement: Supplementary file 11 — Source data Fig. 8 [file 44319_2025_487_MOESM11_ESM.zip › Figure 8/8C/PD21 Brca1 vKO testis -NAC for three weeks anti-PLZF&GFRα1/PD21 Brca1 vKO testis -NAC anti-PLZF.tif]

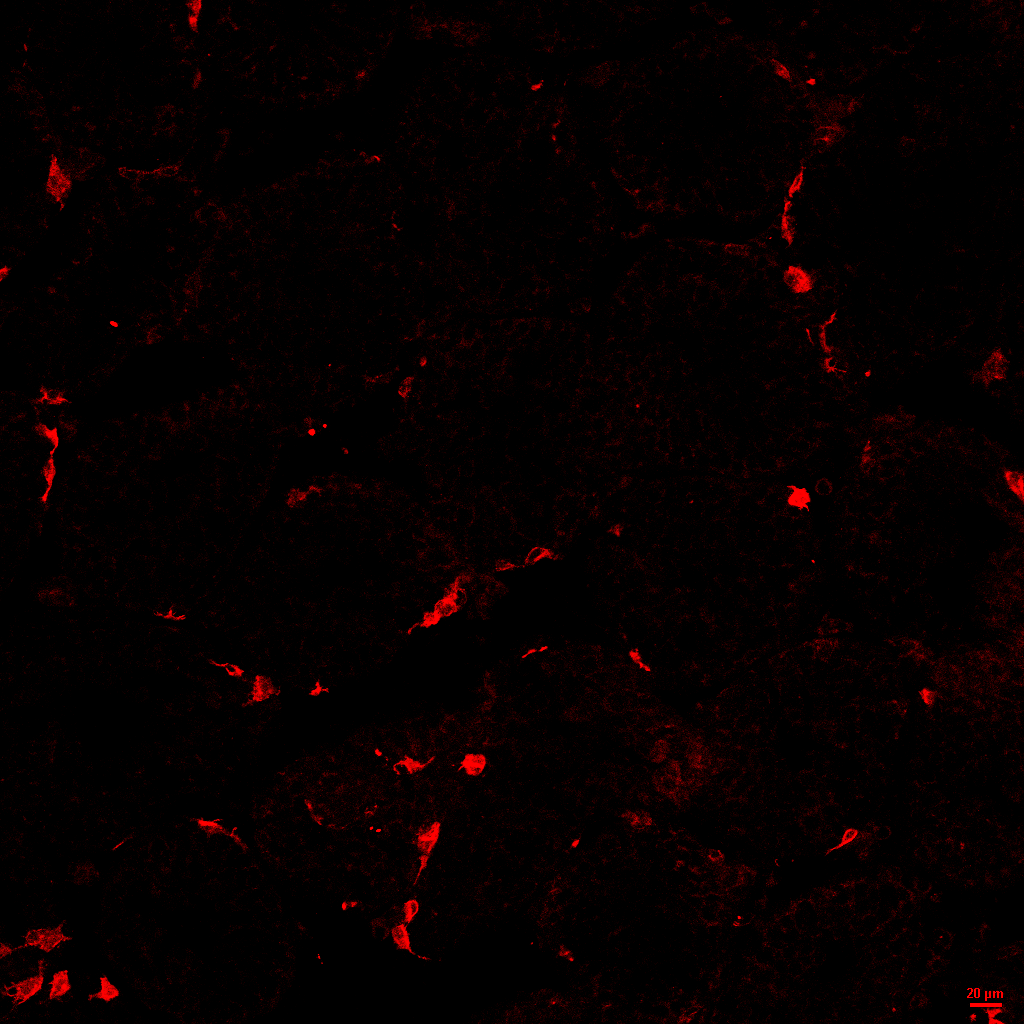

Supplement: Supplementary file 11 — Source data Fig. 8 [file 44319_2025_487_MOESM11_ESM.zip › Figure 8/8E/PD21 Aldh2 KO PLZF&GFRa1/PD21 Aldh2 KO testis anti-GFRa1.tif]

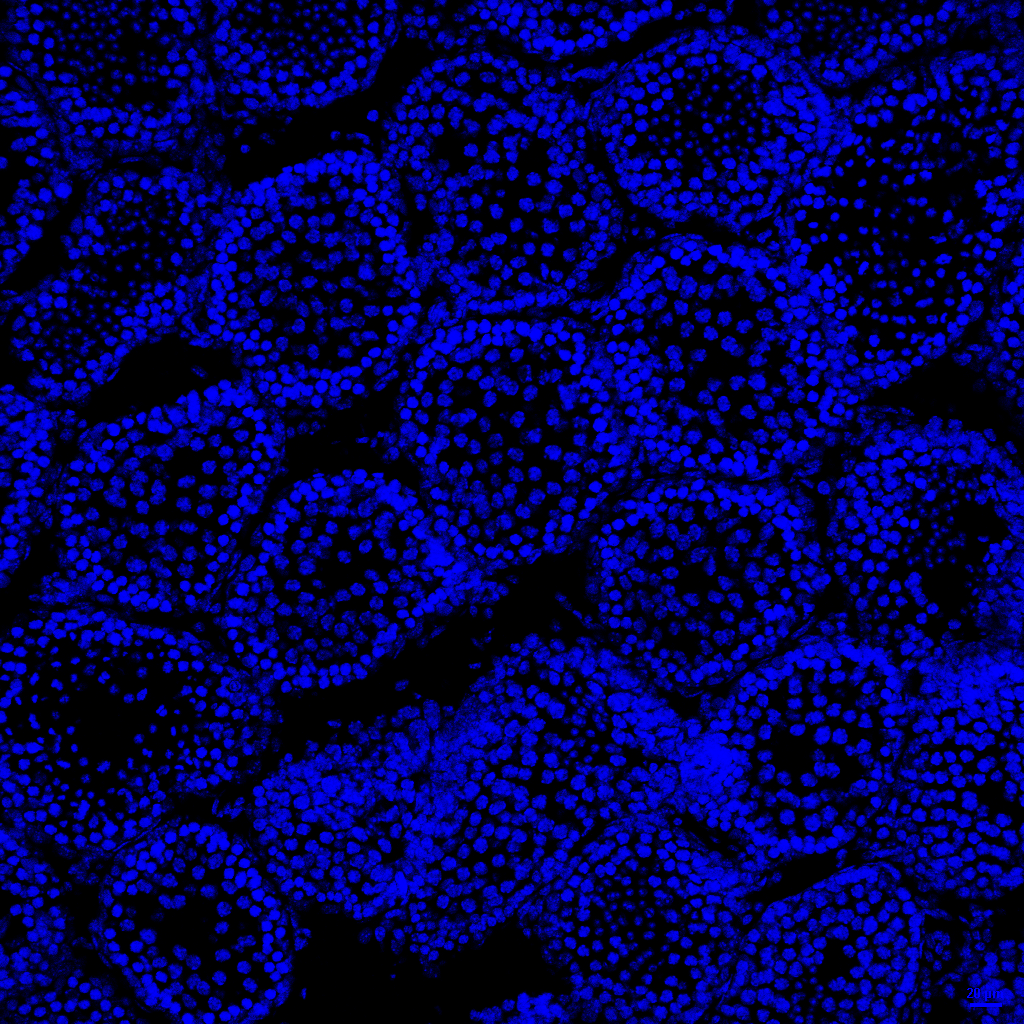

Supplement: Supplementary file 11 — Source data Fig. 8 [file 44319_2025_487_MOESM11_ESM.zip › Figure 8/8E/PD21 Aldh2 KO PLZF&GFRa1/PD21 Aldh2 KO testis anti-PLZF&GFRa1 Hoechst.tif]

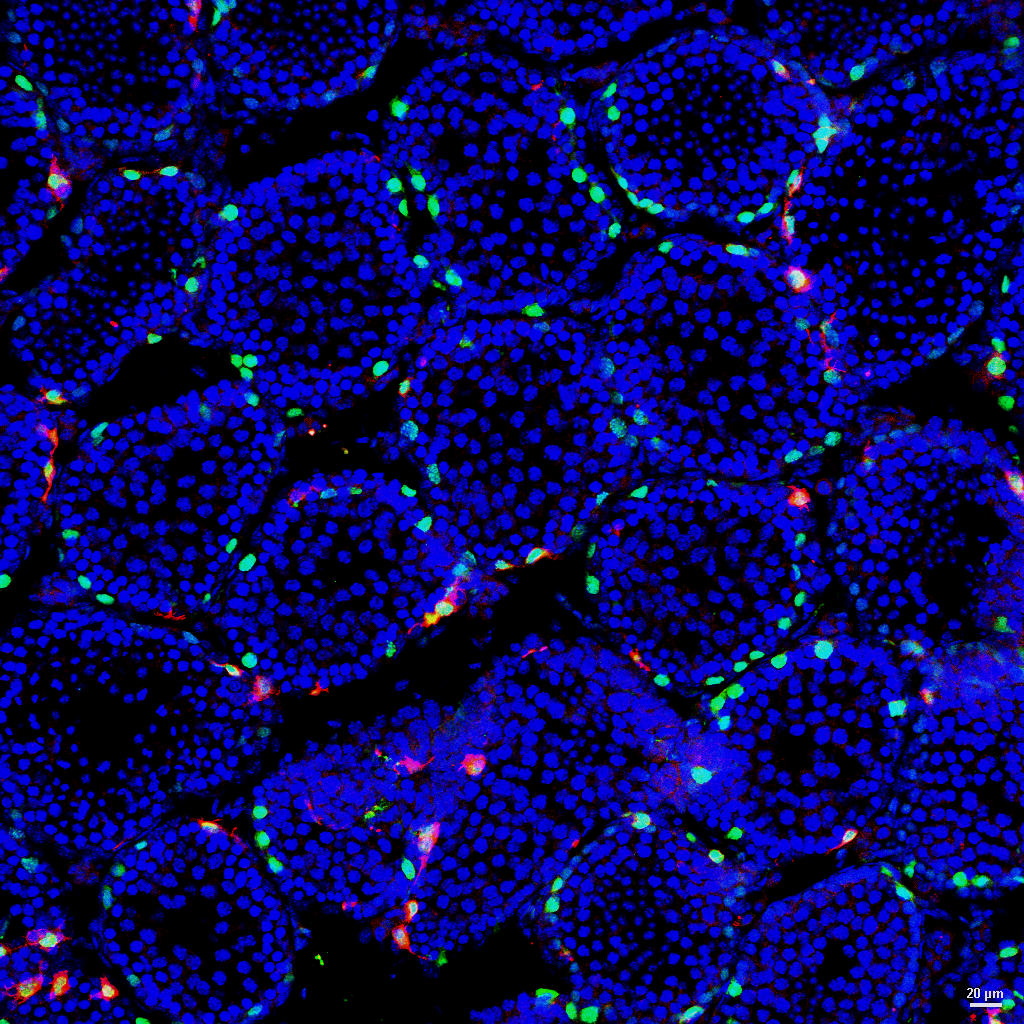

Supplement: Supplementary file 11 — Source data Fig. 8 [file 44319_2025_487_MOESM11_ESM.zip › Figure 8/8E/PD21 Aldh2 KO PLZF&GFRa1/PD21 Aldh2 KO testis anti-PLZF&GFRa1 Hoechst_overlay.tif]

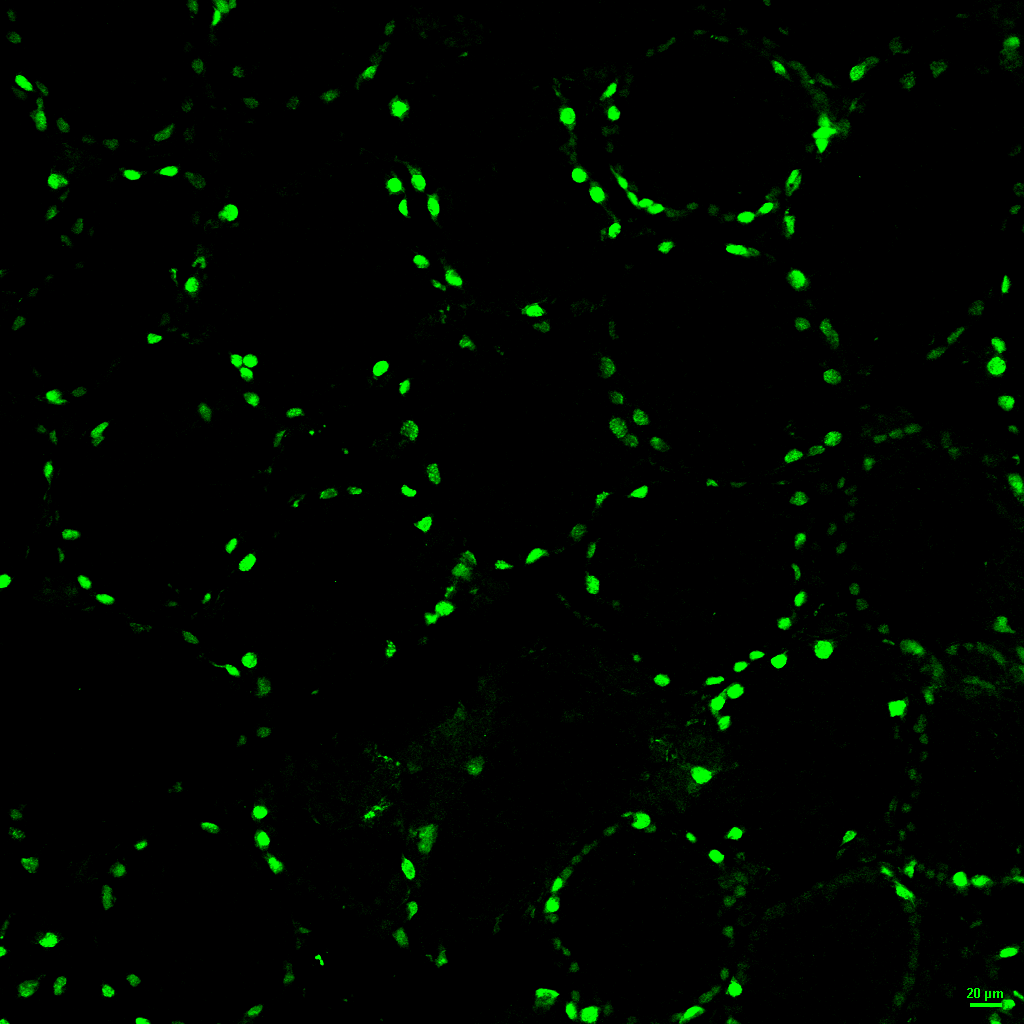

Supplement: Supplementary file 11 — Source data Fig. 8 [file 44319_2025_487_MOESM11_ESM.zip › Figure 8/8E/PD21 Aldh2 KO PLZF&GFRa1/PD21 Aldh2 KO testis anti-PLZF.tif]

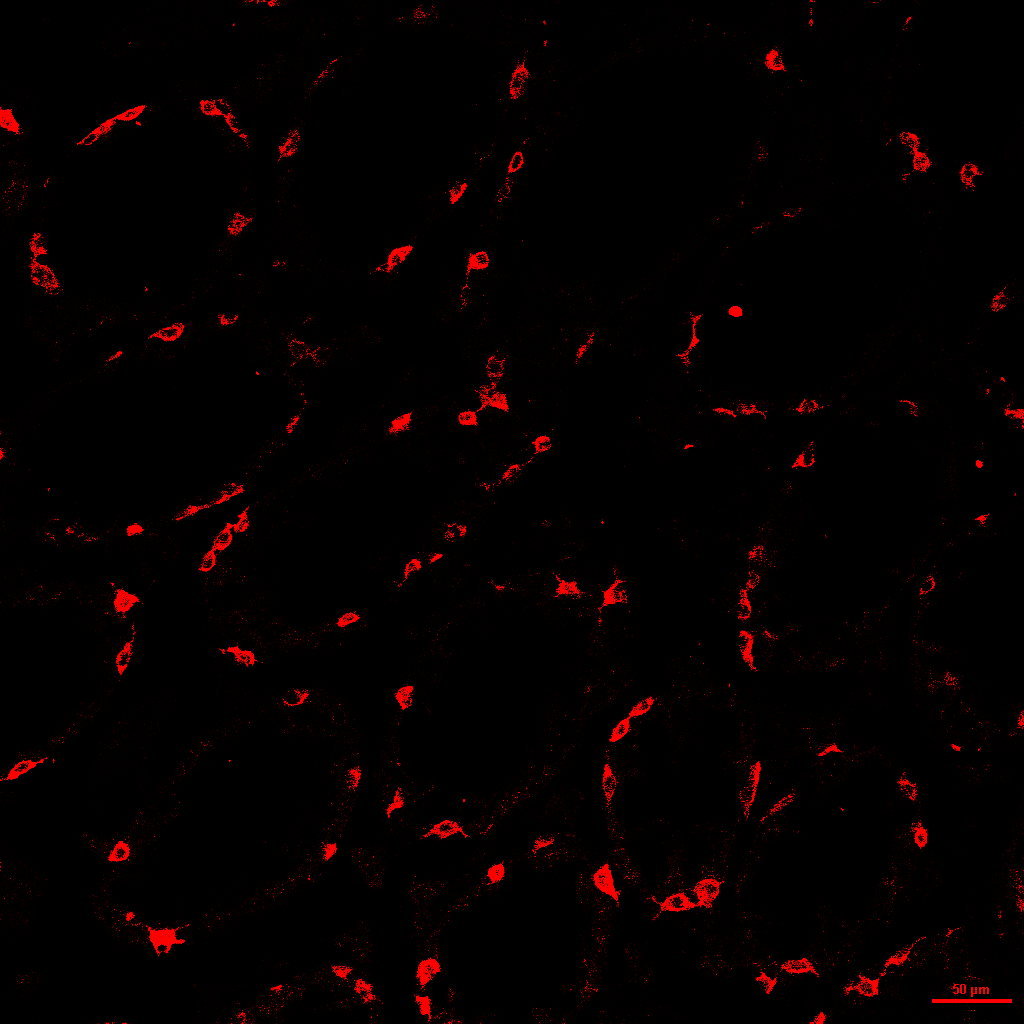

Supplement: Supplementary file 11 — Source data Fig. 8 [file 44319_2025_487_MOESM11_ESM.zip › Figure 8/8E/PD21 Brca1 vKO PLZF&GFRa1/PD21 BRCA1 vKO testis anti-GFRa1.tif]

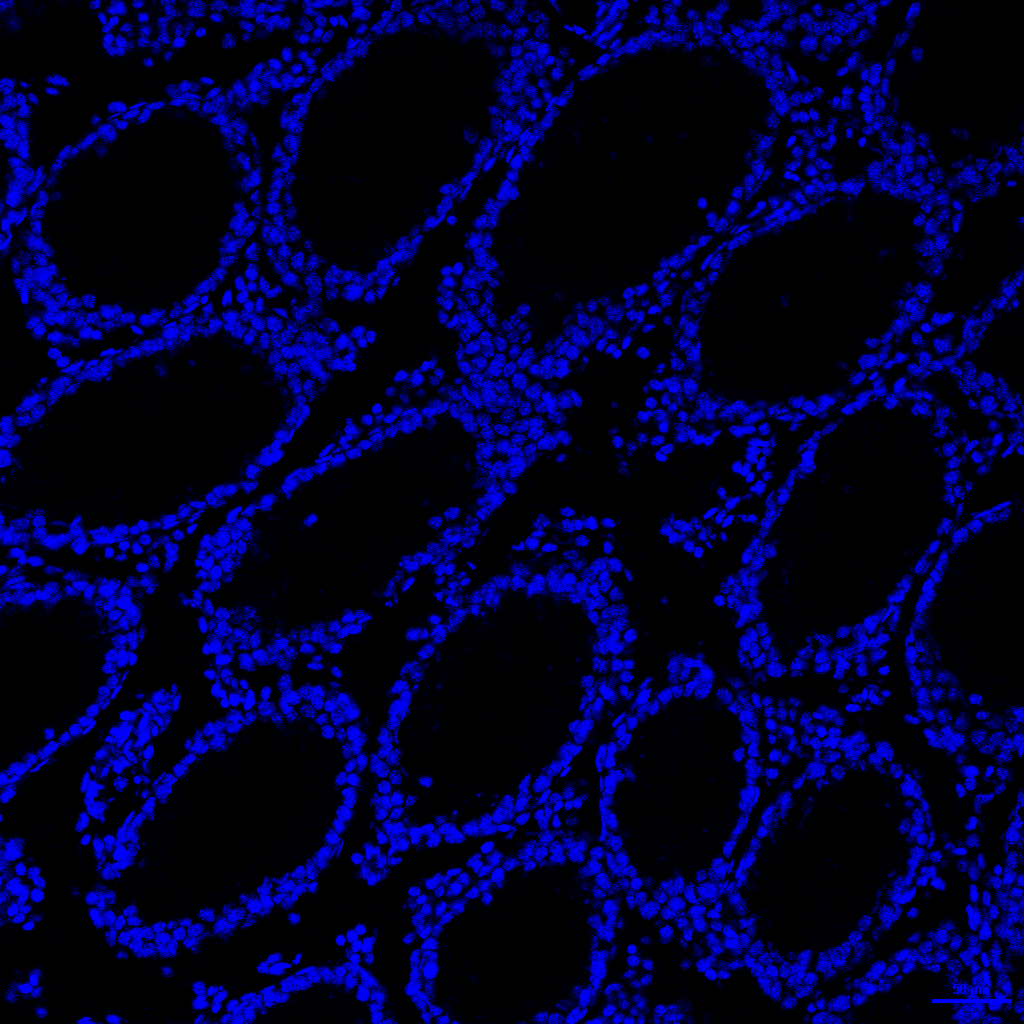

Supplement: Supplementary file 11 — Source data Fig. 8 [file 44319_2025_487_MOESM11_ESM.zip › Figure 8/8E/PD21 Brca1 vKO PLZF&GFRa1/PD21 BRCA1 vKO testis anti-PLZF&GFRa1 Hoechst.tif]

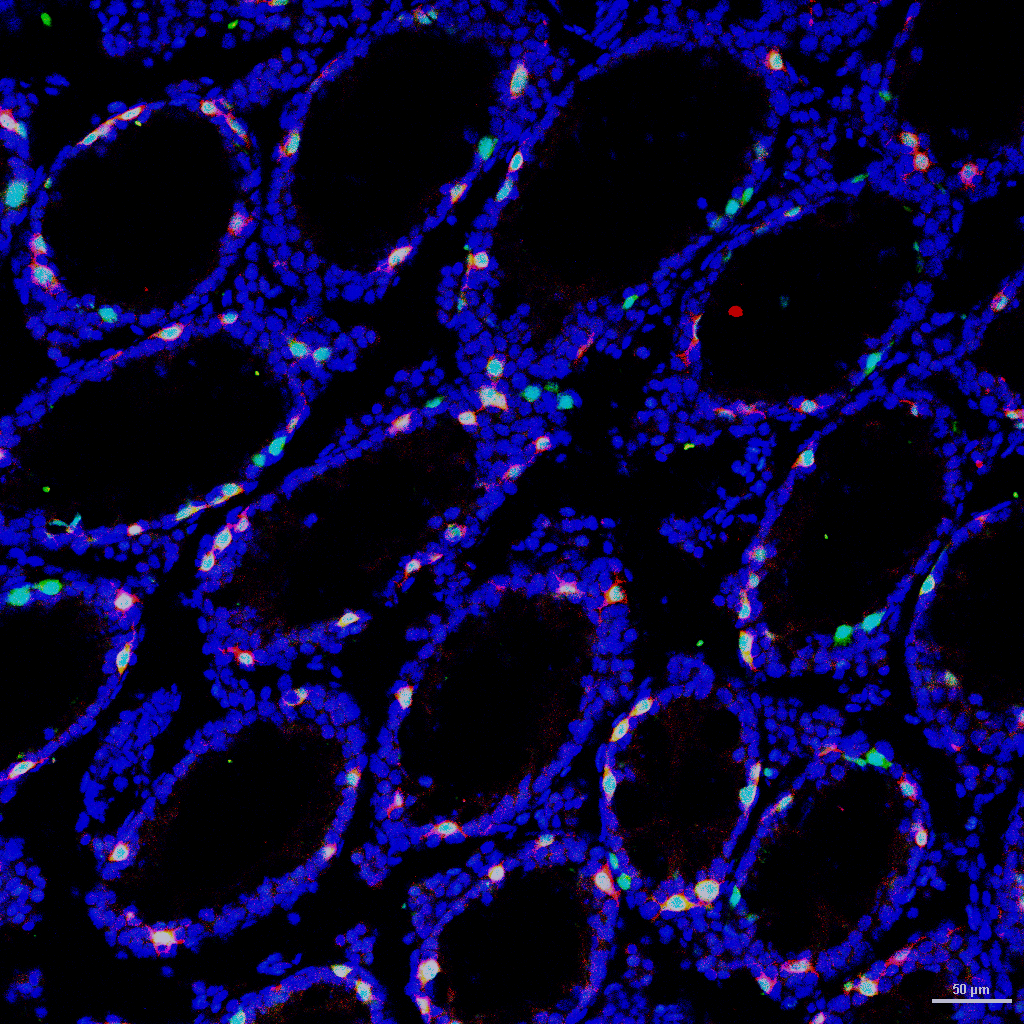

Supplement: Supplementary file 11 — Source data Fig. 8 [file 44319_2025_487_MOESM11_ESM.zip › Figure 8/8E/PD21 Brca1 vKO PLZF&GFRa1/PD21 BRCA1 vKO testis anti-PLZF&GFRa1 Hoechst_overlay.tif]

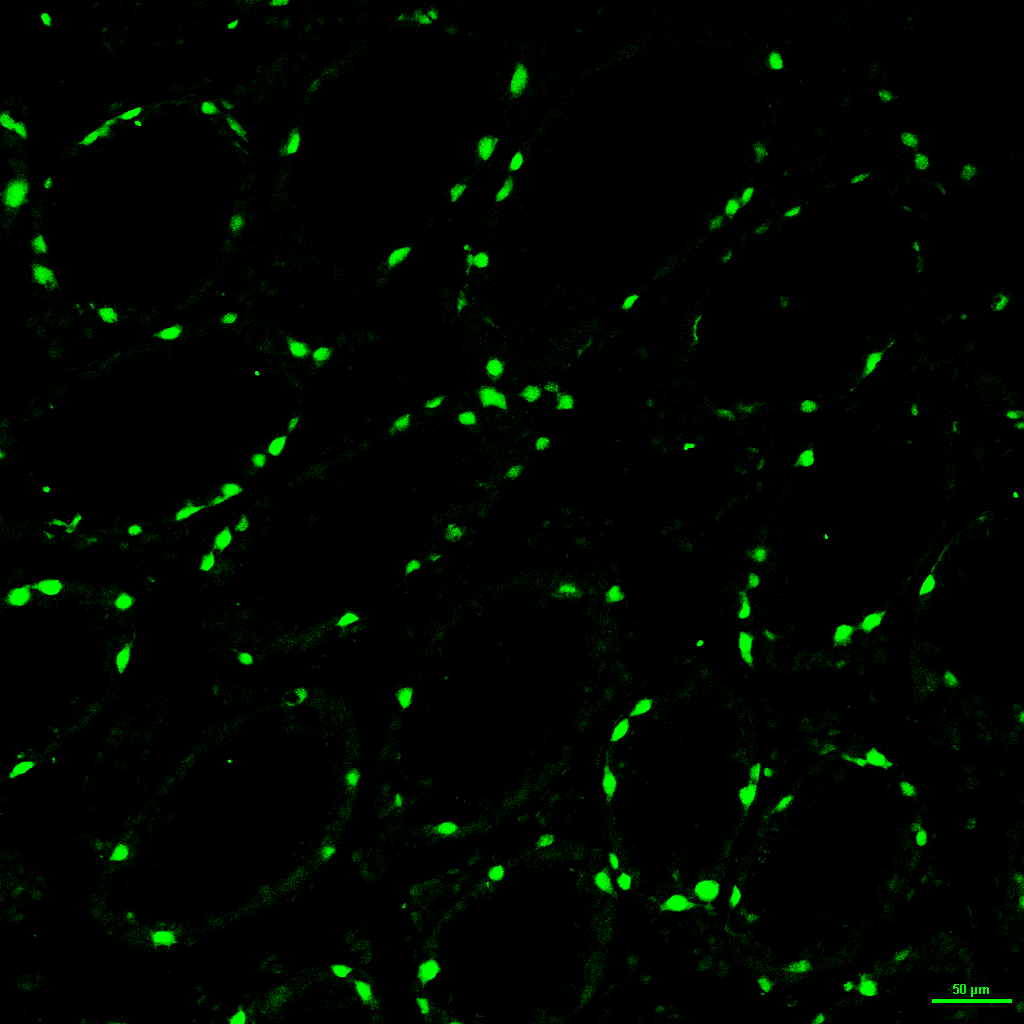

Supplement: Supplementary file 11 — Source data Fig. 8 [file 44319_2025_487_MOESM11_ESM.zip › Figure 8/8E/PD21 Brca1 vKO PLZF&GFRa1/PD21 BRCA1 vKO testis anti-PLZF.tif]

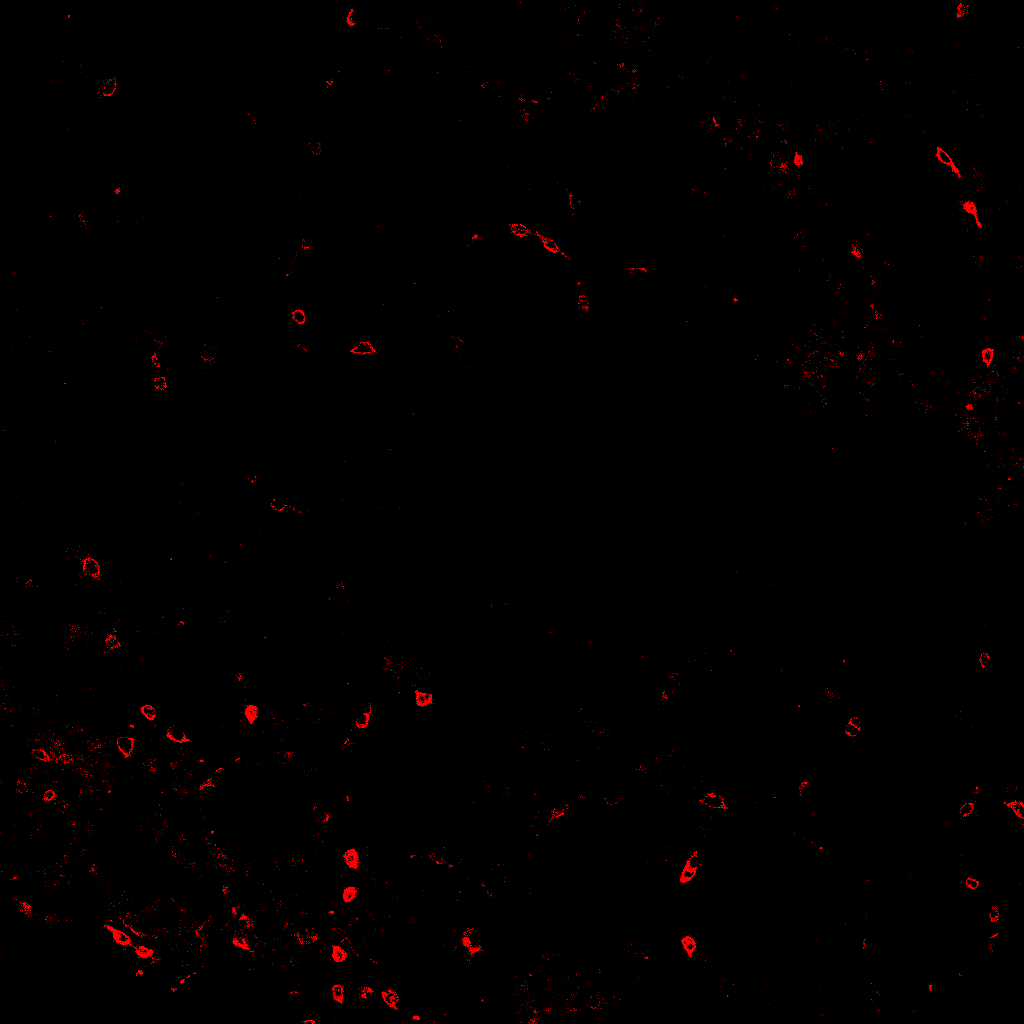

Supplement: Supplementary file 11 — Source data Fig. 8 [file 44319_2025_487_MOESM11_ESM.zip › Figure 8/8E/PD21 Brca1 vKO-Aldh2 KO PLZF&GFRa1/PD21 Brca1 vKO-Aldh2 KO testis anti-GFRa1.tif]

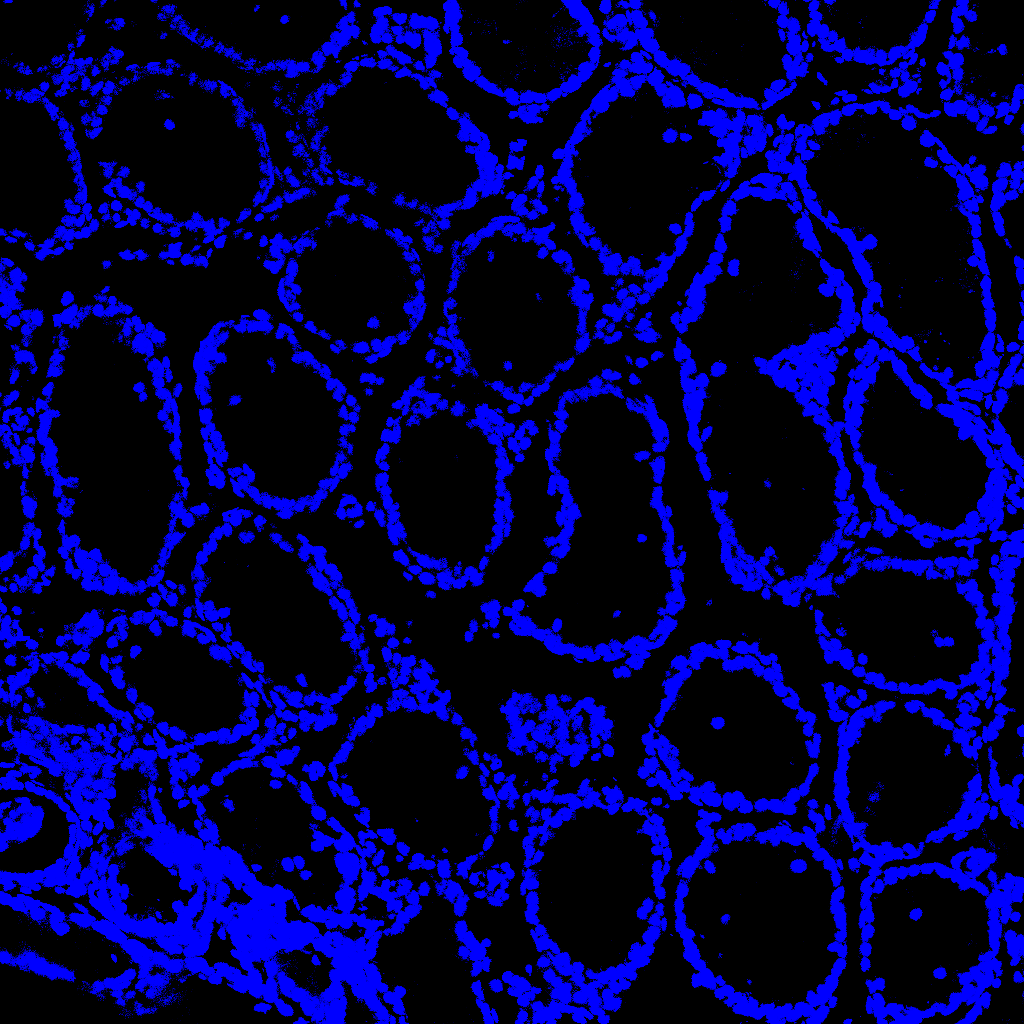

Supplement: Supplementary file 11 — Source data Fig. 8 [file 44319_2025_487_MOESM11_ESM.zip › Figure 8/8E/PD21 Brca1 vKO-Aldh2 KO PLZF&GFRa1/PD21 Brca1 vKO-Aldh2 KO testis anti-PLZF&GFRa1 Hoechst.tif]

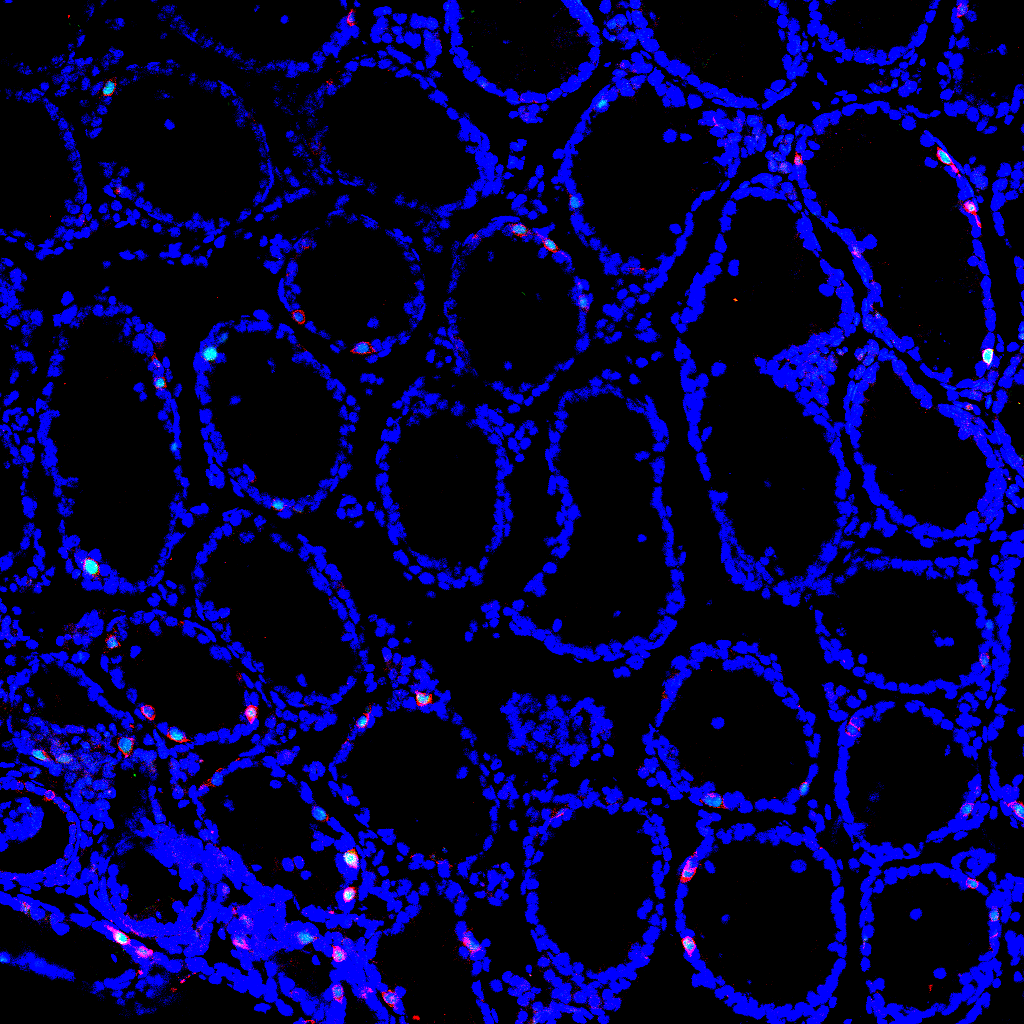

Supplement: Supplementary file 11 — Source data Fig. 8 [file 44319_2025_487_MOESM11_ESM.zip › Figure 8/8E/PD21 Brca1 vKO-Aldh2 KO PLZF&GFRa1/PD21 Brca1 vKO-Aldh2 KO testis anti-PLZF&GFRa1 Hoechst_overlay.tif]

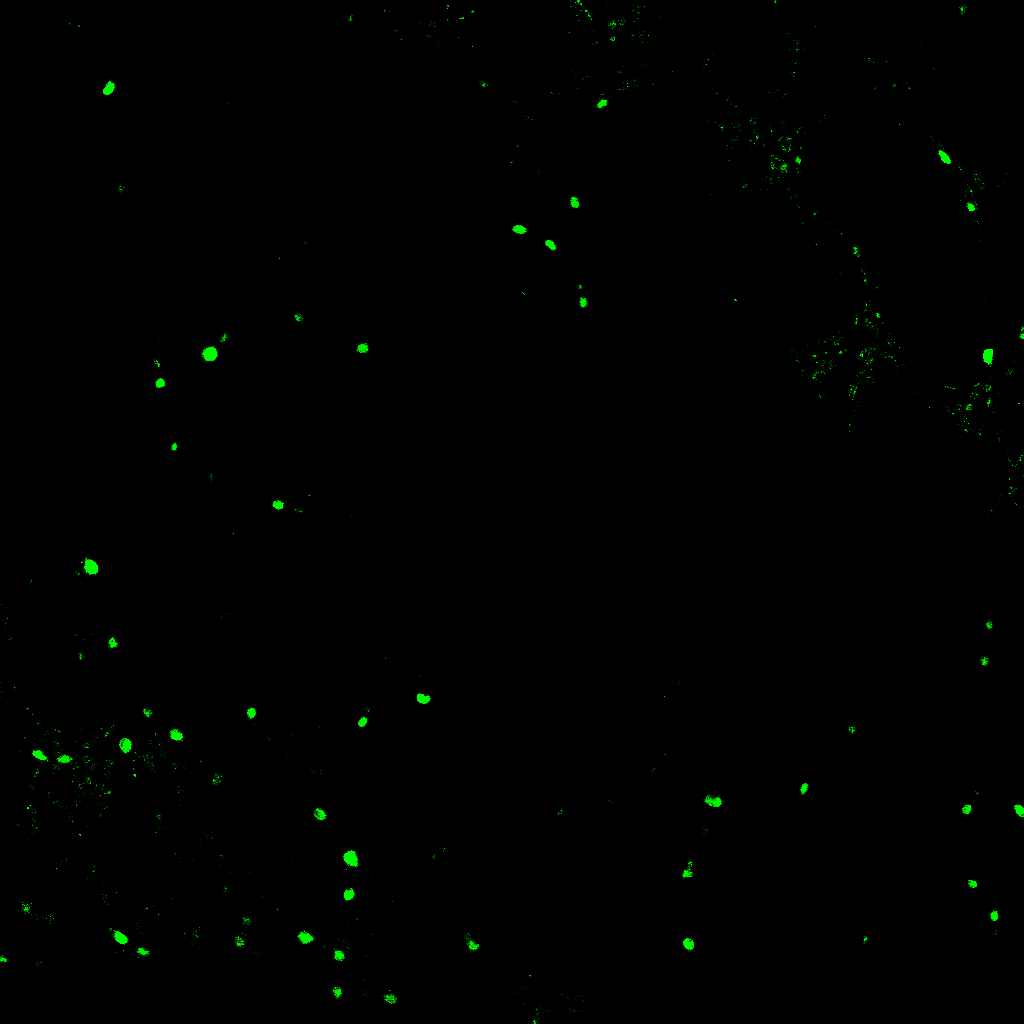

Supplement: Supplementary file 11 — Source data Fig. 8 [file 44319_2025_487_MOESM11_ESM.zip › Figure 8/8E/PD21 Brca1 vKO-Aldh2 KO PLZF&GFRa1/PD21 Brca1 vKO-Aldh2 KO testis anti-PLZF.tif]

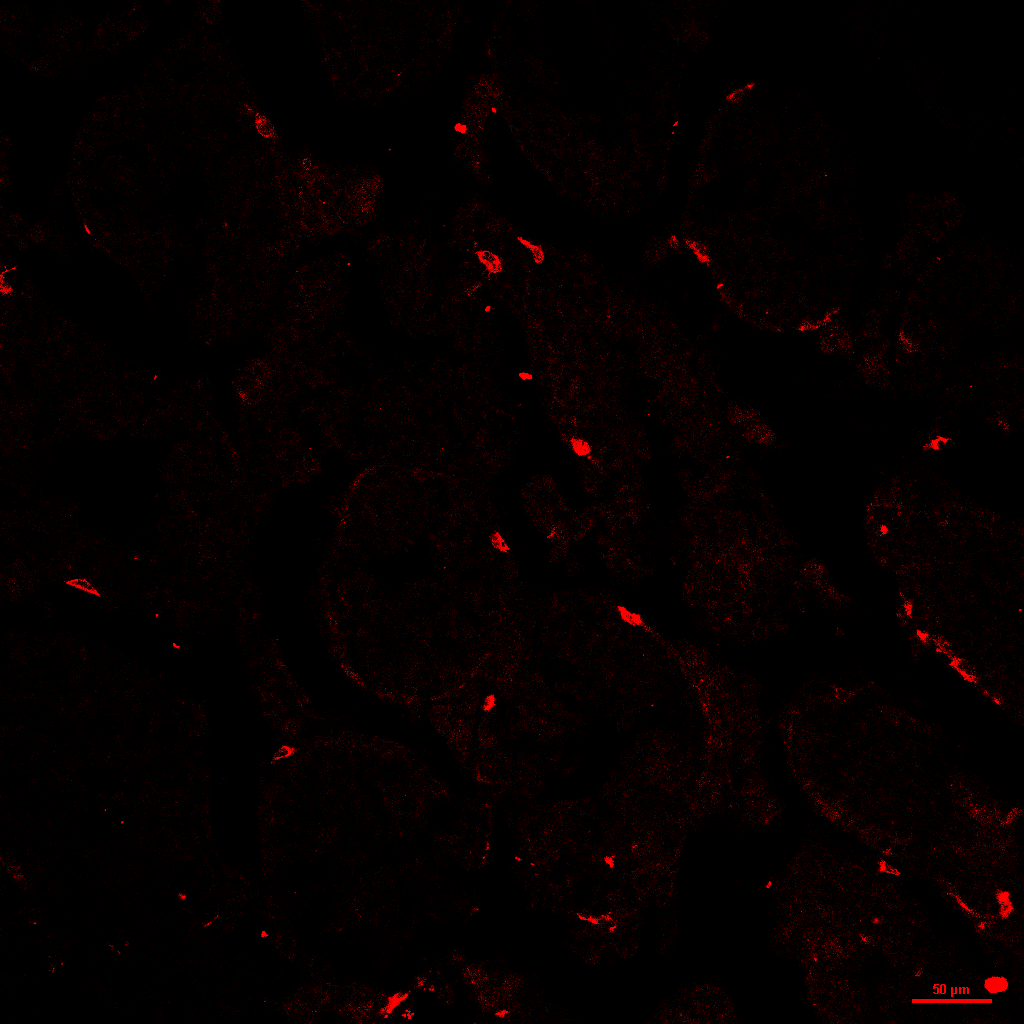

Supplement: Supplementary file 11 — Source data Fig. 8 [file 44319_2025_487_MOESM11_ESM.zip › Figure 8/8E/PD21 WT PLZF&GFRa1/PD21 WT testis anti-GFRa1.tif]

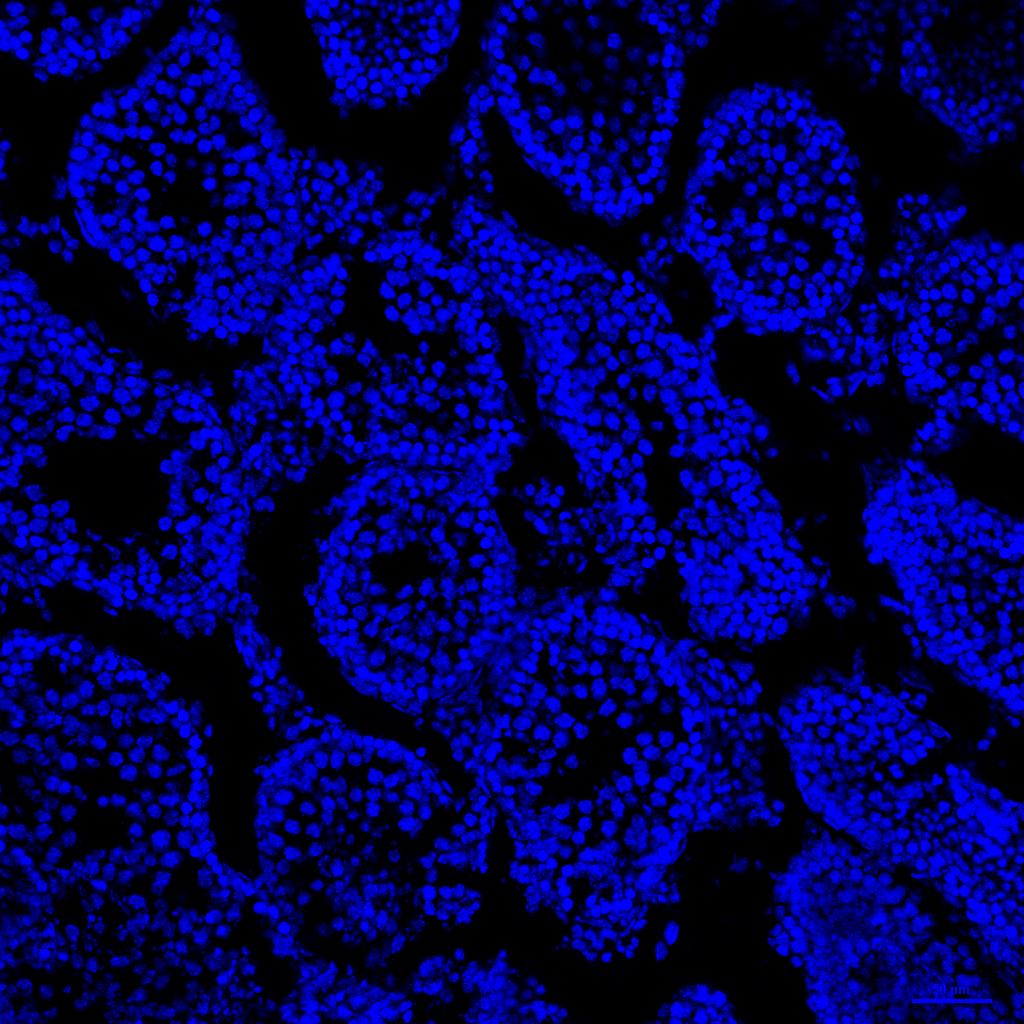

Supplement: Supplementary file 11 — Source data Fig. 8 [file 44319_2025_487_MOESM11_ESM.zip › Figure 8/8E/PD21 WT PLZF&GFRa1/PD21 WT testis anti-PLZF&GFRa1 Hoechst.tif]

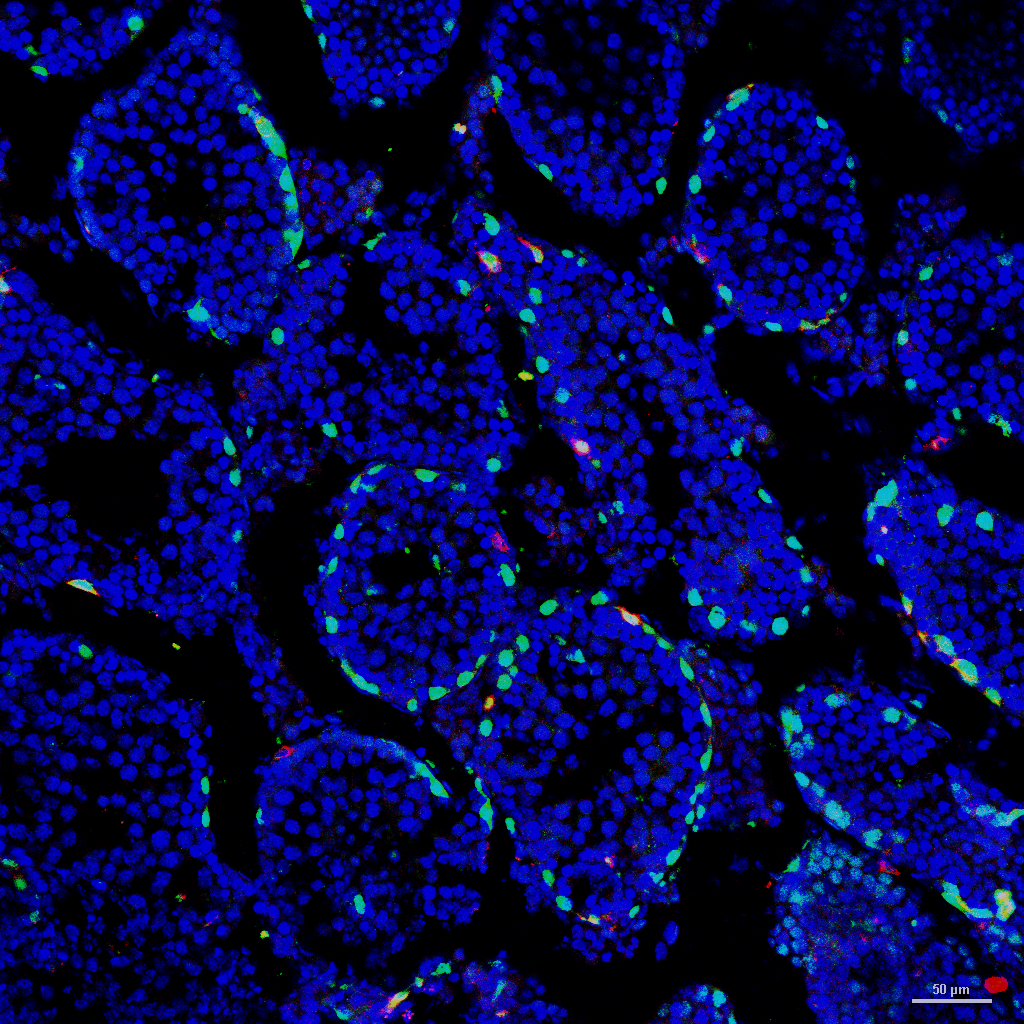

Supplement: Supplementary file 11 — Source data Fig. 8 [file 44319_2025_487_MOESM11_ESM.zip › Figure 8/8E/PD21 WT PLZF&GFRa1/PD21 WT testis anti-PLZF&GFRa1 Hoechst_overlay.tif]

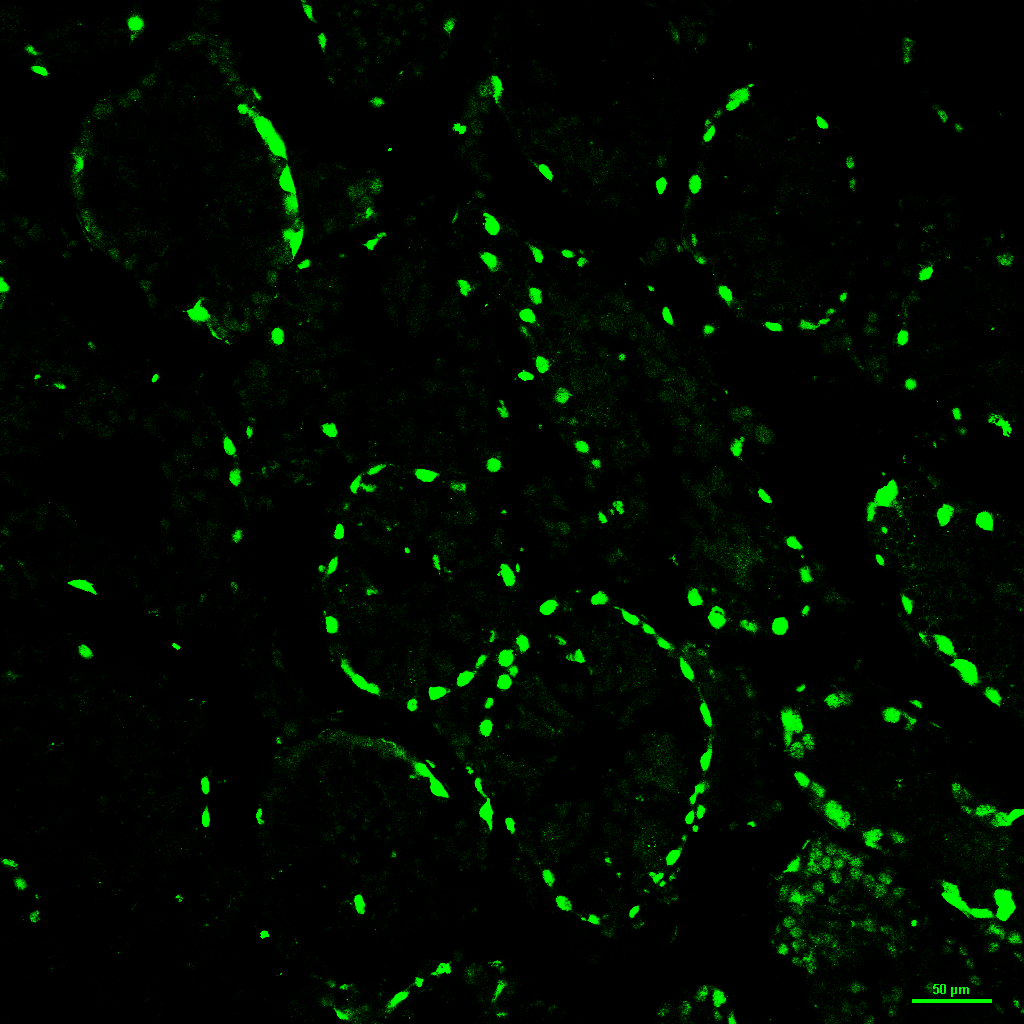

Supplement: Supplementary file 11 — Source data Fig. 8 [file 44319_2025_487_MOESM11_ESM.zip › Figure 8/8E/PD21 WT PLZF&GFRa1/PD21 WT testis anti-PLZF.tif]
