# Supplementary material for: BRCA1 preserves genome integrity during the formation of undifferentiated spermatogonia
Source: EMBO Rep. 2025 May 28;26(15):3747–72. doi: 10.1038/s44319-025-00487-5 (PMC12332178; doi:10.1038/s44319-025-00487-5)
Supplement: Supplementary file 12 — Expanded View Figures [file 44319_2025_487_MOESM12_ESM.pdf]

Expanded View Figures

Figure EV1. Spermatogonial stem cells and progenitor spermatogonia achieve homeostasis when undifferentiated spermatogonia is formed after birth.

(**A**) Cell identity and marker gene expression before and after the formation of undifferentiated spermatogonia, which contain spermatogonial stem cells and progenitor spermatogonia that achieve homeostasis. (**B**) IF staining of PLZF and GCNA in frozen sections of testes from WT mice at E18.5 and PD1. (**C**) IF staining of GFRα1 and PLZF in frozen sections of testes from WT mice at PD1, PD4, PD7, PD14, and PD21. (**D**) IF staining of SOX3 and PLZF in frozen sections of testes from WT mice at PD1, PD4, PD7, PD14, and PD21. (**E**) Statistical analysis of the percentage of GFRα1+ and SOX3+ cells among PLZF+ cells in testes from WT mice at PD1, PD4, PD7, PD14, and PD21. Data information: Data are presented as mean ± SD. Three mice at each timepoint were analyzed. Scale bar, 50 μm. n.s., not significant.

 © The Author(s)

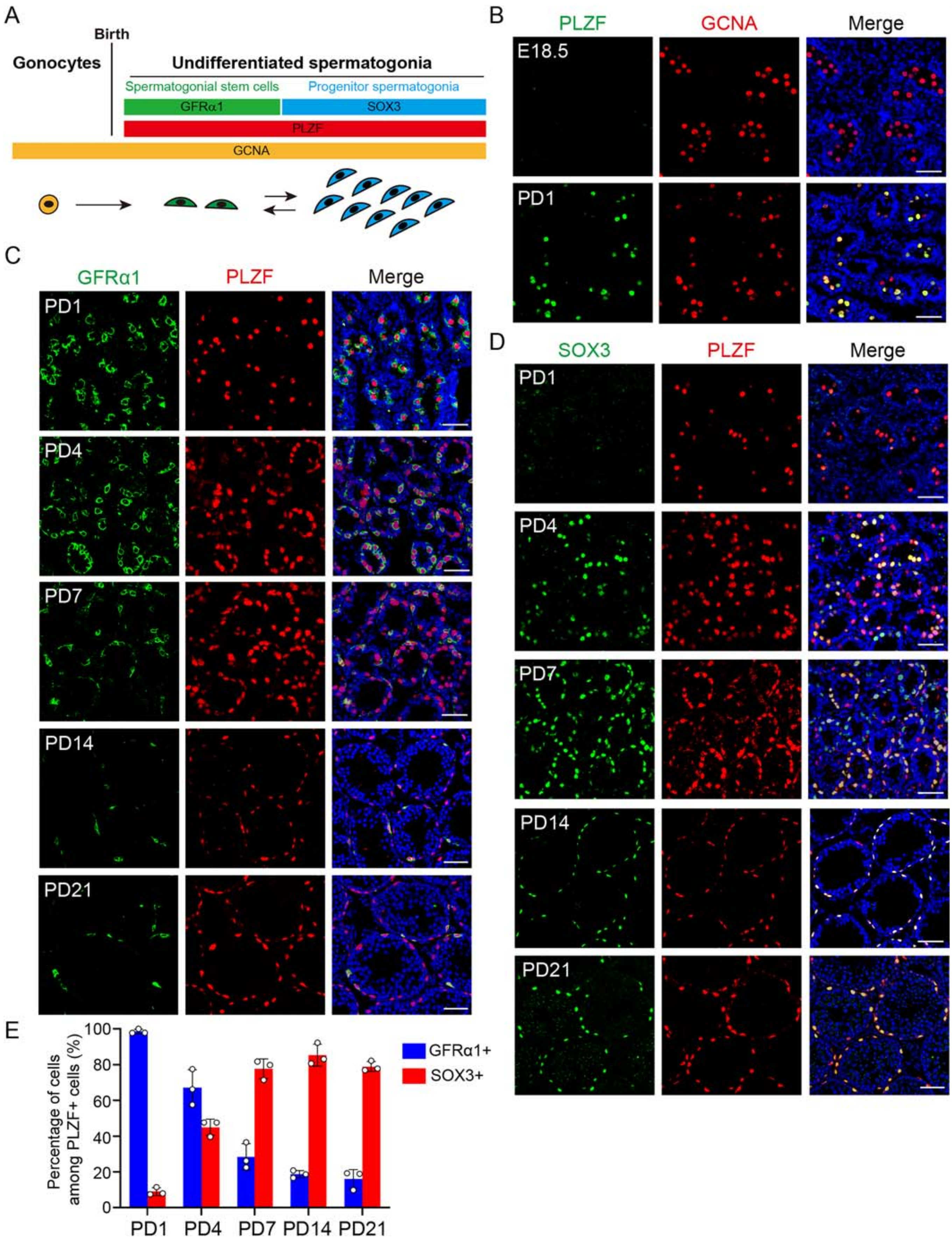
A
Birth
Gonocytes
Undifferentiated spermatogonia
Spermatogonial stem cells
Progenitor spermatogonia
GFRα1
SOX3
PLZF
GCNA
B
PLZF
GCNA
Merge
E18.5
PD1
C
GFRα1
PLZF
Merge
PD1
PD4
PD7
PD14
PD21
D
SOX3
PLZF
Merge
PD1
PD4
PD7
PD14
PD21
E
Percentage of cells among PLZF+ cells (%)
100
80
60
40
20
0
PD1 PD4 PD7 PD14 PD21
GFRα1+
SOX3+

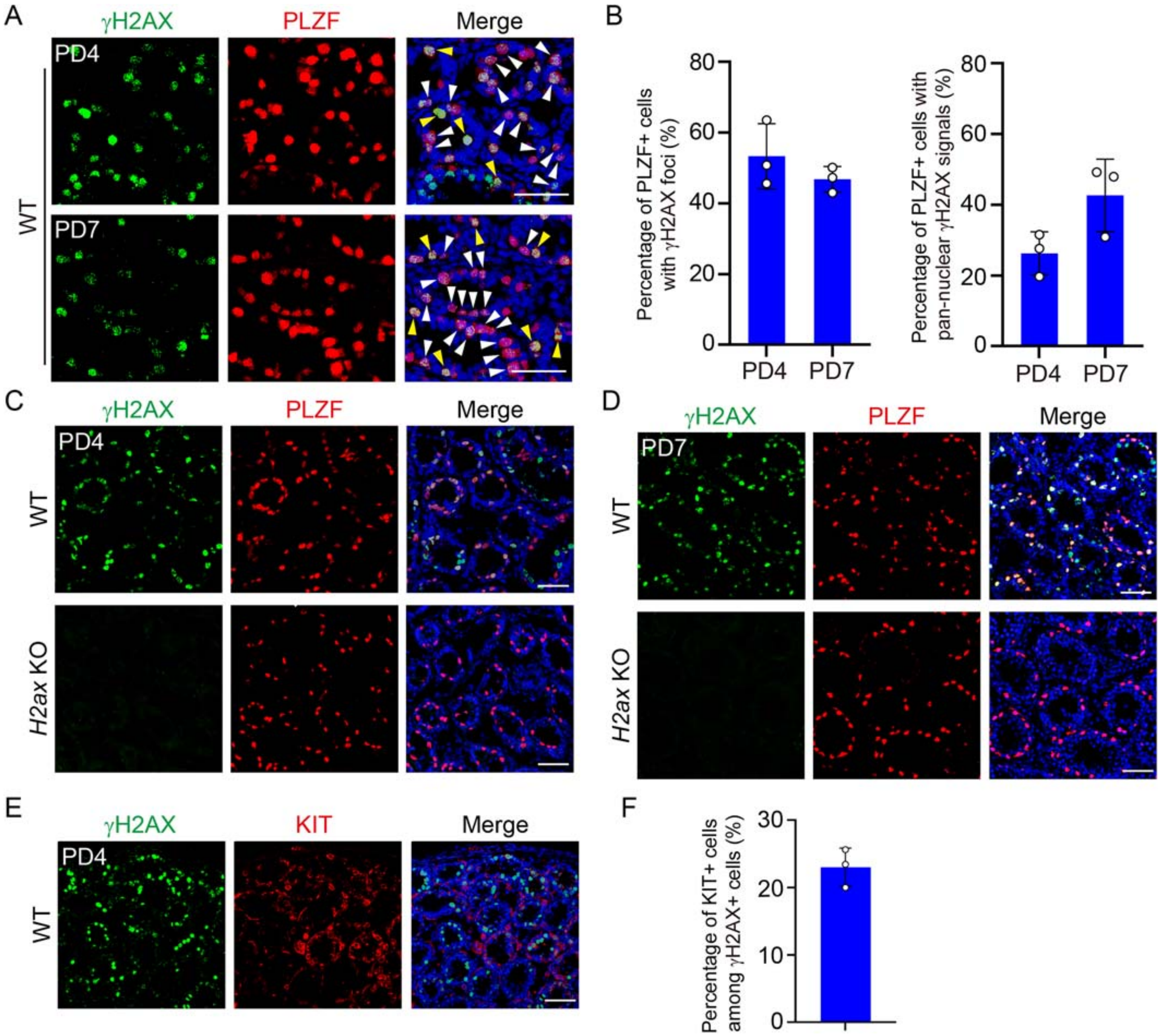

Figure EV2. γH2AX signals are not observed during the formation of undifferentiated spermatogonia in *H2ax* KO mice.

(**A**) Representative images of γH2AX and PLZF staining in frozen sections of testes from WT mice at PD4 and PD7. PLZF+ cells with γH2AX+ foci were labeled by white arrowhead and PLZF+ cells with pan-nuclear γH2AX+ signals were labeled by yellow arrowhead. (**B**) Statistical analysis of the percentage of PLZF+ cells with γH2AX+ foci or with pan-nuclear γH2AX+ signals in WT mice at PD4 and PD7. (**C**, **D**) IF staining of γH2AX and PLZF in frozen sections of testes from WT and *H2ax* KO mice at PD4 (**C**) and PD7 (**D**). (**E**, **F**) IF staining of γH2AX and KIT in frozen sections of testes from WT mice at PD4 and statistical analysis of the percentage of KIT+ cells among γH2AX+ cells. Data information: Data are presented as mean ± SD. 3 mice were analyzed. Scale bar, 50 µm.

 © The Author(s)

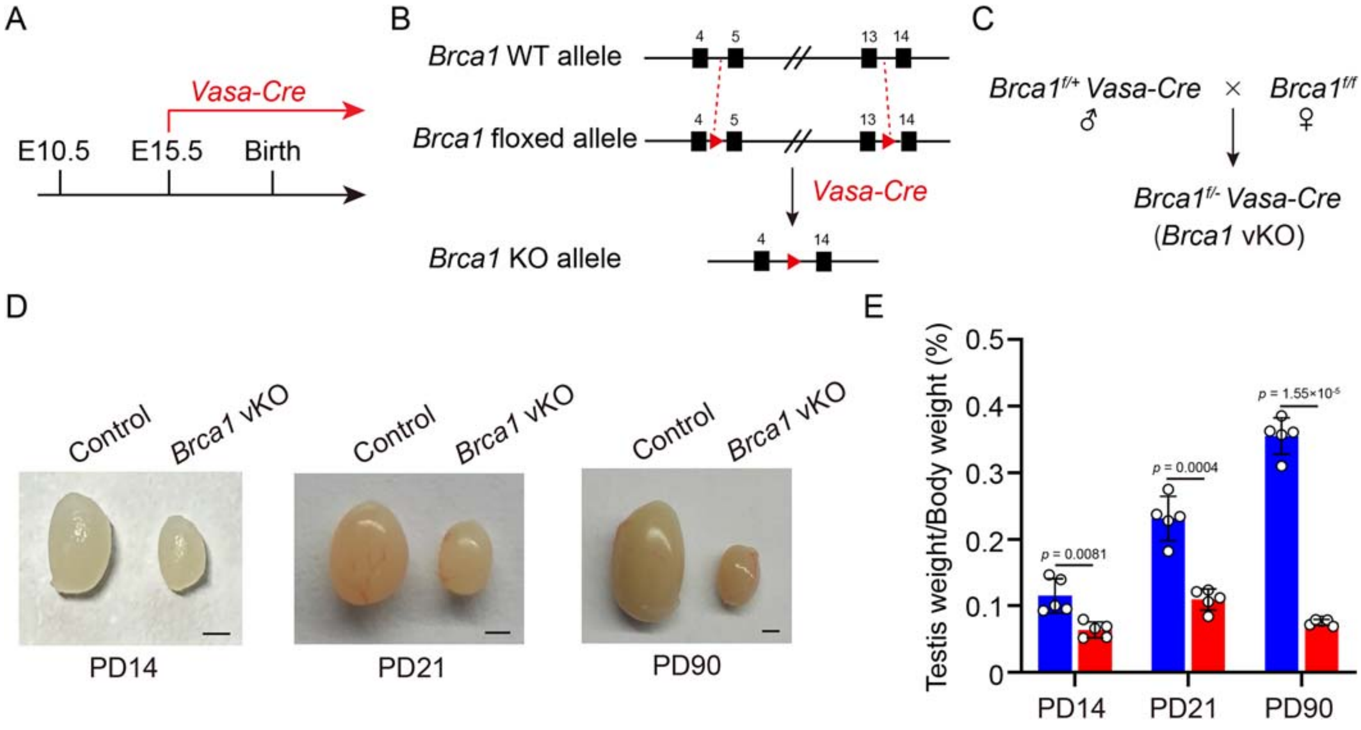

Figure EV3. Generation of *Brca1* germ cell-specific KO mice.

(**A**) Schematic diagram showing the expression timepoint of *Vasa-Cre* during mouse germ cell development. (**B**) Schematic illustrations of *Vasa-Cre* recombinase-mediated deletion of *Brca1* floxed allele. (**C**) Schematic illustrations of mating strategies to obtain *Brca1* vKO mice. (**D**) Representative images of testes from Control and *Brca1* vKO male mice at PD14, PD21, and PD90. Scale bar, 1 mm. (**E**) Statistical analysis of ratios of testis weight to body weight of Control and *Brca1* vKO male mice at PD14, PD21, and PD90. 5 mice of each genotype were analyzed. Data information: Data are presented as mean ± SD. *P* value, two-tailed unpaired Student's *t* test.

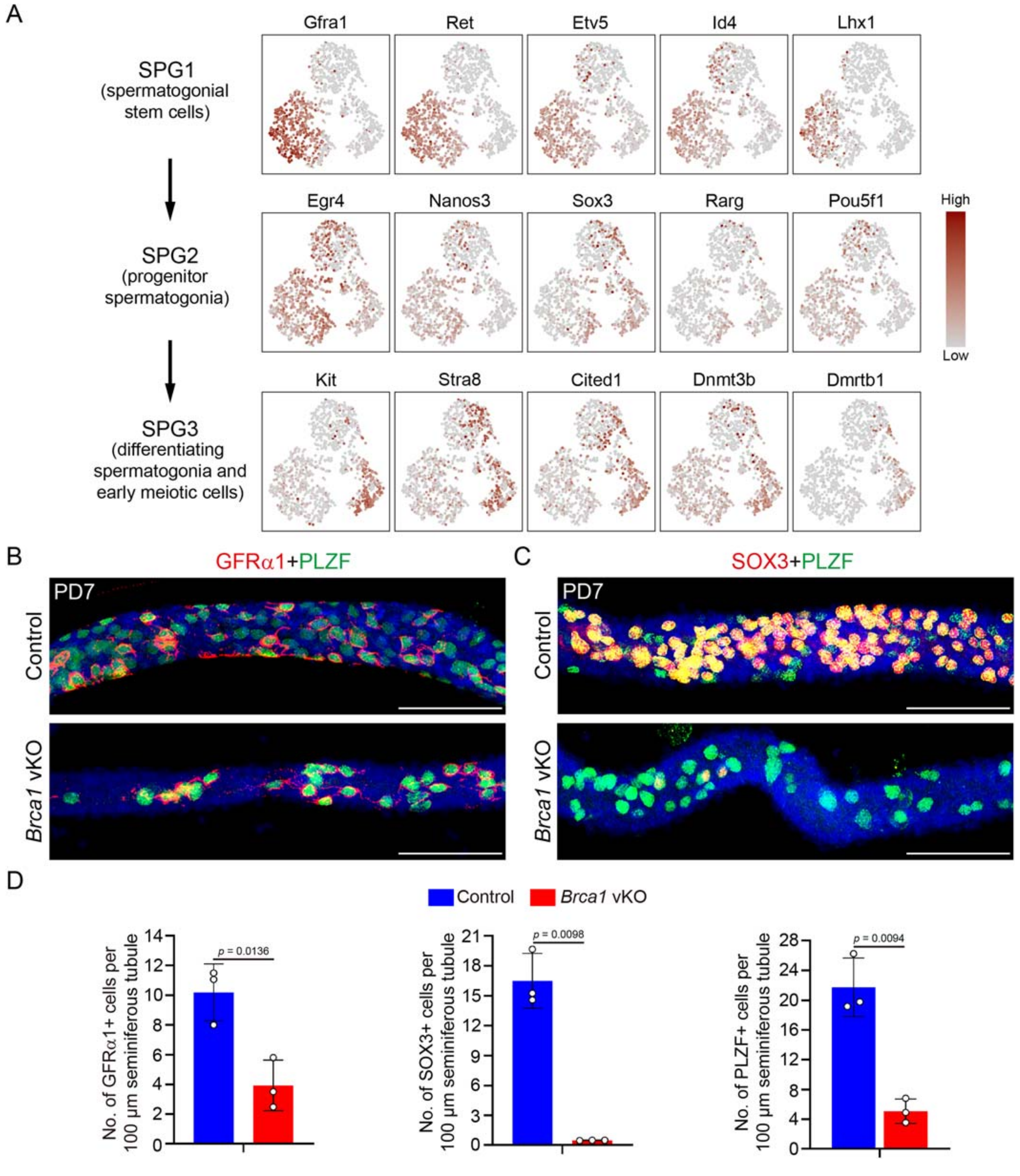
A
SPG1
(spermatogonial stem cells)
SPG2
(progenitor spermatogonia)
SPG3
(differentiating spermatogonia and early meiotic cells)
Gfra1
Ret
Etv5
Id4
Lhx1
Egr4
Nanos3
Sox3
Rarg
Pou5f1
Kit
Stra8
Cited1
Dnmt3b
Dmrtb1
High
Low
B
GFRα1+PLZF
C
SOX3+PLZF
PD7
Control
Brca1 vKO
D
Control
Brca1 vKO
No. of GFRα1+ cells per 100 µm seminiferous tubule
p = 0.0136
No. of SOX3+ cells per 100 µm seminiferous tubule
p = 0.0098
No. of PLZF+ cells per 100 µm seminiferous tubule
p = 0.0094

 © The Author(s)

Figure EV4. Progenitor spermatogonia have a much more dramatic reduction than spermatogonial stem cells after BRCA1 loss.

(**A**) Gene expression patterns of selected marker genes for each subtype of germ cells. The tSNE plot is in reference to all germ cells (control + *Brca1* vKO). (**B**) Whole-mount IF staining of GFRα1 and PLZF in seminiferous tubules of testes from Control and *Brca1* vKO male mice at PD7. (**C**) Whole-mount IF staining of SOX3 and PLZF in seminiferous tubules of testes from Control and *Brca1* vKO male mice at PD7. (**D**) Statistical analysis of the number of GFRα1 + , SOX3+cells, and PLZF+ cells per 100 μm seminiferous tubule in Control and *Brca1* vKO male mice at PD7. Three mice of each genotype were analyzed. Data information: Data are presented as mean ± SD. *P* value, two-tailed unpaired student's t test. Scale bar, 100 μm.

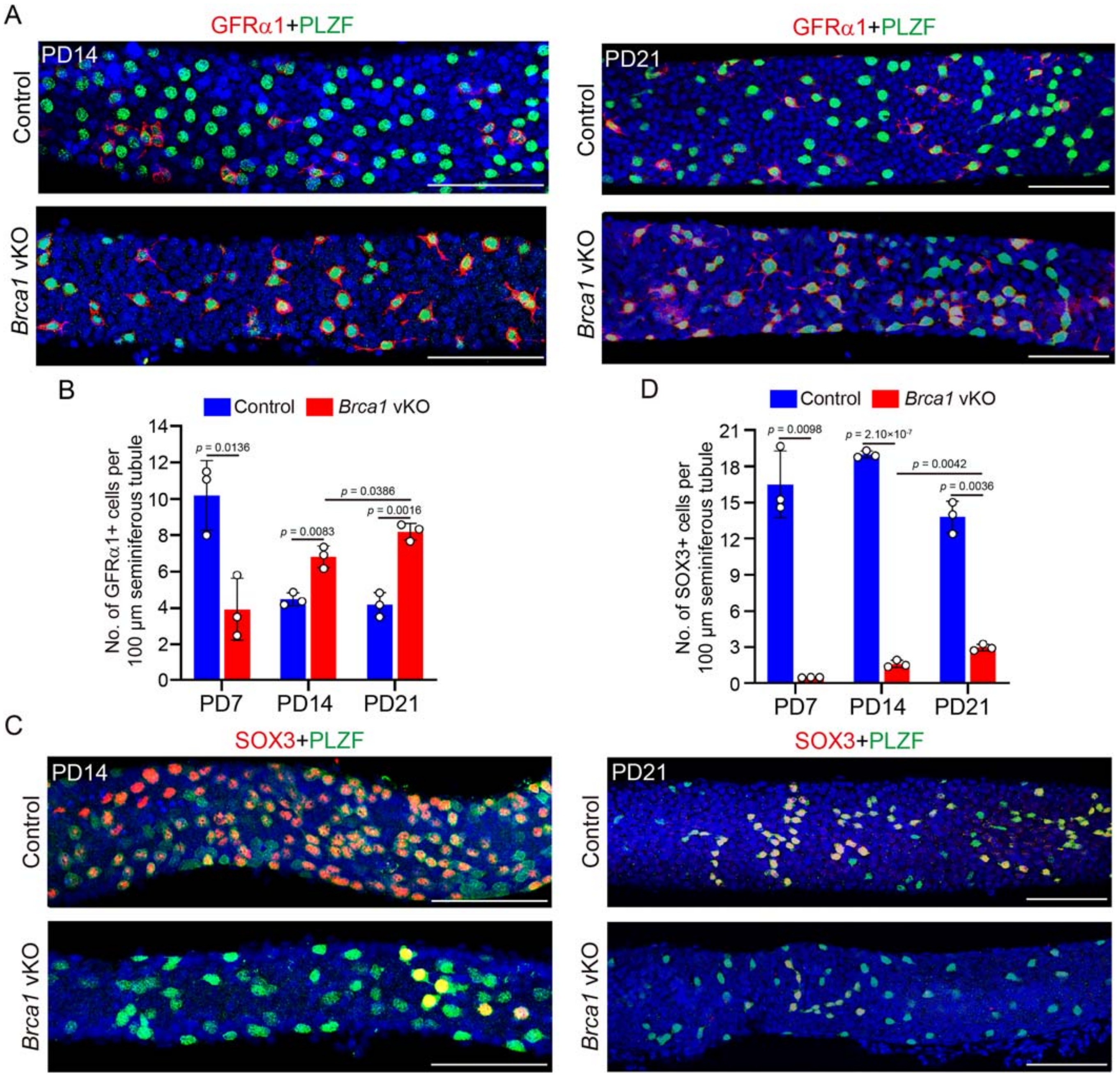

Figure EV5. The number of spermatogonial stem cells gradually increases in *Brca1* vKO testes.

(**A**) Whole-mount IF staining of GFRα1 and PLZF in seminiferous tubules of testes from Control and *Brca1* vKO male mice at PD14 and PD21. (**B**) Statistical analysis of the number of GFRα1+ cells per 100 μm seminiferous tubule in testes from Control and *Brca1* vKO male mice at PD7, PD14, and PD21. Three mice of each genotype were analyzed. (**C**) Whole-mount IF staining of SOX3 and PLZF in seminiferous tubules of testes from Control and *Brca1* vKO male mice at PD14 and PD21. (**D**) Statistical analysis of the number of SOX3+ cells per 100 μm seminiferous tubule in testes from Control and *Brca1* vKO male mice at PD7, PD14, and PD21. Three mice of each genotype were analyzed. Data information: Data are presented as mean ± SD. *P* value, two-tailed unpaired Student's *t* test. Scale bar, 100 μm.

 © The Author(s)
